# How Male and Female Literary Authors Write About Affect Across Cultures and Over Historical Periods

**DOI:** 10.1007/s42761-023-00219-9

**Published:** 2023-09-05

**Authors:** Giada Lettieri, Giacomo Handjaras, Erika Bucci, Pietro Pietrini, Luca Cecchetti

**Affiliations:** 1 Crossmodal Perception and Plasticity Laboratory, Institute of Research in Psychology & Institute of Neuroscience, Université Catholique de Louvain, Louvain-La-Neuve, Belgium; 2 Social and Affective Neuroscience Group, MoMiLab, IMT School for Advanced Studies Lucca, Lucca, Italy; 3 Molecular Mind Laboratory, MoMiLab, IMT School for Advanced Studies Lucca, Lucca, Italy

**Keywords:** Literature, Affect, Sex differences, Valence, Arousal, Cross-cultural

## Abstract

A wealth of literature suggests the existence of sex differences in how emotions are experienced, recognized, expressed, and regulated. However, to what extent these differences result from the put in place of stereotypes and social rules is still a matter of debate. Literature is an essential cultural institution, a transposition of the social life of people but also of their intimate affective experiences, which can serve to address questions of psychological relevance. Here, we created a large corpus of literary fiction enriched by authors’ metadata to measure the extent to which culture influences how men and women write about emotion. Our results show that even though before the twenty-first century and across 116 countries women more than men have written about affect, starting from 2000, this difference has diminished substantially. Also, in the past, women’s narratives were more positively laden and less arousing. While the difference in arousal is ubiquitous and still present nowadays, sex differences in valence vary as a function of culture and have dissolved in recent years. Altogether, these findings suggest that historic evolution is associated with men and women writing similarly about emotions and reveal a sizable impact of culture on the affective characteristics of the lexicon.

**Supplementary Information:**

The online version contains supplementary material available at 10.1007/s42761-023-00219-9.

In contemporary societies worldwide, biological sex lies at the basis of *gender roles*, namely the put in place of behaviors, beliefs, personality characteristics, attitudes, values, and emotions associated with being man or woman (Eagly et al., 2000). Of note, social role theory explains how acting in accordance with these roles and rules confirms stereotypes and segregates individuals in following gender norms (Eagly, 1987).

Concerning affect, a wealth of literature shows that men and women differ statistically in how emotions are experienced (Bradley et al., 2001; Johnson & Whisman, 2013; Maffei & Angrilli, 2019; Marchewka et al., 2014), recognized (Greenberg et al., 2023; Kret & De Gelder, 2012), expressed (LaFrance et al., 2003; McDuff et al., 2017), or regulated (Goubet & Chrysikou, 2019; Nolen-Hoeksema, 2012), as well as in how the brain (Filkowski et al., 2017; Stevens & Hamann, 2012; Whittle et al., 2011) or the body (Deng et al., 2016; Williams et al., 2019) react to emotionally laden stimuli. Although these findings have often been interpreted in the framework of biological reductionism (e.g., Baron-Cohen, 2002; Brizendine, 2006), it is worth noting that recent investigations indicate that the biological dimorphism hypothesis fails to account for gender differences even when the matter of investigation is the brain (Eliot et al., 2021). Also, a relevant amount of studies have been advocating for the strong role that sociocultural factors play in shaping how men and women react and express their emotions. For instance, the existence of stereotypes associated with emotional experiences is not only present in adults (Plant et al., 2000), and influences affective responses (Grossman & Wood, 1993), but arises as early as three years of age (Haugh et al., 1980). Moreover, context plays a crucial role in how the two genders differently experience and express emotions (Barrett et al., 1998; Fivush et al., 2000).

Nevertheless, to what extent sex differences result from the put in place of stereotypes and social rules is still a matter of debate in several fields of psychological science (Bijlstra et al., 2019; Breda et al., 2020; Hsu et al., 2021; Korb et al., 2023; Neel et al., 2012).

Cross-cultural investigations are particularly suited to add to the debate on the origins of gender differences. Indeed, the existence of large cultural variations is often considered proof of the primacy of societal over biological factors, with the exception of the paradoxical increase of gender differences in more equal countries (Stoet & Geary, 2018; but see also Richardson et al., 2020 and Breda et al., 2020). The cross-cultural approach has contributed to the study of gender differences in emotional aspects of non-verbal behavior. For instance, McDuff and associates (2017) report that females tend to smile more than males across 12 countries. However, they also report that the difference between men and women in the furrowing of the brow is more pronounced in more individualist societies. In the context of how emotions are experienced, a study by Fischer and colleagues (Fischer et al., 2004), conducted across 37 countries, shows that the difference in intensity ratings of anger and disgust between men and women do not vary as a function of culture, whereas men from more gender-equal countries report lower intensity scores of fear, sadness, shame, and guilt, as compared to men from less gender-equal nations.

Regarding the relationship between emotion and language, most of what we know about gender differences comes from monocultural studies. In English-speaking samples, researchers have found that, while in pre-schoolers, there are no differences between boys and girls in the use of emotion words during peer interactions (Fabes et al., 2001), 6- (Tenenbaum et al., 2011) and 13-year-old girls (Aldrich & Tenenbaum, 2006) use more emotion labels than boys when interacting with others. Also, a recent investigation on social media content in Chinese individuals shows that women write more positively laden words than men (Feng & Ivanov, 2022), a finding that is confirmed by the analysis of Wikipedia editors’ comments in English (Gallus & Bhatia, 2020). However, one interesting aspect of studying the affective lexicon to understand the nature of gender differences in emotion is that it can be studied as a function of space (i.e., in relation to various cultures) and time (i.e., in relation to societal evolution). This applies to written texts in general and to literature and narratives in particular, as they exist since 2,000 BC (Kovacs, 1989) and represent a fundamental cultural institution in modern and contemporary societies worldwide (Taine, 2019; Wellek & Warren, 1956), reflecting the condition of men and women. Of note, novels represent a window into the intimate psychological life of individuals and are powerful tools to express a gamut of feelings that readers from different cultures and times appreciate.

Up to now, the cross-cultural study of language has already provided insights into how valence explains lexical evolution rates (Jackson et al., 2023) and into the meaning of emotional terms (Jackson et al., 2019). One of the main limitations of applying the same approach to the study of gender differences (Grayson et al., 2017; Twenge et al., 2012) is that large collections of texts lack comprehensive metadata (Pechenick et al., 2015). Here, we create a large corpus of literature, including the author’s sex and nationality, among other metadata, to explore how men and women write about emotion over historical periods and across different cultures.

## Method

### Composition of the Corpus

**Fig. 1:**
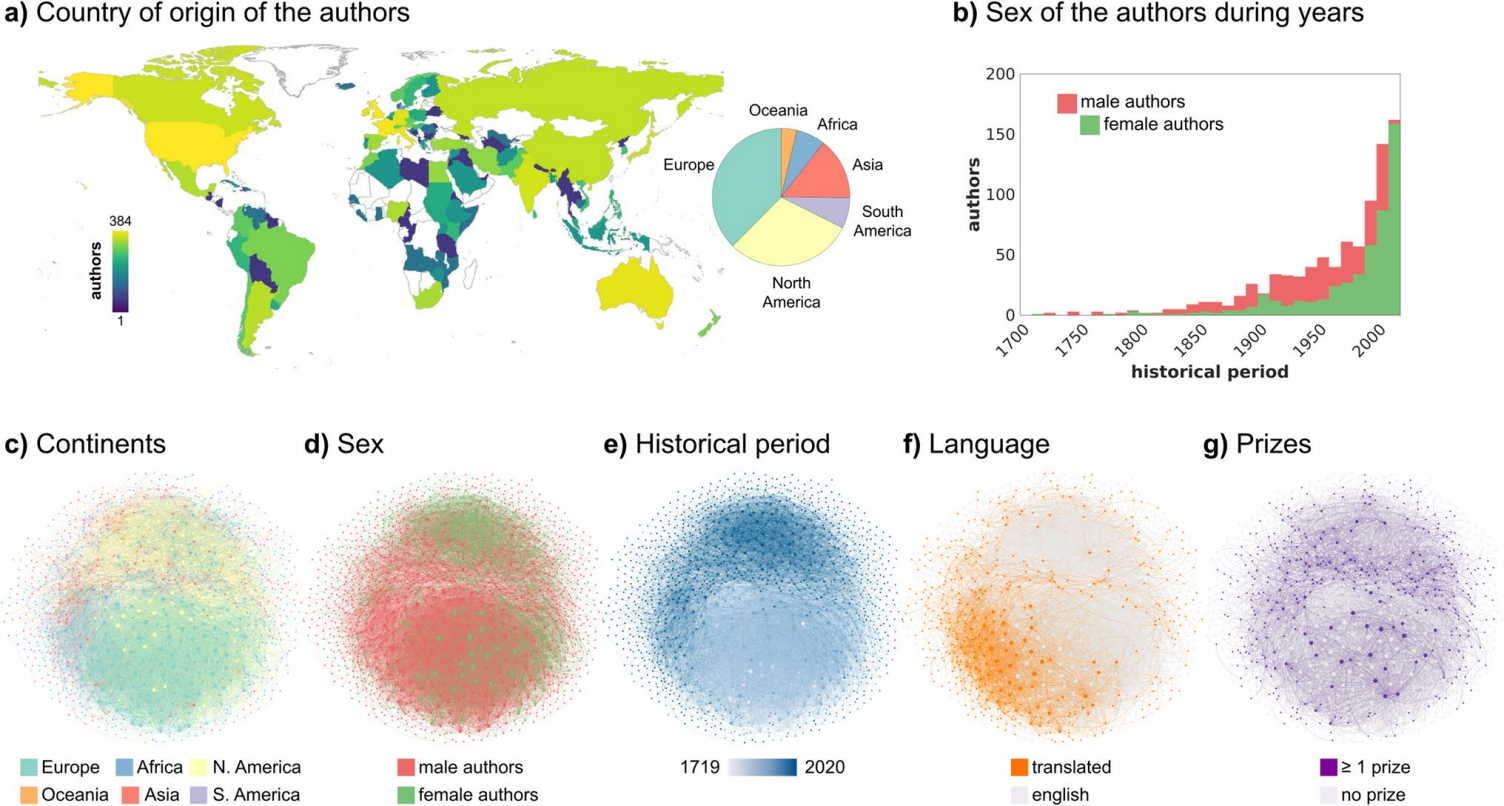
Corpus composition. In panel **a**, we report on the left the distribution of the 1,365 authors across countries, and, on the right, a pie-chart across continents. Panel **b** depicts the composition of author sex across time, with male authors in red and female writers in green. In panels **c**–**g**, we represent graphs resulting from the stylometric analysis, where each dot maps a specific author and edges connect authors with similar use of words. Panel **c** depicts the continent of origin of the authors with the same color coding as panel **a**. Author sex, historical period, language, and being awarded literary prizes are shown in panels **d** to **g**, respectively

We built a corpus of English-version novels, short stories, novellas, fables, and autobiographical narratives published between 1719 and 2020, with a total of 2,281 books (~ 245 M of words) by 1,365 authors (871 male and 494 female writers; Fig. 1; see Table S2 and Supplementary Information—SI). To classify an author as being a man or woman, we relied on their biological sex as reported by the available source of information. Also, to define the culture of origin, we considered the country in which they received their initial education and where they lived at the beginning of their life. The composition of our corpus maintained the actual proportion of the two sexes in currently published literature (Underwood et al., 2018). Since we collected from one to eight books from each author (average: 1.7 ± 1.2), we defined the “historical period” as the median of the years of publication across all published books from the same writer. In line with previous reports (e.g., Greco, 2013), the number of published books increased over time, with an exponential-like growth starting from the late nineties of the last century (Fig. 1b). Regarding the publication language, ~ 67% of the authors originally wrote their narratives in English, whereas ~ 33% were translated into English from other languages. Overall, the selection procedure ensured world coverage in terms of the author countries (i.e., 116 countries; Fig. 1a). Lastly, to avoid possible issues relative to the quality of the optical character recognition procedure (as some pointed out in the case of Google Books; Pechenick et al., 2015), we included only digital text-based versions of the books. Each book was then manually revised and all information not related to the narrative per se was discarded (see SI).

To summarize the influence of the author’s sex, country of origin, language, and the historical period in which they lived on writing style, we used chi-square χ^2^ test or Fisher’s exact test for contingency tables. The binomial test was employed to evaluate the unequal distribution of the sexes in published literature over time. Results for these tests are reported in SI (*Composition of the corpus*). By using the frequency of words as a writer-specific marker (Mosteller & Wallace, 1963), we explored differences between texts from different authors, assessing the impact of sex and cultural factors on word use. First, for each author, we estimated the rate per million words of 25,040 terms occurring in at least 10% of male or female writers. Then, the authors-by-words matrix was normalized using Positive Pointwise Mutual Information (Bullinaria & Levy, 2007) and cosine distance summarized the stylometric differences between authors. We rank-converted the cosine distance and transformed the obtained similarity matrix into a weighted and undirected adjacency matrix. Finally, a graph was created using the minimum-spanning-tree algorithm to obtain the network backbone with an arbitrary average degree of 20 (Hidalgo et al., 2007; Brain Connectivity Toolbox, Rubinov & Sporns, 2010; Gephi v0.9.7, Bastian et al., 2009; Fig. 1c-g).

### Revealing Sex Differences in Literature

**Fig. 2:**
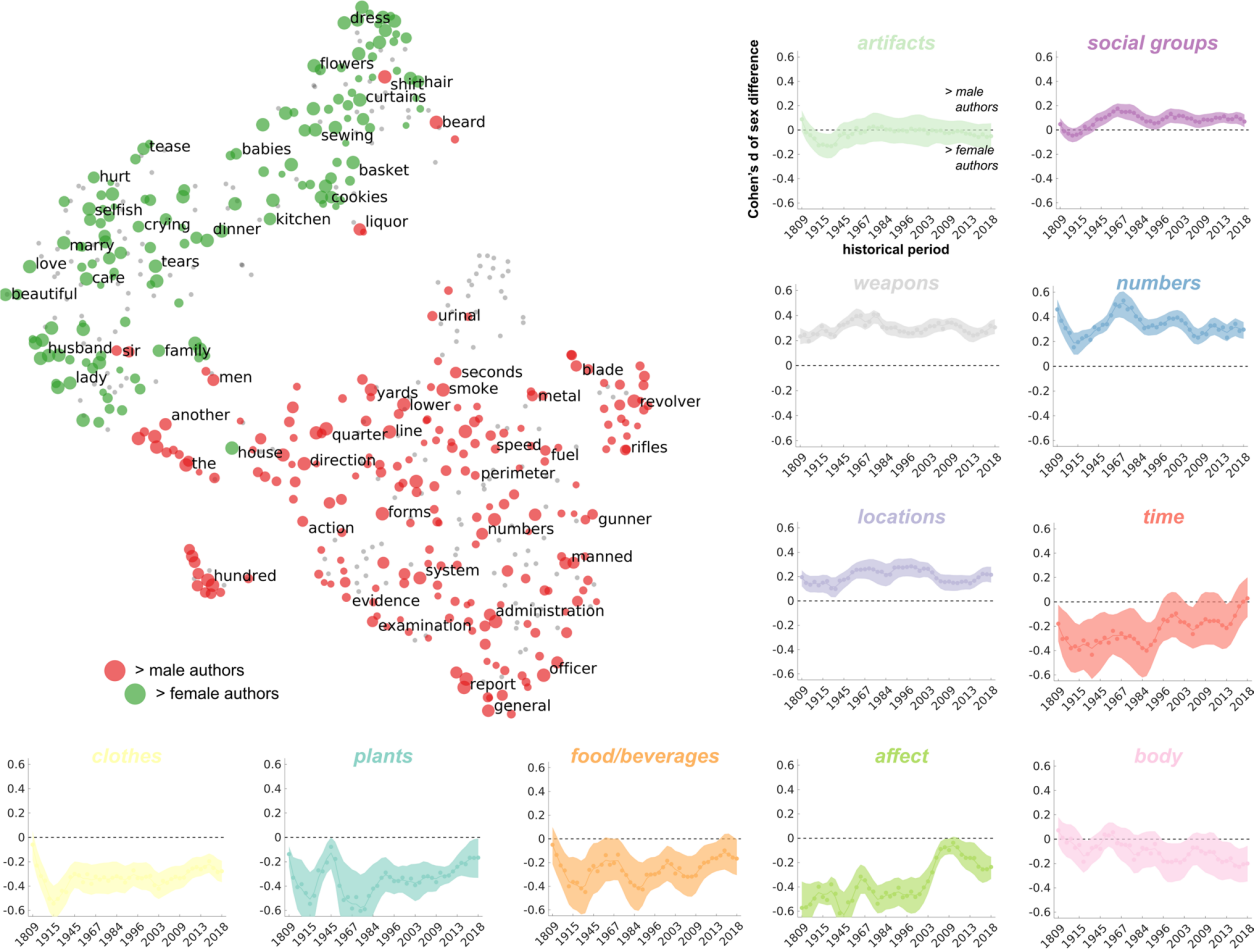
The distribution of sex differences in literature across semantic domains. Words demonstrating a significant *sex* effect are represented in a 2D t-SNE map obtained from word2vec word embeddings. Terms were grouped based on eleven semantic Wordnet domains. Each dot represents a word and is color-coded depending on whether it is used more frequently by male or female authors. In gray, words showing significant *sex* * *historical period* interaction effect. Graphs report sex differences (Cohens’d) over time across semantic domains. Negative values represent a higher frequency in females, whereas positive ones in males. Shaded color areas represent 95% confidence intervals, and the dashed black line marks gender equality in the use of terms. An interactive map of terms showing significant differences between the sexes, and their distribution in time and across countries is available at: https://www.sane-elab.eu/litemo/welcome.php

We built a general linear model (GLM) to predict the frequency of each of the 25,040 words as a function of the author sex, historical period, the interaction between sex and historical period, translation, and the continent of origin: *frequency* ~ *intercept* + *sex* + *historical period* + (sex * historical period) + *translation* + *continent*. For each word, statistical significance was determined through a partial F-test, comparing the full model against a nested simplified version of the model, where *sex* and sex * historical period were excluded, according to the following expression:

1 $$\mathrm{F}-\mathrm{statistic }= \frac{\frac{{RSS}_{nested} - {RSS}_{full}}{p}}{\frac{{RSS}_{full}}{n-k}}$$

where *RSS*_*nested*_ is the residual sum of squares of the simplified model, *RSS*_*full*_ is the residual sum of squares of the full model, *p* is the number of predictors removed from the full model, *n* is the number of authors, and *k* is the number of coefficients in the full model. Word frequency and historical period were rank transformed, and statistical significance for the effects of *sex* and sex * historical period was assessed through a non-parametric permutation test (*n* = 10,000) shuffling the author’s sex at each iteration. A generalized Pareto fit to the empirical permutation distribution provided a precise estimate of *p*-values (Winkler et al., 2016). Adjustment of statistical significance for multiple comparisons across 25,040 statistical tests was based on the Family-Wise Error correction (FWC; Nichols & Holmes, 2002; Winkler et al., 2014). Additionally, we tested the alternative model using country of origin instead of continent. Details and results of this analysis are reported in SI. Words passing the statistical significance were represented in a two-dimensional plane using word embeddings and t-distributed stochastic neighbor embedding (t-SNE; Van der Maaten & Hinton, 2008). First, we generated word embeddings associated with each word of our corpus (word2vec; alpha = .05, size = 512, window = 5, sample = 1e-3, training iterations = 10; Mikolov et al., 2013) and tested their quality against multiple benchmarks (Table S4). Word embeddings of the significant words in the full model (i.e., the one including *sex* and sex * historical period, *p*_FWC_ < .05, see Table S1), were correlated using the cosine distance to generate a dissimilarity matrix which was further reduced using a principal component analysis (66% of the explained variance). Then, t-SNE was applied (dimensions 2, perplexity 40, theta .1; Fig. 2, S3).

The results of the mapping procedure allowed us to represent near to each other words that had a high semantic relatedness. To further characterize sex differences in the semantic space of significant words, we clustered terms in eleven domains (i.e., *artifacts*, *social groups*, *weapons*, *body*, *affect*, *food/beverages*, *numbers*, *time*, *locations*, *clothes*, and *plants*; see Table S3) using WordNet 3.1 (Miller, 1995). In addition, for each semantic domain, differences between male and female writers (i.e., the absolute Cohen’s d of word frequency) were studied over time using a sliding window procedure (see SI).

### Sex Differences in the Affective Lexicon over Time and Across Cultures

**Fig. 3:**
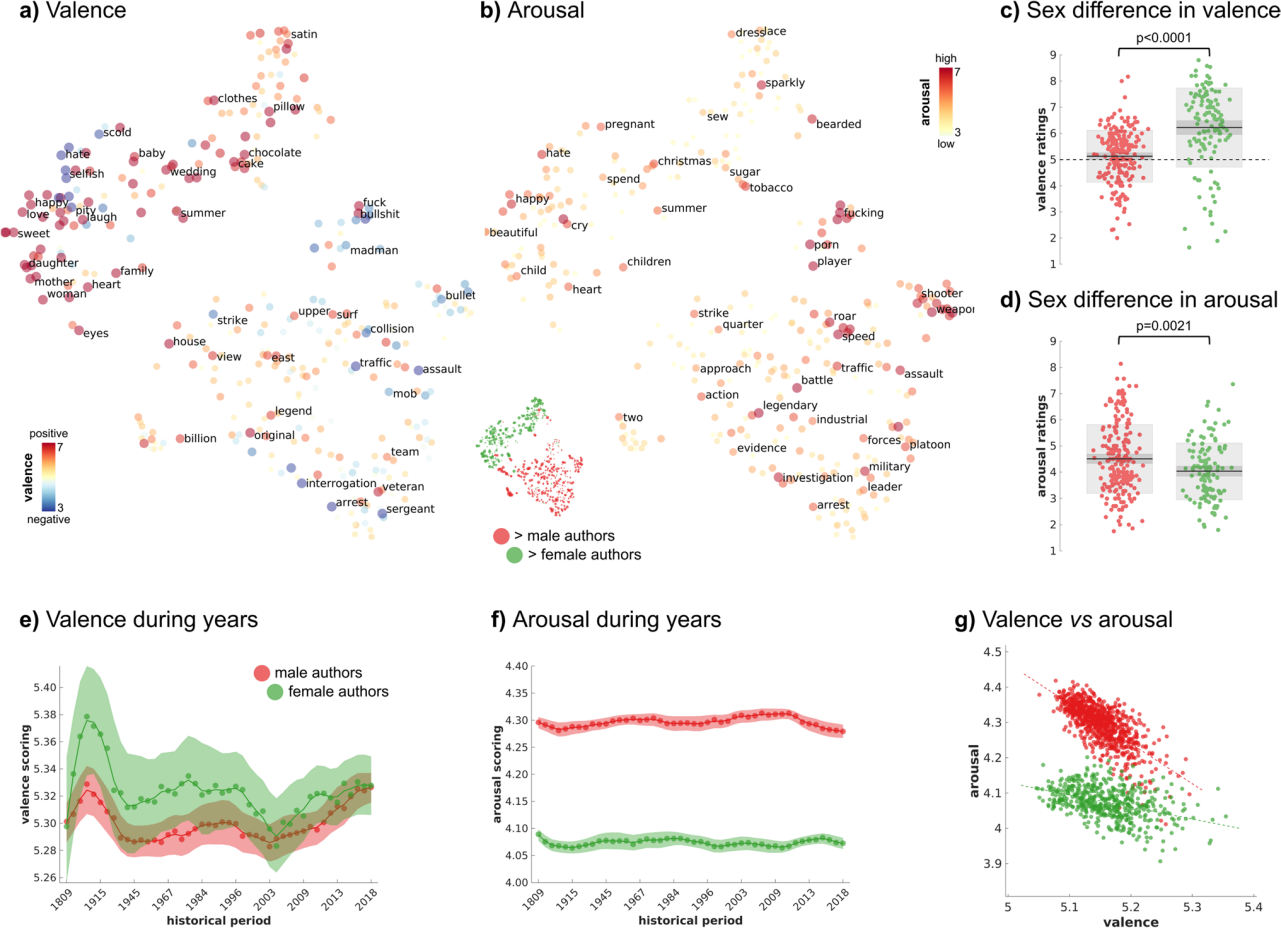
Valence and arousal of terms showing sex differences, and sentiment analysis of writings. Panels **a** and **b** map valence and arousal ratings (Warriner et al., 2013) of words showing a significant sex effect on the 2D t-SNE representation. Panels **c** and **d** show that female authors use more positive and less arousing terms. In box plots, each dot is a significant word also present in the Warriner database (*n* = 339), and the dark-gray shaded area shows the 95% confidence intervals of the SE of the mean, while the light-gray area is the SD. Panels **e** and **f** show the timecourse of valence and arousal in books by males and females computed on the entire Warriner database (*n* = 13,915). Shaded areas represent 95% confidence intervals of the estimation across authors. While sex differences in arousal remained stable across centuries, differences in valence have decreased over the last two decades. In panel **g**, we report the relation between affective dimensions across authors (i.e., each dot represents an author). The negative relationship between valence and arousal (i.e., writings with lower valence are also higher in arousal) is more pronounced in male as compared to female authors

To explore affective characteristics of words and how they relate to sex differences, we relied on the dataset provided by Warriner and colleagues (Warriner et al., 2013), who collected behavioral ratings of ~ 14,000 English lemmas from male and female individuals, scoring valence (i.e., the pleasantness elicited by a term) and arousal (i.e., the intensity of an emotion provoked by a term). In brief, after removing a set of diachronic terms (see SI), we identified words showing significant *sex* or *sex* * *historical period* effects in Warriner’s dataset. We divided these terms into two groups based on the directionality of the *sex* effect (i.e., higher frequency in males or in females). Then, for each word, we obtained affective ratings from either male or female individuals depending on its frequency of use (e.g., male affective norms for words more frequently used by male authors). Lastly, we mapped scores of the affective dimensions onto the t-SNE representation (Fig. 3a, b) and performed a Wilcoxon rank-sum test to measure whether a different frequency in word use between the two sexes also reflected a discrepancy in valence and arousal (Fig. 3c, d).

**Fig. 4:**
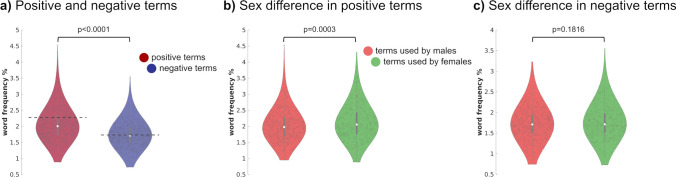
Positive and negative terms in male and female authors. Panel **a** reports the frequency of positive (*n* = 620) and negative (*n* = 743) terms in the corpus using LIWC, and each dot maps the frequency (in percentage) of each word across authors. In our corpus, there is a higher proportion of positive emotions compared to negative ones (mean ± se; positive terms 2.06% ± .01%; negative: 1.75% ± .01%). The dashed line in the violin plots represents the same analysis conducted using the corpus obtained from Google Books (frequency of positive terms: 2.28%; negative: 1.73%). Panels **b** and **c** show the sex difference for positive and negative words, respectively. Each gray dot represents a male or female author

Additionally, we performed a sentiment analysis on writings and estimated for each author the frequency of all the ~ 14,000 terms included in Warriner’s dataset (Warriner et al., 2013). Normalized estimates of valence and arousal were obtained by summing across words the product between affective ratings and the log-adjusted word ranks, as in:

2 $$\mathrm{author{\prime}}\mathrm{s affective score }= \frac{{\sum }_{w=1}^{n}\left({affective rating}_{w}\right) * log({rank}_{w})}{{\sum }_{w=1}^{n}log({rank}_{w})}$$

where *rank* represents the rank of the frequency of a word *w*, and *affective rating* the sex-matched ratings. For valence, analyses were conducted also employing the ratings of happiness of ~ 10,000 words provided by Dodds and colleagues (Dodds et al., 2015), in which the frequency of the terms included in the dictionary is controlled (Fig. S4). We characterized the author’s affective ratings in time using the sliding temporal windows approach (Fig. 3e, f, S4a). We represented in scatter plots (Fig. 3g) the relationship between the two affective dimensions across authors and used a general linear model to assess the effect of *sex* in their associations (i.e., *arousal* ~ *intercept* + *sex* + sex * valence + *valence*). To further explore the relationship between sex and valence in literature, we analyzed the overall frequency of 620 positive and 743 negative affective terms (i.e., raw occurrences) in our corpus employing the Linguistic Inquiry and Word Count (LIWC, version 2015; Pennebaker et al., 2015). A Wilcoxon rank-sum test assessed whether positive terms are more (or less) frequent than negative terms in the literature (Fig. 4a) and whether they differ between the two sexes (Fig. 4b, c).

## Results

### Revealing Sex Differences in Literature

A set of 576 words shows either a significant main effect of the authors’ *sex* or of the *sex* * *historical period* interaction (*p*_FWC_ < .05, Table S1). We represent significant words in a two-dimensional space summarizing their semantic relatedness (Fig. 2a for the *sex* effect, Fig. S3 for the *sex* * *historical period* effect). Similar results were obtained using word rank instead of word frequency (see SI). For each significant term, we make available Cohen’s *d* of the sex effect in each country (Fig. S6a), the historical trend of each word frequency for male and female authors (Fig. S6b), and semantic shifts between the sexes (see SI and Fig. S6c). An interactive summary of the results is available at https://www.sane-elab.eu/litemo/welcome.php. Regarding content words (i.e., nouns, verbs, and adjectives), we focus our analysis on nouns, as ~ 75% of the significant terms have a form pertaining to this category. Therefore, we classify 85% of the identified nouns according to a fixed set of semantic domains derived from WordNet (Miller, 1995). We show that words about numbers (e.g., *hundred*, *second*, *five*) and weapons (e.g., *shotgun*, *ammunition*, *sword*) are more frequently employed by male authors, with the effect size of sex differences being stable over time in the range from small (Cohen’s *d* ≅ .2) to medium (Cohen’s *d* ≅ .5). Females, instead, write more frequently about clothes (e.g., *silk*, *skirt*, *apron*), food/beverages (e.g., *tea*, *milk*, *sugar*), and plants (e.g., *flowers*, *violets*, *daffodils*). Concerning these semantic domains, the effect size of sex differences also ranges from small to medium, though it has recently decreased. Words about social groups (e.g., *commander*, *captain*, *leader*) and locations (e.g., *perimeter*, *stations*, *east*) are more present in male writings, but the effect size ranges from very small (Cohen’s *d* < .1) to small.

Interestingly, sex differences in affect (e.g., *love*, *hate*, *pity*), time (e.g., *winter*, *holidays*, *birthday*), and body parts (e.g., *hair*, *beard*, *skin*) show a significant trend over time. Words about affect and time were more frequent in female writings of the nineteenth century (medium effect size), whereas in the last two decades, they have approached gender equality (small or very small effect size). In sharp contrast, starting in 1960, body terms have become progressively more frequent in female books as compared to male writings.

### Sex Differences in the Affective Lexicon over Time and Across Cultures

Firstly, we explore differences in the affective properties of the lexicon between male and female authors by focusing on the 576 terms showing significant *sex* or *sex* * *historical period* effects. Of these, 339 words are included in the Warriner database (Warriner et al., 2013) with ratings of valence and arousal from contemporary men and women. For instance, *shotgun* is rated as a negatively laden word, but also, in terms of semantic relatedness, is associated with *harm* and *rampage*, among other negative terms. Results show that words preferentially employed by female authors are more positively valenced (difference in ratings on a 1–9 Likert scale =  − 1.22, CI 95 =  − 1.40–0.82, *p* < .0001; Fig. 3a, c) than those used by males. Instead, terms more frequently employed by male authors are judged as more arousing (sex effect =  + .33, CI 95 =  + .23 + .71, *p* = .0021; Fig. 3b, d). We confirm these results by analyzing words showing a significant main effect of sex and a sex * historical period interaction separately (Fig. S8).

In addition to measuring affective dimensions on a set of selected terms, we obtain affective norms of 14,000 terms (Warriner et al., 2013) and evaluate through sentiment analysis the valence and arousal of each author’s writings over time. We confirm that female writers are more likely to publish positively valenced novels, as compared to their male colleagues (Fig. 3e). Importantly, in recent times, this difference has substantially diminished, and writings of contemporary authors show comparable valence scores between the sexes. In addition, male authors typically write more arousing texts across historical periods (Fig. 3f). We observe a significant modulating effect of sex on the relationship between valence and arousal. In particular, while negatively valenced writings authored by males are typically higher in arousal, the same relationship is less pronounced in females (Fig. 3g; adjusted *R*^2^ = .89, *p*-value < .0001; β ± standard error: β_sex*valence_ 0.395 ± 0.024, *t*_(1361)_ 15.89, *p*-value < .0001). Using the ~ 1,400 terms in LIWC, we confirm the presence of the positivity bias in world literature (Augustine et al., 2011; Fig. 4a), as the average frequency (± standard error) of positive terms across authors is 2.06% (± .01%), whereas the average occurrence of negative words is 1.75% (± .01%, difference between positive and negative term frequency *p* < .0001). Also, books written by females contain more positively laden terms (males: 2.02% ± .02%; females: 2.13% ± .02%, males vs. females =  − .11%, *p* = .0003; Fig. 4b), in line with previous evidence (Newman et al., 2008). The distribution of negative affective terms, instead, does not significantly differ between the sexes (males: 1.73% ± .01%; females: 1.77% ± .02%, males vs. females =  − .04%, *p* = .1816; Fig. 4c).

## Discussion

In the present study, we created a large corpus of literary fiction enriched by authors’ metadata to investigate whether sex differences in the affective lexicon are associated with societal progress and to measure the extent to which culture influences how men and women write about emotion. We show that, overall, sex differences are more prominent in semantic domains that align with stereotypes. Interestingly, although before the twenty-first century and across 116 countries women more than men have written about affect, starting from 2000, this difference has diminished substantially. Also, in the past, women’s narratives were more positively laden and less arousing. While the difference in arousal is ubiquitous and still present nowadays, sex differences in valence vary as a function of culture and have dissolved in recent years. Lastly, the authors from more developed countries typically use less negative and less arousing words, regardless of their sex. Altogether, these findings suggest that societal progress worldwide is associated with men and women writing similarly about emotions and reveal a sizable impact of culture on the affective characteristics of the lexicon.

Literature is an essential cultural institution and an expression of society (Wellek & Warren, 1956), a transposition of the social life of people living in a particular place and era (e.g., *Hard Times* by Charles Dickens, 1854). At the same time, books describe intimate affective experiences (e.g., *The Stream of Life* by Clarice Lispector, 1973), so that regular readers are better at recognizing complex emotions (Schwering et al., 2021). Recently, researchers have come to realize that such a detailed depiction of society and individuals can be used to address questions of psychological relevance (Jackson et al., 2022). As a matter of fact, Scheffer and colleagues (2021) have shown that after the year 1850, the use of emotionally laden words has yielded to fact-based argumentation, paralleling the rapid growth of science and technology. This pattern reversed during the last two decades of the twentieth century, with the surge of social media and the economic crisis of early 2000 fostering the spread of the emotion terms in world literature (Scheffer et al., 2021). Here, using a similar approach, we show that starting in 1960, body terms have become increasingly frequent in female books. The attention to the body and the systematic use of related terms could be attributed to the rise of the second wave of feminism (i.e., from 1960 to 1980), which held as fundamental topics reproductive rights, sexuality, and domestic violence, all themes that posit at their core the body.

We show that, while words about affect were more frequent in books authored by women of the past centuries, the frequency of emotionally laden words in men’s and women’s writings became comparable at the beginning of the twenty-first century. The fact that, in the past and across 116 countries, female authors have written more about emotion reflects the stereotype of women being more emotional and expressive than men (Briton & Hall, 1995; Plant et al., 2004; Robinson & Johnson, 1997; Timmers et al., 2003), or having more sophisticated emotion concepts (Seidlitz & Diener, 1998). However, several of these stereotypes have been questioned, as in the case of gender differences in self-conscious emotions (e.g., *guilt* or *shame*). Indeed, Else-Quest and colleagues (Else-Quest et al., 2012) have shown that the differences between men and women are small for guilt and shame and negligible for embarrassment, authentic and hubristic pride. Also, the modest disparity in the use of emotion terms between men and women characterizing the last two decades may reflect the widespread effort of societies to reduce the gender gap (e.g., the ban of sex discrimination, or the recognition of gender-related crimes). In addition to improving the condition of women (Cruea, 2005), ensuring equal opportunities for the two sexes may help to reduce male stereotypes, such as being discouraged from displaying emotions (Fischer & Manstead, 2000). For instance, cultural norms of hegemonic masculinity explain why crying men are more likely to receive help from a woman rather than from another man (Stadel et al., 2019), whereas women do not show this double standard. In this regard, we speculate that because men and women have become increasingly free to express themselves through literature, the stereotypical aversion of male authors to writing emotionally laden books has diminished over time. At the same time, societal progress may have helped to relieve the pressure on women to make extensive use of emotionally laden terms.

In addition to measuring the extent to which the authors concentrate on emotion, by studying texts, we can also gauge the type of feelings they express in their writings. For instance, Acerbi and colleagues (Acerbi et al., 2013) show how negative emotions have risen during World War II and that positively laden terms in literature have substantially diminished over the last 300 years (Morin & Acerbi, 2017). Moreover, by analyzing headlines of written news media between 2000 and 2019 (Rozado et al., 2022) and song lyrics from the last 60 years (Brand et al., 2019), researchers have confirmed a noteworthy increase in sentiment negativity. Here, we apply sentiment analysis to literary fiction to reveal sex differences in valence and arousal.

Firstly, our results demonstrate that, on average, female writers tend to use positive words more frequently than men, whereas there are no differences between the sexes in the use of negative words. This finding is once again in line with the attribution of stereotypical roles to females. For instance, women refer to positive emotions more often than men in conversations and writings (Feng & Ivanov, 2022; Thelwall et al., 2010), and positive emotions are cross-culturally considered more desirable for females than for males (Diener & Lucas, 2004). Nevertheless, as for the frequency of emotionally laden terms, sex differences in valence have diminished as a function of societal progress worldwide, reaching comparable scores in the last decade. Also, different from what stereotypes prescribe, whether male or female authors write more positively laden books varies across cultures. Therefore, sociocultural factors not only influence the extent to which men and women write about emotion but the affective content of their books as well.

Secondly, we observe that male writings are higher in arousal as compared to female books, a difference that is common to all the explored cultures and stable over time. This evidence is supported by a recent study on gender bias in word embeddings (Caliskan et al., 2022), by males using more frequently than females strong swear words (Güvendir, 2015; Mehl & Pennebaker, 2003), and by their preference for emotions higher in arousal, such as fearlessness (Diener & Lucas, 2004) or pride (Brebner, 2003). Moreover, our result dovetails with the prescriptive stereotype that males should demonstrate assertiveness and competitiveness, while females should be warm, sensitive, and cooperative (Koenig, 2018). Interestingly, Eagly and colleagues (Eagly et al., 2020) have shown that the stereotype of men being more agentic has remained stable in the US culture over the last 70 years.

Thirdly, by exploring the relationship between valence and arousal across the sexes, we reveal a significant interaction effect. Indeed, while sex differences in arousal are larger for negatively-laden writings, this gap becomes smaller for positive books. We hypothesize this can be explained by the negative events that men and women are more likely to experience in their lifetime, but also by social norms controlling what is appropriate to publish. For instance, posttraumatic stress disorder in men is often the consequence of war and violence (Kessler et al., 1995), events that are typically described using very arousing words. According to the Affective Norms for English Words (Bradley & Lang, 1999), *war*, *bomb*, *fight*, *gun*, and *terrorist* are all terms rated 7 points or higher (Likert scale from 1 to 9) on the arousal scale. Instead, traumatic experiences among women are associated with rape and sexual assault (Kessler et al., 1995), events narrated using “strong” words as well (e.g., women’s arousal rating of *rapist* = 7.15; Warriner et al., 2013). However, while countless books have been written by men over centuries about wars and battles, writings tackling sexual assaults by women appeared lately (e.g., *The Color Purple* by Alice Walker, 1982), presumably because of social stigma. Also, concerning negative life events, women are more likely than men to receive a diagnosis of depression already starting from pubertal age (Salk et al., 2017) and this may have an influence on the content of their writings.

One potential limitation of our work is that to characterize male and female writings between the eighteenth and the twenty-first centuries, we use affective ratings obtained from US participants in 2012 (Warriner et al., 2013). Valence and arousal ratings collected in modern times may not be respectful of the affective meaning of words in ancient times. However, we demonstrate no significant semantic shifts between the sexes and find only a marginal proportion of diachronic words among the 576 significant terms.

### Supplementary Information

Below is the link to the electronic supplementary material.

Supplementary file1 (DOCX 177 KB)

Supplementary file2 (DOCX 356 KB)

Supplementary file3 (DOCX 17 KB)

Supplementary file4 (DOCX 16 KB)

Supplementary file5 (DOCX 14 KB)

Supplementary file6 (DOCX 2757 KB)

## Additional Information

### Data and Code Availability

All our data and findings can be freely and publicly consulted online at https://www.sane-elab.eu/litemo/welcome.php. and at https://github.com/giacomohandjaras/LitEmo. We release the matrix of word frequencies per author as well as the word embeddings without violating the “fair use” principle of copyright law. Because the aim of our study was to investigate sex differences in the use of words, the replicability of our work does not depend on the release of books in their entirety.
