# Supplementary material for: How Male and Female Literary Authors Write About Affect Across Cultures and Over Historical Periods
Source: Affect Sci. 2023 Sep 5;4(4):770–80. doi: 10.1007/s42761-023-00219-9 (PMC10751284; doi:10.1007/s42761-023-00219-9)
Supplement: Supplementary file 1 — Supplementary file1 (DOCX 177 KB) [file 42761_2023_219_MOESM1_ESM.docx]

|  | **average** | **average** | **std err** | **std err** |  |  |  |  |  |  |  |  |  |  |  |
| --- | --- | --- | --- | --- | --- | --- | --- | --- | --- | --- | --- | --- | --- | --- | --- |
|  |  | **frequency** |  | **frequency** |  | **p-value sex & sex** | **T-coeff** | **p-value** | **T-coeff sex by** | **p-value sex by** |  |  |  |  |  |
| **Term** | **frequency** |  | **frequency** |  | **F-stats** |  |  |  |  |  |  |  |  |  |  |
|  |  | **in females** |  | **in females** |  | **by publication year** | **sex** | **sex** | **publication year** | **publication year** |  |  |  |  |  |
|  | **in males %** | **%** | **in males %** | **%** |  |  |  |  |  |  |  |  |  |  |  |
|  |  |  |  |  |  |  |  |  |  |  |  |  |  |  |  |
| *the* | *5,674299* | *5,030053* | *0,036226* | *0,043271* | 53,46 | 0,0000 | -5,70 | 0,0000 | 0,83 | 0,3962 |  | **Legend** | | |  |
| *of* | *2,346366* | *2,021385* | *0,021395* | *0,023883* | 33,57 | 0,0000 | -3,81 | 0,0001 | -0,16 | 0,8839 |  |  | > Males, p<0.05 | |  |
|  |  |  |  |  |  |  |  |  |  |  |  |  |  |  |  |
| *a* | *2,295643* | *2,140411* | *0,011755* | *0,014313* | 37,31 | 0,0000 | -3,49 | 0,0004 | -0,76 | 0,4380 |  |  | > Females, p<0.05 | |  |
| *he* | *1,573941* | *1,337942* | *0,020713* | *0,025589* | 30,73 | 0,0000 | -2,91 | 0,0016 | -0,96 | 0,3368 |  |  | Sex by Publication Year, p<0.05 |  |  |
| *his* | *0,993697* | *0,814191* | *0,013044* | *0,017297* | 35,79 | 0,0000 | -4,16 | 0,0000 | 0,09 | 0,9287 |  |  | Sex & Sex by Publication Year, p<0.05 FWEcorr | |  |
| *she* | *0,739400* | *1,282806* | *0,017438* | *0,029446* | 130,35 | 0,0000 | 10,33 | 0,0000 | -3,06 | 0,0020 |  |  |  |  |  |
| *her* | *0,716195* | *1,225375* | *0,015274* | *0,028376* | 131,73 | 0,0000 | 9,47 | 0,0000 | -1,94 | 0,0567 |  |  |  |  |  |
| *t* | *0,556798* | *0,685553* | *0,011387* | *0,015805* | 15,75 | 0,0353 | 3,49 | 0,0003 | -0,94 | 0,3336 |  |  |  |  |  |
| *so* | *0,319069* | *0,354392* | *0,003588* | *0,004546* | 33,61 | 0,0000 | 3,58 | 0,0008 | 0,42 | 0,6831 |  |  |  |  |  |
| *by* | *0,325147* | *0,266149* | *0,003955* | *0,004216* | 32,00 | 0,0000 | -3,05 | 0,0028 | -0,89 | 0,3604 |  |  |  |  |  |
| *when* | *0,297617* | *0,339354* | *0,003054* | *0,004165* | 30,37 | 0,0000 | 3,41 | 0,0004 | 0,39 | 0,6859 |  |  |  |  |  |
| *an* | *0,280803* | *0,235796* | *0,006066* | *0,003466* | 32,39 | 0,0000 | -2,88 | 0,0038 | -1,11 | 0,2702 |  |  |  |  |  |
| *man* | *0,169821* | *0,128942* | *0,002882* | *0,003453* | 43,44 | 0,0000 | -5,04 | 0,0000 | 0,64 | 0,5335 |  |  |  |  |  |
| *how* | *0,150211* | *0,175060* | *0,001792* | *0,002349* | 26,58 | 0,0001 | 2,36 | 0,0205 | 1,27 | 0,2064 |  |  |  |  |  |
| *go* | *0,133671* | *0,152842* | *0,002045* | *0,002721* | 23,10 | 0,0006 | 3,86 | 0,0003 | -0,68 | 0,5031 |  |  |  |  |  |
| *little* | *0,128667* | *0,137274* | *0,002876* | *0,003778* | 16,92 | 0,0165 | 3,79 | 0,0002 | -1,20 | 0,2150 |  |  |  |  |  |
| *two* | *0,135517* | *0,115062* | *0,001500* | *0,001841* | 37,61 | 0,0000 | -3,61 | 0,0001 | -0,64 | 0,5219 |  |  |  |  |  |
| *never* | *0,114641* | *0,139419* | *0,001480* | *0,002145* | 66,67 | 0,0000 | 8,49 | 0,0000 | -3,66 | 0,0002 |  |  |  |  |  |
| *too* | *0,115056* | *0,135305* | *0,001580* | *0,002079* | 31,20 | 0,0000 | 3,58 | 0,0004 | 0,26 | 0,7799 |  |  |  |  |  |
| *come* | *0,113011* | *0,123320* | *0,001519* | *0,002023* | 20,81 | 0,0018 | 4,35 | 0,0000 | -1,51 | 0,1511 |  |  |  |  |  |
| *eyes* | *0,104266* | *0,124229* | *0,001805* | *0,003088* | 15,90 | 0,0319 | 0,34 | 0,7351 | 2,42 | 0,0197 |  |  |  |  |  |
| *thought* | *0,100302* | *0,117913* | *0,002200* | *0,002559* | 24,51 | 0,0003 | 5,40 | 0,0000 | -2,57 | 0,0085 |  |  |  |  |  |
|  |  |  |  |  |  |  |  |  |  |  |  |  |  |  |  |
| *its* | *0,103090* | *0,079357* | *0,002203* | *0,002201* | 19,58 | 0,0034 | -2,07 | 0,0495 | -1,04 | 0,3004 |  |  |  |  |  |
| *look* | *0,082884* | *0,099944* | *0,001217* | *0,002153* | 16,96 | 0,0161 | 3,67 | 0,0001 | -1,04 | 0,2966 |  |  |  |  |  |
| *himself* | *0,086470* | *0,056837* | *0,001717* | *0,001804* | 47,64 | 0,0000 | -3,99 | 0,0000 | -0,81 | 0,4120 |  |  |  |  |  |
| *mother* | *0,070355* | *0,128889* | *0,002683* | *0,005512* | 70,80 | 0,0000 | 6,68 | 0,0000 | -1,10 | 0,2756 |  |  |  |  |  |
| *house* | *0,077285* | *0,097455* | *0,001757* | *0,002682* | 24,25 | 0,0003 | 4,79 | 0,0000 | -1,76 | 0,0714 |  |  |  |  |  |
| *knew* | *0,068827* | *0,081579* | *0,001256* | *0,001709* | 16,05 | 0,0289 | 4,88 | 0,0000 | -2,88 | 0,0031 |  |  |  |  |  |
| *always* | *0,068163* | *0,087706* | *0,001132* | *0,002010* | 65,39 | 0,0000 | 8,67 | 0,0000 | -3,99 | 0,0000 |  |  |  |  |  |
| *another* | *0,072731* | *0,066439* | *0,000722* | *0,000922* | 22,65 | 0,0007 | -4,04 | 0,0000 | 0,94 | 0,3370 |  |  |  |  |  |
| *men* | *0,069199* | *0,054617* | *0,001801* | *0,001888* | 15,42 | 0,0443 | -3,94 | 0,0000 | 1,56 | 0,1256 |  |  |  |  |  |
| *things* | *0,064432* | *0,076582* | *0,001181* | *0,001604* | 22,13 | 0,0009 | 3,54 | 0,0002 | -0,38 | 0,7165 |  |  |  |  |  |
| *woman* | *0,063907* | *0,081529* | *0,002121* | *0,002582* | 29,73 | 0,0000 | 4,44 | 0,0000 | -0,84 | 0,4000 |  |  |  |  |  |
| *three* | *0,064706* | *0,052960* | *0,000965* | *0,001072* | 33,02 | 0,0000 | -4,41 | 0,0000 | 0,56 | 0,5811 |  |  |  |  |  |
| *home* | *0,057219* | *0,081942* | *0,001104* | *0,001974* | 52,73 | 0,0000 | 5,97 | 0,0000 | -1,20 | 0,2230 |  |  |  |  |  |
| *ever* | *0,053974* | *0,059753* | *0,000784* | *0,001167* | 17,46 | 0,0118 | 4,91 | 0,0000 | -2,71 | 0,0075 |  |  |  |  |  |
| *love* | *0,049313* | *0,063952* | *0,001581* | *0,002319* | 37,45 | 0,0000 | 5,54 | 0,0000 | -1,65 | 0,0894 |  |  |  |  |  |
| *oh* | *0,041154* | *0,057352* | *0,001438* | *0,002604* | 21,14 | 0,0015 | 3,35 | 0,0013 | -0,25 | 0,8101 |  |  |  |  |  |
| *end* | *0,048771* | *0,042993* | *0,000627* | *0,000720* | 19,85 | 0,0030 | -1,31 | 0,1931 | -1,82 | 0,0771 |  |  |  |  |  |
|  |  |  |  |  |  |  |  |  |  |  |  |  |  |  |  |
| *girl* | *0,041033* | *0,060850* | *0,001146* | *0,002196* | 38,05 | 0,0000 | 3,33 | 0,0016 | 0,98 | 0,3406 |  |  |  |  |  |
|  |  |  |  |  |  |  |  |  |  |  |  |  |  |  |  |
| *herself* | *0,032699* | *0,058686* | *0,001075* | *0,001977* | 103,49 | 0,0000 | 9,09 | 0,0000 | -2,59 | 0,0132 |  |  |  |  |  |
| *mrs* | *0,026183* | *0,055628* | *0,001953* | *0,005202* | 24,95 | 0,0002 | 6,42 | 0,0000 | -4,20 | 0,0000 |  |  |  |  |  |
| *feel* | *0,040082* | *0,054627* | *0,000896* | *0,001580* | 35,25 | 0,0000 | 3,07 | 0,0019 | 1,08 | 0,2762 |  |  |  |  |  |
| *bed* | *0,040146* | *0,052716* | *0,000860* | *0,001432* | 28,08 | 0,0001 | 1,60 | 0,1073 | 2,13 | 0,0260 |  |  |  |  |  |
| *children* | *0,033865* | *0,058312* | *0,001141* | *0,002856* | 39,34 | 0,0000 | 5,77 | 0,0000 | -1,81 | 0,0661 |  |  |  |  |  |
|  |  |  |  |  |  |  |  |  |  |  |  |  |  |  |  |
| *together* | *0,037596* | *0,043222* | *0,000554* | *0,000770* | 20,07 | 0,0026 | 1,43 | 0,1460 | 1,72 | 0,0891 |  |  |  |  |  |
| *heart* | *0,034437* | *0,037647* | *0,001161* | *0,001209* | 22,90 | 0,0006 | 2,15 | 0,0370 | 1,21 | 0,2278 |  |  |  |  |  |
| *hair* | *0,032774* | *0,052136* | *0,000676* | *0,001353* | 70,89 | 0,0000 | 4,16 | 0,0000 | 1,74 | 0,0790 |  |  |  |  |  |
| *keep* | *0,035218* | *0,041324* | *0,000535* | *0,000696* | 23,46 | 0,0005 | 2,34 | 0,0233 | 1,05 | 0,2940 |  |  |  |  |  |
| *leave* | *0,035241* | *0,039957* | *0,000571* | *0,000815* | 18,05 | 0,0083 | 1,55 | 0,1319 | 1,44 | 0,1560 |  |  |  |  |  |
|  |  |  |  |  |  |  |  |  |  |  |  |  |  |  |  |
| *women* | *0,032212* | *0,046587* | *0,000912* | *0,001972* | 22,90 | 0,0006 | 3,13 | 0,0030 | 0,15 | 0,8763 |  |  |  |  |  |

Page 1

| *family* | *0,031705* | *0,043379* | *0,000955* | *0,001482* | 22,01 | 0,0009 | 4,60 | 0,0000 | -1,73 | 0,0906 |  |  |
| --- | --- | --- | --- | --- | --- | --- | --- | --- | --- | --- | --- | --- |
| *help* | *0,031380* | *0,038808* | *0,000520* | *0,000847* | 19,96 | 0,0028 | 3,24 | 0,0019 | -0,22 | 0,8387 |  |  |
| *child* | *0,029327* | *0,045177* | *0,001236* | *0,001926* | 64,47 | 0,0000 | 6,48 | 0,0000 | -1,18 | 0,2456 |  |  |
| *miss* | *0,022036* | *0,039901* | *0,002009* | *0,003991* | 31,42 | 0,0000 | 6,09 | 0,0000 | -2,88 | 0,0082 |  |  |
| *alone* | *0,030295* | *0,033845* | *0,000531* | *0,000704* | 23,74 | 0,0004 | 2,41 | 0,0224 | 1,00 | 0,3090 |  |  |
| *five* | *0,032950* | *0,027075* | *0,000663* | *0,000728* | 23,70 | 0,0004 | -5,09 | 0,0000 | 2,22 | 0,0312 |  |  |
| *four* | *0,032405* | *0,026909* | *0,000588* | *0,000804* | 23,72 | 0,0004 | -4,10 | 0,0000 | 0,93 | 0,3708 |  |  |
| *sometimes* | *0,027641* | *0,034682* | *0,000680* | *0,000971* | 20,59 | 0,0020 | 3,65 | 0,0006 | -0,65 | 0,5047 |  |  |
| *sir* | *0,024990* | *0,017108* | *0,001880* | *0,002568* | 34,94 | 0,0000 | -3,42 | 0,0002 | -0,68 | 0,4860 |  |  |
| *lady* | *0,017700* | *0,025755* | *0,001060* | *0,002427* | 21,88 | 0,0010 | 4,03 | 0,0000 | -1,00 | 0,3136 |  |  |
| *arms* | *0,025572* | *0,033216* | *0,000508* | *0,000967* | 24,55 | 0,0003 | -0,42 | 0,6831 | 3,74 | 0,0003 |  |  |
| *second* | *0,028535* | *0,024020* | *0,000670* | *0,000587* | 23,13 | 0,0005 | -3,73 | 0,0001 | 0,53 | 0,5987 |  |  |
|  |  |  |  |  |  |  |  |  |  |  |  |  |
| *point* | *0,028249* | *0,022765* | *0,000573* | *0,000670* | 24,73 | 0,0003 | -4,28 | 0,0000 | 1,06 | 0,2902 |  |  |
| *school* | *0,025075* | *0,040623* | *0,001189* | *0,002316* | 16,61 | 0,0201 | 4,00 | 0,0000 | -1,51 | 0,1333 |  |  |
| *city* | *0,028352* | *0,021895* | *0,001039* | *0,001153* | 22,56 | 0,0007 | -2,27 | 0,0268 | -1,06 | 0,2788 |  |  |
| *live* | *0,024083* | *0,028642* | *0,000453* | *0,000703* | 25,70 | 0,0002 | 5,33 | 0,0000 | -2,36 | 0,0188 |  |  |
| *often* | *0,022402* | *0,025516* | *0,000570* | *0,000802* | 21,63 | 0,0011 | 5,75 | 0,0000 | -3,50 | 0,0007 |  |  |
| *husband* | *0,019836* | *0,029026* | *0,000766* | *0,001308* | 31,43 | 0,0000 | 4,31 | 0,0000 | -0,57 | 0,5677 |  |  |
| *dear* | *0,018920* | *0,020668* | *0,000991* | *0,001260* | 16,00 | 0,0299 | 4,22 | 0,0000 | -1,88 | 0,0537 |  |  |
| *care* | *0,020102* | *0,025396* | *0,000340* | *0,000571* | 34,84 | 0,0000 | 4,49 | 0,0000 | -0,54 | 0,5987 |  |  |
| *wall* | *0,025169* | *0,021005* | *0,000731* | *0,000690* | 16,78 | 0,0181 | -3,24 | 0,0007 | 0,52 | 0,5927 |  |  |
| *stay* | *0,020255* | *0,027324* | *0,000397* | *0,000573* | 50,66 | 0,0000 | 6,73 | 0,0000 | -2,29 | 0,0250 |  |  |
| *ten* | *0,023401* | *0,018807* | *0,000445* | *0,000476* | 23,43 | 0,0005 | -3,74 | 0,0000 | 0,52 | 0,6141 |  |  |
| *twenty* | *0,023432* | *0,018456* | *0,000499* | *0,000514* | 22,26 | 0,0008 | -3,75 | 0,0001 | 0,62 | 0,5543 |  |  |
| *happy* | *0,019921* | *0,024526* | *0,000469* | *0,000711* | 27,39 | 0,0001 | 6,17 | 0,0000 | -3,43 | 0,0007 |  |  |
| *question* | *0,021565* | *0,017287* | *0,000460* | *0,000442* | 16,75 | 0,0184 | -0,84 | 0,4006 | -2,03 | 0,0435 |  |  |
| *sister* | *0,015804* | *0,025156* | *0,000745* | *0,001157* | 32,69 | 0,0000 | 4,46 | 0,0000 | -0,65 | 0,5135 |  |  |
|  |  |  |  |  |  |  |  |  |  |  |  |  |
| *hundred* | *0,022953* | *0,013230* | *0,000648* | *0,000476* | 63,03 | 0,0000 | -6,99 | 0,0000 | 1,88 | 0,0579 |  |  |
| *beautiful* | *0,019131* | *0,023860* | *0,000614* | *0,000759* | 31,44 | 0,0000 | 4,65 | 0,0000 | -0,97 | 0,3390 |  |  |
| *order* | *0,021423* | *0,014964* | *0,000546* | *0,000549* | 15,97 | 0,0305 | -3,75 | 0,0003 | 1,24 | 0,2186 |  |  |
| *girls* | *0,016482* | *0,031457* | *0,000730* | *0,001889* | 40,09 | 0,0000 | 5,27 | 0,0000 | -1,12 | 0,2664 |  |  |
| *daughter* | *0,016844* | *0,023826* | *0,000672* | *0,000982* | 28,03 | 0,0001 | 2,65 | 0,0092 | 1,05 | 0,3082 |  |  |
| *cried* | *0,016217* | *0,018399* | *0,000971* | *0,001166* | 37,03 | 0,0000 | 3,00 | 0,0044 | 1,27 | 0,2142 |  |  |
| *sit* | *0,018400* | *0,024377* | *0,000446* | *0,000809* | 17,81 | 0,0095 | 3,28 | 0,0009 | -0,47 | 0,6435 |  |  |
| *afraid* | *0,017532* | *0,020236* | *0,000448* | *0,000662* | 18,02 | 0,0084 | 2,94 | 0,0060 | -0,05 | 0,9685 |  |  |
| *kitchen* | *0,016866* | *0,027150* | *0,000621* | *0,001153* | 26,94 | 0,0001 | 4,08 | 0,0000 | -0,63 | 0,5225 |  |  |
| *lived* | *0,018026* | *0,021131* | *0,000410* | *0,000651* | 17,01 | 0,0156 | 5,29 | 0,0000 | -3,45 | 0,0009 |  |  |
| *itself* | *0,019133* | *0,014587* | *0,000447* | *0,000570* | 18,61 | 0,0059 | -3,47 | 0,0002 | 0,62 | 0,5429 |  |  |
| *line* | *0,019903* | *0,016882* | *0,000526* | *0,000499* | 25,78 | 0,0002 | -4,83 | 0,0000 | 1,66 | 0,0927 |  |  |
| *clothes* | *0,016622* | *0,022466* | *0,000407* | *0,000635* | 25,65 | 0,0002 | 3,12 | 0,0033 | 0,38 | 0,7015 |  |  |
| *loved* | *0,013582* | *0,019966* | *0,000398* | *0,000662* | 48,04 | 0,0000 | 7,18 | 0,0000 | -3,08 | 0,0021 |  |  |
| *baby* | *0,011825* | *0,028802* | *0,000669* | *0,001935* | 61,80 | 0,0000 | 3,94 | 0,0000 | 1,56 | 0,1286 |  |  |
| *tears* | *0,014286* | *0,019459* | *0,000390* | *0,000570* | 60,29 | 0,0000 | 5,49 | 0,0000 | -0,22 | 0,8349 |  |  |
| *eat* | *0,015655* | *0,020834* | *0,000485* | *0,000721* | 20,13 | 0,0025 | 2,46 | 0,0185 | 0,66 | 0,5155 |  |  |
| *general* | *0,016520* | *0,010677* | *0,001319* | *0,002538* | 30,85 | 0,0000 | -4,20 | 0,0000 | 0,48 | 0,6389 |  |  |
| *liked* | *0,013459* | *0,018657* | *0,000389* | *0,000657* | 30,22 | 0,0000 | 6,64 | 0,0000 | -3,86 | 0,0001 |  |  |
| *married* | *0,012504* | *0,019406* | *0,000379* | *0,000710* | 43,22 | 0,0000 | 6,59 | 0,0000 | -2,61 | 0,0106 |  |  |
| *number* | *0,017348* | *0,013574* | *0,000488* | *0,000487* | 21,75 | 0,0011 | -3,79 | 0,0001 | 0,71 | 0,4762 |  |  |
| *thousand* | *0,016350* | *0,009020* | *0,000517* | *0,000426* | 47,88 | 0,0000 | -6,40 | 0,0000 | 2,03 | 0,0455 |  |  |
| *skin* | *0,013486* | *0,022194* | *0,000434* | *0,001123* | 22,31 | 0,0008 | -1,28 | 0,1680 | 4,28 | 0,0000 |  |  |
| *captain* | *0,015511* | *0,006394* | *0,001476* | *0,001052* | 30,79 | 0,0000 | -3,04 | 0,0037 | -0,83 | 0,4104 |  |  |
|  |  |  |  |  |  |  |  |  |  |  |  |  |
| *single* | *0,015337* | *0,012518* | *0,000339* | *0,000402* | 15,34 | 0,0469 | -3,91 | 0,0002 | 1,54 | 0,1431 |  |  |
| *parents* | *0,013456* | *0,020599* | *0,000650* | *0,001121* | 15,45 | 0,0434 | 2,76 | 0,0029 | -0,09 | 0,9213 |  |  |
| *led* | *0,013711* | *0,010975* | *0,000284* | *0,000365* | 21,34 | 0,0013 | 0,67 | 0,4826 | -3,72 | 0,0001 |  |  |
| *garden* | *0,012661* | *0,016008* | *0,000509* | *0,000780* | 15,54 | 0,0407 | 4,40 | 0,0000 | -2,20 | 0,0329 |  |  |
|  |  |  |  |  |  |  |  |  |  |  |  |  |

Page 2

| *building* | *0,014914* | *0,012353* | *0,000504* | *0,000546* | 20,16 | 0,0025 | -2,83 | 0,0033 | -0,27 | 0,7767 |  |  |
| --- | --- | --- | --- | --- | --- | --- | --- | --- | --- | --- | --- | --- |
| *tea* | *0,010975* | *0,019478* | *0,000519* | *0,001060* | 30,61 | 0,0000 | 6,33 | 0,0000 | -3,33 | 0,0015 |  |  |
| *laugh* | *0,012314* | *0,015172* | *0,000360* | *0,000504* | 16,81 | 0,0177 | 2,11 | 0,0420 | 0,76 | 0,4594 |  |  |
| *distance* | *0,014207* | *0,010852* | *0,000336* | *0,000328* | 17,42 | 0,0121 | -1,97 | 0,0576 | -0,95 | 0,3450 |  |  |
|  |  |  |  |  |  |  |  |  |  |  |  |  |
| *dinner* | *0,010708* | *0,014993* | *0,000364* | *0,000589* | 25,38 | 0,0002 | 4,56 | 0,0000 | -1,36 | 0,1745 |  |  |
|  |  |  |  |  |  |  |  |  |  |  |  |  |
| *summer* | *0,012319* | *0,015421* | *0,000488* | *0,000680* | 21,03 | 0,0016 | 5,13 | 0,0000 | -2,58 | 0,0096 |  |  |
| *dress* | *0,010249* | *0,018069* | *0,000379* | *0,000770* | 69,42 | 0,0000 | 4,81 | 0,0000 | 0,97 | 0,3246 |  |  |
| *thirty* | *0,013336* | *0,010112* | *0,000340* | *0,000381* | 28,40 | 0,0001 | -3,72 | 0,0002 | 0,10 | 0,9351 |  |  |
| *soft* | *0,010720* | *0,015284* | *0,000297* | *0,000554* | 27,36 | 0,0001 | 2,17 | 0,0402 | 1,51 | 0,1476 |  |  |
| *entered* | *0,012653* | *0,008627* | *0,000413* | *0,000406* | 17,77 | 0,0098 | -2,31 | 0,0212 | -0,63 | 0,5477 |  |  |
| *aunt* | *0,008929* | *0,016039* | *0,000870* | *0,001425* | 37,84 | 0,0000 | 6,52 | 0,0000 | -2,94 | 0,0054 |  |  |
| *fellow* | *0,012223* | *0,006274* | *0,000592* | *0,000395* | 33,41 | 0,0000 | -3,70 | 0,0001 | -0,27 | 0,7657 |  |  |
| *sweet* | *0,010063* | *0,014269* | *0,000394* | *0,000965* | 34,12 | 0,0000 | 3,86 | 0,0001 | 0,15 | 0,9073 |  |  |
| *shot* | *0,012737* | *0,009127* | *0,000414* | *0,000418* | 27,11 | 0,0001 | -3,90 | 0,0001 | 0,40 | 0,6959 |  |  |
| *third* | *0,012213* | *0,009594* | *0,000313* | *0,000331* | 25,00 | 0,0002 | -4,39 | 0,0000 | 1,17 | 0,2398 |  |  |
| *tired* | *0,010970* | *0,014288* | *0,000269* | *0,000412* | 29,70 | 0,0000 | 4,86 | 0,0000 | -1,37 | 0,1954 |  |  |
| *cry* | *0,010208* | *0,014076* | *0,000279* | *0,000453* | 45,27 | 0,0000 | 2,36 | 0,0256 | 2,38 | 0,0178 |  |  |
| *hurt* | *0,009441* | *0,013983* | *0,000280* | *0,000488* | 31,58 | 0,0000 | 3,90 | 0,0000 | -0,08 | 0,9177 |  |  |
| *laughing* | *0,010891* | *0,012735* | *0,000445* | *0,000413* | 15,34 | 0,0467 | 3,35 | 0,0013 | -0,80 | 0,4310 |  |  |
| *glad* | *0,009261* | *0,012013* | *0,000333* | *0,000540* | 17,03 | 0,0154 | 3,36 | 0,0011 | -0,64 | 0,5231 |  |  |
| *form* | *0,011761* | *0,008508* | *0,000323* | *0,000302* | 17,58 | 0,0109 | -4,67 | 0,0000 | 2,33 | 0,0234 |  |  |
| *below* | *0,011771* | *0,008060* | *0,000367* | *0,000302* | 22,91 | 0,0006 | -3,62 | 0,0002 | 0,41 | 0,6759 |  |  |
| *pale* | *0,009429* | *0,010977* | *0,000288* | *0,000392* | 21,20 | 0,0014 | 3,19 | 0,0018 | -0,05 | 0,9635 |  |  |
| *view* | *0,011150* | *0,008258* | *0,000293* | *0,000334* | 23,01 | 0,0006 | -2,34 | 0,0247 | -1,02 | 0,3220 |  |  |
| *sick* | *0,009705* | *0,013047* | *0,000291* | *0,000481* | 22,28 | 0,0008 | 1,95 | 0,0587 | 1,38 | 0,1746 |  |  |
|  |  |  |  |  |  |  |  |  |  |  |  |  |
| *bear* | *0,009267* | *0,010859* | *0,000470* | *0,000551* | 22,91 | 0,0006 | 4,29 | 0,0000 | -1,23 | 0,2114 |  |  |
|  |  |  |  |  |  |  |  |  |  |  |  |  |
| *smoke* | *0,012121* | *0,008936* | *0,000366* | *0,000346* | 26,42 | 0,0001 | -4,47 | 0,0000 | 1,15 | 0,2596 |  |  |
| *position* | *0,010658* | *0,007640* | *0,000316* | *0,000331* | 16,99 | 0,0158 | -3,77 | 0,0010 | 1,15 | 0,2766 |  |  |
| *direction* | *0,010597* | *0,007663* | *0,000276* | *0,000257* | 24,72 | 0,0003 | -4,04 | 0,0000 | 0,76 | 0,4402 |  |  |
| *pointed* | *0,009878* | *0,008211* | *0,000258* | *0,000323* | 18,35 | 0,0069 | -2,49 | 0,0119 | -0,49 | 0,6311 |  |  |
| *shoes* | *0,009336* | *0,012718* | *0,000341* | *0,000445* | 17,96 | 0,0087 | 2,68 | 0,0057 | 0,24 | 0,8145 |  |  |
| *marriage* | *0,007132* | *0,011334* | *0,000322* | *0,000696* | 24,63 | 0,0003 | 5,23 | 0,0000 | -2,33 | 0,0260 |  |  |
| *north* | *0,009599* | *0,008009* | *0,000480* | *0,001024* | 16,54 | 0,0211 | -0,83 | 0,4358 | -2,02 | 0,0451 |  |  |
| *bar* | *0,010688* | *0,007921* | *0,000509* | *0,000657* | 20,03 | 0,0027 | -2,33 | 0,0126 | -0,80 | 0,4020 |  |  |
|  |  |  |  |  |  |  |  |  |  |  |  |  |
| *flowers* | *0,008266* | *0,012133* | *0,000357* | *0,000650* | 45,17 | 0,0000 | 6,24 | 0,0000 | -2,00 | 0,0532 |  |  |
| *army* | *0,009340* | *0,007671* | *0,000505* | *0,001014* | 15,65 | 0,0379 | -1,61 | 0,1125 | -1,18 | 0,2546 |  |  |
|  |  |  |  |  |  |  |  |  |  |  |  |  |
| *shirt* | *0,009425* | *0,009353* | *0,000284* | *0,000421* | 16,79 | 0,0180 | -4,28 | 0,0000 | 1,86 | 0,0622 |  |  |
|  |  |  |  |  |  |  |  |  |  |  |  |  |
| *winter* | *0,008764* | *0,010014* | *0,000389* | *0,000444* | 17,29 | 0,0131 | 5,19 | 0,0000 | -3,21 | 0,0022 |  |  |
| *grown* | *0,008272* | *0,010499* | *0,000256* | *0,000315* | 24,95 | 0,0002 | 4,10 | 0,0000 | -0,82 | 0,4092 |  |  |
| *government* | *0,008721* | *0,007608* | *0,000529* | *0,000855* | 18,84 | 0,0052 | -3,94 | 0,0001 | 1,17 | 0,2530 |  |  |
| *fifty* | *0,009463* | *0,006177* | *0,000261* | *0,000244* | 44,42 | 0,0000 | -5,22 | 0,0000 | 0,79 | 0,4252 |  |  |
| *crying* | *0,007582* | *0,012190* | *0,000241* | *0,000437* | 42,08 | 0,0000 | 3,88 | 0,0000 | 0,61 | 0,5445 |  |  |
| *south* | *0,008740* | *0,007066* | *0,000429* | *0,000678* | 25,87 | 0,0002 | -1,55 | 0,1240 | -2,03 | 0,0504 |  |  |
|  |  |  |  |  |  |  |  |  |  |  |  |  |
| *west* | *0,008305* | *0,005799* | *0,000421* | *0,000355* | 25,01 | 0,0002 | -2,05 | 0,0449 | -1,47 | 0,1360 |  |  |
|  |  |  |  |  |  |  |  |  |  |  |  |  |
| *marry* | *0,005548* | *0,009203* | *0,000267* | *0,000522* | 43,15 | 0,0000 | 6,09 | 0,0000 | -1,94 | 0,0574 |  |  |
| *forty* | *0,008715* | *0,006378* | *0,000248* | *0,000240* | 23,02 | 0,0006 | -3,92 | 0,0002 | 0,76 | 0,4644 |  |  |
| *chief* | *0,007761* | *0,003810* | *0,000588* | *0,000339* | 23,62 | 0,0004 | -3,36 | 0,0005 | 0,05 | 0,9495 |  |  |
| *grandmother* | *0,005563* | *0,015055* | *0,000672* | *0,002777* | 42,11 | 0,0000 | 5,79 | 0,0000 | -1,63 | 0,0944 |  |  |
| *ladies* | *0,005768* | *0,007564* | *0,000319* | *0,000472* | 15,84 | 0,0333 | 3,28 | 0,0016 | -0,66 | 0,4884 |  |  |
| *guard* | *0,008233* | *0,005215* | *0,000468* | *0,000337* | 17,70 | 0,0102 | -4,18 | 0,0000 | 1,62 | 0,1025 |  |  |
| *officer* | *0,009051* | *0,004118* | *0,000527* | *0,000456* | 46,57 | 0,0000 | -4,52 | 0,0000 | -0,15 | 0,8989 |  |  |
| *cousin* | *0,004926* | *0,008860* | *0,000361* | *0,000913* | 16,53 | 0,0211 | 4,76 | 0,0000 | -2,61 | 0,0114 |  |  |
| *spend* | *0,007423* | *0,008521* | *0,000246* | *0,000270* | 15,44 | 0,0436 | 5,02 | 0,0000 | -3,25 | 0,0013 |  |  |
| *colonel* | *0,008277* | *0,002950* | *0,001457* | *0,000553* | 17,77 | 0,0098 | 1,02 | 0,3304 | -3,73 | 0,0000 |  |  |
| *hate* | *0,006076* | *0,008734* | *0,000204* | *0,000376* | 20,89 | 0,0017 | 5,40 | 0,0000 | -3,02 | 0,0037 |  |  |
|  |  |  |  |  |  |  |  |  |  |  |  |  |

Page 3

| *stomach* | *0,006790* | *0,009802* | *0,000234* | *0,000487* | 20,92 | 0,0017 | -4,30 | 0,0000 | 6,10 | 0,0000 |  |  |
| --- | --- | --- | --- | --- | --- | --- | --- | --- | --- | --- | --- | --- |
| *gun* | *0,007827* | *0,004893* | *0,000444* | *0,000487* | 29,47 | 0,0000 | -2,75 | 0,0027 | -1,05 | 0,2978 |  |  |
| *cheeks* | *0,006044* | *0,008498* | *0,000191* | *0,000346* | 16,76 | 0,0183 | 1,88 | 0,0639 | 0,99 | 0,3442 |  |  |
|  |  |  |  |  |  |  |  |  |  |  |  |  |
| *east* | *0,007034* | *0,005152* | *0,000279* | *0,000325* | 26,73 | 0,0001 | -2,64 | 0,0092 | -0,98 | 0,3304 |  |  |
|  |  |  |  |  |  |  |  |  |  |  |  |  |
| *driver* | *0,008212* | *0,006488* | *0,000370* | *0,000401* | 18,50 | 0,0063 | -1,93 | 0,0477 | -1,10 | 0,2428 |  |  |
| *complete* | *0,007180* | *0,005357* | *0,000176* | *0,000230* | 22,35 | 0,0008 | -3,17 | 0,0026 | -0,07 | 0,9381 |  |  |
| *pink* | *0,005691* | *0,010992* | *0,000273* | *0,000470* | 49,55 | 0,0000 | 4,16 | 0,0001 | 0,72 | 0,4756 |  |  |
| *lovely* | *0,005829* | *0,008561* | *0,000269* | *0,000481* | 20,16 | 0,0025 | 4,92 | 0,0000 | -2,38 | 0,0234 |  |  |
| *main* | *0,007545* | *0,005669* | *0,000227* | *0,000298* | 26,14 | 0,0001 | -4,45 | 0,0000 | 1,14 | 0,2606 |  |  |
| *final* | *0,007316* | *0,005566* | *0,000217* | *0,000234* | 29,00 | 0,0000 | -0,93 | 0,3496 | -2,83 | 0,0048 |  |  |
| *hers* | *0,004978* | *0,008966* | *0,000165* | *0,000321* | 77,59 | 0,0000 | 7,13 | 0,0000 | -1,32 | 0,1905 |  |  |
|  |  |  |  |  |  |  |  |  |  |  |  |  |
| *distant* | *0,007184* | *0,005117* | *0,000231* | *0,000228* | 15,74 | 0,0356 | -1,10 | 0,2812 | -1,69 | 0,0998 |  |  |
|  |  |  |  |  |  |  |  |  |  |  |  |  |
| *wear* | *0,005457* | *0,009167* | *0,000152* | *0,000296* | 59,08 | 0,0000 | 5,95 | 0,0000 | -0,83 | 0,4168 |  |  |
|  |  |  |  |  |  |  |  |  |  |  |  |  |
| *lower* | *0,006793* | *0,005127* | *0,000170* | *0,000167* | 19,40 | 0,0038 | -4,41 | 0,0000 | 1,74 | 0,0782 |  |  |
| *battle* | *0,006084* | *0,004020* | *0,000300* | *0,000622* | 18,59 | 0,0060 | -3,03 | 0,0032 | 0,10 | 0,9061 |  |  |
| *milk* | *0,005863* | *0,008750* | *0,000275* | *0,000437* | 22,01 | 0,0009 | 2,52 | 0,0145 | 0,75 | 0,4574 |  |  |
| *wrapped* | *0,005645* | *0,007489* | *0,000162* | *0,000270* | 15,79 | 0,0346 | -0,57 | 0,5527 | 3,19 | 0,0007 |  |  |
| *wedding* | *0,004843* | *0,008249* | *0,000297* | *0,000487* | 34,81 | 0,0000 | 6,13 | 0,0000 | -2,64 | 0,0060 |  |  |
|  |  |  |  |  |  |  |  |  |  |  |  |  |
| *area* | *0,006688* | *0,005287* | *0,000299* | *0,000408* | 24,86 | 0,0002 | -0,60 | 0,5035 | -2,87 | 0,0024 |  |  |
| *action* | *0,006215* | *0,003928* | *0,000241* | *0,000189* | 32,06 | 0,0000 | -3,66 | 0,0001 | -0,23 | 0,8173 |  |  |
|  |  |  |  |  |  |  |  |  |  |  |  |  |
| *pity* | *0,005019* | *0,005619* | *0,000187* | *0,000258* | 24,37 | 0,0003 | 6,18 | 0,0000 | -3,84 | 0,0001 |  |  |
| *report* | *0,006293* | *0,003982* | *0,000318* | *0,000216* | 18,78 | 0,0054 | -4,20 | 0,0000 | 1,52 | 0,1270 |  |  |
| *taught* | *0,005294* | *0,006720* | *0,000172* | *0,000254* | 15,88 | 0,0325 | 5,05 | 0,0000 | -3,21 | 0,0017 |  |  |
| *beer* | *0,006933* | *0,004917* | *0,000359* | *0,000367* | 19,75 | 0,0031 | -1,84 | 0,0565 | -1,29 | 0,1796 |  |  |
|  |  |  |  |  |  |  |  |  |  |  |  |  |
| *spite* | *0,005020* | *0,005295* | *0,000216* | *0,000288* | 15,40 | 0,0450 | 4,41 | 0,0000 | -2,24 | 0,0184 |  |  |
|  |  |  |  |  |  |  |  |  |  |  |  |  |
| *nurse* | *0,004653* | *0,008282* | *0,000264* | *0,000782* | 18,11 | 0,0080 | 5,11 | 0,0000 | -2,94 | 0,0018 |  |  |
| *fucking* | *0,007102* | *0,005526* | *0,000989* | *0,001025* | 20,40 | 0,0022 | -0,77 | 0,2384 | -2,39 | 0,0064 |  |  |
| *dozen* | *0,005974* | *0,003773* | *0,000223* | *0,000189* | 25,85 | 0,0002 | -2,81 | 0,0042 | -0,73 | 0,4854 |  |  |
|  |  |  |  |  |  |  |  |  |  |  |  |  |
| *metal* | *0,006418* | *0,004997* | *0,000310* | *0,000272* | 17,89 | 0,0091 | -3,00 | 0,0001 | 0,13 | 0,8923 |  |  |
| *hated* | *0,004631* | *0,006548* | *0,000181* | *0,000264* | 18,93 | 0,0049 | 4,70 | 0,0000 | -2,20 | 0,0294 |  |  |
| *local* | *0,006100* | *0,005141* | *0,000247* | *0,000340* | 17,37 | 0,0125 | -1,97 | 0,0387 | -0,95 | 0,3306 |  |  |
| *military* | *0,006118* | *0,003508* | *0,000328* | *0,000429* | 32,89 | 0,0000 | -2,40 | 0,0128 | -1,64 | 0,0948 |  |  |
| *comfort* | *0,004337* | *0,005972* | *0,000173* | *0,000251* | 21,68 | 0,0011 | 5,17 | 0,0000 | -2,57 | 0,0142 |  |  |
| *level* | *0,005948* | *0,004283* | *0,000206* | *0,000176* | 22,40 | 0,0008 | -2,43 | 0,0155 | -0,88 | 0,3792 |  |  |
| *christmas* | *0,004342* | *0,007920* | *0,000242* | *0,000558* | 22,39 | 0,0008 | 5,76 | 0,0000 | -3,41 | 0,0010 |  |  |
| *fuck* | *0,006413* | *0,005064* | *0,000576* | *0,000701* | 15,25 | 0,0498 | -1,19 | 0,0756 | -1,56 | 0,0755 |  |  |
|  |  |  |  |  |  |  |  |  |  |  |  |  |
| *seconds* | *0,005735* | *0,004385* | *0,000248* | *0,000252* | 18,24 | 0,0074 | -3,53 | 0,0000 | 0,72 | 0,4526 |  |  |
|  |  |  |  |  |  |  |  |  |  |  |  |  |
| *leading* | *0,005562* | *0,004087* | *0,000153* | *0,000149* | 16,69 | 0,0191 | -2,27 | 0,0282 | -0,58 | 0,5921 |  |  |
| *sisters* | *0,004203* | *0,007183* | *0,000324* | *0,000485* | 21,10 | 0,0015 | 4,24 | 0,0000 | -1,34 | 0,1716 |  |  |
| *system* | *0,005909* | *0,003622* | *0,000334* | *0,000203* | 19,88 | 0,0029 | -4,21 | 0,0000 | 1,43 | 0,1437 |  |  |
| *forgot* | *0,004737* | *0,005793* | *0,000132* | *0,000179* | 28,55 | 0,0001 | 4,65 | 0,0000 | -1,20 | 0,2670 |  |  |
| *headed* | *0,005563* | *0,004561* | *0,000208* | *0,000241* | 17,03 | 0,0154 | -1,31 | 0,1708 | -1,59 | 0,1070 |  |  |
|  |  |  |  |  |  |  |  |  |  |  |  |  |
| *gentlemen* | *0,004344* | *0,002329* | *0,000246* | *0,000230* | 15,46 | 0,0429 | -3,07 | 0,0034 | 0,45 | 0,6331 |  |  |
|  |  |  |  |  |  |  |  |  |  |  |  |  |
| *officers* | *0,005844* | *0,002412* | *0,000449* | *0,000287* | 28,98 | 0,0000 | -3,92 | 0,0001 | 0,28 | 0,7659 |  |  |
| *quarter* | *0,004977* | *0,003497* | *0,000240* | *0,000346* | 17,98 | 0,0086 | -4,36 | 0,0000 | 1,83 | 0,0789 |  |  |
| *silk* | *0,004279* | *0,005534* | *0,000308* | *0,000302* | 15,63 | 0,0385 | 2,86 | 0,0047 | -0,19 | 0,8641 |  |  |
| *evidence* | *0,004681* | *0,003444* | *0,000213* | *0,000203* | 15,25 | 0,0498 | -3,08 | 0,0030 | 0,49 | 0,6267 |  |  |
| *wanting* | *0,003930* | *0,005741* | *0,000137* | *0,000238* | 20,18 | 0,0025 | 2,05 | 0,0383 | 1,11 | 0,2626 |  |  |
| *vast* | *0,005093* | *0,003038* | *0,000234* | *0,000183* | 19,56 | 0,0035 | -3,17 | 0,0031 | 0,17 | 0,8735 |  |  |
| *speed* | *0,005175* | *0,003731* | *0,000221* | *0,000182* | 19,87 | 0,0029 | -3,57 | 0,0003 | 0,63 | 0,5371 |  |  |
| *previous* | *0,004975* | *0,003416* | *0,000160* | *0,000176* | 26,35 | 0,0001 | -2,05 | 0,0446 | -1,56 | 0,1208 |  |  |
| *down-stairs* | *0,003929* | *0,005735* | *0,000180* | *0,000313* | 15,53 | 0,0411 | 4,23 | 0,0000 | -1,95 | 0,0582 |  |  |
| *choose* | *0,003777* | *0,005159* | *0,000117* | *0,000206* | 20,34 | 0,0023 | 4,70 | 0,0000 | -2,03 | 0,0472 |  |  |
| *sugar* | *0,003965* | *0,005638* | *0,000662* | *0,000431* | 19,38 | 0,0038 | 2,25 | 0,0233 | 0,83 | 0,4072 |  |  |
| *century* | *0,004936* | *0,003217* | *0,000258* | *0,000206* | 15,89 | 0,0323 | 0,24 | 0,8167 | -2,93 | 0,0034 |  |  |
|  |  |  |  |  |  |  |  |  |  |  |  |  |

Page 4

| *wash* | *0,004286* | *0,005996* | *0,000157* | *0,000240* | 20,09 | 0,0026 | 2,15 | 0,0323 | 0,99 | 0,3044 |  |  |
| --- | --- | --- | --- | --- | --- | --- | --- | --- | --- | --- | --- | --- |
| *upper* | *0,004571* | *0,003438* | *0,000149* | *0,000160* | 16,55 | 0,0210 | -4,68 | 0,0000 | 2,49 | 0,0135 |  |  |
| *track* | *0,004701* | *0,004060* | *0,000224* | *0,000443* | 22,13 | 0,0009 | -4,45 | 0,0000 | 1,51 | 0,1194 |  |  |
| *leader* | *0,004020* | *0,003033* | *0,000446* | *0,000414* | 18,02 | 0,0084 | -1,76 | 0,0767 | -1,23 | 0,2206 |  |  |
|  |  |  |  |  |  |  |  |  |  |  |  |  |
| *yards* | *0,005020* | *0,002086* | *0,000247* | *0,000130* | 51,53 | 0,0000 | -4,20 | 0,0000 | -0,78 | 0,4352 |  |  |
|  |  |  |  |  |  |  |  |  |  |  |  |  |
| *teach* | *0,003639* | *0,005119* | *0,000121* | *0,000208* | 18,88 | 0,0051 | 4,92 | 0,0000 | -2,54 | 0,0121 |  |  |
| *guards* | *0,004643* | *0,002706* | *0,000412* | *0,000351* | 20,46 | 0,0021 | -3,89 | 0,0001 | 0,96 | 0,3568 |  |  |
| *cell* | *0,004924* | *0,002884* | *0,000450* | *0,000409* | 19,35 | 0,0039 | -2,60 | 0,0108 | -0,45 | 0,6591 |  |  |
| *shoot* | *0,004412* | *0,003193* | *0,000205* | *0,000233* | 15,67 | 0,0373 | -3,07 | 0,0024 | 0,43 | 0,6741 |  |  |
| *national* | *0,004393* | *0,003181* | *0,000239* | *0,000319* | 26,58 | 0,0001 | -2,92 | 0,0021 | -0,67 | 0,4880 |  |  |
| *figures* | *0,004424* | *0,002697* | *0,000171* | *0,000138* | 23,95 | 0,0004 | -1,96 | 0,0587 | -1,48 | 0,1370 |  |  |
|  |  |  |  |  |  |  |  |  |  |  |  |  |
| *record* | *0,004491* | *0,003380* | *0,000227* | *0,000246* | 18,67 | 0,0057 | -2,53 | 0,0083 | -0,47 | 0,6567 |  |  |
|  |  |  |  |  |  |  |  |  |  |  |  |  |
| *lieutenant* | *0,005649* | *0,001401* | *0,000894* | *0,000375* | 23,70 | 0,0004 | -2,53 | 0,0178 | -0,88 | 0,3724 |  |  |
| *staff* | *0,004293* | *0,003472* | *0,000317* | *0,000338* | 16,03 | 0,0293 | -3,44 | 0,0007 | 0,84 | 0,3912 |  |  |
| *grateful* | *0,003251* | *0,004576* | *0,000108* | *0,000191* | 18,50 | 0,0063 | 2,56 | 0,0129 | 0,42 | 0,6989 |  |  |
| *steel* | *0,004230* | *0,002621* | *0,000216* | *0,000160* | 18,04 | 0,0083 | -2,78 | 0,0061 | -0,13 | 0,8833 |  |  |
| *latter* | *0,004123* | *0,002012* | *0,000238* | *0,000220* | 17,18 | 0,0140 | -3,68 | 0,0006 | 1,02 | 0,2978 |  |  |
| *official* | *0,004096* | *0,003121* | *0,000180* | *0,000273* | 20,31 | 0,0023 | -4,06 | 0,0001 | 1,19 | 0,2366 |  |  |
| *produced* | *0,004041* | *0,002359* | *0,000151* | *0,000128* | 29,16 | 0,0000 | -1,61 | 0,1173 | -2,19 | 0,0288 |  |  |
| *fourth* | *0,004279* | *0,002983* | *0,000180* | *0,000146* | 21,96 | 0,0010 | -3,37 | 0,0009 | 0,19 | 0,8523 |  |  |
|  |  |  |  |  |  |  |  |  |  |  |  |  |
| *strike* | *0,003941* | *0,002655* | *0,000139* | *0,000116* | 16,42 | 0,0228 | -3,78 | 0,0000 | 1,23 | 0,2086 |  |  |
| *tender* | *0,003393* | *0,004023* | *0,000199* | *0,000249* | 15,29 | 0,0484 | 2,89 | 0,0048 | -0,25 | 0,8073 |  |  |
| *trade* | *0,003814* | *0,002752* | *0,000180* | *0,000188* | 17,05 | 0,0152 | -4,22 | 0,0000 | 1,74 | 0,0930 |  |  |
| *staying* | *0,003361* | *0,004619* | *0,000098* | *0,000165* | 17,14 | 0,0144 | 4,29 | 0,0000 | -1,83 | 0,0639 |  |  |
| *beard* | *0,004424* | *0,002247* | *0,000194* | *0,000134* | 42,15 | 0,0000 | -5,45 | 0,0000 | 1,21 | 0,2204 |  |  |
| *approach* | *0,003996* | *0,002709* | *0,000130* | *0,000131* | 25,29 | 0,0002 | -3,84 | 0,0002 | 0,47 | 0,6305 |  |  |
| *birthday* | *0,003134* | *0,005819* | *0,000197* | *0,000340* | 25,60 | 0,0002 | 4,41 | 0,0000 | -1,14 | 0,2378 |  |  |
| *traffic* | *0,004261* | *0,003113* | *0,000227* | *0,000186* | 23,57 | 0,0004 | -1,29 | 0,1522 | -2,12 | 0,0274 |  |  |
| *team* | *0,003892* | *0,003161* | *0,000268* | *0,000329* | 17,19 | 0,0139 | -0,21 | 0,8141 | -2,64 | 0,0059 |  |  |
| *numbers* | *0,004103* | *0,003220* | *0,000185* | *0,000210* | 23,31 | 0,0005 | -4,12 | 0,0000 | 0,98 | 0,3324 |  |  |
|  |  |  |  |  |  |  |  |  |  |  |  |  |
| *loving* | *0,002478* | *0,004046* | *0,000105* | *0,000353* | 31,42 | 0,0000 | 6,23 | 0,0000 | -3,09 | 0,0030 |  |  |
| *base* | *0,003819* | *0,002394* | *0,000182* | *0,000141* | 23,15 | 0,0005 | -5,09 | 0,0000 | 2,29 | 0,0240 |  |  |
| *curtains* | *0,003253* | *0,004662* | *0,000174* | *0,000218* | 21,91 | 0,0010 | 4,53 | 0,0000 | -1,64 | 0,0951 |  |  |
| *emerged* | *0,003822* | *0,002612* | *0,000148* | *0,000138* | 18,76 | 0,0054 | -1,17 | 0,2302 | -1,88 | 0,0598 |  |  |
|  |  |  |  |  |  |  |  |  |  |  |  |  |
| *original* | *0,003825* | *0,002402* | *0,000140* | *0,000117* | 28,97 | 0,0000 | -2,21 | 0,0307 | -1,58 | 0,1261 |  |  |
|  |  |  |  |  |  |  |  |  |  |  |  |  |
| *process* | *0,003833* | *0,002950* | *0,000164* | *0,000188* | 18,83 | 0,0052 | -3,31 | 0,0008 | 0,41 | 0,6869 |  |  |
| *shocked* | *0,002584* | *0,004048* | *0,000100* | *0,000174* | 16,41 | 0,0229 | 3,25 | 0,0010 | -0,58 | 0,5773 |  |  |
| *sergeant* | *0,004377* | *0,001569* | *0,000521* | *0,000635* | 40,87 | 0,0000 | -2,89 | 0,0032 | -1,60 | 0,1046 |  |  |
| *loves* | *0,002814* | *0,004327* | *0,000121* | *0,000223* | 31,34 | 0,0000 | 6,26 | 0,0000 | -3,14 | 0,0015 |  |  |
| *western* | *0,003528* | *0,002352* | *0,000232* | *0,000181* | 15,89 | 0,0322 | -3,34 | 0,0009 | 0,74 | 0,4674 |  |  |
| *wheel* | *0,003772* | *0,002920* | *0,000151* | *0,000234* | 17,38 | 0,0124 | -2,38 | 0,0178 | -0,52 | 0,6187 |  |  |
| *sixty* | *0,003502* | *0,002462* | *0,000120* | *0,000130* | 19,68 | 0,0032 | -3,13 | 0,0025 | 0,12 | 0,9013 |  |  |
| *engine* | *0,004083* | *0,002476* | *0,000220* | *0,000177* | 23,63 | 0,0004 | -2,58 | 0,0092 | -0,82 | 0,3982 |  |  |
| *mile* | *0,003729* | *0,002214* | *0,000199* | *0,000154* | 21,61 | 0,0012 | -2,84 | 0,0056 | -0,37 | 0,7101 |  |  |
| *nation* | *0,003182* | *0,002401* | *0,000224* | *0,000292* | 15,44 | 0,0436 | -2,20 | 0,0320 | -0,54 | 0,5855 |  |  |
| *rear* | *0,003839* | *0,001909* | *0,000185* | *0,000152* | 45,20 | 0,0000 | -3,74 | 0,0002 | -0,94 | 0,3268 |  |  |
| *pillow* | *0,002892* | *0,004568* | *0,000110* | *0,000205* | 30,61 | 0,0000 | 3,43 | 0,0006 | 0,39 | 0,6787 |  |  |
| *skirt* | *0,002692* | *0,005438* | *0,000144* | *0,000305* | 43,37 | 0,0000 | 4,72 | 0,0000 | -0,26 | 0,7875 |  |  |
| *total* | *0,003525* | *0,002542* | *0,000147* | *0,000149* | 16,92 | 0,0165 | -0,81 | 0,4030 | -2,07 | 0,0359 |  |  |
| *basket* | *0,002747* | *0,004348* | *0,000157* | *0,000273* | 30,41 | 0,0000 | 4,58 | 0,0000 | -0,96 | 0,3510 |  |  |
|  |  |  |  |  |  |  |  |  |  |  |  |  |
| *commander* | *0,003575* | *0,001688* | *0,000554* | *0,000404* | 22,94 | 0,0006 | -2,86 | 0,0037 | -0,46 | 0,6423 |  |  |
| *cake* | *0,002192* | *0,006152* | *0,000129* | *0,001010* | 39,61 | 0,0000 | 5,79 | 0,0000 | -1,81 | 0,0828 |  |  |
| *district* | *0,003361* | *0,002535* | *0,000243* | *0,000452* | 20,19 | 0,0025 | -2,88 | 0,0025 | -0,21 | 0,8187 |  |  |
| *armed* | *0,003366* | *0,001700* | *0,000163* | *0,000116* | 34,26 | 0,0000 | -3,97 | 0,0000 | -0,03 | 0,9739 |  |  |
| *bastard* | *0,003096* | *0,001931* | *0,000202* | *0,000159* | 20,11 | 0,0026 | -1,33 | 0,1380 | -1,82 | 0,0508 |  |  |
|  |  |  |  |  |  |  |  |  |  |  |  |  |

Page 5

| *pretend* | *0,002717* | *0,003928* | *0,000099* | *0,000158* | 16,86 | 0,0172 | 1,97 | 0,0495 | 0,91 | 0,3444 |  |  |
| --- | --- | --- | --- | --- | --- | --- | --- | --- | --- | --- | --- | --- |
| *right-hand* | *0,003486* | *0,002150* | *0,000155* | *0,000114* | 20,57 | 0,0020 | -3,67 | 0,0004 | 0,67 | 0,4994 |  |  |
| *guns* | *0,003409* | *0,002307* | *0,000239* | *0,000215* | 17,70 | 0,0102 | -3,11 | 0,0028 | 0,28 | 0,7737 |  |  |
| *cared* | *0,002439* | *0,003650* | *0,000088* | *0,000159* | 24,28 | 0,0003 | 4,77 | 0,0000 | -1,73 | 0,0909 |  |  |
| *central* | *0,003581* | *0,002414* | *0,000200* | *0,000188* | 26,22 | 0,0001 | -1,79 | 0,0672 | -1,82 | 0,0670 |  |  |
|  |  |  |  |  |  |  |  |  |  |  |  |  |
| *tray* | *0,002606* | *0,004051* | *0,000123* | *0,000200* | 15,68 | 0,0371 | 3,39 | 0,0008 | -0,81 | 0,4104 |  |  |
|  |  |  |  |  |  |  |  |  |  |  |  |  |
| *fired* | *0,003545* | *0,001805* | *0,000157* | *0,000112* | 35,41 | 0,0000 | -3,53 | 0,0005 | -0,59 | 0,5633 |  |  |
| *section* | *0,003563* | *0,002124* | *0,000201* | *0,000123* | 20,73 | 0,0018 | -2,72 | 0,0057 | -0,44 | 0,6553 |  |  |
| *advance* | *0,003403* | *0,001879* | *0,000218* | *0,000154* | 25,51 | 0,0002 | -4,27 | 0,0000 | 0,98 | 0,3276 |  |  |
| *forces* | *0,003233* | *0,002138* | *0,000213* | *0,000193* | 16,07 | 0,0286 | -3,13 | 0,0012 | 0,46 | 0,6491 |  |  |
| *pregnant* | *0,002334* | *0,005095* | *0,000144* | *0,000450* | 15,91 | 0,0319 | -0,72 | 0,3856 | 3,33 | 0,0007 |  |  |
| *governor* | *0,002738* | *0,001276* | *0,000282* | *0,000215* | 15,61 | 0,0390 | -2,23 | 0,0343 | -0,52 | 0,6089 |  |  |
|  |  |  |  |  |  |  |  |  |  |  |  |  |
| *cooking* | *0,002599* | *0,004324* | *0,000149* | *0,000211* | 22,73 | 0,0007 | 2,99 | 0,0034 | 0,30 | 0,7651 |  |  |
| *recent* | *0,002960* | *0,002283* | *0,000104* | *0,000124* | 15,35 | 0,0466 | -1,41 | 0,1543 | -1,35 | 0,1758 |  |  |
|  |  |  |  |  |  |  |  |  |  |  |  |  |
| *rifle* | *0,003758* | *0,001473* | *0,000346* | *0,000198* | 21,27 | 0,0014 | -3,04 | 0,0015 | -0,12 | 0,8689 |  |  |
|  |  |  |  |  |  |  |  |  |  |  |  |  |
| *clerk* | *0,003102* | *0,001329* | *0,000262* | *0,000110* | 17,22 | 0,0137 | -4,11 | 0,0001 | 1,58 | 0,1164 |  |  |
| *forms* | *0,003118* | *0,001924* | *0,000141* | *0,000118* | 20,32 | 0,0023 | -4,36 | 0,0000 | 1,57 | 0,1280 |  |  |
| *el* | *0,004246* | *0,001877* | *0,001230* | *0,000437* | 17,79 | 0,0096 | -1,77 | 0,0562 | -1,19 | 0,2278 |  |  |
|  |  |  |  |  |  |  |  |  |  |  |  |  |
| *goodness* | *0,002168* | *0,003056* | *0,000140* | *0,000207* | 17,19 | 0,0139 | 4,23 | 0,0000 | -1,74 | 0,0785 |  |  |
|  |  |  |  |  |  |  |  |  |  |  |  |  |
| *mothers* | *0,002331* | *0,004275* | *0,000130* | *0,000251* | 29,67 | 0,0000 | 3,19 | 0,0014 | 0,59 | 0,5449 |  |  |
| *troops* | *0,002991* | *0,001607* | *0,000339* | *0,000293* | 20,79 | 0,0018 | -2,22 | 0,0318 | -0,98 | 0,3398 |  |  |
| *parked* | *0,003076* | *0,002493* | *0,000170* | *0,000164* | 19,78 | 0,0031 | 0,72 | 0,3560 | -3,64 | 0,0000 |  |  |
| *dishes* | *0,002384* | *0,003724* | *0,000099* | *0,000175* | 24,80 | 0,0003 | 3,19 | 0,0013 | 0,24 | 0,8103 |  |  |
|  |  |  |  |  |  |  |  |  |  |  |  |  |
| *agent* | *0,002804* | *0,001661* | *0,000191* | *0,000171* | 17,08 | 0,0149 | -2,24 | 0,0267 | -0,64 | 0,5111 |  |  |
| *roses* | *0,001974* | *0,003922* | *0,000131* | *0,000226* | 51,39 | 0,0000 | 7,24 | 0,0000 | -2,92 | 0,0046 |  |  |
| *inspector* | *0,003160* | *0,000882* | *0,000685* | *0,000206* | 21,19 | 0,0014 | -1,02 | 0,3060 | -2,21 | 0,0226 |  |  |
| *crew* | *0,002906* | *0,001523* | *0,000264* | *0,000148* | 20,76 | 0,0018 | -2,94 | 0,0029 | -0,19 | 0,8581 |  |  |
|  |  |  |  |  |  |  |  |  |  |  |  |  |
| *signal* | *0,002772* | *0,001708* | *0,000126* | *0,000096* | 20,12 | 0,0025 | -3,26 | 0,0024 | 0,22 | 0,8141 |  |  |
| *pistol* | *0,002934* | *0,001015* | *0,000217* | *0,000128* | 33,30 | 0,0000 | -2,09 | 0,0409 | -1,98 | 0,0514 |  |  |
| *range* | *0,002909* | *0,001797* | *0,000155* | *0,000137* | 28,52 | 0,0001 | -3,09 | 0,0017 | -0,62 | 0,5443 |  |  |
| *route* | *0,003054* | *0,002045* | *0,000133* | *0,000135* | 28,81 | 0,0000 | -3,35 | 0,0006 | -0,35 | 0,7115 |  |  |
| *babies* | *0,001961* | *0,004345* | *0,000203* | *0,000297* | 29,63 | 0,0000 | 3,60 | 0,0000 | 0,13 | 0,9101 |  |  |
| *shy* | *0,002199* | *0,002885* | *0,000085* | *0,000122* | 15,26 | 0,0495 | 4,94 | 0,0000 | -3,13 | 0,0029 |  |  |
| *blade* | *0,002554* | *0,001749* | *0,000138* | *0,000125* | 15,68 | 0,0371 | -3,76 | 0,0001 | 1,29 | 0,1867 |  |  |
| *operation* | *0,002676* | *0,001813* | *0,000121* | *0,000186* | 24,66 | 0,0003 | -2,06 | 0,0413 | -1,43 | 0,1549 |  |  |
| *lessons* | *0,001995* | *0,002986* | *0,000109* | *0,000178* | 15,37 | 0,0458 | 4,23 | 0,0000 | -1,98 | 0,0462 |  |  |
| *weapons* | *0,002817* | *0,001527* | *0,000258* | *0,000137* | 19,92 | 0,0028 | -2,51 | 0,0109 | -0,59 | 0,5523 |  |  |
| *per* | *0,003012* | *0,001690* | *0,000234* | *0,000134* | 21,46 | 0,0013 | -3,12 | 0,0015 | -0,05 | 0,9601 |  |  |
| *left-hand* | *0,002774* | *0,001537* | *0,000130* | *0,000089* | 26,92 | 0,0001 | -3,09 | 0,0012 | -0,51 | 0,5931 |  |  |
| *mounted* | *0,002535* | *0,001461* | *0,000158* | *0,000100* | 18,20 | 0,0075 | -0,64 | 0,5407 | -2,33 | 0,0177 |  |  |
| *longing* | *0,002042* | *0,003029* | *0,000115* | *0,000168* | 37,14 | 0,0000 | 6,21 | 0,0000 | -2,55 | 0,0151 |  |  |
|  |  |  |  |  |  |  |  |  |  |  |  |  |
| *banks* | *0,002988* | *0,001983* | *0,000213* | *0,000349* | 18,37 | 0,0068 | -2,24 | 0,0264 | -0,75 | 0,4572 |  |  |
| *slope* | *0,002913* | *0,001457* | *0,000165* | *0,000121* | 18,47 | 0,0064 | -2,80 | 0,0065 | -0,15 | 0,8815 |  |  |
| *velvet* | *0,001831* | *0,002832* | *0,000097* | *0,000164* | 20,72 | 0,0018 | 2,67 | 0,0118 | 0,49 | 0,6065 |  |  |
| *seventy* | *0,002573* | *0,001738* | *0,000101* | *0,000095* | 17,05 | 0,0153 | -1,74 | 0,0862 | -1,16 | 0,2392 |  |  |
|  |  |  |  |  |  |  |  |  |  |  |  |  |
| *shopping* | *0,002188* | *0,003854* | *0,000115* | *0,000230* | 21,44 | 0,0013 | 5,39 | 0,0000 | -2,93 | 0,0013 |  |  |
|  |  |  |  |  |  |  |  |  |  |  |  |  |
| *tobacco* | *0,002805* | *0,001727* | *0,000154* | *0,000181* | 17,00 | 0,0157 | -2,09 | 0,0387 | -0,80 | 0,4336 |  |  |
| *nails* | *0,002245* | *0,003222* | *0,000091* | *0,000150* | 15,93 | 0,0314 | 0,41 | 0,6723 | 2,36 | 0,0184 |  |  |
| *roar* | *0,002551* | *0,001639* | *0,000114* | *0,000092* | 15,86 | 0,0330 | -2,62 | 0,0122 | -0,11 | 0,9041 |  |  |
|  |  |  |  |  |  |  |  |  |  |  |  |  |
| *fellows* | *0,002559* | *0,001047* | *0,000202* | *0,000128* | 19,81 | 0,0030 | -4,89 | 0,0000 | 2,38 | 0,0134 |  |  |
| *weapon* | *0,002603* | *0,001322* | *0,000161* | *0,000116* | 23,98 | 0,0004 | -3,71 | 0,0001 | 0,43 | 0,6733 |  |  |
| *resumed* | *0,002181* | *0,001351* | *0,000102* | *0,000133* | 16,19 | 0,0265 | -2,17 | 0,0353 | -0,63 | 0,5279 |  |  |
| *established* | *0,002421* | *0,001497* | *0,000098* | *0,000101* | 19,80 | 0,0030 | -1,53 | 0,1364 | -1,61 | 0,1135 |  |  |
|  |  |  |  |  |  |  |  |  |  |  |  |  |
| *cups* | *0,002130* | *0,003093* | *0,000103* | *0,000144* | 15,69 | 0,0369 | 4,25 | 0,0000 | -1,96 | 0,0528 |  |  |
|  |  |  |  |  |  |  |  |  |  |  |  |  |
| *reported* | *0,002465* | *0,001567* | *0,000122* | *0,000096* | 16,87 | 0,0170 | -3,98 | 0,0000 | 1,44 | 0,1432 |  |  |

Page 6

| *butter* | *0,001918* | *0,003283* | *0,000100* | *0,000193* | 23,70 | 0,0004 | 4,52 | 0,0000 | -1,46 | 0,1630 |  |  |
| --- | --- | --- | --- | --- | --- | --- | --- | --- | --- | --- | --- | --- |
| *arrest* | *0,002525* | *0,001420* | *0,000155* | *0,000115* | 16,47 | 0,0220 | -3,87 | 0,0000 | 1,34 | 0,1856 |  |  |
| *chocolate* | *0,002094* | *0,003817* | *0,000276* | *0,000293* | 16,14 | 0,0273 | 0,82 | 0,3758 | 1,99 | 0,0396 |  |  |
| *cream* | *0,001767* | *0,003584* | *0,000083* | *0,000188* | 30,06 | 0,0000 | 3,12 | 0,0015 | 0,69 | 0,4970 |  |  |
|  |  |  |  |  |  |  |  |  |  |  |  |  |
| *charged* | *0,002184* | *0,001512* | *0,000081* | *0,000081* | 18,68 | 0,0057 | -3,24 | 0,0020 | 0,34 | 0,7351 |  |  |
| *senior* | *0,001998* | *0,001538* | *0,000128* | *0,000146* | 17,71 | 0,0101 | -0,02 | 0,9917 | -2,86 | 0,0032 |  |  |
| *bullet* | *0,002718* | *0,001546* | *0,000166* | *0,000194* | 24,91 | 0,0002 | -1,48 | 0,1214 | -2,04 | 0,0419 |  |  |
| *dresses* | *0,001603* | *0,003565* | *0,000080* | *0,000222* | 49,33 | 0,0000 | 5,52 | 0,0000 | -0,85 | 0,3966 |  |  |
|  |  |  |  |  |  |  |  |  |  |  |  |  |
| *mister* | *0,002458* | *0,001037* | *0,000620* | *0,000247* | 20,68 | 0,0019 | -2,53 | 0,0075 | -0,64 | 0,5001 |  |  |
| *northern* | *0,002295* | *0,001748* | *0,000168* | *0,000232* | 17,55 | 0,0112 | -1,50 | 0,1323 | -1,45 | 0,1521 |  |  |
| *doll* | *0,001537* | *0,003042* | *0,000155* | *0,000417* | 33,03 | 0,0000 | 5,11 | 0,0000 | -1,43 | 0,1480 |  |  |
| *attacked* | *0,002052* | *0,001443* | *0,000092* | *0,000111* | 15,83 | 0,0335 | -2,17 | 0,0295 | -0,60 | 0,5415 |  |  |
| *authorities* | *0,002521* | *0,001227* | *0,000183* | *0,000123* | 24,14 | 0,0003 | -4,45 | 0,0000 | 1,32 | 0,1843 |  |  |
| *based* | *0,002291* | *0,001558* | *0,000113* | *0,000098* | 15,28 | 0,0488 | -1,76 | 0,0671 | -0,98 | 0,3280 |  |  |
| *longed* | *0,001561* | *0,002438* | *0,000093* | *0,000150* | 32,23 | 0,0000 | 6,71 | 0,0000 | -3,75 | 0,0000 |  |  |
| *completed* | *0,002235* | *0,001368* | *0,000095* | *0,000083* | 20,36 | 0,0022 | -2,24 | 0,0271 | -0,92 | 0,3582 |  |  |
| *offices* | *0,002065* | *0,001352* | *0,000108* | *0,000095* | 15,79 | 0,0344 | -2,32 | 0,0244 | -0,44 | 0,6425 |  |  |
| *interior* | *0,002273* | *0,001314* | *0,000109* | *0,000089* | 20,26 | 0,0024 | -3,17 | 0,0013 | 0,11 | 0,8995 |  |  |
| *lace* | *0,001329* | *0,002904* | *0,000073* | *0,000186* | 48,65 | 0,0000 | 5,29 | 0,0000 | -0,62 | 0,5335 |  |  |
| *eastern* | *0,002071* | *0,001330* | *0,000106* | *0,000105* | 17,56 | 0,0111 | -3,62 | 0,0003 | 0,90 | 0,3736 |  |  |
| *anxiously* | *0,001665* | *0,001949* | *0,000087* | *0,000121* | 15,73 | 0,0358 | 5,27 | 0,0000 | -3,70 | 0,0000 |  |  |
| *complex* | *0,002175* | *0,001399* | *0,000134* | *0,000097* | 15,56 | 0,0403 | -1,05 | 0,2340 | -1,72 | 0,0733 |  |  |
| *investigation* | *0,002159* | *0,000846* | *0,000190* | *0,000104* | 16,30 | 0,0245 | -2,52 | 0,0109 | -0,27 | 0,7865 |  |  |
| *column* | *0,002107* | *0,001360* | *0,000165* | *0,000140* | 19,93 | 0,0028 | -2,94 | 0,0039 | -0,12 | 0,9087 |  |  |
| *indicated* | *0,002002* | *0,001210* | *0,000091* | *0,000081* | 15,55 | 0,0406 | -1,88 | 0,0600 | -0,89 | 0,3944 |  |  |
| *covers* | *0,001842* | *0,002674* | *0,000084* | *0,000134* | 17,43 | 0,0120 | -0,73 | 0,4458 | 3,46 | 0,0011 |  |  |
| *cock* | *0,001999* | *0,001141* | *0,000234* | *0,000203* | 19,37 | 0,0039 | -2,87 | 0,0047 | -0,15 | 0,8695 |  |  |
| *player* | *0,002330* | *0,001468* | *0,000196* | *0,000107* | 19,59 | 0,0034 | -1,08 | 0,2272 | -2,03 | 0,0410 |  |  |
| *scarf* | *0,001619* | *0,002896* | *0,000129* | *0,000246* | 15,66 | 0,0376 | 1,61 | 0,0799 | 1,18 | 0,2192 |  |  |
| *aspect* | *0,001763* | *0,001070* | *0,000097* | *0,000093* | 16,42 | 0,0228 | -2,57 | 0,0147 | -0,22 | 0,8253 |  |  |
| *liquor* | *0,001986* | *0,001327* | *0,000147* | *0,000134* | 15,24 | 0,0500 | -4,22 | 0,0000 | 1,98 | 0,0522 |  |  |
| *vehicle* | *0,002151* | *0,001135* | *0,000173* | *0,000118* | 31,24 | 0,0000 | -1,60 | 0,0926 | -2,33 | 0,0228 |  |  |
| *pilot* | *0,001694* | *0,000777* | *0,000172* | *0,000129* | 22,56 | 0,0007 | -1,86 | 0,0547 | -1,48 | 0,1329 |  |  |
| *barrel* | *0,001983* | *0,001169* | *0,000097* | *0,000095* | 25,81 | 0,0002 | -3,35 | 0,0006 | -0,14 | 0,8975 |  |  |
| *headquarters* | *0,001994* | *0,000868* | *0,000213* | *0,000149* | 19,53 | 0,0035 | -1,90 | 0,0521 | -1,21 | 0,2026 |  |  |
| *shots* | *0,002056* | *0,001048* | *0,000111* | *0,000072* | 21,20 | 0,0014 | -2,72 | 0,0062 | -0,47 | 0,6223 |  |  |
| *proceed* | *0,001530* | *0,000903* | *0,000080* | *0,000086* | 15,54 | 0,0409 | -3,06 | 0,0040 | 0,43 | 0,6845 |  |  |
| *niece* | *0,000905* | *0,001860* | *0,000082* | *0,000185* | 24,39 | 0,0003 | 4,39 | 0,0000 | -1,22 | 0,2330 |  |  |
| *division* | *0,001760* | *0,001154* | *0,000171* | *0,000213* | 16,28 | 0,0250 | -1,22 | 0,2228 | -1,62 | 0,1100 |  |  |
| *skirts* | *0,001384* | *0,002157* | *0,000097* | *0,000138* | 23,03 | 0,0006 | 2,76 | 0,0084 | 0,57 | 0,5771 |  |  |
| *cop* | *0,002236* | *0,000817* | *0,000298* | *0,000140* | 30,28 | 0,0000 | -1,63 | 0,0382 | -2,24 | 0,0157 |  |  |
| *spoon* | *0,001400* | *0,002303* | *0,000085* | *0,000139* | 20,92 | 0,0017 | 0,96 | 0,3014 | 2,25 | 0,0215 |  |  |
| *cells* | *0,001599* | *0,002439* | *0,000171* | *0,001431* | 16,13 | 0,0274 | -2,87 | 0,0013 | 0,14 | 0,8667 |  |  |
| *dealing* | *0,001687* | *0,001194* | *0,000067* | *0,000073* | 15,61 | 0,0388 | -1,03 | 0,2834 | -1,75 | 0,0844 |  |  |
| *rug* | *0,001376* | *0,002570* | *0,000089* | *0,000250* | 19,12 | 0,0044 | 4,16 | 0,0000 | -1,44 | 0,1516 |  |  |
| *louise* | *0,001006* | *0,004164* | *0,000403* | *0,001597* | 18,03 | 0,0084 | 5,63 | 0,0000 | -3,93 | 0,0000 |  |  |
| *cops* | *0,001962* | *0,000916* | *0,000209* | *0,000170* | 21,81 | 0,0010 | -1,39 | 0,0765 | -1,90 | 0,0405 |  |  |
| *examination* | *0,001767* | *0,000937* | *0,000093* | *0,000079* | 16,63 | 0,0199 | -3,92 | 0,0001 | 1,39 | 0,1734 |  |  |
| *bullets* | *0,002135* | *0,000971* | *0,000174* | *0,000112* | 27,22 | 0,0001 | -2,14 | 0,0254 | -1,54 | 0,1242 |  |  |
| *engineer* | *0,001967* | *0,000724* | *0,000223* | *0,000110* | 22,46 | 0,0008 | -2,14 | 0,0302 | -1,19 | 0,2318 |  |  |
| *aboard* | *0,001603* | *0,000575* | *0,000173* | *0,000066* | 15,54 | 0,0408 | -2,55 | 0,0115 | -0,16 | 0,8551 |  |  |
| *previously* | *0,001739* | *0,000936* | *0,000087* | *0,000073* | 18,72 | 0,0056 | -2,34 | 0,0227 | -0,67 | 0,5167 |  |  |
| *ridge* | *0,001907* | *0,000901* | *0,000184* | *0,000156* | 21,04 | 0,0016 | -2,50 | 0,0168 | -0,70 | 0,4780 |  |  |
| *marrying* | *0,000986* | *0,001783* | *0,000054* | *0,000166* | 17,10 | 0,0147 | 4,29 | 0,0001 | -1,84 | 0,0768 |  |  |
| *curls* | *0,000996* | *0,002290* | *0,000057* | *0,000148* | 37,43 | 0,0000 | 4,52 | 0,0000 | -0,40 | 0,6963 |  |  |

Page 7

| *commit* | *0,001618* | *0,000993* | *0,000074* | *0,000069* | 18,84 | 0,0052 | -2,60 | 0,0132 | -0,41 | 0,6729 |
| --- | --- | --- | --- | --- | --- | --- | --- | --- | --- | --- |
| *policemen* | *0,001834* | *0,001013* | *0,000176* | *0,000119* | 16,49 | 0,0218 | -1,88 | 0,0380 | -0,97 | 0,3064 |
| *unit* | *0,001714* | *0,001144* | *0,000139* | *0,000149* | 19,29 | 0,0040 | -1,53 | 0,0613 | -1,57 | 0,0907 |
| *firing* | *0,001880* | *0,000663* | *0,000167* | *0,000064* | 32,60 | 0,0000 | -2,38 | 0,0181 | -1,63 | 0,0942 |
| *apron* | *0,001239* | *0,001977* | *0,000076* | *0,000130* | 17,06 | 0,0151 | 4,29 | 0,0000 | -1,85 | 0,0616 |
| *speaker* | *0,001202* | *0,000807* | *0,000085* | *0,000107* | 17,07 | 0,0150 | -3,05 | 0,0031 | 0,27 | 0,8061 |
| *roofs* | *0,001655* | *0,000963* | *0,000086* | *0,000065* | 16,00 | 0,0300 | -1,97 | 0,0482 | -0,83 | 0,4126 |
| *selfish* | *0,001062* | *0,001679* | *0,000063* | *0,000102* | 15,76 | 0,0351 | 4,12 | 0,0000 | -1,77 | 0,0917 |
| *ribbon* | *0,001126* | *0,001812* | *0,000058* | *0,000135* | 17,28 | 0,0132 | 2,90 | 0,0042 | -0,07 | 0,9601 |
| *butt* | *0,001589* | *0,001280* | *0,000098* | *0,000118* | 15,98 | 0,0303 | -4,66 | 0,0000 | 2,54 | 0,0118 |
| *sewing* | *0,000978* | *0,002561* | *0,000068* | *0,000236* | 35,19 | 0,0000 | 5,20 | 0,0000 | -1,37 | 0,1645 |
| *careless* | *0,001063* | *0,001412* | *0,000058* | *0,000080* | 15,91 | 0,0318 | 4,58 | 0,0000 | -2,42 | 0,0153 |
| *mob* | *0,001296* | *0,000583* | *0,000090* | *0,000058* | 18,79 | 0,0054 | -4,27 | 0,0000 | 1,62 | 0,1058 |
| *holidays* | *0,001134* | *0,001960* | *0,000071* | *0,000125* | 21,46 | 0,0013 | 5,33 | 0,0000 | -2,83 | 0,0051 |
| *deputy* | *0,001514* | *0,000534* | *0,000196* | *0,000103* | 25,71 | 0,0002 | -2,97 | 0,0018 | -0,55 | 0,5673 |
| *shawl* | *0,000979* | *0,001808* | *0,000066* | *0,000178* | 17,90 | 0,0090 | 5,69 | 0,0000 | -4,08 | 0,0000 |
| *explosion* | *0,001475* | *0,000976* | *0,000084* | *0,000070* | 15,55 | 0,0405 | -1,44 | 0,1254 | -1,34 | 0,1725 |
| *satin* | *0,000746* | *0,001906* | *0,000064* | *0,000141* | 45,95 | 0,0000 | 5,52 | 0,0000 | -1,06 | 0,2858 |
| *ranks* | *0,001437* | *0,000763* | *0,000084* | *0,000072* | 21,69 | 0,0011 | -1,23 | 0,2364 | -2,05 | 0,0442 |
| *paces* | *0,001447* | *0,000732* | *0,000089* | *0,000071* | 19,94 | 0,0028 | -5,32 | 0,0000 | 3,01 | 0,0039 |
| *legend* | *0,001439* | *0,000817* | *0,000087* | *0,000070* | 17,76 | 0,0098 | -1,17 | 0,2486 | -1,80 | 0,0737 |
| *imperial* | *0,001407* | *0,000592* | *0,000137* | *0,000096* | 16,67 | 0,0194 | -2,66 | 0,0110 | -0,14 | 0,8759 |
| *players* | *0,001451* | *0,000771* | *0,000110* | *0,000064* | 17,72 | 0,0101 | -0,61 | 0,5129 | -2,32 | 0,0198 |
| *areas* | *0,001386* | *0,001050* | *0,000081* | *0,000100* | 17,88 | 0,0091 | -0,26 | 0,7507 | -2,66 | 0,0054 |
| *tenderly* | *0,001106* | *0,001378* | *0,000069* | *0,000104* | 16,19 | 0,0265 | 5,14 | 0,0000 | -3,32 | 0,0001 |
| *curly* | *0,001062* | *0,001653* | *0,000080* | *0,000142* | 15,27 | 0,0491 | 4,30 | 0,0000 | -2,09 | 0,0390 |
| *recorded* | *0,001405* | *0,000990* | *0,000077* | *0,000094* | 16,06 | 0,0288 | -2,37 | 0,0205 | -0,41 | 0,6769 |
| *embroidered* | *0,000979* | *0,001421* | *0,000063* | *0,000103* | 19,57 | 0,0034 | 2,45 | 0,0123 | 0,63 | 0,5159 |
| *agents* | *0,001270* | *0,000666* | *0,000099* | *0,000071* | 16,80 | 0,0178 | -1,11 | 0,2546 | -1,77 | 0,0829 |
| *gear* | *0,001497* | *0,000868* | *0,000103* | *0,000082* | 28,70 | 0,0000 | -2,28 | 0,0120 | -1,49 | 0,1185 |
| *assault* | *0,001196* | *0,000789* | *0,000075* | *0,000088* | 15,53 | 0,0412 | -2,13 | 0,0326 | -0,62 | 0,5375 |
| *piss* | *0,001384* | *0,001031* | *0,000098* | *0,000108* | 23,57 | 0,0004 | -0,58 | 0,4090 | -2,79 | 0,0024 |
| *administration* | *0,001120* | *0,000777* | *0,000088* | *0,000157* | 20,76 | 0,0018 | -4,00 | 0,0000 | 1,06 | 0,2906 |
| *pillows* | *0,000912* | *0,001807* | *0,000052* | *0,000102* | 34,83 | 0,0000 | 3,55 | 0,0003 | 0,54 | 0,6049 |
| *companies* | *0,001379* | *0,000690* | *0,000131* | *0,000081* | 18,56 | 0,0061 | -3,64 | 0,0000 | 0,83 | 0,4156 |
| *wrap* | *0,000972* | *0,001850* | *0,000048* | *0,000104* | 17,02 | 0,0155 | 2,08 | 0,0265 | 0,81 | 0,4166 |
| *bastards* | *0,001157* | *0,000627* | *0,000090* | *0,000074* | 24,41 | 0,0003 | -1,47 | 0,0735 | -2,01 | 0,0251 |
| *positions* | *0,001225* | *0,000878* | *0,000058* | *0,000073* | 19,75 | 0,0031 | -1,56 | 0,1014 | -1,57 | 0,1168 |
| *rifles* | *0,001532* | *0,000549* | *0,000139* | *0,000078* | 28,46 | 0,0001 | -3,27 | 0,0002 | -0,42 | 0,6799 |
| *squad* | *0,001374* | *0,000581* | *0,000136* | *0,000080* | 20,52 | 0,0020 | -1,35 | 0,1228 | -1,84 | 0,0511 |
| *originally* | *0,001248* | *0,000764* | *0,000070* | *0,000055* | 15,34 | 0,0467 | -1,62 | 0,1121 | -1,14 | 0,2592 |
| *location* | *0,001313* | *0,000834* | *0,000100* | *0,000076* | 18,03 | 0,0083 | -2,47 | 0,0045 | -0,48 | 0,6281 |
| *operations* | *0,001242* | *0,000624* | *0,000094* | *0,000076* | 15,86 | 0,0328 | -2,32 | 0,0217 | -0,45 | 0,6751 |
| *posts* | *0,001365* | *0,000736* | *0,000189* | *0,000067* | 17,40 | 0,0123 | -3,66 | 0,0003 | 0,98 | 0,3328 |
| *revolver* | *0,001572* | *0,000350* | *0,000137* | *0,000069* | 32,30 | 0,0000 | -4,50 | 0,0000 | 0,73 | 0,4264 |
| *bearded* | *0,001267* | *0,000622* | *0,000087* | *0,000051* | 22,50 | 0,0007 | -2,61 | 0,0084 | -0,70 | 0,4880 |
| *nursery* | *0,000786* | *0,001611* | *0,000121* | *0,000201* | 17,94 | 0,0088 | 4,31 | 0,0000 | -1,77 | 0,0743 |
| *lipstick* | *0,000911* | *0,001847* | *0,000080* | *0,000138* | 19,61 | 0,0034 | 0,13 | 0,8467 | 2,91 | 0,0021 |
| *vehicles* | *0,001298* | *0,000692* | *0,000104* | *0,000068* | 20,87 | 0,0017 | -1,47 | 0,0968 | -1,75 | 0,0734 |
| *parallel* | *0,001187* | *0,000683* | *0,000065* | *0,000061* | 21,93 | 0,0010 | -3,61 | 0,0002 | 0,48 | 0,6469 |
| *fuel* | *0,001218* | *0,000764* | *0,000138* | *0,000077* | 15,34 | 0,0468 | -3,71 | 0,0002 | 1,26 | 0,2104 |
| *drivers* | *0,001280* | *0,000797* | *0,000082* | *0,000091* | 24,97 | 0,0002 | -1,79 | 0,0473 | -1,73 | 0,0831 |
| *inspection* | *0,001201* | *0,000616* | *0,000077* | *0,000046* | 19,82 | 0,0030 | -2,02 | 0,0405 | -1,11 | 0,2690 |
| *pearls* | *0,000695* | *0,001180* | *0,000056* | *0,000101* | 18,94 | 0,0049 | 3,13 | 0,0029 | -0,19 | 0,8579 |
| *mother-in-law* | *0,000689* | *0,001583* | *0,000081* | *0,000260* | 15,34 | 0,0466 | 1,42 | 0,1408 | 1,34 | 0,1710 |

Page 8

| *scissors* | *0,000767* | *0,001416* | *0,000052* | *0,000110* | 17,84 | 0,0094 | 1,46 | 0,1280 | 1,51 | 0,1247 |
| --- | --- | --- | --- | --- | --- | --- | --- | --- | --- | --- |
| *patrol* | *0,001234* | *0,000507* | *0,000133* | *0,000081* | 23,51 | 0,0005 | -3,00 | 0,0013 | -0,34 | 0,7261 |
| *tidy* | *0,000791* | *0,001373* | *0,000043* | *0,000089* | 16,79 | 0,0179 | 4,22 | 0,0000 | -1,78 | 0,0765 |
| *warehouse* | *0,001027* | *0,000612* | *0,000103* | *0,000101* | 17,15 | 0,0143 | -1,51 | 0,0930 | -1,41 | 0,1459 |
| *madman* | *0,001144* | *0,000475* | *0,000143* | *0,000049* | 16,97 | 0,0160 | -4,43 | 0,0000 | 2,06 | 0,0344 |
| *stations* | *0,001086* | *0,000713* | *0,000075* | *0,000073* | 15,73 | 0,0359 | -3,72 | 0,0000 | 1,23 | 0,2112 |
| *trigger* | *0,001114* | *0,000597* | *0,000082* | *0,000057* | 21,94 | 0,0010 | -2,36 | 0,0081 | -0,92 | 0,3534 |
| *engines* | *0,001121* | *0,000547* | *0,000081* | *0,000052* | 20,55 | 0,0020 | -2,70 | 0,0047 | -0,45 | 0,6485 |
| *towels* | *0,000758* | *0,001412* | *0,000049* | *0,000098* | 15,27 | 0,0492 | 1,96 | 0,0278 | 0,77 | 0,4364 |
| *comforted* | *0,000732* | *0,001167* | *0,000046* | *0,000079* | 17,63 | 0,0106 | 3,74 | 0,0004 | -1,05 | 0,2998 |
| *adored* | *0,000624* | *0,001058* | *0,000043* | *0,000075* | 21,58 | 0,0012 | 4,30 | 0,0001 | -1,37 | 0,1778 |
| *cargo* | *0,000868* | *0,000493* | *0,000071* | *0,000054* | 15,55 | 0,0406 | -3,44 | 0,0005 | 0,90 | 0,3674 |
| *corporal* | *0,001258* | *0,000197* | *0,000194* | *0,000054* | 16,78 | 0,0181 | -3,26 | 0,0029 | 0,55 | 0,5941 |
| *industrial* | *0,001063* | *0,000573* | *0,000169* | *0,000075* | 18,03 | 0,0083 | -1,72 | 0,0532 | -1,27 | 0,1970 |
| *dolls* | *0,000721* | *0,001380* | *0,000092* | *0,000127* | 17,06 | 0,0152 | 3,95 | 0,0000 | -1,39 | 0,1549 |
| *lashes* | *0,000620* | *0,001207* | *0,000045* | *0,000100* | 25,69 | 0,0002 | 2,46 | 0,0160 | 1,09 | 0,2898 |
| *data* | *0,001036* | *0,000442* | *0,000109* | *0,000067* | 15,24 | 0,0499 | -2,16 | 0,0167 | -0,56 | 0,5633 |
| *interrogation* | *0,000955* | *0,000519* | *0,000120* | *0,000079* | 18,81 | 0,0053 | 1,50 | 0,0878 | -4,19 | 0,0000 |
| *politicians* | *0,000922* | *0,000590* | *0,000069* | *0,000070* | 16,84 | 0,0174 | -0,65 | 0,4810 | -2,21 | 0,0295 |
| *knitting* | *0,000598* | *0,001308* | *0,000061* | *0,000169* | 26,33 | 0,0001 | 5,31 | 0,0000 | -2,26 | 0,0232 |
| *device* | *0,000942* | *0,000510* | *0,000084* | *0,000070* | 24,11 | 0,0003 | -2,15 | 0,0302 | -1,30 | 0,1836 |
| *fucked* | *0,000997* | *0,000757* | *0,000101* | *0,000146* | 24,24 | 0,0003 | -0,61 | 0,3552 | -2,81 | 0,0021 |
| *bullshit* | *0,001024* | *0,000605* | *0,000108* | *0,000094* | 25,04 | 0,0002 | 0,15 | 0,8135 | -3,54 | 0,0000 |
| *teased* | *0,000585* | *0,001130* | *0,000038* | *0,000075* | 17,39 | 0,0123 | 2,57 | 0,0048 | 0,31 | 0,7541 |
| *artillery* | *0,001035* | *0,000407* | *0,000153* | *0,000142* | 18,05 | 0,0082 | -2,93 | 0,0078 | 0,03 | 0,9699 |
| *civilian* | *0,000893* | *0,000405* | *0,000095* | *0,000061* | 19,14 | 0,0044 | -1,41 | 0,1348 | -1,67 | 0,0805 |
| *earrings* | *0,000565* | *0,001324* | *0,000050* | *0,000170* | 18,68 | 0,0057 | 2,10 | 0,0240 | 0,93 | 0,3390 |
| *signals* | *0,000895* | *0,000507* | *0,000063* | *0,000043* | 15,41 | 0,0445 | -2,01 | 0,0366 | -0,73 | 0,4620 |
| *world-war* | *0,000831* | *0,000668* | *0,000064* | *0,000065* | 16,20 | 0,0263 | 1,98 | 0,0099 | -4,31 | 0,0000 |
| *clerks* | *0,000894* | *0,000378* | *0,000085* | *0,000043* | 15,32 | 0,0473 | -3,58 | 0,0004 | 1,10 | 0,2880 |
| *scolded* | *0,000583* | *0,000914* | *0,000043* | *0,000073* | 19,87 | 0,0029 | 4,92 | 0,0000 | -2,41 | 0,0163 |
| *eyelashes* | *0,000619* | *0,001116* | *0,000047* | *0,000082* | 19,85 | 0,0030 | 2,13 | 0,0231 | 1,00 | 0,3180 |
| *aircraft* | *0,000835* | *0,000236* | *0,000135* | *0,000049* | 18,48 | 0,0064 | -0,59 | 0,4610 | -2,40 | 0,0049 |
| *vicinity* | *0,000889* | *0,000393* | *0,000082* | *0,000055* | 15,98 | 0,0303 | -3,52 | 0,0008 | 0,95 | 0,3428 |
| *namely* | *0,000764* | *0,000261* | *0,000063* | *0,000047* | 15,67 | 0,0373 | -4,11 | 0,0002 | 1,77 | 0,0789 |
| *pissed* | *0,000748* | *0,000756* | *0,000061* | *0,000094* | 15,68 | 0,0372 | -0,73 | 0,2616 | -2,04 | 0,0239 |
| *circuit* | *0,000742* | *0,000329* | *0,000056* | *0,000033* | 23,81 | 0,0004 | -1,22 | 0,2242 | -2,21 | 0,0266 |
| *subsequent* | *0,000743* | *0,000395* | *0,000044* | *0,000042* | 24,00 | 0,0004 | -0,65 | 0,5413 | -2,76 | 0,0067 |
| *pistols* | *0,000723* | *0,000224* | *0,000063* | *0,000029* | 17,81 | 0,0095 | -2,25 | 0,0395 | -0,70 | 0,5113 |
| *terminal* | *0,000883* | *0,000481* | *0,000109* | *0,000094* | 20,84 | 0,0017 | -1,23 | 0,0971 | -1,98 | 0,0307 |
| *ammunition* | *0,000825* | *0,000313* | *0,000088* | *0,000039* | 16,28 | 0,0250 | -2,72 | 0,0067 | -0,04 | 0,9691 |
| *peas* | *0,000505* | *0,001139* | *0,000043* | *0,000113* | 20,94 | 0,0016 | 3,01 | 0,0035 | 0,13 | 0,8921 |
| *sentry* | *0,000841* | *0,000277* | *0,000122* | *0,000083* | 16,22 | 0,0260 | -3,38 | 0,0011 | 0,75 | 0,4470 |
| *nightgown* | *0,000470* | *0,001157* | *0,000046* | *0,000114* | 21,68 | 0,0011 | 2,52 | 0,0060 | 0,72 | 0,4384 |
| *tease* | *0,000509* | *0,000925* | *0,000035* | *0,000061* | 17,59 | 0,0109 | 4,28 | 0,0000 | -1,76 | 0,0753 |
| *units* | *0,000689* | *0,000742* | *0,000060* | *0,000357* | 18,04 | 0,0083 | -1,15 | 0,1774 | -1,84 | 0,0485 |
| *regulations* | *0,000764* | *0,000286* | *0,000070* | *0,000034* | 15,54 | 0,0409 | -2,93 | 0,0025 | 0,28 | 0,7665 |
| *forearm* | *0,000699* | *0,000574* | *0,000053* | *0,000058* | 16,62 | 0,0200 | -4,31 | 0,0000 | 1,93 | 0,0476 |
| *commerce* | *0,000667* | *0,000290* | *0,000046* | *0,000033* | 21,86 | 0,0010 | -3,27 | 0,0017 | 0,09 | 0,9371 |
| *shotgun* | *0,000758* | *0,000320* | *0,000085* | *0,000055* | 22,39 | 0,0008 | -1,25 | 0,1359 | -2,07 | 0,0204 |
| *teams* | *0,000696* | *0,000366* | *0,000061* | *0,000050* | 38,31 | 0,0000 | -0,30 | 0,7445 | -3,96 | 0,0001 |
| *charging* | *0,000652* | *0,000404* | *0,000039* | *0,000036* | 17,53 | 0,0113 | -1,21 | 0,2086 | -1,73 | 0,0831 |
| *vertical* | *0,000755* | *0,000363* | *0,000072* | *0,000034* | 16,72 | 0,0187 | -2,39 | 0,0082 | -0,44 | 0,6375 |
| *kitten* | *0,000499* | *0,000884* | *0,000091* | *0,000104* | 17,92 | 0,0089 | 4,87 | 0,0000 | -2,59 | 0,0092 |
| *cookies* | *0,000421* | *0,001123* | *0,000042* | *0,000128* | 16,51 | 0,0215 | 3,80 | 0,0000 | -1,25 | 0,1781 |

Page 9

| *preliminary* | *0,000612* | *0,000287* | *0,000042* | *0,000033* | 16,26 | 0,0253 | -2,50 | 0,0199 | -0,29 | 0,7589 |
| --- | --- | --- | --- | --- | --- | --- | --- | --- | --- | --- |
| *captains* | *0,000496* | *0,000146* | *0,000076* | *0,000027* | 16,28 | 0,0249 | -2,76 | 0,0108 | 0,01 | 0,9873 |
| *convoy* | *0,000739* | *0,000230* | *0,000129* | *0,000039* | 17,00 | 0,0157 | -1,13 | 0,2306 | -1,77 | 0,0756 |
| *apparatus* | *0,000786* | *0,000197* | *0,000154* | *0,000028* | 16,35 | 0,0238 | -3,17 | 0,0022 | 0,48 | 0,6351 |
| *lance* | *0,000579* | *0,000140* | *0,000089* | *0,000025* | 15,89 | 0,0322 | -3,65 | 0,0004 | 1,13 | 0,2504 |
| *legendary* | *0,000587* | *0,000353* | *0,000039* | *0,000036* | 18,60 | 0,0060 | 0,02 | 0,9817 | -2,96 | 0,0016 |
| *braid* | *0,000355* | *0,000881* | *0,000049* | *0,000081* | 34,35 | 0,0000 | 1,61 | 0,0893 | 2,51 | 0,0111 |
| *unkind* | *0,000347* | *0,000617* | *0,000030* | *0,000051* | 16,95 | 0,0162 | 4,35 | 0,0000 | -1,95 | 0,0632 |
| *surf* | *0,000613* | *0,000309* | *0,000052* | *0,000042* | 21,45 | 0,0013 | -2,23 | 0,0203 | -1,02 | 0,2998 |
| *sew* | *0,000348* | *0,000852* | *0,000027* | *0,000083* | 23,55 | 0,0004 | 4,17 | 0,0000 | -1,02 | 0,3112 |
| *embroidery* | *0,000298* | *0,000750* | *0,000027* | *0,000101* | 28,31 | 0,0001 | 4,72 | 0,0000 | -1,31 | 0,1893 |
| *veteran* | *0,000577* | *0,000236* | *0,000047* | *0,000044* | 30,60 | 0,0000 | -1,63 | 0,1043 | -2,27 | 0,0233 |
| *killers* | *0,000518* | *0,000263* | *0,000059* | *0,000038* | 17,59 | 0,0109 | -0,17 | 0,8025 | -2,71 | 0,0026 |
| *uniformed* | *0,000544* | *0,000337* | *0,000041* | *0,000043* | 20,42 | 0,0022 | -1,43 | 0,0875 | -1,75 | 0,0610 |
| *billion* | *0,000590* | *0,000250* | *0,000069* | *0,000045* | 18,64 | 0,0058 | -1,10 | 0,1610 | -1,93 | 0,0457 |
| *prettiest* | *0,000327* | *0,000593* | *0,000029* | *0,000050* | 23,74 | 0,0004 | 4,75 | 0,0000 | -1,76 | 0,0890 |
| *braids* | *0,000292* | *0,000890* | *0,000036* | *0,000092* | 35,56 | 0,0000 | 2,35 | 0,0081 | 1,85 | 0,0600 |
| *platoon* | *0,000550* | *0,000082* | *0,000122* | *0,000026* | 21,73 | 0,0011 | -2,31 | 0,0082 | -0,95 | 0,2992 |
| *scold* | *0,000321* | *0,000550* | *0,000029* | *0,000067* | 16,20 | 0,0262 | 3,78 | 0,0002 | -1,25 | 0,2280 |
| *diesel* | *0,000575* | *0,000166* | *0,000280* | *0,000029* | 18,59 | 0,0060 | -0,48 | 0,4668 | -2,52 | 0,0061 |
| *controls* | *0,000533* | *0,000234* | *0,000065* | *0,000042* | 15,60 | 0,0391 | -0,50 | 0,5639 | -2,25 | 0,0149 |
| *air-force* | *0,000400* | *0,000123* | *0,000052* | *0,000023* | 17,21 | 0,0138 | -0,79 | 0,2778 | -2,11 | 0,0135 |
| *high-heels* | *0,000339* | *0,000748* | *0,000033* | *0,000061* | 21,03 | 0,0016 | 1,81 | 0,0186 | 1,41 | 0,1271 |
| *investigating* | *0,000493* | *0,000235* | *0,000039* | *0,000026* | 15,74 | 0,0355 | -1,95 | 0,0357 | -0,83 | 0,3814 |
| *cylinder* | *0,000498* | *0,000167* | *0,000045* | *0,000025* | 21,43 | 0,0013 | -2,89 | 0,0014 | -0,30 | 0,7443 |
| *avenues* | *0,000521* | *0,000253* | *0,000047* | *0,000043* | 15,98 | 0,0304 | -1,88 | 0,0615 | -0,92 | 0,3632 |
| *violets* | *0,000305* | *0,000563* | *0,000040* | *0,000078* | 21,72 | 0,0011 | 4,27 | 0,0001 | -1,32 | 0,1852 |
| *facilities* | *0,000452* | *0,000247* | *0,000039* | *0,000030* | 20,15 | 0,0025 | -2,79 | 0,0023 | -0,31 | 0,7519 |
| *perimeter* | *0,000437* | *0,000291* | *0,000040* | *0,000038* | 17,62 | 0,0107 | -1,97 | 0,0035 | -0,98 | 0,2934 |
| *sewed* | *0,000265* | *0,000594* | *0,000024* | *0,000055* | 22,76 | 0,0007 | 3,08 | 0,0021 | 0,19 | 0,8381 |
| *opponents* | *0,000398* | *0,000188* | *0,000034* | *0,000027* | 18,53 | 0,0062 | -0,95 | 0,3324 | -2,07 | 0,0416 |
| *fucked-up* | *0,000454* | *0,000311* | *0,000061* | *0,000054* | 16,75 | 0,0184 | 0,23 | 0,6957 | -2,99 | 0,0012 |
| *phenomena* | *0,000564* | *0,000082* | *0,000080* | *0,000013* | 15,70 | 0,0367 | -4,13 | 0,0000 | 1,78 | 0,0748 |
| *iraq* | *0,000502* | *0,000186* | *0,000142* | *0,000054* | 18,92 | 0,0050 | 1,36 | 0,0262 | -4,08 | 0,0000 |
| *collision* | *0,000385* | *0,000203* | *0,000058* | *0,000028* | 15,36 | 0,0460 | -3,00 | 0,0029 | 0,38 | 0,6933 |
| *motherfucker* | *0,000443* | *0,000156* | *0,000095* | *0,000060* | 25,36 | 0,0002 | 0,50 | 0,4114 | -3,87 | 0,0000 |
| *warehouses* | *0,000379* | *0,000167* | *0,000033* | *0,000023* | 16,36 | 0,0237 | -1,94 | 0,0368 | -0,90 | 0,3626 |
| *fart* | *0,000341* | *0,000203* | *0,000031* | *0,000030* | 15,91 | 0,0318 | -0,90 | 0,1965 | -1,89 | 0,0253 |
| *cartridge* | *0,000321* | *0,000083* | *0,000047* | *0,000019* | 18,17 | 0,0077 | -2,83 | 0,0053 | -0,09 | 0,9227 |
| *fucker* | *0,000379* | *0,000273* | *0,000057* | *0,000079* | 20,38 | 0,0022 | -0,80 | 0,1728 | -2,36 | 0,0092 |
| *reproachfully* | *0,000233* | *0,000333* | *0,000023* | *0,000045* | 15,80 | 0,0342 | 5,36 | 0,0000 | -3,89 | 0,0001 |
| *housework* | *0,000214* | *0,000573* | *0,000026* | *0,000067* | 21,95 | 0,0010 | 3,42 | 0,0002 | -0,26 | 0,7847 |
| *manufactured* | *0,000353* | *0,000186* | *0,000025* | *0,000023* | 16,36 | 0,0237 | -2,47 | 0,0110 | -0,33 | 0,7427 |
| *starboard* | *0,000371* | *0,000044* | *0,000065* | *0,000011* | 15,58 | 0,0398 | -3,97 | 0,0001 | 1,58 | 0,1091 |
| *cartridges* | *0,000302* | *0,000100* | *0,000034* | *0,000025* | 15,33 | 0,0471 | -2,83 | 0,0073 | 0,19 | 0,8583 |
| *holster* | *0,000363* | *0,000125* | *0,000039* | *0,000027* | 18,03 | 0,0084 | -1,49 | 0,0803 | -1,50 | 0,0865 |
| *diameter* | *0,000387* | *0,000119* | *0,000051* | *0,000023* | 16,20 | 0,0262 | -3,50 | 0,0002 | 0,91 | 0,3334 |
| *crossly* | *0,000163* | *0,000562* | *0,000022* | *0,000100* | 18,44 | 0,0066 | 5,24 | 0,0000 | -3,11 | 0,0013 |
| *porn* | *0,000312* | *0,000252* | *0,000037* | *0,000048* | 16,37 | 0,0235 | 0,73 | 0,2004 | -3,38 | 0,0007 |
| *gandhi* | *0,000349* | *0,000196* | *0,000095* | *0,000056* | 15,98 | 0,0303 | 0,26 | 0,7315 | -2,96 | 0,0011 |
| *pissing* | *0,000302* | *0,000165* | *0,000030* | *0,000024* | 23,49 | 0,0005 | -0,08 | 0,9141 | -3,24 | 0,0010 |
| *high-heeled* | *0,000186* | *0,000374* | *0,000021* | *0,000036* | 20,53 | 0,0020 | 4,56 | 0,0000 | -1,82 | 0,0409 |
| *testicles* | *0,000254* | *0,000130* | *0,000027* | *0,000023* | 19,00 | 0,0047 | -0,91 | 0,1759 | -2,14 | 0,0110 |
| *manned* | *0,000248* | *0,000088* | *0,000024* | *0,000015* | 21,85 | 0,0010 | -3,96 | 0,0000 | 0,92 | 0,3746 |
| *masonry* | *0,000293* | *0,000072* | *0,000040* | *0,000013* | 15,42 | 0,0443 | -2,76 | 0,0125 | 0,09 | 0,9197 |

Page 10

| *explosives* | *0,000270* | *0,000114* | *0,000030* | *0,000022* | 18,17 | 0,0077 | -0,02 | 1,0000 | -2,89 | 0,0008 |
| --- | --- | --- | --- | --- | --- | --- | --- | --- | --- | --- |
| *forensic* | *0,000270* | *0,000101* | *0,000041* | *0,000025* | 15,47 | 0,0429 | -0,37 | 0,6219 | -2,35 | 0,0144 |
| *mascara* | *0,000127* | *0,000421* | *0,000015* | *0,000049* | 16,40 | 0,0231 | -0,78 | 0,2438 | 3,42 | 0,0005 |
| *girlhood* | *0,000138* | *0,000252* | *0,000019* | *0,000027* | 15,90 | 0,0321 | 4,36 | 0,0001 | -2,10 | 0,0399 |
| *amplified* | *0,000248* | *0,000129* | *0,000021* | *0,000017* | 17,90 | 0,0090 | -0,54 | 0,5149 | -2,40 | 0,0141 |
| *geraniums* | *0,000169* | *0,000310* | *0,000022* | *0,000039* | 19,54 | 0,0035 | 5,82 | 0,0000 | -4,00 | 0,0000 |
| *shooter* | *0,000239* | *0,000101* | *0,000036* | *0,000035* | 15,38 | 0,0454 | -1,67 | 0,0707 | -1,09 | 0,2802 |
| *goatee* | *0,000237* | *0,000075* | *0,000025* | *0,000016* | 24,87 | 0,0002 | -1,60 | 0,0604 | -1,92 | 0,0378 |
| *velocity* | *0,000248* | *0,000071* | *0,000035* | *0,000014* | 19,69 | 0,0032 | -1,73 | 0,0809 | -1,39 | 0,1601 |
| *rusting* | *0,000234* | *0,000141* | *0,000024* | *0,000019* | 15,57 | 0,0400 | -0,35 | 0,6515 | -2,38 | 0,0105 |
| *gunner* | *0,000188* | *0,000036* | *0,000031* | *0,000016* | 17,34 | 0,0127 | -3,60 | 0,0002 | 0,91 | 0,3466 |
| *shitting* | *0,000207* | *0,000134* | *0,000022* | *0,000022* | 17,46 | 0,0118 | -0,19 | 0,7719 | -2,69 | 0,0041 |
| *chiffon* | *0,000109* | *0,000407* | *0,000023* | *0,000066* | 21,96 | 0,0010 | 3,77 | 0,0000 | -0,67 | 0,4478 |
| *tea-table* | *0,000077* | *0,000181* | *0,000012* | *0,000029* | 15,78 | 0,0348 | 5,30 | 0,0000 | -3,74 | 0,0004 |
| *deployed* | *0,000196* | *0,000100* | *0,000023* | *0,000023* | 17,11 | 0,0147 | -0,20 | 0,8229 | -2,65 | 0,0072 |
| *daffodils* | *0,000087* | *0,000339* | *0,000013* | *0,000067* | 17,72 | 0,0101 | 4,75 | 0,0000 | -2,43 | 0,0070 |
| *payroll* | *0,000170* | *0,000061* | *0,000027* | *0,000013* | 17,34 | 0,0127 | -1,29 | 0,1046 | -1,64 | 0,0646 |
| *hesitatingly* | *0,000092* | *0,000109* | *0,000016* | *0,000022* | 17,32 | 0,0129 | 5,78 | 0,0002 | -4,54 | 0,0003 |
| *fuckers* | *0,000170* | *0,000107* | *0,000023* | *0,000034* | 21,13 | 0,0015 | 0,43 | 0,4348 | -3,50 | 0,0001 |
| *converging* | *0,000168* | *0,000051* | *0,000021* | *0,000010* | 15,75 | 0,0354 | -3,05 | 0,0014 | 0,39 | 0,6403 |
| *hairpins* | *0,000093* | *0,000170* | *0,000016* | *0,000024* | 15,88 | 0,0325 | 4,75 | 0,0000 | -2,70 | 0,0025 |
| *pinafore* | *0,000057* | *0,000244* | *0,000014* | *0,000061* | 20,56 | 0,0020 | 5,34 | 0,0000 | -2,96 | 0,0040 |
| *urinal* | *0,000159* | *0,000025* | *0,000027* | *0,000008* | 30,55 | 0,0000 | -2,46 | 0,0005 | -1,43 | 0,0826 |
| *crocheted* | *0,000072* | *0,000198* | *0,000014* | *0,000030* | 19,16 | 0,0043 | 2,42 | 0,0019 | 0,63 | 0,4476 |
| *bridesmaids* | *0,000050* | *0,000237* | *0,000012* | *0,000091* | 20,63 | 0,0019 | 5,04 | 0,0000 | -2,50 | 0,0134 |
| *unkindness* | *0,000041* | *0,000132* | *0,000007* | *0,000021* | 20,88 | 0,0017 | 6,28 | 0,0000 | -4,75 | 0,0000 |
| *urinating* | *0,000118* | *0,000062* | *0,000017* | *0,000016* | 17,15 | 0,0143 | -0,32 | 0,6283 | -2,54 | 0,0010 |
| *crochet* | *0,000055* | *0,000156* | *0,000013* | *0,000031* | 19,70 | 0,0032 | 3,41 | 0,0002 | -0,44 | 0,6421 |
| *fuchsia* | *0,000059* | *0,000183* | *0,000014* | *0,000028* | 17,56 | 0,0111 | 0,30 | 0,7163 | 2,59 | 0,0121 |
| *sparkly* | *0,000023* | *0,000109* | *0,000006* | *0,000016* | 20,26 | 0,0024 | -0,66 | 0,3036 | 3,63 | 0,0001 |
