# Supplementary material for: How Male and Female Literary Authors Write About Affect Across Cultures and Over Historical Periods
Source: Affect Sci. 2023 Sep 5;4(4):770–80. doi: 10.1007/s42761-023-00219-9 (PMC10751284; doi:10.1007/s42761-023-00219-9)
Supplement: Supplementary file 2 — Supplementary file2 (DOCX 356 KB) [file 42761_2023_219_MOESM2_ESM.docx]

| **SURNAME** | **NAME** | **TITLE** | **NATIONALITY** | | **GENDER** | **PUB_YEAR** | **CONTINENT** | **DATE OF BIRTH** | **TRANSLATED** | **NOBEL** | **NEUSTADT** | **BOOKER** | **PULITZER** | **NEBULA** | **CAINE** | **FRANZ KAFKA** | **NBA** | **BBA** | **WOMEN'S PRIZE FOR FICTION** | **NEW YORK TIMES** | **THE GUARDIAN** | **THE NEW YORKER** | **BBC** | **ATLANTIC** |
| --- | --- | --- | --- | --- | --- | --- | --- | --- | --- | --- | --- | --- | --- | --- | --- | --- | --- | --- | --- | --- | --- | --- | --- | --- |
| Abdolah | Kader | The house of the mosque | | IRAN | 0 | 2005 | 4 | 1954 | 1 | 0 | 0 | 0 | 0 | 0 | 0 | 0 | 0 | 0 | 0 | 0 | 0 | 0 | 0 | 0 |
| Aboulela | Leila | Lyrics Alley | | SUDAN | 1 | 2011 | 5 | 1964 | 0 | 0 | 0 | 0 | 0 | 0 | 0 | 0 | 0 | 0 | 0 | 0 | 0 | 0 | 0 | 0 |
| Aboulela | Leila | Minaret | | SUDAN | 1 | 2005 | 5 | 1964 | 0 | 0 | 0 | 0 | 0 | 0 | 0 | 0 | 0 | 0 | 0 | 0 | 0 | 0 | 0 | 0 |
| Aboulela | Leila | The Kindness of Enemies | | SUDAN | 1 | 2015 | 5 | 1964 | 0 | 0 | 0 | 0 | 0 | 0 | 0 | 0 | 0 | 0 | 0 | 0 | 0 | 0 | 0 | 0 |
| Abulhawa | Susan | Mornings in Jenin | | PALESTINE | 1 | 2006 | 4 | 1970 | 0 | 0 | 0 | 0 | 0 | 0 | 0 | 0 | 0 | 0 | 0 | 0 | 0 | 0 | 0 | 0 |
| Abulhawa | Susan | The Blue Between Sky and Water | | PALESTINE | 1 | 2015 | 4 | 1970 | 0 | 0 | 0 | 0 | 0 | 0 | 0 | 0 | 0 | 0 | 0 | 0 | 0 | 0 | 0 | 0 |
| Achaari | Mohammed | The Arch and the Butterfly | | MOROCCO | 0 | 2010 | 5 | 1951 | 1 | 0 | 0 | 0 | 0 | 0 | 0 | 0 | 0 | 0 | 0 | 0 | 0 | 0 | 0 | 0 |
| Achebe | Chinua | No longer at ease | | NIGERIA | 0 | 1960 | 5 | 1930 | 0 | 0 | 0 | 1 | 0 | 0 | 0 | 0 | 0 | 0 | 0 | 0 | 0 | 0 | 0 | 0 |
| Achebe | Chinua | Things fall apart | | NIGERIA | 0 | 1958 | 5 | 1930 | 0 | 0 | 0 | 1 | 0 | 0 | 0 | 0 | 0 | 0 | 0 | 0 | 0 | 0 | 0 | 0 |
| Achebe | Chinua | Arrow of God | | NIGERIA | 0 | 1964 | 5 | 1930 | 0 | 0 | 0 | 1 | 0 | 0 | 0 | 0 | 0 | 0 | 0 | 0 | 0 | 0 | 0 | 0 |
| Aciman | André | Call me by your name | | USA | 0 | 2007 | 2 | 1951 | 0 | 0 | 0 | 0 | 0 | 0 | 0 | 0 | 0 | 0 | 0 | 0 | 0 | 0 | 0 | 1 |
| Aciman | André | Find me | | USA | 0 | 2019 | 2 | 1951 | 0 | 0 | 0 | 0 | 0 | 0 | 0 | 0 | 0 | 0 | 0 | 0 | 0 | 0 | 0 | 0 |
| Acker | Kathy | Blood and Guts in High School | | USA | 1 | 1984 | 2 | 1947 | 0 | 0 | 0 | 0 | 0 | 0 | 0 | 0 | 0 | 0 | 0 | 0 | 0 | 0 | 0 | 0 |
| Ackroyd | Peter | Chatterton | | UK | 0 | 1987 | 1 | 1949 | 0 | 0 | 0 | 0 | 0 | 0 | 0 | 0 | 0 | 1 | 0 | 0 | 0 | 0 | 0 | 0 |
| Adam | Claire | Golden Child | | TRINIDAD | 1 | 2019 | 3 | ? | 0 | 0 | 0 | 0 | 0 | 0 | 0 | 0 | 0 | 0 | 0 | 0 | 0 | 0 | 0 | 0 |
| Adams | Douglas | The Hitchhiker's Guide to the Galaxy | | UK | 0 | 1979 | 1 | 1952 | 0 | 0 | 0 | 0 | 0 | 0 | 0 | 0 | 0 | 0 | 0 | 0 | 0 | 0 | 1 | 0 |
| Adams | Richard | Watership Down | | UK | 0 | 1972 | 1 | 1920 | 0 | 0 | 0 | 0 | 0 | 0 | 0 | 0 | 0 | 0 | 0 | 0 | 0 | 0 | 1 | 0 |
| Adams | Richard | The plague dogs | | UK | 0 | 1977 | 1 | 1920 | 0 | 0 | 0 | 0 | 0 | 0 | 0 | 0 | 0 | 0 | 0 | 0 | 0 | 0 | 0 | 0 |
| Addonia | Sulaiman | The Consequences of Love | | ERITREA | 0 | 2008 | 5 | 1974 | 0 | 0 | 0 | 0 | 0 | 0 | 0 | 0 | 0 | 0 | 0 | 0 | 0 | 0 | 0 | 0 |
| Adébáyò | Ayòbámi | Stay with me | | NIGERIA | 1 | 2017 | 5 | 1988 | 0 | 0 | 0 | 0 | 0 | 0 | 0 | 0 | 0 | 0 | 0 | 0 | 0 | 0 | 0 | 0 |
| Adichie Ngozi | Chimamanda | Purple hibiscus | | NIGERIA | 1 | 2003 | 5 | 1977 | 0 | 0 | 0 | 0 | 0 | 0 | 0 | 0 | 0 | 0 | 0 | 0 | 0 | 0 | 0 | 0 |
| Adichie Ngozi | Chimamanda | The thing around your neck | | NIGERIA | 1 | 2009 | 5 | 1977 | 0 | 0 | 0 | 0 | 0 | 0 | 0 | 0 | 0 | 0 | 0 | 0 | 0 | 0 | 0 | 0 |
| Adichie Ngozi | Chimamanda | Half of a yellow sun | | NIGERIA | 1 | 2006 | 5 | 1977 | 0 | 0 | 0 | 0 | 0 | 0 | 0 | 0 | 0 | 0 | 1 | 0 | 0 | 0 | 0 | 1 |
| Adichie Ngozi | Chimamanda | Americanah | | NIGERIA | 1 | 2013 | 5 | 1977 | 0 | 0 | 0 | 0 | 0 | 0 | 0 | 0 | 0 | 0 | 0 | 1 | 1 | 0 | 1 | 1 |
| Adiga | Aravind | The white tiger | | INDIA | 0 | 2008 | 4 | 1974 | 0 | 0 | 0 | 1 | 0 | 0 | 0 | 0 | 0 | 1 | 0 | 0 | 0 | 0 | 0 | 0 |
| Adiga | Aravind | Last man in tower | | INDIA | 0 | 2011 | 4 | 1974 | 0 | 0 | 0 | 0 | 0 | 0 | 0 | 0 | 0 | 1 | 0 | 0 | 0 | 0 | 0 | 0 |
| Agassi | Andre | Open | | USA | 0 | 2009 | 2 | 1970 | 0 | 0 | 0 | 0 | 0 | 0 | 0 | 0 | 0 | 0 | 0 | 0 | 0 | 0 | 0 | 0 |
| Agee | James | A Death in the Family | | USA | 0 | 1957 | 2 | 1909 | 0 | 0 | 0 | 0 | 1 | 0 | 0 | 0 | 0 | 0 | 0 | 0 | 0 | 0 | 0 | 0 |
| Agnon | Shmuel Yosef | A simple story | | ISRAEL | 0 | 1935 | 4 | 1888 | 1 | 1 | 0 | 0 | 0 | 0 | 0 | 0 | 0 | 0 | 0 | 0 | 0 | 0 | 0 | 0 |
| Agualusa | Josè Eduardo | A general theory of oblivion | | ANGOLA | 0 | 2012 | 5 | 1960 | 1 | 0 | 0 | 0 | 0 | 0 | 0 | 0 | 0 | 0 | 0 | 0 | 0 | 0 | 0 | 0 |
| Agualusa | Josè Eduardo | The Book of Chameleons | | ANGOLA | 0 | 2015 | 5 | 1960 | 1 | 0 | 0 | 0 | 0 | 0 | 0 | 0 | 0 | 0 | 0 | 0 | 0 | 0 | 0 | 0 |
| Ahmad | Jamil | The Wandering Falcon | | PAKISTAN | 0 | 2011 | 4 | 1931 | 0 | 0 | 0 | 0 | 0 | 0 | 0 | 0 | 0 | 0 | 0 | 0 | 0 | 0 | 0 | 1 |
| Aira | César Episode in the Life of a Landscape Pain | | | ARGENTINA | 0 | 2000 | 3 | 1949 | 1 | 0 | 0 | 0 | 0 | 0 | 0 | 0 | 0 | 0 | 0 | 0 | 0 | 0 | 0 | 0 |
| Aira | César Birthday | | | ARGENTINA | 0 | 2000 | 3 | 1949 | 1 | 0 | 0 | 0 | 0 | 0 | 0 | 0 | 0 | 0 | 0 | 0 | 0 | 0 | 0 | 0 |
| Aira | César Dinner | | | ARGENTINA | 0 | 2006 | 3 | 1949 | 1 | 0 | 0 | 0 | 0 | 0 | 0 | 0 | 0 | 0 | 0 | 0 | 0 | 0 | 0 | 0 |
| Akhtar | Ayad | Homeland Elegies | | USA | 0 | 2020 | 2 | 1970 | 0 | 0 | 0 | 0 | 0 | 0 | 0 | 0 | 0 | 0 | 0 | 1 | 0 | 1 | 0 | 0 |
| Akunin | Boris | The Death of Achilles | | RUSSIA | 0 | 1998 | 1 | 1956 | 1 | 0 | 0 | 0 | 0 | 0 | 0 | 0 | 0 | 0 | 0 | 0 | 0 | 0 | 0 | 0 |
| Akunin | Boris | The winter queen | | RUSSIA | 0 | 1998 | 1 | 1956 | 1 | 0 | 0 | 0 | 0 | 0 | 0 | 0 | 0 | 0 | 0 | 0 | 0 | 0 | 0 | 0 |
| Akutagawa | Ryunosuke | Rashomon | | JAPAN | 0 | 1915 | 4 | 1892 | 1 | 0 | 0 | 0 | 0 | 0 | 0 | 0 | 0 | 0 | 0 | 0 | 0 | 0 | 0 | 0 |
| al-Sa'dawi | Nawal | Memoirs of a woman doctor | | EGYPT | 1 | 1958 | 5 | 1931 | 1 | 0 | 0 | 0 | 0 | 0 | 0 | 0 | 0 | 0 | 0 | 0 | 0 | 0 | 0 | 0 |
| al-Sa'dawi | Nawal | Woman at Point Zero | | EGYPT | 1 | 1975 | 5 | 1931 | 1 | 0 | 0 | 0 | 0 | 0 | 0 | 0 | 0 | 0 | 0 | 0 | 0 | 0 | 0 | 0 |
| al-Sa'dawi | Nawal | The Fall of the Imam | | EGYPT | 1 | 1987 | 5 | 1931 | 1 | 0 | 0 | 0 | 0 | 0 | 0 | 0 | 0 | 0 | 0 | 0 | 0 | 0 | 0 | 0 |
| al-Shaykh | Hanan | Beirut Blues | | LEBANON | 1 | 1992 | 4 | 1945 | 1 | 0 | 0 | 0 | 0 | 0 | 0 | 0 | 0 | 0 | 0 | 0 | 0 | 0 | 0 | 0 |
| al-Shaykh | Hanan | I Sweep the Sun off Rooftops | | LEBANON | 1 | 1994 | 4 | 1945 | 1 | 0 | 0 | 0 | 0 | 0 | 0 | 0 | 0 | 0 | 0 | 0 | 0 | 0 | 0 | 0 |
| al-Shaykh | Hanan | Women of Sand and Myrrh | | LEBANON | 1 | 1989 | 4 | 1945 | 1 | 0 | 0 | 0 | 0 | 0 | 0 | 0 | 0 | 0 | 0 | 0 | 0 | 0 | 0 | 0 |
| Alarcón | Daniel | At night we walk in circles | | PERU | 0 | 2013 | 3 | 1977 | 0 | 0 | 0 | 0 | 0 | 0 | 0 | 0 | 0 | 0 | 0 | 0 | 0 | 0 | 0 | 0 |
| Alarcón | Daniel | Lost city radio | | PERU | 0 | 2007 | 3 | 1977 | 0 | 0 | 0 | 0 | 0 | 0 | 0 | 0 | 0 | 0 | 0 | 0 | 0 | 0 | 0 | 0 |
| Alcott | Louisa May The complete little women | | | USA | 1 | 1868 | 2 | 1832 | 0 | 0 | 0 | 0 | 0 | 0 | 0 | 0 | 0 | 0 | 0 | 0 | 0 | 0 | 1 | 0 |
| Alcott | Louisa May An Old Fashioned Girl | | | USA | 1 | 1870 | 2 | 1832 | 0 | 0 | 0 | 0 | 0 | 0 | 0 | 0 | 0 | 0 | 0 | 0 | 0 | 0 | 0 | 0 |
| Alcott | Louisa May The Mysterious Key and What It Opene | | | USA | 1 | 1867 | 2 | 1832 | 0 | 0 | 0 | 0 | 0 | 0 | 0 | 0 | 0 | 0 | 0 | 0 | 0 | 0 | 0 | 0 |
| Alcott | Louisa May Under the Lilacs | | | USA | 1 | 1878 | 2 | 1832 | 0 | 0 | 0 | 0 | 0 | 0 | 0 | 0 | 0 | 0 | 0 | 0 | 0 | 0 | 0 | 0 |
| Alcott | Louisa May Work: A Story of Experience | | | USA | 1 | 1873 | 2 | 1832 | 0 | 0 | 0 | 0 | 0 | 0 | 0 | 0 | 0 | 0 | 0 | 0 | 0 | 0 | 0 | 0 |
| Alderman | Naomi | The power | | UK | 1 | 2016 | 1 | 1974 | 0 | 0 | 0 | 0 | 0 | 0 | 0 | 0 | 0 | 0 | 1 | 1 | 1 | 0 | 0 | 0 |
| Aleksievič | Svetlana | Secondhand time | | BELARUS | 1 | 2013 | 1 | 1948 | 1 | 1 | 0 | 0 | 0 | 0 | 0 | 0 | 0 | 0 | 0 | 0 | 0 | 0 | 0 | 0 |
| Alem | Raja'a | The dove's necklace | | SAUDI ARABIA | 1 | 2012 | 4 | 1970 | 1 | 0 | 0 | 0 | 0 | 0 | 0 | 0 | 0 | 0 | 0 | 0 | 0 | 0 | 0 | 0 |
| Ali | Sabahattin | Madonna in a fur coat | | TURKEY | 0 | 1943 | 4 | 1907 | 1 | 0 | 0 | 0 | 0 | 0 | 0 | 0 | 0 | 0 | 0 | 0 | 0 | 0 | 0 | 0 |
| Ali | Monica | Brick Lane | | BANGLADESH | 1 | 2003 | 4 | 1967 | 0 | 0 | 0 | 0 | 0 | 0 | 0 | 0 | 0 | 1 | 0 | 0 | 0 | 0 | 0 | 0 |
| Ali | Monica | In the kitchen | | BANGLADESH | 1 | 2009 | 4 | 1967 | 0 | 0 | 0 | 0 | 0 | 0 | 0 | 0 | 0 | 0 | 0 | 0 | 0 | 0 | 0 | 0 |
| Allende | Isabel | The House of the Spirits | | CHILE | 1 | 1982 | 3 | 1942 | 1 | 0 | 0 | 0 | 0 | 0 | 0 | 0 | 0 | 0 | 0 | 0 | 0 | 0 | 0 | 0 |
| Allende | Isabel | A long petal of the sea | | CHILE | 1 | 2019 | 3 | 1942 | 1 | 0 | 0 | 0 | 0 | 0 | 0 | 0 | 0 | 0 | 0 | 0 | 0 | 0 | 1 | 0 |
| Allende | Isabel | City of the beasts | | CHILE | 1 | 2002 | 3 | 1942 | 1 | 0 | 0 | 0 | 0 | 0 | 0 | 0 | 0 | 0 | 0 | 0 | 0 | 0 | 0 | 0 |
| Allende | Isabel | Daughter of fortune | | CHILE | 1 | 1999 | 3 | 1942 | 1 | 0 | 0 | 0 | 0 | 0 | 0 | 0 | 0 | 0 | 0 | 0 | 0 | 0 | 0 | 0 |
| Allende | Isabel | Forest of the Pygmies | | CHILE | 1 | 2004 | 3 | 1942 | 1 | 0 | 0 | 0 | 0 | 0 | 0 | 0 | 0 | 0 | 0 | 0 | 0 | 0 | 0 | 0 |
| Allende | Isabel | Eva Luna | | CHILE | 1 | 1987 | 3 | 1942 | 1 | 0 | 0 | 0 | 0 | 0 | 0 | 0 | 0 | 0 | 0 | 0 | 0 | 0 | 0 | 0 |
| Allende | Isabel | Island Beneath the Sea | | CHILE | 1 | 2009 | 3 | 1942 | 1 | 0 | 0 | 0 | 0 | 0 | 0 | 0 | 0 | 0 | 0 | 0 | 0 | 0 | 0 | 0 |
| Allende | Isabel | Inés of My Soul | | CHILE | 1 | 2006 | 3 | 1942 | 1 | 0 | 0 | 0 | 0 | 0 | 0 | 0 | 0 | 0 | 0 | 0 | 0 | 0 | 0 | 0 |
| Alsanea | Rajaa | Girls of Riyadh | | SAUDI ARABIA | 1 | 2005 | 4 | 1981 | 0 | 0 | 0 | 0 | 0 | 0 | 0 | 0 | 0 | 0 | 0 | 0 | 0 | 0 | 0 | 0 |
| Altan | Ahmet | Endgame | | TURKEY | 0 | 2015 | 4 | 1950 | 1 | 0 | 0 | 0 | 0 | 0 | 0 | 0 | 0 | 0 | 0 | 0 | 0 | 0 | 0 | 0 |
| Altan | Ahmet | Like a Sword Wound | | TURKEY | 0 | 1998 | 4 | 1950 | 1 | 0 | 0 | 0 | 0 | 0 | 0 | 0 | 0 | 0 | 0 | 0 | 0 | 0 | 0 | 0 |
| Alvarez | Julia | In the Time of the Butterflies | | DOMINICAN REP. | 1 | 1994 | 3 | 1950 | 0 | 0 | 0 | 0 | 0 | 0 | 0 | 0 | 0 | 0 | 0 | 0 | 0 | 0 | 0 | 0 |
| Amado | Jorge | Gabriela clove and cinnamon | | BRAZIL | 0 | 1958 | 3 | 1912 | 1 | 0 | 0 | 0 | 0 | 0 | 0 | 0 | 0 | 0 | 0 | 0 | 0 | 0 | 0 | 0 |
| Amado | Jorge | Captains of the sands | | BRAZIL | 0 | 1937 | 3 | 1912 | 1 | 0 | 0 | 0 | 0 | 0 | 0 | 0 | 0 | 0 | 0 | 0 | 0 | 0 | 0 | 0 |
| Amado | Jorge | Dona Flor and her two husbands | | BRAZIL | 0 | 1966 | 3 | 1912 | 1 | 0 | 0 | 0 | 0 | 0 | 0 | 0 | 0 | 0 | 0 | 0 | 0 | 0 | 0 | 0 |
| Amis | Kingsley | The Alteration | | UK | 0 | 1976 | 1 | 1922 | 0 | 0 | 0 | 0 | 0 | 0 | 0 | 0 | 0 | 0 | 0 | 0 | 0 | 0 | 0 | 0 |
| Amis | Kingsley | The Old Devils | | UK | 0 | 1986 | 1 | 1922 | 0 | 0 | 0 | 1 | 0 | 0 | 0 | 0 | 0 | 0 | 0 | 0 | 0 | 0 | 0 | 0 |
| Amis | Martin | Time's Arrow | | UK | 0 | 1991 | 1 | 1949 | 0 | 0 | 0 | 0 | 0 | 0 | 0 | 0 | 0 | 1 | 0 | 0 | 0 | 0 | 0 | 0 |
| Ammaniti | Niccolò | Anna | | ITALY | 0 | 2015 | 1 | 1966 | 1 | 0 | 0 | 0 | 0 | 0 | 0 | 0 | 0 | 0 | 0 | 0 | 0 | 0 | 0 | 0 |
| Ammaniti | Niccolò | I'm not scared | | ITALY | 0 | 2001 | 1 | 1966 | 1 | 0 | 0 | 0 | 0 | 0 | 0 | 0 | 0 | 0 | 0 | 0 | 0 | 0 | 0 | 0 |
| Anand | Mulk Râj | Untouchable | | INDIA | 0 | 1935 | 4 | 1905 | 0 | 0 | 0 | 0 | 0 | 0 | 0 | 0 | 0 | 0 | 0 | 0 | 0 | 0 | 0 | 0 |
| Anappara | Deepa | Djinn Patrol on the Purple Line | | INDIA | 1 | 2020 | 4 | ? | 0 | 0 | 0 | 0 | 0 | 0 | 0 | 0 | 0 | 0 | 0 | 0 | 0 | 0 | 0 | 0 |
| Anderson | Sherwood | Winesburg, Ohio | | USA | 0 | 1919 | 2 | 1876 | 0 | 0 | 0 | 0 | 0 | 0 | 0 | 0 | 0 | 0 | 0 | 0 | 0 | 0 | 0 | 0 |
| Anderson | Poul | The High Crusade | | USA | 0 | 1960 | 2 | 1926 | 0 | 0 | 0 | 0 | 0 | 0 | 0 | 0 | 0 | 0 | 0 | 0 | 0 | 0 | 0 | 0 |
| Andrews | VC | Flowers in the Attic | | USA | 1 | 1979 | 2 | 1923 | 0 | 0 | 0 | 0 | 0 | 0 | 0 | 0 | 0 | 0 | 0 | 0 | 0 | 0 | 0 | 0 |
| Andrews | VC | Petals In the Wind | | USA | 1 | 1980 | 2 | 1923 | 0 | 0 | 0 | 0 | 0 | 0 | 0 | 0 | 0 | 0 | 0 | 0 | 0 | 0 | 0 | 0 |
| Andrić | Ivo | The Bridge on the Drina | | BOSNIA | 0 | 1945 | 1 | 1892 | 1 | 1 | 0 | 0 | 0 | 0 | 0 | 0 | 0 | 0 | 0 | 0 | 0 | 0 | 0 | 0 |
| Angelou | Maya | I Know Why the Caged Bird Sings | | USA | 1 | 1970 | 2 | 1928 | 0 | 0 | 0 | 0 | 0 | 0 | 0 | 0 | 0 | 0 | 0 | 0 | 0 | 0 | 0 | 0 |
| Angelou | Maya | A Song Flung Up to Heaven | | USA | 1 | 2002 | 2 | 1928 | 0 | 0 | 0 | 0 | 0 | 0 | 0 | 0 | 0 | 0 | 0 | 0 | 0 | 0 | 0 | 0 |

| **SURNAME** | | **NAME** | **TITLE** | | **NATIONALITY** | | **GENDER** | **PUB_YEAR** | **CONTINENT** | **DATE OF BIRTH** | **TRANSLATED** | **NOBEL** | **NEUSTADT** | **BOOKER** | **PULITZER** | **NEBULA** | **CAINE** | **FRANZ KAFKA** | **NBA** | **BBA** | **WOMEN'S PRIZE FOR FICTION** | **NEW YORK TIMES** | **THE GUARDIAN** | **THE NEW YORKER** | **BBC** | **ATLANTIC** |
| --- | --- | --- | --- | --- | --- | --- | --- | --- | --- | --- | --- | --- | --- | --- | --- | --- | --- | --- | --- | --- | --- | --- | --- | --- | --- | --- |
| Angelou | Maya | | | Gather Together in My Name | | USA | 1 | 1974 | 2 | 1928 | 0 | 0 | 0 | 0 | 0 | 0 | 0 | 0 | 0 | 0 | 0 | 0 | 0 | 0 | 0 | 0 |
| Angelou | Maya | | | The Heart of a Woman | | USA | 1 | 1981 | 2 | 1928 | 0 | 0 | 0 | 0 | 0 | 0 | 0 | 0 | 0 | 0 | 0 | 0 | 0 | 0 | 0 | 0 |
| Ansay | A. Manette | | | Vinegar Hill | | USA | 1 | 1994 | 2 | 1964 | 0 | 0 | 0 | 0 | 0 | 0 | 0 | 0 | 0 | 0 | 0 | 0 | 0 | 0 | 0 | 0 |
| Antunes | António Lobo | | | The splendor of Portugal | | PORTUGAL | 0 | 1997 | 1 | 1942 | 1 | 0 | 0 | 0 | 0 | 0 | 0 | 0 | 0 | 0 | 0 | 0 | 0 | 0 | 0 | 0 |
| Anwar | Arif | | | The storm | | BANGLADESH | 0 | 2018 | 4 | 1970 | 0 | 0 | 0 | 0 | 0 | 0 | 0 | 0 | 0 | 0 | 0 | 0 | 0 | 0 | 0 | 0 |
| Anyi | Wang | | | The Song of Everlasting Sorrow | | CHINA | 1 | 1995 | 4 | 1954 | 1 | 0 | 0 | 0 | 0 | 0 | 0 | 0 | 0 | 0 | 0 | 0 | 0 | 0 | 0 | 0 |
| Appelfeld | Aharon | | | The story of a life | | ISRAEL | 0 | 1999 | 4 | 1932 | 1 | 0 | 0 | 0 | 0 | 0 | 0 | 0 | 0 | 0 | 0 | 0 | 0 | 0 | 0 | 0 |
| Appelfeld | Aharon | | | Tzili | | ISRAEL | 0 | 1982 | 4 | 1932 | 1 | 0 | 0 | 0 | 0 | 0 | 0 | 0 | 0 | 0 | 0 | 0 | 0 | 0 | 0 | 0 |
| Appelfeld | Aharon | | | Blooms of darkness | | ISRAEL | 0 | 2006 | 4 | 1932 | 1 | 0 | 0 | 0 | 0 | 0 | 0 | 0 | 0 | 0 | 0 | 0 | 0 | 0 | 0 | 0 |
| Aramburu | Fernando | | | Homeland | | SPAIN | 0 | 2016 | 1 | 1959 | 1 | 0 | 0 | 0 | 0 | 0 | 0 | 0 | 0 | 0 | 0 | 0 | 0 | 0 | 0 | 0 |
| Archer | Jeffrey | | | Kane and Abel | | UK | 0 | 1979 | 1 | 1940 | 0 | 0 | 0 | 0 | 0 | 0 | 0 | 0 | 0 | 0 | 0 | 0 | 0 | 0 | 1 | 0 |
| Archer | Jeffrey | | | The Eleventh Commandment | | UK | 0 | 1998 | 1 | 1940 | 0 | 0 | 0 | 0 | 0 | 0 | 0 | 0 | 0 | 0 | 0 | 0 | 0 | 0 | 0 | 0 |
| Arenas | Reinaldo | | | Mona and Other Tales | | CUBA | 0 | 2001 | 3 | 1943 | 1 | 0 | 0 | 0 | 0 | 0 | 0 | 0 | 0 | 0 | 0 | 0 | 0 | 0 | 0 | 0 |
| Arenas | Reinaldo | | | The Color of Summer | | CUBA | 0 | 1982 | 3 | 1943 | 1 | 0 | 0 | 0 | 0 | 0 | 0 | 0 | 0 | 0 | 0 | 0 | 0 | 0 | 0 | 0 |
| Arguedas | José María | | | Deep rivers | | PERU | 0 | 1958 | 3 | 1911 | 1 | 0 | 0 | 0 | 0 | 0 | 0 | 0 | 0 | 0 | 0 | 0 | 0 | 0 | 0 | 0 |
| Arlt | Roberto | | | The seven madmen | | ARGENTINA | 0 | 1929 | 3 | 1900 | 1 | 0 | 0 | 0 | 0 | 0 | 0 | 0 | 0 | 0 | 0 | 0 | 0 | 0 | 0 | 0 |
| Arthurs | Alexia | | | How to Love a Jamaican | | JAMAICA | 1 | 2018 | 3 | 1990 | 0 | 0 | 0 | 0 | 0 | 0 | 0 | 0 | 0 | 0 | 0 | 0 | 0 | 0 | 0 | 0 |
| Atkinson | Kate | | | Life after life | | UK | 1 | 2013 | 1 | 1951 | 0 | 0 | 0 | 0 | 0 | 0 | 0 | 0 | 0 | 1 | 0 | 1 | 1 | 0 | 0 | 0 |
| Atogun | Odafe | | | Taduno's song | | NIGERIA | 0 | 2016 | 5 | ? | 0 | 0 | 0 | 0 | 0 | 0 | 0 | 0 | 0 | 0 | 0 | 0 | 0 | 0 | 0 | 0 |
| Atwood | Margaret | | | The handmaid's tale | | CANADA | 1 | 1985 | 2 | 1939 | 0 | 0 | 0 | 0 | 0 | 0 | 0 | 1 | 0 | 0 | 0 | 0 | 0 | 0 | 0 | 0 |
| Atwood | Margaret | | | Alias Grace | | CANADA | 1 | 1996 | 2 | 1939 | 0 | 0 | 0 | 0 | 0 | 0 | 0 | 1 | 0 | 0 | 0 | 0 | 0 | 0 | 0 | 0 |
| Atwood | Margaret | | | The Penelopiad | | CANADA | 1 | 2005 | 2 | 1939 | 0 | 0 | 0 | 0 | 0 | 0 | 0 | 1 | 0 | 0 | 0 | 0 | 0 | 0 | 0 | 0 |
| Atwood | Margaret | | | The testaments | | CANADA | 1 | 2019 | 2 | 1939 | 0 | 0 | 0 | 1 | 0 | 0 | 0 | 1 | 0 | 0 | 0 | 0 | 1 | 0 | 1 | 0 |
| Atwood | Margaret | | | The Blind Assassin | | CANADA | 1 | 2000 | 2 | 1939 | 0 | 0 | 0 | 1 | 0 | 0 | 0 | 1 | 0 | 0 | 0 | 0 | 0 | 0 | 0 | 0 |
| Austen | Jane | | | Pride and Prejudice | | UK | 1 | 1813 | 1 | 1775 | 0 | 0 | 0 | 0 | 0 | 0 | 0 | 0 | 0 | 0 | 0 | 0 | 0 | 1 | 1 | 0 |
| Austen | Jane | | | Emma | | UK | 1 | 1815 | 1 | 1775 | 0 | 0 | 0 | 0 | 0 | 0 | 0 | 0 | 0 | 0 | 0 | 0 | 0 | 0 | 1 | 0 |
| Austen | Jane | | | Lady Susan | | UK | 1 | 1794 | 1 | 1775 | 0 | 0 | 0 | 0 | 0 | 0 | 0 | 0 | 0 | 0 | 0 | 0 | 0 | 0 | 0 | 0 |
| Austen | Jane | | | Mansfield Park | | UK | 1 | 1814 | 1 | 1775 | 0 | 0 | 0 | 0 | 0 | 0 | 0 | 0 | 0 | 0 | 0 | 0 | 0 | 1 | 0 | 0 |
| Austen | Jane | | | Northanger Abbey | | UK | 1 | 1817 | 1 | 1775 | 0 | 0 | 0 | 0 | 0 | 0 | 0 | 0 | 0 | 0 | 0 | 0 | 0 | 0 | 0 | 0 |
| Austen | Jane | | | Persuasion | | UK | 1 | 1817 | 1 | 1775 | 0 | 0 | 0 | 0 | 0 | 0 | 0 | 0 | 0 | 0 | 0 | 0 | 0 | 1 | 1 | 0 |
| Austen | Jane | | | Sense and Sensibility | | UK | 1 | 1811 | 1 | 1775 | 0 | 0 | 0 | 0 | 0 | 0 | 0 | 0 | 0 | 0 | 0 | 0 | 1 | 0 | 0 | 0 |
| Auster | Paul | | | The New York trilogy | | USA | 0 | 1985 | 2 | 1947 | 0 | 0 | 0 | 0 | 0 | 0 | 0 | 0 | 0 | 0 | 0 | 0 | 0 | 0 | 0 | 0 |
| Auster | Paul | | | Man in the dark | | USA | 0 | 2008 | 2 | 1947 | 0 | 0 | 0 | 0 | 0 | 0 | 0 | 0 | 0 | 0 | 0 | 0 | 0 | 0 | 0 | 0 |
| Auster | Paul | | | Invisible | | USA | 0 | 2009 | 2 | 1947 | 0 | 0 | 0 | 0 | 0 | 0 | 0 | 0 | 0 | 0 | 0 | 0 | 0 | 0 | 0 | 0 |
| Auster | Paul | | | Mr Vertigo | | USA | 0 | 1994 | 2 | 1947 | 0 | 0 | 0 | 0 | 0 | 0 | 0 | 0 | 0 | 0 | 0 | 0 | 0 | 0 | 0 | 0 |
| Avery | Ellis | | | The Last Nude | | USA | 1 | 2012 | 2 | 1972 | 0 | 0 | 0 | 0 | 0 | 0 | 0 | 0 | 0 | 0 | 0 | 0 | 0 | 0 | 0 | 0 |
| Aw | Tash | | | Five Star Billionaire | | MALAYSIA | 0 | 2013 | 4 | 1971 | 0 | 0 | 0 | 0 | 0 | 0 | 0 | 0 | 0 | 0 | 0 | 0 | 0 | 0 | 0 | 0 |
| Aw | Tash | | | We, The Survivors | | MALAYSIA | 0 | 2019 | 4 | 1971 | 0 | 0 | 0 | 0 | 0 | 0 | 0 | 0 | 0 | 0 | 0 | 0 | 0 | 0 | 0 | 0 |
| Ayatsuji | Yukito | | | The Decagon House Murders | | JAPAN | 0 | 1987 | 4 | 1960 | 1 | 0 | 0 | 0 | 0 | 0 | 0 | 0 | 0 | 0 | 0 | 0 | 0 | 0 | 0 | 0 |
| Aziz Abdel | Basma | | | The queue | | EGYPT | 1 | 2013 | 5 | 1976 | 1 | 0 | 0 | 0 | 0 | 0 | 0 | 0 | 0 | 0 | 0 | 0 | 0 | 0 | 0 | 0 |
| Azuela | Mariano | | | The underdogs | | MEXICO | 0 | 1915 | 3 | 1873 | 1 | 0 | 0 | 0 | 0 | 0 | 0 | 0 | 0 | 0 | 0 | 0 | 0 | 0 | 0 | 0 |
| Azzopardi | Trezza | | | The Hiding Place | | UK | 1 | 2000 | 1 | 1961 | 0 | 0 | 0 | 0 | 0 | 0 | 0 | 0 | 0 | 0 | 0 | 0 | 0 | 0 | 0 | 0 |
| Bach | Richard | | | Jonathan Livingston Seagull | | USA | 0 | 1970 | 2 | 1936 | 0 | 0 | 0 | 0 | 0 | 0 | 0 | 0 | 0 | 0 | 0 | 0 | 0 | 0 | 0 | 0 |
| Bach | Richard | | | The Bridge Across Forever | | USA | 0 | 1984 | 2 | 1936 | 0 | 0 | 0 | 0 | 0 | 0 | 0 | 0 | 0 | 0 | 0 | 0 | 0 | 0 | 0 | 0 |
| Badani | Sejal | | | The storyteller's secret | | INDIA | 1 | 2018 | 4 | ? | 0 | 0 | 0 | 0 | 0 | 0 | 0 | 0 | 0 | 0 | 0 | 0 | 0 | 0 | 0 | 0 |
| Badani | Sejal | | | Trail of broken wings | | INDIA | 1 | 2015 | 4 | ? | 0 | 0 | 0 | 0 | 0 | 0 | 0 | 0 | 0 | 0 | 0 | 0 | 0 | 0 | 0 | 0 |
| Bader | Ali | | | Papa Sartre | | IRAQ | 0 | 2001 | 4 | 1964 | 1 | 0 | 0 | 0 | 0 | 0 | 0 | 0 | 0 | 0 | 0 | 0 | 0 | 0 | 0 | 0 |
| Bader | Ali | | | The Tobacco Keeper | | IRAQ | 0 | 2008 | 4 | 1964 | 1 | 0 | 0 | 0 | 0 | 0 | 0 | 0 | 0 | 0 | 0 | 0 | 0 | 0 | 0 | 0 |
| Bainbridge | Beryl | | | The Dressmaker | | UK | 1 | 1973 | 1 | 1932 | 0 | 0 | 0 | 0 | 0 | 0 | 0 | 0 | 0 | 1 | 0 | 0 | 0 | 0 | 0 | 0 |
| Baldwin | James | | | Another Country | | USA | 0 | 1962 | 2 | 1924 | 0 | 0 | 0 | 0 | 0 | 0 | 0 | 0 | 0 | 0 | 0 | 0 | 0 | 0 | 0 | 0 |
| Baldwin | James | | | Go Tell It on the Mountain | | USA | 0 | 1953 | 2 | 1924 | 0 | 0 | 0 | 0 | 0 | 0 | 0 | 0 | 0 | 0 | 0 | 0 | 0 | 0 | 0 | 0 |
| Baldwin | James | | | Giovanni's Room | | USA | 0 | 1956 | 2 | 1924 | 0 | 0 | 0 | 0 | 0 | 0 | 0 | 0 | 0 | 0 | 0 | 0 | 0 | 0 | 0 | 0 |
| Ballard | JG | | | High rise | | UK | 0 | 1975 | 1 | 1930 | 0 | 0 | 0 | 0 | 0 | 0 | 0 | 0 | 0 | 0 | 0 | 0 | 0 | 0 | 0 | 0 |
| Ballard | JG | | | Crash | | UK | 0 | 1973 | 1 | 1930 | 0 | 0 | 0 | 0 | 0 | 0 | 0 | 0 | 0 | 0 | 0 | 0 | 0 | 0 | 0 | 0 |
| Bangqing | Han | | | The Sing-song Girls of Shanghai | | CHINA | 0 | 1892 | 4 | 1856 | 1 | 0 | 0 | 0 | 0 | 0 | 0 | 0 | 0 | 0 | 0 | 0 | 0 | 0 | 0 | 0 |
| Banville | John | | | The Sea | | IRELAND | 0 | 2005 | 1 | 1945 | 0 | 0 | 0 | 1 | 0 | 0 | 0 | 1 | 0 | 0 | 0 | 0 | 0 | 0 | 0 | 0 |
| Banville | John | | | The Book of Evidence | | IRELAND | 0 | 1989 | 1 | 1945 | 0 | 0 | 0 | 0 | 0 | 0 | 0 | 1 | 0 | 0 | 0 | 0 | 0 | 0 | 0 | 0 |
| Barakat | Ibtisam | | | Tasting the Sky | | PALESTINE | 1 | 2007 | 4 | 1963 | 0 | 0 | 0 | 0 | 0 | 0 | 0 | 0 | 0 | 0 | 0 | 0 | 0 | 0 | 0 | 0 |
| Barakat | Ibtisam | | | Balcony on the Moon | | PALESTINE | 1 | 2016 | 4 | 1963 | 0 | 0 | 0 | 0 | 0 | 0 | 0 | 0 | 0 | 0 | 0 | 0 | 0 | 0 | 0 | 0 |
| Barakat | Najwa | | | Oh, Salaam! | | LEBANON | 1 | 2014 | 4 | 1966 | 1 | 0 | 0 | 0 | 0 | 0 | 0 | 0 | 0 | 0 | 0 | 0 | 0 | 0 | 0 | 0 |
| Barbery | Muriel | | | The elegance of the hedgehog | | FRANCE | 1 | 2006 | 1 | 1969 | 1 | 0 | 0 | 0 | 0 | 0 | 0 | 0 | 0 | 0 | 0 | 0 | 0 | 0 | 0 | 0 |
| Barclay | Florence L. | | | The Rosary | | UK | 1 | 1909 | 1 | 1862 | 0 | 0 | 0 | 0 | 0 | 0 | 0 | 0 | 0 | 0 | 0 | 0 | 0 | 0 | 0 | 0 |
| Baricco | Alessandro | | | Silk | | ITALY | 0 | 1996 | 1 | 1958 | 1 | 0 | 0 | 0 | 0 | 0 | 0 | 0 | 0 | 0 | 0 | 0 | 0 | 0 | 0 | 0 |
| Barker | Pat | | | The Silence of the Girls | | UK | 1 | 2018 | 1 | 1943 | 0 | 0 | 0 | 0 | 0 | 0 | 0 | 0 | 0 | 0 | 0 | 0 | 0 | 0 | 0 | 0 |
| Barker | Pat | | | The Ghost Road | | UK | 1 | 1995 | 1 | 1943 | 0 | 0 | 0 | 1 | 0 | 0 | 0 | 0 | 0 | 0 | 0 | 0 | 0 | 0 | 0 | 0 |
| Barker | AL | | | John Brown's Body | | UK | 1 | 1970 | 1 | 1918 | 0 | 0 | 0 | 0 | 0 | 0 | 0 | 0 | 0 | 0 | 0 | 0 | 0 | 0 | 0 | 0 |
| Barker | Nicola | | | Darkmans | | UK | 1 | 2007 | 1 | 1966 | 0 | 0 | 0 | 0 | 0 | 0 | 0 | 0 | 0 | 0 | 0 | 0 | 0 | 0 | 0 | 0 |
| Barnes | Julian | | | Levels of life | | UK | 0 | 2013 | 1 | 1946 | 0 | 0 | 0 | 0 | 0 | 0 | 0 | 0 | 0 | 0 | 0 | 0 | 0 | 0 | 0 | 0 |
| Barnes | Julian | | | The noise of time | | UK | 0 | 2016 | 1 | 1946 | 0 | 0 | 0 | 0 | 0 | 0 | 0 | 0 | 0 | 0 | 0 | 0 | 1 | 0 | 0 | 0 |
| Barnes | Julian | | | The Sense of an Ending | | UK | 0 | 2011 | 1 | 1946 | 0 | 0 | 0 | 1 | 0 | 0 | 0 | 0 | 0 | 0 | 0 | 0 | 0 | 0 | 0 | 0 |
| Barnes | Julian | | | Love, etc | | UK | 0 | 2000 | 1 | 1946 | 0 | 0 | 0 | 0 | 0 | 0 | 0 | 0 | 0 | 0 | 0 | 0 | 0 | 0 | 0 | 0 |
| Barnes | Djuna | | | Nightwood | | USA | 1 | 1936 | 2 | 1892 | 0 | 0 | 0 | 0 | 0 | 0 | 0 | 0 | 0 | 0 | 0 | 0 | 0 | 0 | 0 | 0 |
| Barnes | Margaret Ayer | | | Years of Grace | | USA | 1 | 1930 | 2 | 1886 | 0 | 0 | 0 | 0 | 1 | 0 | 0 | 0 | 0 | 0 | 0 | 0 | 0 | 0 | 0 | 0 |
| Barrett | Igoni A | | | Blackass | | NIGERIA | 0 | 2015 | 5 | 1979 | 0 | 0 | 0 | 0 | 0 | 0 | 0 | 0 | 0 | 0 | 0 | 0 | 0 | 0 | 0 | 0 |
| Barrett | Colin | | | Young skins | | IRELAND | 0 | 2015 | 1 | ? | 0 | 0 | 0 | 0 | 0 | 0 | 0 | 0 | 0 | 0 | 0 | 0 | 1 | 0 | 0 | 0 |
| Barrie | James Matthew | | | Peter and Wendy | | UK | 0 | 1911 | 1 | 1860 | 0 | 0 | 0 | 0 | 0 | 0 | 0 | 0 | 0 | 0 | 0 | 0 | 0 | 0 | 0 | 0 |
| Barry | Kevin | | | Night boat to tangier | | IRELAND | 0 | 2019 | 1 | 1969 | 0 | 0 | 0 | 0 | 0 | 0 | 0 | 0 | 0 | 0 | 0 | 1 | 0 | 0 | 0 | 1 |
| Barry | Sebastian | | | Days Without End | | IRELAND | 0 | 2017 | 1 | 1955 | 0 | 0 | 0 | 0 | 0 | 0 | 0 | 0 | 0 | 0 | 0 | 0 | 1 | 0 | 0 | 0 |
| Bassani | Giorgio | | | The garden of the Finzi-Continis | | ITALY | 0 | 1962 | 1 | 1916 | 0 | 0 | 0 | 0 | 0 | 0 | 0 | 0 | 0 | 0 | 0 | 0 | 0 | 0 | 0 | 0 |
| Bastos | Augusto Roa | | | I the Supreme | | PARAGUAY | 0 | 1974 | 3 | 1917 | 1 | 0 | 0 | 0 | 0 | 0 | 0 | 0 | 0 | 0 | 0 | 0 | 0 | 0 | 0 | 0 |
| Bastos | Augusto Roa | | | The Prosecutor | | PARAGUAY | 0 | 1993 | 3 | 1917 | 1 | 0 | 0 | 0 | 0 | 0 | 0 | 0 | 0 | 0 | 0 | 0 | 0 | 0 | 0 | 0 |
| Batalha | Martha | | | The invisible life of Eurídice Gusmão | | BRAZIL | 1 | 2016 | 3 | 1973 | 1 | 0 | 0 | 0 | 0 | 0 | 0 | 0 | 0 | 0 | 0 | 0 | 0 | 0 | 0 | 0 |
| Baum | L Frank | | | The wonderful wizard of Oz | | USA | 0 | 1900 | 2 | 1856 | 0 | 0 | 0 | 0 | 0 | 0 | 0 | 0 | 0 | 0 | 0 | 0 | 0 | 0 | 0 | 0 |
| Baxter | Charles | | | There’s Something I Want You to Do | | USA | 0 | 2015 | 2 | 1947 | 0 | 0 | 0 | 0 | 0 | 0 | 0 | 0 | 0 | 0 | 0 | 0 | 0 | 0 | 0 | 0 |
| Beah | Ishmael | | | long way gone memoirs of a boy soldie | | SIERRA LEONE | 0 | 2007 | 5 | 1980 | 0 | 0 | 0 | 0 | 0 | 0 | 0 | 0 | 0 | 0 | 0 | 0 | 0 | 0 | 0 | 0 |
| Beatty | Paul | | | The Sellout | | USA | 0 | 2015 | 2 | 1962 | 0 | 0 | 0 | 1 | 0 | 0 | 0 | 0 | 0 | 0 | 0 | 1 | 1 | 1 | 0 | 1 |
| Beatty | Paul | | | Tuff | | USA | 0 | 2000 | 2 | 1962 | 0 | 0 | 0 | 0 | 0 | 0 | 0 | 0 | 0 | 0 | 0 | 0 | 0 | 0 | 0 | 0 |

| **SURNAME** | | **NAME** | | **TITLE** | | **NATIONALITY** | | **GENDER** | **PUB_YEAR** | **CONTINENT** | **DATE OF BIRTH** | **TRANSLATED** | **NOBEL** | **NEUSTADT** | **BOOKER** | **PULITZER** | **NEBULA** | **CAINE** | **FRANZ KAFKA** | **NBA** | **BBA** | **WOMEN'S PRIZE FOR FICTION** | **NEW YORK TIMES** | **THE GUARDIAN** | **THE NEW YORKER** | **BBC** | **ATLANTIC** |
| --- | --- | --- | --- | --- | --- | --- | --- | --- | --- | --- | --- | --- | --- | --- | --- | --- | --- | --- | --- | --- | --- | --- | --- | --- | --- | --- | --- |
| Beauman | | Ned | Boxer, Beetle | | | | UK | 0 | 2010 | 1 | 1985 | 0 | 0 | 0 | 0 | 0 | 0 | 0 | 0 | 0 | 0 | 0 | 0 | 1 | 0 | 0 | 0 |
| Beckett | | Samuel | Malone dies | | | | IRELAND | 0 | 1951 | 1 | 1906 | 1 | 1 | 0 | 0 | 0 | 0 | 0 | 0 | 0 | 0 | 0 | 0 | 0 | 0 | 0 | 0 |
| Beckett | | Samuel | Murphy | | | | IRELAND | 0 | 1938 | 1 | 1906 | 1 | 1 | 0 | 0 | 0 | 0 | 0 | 0 | 0 | 0 | 0 | 0 | 0 | 0 | 0 | 0 |
| Bedford | | Sybille | Jigsaw | | | | GERMANY | 1 | 1989 | 1 | 1911 | 0 | 0 | 0 | 0 | 0 | 0 | 0 | 0 | 0 | 0 | 0 | 0 | 0 | 0 | 0 | 0 |
| Belli | | Gioconda | The scroll of seduction | | | | NICARAGUA | 1 | 2005 | 3 | 1948 | 0 | 0 | 0 | 0 | 0 | 0 | 0 | 0 | 0 | 0 | 0 | 0 | 0 | 0 | 0 | 0 |
| Belli | | Gioconda | Infinity in the Palm of Her Hand | | | | NICARAGUA | 1 | 2008 | 3 | 1948 | 0 | 0 | 0 | 0 | 0 | 0 | 0 | 0 | 0 | 0 | 0 | 0 | 0 | 0 | 0 | 0 |
| Bellow | | Saul | Henderson the rain king | | | | USA | 0 | 1959 | 2 | 1915 | 0 | 1 | 0 | 0 | 0 | 0 | 0 | 0 | 0 | 0 | 0 | 0 | 0 | 0 | 0 | 0 |
| Bellow | | Saul | Humboldt's gift | | | | USA | 0 | 1975 | 2 | 1915 | 0 | 1 | 0 | 0 | 1 | 0 | 0 | 0 | 0 | 0 | 0 | 0 | 0 | 0 | 0 | 0 |
| Benchley | | Peter | Jaws | | | | USA | 0 | 1974 | 2 | 1940 | 0 | 0 | 0 | 0 | 0 | 0 | 0 | 0 | 0 | 0 | 0 | 0 | 0 | 0 | 0 | 0 |
| Bender | | Aimee | The particular sadness of lemon cake | | | | USA | 1 | 2010 | 2 | 1969 | 0 | 0 | 0 | 0 | 0 | 0 | 0 | 0 | 0 | 0 | 0 | 0 | 0 | 0 | 0 | 0 |
| Benedetti | | Mario | The Truce: The Diary of Martin Santome | | | | URUGUAY | 0 | 1960 | 3 | 1920 | 1 | 0 | 0 | 0 | 0 | 0 | 0 | 0 | 0 | 0 | 0 | 0 | 0 | 0 | 0 | 0 |
| Benioff | | David | City of thieves | | | | USA | 0 | 2008 | 2 | 1970 | 0 | 0 | 0 | 0 | 0 | 0 | 0 | 0 | 0 | 0 | 0 | 0 | 0 | 0 | 0 | 0 |
| Benmalek | | Anouar | Abduction | | | | MOROCCO | 0 | 2011 | 5 | 1956 | 1 | 0 | 0 | 0 | 0 | 0 | 0 | 0 | 0 | 0 | 0 | 0 | 0 | 0 | 0 | 0 |
| Bennett | | Alan | The lady in the van | | | | UK | 0 | 1989 | 1 | 1934 | 0 | 0 | 0 | 0 | 0 | 0 | 0 | 0 | 0 | 1 | 0 | 0 | 0 | 0 | 0 | 0 |
| Bennett | | Andrea | Galina Petrovna's three-legged dog stor | | | | CANADA | 1 | 2015 | 2 | 1969 | 0 | 0 | 0 | 0 | 0 | 0 | 0 | 0 | 0 | 0 | 0 | 0 | 0 | 0 | 0 | 0 |
| Bennett | | Arnold | The Old Wives’ tale | | | | UK | 0 | 1908 | 1 | 1867 | 0 | 0 | 0 | 0 | 0 | 0 | 0 | 0 | 0 | 0 | 0 | 0 | 0 | 0 | 0 | 0 |
| Berberova | Nina Nikolaevna | | | | The Italics are mine |  | RUSSIA | 1 | 1969 | 1 | 1901 | 1 | 0 | 0 | 0 | 0 | 0 | 0 | 0 | 0 | 0 | 0 | 0 | 0 | 0 | 0 | 0 |
| Berg | | Elizabeth | Open House | | | | USA | 1 | 2000 | 2 | 1948 | 0 | 0 | 0 | 0 | 0 | 0 | 0 | 0 | 0 | 0 | 0 | 0 | 0 | 0 | 0 | 0 |
| Berger | | John | G. | | | | UK | 0 | 1972 | 1 | 1926 | 0 | 0 | 0 | 1 | 0 | 0 | 0 | 0 | 0 | 0 | 0 | 0 | 0 | 0 | 0 | 0 |
| Bernhard | | Thomas | The loser | | | | AUSTRIA | 0 | 1983 | 1 | 1931 | 1 | 0 | 0 | 0 | 0 | 0 | 0 | 0 | 0 | 0 | 0 | 0 | 0 | 0 | 0 | 0 |
| Bibish | | Bibish | The Dancer from Khiva: A Memoir | | | | UZBEKISTAN | 1 | 2008 | 4 | ? | 1 | 0 | 0 | 0 | 0 | 0 | 0 | 0 | 0 | 0 | 0 | 0 | 0 | 0 | 0 | 0 |
| Binchy | | Maeve | The Glass Lake | | | | IRELAND | 1 | 1994 | 1 | 1939 | 0 | 0 | 0 | 0 | 0 | 0 | 0 | 0 | 0 | 1 | 0 | 0 | 0 | 0 | 0 | 0 |
| Binchy | | Maeve | Tara Road | | | | IRELAND | 1 | 1998 | 1 | 1939 | 0 | 0 | 0 | 0 | 0 | 0 | 0 | 0 | 0 | 1 | 0 | 0 | 0 | 0 | 0 | 0 |
| Binebine | | Mahi | Horses of God | | | | MOROCCO | 0 | 2010 | 5 | 1959 | 1 | 0 | 0 | 0 | 0 | 0 | 0 | 0 | 0 | 0 | 0 | 0 | 0 | 0 | 0 | 0 |
| Birch | | Carol | Jamrach's Menagerie | | | | UK | 1 | 2011 | 1 | 1951 | 0 | 0 | 0 | 0 | 0 | 0 | 0 | 0 | 0 | 0 | 0 | 0 | 0 | 0 | 0 | 0 |
| Bjørnson | | Bjørnstjerne | A happy boy | | | | NORWAY | 0 | 1860 | 1 | 1832 | 1 | 1 | 0 | 0 | 0 | 0 | 0 | 0 | 0 | 0 | 0 | 0 | 0 | 0 | 0 | 0 |
| Blackwood | | Caroline | Great Granny Webster | | | | UK | 1 | 1977 | 1 | 1931 | 0 | 0 | 0 | 0 | 0 | 0 | 0 | 0 | 0 | 0 | 0 | 0 | 0 | 0 | 0 | 0 |
| Blatty | | William | The Exorcist | | | | USA | 0 | 1971 | 2 | 1928 | 0 | 0 | 0 | 0 | 0 | 0 | 0 | 0 | 0 | 0 | 0 | 0 | 0 | 0 | 0 | 0 |
| Blixen | | Karen | Out of Africa | | | | DENMARK | 1 | 1937 | 1 | 1885 | 0 | 0 | 0 | 0 | 0 | 0 | 0 | 0 | 0 | 0 | 0 | 0 | 0 | 0 | 0 | 0 |
| Bloch | | Robert | The scarf | | | | USA | 0 | 1947 | 2 | 1917 | 0 | 0 | 0 | 0 | 0 | 0 | 0 | 0 | 0 | 0 | 0 | 0 | 0 | 0 | 0 | 0 |
| Bloch | | Robert | Psycho | | | | USA | 0 | 1959 | 2 | 1917 | 0 | 0 | 0 | 0 | 0 | 0 | 0 | 0 | 0 | 0 | 0 | 0 | 0 | 0 | 0 | 0 |
| Blyton | | Enid | The Enchanted Wood | | | | UK | 1 | 1939 | 1 | 1897 | 0 | 0 | 0 | 0 | 0 | 0 | 0 | 0 | 0 | 0 | 0 | 0 | 0 | 0 | 0 | 0 |
| Blyton | | Enid | The Land of Far-Beyond | | | | UK | 1 | 1942 | 1 | 1897 | 0 | 0 | 0 | 0 | 0 | 0 | 0 | 0 | 0 | 0 | 0 | 0 | 0 | 0 | 0 | 0 |
| Blyton | | Enid | The Mistery of Banshee Towers | | | | UK | 1 | 1961 | 1 | 1897 | 0 | 0 | 0 | 0 | 0 | 0 | 0 | 0 | 0 | 0 | 0 | 0 | 0 | 0 | 0 | 0 |
| Blyton | | Enid | The Mistery of the Disappearing Cat | | | | UK | 1 | 1944 | 1 | 1897 | 0 | 0 | 0 | 0 | 0 | 0 | 0 | 0 | 0 | 0 | 0 | 0 | 0 | 0 | 0 | 0 |
| Blyton | | Enid | The Mistery of the Invisible Thief | | | | UK | 1 | 1950 | 1 | 1897 | 0 | 0 | 0 | 0 | 0 | 0 | 0 | 0 | 0 | 0 | 0 | 0 | 0 | 0 | 0 | 0 |
| Blyton | | Enid | The Mistery of the Strange Bundle | | | | UK | 1 | 1952 | 1 | 1897 | 0 | 0 | 0 | 0 | 0 | 0 | 0 | 0 | 0 | 0 | 0 | 0 | 0 | 0 | 0 | 0 |
| Blyton | | Enid | The Valley of Adventure | | | | UK | 1 | 1947 | 1 | 1897 | 0 | 0 | 0 | 0 | 0 | 0 | 0 | 0 | 0 | 0 | 0 | 0 | 0 | 0 | 0 | 0 |
| Bodrožić | | Ivana | The Hotel Tito | | | | CROATIA | 1 | 2010 | 1 | 1982 | 1 | 0 | 0 | 0 | 0 | 0 | 0 | 0 | 0 | 0 | 0 | 0 | 0 | 0 | 0 | 0 |
| Bohjalian | | Chris | Midwives | | | | USA | 0 | 1997 | 2 | 1962 | 0 | 0 | 0 | 0 | 0 | 0 | 0 | 0 | 0 | 0 | 0 | 0 | 0 | 0 | 0 | 0 |
| Bolaño | | Roberto | 2666 | | | | CHILE | 0 | 2004 | 3 | 1953 | 1 | 0 | 0 | 0 | 0 | 0 | 0 | 0 | 0 | 0 | 0 | 1 | 0 | 0 | 0 | 0 |
| Bolaño | | Roberto | The Savage Detectives | | | | CHILE | 0 | 1998 | 3 | 1953 | 1 | 0 | 0 | 0 | 0 | 0 | 0 | 0 | 0 | 0 | 0 | 1 | 0 | 0 | 0 | 0 |
| Böll | | Heinrich | The clown | | | | GERMANY | 0 | 1963 | 1 | 1917 | 1 | 1 | 0 | 0 | 0 | 0 | 0 | 0 | 0 | 0 | 0 | 0 | 0 | 0 | 0 | 0 |
| Böll | | Heinrich | Group portrait with lady | | | | GERMANY | 0 | 1971 | 1 | 1917 | 1 | 1 | 0 | 0 | 0 | 0 | 0 | 0 | 0 | 0 | 0 | 0 | 0 | 0 | 0 | 0 |
| Böll | | Heinrich | Billiards at Half-Past Nine | | | | GERMANY | 0 | 1959 | 1 | 1917 | 1 | 1 | 0 | 0 | 0 | 0 | 0 | 0 | 0 | 0 | 0 | 0 | 0 | 0 | 0 | 0 |
| Booth | | Martin | The Industry of Souls | | | | UK | 0 | 1998 | 1 | 1944 | 0 | 0 | 0 | 0 | 0 | 0 | 0 | 0 | 0 | 0 | 0 | 0 | 0 | 0 | 0 | 0 |
| Borges | | Jorge Luis | Fictions | | | | ARGENTINA | 0 | 1944 | 3 | 1899 | 1 | 0 | 0 | 0 | 0 | 0 | 0 | 0 | 0 | 0 | 0 | 0 | 0 | 0 | 0 | 0 |
| Borges | | Jorge Luis | Six Problems for Don Isidro Parodi | | | | ARGENTINA | 0 | 1942 | 3 | 1899 | 1 | 0 | 0 | 0 | 0 | 0 | 0 | 0 | 0 | 0 | 0 | 0 | 0 | 0 | 0 | 0 |
| Borges | | Jorge Luis | Doctor Brodie's Report | | | | ARGENTINA | 0 | 1970 | 3 | 1899 | 1 | 0 | 0 | 0 | 0 | 0 | 0 | 0 | 0 | 0 | 0 | 0 | 0 | 0 | 0 | 0 |
| Borgo Sainz | | Karina | It would be night in Caracas | | | | VENEZUELA | 1 | 2019 | 3 | 1982 | 1 | 0 | 0 | 0 | 0 | 0 | 0 | 0 | 0 | 0 | 0 | 0 | 0 | 0 | 0 | 0 |
| Boullosa | | Carmen | Cleopatra Dismounts | | | | MEXICO | 1 | 2002 | 3 | 1954 | 1 | 0 | 0 | 0 | 0 | 0 | 0 | 0 | 0 | 0 | 0 | 0 | 0 | 0 | 0 | 0 |
| Boullosa | | Carmen | Heavens on Earth | | | | MEXICO | 1 | 1997 | 3 | 1954 | 1 | 0 | 0 | 0 | 0 | 0 | 0 | 0 | 0 | 0 | 0 | 0 | 0 | 0 | 0 | 0 |
| Bowen | | Elizabeth | Eva Trout | | | | IRELAND | 1 | 1968 | 1 | 1899 | 0 | 0 | 0 | 0 | 0 | 0 | 0 | 0 | 0 | 0 | 0 | 0 | 0 | 0 | 0 | 0 |
| Bowles | | Paul | The Sheltering Sky | | | | USA | 0 | 1949 | 2 | 1910 | 0 | 0 | 0 | 0 | 0 | 0 | 0 | 0 | 0 | 0 | 0 | 0 | 0 | 0 | 0 | 0 |
| Boyd | | William | An ice-cream war | | | | UK | 0 | 1982 | 1 | 1952 | 0 | 0 | 0 | 0 | 0 | 0 | 0 | 0 | 0 | 0 | 0 | 0 | 0 | 0 | 0 | 0 |
| Boyne | | John | The boy in the striped pajamas | | | | IRELAND | 0 | 2006 | 1 | 1971 | 0 | 0 | 0 | 0 | 0 | 0 | 0 | 0 | 0 | 0 | 0 | 0 | 0 | 0 | 0 | 0 |
| Bradbury | | Ray | Fahreneit 451 | | | | USA | 0 | 1953 | 2 | 1920 | 0 | 0 | 0 | 0 | 0 | 0 | 0 | 0 | 0 | 0 | 0 | 0 | 0 | 0 | 0 | 0 |
| Bradbury | | Malcolm | Rates of Exchange | | | | UK | 0 | 1983 | 1 | 1932 | 0 | 0 | 0 | 0 | 0 | 0 | 0 | 0 | 0 | 0 | 0 | 0 | 0 | 0 | 0 | 0 |
| Braddon | | Mary | Lady Audleys Secret | | | | UK | 1 | 1862 | 1 | 1835 | 0 | 0 | 0 | 0 | 0 | 0 | 0 | 0 | 0 | 0 | 0 | 0 | 0 | 0 | 0 | 0 |
| Braddon | | Mary | London Pride | | | | UK | 1 | 1896 | 1 | 1835 | 0 | 0 | 0 | 0 | 0 | 0 | 0 | 0 | 0 | 0 | 0 | 0 | 0 | 0 | 0 | 0 |
| Braddon | | Mary | Phantom Fortune | | | | UK | 1 | 1883 | 1 | 1835 | 0 | 0 | 0 | 0 | 0 | 0 | 0 | 0 | 0 | 0 | 0 | 0 | 0 | 0 | 0 | 0 |
| Braddon | | Mary | The Doctor's Wife | | | | UK | 1 | 1864 | 1 | 1835 | 0 | 0 | 0 | 0 | 0 | 0 | 0 | 0 | 0 | 0 | 0 | 0 | 0 | 0 | 0 | 0 |
| Braddon | | Mary | The Golden Calf | | | | UK | 1 | 1883 | 1 | 1835 | 0 | 0 | 0 | 0 | 0 | 0 | 0 | 0 | 0 | 0 | 0 | 0 | 0 | 0 | 0 | 0 |
| Braddon | | Mary | Fenton's Quest | | | | UK | 1 | 1871 | 1 | 1835 | 0 | 0 | 0 | 0 | 0 | 0 | 0 | 0 | 0 | 0 | 0 | 0 | 0 | 0 | 0 | 0 |
| Bradley | | Alan | Thrice the Brinded Cat Hath Mew'd | | | | CANADA | 0 | 2016 | 2 | 1938 | 0 | 0 | 0 | 0 | 0 | 0 | 0 | 0 | 0 | 0 | 0 | 0 | 0 | 0 | 0 | 0 |
| Bradley | | Alan | he Weed that Strings the Hangman's Ba | | | | CANADA | 0 | 2010 | 2 | 1938 | 0 | 0 | 0 | 0 | 0 | 0 | 0 | 0 | 0 | 0 | 0 | 0 | 0 | 0 | 0 | 0 |
| Bradley | | Marion | Falcons of Narabedla | | | | USA | 1 | 1957 | 2 | 1930 | 0 | 0 | 0 | 0 | 0 | 0 | 0 | 0 | 0 | 0 | 0 | 0 | 0 | 0 | 0 | 0 |
| Bradley | | Marion | The Brass Dragon | | | | USA | 1 | 1970 | 2 | 1930 | 0 | 0 | 0 | 0 | 0 | 0 | 0 | 0 | 0 | 0 | 0 | 0 | 0 | 0 | 0 | 0 |
| Bradley | | Edward | The Adventures of Mr. Verdant Green | | | | UK | 0 | 1853 | 1 | 1827 | 0 | 0 | 0 | 0 | 0 | 0 | 0 | 0 | 0 | 0 | 0 | 0 | 0 | 0 | 0 | 0 |
| Bradley | | Edward | e Further Adventures of Mr. Verdant Gre | | | | UK | 0 | 1857 | 1 | 1827 | 0 | 0 | 0 | 0 | 0 | 0 | 0 | 0 | 0 | 0 | 0 | 0 | 0 | 0 | 0 | 0 |
| Brink | | Andre | A Dry White Season | | | | SOUTH AFRICA | 0 | 1979 | 5 | 1935 | 1 | 0 | 0 | 0 | 0 | 0 | 0 | 0 | 0 | 0 | 0 | 0 | 0 | 0 | 0 | 0 |
| Brink | | Andre | An Instant in the Wind | | | | SOUTH AFRICA | 0 | 1975 | 5 | 1935 | 0 | 0 | 0 | 0 | 0 | 0 | 0 | 0 | 0 | 0 | 0 | 0 | 0 | 0 | 0 | 0 |
| Brink | | Andre | Philida | | | | SOUTH AFRICA | 0 | 2012 | 5 | 1935 | 0 | 0 | 0 | 0 | 0 | 0 | 0 | 0 | 0 | 0 | 0 | 0 | 0 | 0 | 0 | 0 |
| Bromfield | | Louis | The rains came | | | | USA | 0 | 1937 | 2 | 1896 | 0 | 0 | 0 | 0 | 0 | 0 | 0 | 0 | 0 | 0 | 0 | 0 | 0 | 0 | 0 | 0 |
| Bromfield | | Louis | Early Autumn | | | | USA | 0 | 1926 | 2 | 1896 | 0 | 0 | 0 | 0 | 1 | 0 | 0 | 0 | 0 | 0 | 0 | 0 | 0 | 0 | 0 | 0 |
| Brontë | | Charlotte | Jane Eyre | | | | UK | 1 | 1847 | 1 | 1816 | 0 | 0 | 0 | 0 | 0 | 0 | 0 | 0 | 0 | 0 | 0 | 0 | 0 | 0 | 1 | 0 |
| Brontë | | Charlotte | Villette | | | | UK | 1 | 1853 | 1 | 1816 | 0 | 0 | 0 | 0 | 0 | 0 | 0 | 0 | 0 | 0 | 0 | 0 | 0 | 0 | 0 | 0 |
| Brontë | | Charlotte | Shirley | | | | UK | 1 | 1849 | 1 | 1816 | 0 | 0 | 0 | 0 | 0 | 0 | 0 | 0 | 0 | 0 | 0 | 0 | 0 | 0 | 0 | 0 |
| Brontë | | Emily | Wuthering heights | | | | UK | 1 | 1847 | 1 | 1818 | 0 | 0 | 0 | 0 | 0 | 0 | 0 | 0 | 0 | 0 | 0 | 0 | 0 | 0 | 1 | 0 |
| Brontë | | Anne | The Tenant of Wildfell Hall | | | | UK | 1 | 1848 | 1 | 1820 | 0 | 0 | 0 | 0 | 0 | 0 | 0 | 0 | 0 | 0 | 0 | 0 | 0 | 0 | 0 | 0 |
| Brookner | | Anita | Hotel du Lac | | | | UK | 1 | 1984 | 1 | 1928 | 0 | 0 | 0 | 1 | 0 | 0 | 0 | 0 | 0 | 0 | 0 | 0 | 0 | 0 | 0 | 0 |
| Brookner | | Anita | Leaving Home | | | | UK | 1 | 2005 | 1 | 1928 | 0 | 0 | 0 | 0 | 0 | 0 | 0 | 0 | 0 | 0 | 0 | 0 | 0 | 0 | 0 | 0 |
| Brooks | | Geraldine | Year of wonders | | | | AUSTRALIA | 1 | 2001 | 6 | 1955 | 0 | 0 | 0 | 0 | 0 | 0 | 0 | 0 | 0 | 0 | 0 | 0 | 0 | 0 | 0 | 0 |
| Brooks | | Geraldine | People of the book | | | | AUSTRALIA | 1 | 2008 | 6 | 1955 | 0 | 0 | 0 | 0 | 0 | 0 | 0 | 0 | 0 | 0 | 0 | 0 | 0 | 0 | 0 | 0 |
| Brooks | | Geraldine | Caleb's crossing | | | | AUSTRALIA | 1 | 2011 | 6 | 1955 | 0 | 0 | 0 | 0 | 0 | 0 | 0 | 0 | 0 | 0 | 0 | 0 | 0 | 0 | 0 | 0 |
| Brooks | | Geraldine | March | | | | AUSTRALIA | 1 | 2005 | 6 | 1955 | 0 | 0 | 0 | 0 | 1 | 0 | 0 | 0 | 0 | 0 | 0 | 0 | 0 | 0 | 0 | 0 |
| Brooks | | Terry | The Sword of Shannara | | | | USA | 0 | 1977 | 2 | 1944 | 0 | 0 | 0 | 0 | 0 | 0 | 0 | 0 | 0 | 0 | 0 | 0 | 0 | 0 | 0 | 0 |

| **SURNAME** | **NAME** | | **TITLE** | **NATIONALITY** | **GENDER** | **PUB_YEAR** | **CONTINENT** | **DATE OF BIRTH** | **TRANSLATED** | **NOBEL** | **NEUSTADT** | **BOOKER** | **PULITZER** | **NEBULA** | **CAINE** | **FRANZ KAFKA** | **NBA** | **BBA** | **WOMEN'S PRIZE FOR FICTION** | **NEW YORK TIMES** | **THE GUARDIAN** | **THE NEW YORKER** | **BBC** | **ATLANTIC** |
| --- | --- | --- | --- | --- | --- | --- | --- | --- | --- | --- | --- | --- | --- | --- | --- | --- | --- | --- | --- | --- | --- | --- | --- | --- |
| Brown | Daniel | Angels and demons | | USA | 0 | 2000 | 2 | 1964 | 0 | 0 | 0 | 0 | 0 | 0 | 0 | 0 | 0 | 0 | 0 | 0 | 0 | 0 | 0 | 0 |
| Brown | Daniel | The Da Vinci code | | USA | 0 | 2003 | 2 | 1964 | 0 | 0 | 0 | 0 | 0 | 0 | 0 | 0 | 0 | 1 | 0 | 0 | 0 | 0 | 0 | 0 |
| Brown | Daniel | The Lost Symbol | | USA | 0 | 2009 | 2 | 1964 | 0 | 0 | 0 | 0 | 0 | 0 | 0 | 0 | 0 | 0 | 0 | 0 | 0 | 0 | 0 | 0 |
| Brown | Fredric | What Mad Universe | | USA | 0 | 1949 | 2 | 1906 | 0 | 0 | 0 | 0 | 0 | 0 | 0 | 0 | 0 | 0 | 0 | 0 | 0 | 0 | 0 | 0 |
| Brown | Fredric | The Mind Thing | | USA | 0 | 1961 | 2 | 1906 | 0 | 0 | 0 | 0 | 0 | 0 | 0 | 0 | 0 | 0 | 0 | 0 | 0 | 0 | 0 | 0 |
| Brown | Charles | thur Mervyn or Memoirs of the Year 17 | | USA | 0 | 1799 | 2 | 1771 | 0 | 0 | 0 | 0 | 0 | 0 | 0 | 0 | 0 | 0 | 0 | 0 | 0 | 0 | 0 | 0 |
| Brown | Charles | Wieland or the Transformation | | USA | 0 | 1798 | 2 | 1771 | 0 | 0 | 0 | 0 | 0 | 0 | 0 | 0 | 0 | 0 | 0 | 0 | 0 | 0 | 0 | 0 |
| Brown | Charles | dgar Huntly or Memoirs of a Sleep-Walk | | USA | 0 | 1799 | 2 | 1771 | 0 | 0 | 0 | 0 | 0 | 0 | 0 | 0 | 0 | 0 | 0 | 0 | 0 | 0 | 0 | 0 |
| Brown | Charles | Jane Talbot | | USA | 0 | 1801 | 2 | 1771 | 0 | 0 | 0 | 0 | 0 | 0 | 0 | 0 | 0 | 0 | 0 | 0 | 0 | 0 | 0 | 0 |
| Brown | William Wells | Clotel; or, The President's Daughter | | USA | 0 | 1853 | 2 | 1814 | 0 | 0 | 0 | 0 | 0 | 0 | 0 | 0 | 0 | 0 | 0 | 0 | 0 | 0 | 0 | 0 |
| Buarque | Chico | My german brother | | BRAZIL | 0 | 2018 | 3 | 1944 | 1 | 0 | 0 | 0 | 0 | 0 | 0 | 0 | 0 | 0 | 0 | 0 | 0 | 0 | 0 | 0 |
| Buarque | Chico | Spilt Milk | | BRAZIL | 0 | 2009 | 3 | 1944 | 1 | 0 | 0 | 0 | 0 | 0 | 0 | 0 | 0 | 0 | 0 | 0 | 0 | 0 | 0 | 0 |
| Buchan | John | Prester John | | UK | 0 | 1910 | 1 | 1875 | 0 | 0 | 0 | 0 | 0 | 0 | 0 | 0 | 0 | 0 | 0 | 0 | 0 | 0 | 0 | 0 |
| Buchan | John | The Thirty-Nine Steps | | UK | 0 | 1915 | 1 | 1875 | 0 | 0 | 0 | 0 | 0 | 0 | 0 | 0 | 0 | 0 | 0 | 0 | 0 | 0 | 0 | 0 |
| Buchan | John | Greenmantle | | UK | 0 | 1916 | 1 | 1875 | 0 | 0 | 0 | 0 | 0 | 0 | 0 | 0 | 0 | 0 | 0 | 0 | 0 | 0 | 0 | 0 |
| Buchan | John | Mr Standfast | | UK | 0 | 1919 | 1 | 1875 | 0 | 0 | 0 | 0 | 0 | 0 | 0 | 0 | 0 | 0 | 0 | 0 | 0 | 0 | 0 | 0 |
| Buchan | John | Huntingtower | | UK | 0 | 1922 | 1 | 1875 | 0 | 0 | 0 | 0 | 0 | 0 | 0 | 0 | 0 | 0 | 0 | 0 | 0 | 0 | 0 | 0 |
| Buck | Pearl S. | The Good Earth | | USA | 1 | 1931 | 2 | 1892 | 0 | 1 | 0 | 0 | 1 | 0 | 0 | 0 | 0 | 0 | 0 | 0 | 0 | 0 | 0 | 0 |
| Buck | Pearl S. | Dragon seed | | USA | 1 | 1942 | 2 | 1892 | 0 | 1 | 0 | 0 | 0 | 0 | 0 | 0 | 0 | 0 | 0 | 0 | 0 | 0 | 0 | 0 |
| Bukowski | Charles | Post office | | USA | 0 | 1971 | 2 | 1920 | 0 | 0 | 0 | 0 | 0 | 0 | 0 | 0 | 0 | 0 | 0 | 0 | 0 | 0 | 0 | 0 |
| Bukowski | Charles | Factotum | | USA | 0 | 1975 | 2 | 1920 | 0 | 0 | 0 | 0 | 0 | 0 | 0 | 0 | 0 | 0 | 0 | 0 | 0 | 0 | 0 | 0 |
| Bulgakov | Mikhail | The white guard | | RUSSIA | 0 | 1925 | 1 | 1891 | 1 | 0 | 0 | 0 | 0 | 0 | 0 | 0 | 0 | 0 | 0 | 0 | 0 | 0 | 0 | 0 |
| Bulgakov | Mikhail | Master and Margarita | | RUSSIA | 0 | 1966 | 1 | 1891 | 1 | 0 | 0 | 0 | 0 | 0 | 0 | 0 | 0 | 0 | 0 | 0 | 0 | 0 | 0 | 0 |
| Bunin | Ivan | The Gentleman From San Francisco | | RUSSIA | 0 | 1916 | 1 | 1870 | 1 | 1 | 0 | 0 | 0 | 0 | 0 | 0 | 0 | 0 | 0 | 0 | 0 | 0 | 0 | 0 |
| Bunker | Edward | No beast so fierce | | USA | 0 | 1973 | 2 | 1933 | 0 | 0 | 0 | 0 | 0 | 0 | 0 | 0 | 0 | 0 | 0 | 0 | 0 | 0 | 0 | 0 |
| Bunker | Edward | Dog eat dog | | USA | 0 | 1995 | 2 | 1933 | 0 | 0 | 0 | 0 | 0 | 0 | 0 | 0 | 0 | 0 | 0 | 0 | 0 | 0 | 0 | 0 |
| Burgess | Anthony | Earthly Powers | | UK | 0 | 1980 | 1 | 1917 | 0 | 0 | 0 | 0 | 0 | 0 | 0 | 0 | 0 | 0 | 0 | 0 | 0 | 0 | 0 | 0 |
| Burnet Macrae | Graeme | The Accident on the A35 | | UK | 0 | 2017 | 1 | 1967 | 0 | 0 | 0 | 0 | 0 | 0 | 0 | 0 | 0 | 0 | 0 | 0 | 0 | 0 | 0 | 0 |
| Burney | Frances | he History of A Young Lady's Entrance i | | UK | 1 | 1778 | 1 | 1752 | 0 | 0 | 0 | 0 | 0 | 0 | 0 | 0 | 0 | 0 | 0 | 0 | 0 | 0 | 0 | 0 |
| Burney | Frances | Camilla: Or, A Picture of Youth | | UK | 1 | 1802 | 1 | 1752 | 0 | 0 | 0 | 0 | 0 | 0 | 0 | 0 | 0 | 0 | 0 | 0 | 0 | 0 | 0 | 0 |
| Burney | Frances | Cecilia: Or, Memoirs of an Heiress | | UK | 1 | 1782 | 1 | 1752 | 0 | 0 | 0 | 0 | 0 | 0 | 0 | 0 | 0 | 0 | 0 | 0 | 0 | 0 | 0 | 0 |
| Burney | Frances | The Wanderer: Or, Female Difficulties | | UK | 1 | 1814 | 1 | 1752 | 0 | 0 | 0 | 0 | 0 | 0 | 0 | 0 | 0 | 0 | 0 | 0 | 0 | 0 | 0 | 0 |
| Burns | Anna | Milkman | | UK | 1 | 2018 | 1 | 1962 | 0 | 0 | 0 | 1 | 0 | 0 | 0 | 0 | 0 | 0 | 0 | 0 | 0 | 0 | 0 | 0 |
| Burroughs | William S | Naked lunch | | USA | 0 | 1959 | 2 | 1914 | 0 | 0 | 0 | 0 | 0 | 0 | 0 | 0 | 0 | 0 | 0 | 0 | 0 | 0 | 0 | 0 |
| Burroughs | William S | Cities of the Red Night | | USA | 0 | 1981 | 2 | 1914 | 0 | 0 | 0 | 0 | 0 | 0 | 0 | 0 | 0 | 0 | 0 | 0 | 0 | 0 | 0 | 0 |
| Burroughs | William S | Junkie | | USA | 0 | 1953 | 2 | 1914 | 0 | 0 | 0 | 0 | 0 | 0 | 0 | 0 | 0 | 0 | 0 | 0 | 0 | 0 | 0 | 0 |
| Burroughs | William S | Nova Express | | USA | 0 | 1964 | 2 | 1914 | 0 | 0 | 0 | 0 | 0 | 0 | 0 | 0 | 0 | 0 | 0 | 0 | 0 | 0 | 0 | 0 |
| Burroughs | William S | The Soft Machine | | USA | 0 | 1961 | 2 | 1914 | 0 | 0 | 0 | 0 | 0 | 0 | 0 | 0 | 0 | 0 | 0 | 0 | 0 | 0 | 0 | 0 |
| Burroughs | William S | The Ticket That Exploded | | USA | 0 | 1962 | 2 | 1914 | 0 | 0 | 0 | 0 | 0 | 0 | 0 | 0 | 0 | 0 | 0 | 0 | 0 | 0 | 0 | 0 |
| Burroughs | Edgar | Tarzan of the Apes | | USA | 0 | 1914 | 2 | 1875 | 0 | 0 | 0 | 0 | 0 | 0 | 0 | 0 | 0 | 0 | 0 | 0 | 0 | 0 | 0 | 0 |
| Burroughs | Edgar | The Gods of Mars | | USA | 0 | 1913 | 2 | 1875 | 0 | 0 | 0 | 0 | 0 | 0 | 0 | 0 | 0 | 0 | 0 | 0 | 0 | 0 | 0 | 0 |
| Butler | Octavia | Kindred | | USA | 1 | 1979 | 2 | 1947 | 0 | 0 | 0 | 0 | 0 | 0 | 0 | 0 | 0 | 0 | 0 | 0 | 0 | 0 | 0 | 0 |
| Butler | Octavia | Parable of the sower | | USA | 1 | 1993 | 2 | 1947 | 0 | 0 | 0 | 0 | 0 | 0 | 0 | 0 | 0 | 0 | 0 | 0 | 0 | 0 | 0 | 0 |
| Butler | Samuel | Erewhon | | UK | 0 | 1872 | 1 | 1835 | 0 | 0 | 0 | 0 | 0 | 0 | 0 | 0 | 0 | 0 | 0 | 0 | 0 | 0 | 0 | 0 |
| Butler | Robert | A Good Scent from a Strange Mountain | | USA | 0 | 1992 | 2 | 1945 | 0 | 0 | 0 | 0 | 1 | 0 | 0 | 0 | 0 | 0 | 0 | 0 | 0 | 0 | 0 | 0 |
| Buzzati | Dino | The Tartar Steppe | | ITALY | 0 | 1940 | 1 | 1906 | 1 | 0 | 0 | 0 | 0 | 0 | 0 | 0 | 0 | 0 | 0 | 0 | 0 | 0 | 0 | 0 |
| Byatt | Antonia Susan | The children's book | | UK | 1 | 2009 | 1 | 1936 | 0 | 0 | 0 | 0 | 0 | 0 | 0 | 0 | 0 | 0 | 0 | 0 | 0 | 0 | 0 | 0 |
| Byatt | Antonia Susan | Possession | | UK | 1 | 1990 | 1 | 1936 | 0 | 0 | 0 | 1 | 0 | 0 | 0 | 0 | 0 | 0 | 0 | 0 | 0 | 0 | 0 | 0 |
| Cain | James | The postman always rings twice | | USA | 0 | 1934 | 2 | 1892 | 0 | 0 | 0 | 0 | 0 | 0 | 0 | 0 | 0 | 0 | 0 | 0 | 0 | 0 | 0 | 0 |
| Caldwell | Erskine | Tobacco road | | USA | 0 | 1932 | 2 | 1903 | 0 | 0 | 0 | 0 | 0 | 0 | 0 | 0 | 0 | 0 | 0 | 0 | 0 | 0 | 0 | 0 |
| Caldwell | Erskine | God's Little Acre | | USA | 0 | 1933 | 2 | 1903 | 0 | 0 | 0 | 0 | 0 | 0 | 0 | 0 | 0 | 0 | 0 | 0 | 0 | 0 | 0 | 0 |
| Caldwell | Erskine | Journeyman | | USA | 0 | 1935 | 2 | 1903 | 0 | 0 | 0 | 0 | 0 | 0 | 0 | 0 | 0 | 0 | 0 | 0 | 0 | 0 | 0 | 0 |
| Calvino | Italo | Palomar | | ITALY | 0 | 1983 | 1 | 1923 | 1 | 0 | 0 | 0 | 0 | 0 | 0 | 0 | 0 | 0 | 0 | 0 | 0 | 0 | 0 | 0 |
| Calvino | Italo | Cosmicomics | | ITALY | 0 | 1965 | 1 | 1923 | 1 | 0 | 0 | 0 | 0 | 0 | 0 | 0 | 0 | 0 | 0 | 0 | 0 | 0 | 0 | 0 |
| Calvino | Italo | Difficult loves | | ITALY | 0 | 1970 | 1 | 1923 | 1 | 0 | 0 | 0 | 0 | 0 | 0 | 0 | 0 | 0 | 0 | 0 | 0 | 0 | 0 | 0 |
| Calvino | Italo | If on a winter's night a traveler | | ITALY | 0 | 1979 | 1 | 1923 | 1 | 0 | 0 | 0 | 0 | 0 | 0 | 0 | 0 | 0 | 0 | 0 | 0 | 0 | 0 | 0 |
| Calvino | Italo | Invisible cities | | ITALY | 0 | 1972 | 1 | 1923 | 1 | 0 | 0 | 0 | 0 | 0 | 0 | 0 | 0 | 0 | 0 | 0 | 0 | 0 | 0 | 0 |
| Calvino | Italo | The nonexistent knight | | ITALY | 0 | 1959 | 1 | 1923 | 1 | 0 | 0 | 0 | 0 | 0 | 0 | 0 | 0 | 0 | 0 | 0 | 0 | 0 | 0 | 0 |
| Cameron | Peter | Someday this pain will be useful to you | | USA | 0 | 2007 | 2 | 1959 | 0 | 0 | 0 | 0 | 0 | 0 | 0 | 0 | 0 | 0 | 0 | 0 | 0 | 0 | 0 | 0 |
| Campbell | John | Who Goes There? | | USA | 0 | 1938 | 2 | 1910 | 0 | 0 | 0 | 0 | 0 | 0 | 0 | 0 | 0 | 0 | 0 | 0 | 0 | 0 | 0 | 0 |
| Campbell | John | The Incredible Planet | | USA | 0 | 1949 | 2 | 1910 | 0 | 0 | 0 | 0 | 0 | 0 | 0 | 0 | 0 | 0 | 0 | 0 | 0 | 0 | 0 | 0 |
| Camus | Albert | The first man | | FRANCE | 0 | 1994 | 1 | 1913 | 1 | 1 | 0 | 0 | 0 | 0 | 0 | 0 | 0 | 0 | 0 | 0 | 0 | 0 | 0 | 0 |
| Camus | Albert | The plague | | FRANCE | 0 | 1947 | 1 | 1913 | 1 | 1 | 0 | 0 | 0 | 0 | 0 | 0 | 0 | 0 | 0 | 0 | 1 | 0 | 0 | 0 |
| Camus | Albert | The Stranger | | FRANCE | 0 | 1942 | 1 | 1913 | 1 | 1 | 0 | 0 | 0 | 0 | 0 | 0 | 0 | 0 | 0 | 0 | 0 | 0 | 0 | 0 |
| Canetti | Elias | Auto da fe | | BULGARIA | 0 | 1935 | 1 | 1905 | 1 | 1 | 0 | 0 | 0 | 0 | 0 | 0 | 0 | 0 | 0 | 0 | 0 | 0 | 0 | 0 |
| Capote | Truman | Breakfast at Tiffany's | | USA | 0 | 1958 | 2 | 1924 | 0 | 0 | 0 | 0 | 0 | 0 | 0 | 0 | 0 | 0 | 0 | 0 | 0 | 0 | 0 | 0 |
| Capote | Truman | In cold blood | | USA | 0 | 1965 | 2 | 1924 | 0 | 0 | 0 | 0 | 0 | 0 | 0 | 0 | 0 | 0 | 0 | 0 | 0 | 0 | 0 | 1 |
| Capote | Truman | Other Voices, Other Rooms | | USA | 0 | 1948 | 2 | 1924 | 0 | 0 | 0 | 0 | 0 | 0 | 0 | 0 | 0 | 0 | 0 | 0 | 0 | 0 | 0 | 0 |
| Capote | Truman | Summer Crossing | | USA | 0 | 1943 | 2 | 1924 | 0 | 0 | 0 | 0 | 0 | 0 | 0 | 0 | 0 | 0 | 0 | 0 | 0 | 0 | 0 | 0 |
| Carey | Peter | Parrot and Olivier in America | | AUSTRALIA | 0 | 2009 | 6 | 1943 | 0 | 0 | 0 | 0 | 0 | 0 | 0 | 0 | 0 | 0 | 0 | 0 | 0 | 0 | 0 | 0 |
| Carey | Peter | A long way from home | | AUSTRALIA | 0 | 2018 | 6 | 1943 | 0 | 0 | 0 | 0 | 0 | 0 | 0 | 0 | 0 | 0 | 0 | 0 | 0 | 0 | 0 | 0 |
| Carey | Peter | True History of the Kelly Gang | | AUSTRALIA | 0 | 2000 | 6 | 1943 | 0 | 0 | 0 | 1 | 0 | 0 | 0 | 0 | 0 | 0 | 0 | 0 | 0 | 0 | 0 | 0 |
| Carey | Peter | Oscar and Lucinda | | AUSTRALIA | 0 | 1998 | 6 | 1943 | 0 | 0 | 0 | 1 | 0 | 0 | 0 | 0 | 0 | 0 | 0 | 0 | 0 | 0 | 0 | 0 |
| Carpentier | Alejo | War of time | | CUBA | 0 | 1955 | 3 | 1904 | 1 | 0 | 0 | 0 | 0 | 0 | 0 | 0 | 0 | 0 | 0 | 0 | 0 | 0 | 0 | 0 |
| Carpentier | Alejo | Kingdom of This World | | CUBA | 0 | 1949 | 3 | 1904 | 1 | 0 | 0 | 0 | 0 | 0 | 0 | 0 | 0 | 0 | 0 | 0 | 0 | 0 | 0 | 0 |
| Carpentier | Alejo | The Lost Steps | | CUBA | 0 | 1953 | 3 | 1904 | 1 | 0 | 0 | 0 | 0 | 0 | 0 | 0 | 0 | 0 | 0 | 0 | 0 | 0 | 0 | 0 |
| Carr | JL | A Month in the Country | | UK | 0 | 1980 | 1 | 1912 | 0 | 0 | 0 | 0 | 0 | 0 | 0 | 0 | 0 | 0 | 0 | 0 | 0 | 0 | 0 | 0 |
| Carrère | Emmanuel | Limonov | | FRANCE | 0 | 2011 | 1 | 1957 | 1 | 0 | 0 | 0 | 0 | 0 | 0 | 0 | 0 | 0 | 0 | 0 | 0 | 0 | 0 | 0 |
| Carrisi | Donato | The lost girls of Rome | | ITALY | 0 | 2011 | 1 | 1973 | 1 | 0 | 0 | 0 | 0 | 0 | 0 | 0 | 0 | 0 | 0 | 0 | 0 | 0 | 0 | 0 |
| Carrisi | Donato | The whisperer | | ITALY | 0 | 2009 | 1 | 1973 | 1 | 0 | 0 | 0 | 0 | 0 | 0 | 0 | 0 | 0 | 0 | 0 | 0 | 0 | 0 | 0 |
| Carrisi | Donato | The vanished ones | | ITALY | 0 | 2013 | 1 | 1973 | 1 | 0 | 0 | 0 | 0 | 0 | 0 | 0 | 0 | 0 | 0 | 0 | 0 | 0 | 0 | 0 |
| Carrisi | Donato | The hunter of the dark | | ITALY | 0 | 2014 | 1 | 1973 | 1 | 0 | 0 | 0 | 0 | 0 | 0 | 0 | 0 | 0 | 0 | 0 | 0 | 0 | 0 | 0 |
| Carroll | Lewis | Alice in wonderland | | UK | 0 | 1865 | 1 | 1832 | 0 | 0 | 0 | 0 | 0 | 0 | 0 | 0 | 0 | 0 | 0 | 0 | 0 | 0 | 1 | 0 |
| Carroll | Lewis | Sylvie and Bruno | | UK | 0 | 1889 | 1 | 1832 | 0 | 0 | 0 | 0 | 0 | 0 | 0 | 0 | 0 | 0 | 0 | 0 | 0 | 0 | 0 | 0 |
| Carroll | Lewis | Through the Looking-Glass | | UK | 0 | 1871 | 1 | 1832 | 0 | 0 | 0 | 0 | 0 | 0 | 0 | 0 | 0 | 0 | 0 | 0 | 0 | 0 | 0 | 0 |
| Carroll | Lewis | A Tangled Tale | | UK | 0 | 1880 | 1 | 1832 | 0 | 0 | 0 | 0 | 0 | 0 | 0 | 0 | 0 | 0 | 0 | 0 | 0 | 0 | 0 | 0 |
| Cărtărescu | Mircea | Blinding the left wing | | ROMANIA | 0 | 1996 | 1 | 1956 | 1 | 0 | 0 | 0 | 0 | 0 | 0 | 0 | 0 | 0 | 0 | 0 | 0 | 0 | 0 | 0 |

| **SURNAME** | | **NAME** | | **TITLE** | | **NATIONALITY** | | **GENDER** | **PUB_YEAR** | **CONTINENT** | **DATE OF BIRTH** | **TRANSLATED** | **NOBEL** | **NEUSTADT** | **BOOKER** | **PULITZER** | **NEBULA** | **CAINE** | **FRANZ KAFKA** | **NBA** | **BBA** | **WOMEN'S PRIZE FOR FICTION** | **NEW YORK TIMES** | **THE GUARDIAN** | **THE NEW YORKER** | **BBC** | **ATLANTIC** |
| --- | --- | --- | --- | --- | --- | --- | --- | --- | --- | --- | --- | --- | --- | --- | --- | --- | --- | --- | --- | --- | --- | --- | --- | --- | --- | --- | --- |
| Carter | | Angela | Fireworks | | | | UK | 1 | 1974 | 1 | 1940 | 0 | 0 | 0 | 0 | 0 | 0 | 0 | 0 | 0 | 0 | 0 | 0 | 0 | 0 | 0 | 0 |
| Cartland | | Barbara | Cupid Rides Pillion | | | | UK | 1 | 1952 | 1 | 1901 | 0 | 0 | 0 | 0 | 0 | 0 | 0 | 0 | 0 | 0 | 0 | 0 | 0 | 0 | 0 | 0 |
| Cartland | | Barbara | The Poor Governess | | | | UK | 1 | 1982 | 1 | 1901 | 0 | 0 | 0 | 0 | 0 | 0 | 0 | 0 | 0 | 0 | 0 | 0 | 0 | 0 | 0 | 0 |
| Carver | | Raymond | hat we talk about when we talk about lo | | | | USA | 0 | 1981 | 2 | 1938 | 0 | 0 | 0 | 0 | 0 | 0 | 0 | 0 | 0 | 0 | 0 | 0 | 0 | 0 | 0 | 0 |
| Carver | | Raymond | Cathedral | | | | USA | 0 | 1983 | 2 | 1938 | 0 | 0 | 0 | 0 | 0 | 0 | 0 | 0 | 0 | 0 | 0 | 0 | 0 | 0 | 0 | 0 |
| Casares | | Adolfo Bioy | The invention of Morel | | | | ARGENTINA | 0 | 1940 | 3 | 1914 | 1 | 0 | 0 | 0 | 0 | 0 | 0 | 0 | 0 | 0 | 0 | 0 | 0 | 0 | 0 | 0 |
| Castaneda | | Carlos | The teachings of Don Juan | | | | PERU | 0 | 1968 | 3 | 1925 | 0 | 0 | 0 | 0 | 0 | 0 | 0 | 0 | 0 | 0 | 0 | 0 | 0 | 0 | 0 | 0 |
| Castaneda | | Carlos | The power of silence | | | | PERU | 0 | 1987 | 3 | 1925 | 0 | 0 | 0 | 0 | 0 | 0 | 0 | 0 | 0 | 0 | 0 | 0 | 0 | 0 | 0 | 0 |
| Cather | | Willa | My mortal enemy | | | | USA | 1 | 1926 | 2 | 1873 | 0 | 0 | 0 | 0 | 0 | 0 | 0 | 0 | 0 | 0 | 0 | 0 | 0 | 0 | 0 | 0 |
| Cather | | Willa | One of Ours | | | | USA | 1 | 1922 | 2 | 1873 | 0 | 0 | 0 | 0 | 1 | 0 | 0 | 0 | 0 | 0 | 0 | 0 | 0 | 0 | 0 | 0 |
| Cather | | Willa | The Song of the Lark | | | | USA | 1 | 1915 | 2 | 1873 | 0 | 0 | 0 | 0 | 0 | 0 | 0 | 0 | 0 | 0 | 0 | 0 | 0 | 0 | 0 | 0 |
| Cather | | Willa | My Ántonia | | | | USA | 1 | 1918 | 2 | 1873 | 0 | 0 | 0 | 0 | 0 | 0 | 0 | 0 | 0 | 0 | 0 | 0 | 0 | 0 | 0 | 0 |
| Cather | | Willa | O Pioneers! | | | | USA | 1 | 1913 | 2 | 1873 | 0 | 0 | 0 | 0 | 0 | 0 | 0 | 0 | 0 | 0 | 0 | 0 | 0 | 0 | 0 | 0 |
| Catozzella | | Giuseppe | Don't tell me you are afraid | | | | ITALY | 0 | 2014 | 1 | 1976 | 1 | 0 | 0 | 0 | 0 | 0 | 0 | 0 | 0 | 0 | 0 | 0 | 0 | 0 | 0 | 0 |
| Catton | | Eleanor | The Luminaries | | | | NEW ZEALAND | 1 | 2013 | 6 | 1985 | 0 | 0 | 0 | 1 | 0 | 0 | 0 | 0 | 0 | 0 | 0 | 0 | 1 | 0 | 0 | 0 |
| Cave | | Nick | And the Ass Saw the Angel | | | | AUSTRALIA | 0 | 1989 | 6 | 1957 | 0 | 0 | 0 | 0 | 0 | 0 | 0 | 0 | 0 | 0 | 0 | 0 | 0 | 0 | 0 | 0 |
| Cela | | Camilo José | The Family of Pascual Duarte | | | | SPAIN | 0 | 1942 | 1 | 1916 | 1 | 1 | 0 | 0 | 0 | 0 | 0 | 0 | 0 | 0 | 0 | 0 | 0 | 0 | 0 | 0 |
| Celine  Celine | Louis Ferdinand  Louis Ferdinand | | | | Castle to castle  Death on Credit |  | FRANCE  FRANCE | 0  0 | 1957  1936 | 1  1 | 1894  1894 | 1  1 | 0  0 | 0  0 | 0  0 | 0  0 | 0  0 | 0  0 | 0  0 | 0  0 | 0  0 | 0  0 | 0  0 | 0  0 | 0  0 | 0  0 | 0  0 |
| Cercas | | Javier | Soldiers of Salamis | | | | SPAIN | 0 | 2001 | 1 | 1962 | 1 | 0 | 0 | 0 | 0 | 0 | 0 | 0 | 0 | 0 | 0 | 0 | 0 | 0 | 0 | 0 |
| Cezair-Thompson | | Margaret | The True History of Paradise | | | | JAMAICA | 1 | 1999 | 3 | 1956 | 0 | 0 | 0 | 0 | 0 | 0 | 0 | 0 | 0 | 0 | 0 | 0 | 0 | 0 | 0 | 0 |
| Chabon | | Michael | e amazing adventures of Kavalier and C | | | | USA | 0 | 2000 | 2 | 1963 | 0 | 0 | 0 | 0 | 1 | 0 | 0 | 0 | 0 | 0 | 0 | 0 | 0 | 0 | 0 | 0 |
| Chandler | | Raymond | The big sleep | | | | USA | 0 | 1939 | 2 | 1888 | 0 | 0 | 0 | 0 | 0 | 0 | 0 | 0 | 0 | 0 | 0 | 0 | 0 | 0 | 0 | 0 |
| Chandler | | Raymond | The long goodbye | | | | USA | 0 | 1953 | 2 | 1888 | 0 | 0 | 0 | 0 | 0 | 0 | 0 | 0 | 0 | 0 | 0 | 0 | 0 | 0 | 0 | 0 |
| Chandler | | Raymond | Farewell my lovely | | | | USA | 0 | 1940 | 2 | 1888 | 0 | 0 | 0 | 0 | 0 | 0 | 0 | 0 | 0 | 0 | 0 | 0 | 0 | 0 | 0 | 0 |
| Chandra | | Vikram | Sacred games | | | | INDIA | 0 | 2006 | 4 | 1961 | 0 | 0 | 0 | 0 | 0 | 0 | 0 | 0 | 0 | 0 | 0 | 0 | 0 | 0 | 0 | 0 |
| Chang | | Jung | Wild swans | | | | CHINA | 1 | 1991 | 4 | 1952 | 0 | 0 | 0 | 0 | 0 | 0 | 0 | 0 | 0 | 1 | 0 | 0 | 0 | 0 | 0 | 0 |
| Channer | | Colin | The Girl With the Golden Shoes | | | | JAMAICA | 0 | 2007 | 3 | 1963 | 0 | 0 | 0 | 0 | 0 | 0 | 0 | 0 | 0 | 0 | 0 | 0 | 0 | 0 | 0 | 0 |
| Charriere | | Henri | Papillon | | | | FRANCE | 0 | 1969 | 1 | 1906 | 1 | 0 | 0 | 0 | 0 | 0 | 0 | 0 | 0 | 0 | 0 | 0 | 0 | 0 | 0 | 0 |
| Charriere | | Henri | Banco | | | | FRANCE | 0 | 1972 | 1 | 1906 | 1 | 0 | 0 | 0 | 0 | 0 | 0 | 0 | 0 | 0 | 0 | 0 | 0 | 0 | 0 | 0 |
| Chatterji | Bankimchandra | | | | Anandamath |  | INDIA | 0 | 1882 | 4 | 1838 | 1 | 0 | 0 | 0 | 0 | 0 | 0 | 0 | 0 | 0 | 0 | 0 | 0 | 0 | 0 | 0 |
| Chatwin | | Bruce | What am I doing here | | | | UK | 0 | 1988 | 1 | 1940 | 0 | 0 | 0 | 0 | 0 | 0 | 0 | 0 | 0 | 0 | 0 | 0 | 0 | 0 | 0 | 0 |
| Chatwin | | Bruce | In Patagonia | | | | UK | 0 | 1977 | 1 | 1940 | 0 | 0 | 0 | 0 | 0 | 0 | 0 | 0 | 0 | 0 | 0 | 0 | 0 | 0 | 0 | 0 |
| Chatwin | | Bruce | The songlines | | | | UK | 0 | 1987 | 1 | 1940 | 0 | 0 | 0 | 0 | 0 | 0 | 0 | 0 | 0 | 0 | 0 | 0 | 0 | 0 | 0 | 0 |
| Chavarría | | Daniel | Adios Muchachos | | | | URUGUAY | 0 | 1994 | 3 | 1933 | 1 | 0 | 0 | 0 | 0 | 0 | 0 | 0 | 0 | 0 | 0 | 0 | 0 | 0 | 0 | 0 |
| Cheever | | John | Oh what a paradise it seems | | | | USA | 0 | 1982 | 2 | 1912 | 0 | 0 | 0 | 0 | 0 | 0 | 0 | 0 | 0 | 0 | 0 | 0 | 0 | 0 | 0 | 0 |
| Cheever | | John | Bullet park | | | | USA | 0 | 1969 | 2 | 1912 | 0 | 0 | 0 | 0 | 0 | 0 | 0 | 0 | 0 | 0 | 0 | 0 | 0 | 0 | 0 | 0 |
| Chikwava | | Brian | Harare North | | | | ZIMBABWE | 0 | 2009 | 5 | 1972 | 0 | 0 | 0 | 0 | 0 | 0 | 0 | 0 | 0 | 0 | 0 | 0 | 0 | 0 | 0 | 0 |
| Chiziane | | Paulina | The First Wife | | | | MOZAMBIQUE | 1 | 2002 | 5 | 1955 | 1 | 0 | 0 | 0 | 0 | 0 | 0 | 0 | 0 | 0 | 0 | 0 | 0 | 0 | 0 | 0 |
| Choden | | Kunzang | Folktales of Bhutan | | | | BHUTAN | 1 | 2009 | 4 | 1952 | 0 | 0 | 0 | 0 | 0 | 0 | 0 | 0 | 0 | 0 | 0 | 0 | 0 | 0 | 0 | 0 |
| Chopin | | Kate | At Fault | | | | USA | 1 | 1890 | 2 | 1850 | 0 | 0 | 0 | 0 | 0 | 0 | 0 | 0 | 0 | 0 | 0 | 0 | 0 | 0 | 0 | 0 |
| Chopin | | Kate | The Awakening | | | | USA | 1 | 1899 | 2 | 1850 | 0 | 0 | 0 | 0 | 0 | 0 | 0 | 0 | 0 | 0 | 0 | 0 | 0 | 0 | 0 | 0 |
| Chreiteh | | Alexandra | Always Coca-Cola | | | | LEBANON | 1 | 2009 | 4 | 1987 | 1 | 0 | 0 | 0 | 0 | 0 | 0 | 0 | 0 | 0 | 0 | 0 | 0 | 0 | 0 | 0 |
| Christie | | Agatha | And then there were none | | | | UK | 1 | 1939 | 1 | 1890 | 0 | 0 | 0 | 0 | 0 | 0 | 0 | 0 | 0 | 0 | 0 | 0 | 0 | 0 | 0 | 0 |
| Christie | | Agatha | Murder on the Orient Express | | | | UK | 1 | 1934 | 1 | 1890 | 0 | 0 | 0 | 0 | 0 | 0 | 0 | 0 | 0 | 0 | 0 | 0 | 0 | 0 | 0 | 0 |
| Christie | | Agatha | Evil under the sun | | | | UK | 1 | 1941 | 1 | 1890 | 0 | 0 | 0 | 0 | 0 | 0 | 0 | 0 | 0 | 0 | 0 | 0 | 0 | 0 | 0 | 0 |
| Christie | | Agatha | Mrs McGinty's dead | | | | UK | 1 | 1952 | 1 | 1890 | 0 | 0 | 0 | 0 | 0 | 0 | 0 | 0 | 0 | 0 | 0 | 0 | 0 | 0 | 0 | 0 |
| Christie | | Agatha | Dumb witness | | | | UK | 1 | 1937 | 1 | 1890 | 0 | 0 | 0 | 0 | 0 | 0 | 0 | 0 | 0 | 0 | 0 | 0 | 0 | 0 | 0 | 0 |
| Christie | | Agatha | Elephants can remember | | | | UK | 1 | 1972 | 1 | 1890 | 0 | 0 | 0 | 0 | 0 | 0 | 0 | 0 | 0 | 0 | 0 | 0 | 0 | 0 | 0 | 0 |
| Christie | | Agatha | The secret adversary | | | | UK | 1 | 1922 | 1 | 1890 | 0 | 0 | 0 | 0 | 0 | 0 | 0 | 0 | 0 | 0 | 0 | 0 | 0 | 0 | 0 | 0 |
| Cixin | | Liu | The three body problem | | | | CHINA | 0 | 2008 | 4 | 1963 | 1 | 0 | 0 | 0 | 0 | 0 | 0 | 0 | 0 | 0 | 0 | 0 | 0 | 0 | 0 | 1 |
| Cixin | | Liu | The dark forest | | | | CHINA | 0 | 2008 | 4 | 1963 | 1 | 0 | 0 | 0 | 0 | 0 | 0 | 0 | 0 | 0 | 0 | 0 | 0 | 0 | 0 | 0 |
| Clancy | | Tom | The hunt for red october | | | | USA | 0 | 1984 | 2 | 1947 | 0 | 0 | 0 | 0 | 0 | 0 | 0 | 0 | 0 | 0 | 0 | 0 | 0 | 0 | 0 | 0 |
| Clancy | | Tom | The cardinal of the Kremlin | | | | USA | 0 | 1988 | 2 | 1947 | 0 | 0 | 0 | 0 | 0 | 0 | 0 | 0 | 0 | 0 | 0 | 0 | 0 | 0 | 0 | 0 |
| Clark | | Georgia | The regulars | | | | AUSTRALIA | 1 | 2016 | 6 | ? | 0 | 0 | 0 | 0 | 0 | 0 | 0 | 0 | 0 | 0 | 0 | 0 | 0 | 0 | 0 | 0 |
| Clark | | Mary | A Cry In The Night | | | | USA | 1 | 1982 | 2 | 1927 | 0 | 0 | 0 | 0 | 0 | 0 | 0 | 0 | 0 | 0 | 0 | 0 | 0 | 0 | 0 | 0 |
| Clark | | Mary | A Stranger Is Watching | | | | USA | 1 | 1977 | 2 | 1927 | 0 | 0 | 0 | 0 | 0 | 0 | 0 | 0 | 0 | 0 | 0 | 0 | 0 | 0 | 0 | 0 |
| Clark | | Marcus | For the Term of His Natural Life | | | | UK | 0 | 1870 | 1 | 1846 | 0 | 0 | 0 | 0 | 0 | 0 | 0 | 0 | 0 | 0 | 0 | 0 | 0 | 0 | 0 | 0 |
| Clarke | | Arthur | Rendezvous with Rama | | | | UK | 0 | 1972 | 1 | 1917 | 0 | 0 | 0 | 0 | 0 | 1 | 0 | 0 | 0 | 0 | 0 | 0 | 0 | 0 | 0 | 0 |
| Clarke | | Arthur | Childhood's End | | | | UK | 0 | 1953 | 1 | 1917 | 0 | 0 | 0 | 0 | 0 | 0 | 0 | 0 | 0 | 0 | 0 | 0 | 0 | 0 | 0 | 0 |
| Clavell | | James | Whirlwind | | | | AUSTRALIA | 0 | 1986 | 6 | 1921 | 0 | 0 | 0 | 0 | 0 | 0 | 0 | 0 | 0 | 0 | 0 | 0 | 0 | 0 | 0 | 0 |
| Clavell | | James | Shōgun | | | | AUSTRALIA | 0 | 1975 | 6 | 1921 | 0 | 0 | 0 | 0 | 0 | 0 | 0 | 0 | 0 | 0 | 0 | 0 | 0 | 0 | 0 | 0 |
| Cleage | | Pearl | hat Looks Like Crazy on an Ordinary D | | | | USA | 1 | 1997 | 2 | 1948 | 0 | 0 | 0 | 0 | 0 | 0 | 0 | 0 | 0 | 0 | 0 | 0 | 0 | 0 | 0 | 0 |
| Cleland | | John | Memoirs Of Fanny Hill | | | | UK | 0 | 1748 | 1 | 1709 | 0 | 0 | 0 | 0 | 0 | 0 | 0 | 0 | 0 | 0 | 0 | 0 | 0 | 0 | 0 | 0 |
| Cline | | Ernest | Ready player two | | | | USA | 0 | 2020 | 2 | 1972 | 0 | 0 | 0 | 0 | 0 | 0 | 0 | 0 | 0 | 0 | 0 | 0 | 0 | 0 | 1 | 0 |
| Cobb | | Humphrey | Paths of glory | | | | CANADA | 0 | 1935 | 2 | 1899 | 0 | 0 | 0 | 0 | 0 | 0 | 0 | 0 | 0 | 0 | 0 | 0 | 0 | 0 | 0 | 0 |
| Cocteau | | Jean | The holy terrors | | | | FRANCE | 0 | 1929 | 1 | 1889 | 1 | 0 | 0 | 0 | 0 | 0 | 0 | 0 | 0 | 0 | 0 | 0 | 0 | 0 | 0 | 0 |
| Coe | | Jonathan | Expo 58 | | | | UK | 0 | 2013 | 1 | 1961 | 0 | 0 | 0 | 0 | 0 | 0 | 0 | 0 | 0 | 0 | 0 | 0 | 0 | 0 | 0 | 0 |
| Coe | | Jonathan | The house of sleep | | | | UK | 0 | 1998 | 1 | 1961 | 0 | 0 | 0 | 0 | 0 | 0 | 0 | 0 | 0 | 0 | 0 | 0 | 0 | 0 | 0 | 0 |
| Coe | | Jonathan | The rain before it falls | | | | UK | 0 | 2007 | 1 | 1961 | 0 | 0 | 0 | 0 | 0 | 0 | 0 | 0 | 0 | 0 | 0 | 0 | 0 | 0 | 0 | 0 |
| Coelho | | Paulo | Veronika decides to die | | | | BRAZIL | 0 | 1998 | 3 | 1947 | 1 | 0 | 0 | 0 | 0 | 0 | 0 | 0 | 0 | 0 | 0 | 0 | 0 | 0 | 0 | 0 |
| Coelho | | Paulo | The pilgrimage | | | | BRAZIL | 0 | 1987 | 3 | 1947 | 1 | 0 | 0 | 0 | 0 | 0 | 0 | 0 | 0 | 0 | 0 | 0 | 0 | 0 | 0 | 0 |
| Coetzee | | JM | Life & times of Michael K | | | | SOUTH AFRICA | 0 | 1983 | 5 | 1940 | 0 | 1 | 0 | 1 | 0 | 0 | 0 | 0 | 0 | 0 | 0 | 0 | 0 | 0 | 0 | 0 |
| Coetzee | | JM | The Master of Petersburg | | | | SOUTH AFRICA | 0 | 1994 | 5 | 1940 | 0 | 1 | 0 | 0 | 0 | 0 | 0 | 0 | 0 | 0 | 0 | 0 | 0 | 0 | 0 | 0 |
| Coetzee | | JM | Disgrace | | | | SOUTH AFRICA | 0 | 1999 | 5 | 1940 | 1 | 1 | 0 | 1 | 0 | 0 | 0 | 0 | 0 | 0 | 0 | 0 | 0 | 0 | 0 | 0 |
| Cognetti | | Paolo | The eight mountains | | | | ITALY | 0 | 2016 | 1 | 1978 | 1 | 0 | 0 | 0 | 0 | 0 | 0 | 0 | 0 | 0 | 0 | 0 | 0 | 0 | 0 | 0 |
| Cole | | Teju | Open city | | | | NIGERIA | 0 | 2011 | 5 | 1975 | 1 | 0 | 0 | 0 | 0 | 0 | 0 | 0 | 0 | 0 | 0 | 0 | 0 | 0 | 0 | 0 |
| Collins | | Suzanne | Catching Fire | | | | USA | 1 | 2009 | 2 | 1962 | 0 | 0 | 0 | 0 | 0 | 0 | 0 | 0 | 0 | 0 | 0 | 0 | 0 | 0 | 0 | 0 |
| Collins | | Suzanne | Mockingjay | | | | USA | 1 | 2010 | 2 | 1962 | 0 | 0 | 0 | 0 | 0 | 0 | 0 | 0 | 0 | 0 | 0 | 0 | 0 | 0 | 0 | 0 |
| Collins | | Suzanne | The hunger games | | | | USA | 1 | 2008 | 2 | 1962 | 0 | 0 | 0 | 0 | 0 | 0 | 0 | 0 | 0 | 0 | 0 | 0 | 0 | 0 | 0 | 0 |
| Collins | | Wilkie | The Moonstone | | | | UK | 0 | 1868 | 1 | 1824 | 0 | 0 | 0 | 0 | 0 | 0 | 0 | 0 | 0 | 0 | 0 | 0 | 0 | 0 | 0 | 0 |
| Collins | | Wilkie | The Woman in White | | | | UK | 0 | 1860 | 1 | 1824 | 0 | 0 | 0 | 0 | 0 | 0 | 0 | 0 | 0 | 0 | 0 | 0 | 0 | 0 | 1 | 0 |
| Collodi | | Carlo | Pinocchio | | | | ITALY | 0 | 1883 | 1 | 1826 | 1 | 0 | 0 | 0 | 0 | 0 | 0 | 0 | 0 | 0 | 0 | 0 | 0 | 0 | 0 | 0 |
| Coloane | | Francisco | Tierra del fuego | | | | CHILE | 0 | 1956 | 3 | 1910 | 1 | 0 | 0 | 0 | 0 | 0 | 0 | 0 | 0 | 0 | 0 | 0 | 0 | 0 | 0 | 0 |
| Connelly | | Michael | Dark sacred night | | | | USA | 0 | 2019 | 2 | 1956 | 0 | 0 | 0 | 0 | 0 | 0 | 0 | 0 | 0 | 0 | 0 | 0 | 0 | 0 | 0 | 0 |
| Conrad | | Joseph | Heart of darkness | | | | UK | 0 | 1899 | 1 | 1857 | 0 | 0 | 0 | 0 | 0 | 0 | 0 | 0 | 0 | 0 | 0 | 0 | 0 | 0 | 0 | 0 |
| Cook | | Diane | The New Wilderness | | | | USA | 1 | 2020 | 2 | 1976 | 0 | 0 | 0 | 0 | 0 | 0 | 0 | 0 | 0 | 0 | 0 | 0 | 0 | 0 | 1 | 0 |

| **SURNAME** | | **NAME** | **TITLE** | | **NATIONALITY** | **GENDER** | **PUB_YEAR** | **CONTINENT** | **DATE OF BIRTH** | **TRANSLATED** | **NOBEL** | **NEUSTADT** | **BOOKER** | **PULITZER** | **NEBULA** | **CAINE** | **FRANZ KAFKA** | **NBA** | **BBA** | **WOMEN'S PRIZE FOR FICTION** | **NEW YORK TIMES** | **THE GUARDIAN** | **THE NEW YORKER** | **BBC** | **ATLANTIC** |
| --- | --- | --- | --- | --- | --- | --- | --- | --- | --- | --- | --- | --- | --- | --- | --- | --- | --- | --- | --- | --- | --- | --- | --- | --- | --- |
| Cooper | Glenn | | | Three Marys | USA | 0 | 2018 | 2 | 1953 | 0 | 0 | 0 | 0 | 0 | 0 | 0 | 0 | 0 | 0 | 0 | 0 | 0 | 0 | 0 | 0 |
| Cooper | James | | | Last of the Mohicans | USA | 0 | 1826 | 2 | 1789 | 0 | 0 | 0 | 0 | 0 | 0 | 0 | 0 | 0 | 0 | 0 | 0 | 0 | 0 | 0 | 0 |
| Cooper | James | | | The Deerslayer | USA | 0 | 1841 | 2 | 1789 | 0 | 0 | 0 | 0 | 0 | 0 | 0 | 0 | 0 | 0 | 0 | 0 | 0 | 0 | 0 | 0 |
| Cooper | James | | | Afloat and Ashore | USA | 0 | 1844 | 2 | 1789 | 0 | 0 | 0 | 0 | 0 | 0 | 0 | 0 | 0 | 0 | 0 | 0 | 0 | 0 | 0 | 0 |
| Cooper | James | | | The Bravo | USA | 0 | 1831 | 2 | 1789 | 0 | 0 | 0 | 0 | 0 | 0 | 0 | 0 | 0 | 0 | 0 | 0 | 0 | 0 | 0 | 0 |
| Cooper | James | | | The Chainbearer | USA | 0 | 1845 | 2 | 1789 | 0 | 0 | 0 | 0 | 0 | 0 | 0 | 0 | 0 | 0 | 0 | 0 | 0 | 0 | 0 | 0 |
| Cooper | James | | | The Crater | USA | 0 | 1847 | 2 | 1789 | 0 | 0 | 0 | 0 | 0 | 0 | 0 | 0 | 0 | 0 | 0 | 0 | 0 | 0 | 0 | 0 |
| Cooper | James | | | The Headsman | USA | 0 | 1833 | 2 | 1789 | 0 | 0 | 0 | 0 | 0 | 0 | 0 | 0 | 0 | 0 | 0 | 0 | 0 | 0 | 0 | 0 |
| Cooper | Helene | | | The House at Sugar Beach | LIBERIA | 1 | 2008 | 5 | 1966 | 0 | 0 | 0 | 0 | 0 | 0 | 0 | 0 | 0 | 0 | 0 | 0 | 0 | 0 | 0 | 0 |
| Coovadia | Imraan | | | A spy in time | SOUTH AFRICA | 0 | 2018 | 5 | 1970 | 0 | 0 | 0 | 0 | 0 | 0 | 0 | 0 | 0 | 0 | 0 | 0 | 0 | 0 | 0 | 0 |
| Coover | Robert | | | The Public Burning | USA | 0 | 1977 | 2 | 1932 | 0 | 0 | 0 | 0 | 0 | 0 | 0 | 0 | 0 | 0 | 0 | 0 | 0 | 0 | 0 | 0 |
| Corelli | Marie | | | A Romance of Two Worlds | UK | 1 | 1886 | 1 | 1855 | 0 | 0 | 0 | 0 | 0 | 0 | 0 | 0 | 0 | 0 | 0 | 0 | 0 | 0 | 0 | 0 |
| Corelli | Marie | | | The Sorrows of Satan | UK | 1 | 1895 | 1 | 1855 | 0 | 0 | 0 | 0 | 0 | 0 | 0 | 0 | 0 | 0 | 0 | 0 | 0 | 0 | 0 | 0 |
| Correa | Armando Lucas | | | The german girl | CUBA | 0 | 2016 | 3 | 1959 | 1 | 0 | 0 | 0 | 0 | 0 | 0 | 0 | 0 | 0 | 0 | 0 | 0 | 0 | 0 | 0 |
| Cortázar | Julio | | | Cronopios and Famas | ARGENTINA | 0 | 1962 | 3 | 1914 | 1 | 0 | 0 | 0 | 0 | 0 | 0 | 0 | 0 | 0 | 0 | 0 | 0 | 0 | 0 | 0 |
| Cortázar | Julio | | | Final exam | ARGENTINA | 0 | 1950 | 3 | 1914 | 1 | 0 | 0 | 0 | 0 | 0 | 0 | 0 | 0 | 0 | 0 | 0 | 0 | 0 | 0 | 0 |
| Cossery | Albert | | | Laziness in the Fertile Valley | EGYPT | 0 | 1948 | 5 | 1913 | 1 | 0 | 0 | 0 | 0 | 0 | 0 | 0 | 0 | 0 | 0 | 0 | 0 | 0 | 0 | 0 |
| Couperus | Louis | | | Eline Vere | NETHERLANDS | 0 | 1889 | 1 | 1863 | 1 | 0 | 0 | 0 | 0 | 0 | 0 | 0 | 0 | 0 | 0 | 0 | 0 | 0 | 0 | 0 |
| Couto | Mia | | | The tuner of silences | MOZAMBIQUE | 0 | 2009 | 5 | 1955 | 1 | 0 | 1 | 0 | 0 | 0 | 0 | 0 | 0 | 0 | 0 | 0 | 0 | 0 | 0 | 0 |
| Couto | Mia | | | Confession of the Lioness | MOZAMBIQUE | 0 | 2012 | 5 | 1955 | 1 | 0 | 1 | 0 | 0 | 0 | 0 | 0 | 0 | 0 | 0 | 0 | 0 | 0 | 0 | 0 |
| Cozzens | James Gould | | | Guard of Honor | USA | 0 | 1948 | 2 | 1903 | 0 | 0 | 0 | 0 | 1 | 0 | 0 | 0 | 0 | 0 | 0 | 0 | 0 | 0 | 0 | 0 |
| Crace | Jim | | | Quarantine | UK | 0 | 1997 | 1 | 1946 | 0 | 0 | 0 | 0 | 0 | 0 | 0 | 0 | 0 | 0 | 0 | 0 | 0 | 0 | 0 | 0 |
| Crane | Stephen | | | Maggie a girl of the streets | USA | 0 | 1893 | 2 | 1871 | 0 | 0 | 0 | 0 | 0 | 0 | 0 | 0 | 0 | 0 | 0 | 0 | 0 | 0 | 0 | 0 |
| Crichton | Michael | | | Jurassic Park | USA | 0 | 1990 | 2 | 1942 | 0 | 0 | 0 | 0 | 0 | 0 | 0 | 0 | 0 | 0 | 0 | 0 | 0 | 0 | 0 | 0 |
| Cronin | AJ | | | The Citadel | UK | 0 | 1937 | 1 | 1896 | 0 | 0 | 0 | 0 | 0 | 0 | 0 | 0 | 1 | 0 | 0 | 0 | 0 | 0 | 0 | 0 |
| Cronin | AJ | | | Hatter's Castle | UK | 0 | 1931 | 1 | 1896 | 0 | 0 | 0 | 0 | 0 | 0 | 0 | 0 | 0 | 0 | 0 | 0 | 0 | 0 | 0 | 0 |
| Cunningham | Michael | | | A home at the end of the world | USA | 0 | 1990 | 2 | 1952 | 0 | 0 | 0 | 0 | 0 | 0 | 0 | 0 | 0 | 0 | 0 | 0 | 0 | 0 | 0 | 0 |
| Cunningham | Michael | | | The hours | USA | 0 | 1998 | 2 | 1952 | 0 | 0 | 0 | 0 | 1 | 0 | 0 | 0 | 0 | 0 | 0 | 0 | 0 | 0 | 0 | 0 |
| Cunningham | Michael | | | By Nightfall | USA | 0 | 2010 | 2 | 1952 | 0 | 0 | 0 | 0 | 0 | 0 | 0 | 0 | 0 | 0 | 0 | 0 | 0 | 0 | 0 | 0 |
| d'Annunzio | Gabriele | | | Pleasure | ITALY | 0 | 1889 | 1 | 1863 | 1 | 0 | 0 | 0 | 0 | 0 | 0 | 0 | 0 | 0 | 0 | 0 | 0 | 0 | 0 | 0 |
| Dahl | Roald | | | Charlie and the chocolate factory | UK | 0 | 1964 | 1 | 1916 | 0 | 0 | 0 | 0 | 0 | 0 | 0 | 0 | 0 | 1 | 0 | 0 | 0 | 0 | 1 | 0 |
| Dahl | Roald | | | James and the Giant Peach | UK | 0 | 1961 | 1 | 1916 | 0 | 0 | 0 | 0 | 0 | 0 | 0 | 0 | 0 | 1 | 0 | 0 | 0 | 0 | 0 | 0 |
| Dalton | Trent | | | Boy swallows universe | AUSTRALIA | 0 | 2018 | 6 | ? | 0 | 0 | 0 | 0 | 0 | 0 | 0 | 0 | 0 | 0 | 0 | 0 | 0 | 0 | 0 | 0 |
| Dangarembga | Tsitsi | | | This Mournable Body | ZIMBABWE | 1 | 2018 | 5 | 1959 | 0 | 0 | 0 | 0 | 0 | 0 | 0 | 0 | 0 | 0 | 0 | 0 | 1 | 0 | 1 | 0 |
| Danticat | Edwidge | | | Breath, Eyes, Memory | HAITI | 1 | 1994 | 3 | 1969 | 0 | 0 | 1 | 0 | 0 | 0 | 0 | 0 | 0 | 0 | 0 | 0 | 0 | 0 | 0 | 0 |
| Dao | Bei | | | City Gate, Open Up | CHINA | 0 | 2010 | 4 | 1949 | 1 | 0 | 0 | 0 | 0 | 0 | 0 | 0 | 0 | 0 | 0 | 0 | 0 | 0 | 0 | 0 |
| Davies | Robertson | | | What's Bred in the Bone | CANADA | 0 | 1985 | 2 | 1913 | 0 | 0 | 0 | 0 | 0 | 0 | 0 | 0 | 0 | 0 | 0 | 0 | 0 | 0 | 0 | 0 |
| Davis | Harold Lenoir | | | Honey in the Horn | USA | 0 | 1935 | 2 | 1894 | 0 | 0 | 0 | 0 | 1 | 0 | 0 | 0 | 0 | 0 | 0 | 0 | 0 | 0 | 0 | 0 |
| Dawkins | Richard | | | The God delusion | UK | 0 | 2006 | 1 | 1941 | 0 | 0 | 0 | 0 | 0 | 0 | 0 | 0 | 0 | 1 | 0 | 0 | 0 | 0 | 0 | 0 |
| Dazai | Osamu | | | No Longer Human | JAPAN | 0 | 1948 | 4 | 1909 | 1 | 0 | 0 | 0 | 0 | 0 | 0 | 0 | 0 | 0 | 0 | 0 | 0 | 0 | 0 | 0 |
| De Amicis | Edmondo | | | Heart | ITALY | 0 | 1886 | 1 | 1846 | 1 | 0 | 0 | 0 | 0 | 0 | 0 | 0 | 0 | 0 | 0 | 0 | 0 | 0 | 0 | 0 |
| de Assis Machado Joaquim Maria  de Assis Machado Joaquim Maria | | | he Posthumous Memoirs of Brás Cuba  Dom Casmurro | | BRAZIL  BRAZIL | 0  0 | 1881  1899 | 3  3 | 1839  1839 | 1  1 | 0  0 | 0  0 | 0  0 | 0  0 | 0  0 | 0  0 | 0  0 | 0  0 | 0  0 | 0  0 | 0  0 | 0  0 | 0  0 | 0  0 | 0  0 |
| de Balzac | Honoré | | | The human comedy | FRANCE | 0 | 1831 | 1 | 1799 | 1 | 0 | 0 | 0 | 0 | 0 | 0 | 0 | 0 | 0 | 0 | 0 | 0 | 0 | 0 | 0 |
| de Balzac | Honoré | | | Old man Goriot | FRANCE | 0 | 1835 | 1 | 1799 | 1 | 0 | 0 | 0 | 0 | 0 | 0 | 0 | 0 | 0 | 0 | 0 | 0 | 0 | 0 | 0 |
| de Balzac | Honoré | | | The magic skin | FRANCE | 0 | 1831 | 1 | 1799 | 1 | 0 | 0 | 0 | 0 | 0 | 0 | 0 | 0 | 0 | 0 | 0 | 0 | 0 | 0 | 0 |
| de Balzac | Honoré | | | A harlot high and low | FRANCE | 0 | 1838 | 1 | 1799 | 1 | 0 | 0 | 0 | 0 | 0 | 0 | 0 | 0 | 0 | 0 | 0 | 0 | 0 | 0 | 0 |
| de Beauvoir | Simone | | | Memoirs of a Dutiful Daughter | FRANCE | 1 | 1958 | 1 | 1908 | 1 | 0 | 0 | 0 | 0 | 0 | 0 | 0 | 0 | 0 | 0 | 0 | 0 | 0 | 0 | 0 |
| de Beauvoir | Simone | | | The Mandarins | FRANCE | 1 | 1954 | 1 | 1908 | 1 | 0 | 0 | 0 | 0 | 0 | 0 | 0 | 0 | 0 | 0 | 0 | 0 | 0 | 0 | 0 |
| de Beauvoir | Simone | | | She came to stay | FRANCE | 1 | 1943 | 1 | 1908 | 1 | 0 | 0 | 0 | 0 | 0 | 0 | 0 | 0 | 0 | 0 | 0 | 0 | 0 | 0 | 0 |
| De Giovanni | Maurizio | | | Everyone in their place | ITALY | 0 | 2009 | 1 | 1958 | 1 | 0 | 0 | 0 | 0 | 0 | 0 | 0 | 0 | 0 | 0 | 0 | 0 | 0 | 0 | 0 |
| De Giovanni | Maurizio | | | The bastards of Pizzofalcone | ITALY | 0 | 2013 | 1 | 1958 | 1 | 0 | 0 | 0 | 0 | 0 | 0 | 0 | 0 | 0 | 0 | 0 | 0 | 0 | 0 | 0 |
| De Giovanni | Maurizio | | | arkness for the bastards of Pizzofalcon | ITALY | 0 | 2017 | 1 | 1958 | 1 | 0 | 0 | 0 | 0 | 0 | 0 | 0 | 0 | 0 | 0 | 0 | 0 | 0 | 0 | 0 |
| de Maupassant | Guy | | | A life | FRANCE | 0 | 1883 | 1 | 1850 | 1 | 0 | 0 | 0 | 0 | 0 | 0 | 0 | 0 | 0 | 0 | 0 | 0 | 0 | 0 | 0 |
| de Maupassant | Guy | | | Bel-Ami | FRANCE | 0 | 1888 | 1 | 1850 | 1 | 0 | 0 | 0 | 0 | 0 | 0 | 0 | 0 | 0 | 0 | 0 | 0 | 0 | 0 | 0 |
| de Maupassant | Guy | | | Pierre et Jean | FRANCE | 0 | 1885 | 1 | 1850 | 1 | 0 | 0 | 0 | 0 | 0 | 0 | 0 | 0 | 0 | 0 | 0 | 0 | 0 | 0 | 0 |
| De Pontes Peeble Frances | | | The air you breathe | | BRAZIL | 1 | 2018 | 3 | ? | 1 | 0 | 0 | 0 | 0 | 0 | 0 | 0 | 0 | 0 | 0 | 0 | 0 | 0 | 0 | 0 |
| de Recacoechea | Juan | | | American Visa | BOLIVIA | 0 | 2007 | 3 | 1935 | 1 | 0 | 0 | 0 | 0 | 0 | 0 | 0 | 0 | 0 | 0 | 0 | 0 | 0 | 0 | 0 |
| de Roberto | Federico | | | The Viceroys | ITALY | 0 | 1894 | 1 | 1861 | 1 | 0 | 0 | 0 | 0 | 0 | 0 | 0 | 0 | 0 | 0 | 0 | 0 | 0 | 0 | 0 |
| de Sade | Marquis | | | Justine | FRANCE | 0 | 1791 | 1 | 1740 | 1 | 0 | 0 | 0 | 0 | 0 | 0 | 0 | 0 | 0 | 0 | 0 | 0 | 0 | 0 | 0 |
| de Saint-Exupéry | Antoine | | | The little prince | FRANCE | 0 | 1943 | 1 | 1900 | 1 | 0 | 0 | 0 | 0 | 0 | 0 | 0 | 0 | 0 | 0 | 0 | 0 | 0 | 0 | 0 |
| de Saint-Pierre | Bernardin | | | Paul and Virginia | FRANCE | 0 | 1788 | 1 | 1737 | 1 | 0 | 0 | 0 | 0 | 0 | 0 | 0 | 0 | 0 | 0 | 0 | 0 | 0 | 0 | 0 |
| de Waal | Edmund | | | The hare with amber eyes | UK | 0 | 2010 | 1 | 1964 | 0 | 0 | 0 | 0 | 0 | 0 | 0 | 0 | 0 | 1 | 0 | 0 | 0 | 0 | 0 | 0 |
| Defoe | Daniel | | | A Journal of the Plague Year | UK | 0 | 1722 | 1 | 1660 | 0 | 0 | 0 | 0 | 0 | 0 | 0 | 0 | 0 | 0 | 0 | 0 | 1 | 0 | 0 | 0 |
| Defoe | Daniel | | | Moll Flanders | UK | 0 | 1722 | 1 | 1660 | 0 | 0 | 0 | 0 | 0 | 0 | 0 | 0 | 0 | 0 | 0 | 0 | 0 | 0 | 0 | 0 |
| Defoe | Daniel | | | Robinson Crusoe | UK | 0 | 1719 | 1 | 1660 | 0 | 0 | 0 | 0 | 0 | 0 | 0 | 0 | 0 | 0 | 0 | 0 | 0 | 0 | 0 | 0 |
| Defoe | Daniel | | | The Fortunate Mistress | UK | 0 | 1724 | 1 | 1660 | 0 | 0 | 0 | 0 | 0 | 0 | 0 | 0 | 0 | 0 | 0 | 0 | 0 | 0 | 0 | 0 |
| Defoe | Daniel | | | Captain Singleton | UK | 0 | 1720 | 1 | 1660 | 0 | 0 | 0 | 0 | 0 | 0 | 0 | 0 | 0 | 0 | 0 | 0 | 0 | 0 | 0 | 0 |
| Defoe | Daniel | | | Memoirs of a Cavalier | UK | 0 | 1720 | 1 | 1660 | 0 | 0 | 0 | 0 | 0 | 0 | 0 | 0 | 0 | 0 | 0 | 0 | 0 | 0 | 0 | 0 |
| Defoe | Daniel | | | e Farther Adventures of Robinson Crus | UK | 0 | 1719 | 1 | 1660 | 0 | 0 | 0 | 0 | 0 | 0 | 0 | 0 | 0 | 0 | 0 | 0 | 0 | 0 | 0 | 0 |
| Deledda | Grazia | | | Church of solitude | ITALY | 1 | 1936 | 1 | 1871 | 1 | 1 | 0 | 0 | 0 | 0 | 0 | 0 | 0 | 0 | 0 | 0 | 0 | 0 | 0 | 0 |
| Deledda | Grazia | | | The Mother | ITALY | 1 | 1919 | 1 | 1871 | 1 | 1 | 0 | 0 | 0 | 0 | 0 | 0 | 0 | 0 | 0 | 0 | 0 | 0 | 0 | 0 |
| Delijani | Sahar | | | Children of the Jacaranda Tree | IRAN | 1 | 2013 | 4 | 1983 | 0 | 0 | 0 | 0 | 0 | 0 | 0 | 0 | 0 | 0 | 0 | 0 | 0 | 0 | 0 | 0 |
| DeLillo | Don | | | Underworld | USA | 0 | 1997 | 2 | 1936 | 0 | 0 | 0 | 0 | 0 | 0 | 0 | 0 | 0 | 0 | 0 | 0 | 0 | 0 | 0 | 0 |
| DeLillo | Don | | | Cosmopolis | USA | 0 | 2003 | 2 | 1936 | 0 | 0 | 0 | 0 | 0 | 0 | 0 | 0 | 0 | 0 | 0 | 0 | 0 | 0 | 0 | 0 |
| DeLillo | Don | | | White noise | USA | 0 | 1985 | 2 | 1936 | 0 | 0 | 0 | 0 | 0 | 0 | 0 | 0 | 1 | 0 | 0 | 0 | 0 | 0 | 0 | 0 |
| Dennis-Benn | Nicole | | | Here Comes the Sun | JAMAICA | 1 | 2016 | 3 | 1982 | 0 | 0 | 0 | 0 | 0 | 0 | 0 | 0 | 0 | 0 | 0 | 0 | 0 | 0 | 0 | 0 |
| Desai | Anita | | | Baumgartner's Bombay | INDIA | 1 | 1988 | 4 | 1937 | 0 | 0 | 0 | 0 | 0 | 0 | 0 | 0 | 0 | 0 | 0 | 0 | 0 | 0 | 0 | 0 |
| Desai | Anita | | | In custody | INDIA | 1 | 1984 | 4 | 1937 | 0 | 0 | 0 | 0 | 0 | 0 | 0 | 0 | 0 | 0 | 0 | 0 | 0 | 0 | 0 | 0 |
| Desai | Anita | | | Clear Light of Day | INDIA | 1 | 1980 | 4 | 1937 | 0 | 0 | 0 | 0 | 0 | 0 | 0 | 0 | 0 | 0 | 0 | 0 | 0 | 0 | 0 | 0 |
| Desai | Anita | | | Fire On the Mountain | INDIA | 1 | 1977 | 4 | 1937 | 0 | 0 | 0 | 0 | 0 | 0 | 0 | 0 | 0 | 0 | 0 | 0 | 0 | 0 | 0 | 0 |
| Desai | Anita | | | Fasting, Feasting | INDIA | 1 | 1999 | 4 | 1937 | 0 | 0 | 0 | 0 | 0 | 0 | 0 | 0 | 0 | 0 | 0 | 0 | 0 | 0 | 0 | 0 |
| Desai | Kiran | | | The Inheritance of Loss | INDIA | 1 | 2006 | 4 | 1971 | 0 | 0 | 0 | 1 | 0 | 0 | 0 | 0 | 0 | 0 | 0 | 0 | 0 | 0 | 0 | 0 |
| deWitt | Patrick | | | The Sisters Brothers | CANADA | 0 | 2011 | 2 | 1975 | 0 | 0 | 0 | 0 | 0 | 0 | 0 | 0 | 0 | 0 | 0 | 0 | 0 | 0 | 0 | 0 |
| Díaz | Junot | | | Drown | DOMINICAN REP. | 0 | 1996 | 3 | 1968 | 0 | 0 | 0 | 0 | 0 | 0 | 0 | 0 | 0 | 0 | 0 | 0 | 0 | 0 | 0 | 0 |
| Díaz | Junot | | | The Brief Wondrous Life of Oscar Wao | DOMINICAN REP. | 0 | 2007 | 3 | 1968 | 0 | 0 | 0 | 0 | 1 | 0 | 0 | 0 | 0 | 0 | 0 | 0 | 0 | 0 | 0 | 0 |
| Díaz | Junot | | | This Is How You Lose Her | DOMINICAN REP. | 0 | 2012 | 3 | 1968 | 0 | 0 | 0 | 0 | 0 | 0 | 0 | 0 | 0 | 0 | 0 | 0 | 0 | 0 | 0 | 0 |

| **SURNAME** | **NAME** | | **TITLE** | **NATIONALITY** | | **GENDER** | **PUB_YEAR** | **CONTINENT** | **DATE OF BIRTH** | **TRANSLATED** | **NOBEL** | **NEUSTADT** | **BOOKER** | **PULITZER** | **NEBULA** | **CAINE** | **FRANZ KAFKA** | **NBA** | **BBA** | **WOMEN'S PRIZE FOR FICTION** | **NEW YORK TIMES** | **THE GUARDIAN** | **THE NEW YORKER** | **BBC** | **ATLANTIC** |
| --- | --- | --- | --- | --- | --- | --- | --- | --- | --- | --- | --- | --- | --- | --- | --- | --- | --- | --- | --- | --- | --- | --- | --- | --- | --- |
| Dick | Philip Kindred | | Flow my tears the policeman said | | USA | 0 | 1974 | 2 | 1928 | 0 | 0 | 0 | 0 | 0 | 0 | 0 | 0 | 0 | 0 | 0 | 0 | 0 | 0 | 0 | 0 |
| Dick | Philip Kindred | | Do androids dream of electric sheep | | USA | 0 | 1968 | 2 | 1928 | 0 | 0 | 0 | 0 | 0 | 0 | 0 | 0 | 0 | 0 | 0 | 0 | 0 | 0 | 0 | 0 |
| Dick | Philip Kindred | | Deus Irae | | USA | 0 | 1976 | 2 | 1928 | 0 | 0 | 0 | 0 | 0 | 0 | 0 | 0 | 0 | 0 | 0 | 0 | 0 | 0 | 0 | 0 |
| Dickens | Charles | | The Pickwick papers | | UK | 0 | 1837 | 1 | 1812 | 0 | 0 | 0 | 0 | 0 | 0 | 0 | 0 | 0 | 0 | 0 | 0 | 0 | 0 | 0 | 0 |
| Dickens | Charles | | Great expectations | | UK | 0 | 1861 | 1 | 1812 | 0 | 0 | 0 | 0 | 0 | 0 | 0 | 0 | 0 | 0 | 0 | 0 | 0 | 0 | 1 | 0 |
| Dickens | Charles | | A tale of two cities | | UK | 0 | 1859 | 1 | 1812 | 0 | 0 | 0 | 0 | 0 | 0 | 0 | 0 | 0 | 0 | 0 | 0 | 0 | 0 | 1 | 0 |
| Dickens | Charles | | David Copperfield | | UK | 0 | 1850 | 1 | 1812 | 0 | 0 | 0 | 0 | 0 | 0 | 0 | 0 | 0 | 0 | 0 | 0 | 0 | 0 | 1 | 0 |
| Dickens | Charles | | A Christmas Carol | | UK | 0 | 1843 | 1 | 1812 | 0 | 0 | 0 | 0 | 0 | 0 | 0 | 0 | 0 | 0 | 0 | 0 | 0 | 0 | 1 | 0 |
| Dickens | Charles | | Hard Times | | UK | 0 | 1854 | 1 | 1812 | 0 | 0 | 0 | 0 | 0 | 0 | 0 | 0 | 0 | 0 | 0 | 0 | 0 | 0 | 0 | 0 |
| Dickens | Charles | | Oliver Twist | | UK | 0 | 1838 | 1 | 1812 | 0 | 0 | 0 | 0 | 0 | 0 | 0 | 0 | 0 | 0 | 0 | 0 | 0 | 0 | 0 | 0 |
| Dicker | Joël | | The truth about the Harry Quebert affair | | SWITZERLAND | 0 | 2012 | 1 | 1985 | 1 | 0 | 0 | 0 | 0 | 0 | 0 | 0 | 0 | 0 | 0 | 0 | 0 | 0 | 0 | 0 |
| Didion | Joan | | The year of magical thinking | | USA | 1 | 2005 | 2 | 1934 | 0 | 0 | 0 | 0 | 0 | 0 | 0 | 0 | 0 | 0 | 0 | 0 | 0 | 0 | 0 | 0 |
| Diop | Boubacar Boris | | Kaveena | | SENEGAL | 0 | 2006 | 5 | 1946 | 1 | 0 | 1 | 0 | 0 | 0 | 0 | 0 | 0 | 0 | 0 | 0 | 0 | 0 | 0 | 0 |
| Dirie | Waris | | Desert flower | | SOMALIA | 1 | 1998 | 5 | 1965 | 0 | 0 | 0 | 0 | 0 | 0 | 0 | 0 | 0 | 0 | 0 | 0 | 0 | 0 | 0 | 0 |
| Disraeli | Benjamin | | Coningsby or the New Generation | | UK | 0 | 1844 | 1 | 1804 | 0 | 0 | 0 | 0 | 0 | 0 | 0 | 0 | 0 | 0 | 0 | 0 | 0 | 0 | 0 | 0 |
| Disraeli | Benjamin | | Endymion | | UK | 0 | 1880 | 1 | 1804 | 0 | 0 | 0 | 0 | 0 | 0 | 0 | 0 | 0 | 0 | 0 | 0 | 0 | 0 | 0 | 0 |
| Disraeli | Benjamin | | Henrietta Temple | | UK | 0 | 1837 | 1 | 1804 | 0 | 0 | 0 | 0 | 0 | 0 | 0 | 0 | 0 | 0 | 0 | 0 | 0 | 0 | 0 | 0 |
| Disraeli | Benjamin | | Ixion in Heaven | | UK | 0 | 1834 | 1 | 1804 | 0 | 0 | 0 | 0 | 0 | 0 | 0 | 0 | 0 | 0 | 0 | 0 | 0 | 0 | 0 | 0 |
| Disraeli | Benjamin | | Sybil or The Two Nations | | UK | 0 | 1845 | 1 | 1804 | 0 | 0 | 0 | 0 | 0 | 0 | 0 | 0 | 0 | 0 | 0 | 0 | 0 | 0 | 0 | 0 |
| Disraeli | Benjamin | | Tancred or the New Crusade | | UK | 0 | 1847 | 1 | 1804 | 0 | 0 | 0 | 0 | 0 | 0 | 0 | 0 | 0 | 0 | 0 | 0 | 0 | 0 | 0 | 0 |
| Disraeli | Benjamin | | The Voyage of Captain Popanilla | | UK | 0 | 1828 | 1 | 1804 | 0 | 0 | 0 | 0 | 0 | 0 | 0 | 0 | 0 | 0 | 0 | 0 | 0 | 0 | 0 | 0 |
| Divakaruni | Chitra Banerjee | | The palace of illusions | | INDIA | 1 | 2008 | 4 | 1956 | 0 | 0 | 0 | 0 | 0 | 0 | 0 | 0 | 0 | 0 | 0 | 0 | 0 | 0 | 0 | 0 |
| Divakaruni | Chitra Banerjee | | Sister of my heart | | INDIA | 1 | 1999 | 4 | 1956 | 0 | 0 | 0 | 0 | 0 | 0 | 0 | 0 | 0 | 0 | 0 | 0 | 0 | 0 | 0 | 0 |
| Djebar | Assia | | The tongue's blood does not run dry | | ALGERIA | 1 | 1997 | 5 | 1936 | 1 | 0 | 1 | 0 | 0 | 0 | 0 | 0 | 0 | 0 | 0 | 0 | 0 | 0 | 0 | 0 |
| Djebar | Assia | | So Vast the Prison | | ALGERIA | 1 | 1995 | 5 | 1936 | 1 | 0 | 1 | 0 | 0 | 0 | 0 | 0 | 0 | 0 | 0 | 0 | 0 | 0 | 0 | 0 |
| Döblin | Alfred | | Berlin Alexanderplatz | | GERMANY | 0 | 1929 | 1 | 1878 | 1 | 0 | 0 | 0 | 0 | 0 | 0 | 0 | 0 | 0 | 0 | 0 | 0 | 0 | 0 | 0 |
| Doctorow | EL | | Ragtime | | USA | 0 | 1975 | 2 | 1931 | 0 | 0 | 0 | 0 | 0 | 0 | 0 | 0 | 0 | 0 | 0 | 0 | 0 | 0 | 0 | 0 |
| Doerr | Anthony | | All the Light We Cannot See | | USA | 0 | 2014 | 2 | 1973 | 0 | 0 | 0 | 0 | 1 | 0 | 0 | 0 | 0 | 0 | 0 | 1 | 0 | 0 | 0 | 0 |
| Doerr | Anthony | | About Grace | | USA | 0 | 2004 | 2 | 1973 | 0 | 0 | 0 | 0 | 0 | 0 | 0 | 0 | 0 | 0 | 0 | 0 | 0 | 0 | 0 | 0 |
| Donleavy | JP | | The ginger man | | USA | 0 | 1955 | 2 | 1926 | 0 | 0 | 0 | 0 | 0 | 0 | 0 | 0 | 0 | 0 | 0 | 0 | 0 | 0 | 0 | 0 |
| Donoghue | Emma | | Room | | IRELAND | 1 | 2010 | 1 | 1969 | 0 | 0 | 0 | 0 | 0 | 0 | 0 | 0 | 0 | 1 | 0 | 1 | 0 | 0 | 0 | 0 |
| Doshi | Tishani | | The pleasure seekers | | INDIA | 1 | 2010 | 4 | 1975 | 0 | 0 | 0 | 0 | 0 | 0 | 0 | 0 | 0 | 0 | 0 | 0 | 0 | 0 | 0 | 0 |
| Dostoevsky | Fyodor | | The idiot | | RUSSIA | 0 | 1869 | 1 | 1821 | 1 | 0 | 0 | 0 | 0 | 0 | 0 | 0 | 0 | 0 | 0 | 0 | 0 | 0 | 0 | 0 |
| Dostoevsky | Fyodor | | Brothers Karamazov | | RUSSIA | 0 | 1880 | 1 | 1821 | 1 | 0 | 0 | 0 | 0 | 0 | 0 | 0 | 0 | 0 | 0 | 0 | 0 | 0 | 0 | 0 |
| Dostoevsky | Fyodor | | White nights | | RUSSIA | 0 | 1848 | 1 | 1821 | 1 | 0 | 0 | 0 | 0 | 0 | 0 | 0 | 0 | 0 | 0 | 0 | 0 | 0 | 0 | 0 |
| Dostoevsky | Fyodor | | Notes from the underground | | RUSSIA | 0 | 1864 | 1 | 1821 | 1 | 0 | 0 | 0 | 0 | 0 | 0 | 0 | 0 | 0 | 0 | 0 | 0 | 0 | 0 | 0 |
| Dostoevsky | Fyodor | | Crime and punishment | | RUSSIA | 0 | 1866 | 1 | 1821 | 1 | 0 | 0 | 0 | 0 | 0 | 0 | 0 | 0 | 0 | 0 | 0 | 0 | 0 | 1 | 0 |
| Douaihy | Jabbour | | June rain | | LEBANON | 0 | 2010 | 4 | 1949 | 1 | 0 | 0 | 0 | 0 | 0 | 0 | 0 | 0 | 0 | 0 | 0 | 0 | 0 | 0 | 0 |
| Douglass | Frederick | | My Bondage and My Freedom | | USA | 0 | 1855 | 2 | 1818 | 0 | 0 | 0 | 0 | 0 | 0 | 0 | 0 | 0 | 0 | 0 | 0 | 0 | 0 | 0 | 0 |
| Dovlatov | Sergei | | Pushkin hills | | RUSSIA | 0 | 1983 | 1 | 1941 | 1 | 0 | 0 | 0 | 0 | 0 | 0 | 0 | 0 | 0 | 0 | 0 | 0 | 0 | 0 | 0 |
| Dowlatabadi | Mahmoud | | Missing Soluch | | IRAN | 0 | 1979 | 4 | 1940 | 1 | 0 | 0 | 0 | 0 | 0 | 0 | 0 | 0 | 0 | 0 | 0 | 0 | 0 | 0 | 0 |
| Dowlatabadi | Mahmoud | | The colonel | | IRAN | 0 | 2011 | 4 | 1940 | 1 | 0 | 0 | 0 | 0 | 0 | 0 | 0 | 0 | 0 | 0 | 0 | 0 | 0 | 0 | 0 |
| Dowlatabadi | Mahmoud | | Thirst | | IRAN | 0 | 2014 | 4 | 1940 | 1 | 0 | 0 | 0 | 0 | 0 | 0 | 0 | 0 | 0 | 0 | 0 | 0 | 0 | 0 | 0 |
| Doxiadis | Apostolos | Uncle Petros and Goldbach's Conjectur | |  | GREECE | 0 | 1992 | 1 | 1953 | 1 | 0 | 0 | 0 | 0 | 0 | 0 | 0 | 0 | 0 | 0 | 0 | 0 | 0 | 0 | 0 |
| Doyle | Arthur Conan | | A study in scarlet | | UK | 0 | 1887 | 1 | 1859 | 0 | 0 | 0 | 0 | 0 | 0 | 0 | 0 | 0 | 0 | 0 | 0 | 0 | 0 | 0 | 0 |
| Doyle | Arthur Conan | | The sign of the four | | UK | 0 | 1890 | 1 | 1859 | 0 | 0 | 0 | 0 | 0 | 0 | 0 | 0 | 0 | 0 | 0 | 0 | 0 | 0 | 0 | 0 |
| Doyle | Arthur Conan | | The hound of the Baskervilles | | UK | 0 | 1902 | 1 | 1859 | 0 | 0 | 0 | 0 | 0 | 0 | 0 | 0 | 0 | 0 | 0 | 0 | 0 | 0 | 0 | 0 |
| Doyle | Arthur Conan | | His Last Bow | | UK | 0 | 1917 | 1 | 1859 | 0 | 0 | 0 | 0 | 0 | 0 | 0 | 0 | 0 | 0 | 0 | 0 | 0 | 0 | 0 | 0 |
| Doyle | Arthur Conan | | The Lost World | | UK | 0 | 1912 | 1 | 1859 | 0 | 0 | 0 | 0 | 0 | 0 | 0 | 0 | 0 | 0 | 0 | 0 | 0 | 0 | 0 | 0 |
| Doyle | Arthur Conan | | The Memoirs of Sherlock Holmes | | UK | 0 | 1894 | 1 | 1859 | 0 | 0 | 0 | 0 | 0 | 0 | 0 | 0 | 0 | 0 | 0 | 0 | 0 | 0 | 0 | 0 |
| Doyle | Arthur Conan | | The Return of Sherlock Holmes | | UK | 0 | 1903 | 1 | 1859 | 0 | 0 | 0 | 0 | 0 | 0 | 0 | 0 | 0 | 0 | 0 | 0 | 0 | 0 | 0 | 0 |
| Doyle | Roddy | | Paddy Clarke Ha Ha Ha | | IRELAND | 0 | 1993 | 1 | 1958 | 0 | 0 | 0 | 1 | 0 | 0 | 0 | 0 | 0 | 1 | 0 | 0 | 0 | 0 | 0 | 0 |
| Doyle | Roddy | | Bullfighting | | IRELAND | 0 | 2011 | 1 | 1958 | 0 | 0 | 0 | 0 | 0 | 0 | 0 | 0 | 0 | 1 | 0 | 0 | 0 | 0 | 0 | 0 |
| Drury | Allen | | A God Against the Gods | | USA | 0 | 1976 | 2 | 1918 | 0 | 0 | 0 | 0 | 0 | 0 | 0 | 0 | 0 | 0 | 0 | 0 | 0 | 0 | 0 | 0 |
| Drury | Allen | | A Shade of Difference | | USA | 0 | 1962 | 2 | 1918 | 0 | 0 | 0 | 0 | 0 | 0 | 0 | 0 | 0 | 0 | 0 | 0 | 0 | 0 | 0 | 0 |
| Drury | Allen | | Advise and Consent | | USA | 0 | 1959 | 2 | 1918 | 0 | 0 | 0 | 0 | 1 | 0 | 0 | 0 | 0 | 0 | 0 | 0 | 0 | 0 | 0 | 0 |
| Drury | Allen | | Capable of Honor | | USA | 0 | 1966 | 2 | 1918 | 0 | 0 | 0 | 0 | 0 | 0 | 0 | 0 | 0 | 0 | 0 | 0 | 0 | 0 | 0 | 0 |
| Drury | Allen | | Come Nineveh, Come Tyre | | USA | 0 | 1973 | 2 | 1918 | 0 | 0 | 0 | 0 | 0 | 0 | 0 | 0 | 0 | 0 | 0 | 0 | 0 | 0 | 0 | 0 |
| Drury | Allen | | Preserve and Protect | | USA | 0 | 1968 | 2 | 1918 | 0 | 0 | 0 | 0 | 0 | 0 | 0 | 0 | 0 | 0 | 0 | 0 | 0 | 0 | 0 | 0 |
| Drury | Allen | | The Promise of Joy | | USA | 0 | 1975 | 2 | 1918 | 0 | 0 | 0 | 0 | 0 | 0 | 0 | 0 | 0 | 0 | 0 | 0 | 0 | 0 | 0 | 0 |
| du Gard | Roger | | The Thibaults | | FRANCE | 0 | 1922 | 1 | 1881 | 1 | 1 | 0 | 0 | 0 | 0 | 0 | 0 | 0 | 0 | 0 | 0 | 0 | 0 | 0 | 0 |
| du Maurier | Daphne | | My cousin Rachel | | UK | 1 | 1951 | 1 | 1907 | 0 | 0 | 0 | 0 | 0 | 0 | 0 | 0 | 0 | 0 | 0 | 0 | 0 | 0 | 0 | 0 |
| du Maurier | Daphne | | Rebecca | | UK | 1 | 1938 | 1 | 1907 | 0 | 0 | 0 | 0 | 0 | 0 | 0 | 0 | 1 | 0 | 0 | 0 | 0 | 0 | 1 | 0 |
| du Maurier | Daphne | | Jamaica Inn | | UK | 1 | 1936 | 1 | 1907 | 0 | 0 | 0 | 0 | 0 | 0 | 0 | 0 | 0 | 0 | 0 | 0 | 0 | 0 | 0 | 0 |
| du Maurier | Daphne | | The House on the Strand | | UK | 1 | 1969 | 1 | 1907 | 0 | 0 | 0 | 0 | 0 | 0 | 0 | 0 | 0 | 0 | 0 | 0 | 0 | 0 | 0 | 0 |
| du Maurier | Daphne | | Frenchman's Creek | | UK | 1 | 1941 | 1 | 1907 | 0 | 0 | 0 | 0 | 0 | 0 | 0 | 0 | 0 | 0 | 0 | 0 | 0 | 0 | 0 | 0 |
| du Maurier | Daphne | | The King's General | | UK | 1 | 1946 | 1 | 1907 | 0 | 0 | 0 | 0 | 0 | 0 | 0 | 0 | 0 | 0 | 0 | 0 | 0 | 0 | 0 | 0 |
| Dubus III | Andre | | House of Sand and Fog | | USA | 0 | 1999 | 2 | 1959 | 0 | 0 | 0 | 0 | 0 | 0 | 0 | 0 | 0 | 0 | 0 | 0 | 0 | 0 | 0 | 0 |
| Dueñas | María | | The vineyard | | SPAIN | 1 | 2015 | 1 | 1964 | 1 | 0 | 0 | 0 | 0 | 0 | 0 | 0 | 0 | 0 | 0 | 0 | 0 | 0 | 0 | 0 |
| Dumas | Alexandre | | The count of Monte Cristo | | FRANCE | 0 | 1846 | 1 | 1802 | 1 | 0 | 0 | 0 | 0 | 0 | 0 | 0 | 0 | 0 | 0 | 0 | 0 | 0 | 1 | 0 |
| Dumas | Alexandre | | The three musketeers | | FRANCE | 0 | 1844 | 1 | 1802 | 1 | 0 | 0 | 0 | 0 | 0 | 0 | 0 | 0 | 0 | 0 | 0 | 0 | 0 | 0 | 0 |
| Duras | Marguerite | | The lover | | FRANCE | 1 | 1984 | 1 | 1914 | 1 | 0 | 0 | 0 | 0 | 0 | 0 | 0 | 0 | 0 | 0 | 0 | 0 | 0 | 0 | 0 |
| Durham | David Anthony | | Acacia: The Other Lands | | USA | 0 | 2009 | 2 | 1969 | 0 | 0 | 0 | 0 | 0 | 0 | 0 | 0 | 0 | 0 | 0 | 0 | 0 | 0 | 0 | 0 |
| Durham | David Anthony | | Acacia: The Sacred Band | | USA | 0 | 2011 | 2 | 1969 | 0 | 0 | 0 | 0 | 0 | 0 | 0 | 0 | 0 | 0 | 0 | 0 | 0 | 0 | 0 | 0 |
| Durham | David Anthony | | Acacia: The War with the Mein | | USA | 0 | 2007 | 2 | 1969 | 0 | 0 | 0 | 0 | 0 | 0 | 0 | 0 | 0 | 0 | 0 | 0 | 0 | 0 | 0 | 0 |
| Durham | David Anthony | | Gabriel's Story | | USA | 0 | 2001 | 2 | 1969 | 0 | 0 | 0 | 0 | 0 | 0 | 0 | 0 | 0 | 0 | 0 | 0 | 0 | 0 | 0 | 0 |
| Durrell | Gerald | | My family and other animals | | UK | 0 | 1956 | 1 | 1925 | 0 | 0 | 0 | 0 | 0 | 0 | 0 | 0 | 0 | 0 | 0 | 0 | 0 | 0 | 0 | 0 |
| Eco | Umberto | | The name of the rose | | ITALY | 0 | 1980 | 1 | 1932 | 1 | 0 | 0 | 0 | 0 | 0 | 0 | 0 | 0 | 0 | 0 | 0 | 0 | 0 | 0 | 0 |
| Edgeworth | Maria | | Castle Rackrent | | UK | 1 | 1800 | 1 | 1768 | 0 | 0 | 0 | 0 | 0 | 0 | 0 | 0 | 0 | 0 | 0 | 0 | 0 | 0 | 0 | 0 |
| Edgeworth | Maria | | Belinda | | UK | 1 | 1801 | 1 | 1768 | 0 | 0 | 0 | 0 | 0 | 0 | 0 | 0 | 0 | 0 | 0 | 0 | 0 | 0 | 0 | 0 |
| Edgeworth | Maria | | Ormond | | UK | 1 | 1817 | 1 | 1768 | 0 | 0 | 0 | 0 | 0 | 0 | 0 | 0 | 0 | 0 | 0 | 0 | 0 | 0 | 0 | 0 |
| Edgeworth | Maria | | The Absentee | | UK | 1 | 1812 | 1 | 1768 | 0 | 0 | 0 | 0 | 0 | 0 | 0 | 0 | 0 | 0 | 0 | 0 | 0 | 0 | 0 | 0 |
| Edugyan | Esi | | Half-Blood Blues | | CANADA | 1 | 2011 | 2 | 1978 | 0 | 0 | 0 | 0 | 0 | 0 | 0 | 0 | 0 | 0 | 0 | 0 | 0 | 0 | 0 | 0 |
| Egan | Jennifer | | A Visit from the Goon Squad | | USA | 1 | 2010 | 2 | 1962 | 0 | 0 | 0 | 0 | 1 | 0 | 0 | 0 | 0 | 1 | 0 | 1 | 0 | 0 | 0 | 0 |
| Eggers | Dave | | The circle | | USA | 0 | 2013 | 2 | 1970 | 0 | 0 | 0 | 0 | 0 | 0 | 0 | 0 | 0 | 0 | 0 | 0 | 1 | 0 | 0 | 0 |
| Eggers | Dave | | heartbreaking work of staggering geniu | | USA | 0 | 2000 | 2 | 1970 | 0 | 0 | 0 | 0 | 0 | 0 | 0 | 0 | 0 | 0 | 0 | 0 | 0 | 0 | 0 | 0 |
| El Akkad | Omar | | American War | | EGYPT | 0 | 2017 | 5 | 1982 | 0 | 0 | 0 | 0 | 0 | 0 | 0 | 0 | 0 | 0 | 0 | 0 | 0 | 0 | 0 | 0 |

| **SURNAME** | **NAME** | | **TITLE** | **NATIONALITY** | **GENDER** | **PUB_YEAR** | **CONTINENT** | **DATE OF BIRTH** | **TRANSLATED** | **NOBEL** | **NEUSTADT** | **BOOKER** | **PULITZER** | **NEBULA** | **CAINE** | **FRANZ KAFKA** | **NBA** | **BBA** | **WOMEN'S PRIZE FOR FICTION** | **NEW YORK TIMES** | **THE GUARDIAN** | **THE NEW YORKER** | **BBC** | **ATLANTIC** |
| --- | --- | --- | --- | --- | --- | --- | --- | --- | --- | --- | --- | --- | --- | --- | --- | --- | --- | --- | --- | --- | --- | --- | --- | --- |
| El Hachmi | Najat | The last patriarch | | MOROCCO | 1 | 2008 | 5 | 1979 | 1 | 0 | 0 | 0 | 0 | 0 | 0 | 0 | 0 | 0 | 0 | 0 | 0 | 0 | 0 | 0 |
| El Rashidi | Yasmine | Chronicle of a Last Summer | | EGYPT | 1 | 2016 | 5 | 1977 | 0 | 0 | 0 | 0 | 0 | 0 | 0 | 0 | 0 | 0 | 0 | 0 | 0 | 0 | 0 | 0 |
| Eliot | George | Middlemarch | | UK | 1 | 1871 | 1 | 1819 | 0 | 0 | 0 | 0 | 0 | 0 | 0 | 0 | 0 | 0 | 0 | 0 | 0 | 0 | 1 | 0 |
| Eliot | George | Adam Bede | | UK | 1 | 1859 | 1 | 1819 | 0 | 0 | 0 | 0 | 0 | 0 | 0 | 0 | 0 | 0 | 0 | 0 | 0 | 0 | 0 | 0 |
| Eliot | George | Daniel Deronda | | UK | 1 | 1876 | 1 | 1819 | 0 | 0 | 0 | 0 | 0 | 0 | 0 | 0 | 0 | 0 | 0 | 0 | 0 | 0 | 0 | 0 |
| Eliot | George | Romola | | UK | 1 | 1863 | 1 | 1819 | 0 | 0 | 0 | 0 | 0 | 0 | 0 | 0 | 0 | 0 | 0 | 0 | 0 | 0 | 0 | 0 |
| Eliot | George | Silas Marner | | UK | 1 | 1861 | 1 | 1819 | 0 | 0 | 0 | 0 | 0 | 0 | 0 | 0 | 0 | 0 | 0 | 0 | 0 | 0 | 0 | 0 |
| Eliot | George | The Mill on the Floss | | UK | 1 | 1860 | 1 | 1819 | 0 | 0 | 0 | 0 | 0 | 0 | 0 | 0 | 0 | 0 | 0 | 0 | 0 | 0 | 0 | 0 |
| Eliot | George | Felix Holt the Radical | | UK | 1 | 1866 | 1 | 1819 | 0 | 0 | 0 | 0 | 0 | 0 | 0 | 0 | 0 | 0 | 0 | 0 | 0 | 0 | 0 | 0 |
| Ellis | Bret Easton | Less than zero | | USA | 0 | 1985 | 2 | 1964 | 0 | 0 | 0 | 0 | 0 | 0 | 0 | 0 | 0 | 0 | 0 | 0 | 0 | 0 | 0 | 0 |
| Ellis | Bret Easton | American psycho | | USA | 0 | 1991 | 2 | 1964 | 0 | 0 | 0 | 0 | 0 | 0 | 0 | 0 | 0 | 0 | 0 | 0 | 0 | 0 | 0 | 0 |
| Ellison | Ralph | Invisible Man | | USA | 0 | 1952 | 2 | 1914 | 0 | 0 | 0 | 0 | 0 | 0 | 0 | 0 | 1 | 0 | 0 | 0 | 0 | 0 | 0 | 0 |
| Ellmann | Lucy | Ducks, Newburyport | | USA | 1 | 2019 | 2 | 1956 | 0 | 0 | 0 | 0 | 0 | 0 | 0 | 0 | 0 | 0 | 0 | 0 | 1 | 1 | 0 | 0 |
| Elsir | Amir Tag | Telepathy | | SUDAN | 0 | 2016 | 5 | 1960 | 1 | 0 | 0 | 0 | 0 | 0 | 0 | 0 | 0 | 0 | 0 | 0 | 0 | 0 | 0 | 0 |
| Eltayeb | Tarek | The Palm House | | SUDAN | 0 | 2012 | 5 | 1959 | 1 | 0 | 0 | 0 | 0 | 0 | 0 | 0 | 0 | 0 | 0 | 0 | 0 | 0 | 0 | 0 |
| Emecheta | Buchi | Second Class Citizen | | NIGERIA | 1 | 1974 | 5 | 1944 | 0 | 0 | 0 | 0 | 0 | 0 | 0 | 0 | 0 | 0 | 0 | 0 | 0 | 0 | 0 | 0 |
| Emezi | Akwaeke | Freshwater | | NIGERIA | 1 | 2018 | 5 | 1987 | 0 | 0 | 0 | 0 | 0 | 0 | 0 | 0 | 0 | 0 | 0 | 0 | 0 | 1 | 0 | 0 |
| Ende | Michael | The Neverending Story | | GERMANY | 0 | 1979 | 1 | 1929 | 1 | 0 | 0 | 0 | 0 | 0 | 0 | 0 | 0 | 0 | 0 | 0 | 0 | 0 | 0 | 0 |
| Ende | Michael | Momo | | GERMANY | 0 | 1973 | 1 | 1929 | 1 | 0 | 0 | 0 | 0 | 0 | 0 | 0 | 0 | 0 | 0 | 0 | 0 | 0 | 0 | 0 |
| Endō | Shūsaku | Silence | | JAPAN | 0 | 1966 | 4 | 1923 | 1 | 0 | 0 | 0 | 0 | 0 | 0 | 0 | 0 | 0 | 0 | 0 | 0 | 0 | 0 | 0 |
| Enright | Anne | The Gathering | | IRELAND | 1 | 2007 | 1 | 1962 | 0 | 0 | 0 | 1 | 0 | 0 | 0 | 0 | 0 | 0 | 0 | 0 | 0 | 0 | 0 | 0 |
| Enrigue | Alvaro | Sudden death | | MEXICO | 0 | 2016 | 3 | 1969 | 1 | 0 | 0 | 0 | 0 | 0 | 0 | 0 | 0 | 0 | 0 | 0 | 1 | 0 | 0 | 0 |
| Enríquez | Mariana | Things we lost in the fire | | ARGENTINA | 1 | 2016 | 3 | 1973 | 1 | 0 | 0 | 0 | 0 | 0 | 0 | 0 | 0 | 0 | 0 | 0 | 0 | 0 | 0 | 0 |
| Erdrich | Louise | The Night Watchman | | USA | 1 | 2020 | 2 | 1954 | 0 | 0 | 0 | 0 | 1 | 0 | 0 | 0 | 0 | 0 | 0 | 0 | 0 | 0 | 1 | 0 |
| Ernaux | Annie | A woman's story | | FRANCE | 1 | 1988 | 1 | 1940 | 1 | 0 | 0 | 0 | 0 | 0 | 0 | 0 | 0 | 0 | 0 | 0 | 0 | 0 | 0 | 0 |
| Erpenbeck | Jenny | Visitation | | GERMANY | 1 | 2008 | 1 | 1967 | 1 | 0 | 0 | 0 | 0 | 0 | 0 | 0 | 0 | 0 | 0 | 0 | 1 | 0 | 0 | 0 |
| Escobar | Melba | House of beauty | | COLOMBIA | 1 | 2015 | 3 | 1976 | 1 | 0 | 0 | 0 | 0 | 0 | 0 | 0 | 0 | 0 | 0 | 0 | 0 | 0 | 0 | 0 |
| Esquivel | Laura | Pierced by the sun | | MEXICO | 1 | 2014 | 3 | 1950 | 1 | 0 | 0 | 0 | 0 | 0 | 0 | 0 | 0 | 0 | 0 | 0 | 0 | 0 | 0 | 0 |
| Eteraz | Ali | Children of dust | | PAKISTAN | 0 | 2009 | 4 | 1980 | 0 | 0 | 0 | 0 | 0 | 0 | 0 | 0 | 0 | 0 | 0 | 0 | 0 | 0 | 0 | 0 |
| Eugenides | Jeffrey | Middlesex | | USA | 0 | 2002 | 2 | 1960 | 0 | 0 | 0 | 0 | 1 | 0 | 0 | 0 | 0 | 0 | 0 | 0 | 0 | 0 | 0 | 0 |
| Eugenides | Jeffrey | The virgin suicides | | USA | 0 | 1993 | 2 | 1960 | 0 | 0 | 0 | 0 | 0 | 0 | 0 | 0 | 0 | 0 | 0 | 0 | 0 | 0 | 0 | 0 |
| Evans | Nicholas | The Horse Whisperer | | UK | 0 | 1995 | 1 | 1950 | 0 | 0 | 0 | 0 | 0 | 0 | 0 | 0 | 0 | 0 | 0 | 0 | 0 | 0 | 0 | 0 |
| Evans | Nicholas | The loop | | UK | 0 | 1998 | 1 | 1950 | 0 | 0 | 0 | 0 | 0 | 0 | 0 | 0 | 0 | 0 | 0 | 0 | 0 | 0 | 0 | 0 |
| Evaristo | Bernardine | Girl, Woman, Other | | UK | 1 | 2019 | 1 | 1959 | 0 | 0 | 0 | 1 | 0 | 0 | 0 | 0 | 0 | 1 | 0 | 0 | 1 | 1 | 1 | 0 |
| Faber | Michael | The Crimson Petal and the White | | NETHERLANDS | 0 | 2002 | 1 | 1960 | 0 | 0 | 0 | 0 | 0 | 0 | 0 | 0 | 0 | 0 | 0 | 0 | 0 | 0 | 0 | 0 |
| Fallaci | Oriana | Letter to a Child Never Born | | ITALY | 1 | 1975 | 1 | 1929 | 1 | 0 | 0 | 0 | 0 | 0 | 0 | 0 | 0 | 0 | 0 | 0 | 0 | 0 | 0 | 0 |
| Fante | John | Ask the dust | | USA | 0 | 1939 | 2 | 1909 | 0 | 0 | 0 | 0 | 0 | 0 | 0 | 0 | 0 | 0 | 0 | 0 | 0 | 0 | 0 | 1 |
| Fante | John | The brotherhood of the grape | | USA | 0 | 1977 | 2 | 1909 | 0 | 0 | 0 | 0 | 0 | 0 | 0 | 0 | 0 | 0 | 0 | 0 | 0 | 0 | 0 | 0 |
| Fante | John | Wait until spring Bandini | | USA | 0 | 1938 | 2 | 1909 | 0 | 0 | 0 | 0 | 0 | 0 | 0 | 0 | 0 | 0 | 0 | 0 | 0 | 0 | 0 | 0 |
| Farah | Nuruddin | From a Crooked Rib | | SOMALIA | 0 | 1970 | 5 | 1945 | 0 | 0 | 1 | 0 | 0 | 0 | 0 | 0 | 0 | 0 | 0 | 0 | 0 | 0 | 0 | 0 |
| Farah | Nuruddin | Maps | | SOMALIA | 0 | 1986 | 5 | 1945 | 0 | 0 | 1 | 0 | 0 | 0 | 0 | 0 | 0 | 0 | 0 | 0 | 0 | 0 | 0 | 0 |
| Farah | Nuruddin | North of Dawn | | SOMALIA | 0 | 2018 | 5 | 1945 | 0 | 0 | 1 | 0 | 0 | 0 | 0 | 0 | 0 | 0 | 0 | 0 | 0 | 0 | 0 | 0 |
| Farrell | James Gordon | The Siege of Krishnapur | | UK | 0 | 1973 | 1 | 1935 | 0 | 0 | 0 | 1 | 0 | 0 | 0 | 0 | 0 | 0 | 0 | 0 | 0 | 0 | 0 | 0 |
| Farrell | James Gordon | Troubles | | UK | 0 | 1970 | 1 | 1935 | 0 | 0 | 0 | 1 | 0 | 0 | 0 | 0 | 0 | 0 | 0 | 0 | 0 | 0 | 0 | 0 |
| Faulkner | William | The sound and the fury | | USA | 0 | 1929 | 2 | 1897 | 0 | 1 | 0 | 0 | 0 | 0 | 0 | 0 | 0 | 0 | 0 | 0 | 0 | 0 | 0 | 0 |
| Faulkner | William | As I lay dying | | USA | 0 | 1930 | 2 | 1897 | 0 | 1 | 0 | 0 | 0 | 0 | 0 | 0 | 0 | 0 | 0 | 0 | 0 | 0 | 0 | 0 |
| Faulkner | William | Sanctuary | | USA | 0 | 1931 | 2 | 1897 | 0 | 1 | 0 | 0 | 0 | 0 | 0 | 0 | 0 | 0 | 0 | 0 | 0 | 0 | 0 | 0 |
| Faulkner | William | A Fable | | USA | 0 | 1954 | 2 | 1897 | 0 | 1 | 0 | 0 | 1 | 0 | 0 | 0 | 1 | 0 | 0 | 0 | 0 | 0 | 0 | 0 |
| Faye | Gaël | Small Country | | BURUNDI | 0 | 2016 | 5 | 1982 | 1 | 0 | 0 | 0 | 0 | 0 | 0 | 0 | 0 | 0 | 0 | 0 | 0 | 0 | 0 | 0 |
| Feiyu | Bi | Three sisters | | CHINA | 0 | 2002 | 4 | 1964 | 1 | 0 | 0 | 0 | 0 | 0 | 0 | 0 | 0 | 0 | 0 | 0 | 0 | 0 | 0 | 0 |
| Ferber | Edna | So Big | | USA | 1 | 1924 | 2 | 1885 | 0 | 0 | 0 | 0 | 1 | 0 | 0 | 0 | 0 | 0 | 0 | 0 | 0 | 0 | 0 | 0 |
| Ferguson | Will | Hitching Rides With Buddha | | CANADA | 0 | 1998 | 2 | 1964 | 0 | 0 | 0 | 0 | 0 | 0 | 0 | 0 | 0 | 0 | 0 | 0 | 0 | 0 | 0 | 0 |
| Ferrante | Elena | Troubling love | | ITALY | 1 | 1995 | 1 | 1953 | 1 | 0 | 0 | 0 | 0 | 0 | 0 | 0 | 0 | 0 | 0 | 0 | 0 | 1 | 0 | 0 |
| Ferrante | Elena | The days of abandonment | | ITALY | 1 | 2002 | 1 | 1953 | 1 | 0 | 0 | 0 | 0 | 0 | 0 | 0 | 0 | 0 | 0 | 0 | 0 | 1 | 0 | 0 |
| Ferrante | Elena | The lost daughter | | ITALY | 1 | 2006 | 1 | 1953 | 1 | 0 | 0 | 0 | 0 | 0 | 0 | 0 | 0 | 0 | 0 | 0 | 0 | 1 | 0 | 0 |
| Ferrante | Elena | My brilliant friend | | ITALY | 1 | 2011 | 1 | 1953 | 1 | 0 | 0 | 0 | 0 | 0 | 0 | 0 | 0 | 0 | 0 | 0 | 0 | 1 | 0 | 0 |
| Ferrante | Elena | The story of a new name | | ITALY | 1 | 2012 | 1 | 1953 | 1 | 0 | 0 | 0 | 0 | 0 | 0 | 0 | 0 | 0 | 0 | 0 | 0 | 1 | 0 | 0 |
| Ferrante | Elena | Those who leave and those who stay | | ITALY | 1 | 2013 | 1 | 1953 | 1 | 0 | 0 | 0 | 0 | 0 | 0 | 0 | 0 | 0 | 0 | 0 | 1 | 1 | 0 | 0 |
| Ferrante | Elena | The story of the lost child | | ITALY | 1 | 2006 | 1 | 1953 | 1 | 0 | 0 | 0 | 0 | 0 | 0 | 0 | 0 | 0 | 0 | 1 | 1 | 1 | 1 | 0 |
| Ferris | Joshua | To Rise Again at a Decent Hour | | USA | 0 | 2014 | 2 | 1974 | 0 | 0 | 0 | 0 | 0 | 0 | 0 | 0 | 0 | 0 | 0 | 0 | 0 | 0 | 0 | 0 |
| Fielding | Helen | Bridget Jones's diary | | UK | 1 | 1995 | 1 | 1958 | 0 | 0 | 0 | 0 | 0 | 0 | 0 | 0 | 0 | 1 | 0 | 0 | 0 | 0 | 1 | 0 |
| Fielding | Henry | The history of Tom Jones | | UK | 0 | 1749 | 1 | 1707 | 0 | 0 | 0 | 0 | 0 | 0 | 0 | 0 | 0 | 0 | 0 | 0 | 0 | 0 | 0 | 0 |
| Fielding | Henry | A Journey from this World to the Next | | UK | 0 | 1749 | 1 | 1707 | 0 | 0 | 0 | 0 | 0 | 0 | 0 | 0 | 0 | 0 | 0 | 0 | 0 | 0 | 0 | 0 |
| Fielding | Henry | Amelia | | UK | 0 | 1751 | 1 | 1707 | 0 | 0 | 0 | 0 | 0 | 0 | 0 | 0 | 0 | 0 | 0 | 0 | 0 | 0 | 0 | 0 |
| Fielding | Henry | pology for the Life of Mrs. Shamela And | | UK | 0 | 1741 | 1 | 1707 | 0 | 0 | 0 | 0 | 0 | 0 | 0 | 0 | 0 | 0 | 0 | 0 | 0 | 0 | 0 | 0 |
| Fielding | Henry | Life and Death of Jonathan Wild the G | | UK | 0 | 1743 | 1 | 1707 | 0 | 0 | 0 | 0 | 0 | 0 | 0 | 0 | 0 | 0 | 0 | 0 | 0 | 0 | 0 | 0 |
| Fischer | Tibor | Under the Frog | | UK | 0 | 1992 | 1 | 1959 | 0 | 0 | 0 | 0 | 0 | 0 | 0 | 0 | 0 | 0 | 0 | 0 | 0 | 0 | 0 | 0 |
| Fitch | Janet | White Oleander | | USA | 1 | 1999 | 2 | 1955 | 0 | 0 | 0 | 0 | 0 | 0 | 0 | 0 | 0 | 0 | 0 | 0 | 0 | 0 | 0 | 0 |
| Fitzgerald | Francis Scott | Tender is the night | | USA | 0 | 1934 | 2 | 1896 | 0 | 0 | 0 | 0 | 0 | 0 | 0 | 0 | 0 | 0 | 0 | 0 | 0 | 0 | 0 | 0 |
| Fitzgerald | Francis Scott | The great Gatsby | | USA | 0 | 1925 | 2 | 1896 | 0 | 0 | 0 | 0 | 0 | 0 | 0 | 0 | 0 | 0 | 0 | 0 | 0 | 0 | 1 | 0 |
| Fitzgerald | Penelope | Offshore | | UK | 1 | 1979 | 1 | 1916 | 0 | 0 | 0 | 1 | 0 | 0 | 0 | 0 | 0 | 0 | 0 | 0 | 0 | 0 | 0 | 0 |
| Fitzgerald | Penelope | The Bookshop | | UK | 1 | 1978 | 1 | 1916 | 0 | 0 | 0 | 0 | 0 | 0 | 0 | 0 | 0 | 0 | 0 | 0 | 0 | 0 | 0 | 0 |
| Flagg | Fannie | ed green tomatoes at the Whistle stop c | | USA | 1 | 1987 | 2 | 1944 | 0 | 0 | 0 | 0 | 0 | 0 | 0 | 0 | 0 | 0 | 0 | 0 | 0 | 0 | 0 | 0 |
| Flanagan | Richard | First person | | AUSTRALIA | 0 | 2017 | 6 | 1961 | 0 | 0 | 0 | 0 | 0 | 0 | 0 | 0 | 0 | 0 | 0 | 0 | 0 | 0 | 0 | 0 |
| Flanagan | Richard | Gould's book of fish | | AUSTRALIA | 0 | 2001 | 6 | 1961 | 0 | 0 | 0 | 0 | 0 | 0 | 0 | 0 | 0 | 0 | 0 | 0 | 0 | 0 | 0 | 0 |
| Flanagan | Richard | Death of a river guide | | AUSTRALIA | 0 | 1994 | 6 | 1961 | 0 | 0 | 0 | 0 | 0 | 0 | 0 | 0 | 0 | 0 | 0 | 0 | 0 | 0 | 0 | 0 |
| Flanagan | Richard | The Narrow Road to the Deep North | | AUSTRALIA | 0 | 2013 | 6 | 1961 | 0 | 0 | 0 | 1 | 0 | 0 | 0 | 0 | 0 | 0 | 0 | 0 | 1 | 0 | 0 | 0 |
| Flaubert | Gustave | Madame Bovary | | FRANCE | 0 | 1856 | 1 | 1821 | 1 | 0 | 0 | 0 | 0 | 0 | 0 | 0 | 0 | 0 | 0 | 0 | 1 | 0 | 0 | 0 |
| Flaubert | Gustave | Sentimental education | | FRANCE | 0 | 1869 | 1 | 1821 | 1 | 0 | 0 | 0 | 0 | 0 | 0 | 0 | 0 | 0 | 0 | 0 | 0 | 0 | 0 | 0 |
| Flavin | Martin | Journey in the Dark | | USA | 0 | 1943 | 2 | 1883 | 0 | 0 | 0 | 0 | 1 | 0 | 0 | 0 | 0 | 0 | 0 | 0 | 0 | 0 | 0 | 0 |
| Flynn | Gillian | Gone girl | | USA | 1 | 2012 | 2 | 1971 | 0 | 0 | 0 | 0 | 0 | 0 | 0 | 0 | 0 | 1 | 0 | 0 | 0 | 0 | 0 | 0 |
| Flynn | Gillian | Sharp objects | | USA | 1 | 2006 | 2 | 1971 | 0 | 0 | 0 | 0 | 0 | 0 | 0 | 0 | 0 | 0 | 0 | 0 | 0 | 0 | 0 | 0 |
| Foer  Foer | onathan Safran Everything is illuminated  onathan Safran Extremely loud and incredibly close | | | USA  USA | 0  0 | 2002  2005 | 2  2 | 1977  1977 | 0  0 | 0  0 | 0  0 | 0  0 | 0  0 | 0  0 | 0  0 | 0  0 | 0  0 | 0  0 | 0  0 | 0  0 | 0  0 | 0  0 | 0  0 | 0  0 |
| Fogazzaro | Antonio | The patriot | | ITALY | 0 | 1895 | 1 | 1842 | 1 | 0 | 0 | 0 | 0 | 0 | 0 | 0 | 0 | 0 | 0 | 0 | 0 | 0 | 0 | 0 |
| Follett | Ken | Fall of giants | | UK | 0 | 2010 | 1 | 1949 | 0 | 0 | 0 | 0 | 0 | 0 | 0 | 0 | 0 | 0 | 0 | 0 | 0 | 0 | 0 | 0 |
| Follett | Ken | Edge of eternity | | UK | 0 | 2014 | 1 | 1949 | 0 | 0 | 0 | 0 | 0 | 0 | 0 | 0 | 0 | 0 | 0 | 0 | 0 | 0 | 0 | 0 |

| **SURNAME** | | **NAME** | **TITLE** | | **NATIONALITY** | **GENDER** | **PUB_YEAR** | **CONTINENT** | **DATE OF BIRTH** | **TRANSLATED** | **NOBEL** | **NEUSTADT** | **BOOKER** | **PULITZER** | **NEBULA** | **CAINE** | **FRANZ KAFKA** | **NBA** | **BBA** | **WOMEN'S PRIZE FOR FICTION** | **NEW YORK TIMES** | **THE GUARDIAN** | **THE NEW YORKER** | **BBC** | **ATLANTIC** |
| --- | --- | --- | --- | --- | --- | --- | --- | --- | --- | --- | --- | --- | --- | --- | --- | --- | --- | --- | --- | --- | --- | --- | --- | --- | --- |
| Follett  Follett | Ken  Ken | | | Winter of the world  The Pillars of the Earth | UK  UK | 0  0 | 2012  1989 | 1  1 | 1949  1949 | 0  0 | 0  0 | 0  0 | 0  0 | 0  0 | 0  0 | 0  0 | 0  0 | 0  0 | 0  0 | 0  0 | 0  0 | 0  0 | 0  0 | 0  1 | 0  0 |
| Fonseca | Rubem | | | Winning the Game | BRAZIL | 0 | 2013 | 3 | 1925 | 1 | 0 | 0 | 0 | 0 | 0 | 0 | 0 | 0 | 0 | 0 | 0 | 0 | 0 | 0 | 0 |
| Fontane | Theodor | | | Effi Briest | GERMANY | 0 | 1895 | 1 | 1819 | 1 | 0 | 0 | 0 | 0 | 0 | 0 | 0 | 0 | 0 | 0 | 0 | 0 | 0 | 0 | 0 |
| Ford  Ford | Richard  Richard | | | Canada  Independence Day | USA  USA | 0  0 | 2012  1995 | 2  2 | 1944  1944 | 0  0 | 0  0 | 0  0 | 0  0 | 0  1 | 0  0 | 0  0 | 0  0 | 0  0 | 0  0 | 0  0 | 0  0 | 0  0 | 0  0 | 0  0 | 0  0 |
| Forna Forna  Forna | Aminatta Aminatta  Aminatta | | | The Devil that Danced on the Water The Hired Man  The Memory of Love | SIERRA LEONE SIERRA LEONE  SIERRA LEONE | 1  1  1 | 2003  2013  2010 | 5  5  5 | 1964  1964  1964 | 0  0  0 | 0  0  0 | 0  0  0 | 0  0  0 | 0  0  0 | 0  0  0 | 0  0  0 | 0  0  0 | 0  0  0 | 0  0  0 | 0  0  0 | 0  0  0 | 0  0  0 | 0  0  0 | 0  0  0 | 0  0  0 |
| Foroutan | Parnaz | | | The girl from the garden | IRAN | 1 | 2015 | 4 | ? | 0 | 0 | 0 | 0 | 0 | 0 | 0 | 0 | 0 | 0 | 0 | 0 | 0 | 0 | 0 | 0 |
| Forster Forster Forster Forster  Forster | Edward Morgan Edward Morgan Edward Morgan Edward Morgan  Edward Morgan | | | A Passage to India Howards End Maurice  Room with a View  Where Angels Fear to Tread | UK UK UK UK  UK | 0  0  0  0  0 | 1924  1910  1971  1908  1905 | 1  1  1  1  1 | 1879  1879  1879  1879  1879 | 0  0  0  0  0 | 0  0  0  0  0 | 0  0  0  0  0 | 0  0  0  0  0 | 0  0  0  0  0 | 0  0  0  0  0 | 0  0  0  0  0 | 0  0  0  0  0 | 0  0  0  0  0 | 0  0  0  0  0 | 0  0  0  0  0 | 0  0  0  0  0 | 0  0  0  0  0 | 0  0  0  0  0 | 0  0  0  0  0 | 0  0  0  0  0 |
| Forsyth  Forsyth | Frederick  Frederick | | | The Odessa file  No Comebacks | UK  UK | 0  0 | 1972  1983 | 1  1 | 1938  1938 | 0  0 | 0  0 | 0  0 | 0  0 | 0  0 | 0  0 | 0  0 | 0  0 | 0  0 | 0  0 | 0  0 | 0  0 | 0  0 | 0  0 | 0  0 | 0  0 |
| Foulds | Adam | | | The Quickening Maze | UK | 0 | 2009 | 1 | 1974 | 0 | 0 | 0 | 0 | 0 | 0 | 0 | 0 | 0 | 0 | 0 | 0 | 0 | 0 | 0 | 0 |
| Fowler Karen We Are All Completely Beside Ourselve USA 1 2014 2 1950 0 0 0 0 0 0 0 0 0 1 0 0 0 0 0 0 | | | | | | | | | | | | | | | | | | | | | | | | | |
| Fowles  Fowles | John  John | | | Daniel Martin  The Magus | UK  UK | 0  0 | 1977  1965 | 1  1 | 1926  1926 | 0  0 | 0  0 | 0  0 | 0  0 | 0  0 | 0  0 | 0  0 | 0  0 | 0  0 | 0  0 | 0  0 | 0  0 | 0  0 | 0  0 | 0  1 | 0  0 |
| Fraillon | Zana | | | The bone sparrow | AUSTRALIA | 1 | 2016 | 6 | 1981 | 0 | 0 | 0 | 0 | 0 | 0 | 0 | 0 | 0 | 0 | 0 | 0 | 0 | 0 | 0 | 0 |
| Frame  Frame | Janet  Janet | | | Towards another summer  Owls do cry | NEW ZEALAND  NEW ZEALAND | 1  1 | 2007  1957 | 6  6 | 1924  1924 | 0  0 | 0  0 | 0  0 | 0  0 | 0  0 | 0  0 | 0  0 | 0  0 | 0  0 | 0  0 | 0  0 | 0  0 | 0  0 | 0  0 | 0  0 | 0  0 |
| France  France | Anatole  Anatole | | | Penguin island  Thaïs | FRANCE  FRANCE | 0  0 | 1908  1890 | 1  1 | 1844  1844 | 1  1 | 1  1 | 0  0 | 0  0 | 0  0 | 0  0 | 0  0 | 0  0 | 0  0 | 0  0 | 0  0 | 0  0 | 0  0 | 0  0 | 0  0 | 0  0 |
| Frank | Anne | | | The diary of Anne Frank | NETHERLANDS | 1 | 1947 | 1 | 1929 | 1 | 0 | 0 | 0 | 0 | 0 | 0 | 0 | 0 | 0 | 0 | 0 | 0 | 0 | 0 | 0 |
| Franzen  Franzen Franzen | Jonathan  Jonathan Jonathan | | | Purity  The corrections Freedom | USA  USA USA | 0  0  0 | 2015  2001  2010 | 2  2  2 | 1959  1959  1959 | 0  0  0 | 0  0  0 | 0  0  0 | 0  0  0 | 0  0  0 | 0  0  0 | 0  0  0 | 0  0  0 | 0  1  0 | 0  0  1 | 0  0  0 | 0  0  1 | 1  0  1 | 0  0  0 | 0  0  0 | 0  0  0 |
| Frayn | Michael | | | Headlong | UK | 0 | 1999 | 1 | 1933 | 0 | 0 | 0 | 0 | 0 | 0 | 0 | 0 | 0 | 0 | 0 | 0 | 0 | 0 | 0 | 0 |
| Freeman  Freeman | Ru  Ru | | | A Disobedient Girl  On Sal Mal Lane | SRI LANKA  SRI LANKA | 1  1 | 2009  2013 | 4  4 | 1967  1967 | 0  0 | 0  0 | 0  0 | 0  0 | 0  0 | 0  0 | 0  0 | 0  0 | 0  0 | 0  0 | 0  0 | 0  0 | 0  0 | 0  0 | 0  0 | 0  0 |
| French | Marilyn | | | The Women's Room | USA | 1 | 1977 | 2 | 1929 | 0 | 0 | 0 | 0 | 0 | 0 | 0 | 0 | 0 | 0 | 0 | 0 | 0 | 0 | 0 | 0 |
| Frey  Frey | James  James | | | A Million Little Pieces  My Friend Leonard | USA  USA | 0  0 | 2003  2005 | 2  2 | 1969  1969 | 0  0 | 0  0 | 0  0 | 0  0 | 0  0 | 0  0 | 0  0 | 0  0 | 0  0 | 0  0 | 0  0 | 0  0 | 0  0 | 0  0 | 0  0 | 0  0 |
| Fridlund | Emily | | | History of Wolves | USA | 1 | 2017 | 2 | 1979 | 0 | 0 | 0 | 0 | 0 | 0 | 0 | 0 | 0 | 0 | 0 | 0 | 0 | 0 | 0 | 0 |
| Frisch | Max | | | I'm Not Stiller | SWITZERLAND | 0 | 1954 | 1 | 1911 | 1 | 0 | 1 | 0 | 0 | 0 | 0 | 0 | 0 | 0 | 0 | 0 | 0 | 0 | 0 | 0 |
| Fuentes Fuentes  Fuentes | Carlos Carlos  Carlos | | | The old gringo Destiny and desire  The Death of Artemio Cruz | MEXICO MEXICO  MEXICO | 0  0  0 | 1985  2008  1962 | 3  3  3 | 1928  1928  1928 | 1  1  1 | 0  0  0 | 0  0  0 | 0  0  0 | 0  0  0 | 0  0  0 | 0  0  0 | 0  0  0 | 0  0  0 | 0  0  0 | 0  0  0 | 0  0  0 | 0  0  0 | 0  0  0 | 0  0  0 | 0  0  0 |
| Fuller | John | | | Flying to Nowhere | UK | 0 | 1983 | 1 | 1937 | 0 | 0 | 0 | 0 | 0 | 0 | 0 | 0 | 0 | 0 | 0 | 0 | 0 | 0 | 0 | 0 |
| Gaarder  Gaarder | Jostein  Jostein | | | Sophie's World  The Orange Girl | NORWAY  NORWAY | 0  0 | 1991  2003 | 1  1 | 1952  1952 | 1  1 | 0  0 | 0  0 | 0  0 | 0  0 | 0  0 | 0  0 | 0  0 | 0  0 | 0  0 | 0  0 | 0  0 | 0  0 | 0  0 | 0  0 | 0  0 |
| Gaddis | William | | | J R | USA | 0 | 1975 | 2 | 1922 | 0 | 0 | 0 | 0 | 0 | 0 | 0 | 0 | 1 | 0 | 0 | 0 | 0 | 0 | 0 | 0 |
| Gaiman | Neil | | | Fragile things | UK | 0 | 2006 | 1 | 1960 | 0 | 0 | 0 | 0 | 0 | 0 | 0 | 0 | 0 | 0 | 0 | 0 | 0 | 0 | 0 | 0 |
| Gaines | Ernest J. | | | A Lesson Before Dying | USA | 0 | 1993 | 2 | 1933 | 0 | 0 | 0 | 0 | 0 | 0 | 0 | 0 | 0 | 0 | 0 | 0 | 0 | 0 | 0 | 0 |
| Galdos | Benito Perez | | | Tristana | SPAIN | 0 | 1897 | 1 | 1843 | 1 | 0 | 0 | 0 | 0 | 0 | 0 | 0 | 0 | 0 | 0 | 0 | 0 | 0 | 0 | 0 |
| Galgut | Damon | | | The Good Doctor | SOUTH AFRICA | 0 | 2003 | 5 | 1963 | 0 | 0 | 0 | 0 | 0 | 0 | 0 | 0 | 0 | 0 | 0 | 0 | 0 | 0 | 0 | 0 |
| Gallmann | Kuki | | | I dreamed of Africa | KENYA | 1 | 1991 | 5 | 1943 | 0 | 0 | 0 | 0 | 0 | 0 | 0 | 0 | 0 | 0 | 0 | 0 | 0 | 0 | 0 | 0 |
| Galsworthy | John | | | The Forsyte Saga | UK | 0 | 1922 | 1 | 1867 | 0 | 1 | 0 | 0 | 0 | 0 | 0 | 0 | 0 | 0 | 0 | 0 | 0 | 0 | 0 | 0 |
| Gamboa | Santiago | | | Return to the Dark Valley | COLOMBIA | 0 | 2016 | 3 | 1965 | 1 | 0 | 0 | 0 | 0 | 0 | 0 | 0 | 0 | 0 | 0 | 0 | 0 | 0 | 0 | 0 |
| Gappah  Gappah | Petina  Petina | | | The book of Memory  An Elegy for Easterly | ZIMBABWE  ZIMBABWE | 1  1 | 2015  2009 | 5  5 | 1971  1971 | 0  0 | 0  0 | 0  0 | 0  0 | 0  0 | 0  0 | 0  0 | 0  0 | 0  0 | 0  0 | 0  0 | 0  0 | 0  0 | 0  0 | 0  0 | 0  0 |
| Gardam | Jane | | | God on the Rocks | UK | 1 | 1978 | 1 | 1928 | 0 | 0 | 0 | 0 | 0 | 0 | 0 | 0 | 0 | 0 | 0 | 0 | 0 | 0 | 0 | 0 |
| Gardner  Gardner | Erle Stanley  Erle Stanley | | | The Case of the Velvet Claws  The Case of the Sulky Girl | USA  USA | 0  0 | 1933  1933 | 2  2 | 1889  1889 | 0  0 | 0  0 | 0  0 | 0  0 | 0  0 | 0  0 | 0  0 | 0  0 | 0  0 | 0  0 | 0  0 | 0  0 | 0  0 | 0  0 | 0  0 | 0  0 |
| Gargash  Gargash | Maha  Maha | | | That Other Me: A Novel  The Sand Fish | UAE  UAE | 1  1 | 2016  2009 | 4  4 | ?  ? | 0  0 | 0  0 | 0  0 | 0  0 | 0  0 | 0  0 | 0  0 | 0  0 | 0  0 | 0  0 | 0  0 | 0  0 | 0  0 | 0  0 | 0  0 | 0  0 |
| Gary | Romain | | | The kites | FRANCE | 0 | 1980 | 1 | 1914 | 1 | 0 | 0 | 0 | 0 | 0 | 0 | 0 | 0 | 0 | 0 | 0 | 0 | 0 | 0 | 0 |
| Gaskell Gaskell Gaskell Gaskell  Gaskell | Elizabeth Elizabeth Elizabeth Elizabeth  Elizabeth | | | Life of Charlotte Bronte Mary Barton  North and South Wives and Daughters  Sylvia's Lovers | UK UK UK UK  UK | 1  1  1  1  1 | 1857  1848  1854  1866  1863 | 1  1  1  1  1 | 1810  1810  1810  1810  1810 | 0  0  0  0  0 | 0  0  0  0  0 | 0  0  0  0  0 | 0  0  0  0  0 | 0  0  0  0  0 | 0  0  0  0  0 | 0  0  0  0  0 | 0  0  0  0  0 | 0  0  0  0  0 | 0  0  0  0  0 | 0  0  0  0  0 | 0  0  0  0  0 | 0  0  0  0  0 | 0  0  0  0  0 | 0  0  0  0  0 | 0  0  0  0  0 |
| Genet Genet Genet  Genet | Jean Jean Jean  Jean | | | Miracle of the Rose Our Lady of the Flowers  Querelle of Brest  The Thief's Journal | FRANCE FRANCE FRANCE  FRANCE | 0  0  0  0 | 1946  1943  1947  1949 | 1  1  1  1 | 1910  1910  1910  1910 | 1  1  1  1 | 0  0  0  0 | 0  0  0  0 | 0  0  0  0 | 0  0  0  0 | 0  0  0  0 | 0  0  0  0 | 0  0  0  0 | 0  0  0  0 | 0  0  0  0 | 0  0  0  0 | 0  0  0  0 | 0  0  0  0 | 0  0  0  0 | 0  0  0  0 | 0  0  0  0 |
| Ghosh Ghosh Ghosh Ghosh  Ghosh | Amitav Amitav Amitav Amitav  Amitav | | | Sea of poppies Calcutta chromosome Flood of fire  River of Smoke  Gun island | INDIA INDIA INDIA INDIA  INDIA | 0  0  0  0  0 | 2008  1995  2012  2011  2019 | 4  4  4  4  4 | 1956  1956  1956  1956  1956 | 0  0  0  0  0 | 0  0  0  0  0 | 0  0  0  0  0 | 0  0  0  0  0 | 0  0  0  0  0 | 0  0  0  0  0 | 0  0  0  0  0 | 0  0  0  0  0 | 0  0  0  0  0 | 0  0  0  0  0 | 0  0  0  0  0 | 0  0  0  0  0 | 0  0  1  0  0 | 0  0  0  0  0 | 0  0  0  0  0 | 0  0  0  0  0 |

| Gibbons | Kaye | A Virtuous Woman | USA | 1 | 1989 | 2 | 1960 | 0 | 0 | 0 | 0 | 0 | 0 | 0 | 0 | 0 0 | 0 | 0 | 0 | 0 | 0 | 0 |
| --- | --- | --- | --- | --- | --- | --- | --- | --- | --- | --- | --- | --- | --- | --- | --- | --- | --- | --- | --- | --- | --- | --- |
| Gibbons | Kaye | Ellen Foster | USA | 1 | 1987 | 2 | 1960 | 0 | 0 | 0 | 0 | 0 | 0 | 0 | 0 | 0 0 | 0 | 0 | 0 | 0 | 0 | 0 |

| Gibson | William | Neuromancer | USA | 0 | 1984 | 2 | 1948 | 0 | 0 | 0 | 0 | 0 | 1 | 0 | 0 | 0 | 0 | 0 | 0 | 0 | 0 | 0 | 0 |
| --- | --- | --- | --- | --- | --- | --- | --- | --- | --- | --- | --- | --- | --- | --- | --- | --- | --- | --- | --- | --- | --- | --- | --- |
| Gide | André | The Counterfeiters | FRANCE | 0 | 1925 | 1 | 1869 | 1 | 1 | 0 | 0 | 0 | 0 | 0 | 0 | 0 | 0 | 0 | 0 | 0 | 0 | 0 | 0 |
| Ginzburg | Natalia | Family lexicon | ITALY | 1 | 1963 | 1 | 1916 | 1 | 0 | 0 | 0 | 0 | 0 | 0 | 0 | 0 | 0 | 0 | 0 | 0 | 0 | 0 | 0 |
| Glasgow | Ellen | In This Our Life | USA | 1 | 1941 | 2 | 1873 | 0 | 0 | 0 | 0 | 1 | 0 | 0 | 0 | 0 | 0 | 0 | 0 | 0 | 0 | 0 | 0 |
| Glass | Emma | Peach | UK | 1 | 2018 | 1 | 1987 | 0 | 0 | 0 | 0 | 0 | 0 | 0 | 0 | 0 | 0 | 0 | 0 | 0 | 0 | 0 | 0 |
| Glyn | Elinor | Three Weeks | UK | 1 | 1907 | 1 | 1864 | 0 | 0 | 0 | 0 | 0 | 0 | 0 | 0 | 0 | 0 | 0 | 0 | 0 | 0 | 0 | 0 |
| Gogol | Nikolai | Dead soul | RUSSIA | 0 | 1842 | 1 | 1809 | 1 | 0 | 0 | 0 | 0 | 0 | 0 | 0 | 0 | 0 | 0 | 0 | 0 | 0 | 0 | 0 |
| Gogol | Nikolai | The nose | RUSSIA | 0 | 1836 | 1 | 1809 | 1 | 0 | 0 | 0 | 0 | 0 | 0 | 0 | 0 | 0 | 0 | 0 | 0 | 0 | 0 | 0 |
| Golden | Arthur | Memoirs of a Geisha | USA | 0 | 1997 | 2 | 1956 | 0 | 0 | 0 | 0 | 0 | 0 | 0 | 0 | 0 | 0 | 0 | 0 | 0 | 0 | 1 | 0 |
| Golding | William | Lord of the flies | UK | 0 | 1954 | 1 | 1911 | 0 | 1 | 0 | 0 | 0 | 0 | 0 | 0 | 0 | 0 | 0 | 0 | 0 | 0 | 1 | 0 |
| Golding | William | Rites of Passage | UK | 0 | 1980 | 1 | 1911 | 0 | 1 | 0 | 1 | 0 | 0 | 0 | 0 | 0 | 0 | 0 | 0 | 0 | 0 | 0 | 0 |

| **SURNAME** | **NAME** | **TITLE** | **NATIONALITY** | **GENDER** | **PUB_YEAR** | **CONTINENT** | **DATE OF BIRTH** | **TRANSLATED** | **NOBEL** | **NEUSTADT** | **BOOKER** | **PULITZER** | **NEBULA** | **CAINE** | **FRANZ KAFKA** | **NBA** | **BBA** | **WOMEN'S PRIZE FOR FICTION** | **NEW YORK TIMES** | **THE GUARDIAN** | **THE NEW YORKER** | **BBC** | **ATLANTIC** |
| --- | --- | --- | --- | --- | --- | --- | --- | --- | --- | --- | --- | --- | --- | --- | --- | --- | --- | --- | --- | --- | --- | --- | --- |
| Goldsmith | Oliver | The Vicar of Wakefield | IRELAND | 0 | 1766 | 1 | 1728 | 0 | 0 | 0 | 0 | 0 | 0 | 0 | 0 | 0 | 0 | 0 | 0 | 0 | 0 | 0 | 0 |
| Goldsmith | Oliver | She Stoops to Conquer | IRELAND | 0 | 1771 | 1 | 1728 | 0 | 0 | 0 | 0 | 0 | 0 | 0 | 0 | 0 | 0 | 0 | 0 | 0 | 0 | 0 | 0 |
| Gončarov | Ivan | Oblomov | RUSSIA | 0 | 1859 | 1 | 1812 | 1 | 0 | 0 | 0 | 0 | 0 | 0 | 0 | 0 | 0 | 0 | 0 | 0 | 0 | 0 | 0 |
| Gordimer | Nadine | Burger's daughter | SOUTH AFRICA | 1 | 1979 | 5 | 1923 | 0 | 1 | 0 | 0 | 0 | 0 | 0 | 0 | 0 | 0 | 0 | 0 | 0 | 0 | 0 | 0 |
| Gordimer | Nadine | A world of strangers | SOUTH AFRICA | 1 | 1958 | 5 | 1923 | 0 | 1 | 0 | 0 | 0 | 0 | 0 | 0 | 0 | 0 | 0 | 0 | 0 | 0 | 0 | 0 |
| Gordimer | Nadine | Beethoven was one-sixteenth black | SOUTH AFRICA | 1 | 2007 | 5 | 1923 | 0 | 1 | 0 | 0 | 0 | 0 | 0 | 0 | 0 | 0 | 0 | 0 | 0 | 0 | 0 | 0 |
| Gordimer | Nadine | The conservationist | SOUTH AFRICA | 1 | 1974 | 5 | 1923 | 0 | 1 | 0 | 1 | 0 | 0 | 0 | 0 | 0 | 0 | 0 | 0 | 0 | 0 | 0 | 0 |
| Goytisolo | Juan | Marks of Identity | SPAIN | 0 | 1966 | 1 | 1931 | 1 | 0 | 0 | 0 | 0 | 0 | 0 | 0 | 0 | 0 | 0 | 0 | 0 | 0 | 0 | 0 |
| Grahame | Kenneth | The Wind in the Willows | UK | 0 | 1908 | 1 | 1859 | 0 | 0 | 0 | 0 | 0 | 0 | 0 | 0 | 0 | 0 | 0 | 0 | 0 | 0 | 1 | 0 |
| Grandes | Almudena | The ages of Lulù | SPAIN | 1 | 1989 | 1 | 1960 | 1 | 0 | 0 | 0 | 0 | 0 | 0 | 0 | 0 | 0 | 0 | 0 | 0 | 0 | 0 | 0 |
| Grandes | Almudena | The frozen heart | SPAIN | 1 | 2007 | 1 | 1960 | 1 | 0 | 0 | 0 | 0 | 0 | 0 | 0 | 0 | 0 | 0 | 0 | 0 | 0 | 0 | 0 |
| Grant | Linda | The Clothes on Their Backs | UK | 1 | 2008 | 1 | 1951 | 0 | 0 | 0 | 0 | 0 | 0 | 0 | 0 | 0 | 0 | 0 | 0 | 0 | 0 | 0 | 0 |
| Grass | Günter | The tin drum | GERMANY | 0 | 1959 | 1 | 1927 | 1 | 1 | 0 | 0 | 0 | 0 | 0 | 0 | 0 | 0 | 0 | 0 | 0 | 0 | 0 | 0 |
| Grass | Günter | The flounder | GERMANY | 0 | 1977 | 1 | 1927 | 1 | 1 | 0 | 0 | 0 | 0 | 0 | 0 | 0 | 0 | 0 | 0 | 0 | 0 | 0 | 0 |
| Grau | Shirley Ann | The Keepers of the House | USA | 1 | 1964 | 2 | 1929 | 0 | 0 | 0 | 0 | 1 | 0 | 0 | 0 | 0 | 0 | 0 | 0 | 0 | 0 | 0 | 0 |
| Green | John | The fault in our stars | USA | 0 | 2012 | 2 | 1977 | 0 | 0 | 0 | 0 | 0 | 0 | 0 | 0 | 0 | 0 | 0 | 0 | 0 | 0 | 0 | 0 |
| Green | John | Looking for Alaska | USA | 0 | 2005 | 2 | 1977 | 0 | 0 | 0 | 0 | 0 | 0 | 0 | 0 | 0 | 0 | 0 | 0 | 0 | 0 | 0 | 0 |
| Green | Henry | Loving | UK | 0 | 1945 | 1 | 1905 | 0 | 0 | 0 | 0 | 0 | 0 | 0 | 0 | 0 | 0 | 0 | 0 | 0 | 0 | 0 | 0 |
| Greenwell | Garth | Cleanness | USA | 0 | 2020 | 2 | 1978 | 0 | 0 | 0 | 0 | 0 | 0 | 0 | 0 | 0 | 0 | 0 | 0 | 0 | 1 | 1 | 0 |
| Greer | Andrew Sean | Less | USA | 0 | 2017 | 2 | 1970 | 0 | 0 | 0 | 0 | 1 | 0 | 0 | 0 | 0 | 0 | 0 | 0 | 0 | 0 | 0 | 1 |
| Grenville | Kate | The secret river | AUSTRALIA | 1 | 2005 | 6 | 1950 | 0 | 0 | 0 | 0 | 0 | 0 | 0 | 0 | 0 | 0 | 0 | 0 | 0 | 0 | 0 | 0 |
| Grimm | Jacob Wilhelm | The complete Grimms fairy tales | GERMANY | 0 | 1812 | 1 | 1785 | 1 | 0 | 0 | 0 | 0 | 0 | 0 | 0 | 0 | 0 | 0 | 0 | 0 | 0 | 0 | 0 |
| Grisham | John | The firm | USA | 0 | 1991 | 2 | 1955 | 0 | 0 | 0 | 0 | 0 | 0 | 0 | 0 | 0 | 1 | 0 | 0 | 0 | 0 | 0 | 0 |
| Grisham | John | The rainmaker | USA | 0 | 1995 | 2 | 1955 | 0 | 0 | 0 | 0 | 0 | 0 | 0 | 0 | 0 | 1 | 0 | 0 | 0 | 0 | 0 | 0 |
| Grisham | John | The runaway jury | USA | 0 | 1996 | 2 | 1955 | 0 | 0 | 0 | 0 | 0 | 0 | 0 | 0 | 0 | 1 | 0 | 0 | 0 | 0 | 0 | 0 |
| Grisham | John | The client | USA | 0 | 1993 | 2 | 1955 | 0 | 0 | 0 | 0 | 0 | 0 | 0 | 0 | 0 | 1 | 0 | 0 | 0 | 0 | 0 | 0 |
| Groff | Lauren | Fates and furies | USA | 1 | 2015 | 2 | 1978 | 0 | 0 | 0 | 0 | 0 | 0 | 0 | 0 | 0 | 0 | 0 | 0 | 0 | 0 | 0 | 1 |
| Grogan | John | Marley & me | USA | 0 | 2005 | 2 | 1957 | 0 | 0 | 0 | 0 | 0 | 0 | 0 | 0 | 0 | 0 | 0 | 0 | 0 | 0 | 0 | 0 |
| Groom | Winston | Forrest Gump | USA | 0 | 1986 | 2 | 1943 | 0 | 0 | 0 | 0 | 0 | 0 | 0 | 0 | 0 | 0 | 0 | 0 | 0 | 0 | 0 | 0 |
| Grossman | David | To the end of the land | ISRAEL | 0 | 2008 | 4 | 1954 | 1 | 0 | 0 | 0 | 0 | 0 | 0 | 0 | 0 | 0 | 0 | 0 | 1 | 0 | 0 | 0 |
| Grossman | David | Be my knife | ISRAEL | 0 | 1999 | 4 | 1954 | 1 | 0 | 0 | 0 | 0 | 0 | 0 | 0 | 0 | 0 | 0 | 0 | 0 | 0 | 0 | 0 |
| Grossman | Vasily | Everything flows | RUSSIA | 0 | 1970 | 1 | 1905 | 1 | 0 | 0 | 0 | 0 | 0 | 0 | 0 | 0 | 0 | 0 | 0 | 0 | 0 | 0 | 0 |
| Grossman | Vasily | Life and fate | RUSSIA | 0 | 1980 | 1 | 1905 | 1 | 0 | 0 | 0 | 0 | 0 | 0 | 0 | 0 | 0 | 0 | 0 | 0 | 0 | 0 | 0 |
| Grossmith | George | Diary of a Nobody | UK | 0 | 1892 | 1 | 1847 | 0 | 0 | 0 | 0 | 0 | 0 | 0 | 0 | 0 | 0 | 0 | 0 | 0 | 0 | 0 | 0 |
| Guevara | Ernesto | The motorcycle diaries | ARGENTINA | 0 | 1992 | 3 | 1928 | 1 | 0 | 0 | 0 | 0 | 0 | 0 | 0 | 0 | 0 | 0 | 0 | 0 | 0 | 0 | 0 |
| Gundar-Goshen | Ayelet | One Night, Markovitch | ISRAEL | 1 | 2012 | 4 | 1982 | 1 | 0 | 0 | 0 | 0 | 0 | 0 | 0 | 0 | 0 | 0 | 0 | 0 | 0 | 0 | 0 |
| Gundar-Goshen | Ayelet | Waking lions | ISRAEL | 1 | 2014 | 4 | 1982 | 0 | 0 | 0 | 0 | 0 | 0 | 0 | 0 | 0 | 0 | 0 | 0 | 0 | 0 | 0 | 0 |
| Gunesekera | Romesh | Heaven's Edge | SRI LANKA | 0 | 2002 | 4 | 1954 | 0 | 0 | 0 | 0 | 0 | 0 | 0 | 0 | 0 | 0 | 0 | 0 | 0 | 0 | 0 | 0 |
| Gunesekera | Romesh | The Match | SRI LANKA | 0 | 2006 | 4 | 1954 | 0 | 0 | 0 | 0 | 0 | 0 | 0 | 0 | 0 | 0 | 0 | 0 | 0 | 0 | 0 | 0 |
| Gurdjieff | Georges | Meetings with remarkable men | RUSSIA | 0 | 1963 | 1 | 1872 | 1 | 0 | 0 | 0 | 0 | 0 | 0 | 0 | 0 | 0 | 0 | 0 | 0 | 0 | 0 | 0 |
| Gurnah | Abdulrazak | Desertion | TANZANIA | 0 | 2005 | 5 | 1948 | 0 | 1 | 0 | 0 | 0 | 0 | 0 | 0 | 0 | 0 | 0 | 0 | 0 | 0 | 0 | 0 |
| Gurnah | Abdulrazak | Afterlives | TANZANIA | 0 | 2020 | 5 | 1948 | 0 | 1 | 0 | 0 | 0 | 0 | 0 | 0 | 0 | 0 | 0 | 0 | 0 | 0 | 0 | 0 |
| Gurnah | Abdulrazak | Gravel Heart | TANZANIA | 0 | 2017 | 5 | 1948 | 0 | 1 | 0 | 0 | 0 | 0 | 0 | 0 | 0 | 0 | 0 | 0 | 0 | 0 | 0 | 0 |
| Gurnah | Abdulrazak | Memory of Departure | TANZANIA | 0 | 1987 | 5 | 1948 | 0 | 1 | 0 | 0 | 0 | 0 | 0 | 0 | 0 | 0 | 0 | 0 | 0 | 0 | 0 | 0 |
| Guthrie | lfred Bertram J | The Way West | USA | 0 | 1949 | 2 | 1901 | 0 | 0 | 0 | 0 | 1 | 0 | 0 | 0 | 0 | 0 | 0 | 0 | 0 | 0 | 0 | 0 |
| Gyasi | Yaa | Homegoing | GHANA | 1 | 2016 | 5 | 1989 | 0 | 0 | 0 | 0 | 0 | 0 | 0 | 0 | 0 | 0 | 0 | 0 | 1 | 0 | 1 | 1 |
| Hachtroudi | Fariba | The Man Who Snapped His Fingers | IRAN | 1 | 2016 | 4 | 1951 | 1 | 0 | 0 | 0 | 0 | 0 | 0 | 0 | 0 | 0 | 0 | 0 | 0 | 0 | 0 | 0 |
| Haddon | Mark | curious incident of the dog in the night- | UK | 0 | 2003 | 1 | 1962 | 0 | 0 | 0 | 0 | 0 | 0 | 0 | 0 | 0 | 1 | 0 | 0 | 0 | 0 | 0 | 0 |
| Hagedorn | Jessica | Dogeaters | PHILIPPINES | 1 | 1990 | 4 | 1949 | 0 | 0 | 0 | 0 | 0 | 0 | 0 | 0 | 0 | 0 | 0 | 0 | 0 | 0 | 0 | 0 |
| Hagedorn | Jessica | Toxicology | PHILIPPINES | 1 | 2011 | 4 | 1949 | 0 | 0 | 0 | 0 | 0 | 0 | 0 | 0 | 0 | 0 | 0 | 0 | 0 | 0 | 0 | 0 |
| Haggard | Rider | King Solomon's Mines | UK | 0 | 1885 | 1 | 1856 | 0 | 0 | 0 | 0 | 0 | 0 | 0 | 0 | 0 | 0 | 0 | 0 | 0 | 0 | 0 | 0 |
| Haggard | Rider | She: A History of Adventure | UK | 0 | 1887 | 1 | 1856 | 0 | 0 | 0 | 0 | 0 | 0 | 0 | 0 | 0 | 0 | 0 | 0 | 0 | 0 | 0 | 0 |
| Haggard | Rider | Cleopatra | UK | 0 | 1889 | 1 | 1856 | 0 | 0 | 0 | 0 | 0 | 0 | 0 | 0 | 0 | 0 | 0 | 0 | 0 | 0 | 0 | 0 |
| Haggard | Rider | Montezuma's Daughter | UK | 0 | 1893 | 1 | 1856 | 0 | 0 | 0 | 0 | 0 | 0 | 0 | 0 | 0 | 0 | 0 | 0 | 0 | 0 | 0 | 0 |
| Haggard | Rider | Moon of Israel | UK | 0 | 1918 | 1 | 1856 | 0 | 0 | 0 | 0 | 0 | 0 | 0 | 0 | 0 | 0 | 0 | 0 | 0 | 0 | 0 | 0 |
| Haggard | Rider | The Brethren | UK | 0 | 1904 | 1 | 1856 | 0 | 0 | 0 | 0 | 0 | 0 | 0 | 0 | 0 | 0 | 0 | 0 | 0 | 0 | 0 | 0 |
| Haggard | Rider | The People of the Mist | UK | 0 | 1894 | 1 | 1856 | 0 | 0 | 0 | 0 | 0 | 0 | 0 | 0 | 0 | 0 | 0 | 0 | 0 | 0 | 0 | 0 |
| Haley | Alex | Roots | USA | 0 | 1976 | 2 | 1921 | 0 | 0 | 0 | 0 | 0 | 0 | 0 | 0 | 0 | 0 | 0 | 0 | 0 | 0 | 0 | 0 |
| Halfon | Eduardo | The Polish Boxer | GUATEMALA | 0 | 2008 | 3 | 1971 | 1 | 0 | 0 | 0 | 0 | 0 | 0 | 0 | 0 | 0 | 0 | 0 | 0 | 0 | 0 | 0 |
| Hall | Sarah | The Electric Michelangelo | UK | 1 | 2004 | 1 | 1974 | 0 | 0 | 0 | 0 | 0 | 0 | 0 | 0 | 0 | 0 | 0 | 0 | 0 | 0 | 0 | 0 |
| Ham | Rosalie | The dressmaker | AUSTRALIA | 1 | 1973 | 6 | 1955 | 0 | 0 | 0 | 0 | 0 | 0 | 0 | 0 | 0 | 0 | 0 | 0 | 0 | 0 | 0 | 0 |
| Hamid | Mohsin | Exit west | PAKISTAN | 0 | 2017 | 4 | 1971 | 0 | 0 | 0 | 0 | 0 | 0 | 0 | 0 | 0 | 0 | 0 | 1 | 1 | 0 | 0 | 1 |
| Hamid | Mohsin | Moth smoke | PAKISTAN | 0 | 2000 | 4 | 1971 | 0 | 0 | 0 | 0 | 0 | 0 | 0 | 0 | 0 | 0 | 0 | 0 | 0 | 0 | 0 | 0 |
| Hamid | Mohsin | How to Get Filthy Rich in Rising Asia | PAKISTAN | 0 | 2013 | 4 | 1971 | 0 | 0 | 0 | 0 | 0 | 0 | 0 | 0 | 0 | 0 | 0 | 0 | 0 | 0 | 0 | 0 |
| Hamid | Mohsin | The Reluctant Fundamentalist | PAKISTAN | 0 | 2007 | 4 | 1971 | 0 | 0 | 0 | 0 | 0 | 0 | 0 | 0 | 0 | 0 | 0 | 0 | 0 | 0 | 0 | 0 |
| Hamilton | Jane | A Map of the World | USA | 1 | 1994 | 2 | 1957 | 0 | 0 | 0 | 0 | 0 | 0 | 0 | 0 | 0 | 0 | 0 | 0 | 0 | 0 | 0 | 0 |
| Hamilton | Jane | The Book of Ruth | USA | 1 | 1988 | 2 | 1957 | 0 | 0 | 0 | 0 | 0 | 0 | 0 | 0 | 0 | 0 | 0 | 0 | 0 | 0 | 0 | 0 |
| Hammett | Dashiell | The glass key | USA | 0 | 1931 | 2 | 1894 | 0 | 0 | 0 | 0 | 0 | 0 | 0 | 0 | 0 | 0 | 0 | 0 | 0 | 0 | 0 | 0 |
| Hammett | Dashiell | The maltese falcon | USA | 0 | 1929 | 2 | 1894 | 0 | 0 | 0 | 0 | 0 | 0 | 0 | 0 | 0 | 0 | 0 | 0 | 0 | 0 | 0 | 0 |
| Hampson | Amanda | The french perfumer | NEW ZEALAND | 1 | 2016 | 6 | ? | 0 | 0 | 0 | 0 | 0 | 0 | 0 | 0 | 0 | 0 | 0 | 0 | 0 | 0 | 0 | 0 |
| Hamsun | Knut | Under the autumn star | NORWAY | 0 | 1906 | 1 | 1859 | 1 | 1 | 0 | 0 | 0 | 0 | 0 | 0 | 0 | 0 | 0 | 0 | 0 | 0 | 0 | 0 |
| Hamsun | Knut | Hunger | NORWAY | 0 | 1890 | 1 | 1859 | 1 | 1 | 0 | 0 | 0 | 0 | 0 | 0 | 0 | 0 | 0 | 0 | 0 | 0 | 0 | 0 |
| Handke | Peter | The Left-Handed Woman | AUSTRIA | 0 | 1976 | 1 | 1942 | 1 | 1 | 0 | 0 | 0 | 0 | 0 | 1 | 0 | 0 | 0 | 0 | 0 | 0 | 0 | 0 |
| Haratischwili | Nino | The Eighth Life | GEORGIA | 1 | 2014 | 4 | 1983 | 1 | 0 | 0 | 0 | 0 | 0 | 0 | 0 | 0 | 0 | 0 | 0 | 0 | 0 | 0 | 0 |
| Harbach | Chad | The Art of Fielding | USA | 0 | 2011 | 2 | 1975 | 0 | 0 | 0 | 0 | 0 | 0 | 0 | 0 | 0 | 0 | 0 | 1 | 0 | 0 | 0 | 0 |
| Harding | Paul | Tinkers | USA | 0 | 2009 | 2 | 1967 | 0 | 0 | 0 | 0 | 1 | 0 | 0 | 0 | 0 | 0 | 0 | 0 | 0 | 0 | 0 | 0 |
| Harding | Paul | The anger of God | USA | 0 | 1993 | 2 | 1967 | 0 | 0 | 0 | 0 | 0 | 0 | 0 | 0 | 0 | 0 | 0 | 0 | 0 | 0 | 0 | 0 |
| Hardy | Thomas | Tess of the Urbervilles | UK | 0 | 1891 | 1 | 1840 | 0 | 0 | 0 | 0 | 0 | 0 | 0 | 0 | 0 | 0 | 0 | 0 | 0 | 0 | 1 | 0 |
| Hardy | Thomas | Far from the Madding Crowd | UK | 0 | 1874 | 1 | 1840 | 0 | 0 | 0 | 0 | 0 | 0 | 0 | 0 | 0 | 0 | 0 | 0 | 0 | 0 | 1 | 0 |
| Hardy | Thomas | Desperate Remedies | UK | 0 | 1871 | 1 | 1840 | 0 | 0 | 0 | 0 | 0 | 0 | 0 | 0 | 0 | 0 | 0 | 0 | 0 | 0 | 0 | 0 |
| Harrison | MJ | The Sunken Land Begins to Rise Again | UK | 0 | 2020 | 1 | 1945 | 0 | 0 | 0 | 0 | 0 | 0 | 0 | 0 | 0 | 0 | 0 | 0 | 1 | 0 | 0 | 0 |
| Hartnett | Sonya | The children of the king | AUSTRALIA | 1 | 2012 | 6 | 1968 | 0 | 0 | 0 | 0 | 0 | 0 | 0 | 0 | 0 | 0 | 0 | 0 | 0 | 0 | 0 | 0 |
| Hartnett | Sonya | Golden boys | AUSTRALIA | 1 | 2014 | 6 | 1968 | 0 | 0 | 0 | 0 | 0 | 0 | 0 | 0 | 0 | 0 | 0 | 0 | 0 | 0 | 0 | 0 |
| Hašek | Jaroslav | The Good Soldier Schweik | CZECH REP. | 0 | 1923 | 1 | 1883 | 1 | 0 | 0 | 0 | 0 | 0 | 0 | 0 | 0 | 0 | 0 | 0 | 0 | 0 | 0 | 0 |
| Haslett | Adam | Imagine me gone | USA | 0 | 2016 | 2 | 1970 | 0 | 0 | 0 | 0 | 0 | 0 | 0 | 0 | 0 | 0 | 0 | 0 | 0 | 0 | 1 | 0 |
| Hauptmann | Gerhart | Atlantis | GERMANY | 0 | 1912 | 1 | 1862 | 1 | 1 | 0 | 0 | 0 | 0 | 0 | 0 | 0 | 0 | 0 | 0 | 0 | 0 | 0 | 0 |
| Hawkins | Paula | The girl on the train | UK | 1 | 2015 | 1 | 1972 | 0 | 0 | 0 | 0 | 0 | 0 | 0 | 0 | 0 | 0 | 0 | 0 | 0 | 0 | 0 | 0 |

| **SURNAME** | **NAME** | | **TITLE** | **NATIONALITY** | | **GENDER** | **PUB_YEAR** | **CONTINENT** | **DATE OF BIRTH** | **TRANSLATED** | **NOBEL** | **NEUSTADT** | **BOOKER** | **PULITZER** | **NEBULA** | **CAINE** | **FRANZ KAFKA** | **NBA** | **BBA** | **WOMEN'S PRIZE FOR FICTION** | **NEW YORK TIMES** | **THE GUARDIAN** | **THE NEW YORKER** | **BBC** | **ATLANTIC** |
| --- | --- | --- | --- | --- | --- | --- | --- | --- | --- | --- | --- | --- | --- | --- | --- | --- | --- | --- | --- | --- | --- | --- | --- | --- | --- |
| Hawthorne | Nathaniel | | The scarlet letter | | USA | 0 | 1850 | 2 | 1804 | 0 | 0 | 0 | 0 | 0 | 0 | 0 | 0 | 0 | 0 | 0 | 0 | 0 | 0 | 0 | 0 |
| Hawthorne | Nathaniel | | The House of the Seven Gables | | USA | 0 | 1851 | 2 | 1804 | 0 | 0 | 0 | 0 | 0 | 0 | 0 | 0 | 0 | 0 | 0 | 0 | 0 | 0 | 0 | 0 |
| Hay | Ashley | | The Railwayman's Wife | | AUSTRALIA | 1 | 2013 | 6 | ? | 0 | 0 | 0 | 0 | 0 | 0 | 0 | 0 | 0 | 0 | 0 | 0 | 0 | 0 | 0 | 0 |
| Haynes | Melinda | | Mother of pearl | | USA | 1 | 1999 | 2 | 1955 | 0 | 0 | 0 | 0 | 0 | 0 | 0 | 0 | 0 | 0 | 0 | 0 | 0 | 0 | 0 | 0 |
| Haywood | Eliza | | Love in Excess, or The Fatal Enquiry | | UK | 1 | 1719 | 1 | 1693 | 0 | 0 | 0 | 0 | 0 | 0 | 0 | 0 | 0 | 0 | 0 | 0 | 0 | 0 | 0 | 0 |
| Hazzard | Shirley | | The Bay of Noon | | AUSTRALIA | 1 | 1970 | 6 | 1931 | 0 | 0 | 0 | 0 | 0 | 0 | 0 | 0 | 0 | 0 | 0 | 0 | 0 | 0 | 0 | 0 |
| Hedayat | Sadeq | | The blind owl | | IRAN | 0 | 1936 | 4 | 1903 | 1 | 0 | 0 | 0 | 0 | 0 | 0 | 0 | 0 | 0 | 0 | 0 | 0 | 0 | 0 | 0 |
| Hedayat | Sadeq | | Three drops of blood | | IRAN | 0 | 1932 | 4 | 1903 | 1 | 0 | 0 | 0 | 0 | 0 | 0 | 0 | 0 | 0 | 0 | 0 | 0 | 0 | 0 | 0 |
| Hegi | Ursula | | Stones from the River | | GERMANY | 1 | 1994 | 1 | 1946 | 0 | 0 | 0 | 0 | 0 | 0 | 0 | 0 | 0 | 0 | 0 | 0 | 0 | 0 | 0 | 0 |
| Heinlein | Robert A. | | Stranger in a Strange Land | | USA | 0 | 1964 | 2 | 1907 | 0 | 0 | 0 | 0 | 0 | 0 | 0 | 0 | 0 | 0 | 0 | 0 | 0 | 0 | 0 | 0 |
| Heinlein | Robert A. | | Citizen of the Galaxy | | USA | 0 | 1957 | 2 | 1907 | 0 | 0 | 0 | 0 | 0 | 0 | 0 | 0 | 0 | 0 | 0 | 0 | 0 | 0 | 0 | 0 |
| Heller | Joseph | | Catch-22 | | USA | 0 | 1961 | 2 | 1923 | 0 | 0 | 0 | 0 | 0 | 0 | 0 | 0 | 0 | 0 | 0 | 0 | 0 | 0 | 1 | 0 |
| Heller | Zoë | | Notes on a Scandal | | UK | 1 | 2003 | 1 | 1965 | 0 | 0 | 0 | 0 | 0 | 0 | 0 | 0 | 0 | 0 | 0 | 0 | 0 | 0 | 0 | 0 |
| Hemingway | Ernest | | For whom the bell tolls | | USA | 0 | 1940 | 2 | 1899 | 0 | 1 | 0 | 0 | 0 | 0 | 0 | 0 | 0 | 0 | 0 | 0 | 0 | 0 | 0 | 0 |
| Hemingway | Ernest | | The old man and the sea | | USA | 0 | 1952 | 2 | 1899 | 0 | 1 | 0 | 0 | 1 | 0 | 0 | 0 | 0 | 0 | 0 | 0 | 0 | 0 | 0 | 0 |
| Hemingway | Ernest | | Green hills of Africa | | USA | 0 | 1935 | 2 | 1899 | 0 | 1 | 0 | 0 | 0 | 0 | 0 | 0 | 0 | 0 | 0 | 0 | 0 | 0 | 0 | 0 |
| Hemingway | Ernest | | The sun also rises | | USA | 0 | 1927 | 2 | 1899 | 0 | 1 | 0 | 0 | 0 | 0 | 0 | 0 | 0 | 0 | 0 | 0 | 0 | 0 | 0 | 0 |
| Hemingway | Ernest | | A farewell to arms | | USA | 0 | 1929 | 2 | 1899 | 0 | 1 | 0 | 0 | 0 | 0 | 0 | 0 | 0 | 0 | 0 | 0 | 0 | 0 | 0 | 0 |
| Hemon | Aleksandar | | The Making of Zombie Wars | | USA | 0 | 2015 | 2 | 1964 | 0 | 0 | 0 | 0 | 0 | 0 | 0 | 0 | 0 | 0 | 0 | 0 | 0 | 0 | 0 | 0 |
| Heng | Rachel | | Suicide club | | SINGAPORE | 1 | 2018 | 4 | 1988 | 0 | 0 | 0 | 0 | 0 | 0 | 0 | 0 | 0 | 0 | 0 | 0 | 0 | 0 | 0 | 0 |
| Hensher | Philip | | The Northern Clemency | | UK | 0 | 2008 | 1 | 1965 | 0 | 0 | 0 | 0 | 0 | 0 | 0 | 0 | 0 | 0 | 0 | 0 | 0 | 0 | 0 | 0 |
| Hepworth | Sally | | The secrets of midwives | | AUSTRALIA | 1 | 2015 | 6 | 1980 | 0 | 0 | 0 | 0 | 0 | 0 | 0 | 0 | 0 | 0 | 0 | 0 | 0 | 0 | 0 | 0 |
| Herbert | Frank | | Dune | | USA | 0 | 1965 | 2 | 1920 | 0 | 0 | 0 | 0 | 0 | 1 | 0 | 0 | 0 | 0 | 0 | 0 | 0 | 0 | 1 | 0 |
| Herbert | Xavier | | Poor Fellow My Country | | AUSTRALIA | 0 | 1975 | 6 | 1901 | 0 | 0 | 0 | 0 | 0 | 0 | 0 | 0 | 0 | 0 | 0 | 0 | 0 | 0 | 0 | 0 |
| Herlihy | James | | Midnight Cowboy | | USA | 0 | 1965 | 2 | 1927 | 0 | 0 | 0 | 0 | 0 | 0 | 0 | 0 | 0 | 0 | 0 | 0 | 0 | 0 | 0 | 0 |
| Hernandez | Felisberto | | Piano Stories | | URUGUAY | 0 | 1993 | 3 | 1902 | 1 | 0 | 0 | 0 | 0 | 0 | 0 | 0 | 0 | 0 | 0 | 0 | 0 | 0 | 0 | 0 |
| Herrera | Yuri | | Signs Preceding the End of the World | | MEXICO | 0 | 2009 | 3 | 1970 | 1 | 0 | 0 | 0 | 0 | 0 | 0 | 0 | 0 | 0 | 0 | 0 | 1 | 0 | 0 | 0 |
| Hersey | John | | A Bell for Adano | | CHINA | 0 | 1944 | 4 | 1914 | 0 | 0 | 0 | 0 | 1 | 0 | 0 | 0 | 0 | 0 | 0 | 0 | 0 | 0 | 0 | 0 |
| Hertmans | Stefan | | War and Turpentine | | BELGIUM | 0 | 2016 | 1 | 1951 | 1 | 0 | 0 | 0 | 0 | 0 | 0 | 0 | 0 | 0 | 0 | 1 | 0 | 0 | 0 | 0 |
| Hesse | Herman | | Siddharta | | SWITZERLAND | 0 | 1922 | 1 | 1877 | 1 | 1 | 0 | 0 | 0 | 0 | 0 | 0 | 0 | 0 | 0 | 0 | 0 | 0 | 0 | 0 |
| Hesse | Herman | | Narcissus and Goldmund | | SWITZERLAND | 0 | 1930 | 1 | 1877 | 1 | 1 | 0 | 0 | 0 | 0 | 0 | 0 | 0 | 0 | 0 | 0 | 0 | 0 | 0 | 0 |
| Heyer | Georgette | | These Old Shades | | UK | 1 | 1926 | 1 | 1902 | 0 | 0 | 0 | 0 | 0 | 0 | 0 | 0 | 0 | 0 | 0 | 0 | 0 | 0 | 0 | 0 |
| Heyer | Georgette | | Regency Buck | | UK | 1 | 1935 | 1 | 1902 | 0 | 0 | 0 | 0 | 0 | 0 | 0 | 0 | 0 | 0 | 0 | 0 | 0 | 0 | 0 | 0 |
| Heyer | Georgette | | Footsteps in the Dark | | UK | 1 | 1932 | 1 | 1902 | 0 | 0 | 0 | 0 | 0 | 0 | 0 | 0 | 0 | 0 | 0 | 0 | 0 | 0 | 0 | 0 |
| Heyer | Georgette | | A Blunt Instrument | | UK | 1 | 1938 | 1 | 1902 | 0 | 0 | 0 | 0 | 0 | 0 | 0 | 0 | 0 | 0 | 0 | 0 | 0 | 0 | 0 | 0 |
| Heyer | Georgette | | The Black Moth | | UK | 1 | 1921 | 1 | 1902 | 0 | 0 | 0 | 0 | 0 | 0 | 0 | 0 | 0 | 0 | 0 | 0 | 0 | 0 | 0 | 0 |
| Heyse | Paul | | L'arrabbiata | | GERMANY | 0 | 1855 | 1 | 1830 | 1 | 1 | 0 | 0 | 0 | 0 | 0 | 0 | 0 | 0 | 0 | 0 | 0 | 0 | 0 | 0 |
| Higgins | Jack | | The Eagle Has Landed | | UK | 0 | 1975 | 1 | 1929 | 0 | 0 | 0 | 0 | 0 | 0 | 0 | 0 | 0 | 0 | 0 | 0 | 0 | 0 | 0 | 0 |
| Highsmith | Patricia | | The talented Mr Ripley | | USA | 1 | 1955 | 2 | 1921 | 0 | 0 | 0 | 0 | 0 | 0 | 0 | 0 | 0 | 0 | 0 | 0 | 0 | 0 | 0 | 0 |
| Hijuelos | Oscar | | The Mambo Kings Play Songs of Love | | USA | 0 | 1989 | 2 | 1951 | 0 | 0 | 0 | 0 | 1 | 0 | 0 | 0 | 0 | 0 | 0 | 0 | 0 | 0 | 0 | 0 |
| Hilton | James | | Goodbye Mr Chips | | UK | 0 | 1934 | 1 | 1900 | 0 | 0 | 0 | 0 | 0 | 0 | 0 | 0 | 0 | 0 | 0 | 0 | 0 | 0 | 0 | 0 |
| Hilton | James | | Lost horizon | | UK | 0 | 1933 | 1 | 1900 | 0 | 0 | 0 | 0 | 0 | 0 | 0 | 0 | 0 | 0 | 0 | 0 | 0 | 0 | 0 | 0 |
| Himmich | Bensalem | | A Muslim Suicide | | MOROCCO | 0 | 2011 | 5 | 1948 | 1 | 0 | 0 | 0 | 0 | 0 | 0 | 0 | 0 | 0 | 0 | 0 | 0 | 0 | 0 | 0 |
| Himmich | Bensalem | | The Theocrat | | MOROCCO | 0 | 2009 | 5 | 1948 | 1 | 0 | 0 | 0 | 0 | 0 | 0 | 0 | 0 | 0 | 0 | 0 | 0 | 0 | 0 | 0 |
| Hinton | SE | | The Outsiders | | USA | 1 | 1967 | 2 | 1948 | 0 | 0 | 0 | 0 | 0 | 0 | 0 | 0 | 0 | 0 | 0 | 0 | 0 | 0 | 0 | 0 |
| Hirata | Andrea | | The rainbow troops | | INDONESIA | 0 | 2005 | 4 | 1967 | 1 | 0 | 0 | 0 | 0 | 0 | 0 | 0 | 0 | 0 | 0 | 0 | 0 | 0 | 0 | 0 |
| Høeg | Peter | | Miss Smilla's Feeling for Snow | | DENMARK | 0 | 1992 | 1 | 1957 | 1 | 0 | 0 | 0 | 0 | 0 | 0 | 0 | 0 | 0 | 0 | 0 | 0 | 0 | 0 | 0 |
| Hoffman | Alice | | Here on Earth | | USA | 1 | 1997 | 2 | 1952 | 0 | 0 | 0 | 0 | 0 | 0 | 0 | 0 | 0 | 0 | 0 | 0 | 0 | 0 | 0 | 0 |
| Hoffmann | ETA | | he Life and Opinions of the Tomcat Mur | | GERMANY | 0 | 1820 | 1 | 1776 | 1 | 0 | 0 | 0 | 0 | 0 | 0 | 0 | 0 | 0 | 0 | 0 | 0 | 0 | 0 | 0 |
| Hogg | James | | te Memoirs and Confessions of a Justifi | | UK | 0 | 1824 | 1 | 1770 | 0 | 0 | 0 | 0 | 0 | 0 | 0 | 0 | 0 | 0 | 0 | 0 | 0 | 0 | 0 | 0 |
| Holleran | Andrew | | Dancer from the Dance | | USA | 0 | 1978 | 2 | 1943 | 0 | 0 | 0 | 0 | 0 | 0 | 0 | 0 | 0 | 0 | 0 | 0 | 0 | 0 | 0 | 0 |
| Hollinghurst | Alan | | The Line of Beauty | | UK | 0 | 2004 | 1 | 1954 | 0 | 0 | 0 | 1 | 0 | 0 | 0 | 0 | 0 | 0 | 0 | 0 | 0 | 0 | 0 | 0 |
| Hollinghurst | Alan | | The Folding Star | | UK | 0 | 1994 | 1 | 1954 | 0 | 0 | 0 | 0 | 0 | 0 | 0 | 0 | 0 | 0 | 0 | 0 | 0 | 0 | 0 | 0 |
| Hollinghurst | Alan | | The Spell | | UK | 0 | 1998 | 1 | 1954 | 0 | 0 | 0 | 0 | 0 | 0 | 0 | 0 | 0 | 0 | 0 | 0 | 0 | 0 | 0 | 0 |
| Hollinghurst | Alan | | The Swimming-Pool Library | | UK | 0 | 1988 | 1 | 1954 | 0 | 0 | 0 | 0 | 0 | 0 | 0 | 0 | 0 | 0 | 0 | 0 | 0 | 0 | 0 | 0 |
| Holt | Victoria | | Mistress of Mellyn | | UK | 1 | 1960 | 1 | 1906 | 0 | 0 | 0 | 0 | 0 | 0 | 0 | 0 | 0 | 0 | 0 | 0 | 0 | 0 | 0 | 0 |
| Holt | Victoria | | The Secret Woman | | UK | 1 | 1970 | 1 | 1906 | 0 | 0 | 0 | 0 | 0 | 0 | 0 | 0 | 0 | 0 | 0 | 0 | 0 | 0 | 0 | 0 |
| Homes | Amy | | This book will save your life | | USA | 1 | 2006 | 2 | 1961 | 0 | 0 | 0 | 0 | 0 | 0 | 0 | 0 | 0 | 0 | 0 | 0 | 0 | 0 | 0 | 0 |
| Honeyman | Gail | | Eleanor Oliphant is completely fine | | UK | 1 | 2017 | 1 | 1972 | 0 | 0 | 0 | 0 | 0 | 0 | 0 | 0 | 0 | 1 | 0 | 0 | 0 | 0 | 0 | 0 |
| Hornby | Nick | | A long way down | | UK | 0 | 2005 | 1 | 1957 | 0 | 0 | 0 | 0 | 0 | 0 | 0 | 0 | 0 | 0 | 0 | 0 | 0 | 0 | 0 | 0 |
| Hornby | Nick | | High fidelity | | UK | 0 | 1995 | 1 | 1957 | 0 | 0 | 0 | 0 | 0 | 0 | 0 | 0 | 0 | 0 | 0 | 0 | 0 | 0 | 0 | 0 |
| Hosseini | Khaled | | The kite runner | | AFGHANISTAN | 0 | 2003 | 4 | 1965 | 0 | 0 | 0 | 0 | 0 | 0 | 0 | 0 | 0 | 0 | 0 | 0 | 0 | 0 | 0 | 0 |
| Hosseini | Khaled | | A thousand splendid suns | | AFGHANISTAN | 0 | 2007 | 4 | 1965 | 0 | 0 | 0 | 0 | 0 | 0 | 0 | 0 | 0 | 1 | 0 | 0 | 0 | 0 | 0 | 0 |
| Hosseini | Khaled | | And the mountains echoed | | AFGHANISTAN | 0 | 2013 | 4 | 1965 | 0 | 0 | 0 | 0 | 0 | 0 | 0 | 0 | 0 | 0 | 0 | 0 | 0 | 0 | 0 | 0 |
| Houellebecq | Michel | | Serotonin | | FRANCE | 0 | 2019 | 1 | 1956 | 1 | 0 | 0 | 0 | 0 | 0 | 0 | 0 | 0 | 0 | 0 | 0 | 0 | 0 | 0 | 0 |
| Houellebecq | Michel | | The elementary particles | | FRANCE | 0 | 1998 | 1 | 1956 | 1 | 0 | 0 | 0 | 0 | 0 | 0 | 0 | 0 | 0 | 0 | 0 | 0 | 0 | 0 | 0 |
| House | Richard | | The kills | | USA | 0 | 2014 | 2 | 1961 | 0 | 0 | 0 | 0 | 0 | 0 | 0 | 0 | 0 | 0 | 0 | 0 | 0 | 0 | 1 | 0 |
| Howard | Elizabeth Jane | | Something in disguise | | UK | 1 | 1969 | 1 | 1923 | 0 | 0 | 0 | 0 | 0 | 0 | 0 | 0 | 0 | 0 | 0 | 0 | 0 | 0 | 0 | 0 |
| Howard | Robert | | The hour of the dragon | | USA | 0 | 1950 | 2 | 1906 | 0 | 0 | 0 | 0 | 0 | 0 | 0 | 0 | 0 | 0 | 0 | 0 | 0 | 0 | 0 | 0 |
| Howatch | Susan | | Penmarric | | UK | 1 | 1971 | 1 | 1940 | 0 | 0 | 0 | 0 | 0 | 0 | 0 | 0 | 0 | 0 | 0 | 0 | 0 | 0 | 0 | 0 |
| Hrabal | Bohumil | | Too loud a solitude | | CZECH REP. | 0 | 1976 | 1 | 1914 | 1 | 0 | 0 | 0 | 0 | 0 | 0 | 0 | 0 | 0 | 0 | 0 | 0 | 0 | 0 | 0 |
| Hua | Yu | | Brothers | | CHINA | 0 | 2005 | 4 | 1960 | 1 | 0 | 0 | 0 | 0 | 0 | 0 | 0 | 0 | 0 | 0 | 0 | 0 | 0 | 0 | 0 |
| Hua | Yu | | Chronicle of a blood merchant | | CHINA | 0 | 1995 | 4 | 1960 | 1 | 0 | 0 | 0 | 0 | 0 | 0 | 0 | 0 | 0 | 0 | 0 | 0 | 0 | 0 | 0 |
| Hua | Yu | | The Seventh Day | | CHINA | 0 | 2013 | 4 | 1960 | 1 | 0 | 0 | 0 | 0 | 0 | 0 | 0 | 0 | 0 | 0 | 0 | 0 | 0 | 0 | 0 |
| Hubbard | Ron | | Battlefield Earth | | USA | 0 | 1982 | 2 | 1911 | 0 | 0 | 0 | 0 | 0 | 0 | 0 | 0 | 0 | 0 | 0 | 0 | 0 | 0 | 0 | 0 |
| Hugo | Victor | | Les miserables | | FRANCE | 0 | 1862 | 1 | 1802 | 1 | 0 | 0 | 0 | 0 | 0 | 0 | 0 | 0 | 0 | 0 | 0 | 0 | 0 | 0 | 0 |
| Hugo | Victor | | The man who laughs | | FRANCE | 0 | 1869 | 1 | 1802 | 1 | 0 | 0 | 0 | 0 | 0 | 0 | 0 | 0 | 0 | 0 | 0 | 0 | 0 | 0 | 0 |
| Hugo | Victor | | Notre Dame de Paris | | FRANCE | 0 | 1831 | 1 | 1802 | 1 | 0 | 0 | 0 | 0 | 0 | 0 | 0 | 0 | 0 | 0 | 0 | 0 | 0 | 0 | 0 |
| Hulme | Keri | | The Bone People | | NEW ZEALAND | 1 | 1984 | 6 | 1947 | 0 | 0 | 0 | 1 | 0 | 0 | 0 | 0 | 0 | 0 | 0 | 0 | 0 | 0 | 0 | 0 |
| Humaydan Younes | | Iman | Other Lives |  | LEBANON | 1 | 2014 | 4 | 1956 | 1 | 0 | 0 | 0 | 0 | 0 | 0 | 0 | 0 | 0 | 0 | 0 | 0 | 0 | 0 | 0 |
| Hume | Fergus | | The Mystery of a Hansom Cab | | UK | 0 | 1886 | 1 | 1859 | 0 | 0 | 0 | 0 | 0 | 0 | 0 | 0 | 0 | 0 | 0 | 0 | 0 | 0 | 0 | 0 |
| Hume | Fergus | | A Woman's Burden | | UK | 0 | 1901 | 1 | 1859 | 0 | 0 | 0 | 0 | 0 | 0 | 0 | 0 | 0 | 0 | 0 | 0 | 0 | 0 | 0 | 0 |
| Hume | Fergus | | Madame Midas | | UK | 0 | 1888 | 1 | 1859 | 0 | 0 | 0 | 0 | 0 | 0 | 0 | 0 | 0 | 0 | 0 | 0 | 0 | 0 | 0 | 0 |
| Hume | Fergus | | The Bishop's Secret | | UK | 0 | 1900 | 1 | 1859 | 0 | 0 | 0 | 0 | 0 | 0 | 0 | 0 | 0 | 0 | 0 | 0 | 0 | 0 | 0 | 0 |
| Hume | Fergus | | The Green Mummy | | UK | 0 | 1908 | 1 | 1859 | 0 | 0 | 0 | 0 | 0 | 0 | 0 | 0 | 0 | 0 | 0 | 0 | 0 | 0 | 0 | 0 |
| Hume | Fergus | | The Opal Serpent | | UK | 0 | 1905 | 1 | 1859 | 0 | 0 | 0 | 0 | 0 | 0 | 0 | 0 | 0 | 0 | 0 | 0 | 0 | 0 | 0 | 0 |
| Hume | Fergus | | The Pagan's Cup | | UK | 0 | 1902 | 1 | 1859 | 0 | 0 | 0 | 0 | 0 | 0 | 0 | 0 | 0 | 0 | 0 | 0 | 0 | 0 | 0 | 0 |

| **SURNAME** | **NAME** | **TITLE** | | **NATIONALITY** | | **GENDER** | **PUB_YEAR** | **CONTINENT** | **DATE OF BIRTH** | **TRANSLATED** | **NOBEL** | **NEUSTADT** | **BOOKER** | **PULITZER** | **NEBULA** | **CAINE** | **FRANZ KAFKA** | **NBA** | **BBA** | **WOMEN'S PRIZE FOR FICTION** | **NEW YORK TIMES** | **THE GUARDIAN** | **THE NEW YORKER** | **BBC** | **ATLANTIC** |
| --- | --- | --- | --- | --- | --- | --- | --- | --- | --- | --- | --- | --- | --- | --- | --- | --- | --- | --- | --- | --- | --- | --- | --- | --- | --- |
| Huong | Duong Thu | The Zenith | | | VIETNAM | 1 | 2009 | 4 | 1947 | 1 | 0 | 0 | 0 | 0 | 0 | 0 | 0 | 0 | 0 | 0 | 0 | 0 | 0 | 0 | 0 |
| Hurston | Zona Neale | Jonah's Gourd Vine | | | USA | 1 | 1934 | 2 | 1891 | 0 | 0 | 0 | 0 | 0 | 0 | 0 | 0 | 0 | 0 | 0 | 0 | 0 | 0 | 0 | 0 |
| Hurston | Zona Neale | Their Eyes Were Watching God | | | USA | 1 | 1937 | 2 | 1891 | 0 | 0 | 0 | 0 | 0 | 0 | 0 | 0 | 0 | 0 | 0 | 0 | 0 | 0 | 0 | 0 |
| Hustvedt | Siri | What I Loved | | | USA | 1 | 2003 | 2 | 1955 | 0 | 0 | 0 | 0 | 0 | 0 | 0 | 0 | 0 | 0 | 0 | 0 | 0 | 0 | 0 | 0 |
| Huxley | Aldous | Brave new world | | | UK | 0 | 1932 | 1 | 1894 | 0 | 0 | 0 | 0 | 0 | 0 | 0 | 0 | 0 | 0 | 0 | 0 | 0 | 0 | 1 | 0 |
| Huxley | Aldous | Brave new world revisited | | | UK | 0 | 1932 | 1 | 1894 | 0 | 0 | 0 | 0 | 0 | 0 | 0 | 0 | 0 | 0 | 0 | 0 | 0 | 0 | 0 | 0 |
| Huxley | Aldous | he doors of perception - Heaven and he | | | UK | 0 | 1954 | 1 | 1894 | 0 | 0 | 0 | 0 | 0 | 0 | 0 | 0 | 0 | 0 | 0 | 0 | 0 | 0 | 0 | 0 |
| Huysmans | Joris Karl | Against nature | | | FRANCE | 0 | 1884 | 1 | 1848 | 1 | 0 | 0 | 0 | 0 | 0 | 0 | 0 | 0 | 0 | 0 | 0 | 0 | 0 | 0 | 0 |
| Hwang | Sok-yong | Princess Bari | | | SOUTH KOREA | 0 | 2007 | 4 | 1943 | 1 | 0 | 0 | 0 | 0 | 0 | 0 | 0 | 0 | 0 | 0 | 0 | 0 | 0 | 0 | 0 |
| Hwang | Sok-yong | Familiar Things | | | SOUTH KOREA | 0 | 2017 | 4 | 1943 | 1 | 0 | 0 | 0 | 0 | 0 | 0 | 0 | 0 | 0 | 0 | 0 | 0 | 0 | 0 | 0 |
| Hwang | Sok-yong | At Dusk | | | SOUTH KOREA | 0 | 2018 | 4 | 1943 | 1 | 0 | 0 | 0 | 0 | 0 | 0 | 0 | 0 | 0 | 0 | 0 | 0 | 0 | 0 | 0 |
| Hyland | MJ | Carry me down | | | UK | 1 | 2006 | 1 | 1968 | 0 | 0 | 0 | 0 | 0 | 0 | 0 | 0 | 0 | 0 | 0 | 0 | 0 | 0 | 0 | 0 |
| Ibrahim | Djamila | Things are good now | | | ETHIOPIA | 1 | 2018 | 5 | ? | 0 | 0 | 0 | 0 | 0 | 0 | 0 | 0 | 0 | 0 | 0 | 0 | 0 | 0 | 0 | 0 |
| Ibrahim | Sonallah | That Smell and Notes From Prison | | | EGYPT | 0 | 1966 | 5 | 1937 | 1 | 0 | 0 | 0 | 0 | 0 | 0 | 0 | 0 | 0 | 0 | 0 | 0 | 0 | 0 | 0 |
| Ibrahim | Sonallah | The Committee | | | EGYPT | 0 | 1981 | 5 | 1937 | 1 | 0 | 0 | 0 | 0 | 0 | 0 | 0 | 0 | 0 | 0 | 0 | 0 | 0 | 0 | 0 |
| Inchbald | Elizabeth | A Simple Story | | | UK | 1 | 1791 | 1 | 1753 | 0 | 0 | 0 | 0 | 0 | 0 | 0 | 0 | 0 | 0 | 0 | 0 | 0 | 0 | 0 | 0 |
| Irving | John | The world according to Garp | | | USA | 0 | 1978 | 2 | 1942 | 0 | 0 | 0 | 0 | 0 | 0 | 0 | 0 | 1 | 0 | 0 | 0 | 0 | 0 | 0 | 0 |
| Irving | John | Hotel New Hampshire | | | USA | 0 | 1981 | 2 | 1942 | 0 | 0 | 0 | 0 | 0 | 0 | 0 | 0 | 0 | 0 | 0 | 0 | 0 | 0 | 0 | 0 |
| Irving | John | In one person | | | USA | 0 | 2012 | 2 | 1942 | 0 | 0 | 0 | 0 | 0 | 0 | 0 | 0 | 0 | 0 | 0 | 0 | 0 | 0 | 0 | 0 |
| Irving | Washington | The Legend of Sleepy Hollow | | | USA | 0 | 1820 | 2 | 1783 | 0 | 0 | 0 | 0 | 0 | 0 | 0 | 0 | 0 | 0 | 0 | 0 | 0 | 0 | 0 | 0 |
| Isaacson | Walter | Steve Jobs | | | USA | 0 | 2011 | 2 | 1952 | 0 | 0 | 0 | 0 | 0 | 0 | 0 | 0 | 0 | 0 | 0 | 0 | 0 | 0 | 0 | 1 |
| Isegawa | Moses | Abyssinian Chronicles | | | UGANDA | 0 | 1998 | 5 | 1963 | 0 | 0 | 0 | 0 | 0 | 0 | 0 | 0 | 0 | 0 | 0 | 0 | 0 | 0 | 0 | 0 |
| Isegawa | Moses | Snakepit | | | UGANDA | 0 | 1999 | 5 | 1963 | 1 | 0 | 0 | 0 | 0 | 0 | 0 | 0 | 0 | 0 | 0 | 0 | 0 | 0 | 0 | 0 |
| Isherwood | Christopher | A single man | | | USA | 0 | 1964 | 2 | 1904 | 0 | 0 | 0 | 0 | 0 | 0 | 0 | 0 | 0 | 0 | 0 | 0 | 0 | 0 | 0 | 0 |
| Ishiguro | Kazuo | The buried giant | | | UK | 0 | 2015 | 1 | 1954 | 0 | 1 | 0 | 0 | 0 | 0 | 0 | 0 | 0 | 0 | 0 | 0 | 1 | 0 | 0 | 0 |
| Ishiguro | Kazuo | Never let me go | | | UK | 0 | 2005 | 1 | 1954 | 0 | 1 | 0 | 0 | 0 | 0 | 0 | 0 | 0 | 0 | 0 | 0 | 0 | 0 | 0 | 0 |
| Ishiguro | Kazuo | The Remains of the Day | | | UK | 0 | 1989 | 1 | 1954 | 0 | 1 | 0 | 1 | 0 | 0 | 0 | 0 | 0 | 0 | 0 | 0 | 0 | 0 | 0 | 0 |
| Ismailov | Hamid | The Dead Lake | | | UZBEKISTAN | 0 | 2014 | 4 | 1954 | 1 | 0 | 0 | 0 | 0 | 0 | 0 | 0 | 0 | 0 | 0 | 0 | 0 | 0 | 0 | 0 |
| Ismailov | Hamid | The Devils' Dance | | | UZBEKISTAN | 0 | 2016 | 4 | 1954 | 1 | 0 | 0 | 0 | 0 | 0 | 0 | 0 | 0 | 0 | 0 | 0 | 0 | 0 | 0 | 0 |
| Ismailov | Hamid | The Railway | | | UZBEKISTAN | 0 | 2006 | 4 | 1954 | 1 | 0 | 0 | 0 | 0 | 0 | 0 | 0 | 0 | 0 | 0 | 0 | 0 | 0 | 0 | 0 |
| Iweala | Uzodinma | Speak no evil | | | USA | 0 | 2018 | 2 | 1982 | 0 | 0 | 0 | 0 | 0 | 0 | 0 | 0 | 0 | 0 | 0 | 0 | 0 | 0 | 0 | 0 |
| Jackson | Shirley | We have always lived in the castle | | | USA | 1 | 1962 | 2 | 1916 | 0 | 0 | 0 | 0 | 0 | 0 | 0 | 0 | 0 | 0 | 0 | 0 | 0 | 0 | 0 | 0 |
| Jackson | Mick | The Underground Man | | | UK | 0 | 1997 | 1 | 1960 | 0 | 0 | 0 | 0 | 0 | 0 | 0 | 0 | 0 | 0 | 0 | 0 | 0 | 0 | 0 | 0 |
| Jacobson | Howard | The Finkler Question | | | UK | 0 | 2010 | 1 | 1942 | 0 | 0 | 0 | 1 | 0 | 0 | 0 | 0 | 0 | 0 | 0 | 0 | 1 | 0 | 0 | 0 |
| Jacobson | Howard | J | | | UK | 0 | 2014 | 1 | 1942 | 0 | 0 | 0 | 0 | 0 | 0 | 0 | 0 | 0 | 0 | 0 | 0 | 0 | 0 | 0 | 0 |
| James | Henry | The turn of the screw | | | USA | 0 | 1898 | 2 | 1843 | 0 | 0 | 0 | 0 | 0 | 0 | 0 | 0 | 0 | 0 | 0 | 0 | 0 | 0 | 0 | 0 |
| James | Henry | The portrait of a lady | | | USA | 0 | 1881 | 2 | 1843 | 0 | 0 | 0 | 0 | 0 | 0 | 0 | 0 | 0 | 0 | 0 | 0 | 0 | 0 | 0 | 0 |
| James | Henry | The Ambassadors | | | USA | 0 | 1903 | 2 | 1843 | 0 | 0 | 0 | 0 | 0 | 0 | 0 | 0 | 0 | 0 | 0 | 0 | 0 | 0 | 0 | 0 |
| James | EL | Fifty shades of grey | | | UK | 1 | 2011 | 1 | 1963 | 0 | 0 | 0 | 0 | 0 | 0 | 0 | 0 | 0 | 1 | 0 | 0 | 0 | 0 | 0 | 0 |
| James | EL | Fifty shades freed | | | UK | 1 | 2012 | 1 | 1963 | 0 | 0 | 0 | 0 | 0 | 0 | 0 | 0 | 0 | 0 | 0 | 0 | 0 | 0 | 0 | 0 |
| James | Marlon | A Brief History of Seven Killings | | | JAMAICA | 1 | 2014 | 3 | 1970 | 0 | 0 | 0 | 1 | 0 | 0 | 0 | 0 | 0 | 0 | 0 | 0 | 1 | 0 | 1 | 0 |
| Jansson | Tove | The summer book | | | FINLAND | 1 | 1972 | 1 | 1914 | 1 | 0 | 0 | 0 | 0 | 0 | 0 | 0 | 0 | 0 | 0 | 0 | 0 | 0 | 0 | 0 |
| Jebreal | Rula | Miral | | | PALESTINE | 1 | 2004 | 4 | 1973 | 1 | 0 | 0 | 0 | 0 | 0 | 0 | 0 | 0 | 0 | 0 | 0 | 0 | 0 | 0 | 0 |
| Jelinek | Elfriede | Wonderful, Wonderful Times | | | AUSTRIA | 1 | 1980 | 1 | 1946 | 1 | 1 | 0 | 0 | 0 | 0 | 0 | 1 | 0 | 0 | 0 | 0 | 0 | 0 | 0 | 0 |
| Jelloun | Tahar Ben | The Last Friend | | | MOROCCO | 0 | 2006 | 5 | 1947 | 1 | 0 | 0 | 0 | 0 | 0 | 0 | 0 | 0 | 0 | 0 | 0 | 0 | 0 | 0 | 0 |
| Jelloun | Tahar Ben | The Happy Marriage | | | MOROCCO | 0 | 2012 | 5 | 1947 | 1 | 0 | 0 | 0 | 0 | 0 | 0 | 0 | 0 | 0 | 0 | 0 | 0 | 0 | 0 | 0 |
| Jerome | Jerome | Three men in a boat | | | UK | 0 | 1889 | 1 | 1859 | 0 | 0 | 0 | 0 | 0 | 0 | 0 | 0 | 0 | 0 | 0 | 0 | 0 | 0 | 0 | 0 |
| Jhabvala | Ruth Prawer | Heat and Dust | | | GERMANY | 1 | 1975 | 1 | 1927 | 0 | 0 | 0 | 1 | 0 | 0 | 0 | 0 | 0 | 0 | 0 | 0 | 0 | 0 | 0 | 0 |
| Jian | Ma | The dark road | | | CHINA | 0 | 2012 | 4 | 1953 | 1 | 0 | 0 | 0 | 0 | 0 | 0 | 0 | 0 | 0 | 0 | 0 | 0 | 0 | 0 | 0 |
| Jian | Ma | The Noodle Maker | | | CHINA | 0 | 2006 | 4 | 1953 | 1 | 0 | 0 | 0 | 0 | 0 | 0 | 0 | 0 | 0 | 0 | 0 | 0 | 0 | 0 | 0 |
| Johnson | Adam | The orphan master's son | | | USA | 0 | 2012 | 2 | 1967 | 0 | 0 | 0 | 0 | 1 | 0 | 0 | 0 | 0 | 0 | 0 | 0 | 0 | 0 | 0 | 0 |
| Johnson | Uwe | versaries: From a Year in the Life of Ge | | | GERMANY | 0 | 1970 | 1 | 1934 | 1 | 0 | 0 | 0 | 0 | 0 | 0 | 0 | 0 | 0 | 0 | 0 | 0 | 0 | 0 | 0 |
| Johnson | sephine Winslo | | Now in November |  | USA | 1 | 1934 | 2 | 1910 | 0 | 0 | 0 | 0 | 1 | 0 | 0 | 0 | 0 | 0 | 0 | 0 | 0 | 0 | 0 | 0 |
| Johnson | Denis | The Largesse of the Sea Maiden | | | USA | 0 | 2018 | 2 | 1949 | 0 | 0 | 0 | 0 | 0 | 0 | 0 | 0 | 0 | 0 | 0 | 0 | 0 | 0 | 0 | 0 |
| Johnson | Daisy | Everything Under | | | UK | 1 | 2018 | 1 | 1990 | 0 | 0 | 0 | 0 | 0 | 0 | 0 | 0 | 0 | 0 | 0 | 0 | 0 | 0 | 0 | 0 |
| Jones | Diana | Howl's Moving Castle | | | UK | 1 | 1986 | 1 | 1934 | 0 | 0 | 0 | 0 | 0 | 0 | 0 | 0 | 0 | 0 | 0 | 0 | 0 | 0 | 0 | 0 |
| Jones | Edward Paul | The Known World | | | USA | 0 | 2003 | 2 | 1951 | 0 | 0 | 0 | 0 | 1 | 0 | 0 | 0 | 0 | 0 | 0 | 0 | 0 | 0 | 0 | 0 |
| Jones | Lloyd | Mister Pip | | | NEW ZEALAND | 0 | 2006 | 6 | 1955 | 0 | 0 | 0 | 0 | 0 | 0 | 0 | 0 | 0 | 0 | 0 | 0 | 0 | 0 | 0 | 0 |
| Jong | Erica | Fear of Flying | | | USA | 1 | 1973 | 2 | 1942 | 0 | 0 | 0 | 0 | 0 | 0 | 0 | 0 | 0 | 0 | 0 | 0 | 0 | 0 | 0 | 0 |
| Jordan | Toni | Our Tiny, Useless Hearts | | | AUSTRALIA | 1 | 2016 | 6 | 1966 | 0 | 0 | 0 | 0 | 0 | 0 | 0 | 0 | 0 | 0 | 0 | 0 | 0 | 0 | 0 | 0 |
| Joyce | James | Dubliners | | | IRELAND | 0 | 1914 | 1 | 1882 | 0 | 0 | 0 | 0 | 0 | 0 | 0 | 0 | 0 | 0 | 0 | 0 | 0 | 0 | 0 | 0 |
| Joyce | James | A Portrait of the Artist as a Young Man | | | IRELAND | 0 | 1916 | 1 | 1882 | 0 | 0 | 0 | 0 | 0 | 0 | 0 | 0 | 0 | 0 | 0 | 0 | 0 | 0 | 0 | 0 |
| Joyce | James | Ulysses | | | IRELAND | 0 | 1922 | 1 | 1882 | 0 | 0 | 0 | 0 | 0 | 0 | 0 | 0 | 0 | 0 | 0 | 0 | 0 | 0 | 1 | 0 |
| Jufresa | Laia | Umami | | | MEXICO | 1 | 2015 | 3 | 1983 | 1 | 0 | 0 | 0 | 0 | 0 | 0 | 0 | 0 | 0 | 0 | 0 | 0 | 0 | 0 | 0 |
| Jünger | Ernst | Storm of steel | | | GERMANY | 0 | 1920 | 1 | 1895 | 1 | 0 | 0 | 0 | 0 | 0 | 0 | 0 | 0 | 0 | 0 | 0 | 0 | 0 | 0 | 0 |
| Kadare | Ismail | Broken April | | | ALBANIA | 0 | 1980 | 1 | 1936 | 1 | 0 | 1 | 1 | 0 | 0 | 0 | 0 | 0 | 0 | 0 | 0 | 0 | 0 | 0 | 0 |
| Kadare | Ismail | The Pyramid | | | ALBANIA | 0 | 1995 | 1 | 1936 | 1 | 0 | 1 | 1 | 0 | 0 | 0 | 0 | 0 | 0 | 0 | 0 | 0 | 0 | 0 | 0 |
| Kafka | Franz | The trial | | | CZECH REP. | 0 | 1925 | 1 | 1883 | 1 | 0 | 0 | 0 | 0 | 0 | 0 | 0 | 0 | 0 | 0 | 0 | 0 | 0 | 0 | 0 |
| Kafka | Franz | Metamorphosis | | | CZECH REP. | 0 | 1915 | 1 | 1883 | 1 | 0 | 0 | 0 | 0 | 0 | 0 | 0 | 0 | 0 | 0 | 0 | 0 | 0 | 0 | 0 |
| Kafka | Franz | The castle | | | CZECH REP. | 0 | 1926 | 1 | 1883 | 1 | 0 | 0 | 0 | 0 | 0 | 0 | 0 | 0 | 0 | 0 | 0 | 0 | 0 | 0 | 0 |
| Kakuta | Mitsuyo | Woman on the other shore | | | JAPAN | 1 | 2007 | 4 | 1967 | 1 | 0 | 0 | 0 | 0 | 0 | 0 | 0 | 0 | 0 | 0 | 0 | 0 | 0 | 0 | 0 |
| Kan | Karoline | Under red skies | | | CHINA | 1 | 2019 | 4 | ? | 0 | 0 | 0 | 0 | 0 | 0 | 0 | 0 | 0 | 0 | 0 | 0 | 0 | 0 | 0 | 0 |
| Kandasamy | Meena | When I hit you | | | INDIA | 1 | 2017 | 4 | 1984 | 0 | 0 | 0 | 0 | 0 | 0 | 0 | 0 | 0 | 0 | 0 | 0 | 1 | 0 | 0 | 0 |
| Kanehara | Hitomi | Snakes and earrings | | | JAPAN | 1 | 2005 | 4 | 1983 | 1 | 0 | 0 | 0 | 0 | 0 | 0 | 0 | 0 | 0 | 0 | 0 | 0 | 0 | 0 | 0 |
| Kang | Han | The vegetarian | | | SOUTH KOREA | 1 | 2007 | 4 | 1970 | 1 | 0 | 0 | 1 | 0 | 0 | 0 | 0 | 0 | 0 | 0 | 1 | 1 | 0 | 0 | 1 |
| Kang | Han | Human acts | | | SOUTH KOREA | 1 | 2014 | 4 | 1970 | 1 | 0 | 0 | 0 | 0 | 0 | 0 | 0 | 0 | 0 | 0 | 0 | 0 | 0 | 0 | 1 |
| Karr | Mary | The liar's club | | | USA | 1 | 1995 | 2 | 1955 | 0 | 0 | 0 | 0 | 0 | 0 | 0 | 0 | 0 | 0 | 0 | 0 | 0 | 0 | 0 | 0 |
| Karunatilaka | Shehan | Chinaman | | | SRI LANKA | 0 | 2010 | 4 | 1975 | 0 | 0 | 0 | 0 | 0 | 0 | 0 | 0 | 0 | 0 | 0 | 0 | 0 | 0 | 0 | 0 |
| Kawabata | Yasunari | Beauty and sadness | | | JAPAN | 0 | 1964 | 4 | 1899 | 1 | 1 | 0 | 0 | 0 | 0 | 0 | 0 | 0 | 0 | 0 | 0 | 0 | 0 | 0 | 0 |
| Kawabata | Yasunari | The Sound of the Mountain | | | JAPAN | 0 | 1954 | 4 | 1899 | 1 | 1 | 0 | 0 | 0 | 0 | 0 | 0 | 0 | 0 | 0 | 0 | 0 | 0 | 0 | 0 |
| Kawaguchi | Toshikazu | Before the cofee gets cold | | | JAPAN | 0 | 2015 | 4 | 1971 | 1 | 0 | 0 | 0 | 0 | 0 | 0 | 0 | 0 | 0 | 0 | 0 | 0 | 0 | 0 | 0 |
| Kawakami | Mieko | Breasts and eggs | | | JAPAN | 1 | 2008 | 4 | 1976 | 1 | 0 | 0 | 0 | 0 | 0 | 0 | 0 | 0 | 0 | 0 | 0 | 0 | 0 | 0 | 1 |
| Kawakami | Hiromi | Strange Weather in Tokyo | | | JAPAN | 1 | 2001 | 4 | 1958 | 1 | 0 | 0 | 0 | 0 | 0 | 0 | 0 | 0 | 0 | 0 | 0 | 0 | 0 | 0 | 0 |
| Kawakami | Hiromi | Record of a Night too Brief | | | JAPAN | 1 | 1996 | 4 | 1958 | 1 | 0 | 0 | 0 | 0 | 0 | 0 | 0 | 0 | 0 | 0 | 0 | 0 | 0 | 0 | 0 |
| Kawamura | Genki | If cats disappeared from the world | | | JAPAN | 0 | 2012 | 4 | 1979 | 1 | 0 | 0 | 0 | 0 | 0 | 0 | 0 | 0 | 0 | 0 | 0 | 0 | 0 | 0 | 0 |
| Kazantzakis | Nikos | Zorba the greek | | | GREECE | 0 | 1946 | 1 | 1883 | 1 | 0 | 0 | 0 | 0 | 0 | 0 | 0 | 0 | 0 | 0 | 0 | 0 | 0 | 0 | 0 |
| Kazantzakis | Nikos | The last temptation of Christ | | | GREECE | 0 | 1952 | 1 | 1883 | 1 | 0 | 0 | 0 | 0 | 0 | 0 | 0 | 0 | 0 | 0 | 0 | 0 | 0 | 0 | 0 |

| **SURNAME** | **NAME** | | **TITLE** | **NATIONALITY** | | **GENDER** | **PUB_YEAR** | **CONTINENT** | **DATE OF BIRTH** | **TRANSLATED** | **NOBEL** | **NEUSTADT** | **BOOKER** | **PULITZER** | **NEBULA** | **CAINE** | **FRANZ KAFKA** | **NBA** | **BBA** | **WOMEN'S PRIZE FOR FICTION** | **NEW YORK TIMES** | **THE GUARDIAN** | **THE NEW YORKER** | **BBC** | **ATLANTIC** |
| --- | --- | --- | --- | --- | --- | --- | --- | --- | --- | --- | --- | --- | --- | --- | --- | --- | --- | --- | --- | --- | --- | --- | --- | --- | --- |
| Keane | Molly | Good Behaviour | | | IRELAND | 1 | 1981 | 1 | 1904 | 0 | 0 | 0 | 0 | 0 | 0 | 0 | 0 | 0 | 0 | 0 | 0 | 0 | 0 | 0 | 0 |
| Kehlmann | Daniel | Measuring the world | | | GERMANY | 0 | 2005 | 1 | 1975 | 1 | 0 | 0 | 0 | 0 | 0 | 0 | 0 | 0 | 0 | 0 | 0 | 0 | 0 | 0 | 0 |
| Kehlmann | Daniel | Tyll | | | GERMANY | 0 | 2020 | 1 | 1975 | 1 | 0 | 0 | 0 | 0 | 0 | 0 | 0 | 0 | 0 | 0 | 0 | 0 | 0 | 0 | 0 |
| Kelman | James | A Disaffection | | | UK | 0 | 1989 | 1 | 1946 | 0 | 0 | 0 | 0 | 0 | 0 | 0 | 0 | 0 | 0 | 0 | 0 | 0 | 0 | 0 | 0 |
| Kelman | Stephen | Pigeon English | | | UK | 0 | 2011 | 1 | 1976 | 0 | 0 | 0 | 0 | 0 | 0 | 0 | 0 | 0 | 0 | 0 | 0 | 0 | 0 | 0 | 0 |
| Kempadoo | Oonya | All Decent Animals | | | GUYANA | 1 | 2013 | 3 | 1966 | 0 | 0 | 0 | 0 | 0 | 0 | 0 | 0 | 0 | 0 | 0 | 0 | 0 | 0 | 0 | 0 |
| Keneally | Thomas | Schindler's ark | | | AUSTRALIA | 0 | 1982 | 6 | 1935 | 0 | 0 | 0 | 1 | 0 | 0 | 0 | 0 | 0 | 0 | 0 | 0 | 0 | 0 | 0 | 0 |
| Kennedy | William | Ironweed | | | USA | 0 | 1983 | 2 | 1928 | 0 | 0 | 0 | 0 | 1 | 0 | 0 | 0 | 0 | 0 | 0 | 0 | 0 | 0 | 0 | 0 |
| Kent | Hannah | Burial rites | | | AUSTRALIA | 1 | 2013 | 6 | 1985 | 0 | 0 | 0 | 0 | 0 | 0 | 0 | 0 | 0 | 0 | 0 | 0 | 0 | 0 | 0 | 0 |
| Kent | Hannah | The good people | | | AUSTRALIA | 1 | 2016 | 6 | 1985 | 0 | 0 | 0 | 0 | 0 | 0 | 0 | 0 | 0 | 0 | 0 | 0 | 0 | 0 | 0 | 0 |
| Kerouac | Jack | On the road | | | USA | 0 | 1957 | 2 | 1922 | 0 | 0 | 0 | 0 | 0 | 0 | 0 | 0 | 0 | 0 | 0 | 0 | 0 | 0 | 1 | 0 |
| Kerr | Judith | When Hitler Stole Pink Rabbit | | | GERMANY | 1 | 1971 | 1 | 1923 | 0 | 0 | 0 | 0 | 0 | 0 | 0 | 0 | 0 | 0 | 0 | 0 | 0 | 0 | 0 | 0 |
| Kertész | Imre | Fatelessness | | | HUNGARY | 0 | 1975 | 1 | 1929 | 1 | 1 | 0 | 0 | 0 | 0 | 0 | 0 | 0 | 0 | 0 | 0 | 0 | 0 | 0 | 0 |
| Kertész | Imre | Kaddish for an Unborn Child | | | HUNGARY | 0 | 1990 | 1 | 1929 | 1 | 1 | 0 | 0 | 0 | 0 | 0 | 0 | 0 | 0 | 0 | 0 | 0 | 0 | 0 | 0 |
| Khadra | Yasmina | The attack | | | ALGERIA | 0 | 2005 | 5 | 1955 | 1 | 0 | 0 | 0 | 0 | 0 | 0 | 0 | 0 | 0 | 0 | 0 | 0 | 0 | 0 | 0 |
| Khadra | Yasmina | The Sirens of Baghdad | | | ALGERIA | 0 | 2006 | 5 | 1955 | 1 | 0 | 0 | 0 | 0 | 0 | 0 | 0 | 0 | 0 | 0 | 0 | 0 | 0 | 0 | 0 |
| Khal | Abdo | Throwing sparks | | | SAUDI ARABIA | 0 | 2016 | 4 | 1962 | 1 | 0 | 0 | 0 | 0 | 0 | 0 | 0 | 0 | 0 | 0 | 0 | 0 | 0 | 0 | 0 |
| Khalifa | Khaled | In praise of hatred | | | SYRIA | 0 | 2006 | 4 | 1964 | 1 | 0 | 0 | 0 | 0 | 0 | 0 | 0 | 0 | 0 | 0 | 0 | 0 | 0 | 0 | 0 |
| Khalifa | Khaled | Death Is Hard Work | | | SYRIA | 0 | 2016 | 4 | 1964 | 1 | 0 | 0 | 0 | 0 | 0 | 0 | 0 | 0 | 0 | 0 | 0 | 1 | 0 | 0 | 0 |
| Khoury | Elias | Gate of the Sun | | | LEBANON | 0 | 2000 | 4 | 1948 | 1 | 0 | 0 | 0 | 0 | 0 | 0 | 0 | 0 | 0 | 0 | 0 | 0 | 0 | 0 | 0 |
| Khoury | Elias | As Though She Were Sleeping | | | LEBANON | 0 | 2007 | 4 | 1948 | 1 | 0 | 0 | 0 | 0 | 0 | 0 | 0 | 0 | 0 | 0 | 0 | 0 | 0 | 0 | 0 |
| Khoury | Elias | Yalo | | | LEBANON | 0 | 2002 | 4 | 1948 | 1 | 0 | 0 | 0 | 0 | 0 | 0 | 0 | 0 | 0 | 0 | 0 | 0 | 0 | 0 | 0 |
| Kimani | Peter | Dance of the Jakaranda | | | KENYA | 0 | 2017 | 5 | ? | 0 | 0 | 0 | 0 | 0 | 0 | 0 | 0 | 0 | 0 | 0 | 0 | 0 | 0 | 0 | 0 |
| Kincaid | Jamaica | The autobiography of my mother | | | ANTIGUA | 1 | 1996 | 3 | 1949 | 0 | 0 | 0 | 0 | 0 | 0 | 0 | 0 | 0 | 0 | 0 | 0 | 0 | 0 | 0 | 0 |
| Kincaid | Jamaica | Annie John | | | ANTIGUA | 1 | 1985 | 3 | 1949 | 0 | 0 | 0 | 0 | 0 | 0 | 0 | 0 | 0 | 0 | 0 | 0 | 0 | 0 | 0 | 0 |
| Kincaid | Jamaica | Lucy: A Novel | | | ANTIGUA | 1 | 1990 | 3 | 1949 | 0 | 0 | 0 | 0 | 0 | 0 | 0 | 0 | 0 | 0 | 0 | 0 | 0 | 0 | 0 | 0 |
| Kincaid | Jamaica | See Now Then | | | ANTIGUA | 1 | 2013 | 3 | 1949 | 0 | 0 | 0 | 0 | 0 | 0 | 0 | 0 | 0 | 0 | 0 | 0 | 0 | 0 | 0 | 0 |
| King | Stephen | It | | | USA | 0 | 1986 | 2 | 1947 | 0 | 0 | 0 | 0 | 0 | 0 | 0 | 0 | 0 | 0 | 0 | 0 | 0 | 0 | 0 | 0 |
| King | Stephen | Shining | | | USA | 0 | 1977 | 2 | 1947 | 0 | 0 | 0 | 0 | 0 | 0 | 0 | 0 | 0 | 0 | 0 | 0 | 0 | 0 | 0 | 0 |
| King | Stephen | The green mile | | | USA | 0 | 1996 | 2 | 1947 | 0 | 0 | 0 | 0 | 0 | 0 | 0 | 0 | 0 | 0 | 0 | 0 | 0 | 0 | 0 | 0 |
| King | Stephen | Joyland | | | USA | 0 | 2013 | 2 | 1947 | 0 | 0 | 0 | 0 | 0 | 0 | 0 | 0 | 0 | 0 | 0 | 0 | 0 | 0 | 0 | 0 |
| King-Gamble | Marcia | Down and Out in Flamingo Beach | | | S. VINCENT | 1 | 2007 | 3 | ? | 0 | 0 | 0 | 0 | 0 | 0 | 0 | 0 | 0 | 0 | 0 | 0 | 0 | 0 | 0 | 0 |
| King-Gamble | Marcia | Flamingo Place | | | S. VINCENT | 1 | 2006 | 3 | ? | 0 | 0 | 0 | 0 | 0 | 0 | 0 | 0 | 0 | 0 | 0 | 0 | 0 | 0 | 0 | 0 |
| King-Gamble | Marcia | His Golden Heart | | | S. VINCENT | 1 | 2016 | 3 | ? | 0 | 0 | 0 | 0 | 0 | 0 | 0 | 0 | 0 | 0 | 0 | 0 | 0 | 0 | 0 | 0 |
| Kingsley | Charles | The Water-Babies | | | UK | 0 | 1863 | 1 | 1819 | 0 | 0 | 0 | 0 | 0 | 0 | 0 | 0 | 0 | 0 | 0 | 0 | 0 | 0 | 0 | 0 |
| Kingsnorth | Paul | Beast | | | UK | 0 | 2016 | 1 | 1972 | 0 | 0 | 0 | 0 | 0 | 0 | 0 | 0 | 0 | 0 | 0 | 0 | 1 | 0 | 0 | 0 |
| Kingsolver | Barbara | The Poisonwood Bible | | | USA | 1 | 1998 | 2 | 1955 | 0 | 0 | 0 | 0 | 0 | 0 | 0 | 0 | 0 | 0 | 0 | 0 | 0 | 0 | 0 | 0 |
| Kinsella | Sophie | I love shopping | | | UK | 1 | 2000 | 1 | 1969 | 0 | 0 | 0 | 0 | 0 | 0 | 0 | 0 | 0 | 0 | 0 | 0 | 0 | 0 | 0 | 0 |
| Kipling | Rudyard | The jungle book | | | UK | 0 | 1894 | 1 | 1865 | 0 | 1 | 0 | 0 | 0 | 0 | 0 | 0 | 0 | 0 | 0 | 0 | 0 | 0 | 0 | 0 |
| Kipling | Rudyard | Captains courageous | | | UK | 0 | 1896 | 1 | 1865 | 0 | 1 | 0 | 0 | 0 | 0 | 0 | 0 | 0 | 0 | 0 | 0 | 0 | 0 | 0 | 0 |
| Kirino | Natsuo | Out | | | JAPAN | 1 | 1997 | 4 | 1951 | 1 | 0 | 0 | 0 | 0 | 0 | 0 | 0 | 0 | 0 | 0 | 0 | 0 | 0 | 0 | 0 |
| Kirino | Natsuo | Grotesque | | | JAPAN | 1 | 2003 | 4 | 1951 | 1 | 0 | 0 | 0 | 0 | 0 | 0 | 0 | 0 | 0 | 0 | 0 | 0 | 0 | 0 | 0 |
| Kirino | Natsuo | Real world | | | JAPAN | 1 | 2003 | 4 | 1951 | 1 | 0 | 0 | 0 | 0 | 0 | 0 | 0 | 0 | 0 | 0 | 0 | 0 | 0 | 0 | 0 |
| Klay | Phil | Redeployment | | | USA | 0 | 2014 | 2 | 1983 | 0 | 0 | 0 | 0 | 0 | 0 | 0 | 0 | 1 | 0 | 0 | 1 | 1 | 0 | 0 | 0 |
| Klíma | Ivan | Love and Garbage | | | CZECH REP. | 0 | 1986 | 1 | 1931 | 1 | 0 | 0 | 0 | 0 | 0 | 0 | 1 | 0 | 0 | 0 | 0 | 0 | 0 | 0 | 0 |
| Knausgård | Karl Ove | My struggle 1 | | | NORWAY | 0 | 2009 | 1 | 1968 | 1 | 0 | 0 | 0 | 0 | 0 | 0 | 0 | 0 | 0 | 0 | 0 | 0 | 0 | 0 | 0 |
| Knausgård | Karl Ove | My struggle 2 | | | NORWAY | 0 | 2010 | 1 | 1968 | 1 | 0 | 0 | 0 | 0 | 0 | 0 | 0 | 0 | 0 | 0 | 0 | 0 | 0 | 0 | 0 |
| Knausgård | Karl Ove | My struggle 3 | | | NORWAY | 0 | 2011 | 1 | 1968 | 1 | 0 | 0 | 0 | 0 | 0 | 0 | 0 | 0 | 0 | 0 | 0 | 0 | 0 | 0 | 0 |
| Kneale | Matthew | English Passengers | | | UK | 0 | 2000 | 1 | 1960 | 0 | 0 | 0 | 0 | 0 | 0 | 0 | 0 | 0 | 0 | 0 | 0 | 0 | 0 | 0 | 0 |
| Koestler | Arthur | Darkness at Noon | | | HUNGARY | 0 | 1940 | 1 | 1905 | 1 | 0 | 0 | 0 | 0 | 0 | 0 | 0 | 0 | 0 | 0 | 0 | 0 | 0 | 0 | 0 |
| Kohan | Martin | School For Patriots | | | ARGENTINA | 0 | 2007 | 3 | 1967 | 1 | 0 | 0 | 0 | 0 | 0 | 0 | 0 | 0 | 0 | 0 | 0 | 0 | 0 | 0 | 0 |
| Koonchung | Chan | Fat Years | | | CHINA | 1 | 2012 | 4 | 1952 | 1 | 0 | 0 | 0 | 0 | 0 | 0 | 0 | 0 | 0 | 0 | 0 | 0 | 0 | 0 | 0 |
| Krakauer | Jon | Into the wild | | | USA | 0 | 1996 | 2 | 1954 | 0 | 0 | 0 | 0 | 0 | 0 | 0 | 0 | 0 | 0 | 0 | 0 | 0 | 0 | 0 | 0 |
| Krien | Daniela | Someday we'll tell each other everything | | | GERMANY | 1 | 2011 | 1 | 1975 | 1 | 0 | 0 | 0 | 0 | 0 | 0 | 0 | 0 | 0 | 0 | 0 | 0 | 0 | 0 | 0 |
| Krishnamurthy | Kalki | Ponniyin Selvan Book 1: Fresh Floods | | | INDIA | 0 | 1951 | 4 | 1899 | 1 | 0 | 0 | 0 | 0 | 0 | 0 | 0 | 0 | 0 | 0 | 0 | 0 | 0 | 0 | 0 |
| Krishnamurthy | Kalki | Ponniyin Selvan Book 2: Whirlwinds | | | INDIA | 0 | 1951 | 4 | 1899 | 1 | 0 | 0 | 0 | 0 | 0 | 0 | 0 | 0 | 0 | 0 | 0 | 0 | 0 | 0 | 0 |
| Kristof | Ágota | The notebook The proof The third lie | | | SWITZERLAND | 1 | 1986 | 1 | 1935 | 1 | 0 | 0 | 0 | 0 | 0 | 0 | 0 | 0 | 0 | 0 | 0 | 0 | 0 | 0 | 0 |
| Kuang | RF | The Poppy War | | | CHINA | 1 | 2018 | 4 | 1996 | 0 | 0 | 0 | 0 | 0 | 0 | 0 | 0 | 0 | 0 | 0 | 0 | 0 | 0 | 0 | 0 |
| Kulin | Ayşe | Last train to Istanbul | | | TURKEY | 1 | 2002 | 4 | 1941 | 1 | 0 | 0 | 0 | 0 | 0 | 0 | 0 | 0 | 0 | 0 | 0 | 0 | 0 | 0 | 0 |
| Kundera | Milan | Laughable loves | | | CZECH REP. | 0 | 1969 | 1 | 1929 | 1 | 0 | 0 | 0 | 0 | 0 | 0 | 1 | 0 | 0 | 0 | 0 | 0 | 0 | 0 | 0 |
| Kundera | Milan | The unbearable lightness of being | | | CZECH REP. | 0 | 1984 | 1 | 1929 | 1 | 0 | 0 | 0 | 0 | 0 | 0 | 1 | 0 | 0 | 0 | 0 | 0 | 0 | 0 | 0 |
| Kunzru | Hari | White tears | | | UK | 0 | 2017 | 1 | 1969 | 0 | 0 | 0 | 0 | 0 | 0 | 0 | 0 | 0 | 0 | 0 | 0 | 0 | 0 | 0 | 0 |
| Kureishi | Hanif | The Buddha of Suburbia | | | UK | 0 | 1990 | 1 | 1954 | 0 | 0 | 0 | 0 | 0 | 0 | 0 | 0 | 0 | 0 | 0 | 0 | 0 | 0 | 0 | 0 |
| Kurniawan | Eka | Beauty is a wound | | | INDONESIA | 0 | 2002 | 4 | 1975 | 1 | 0 | 0 | 0 | 0 | 0 | 0 | 0 | 0 | 0 | 0 | 0 | 0 | 0 | 0 | 0 |
| Kurniawan | Eka | Vengeance is mine | | | INDONESIA | 0 | 2014 | 4 | 1975 | 1 | 0 | 0 | 0 | 0 | 0 | 0 | 0 | 0 | 0 | 0 | 0 | 0 | 0 | 0 | 0 |
| Kuroyanagi | Tetsuko | Totto-Chan: The Little Girl at the Window | | | JAPAN | 1 | 1981 | 4 | 1933 | 1 | 0 | 0 | 0 | 0 | 0 | 0 | 0 | 0 | 0 | 0 | 0 | 0 | 0 | 0 | 0 |
| Kushner | Rachel | The Mars Room | | | USA | 1 | 2018 | 2 | 1968 | 0 | 0 | 0 | 0 | 0 | 0 | 0 | 0 | 0 | 0 | 0 | 0 | 0 | 0 | 0 | 0 |
| Kwan | Kevin | Crazy Rich Asians | | | SINGAPORE | 0 | 2013 | 4 | 1973 | 0 | 0 | 0 | 0 | 0 | 0 | 0 | 0 | 0 | 0 | 0 | 0 | 0 | 0 | 0 | 0 |
| Kyomuhendo | Goretti | Waiting | | | UGANDA | 1 | 2007 | 5 | 1965 | 0 | 0 | 0 | 0 | 0 | 0 | 0 | 0 | 0 | 0 | 0 | 0 | 0 | 0 | 0 | 0 |
| L'Engle | Madeleine | A Wrinkle in Time | | | USA | 1 | 1962 | 2 | 1918 | 0 | 0 | 0 | 0 | 0 | 0 | 0 | 0 | 0 | 0 | 0 | 0 | 0 | 0 | 0 | 0 |
| La Farge | Oliver | Laughing Boy | | | USA | 0 | 1929 | 2 | 1901 | 0 | 0 | 0 | 0 | 1 | 0 | 0 | 0 | 0 | 0 | 0 | 0 | 0 | 0 | 0 | 0 |
| La Motte | Ellen | Civilization | | | USA | 1 | 1919 | 2 | 1873 | 0 | 0 | 0 | 0 | 0 | 0 | 0 | 0 | 0 | 0 | 0 | 0 | 0 | 0 | 0 | 0 |
| La Motte | Ellen | Peking dust | | | USA | 1 | 1919 | 2 | 1873 | 0 | 0 | 0 | 0 | 0 | 0 | 0 | 0 | 0 | 0 | 0 | 0 | 0 | 0 | 0 | 0 |
| La Motte | Ellen | The Backwash of War | | | USA | 1 | 1916 | 2 | 1873 | 0 | 0 | 0 | 0 | 0 | 0 | 0 | 0 | 0 | 0 | 0 | 0 | 0 | 0 | 0 | 0 |
| Labelle | Claude | e Ranger Boys and the Border Smuggl | | | USA | 0 | 1922 | 2 | ? | 0 | 0 | 0 | 0 | 0 | 0 | 0 | 0 | 0 | 0 | 0 | 0 | 0 | 0 | 0 | 0 |
| Labelle | Claude | he Ranger Boys Outwit the Timber Thie | | | USA | 0 | 1922 | 2 | ? | 0 | 0 | 0 | 0 | 0 | 0 | 0 | 0 | 0 | 0 | 0 | 0 | 0 | 0 | 0 | 0 |
| Läckberg | Camilla | The drowning | | | SWEDEN | 1 | 2008 | 1 | 1974 | 1 | 0 | 0 | 0 | 0 | 0 | 0 | 0 | 0 | 0 | 0 | 0 | 0 | 0 | 0 | 0 |
| Laclos | Pierre | Dangerous liaisons | | | FRANCE | 0 | 1782 | 1 | 1741 | 1 | 0 | 0 | 0 | 0 | 0 | 0 | 0 | 0 | 0 | 0 | 0 | 0 | 0 | 0 | 0 |
| Lagercrantz | David | The girl in the spider's web | | | SWEDEN | 0 | 2015 | 1 | 1962 | 1 | 0 | 0 | 0 | 0 | 0 | 0 | 0 | 0 | 0 | 0 | 0 | 0 | 0 | 0 | 0 |
| Lagerkvist | Pär | Barabbas | | | SWEDEN | 0 | 1950 | 1 | 1891 | 1 | 1 | 0 | 0 | 0 | 0 | 0 | 0 | 0 | 0 | 0 | 0 | 0 | 0 | 0 | 0 |
| Lagerkvist | Pär | The dwarf | | | SWEDEN | 0 | 1944 | 1 | 1891 | 1 | 1 | 0 | 0 | 0 | 0 | 0 | 0 | 0 | 0 | 0 | 0 | 0 | 0 | 0 | 0 |
| Lagerlöf | Selma | The Saga of Gosta Berling | | | SWEDEN | 1 | 1891 | 1 | 1858 | 1 | 1 | 0 | 0 | 0 | 0 | 0 | 0 | 0 | 0 | 0 | 0 | 0 | 0 | 0 | 0 |
| Lagioia | Nicola | Ferocity | | | ITALY | 0 | 2014 | 1 | 1973 | 1 | 0 | 0 | 0 | 0 | 0 | 0 | 0 | 0 | 0 | 0 | 0 | 0 | 0 | 0 | 0 |
| Lahiri | Jhumpa | In other words | | | USA | 1 | 2015 | 2 | 1967 | 1 | 0 | 0 | 0 | 0 | 0 | 0 | 0 | 0 | 0 | 0 | 0 | 0 | 0 | 0 | 0 |
| Lahiri | Jhumpa | Interpreter of Maladies | | | USA | 1 | 2000 | 2 | 1967 | 0 | 0 | 0 | 0 | 1 | 0 | 0 | 0 | 0 | 0 | 0 | 0 | 0 | 0 | 0 | 0 |
| Laing | Olivia | The lonely city | | | UK | 1 | 2016 | 1 | 1977 | 0 | 0 | 0 | 0 | 0 | 0 | 0 | 0 | 0 | 0 | 0 | 0 | 0 | 0 | 0 | 0 |
| Lamb | Wally | She's Come Undone | | | USA | 0 | 1992 | 2 | 1950 | 0 | 0 | 0 | 0 | 0 | 0 | 0 | 0 | 0 | 0 | 0 | 0 | 0 | 0 | 0 | 0 |

| **SURNAME** | **NAME** | **TITLE** | **NATIONALITY** | | **GENDER** | **PUB_YEAR** | **CONTINENT** | **DATE OF BIRTH** | **TRANSLATED** | **NOBEL** | **NEUSTADT** | **BOOKER** | **PULITZER** | **NEBULA** | **CAINE** | **FRANZ KAFKA** | **NBA** | **BBA** | **WOMEN'S PRIZE FOR FICTION** | **NEW YORK TIMES** | **THE GUARDIAN** | **THE NEW YORKER** | **BBC** | **ATLANTIC** |
| --- | --- | --- | --- | --- | --- | --- | --- | --- | --- | --- | --- | --- | --- | --- | --- | --- | --- | --- | --- | --- | --- | --- | --- | --- |
| Lambourne | Alfred The Pioneer Trail | | | UK | 0 | 1913 | 1 | 1850 | 0 | 0 | 0 | 0 | 0 | 0 | 0 | 0 | 0 | 0 | 0 | 0 | 0 | 0 | 0 | 0 |
| Lampedusa | iuseppe Tomas The leopard | | | ITALY | 0 | 1958 | 1 | 1896 | 1 | 0 | 0 | 0 | 0 | 0 | 0 | 0 | 0 | 0 | 0 | 0 | 0 | 0 | 0 | 0 |
| Lanchester | John Reality, and other stories | | | UK | 0 | 2020 | 1 | 1962 | 0 | 0 | 0 | 0 | 0 | 0 | 0 | 0 | 0 | 0 | 0 | 0 | 1 | 0 | 0 | 0 |
| Landa | Gertrude Jewish Fairy Tales And Legends | | | USA | 1 | 1943 | 2 | 1892 | 0 | 0 | 0 | 0 | 0 | 0 | 0 | 0 | 0 | 0 | 0 | 0 | 0 | 0 | 0 | 0 |
| Landor | Edward The Bushman | | | UK | 0 | 1847 | 1 | 1811 | 0 | 0 | 0 | 0 | 0 | 0 | 0 | 0 | 0 | 0 | 0 | 0 | 0 | 0 | 0 | 0 |
| Lane | Anna The Champagne Standard | | | UK | 1 | 1905 | 1 | 1856 | 0 | 0 | 0 | 0 | 0 | 0 | 0 | 0 | 0 | 0 | 0 | 0 | 0 | 0 | 0 | 0 |
| Lane | Elinor Katrine | | | USA | 1 | 1909 | 2 | 1864 | 0 | 0 | 0 | 0 | 0 | 0 | 0 | 0 | 0 | 0 | 0 | 0 | 0 | 0 | 0 | 0 |
| Lane | Elinor Nancy Stair | | | USA | 1 | 1904 | 2 | 1864 | 0 | 0 | 0 | 0 | 0 | 0 | 0 | 0 | 0 | 0 | 0 | 0 | 0 | 0 | 0 | 0 |
| Lang | George Pixy's Holiday Journey | | | UK | 0 | 1906 | 1 | ? | 1 | 0 | 0 | 0 | 0 | 0 | 0 | 0 | 0 | 0 | 0 | 0 | 0 | 0 | 0 | 0 |
| Lange | Norah People in the room | | | ARGENTINA | 1 | 1950 | 3 | 1905 | 1 | 0 | 0 | 0 | 0 | 0 | 0 | 0 | 0 | 0 | 0 | 0 | 0 | 0 | 0 | 0 |
| Lange | Dietrich The Lure Of The Mississippi | | | GERMANY | 0 | 1917 | 1 | 1863 | 1 | 0 | 0 | 0 | 0 | 0 | 0 | 0 | 0 | 0 | 0 | 0 | 0 | 0 | 0 | 0 |
| Langworthy | Daniel iscences of a Prisoner of War and His E | | | USA | 0 | 1915 | 2 | 1832 | 0 | 0 | 0 | 0 | 0 | 0 | 0 | 0 | 0 | 0 | 0 | 0 | 0 | 0 | 0 | 0 |
| Lanman | Charles Letters from the Alleghany Mountains | | | USA | 0 | 1849 | 2 | 1819 | 0 | 0 | 0 | 0 | 0 | 0 | 0 | 0 | 0 | 0 | 0 | 0 | 0 | 0 | 0 | 0 |
| Lapena | Shari An unwanted guest | | | CANADA | 1 | 2018 | 2 | 1960 | 0 | 0 | 0 | 0 | 0 | 0 | 0 | 0 | 0 | 0 | 0 | 0 | 0 | 0 | 0 | 0 |
| Larsson | Stieg The girl with the dragon tattoo | | | SWEDEN | 0 | 2005 | 1 | 1954 | 1 | 0 | 0 | 0 | 0 | 0 | 0 | 0 | 0 | 1 | 0 | 0 | 0 | 0 | 0 | 0 |
| Larsson | Stieg The girl who played with fire | | | SWEDEN | 0 | 2006 | 1 | 1954 | 1 | 0 | 0 | 0 | 0 | 0 | 0 | 0 | 0 | 0 | 0 | 0 | 0 | 0 | 0 | 0 |
| Larsson | Stieg The girl who kicked the hornets' nest | | | SWEDEN | 0 | 2007 | 1 | 1954 | 1 | 0 | 0 | 0 | 0 | 0 | 0 | 0 | 0 | 0 | 0 | 0 | 0 | 0 | 0 | 0 |
| Laughlin | Clara Everybody's Lonesome | | | USA | 1 | 1910 | 2 | 1873 | 0 | 0 | 0 | 0 | 0 | 0 | 0 | 0 | 0 | 0 | 0 | 0 | 0 | 0 | 0 | 0 |
| Laughlin | Clara A Life of the Supreme Commander of th | | | USA | 1 | 1918 | 2 | 1873 | 0 | 0 | 0 | 0 | 0 | 0 | 0 | 0 | 0 | 0 | 0 | 0 | 0 | 0 | 0 | 0 |
| Laurain | Antoine The red notebook | | | FRANCE | 0 | 2014 | 1 | 1972 | 1 | 0 | 0 | 0 | 0 | 0 | 0 | 0 | 0 | 0 | 0 | 0 | 0 | 0 | 0 | 0 |
| Laut | Agnes The Freebooters of the Wilderness | | | CANADA | 1 | 1910 | 2 | 1871 | 0 | 0 | 0 | 0 | 0 | 0 | 0 | 0 | 0 | 0 | 0 | 0 | 0 | 0 | 0 | 0 |
| Laut | Agnes The Story of the Trapper | | | CANADA | 1 | 1916 | 2 | 1871 | 0 | 0 | 0 | 0 | 0 | 0 | 0 | 0 | 0 | 0 | 0 | 0 | 0 | 0 | 0 | 0 |
| Lavalle | Victor The Changeling | | | USA | 0 | 2017 | 2 | 1972 | 0 | 0 | 0 | 0 | 0 | 0 | 0 | 0 | 0 | 0 | 0 | 0 | 0 | 0 | 0 | 0 |
| Lavell | Edith The Girl Scouts' Good Turn | | | USA | 1 | 1922 | 2 | 1892 | 0 | 0 | 0 | 0 | 0 | 0 | 0 | 0 | 0 | 0 | 0 | 0 | 0 | 0 | 0 | 0 |
| Lawrence | DH Lady Chatterley's Lover | | | UK | 0 | 1928 | 1 | 1885 | 0 | 0 | 0 | 0 | 0 | 0 | 0 | 0 | 0 | 0 | 0 | 0 | 0 | 0 | 0 | 0 |
| Lawrence | DH Sons and lovers | | | UK | 0 | 1913 | 1 | 1885 | 0 | 0 | 0 | 0 | 0 | 0 | 0 | 0 | 0 | 0 | 0 | 0 | 0 | 0 | 0 | 0 |
| Lawrence | DH Women in Love | | | UK | 0 | 1920 | 1 | 1885 | 0 | 0 | 0 | 0 | 0 | 0 | 0 | 0 | 0 | 0 | 0 | 0 | 0 | 0 | 0 | 0 |
| Laxness | Halldór Iceland's bell | | | ICELAND | 0 | 1943 | 1 | 1902 | 1 | 1 | 0 | 0 | 0 | 0 | 0 | 0 | 0 | 0 | 0 | 0 | 0 | 0 | 0 | 0 |
| Lazell | Frederick Spring Days in Iowa | | | USA | 0 | 1907 | 2 | ? | 0 | 0 | 0 | 0 | 0 | 0 | 0 | 0 | 0 | 0 | 0 | 0 | 0 | 0 | 0 | 0 |
| Lazell | Frederick Summer Days in Iowa | | | USA | 0 | 1909 | 2 | ? | 0 | 0 | 0 | 0 | 0 | 0 | 0 | 0 | 0 | 0 | 0 | 0 | 0 | 0 | 0 | 0 |
| Lazell | Frederick Winter Days in Iowa | | | USA | 0 | 1908 | 2 | ? | 0 | 0 | 0 | 0 | 0 | 0 | 0 | 0 | 0 | 0 | 0 | 0 | 0 | 0 | 0 | 0 |
| Le Clezio | JMG The flood | | | FRANCE | 0 | 1966 | 1 | 1940 | 1 | 1 | 0 | 0 | 0 | 0 | 0 | 0 | 0 | 0 | 0 | 0 | 0 | 0 | 0 | 0 |
| Le Fanu | Sheridan In a Glass Darkly | | | IRELAND | 0 | 1872 | 1 | 1814 | 0 | 0 | 0 | 0 | 0 | 0 | 0 | 0 | 0 | 0 | 0 | 0 | 0 | 0 | 0 | 0 |
| Le Fanu | Sheridan Haunted Lives | | | IRELAND | 0 | 1868 | 1 | 1814 | 0 | 0 | 0 | 0 | 0 | 0 | 0 | 0 | 0 | 0 | 0 | 0 | 0 | 0 | 0 | 0 |
| Le Fanu | Sheridan The House by the Churchyard | | | IRELAND | 0 | 1863 | 1 | 1814 | 0 | 0 | 0 | 0 | 0 | 0 | 0 | 0 | 0 | 0 | 0 | 0 | 0 | 0 | 0 | 0 |
| Le Fanu | Sheridan Uncle Silas | | | IRELAND | 0 | 1864 | 1 | 1814 | 0 | 0 | 0 | 0 | 0 | 0 | 0 | 0 | 0 | 0 | 0 | 0 | 0 | 0 | 0 | 0 |
| Le Fanu | Sheridan Willing to Die | | | IRELAND | 0 | 1872 | 1 | 1814 | 0 | 0 | 0 | 0 | 0 | 0 | 0 | 0 | 0 | 0 | 0 | 0 | 0 | 0 | 0 | 0 |
| Le Guin | Ursula The Lathe of Heaven | | | USA | 1 | 1971 | 2 | 1929 | 0 | 0 | 0 | 0 | 0 | 0 | 0 | 0 | 0 | 0 | 0 | 0 | 0 | 0 | 0 | 0 |
| Le Guin | Ursula Earthsea trilogy | | | USA | 1 | 1964 | 2 | 1929 | 0 | 0 | 0 | 0 | 0 | 0 | 0 | 0 | 0 | 0 | 0 | 0 | 0 | 0 | 0 | 0 |
| Le Guin | Ursula The Left Hand of Darkness | | | USA | 1 | 1969 | 2 | 1929 | 0 | 0 | 0 | 0 | 0 | 1 | 0 | 0 | 0 | 0 | 0 | 0 | 0 | 0 | 0 | 0 |
| Le Queux | William The Mystery Of The Green Ray | | | UK | 0 | 1915 | 1 | 1864 | 0 | 0 | 0 | 0 | 0 | 0 | 0 | 0 | 0 | 0 | 0 | 0 | 0 | 0 | 0 | 0 |
| Le Queux | William The Great White Queen | | | UK | 0 | 1896 | 1 | 1864 | 0 | 0 | 0 | 0 | 0 | 0 | 0 | 0 | 0 | 0 | 0 | 0 | 0 | 0 | 0 | 0 |
| Leamy | Edmund Irish Fairy Tales | | | IRELAND | 0 | 1906 | 1 | 1848 | 0 | 0 | 0 | 0 | 0 | 0 | 0 | 0 | 0 | 0 | 0 | 0 | 0 | 0 | 0 | 0 |
| Leavitt | David Family dancing | | | USA | 0 | 1984 | 2 | 1961 | 0 | 0 | 0 | 0 | 0 | 0 | 0 | 0 | 0 | 0 | 0 | 0 | 0 | 0 | 0 | 0 |
| Leavitt | David The lost language of cranes | | | USA | 0 | 1986 | 2 | 1961 | 0 | 0 | 0 | 0 | 0 | 0 | 0 | 0 | 0 | 0 | 0 | 0 | 0 | 0 | 0 | 0 |
| LeBlanc | Maurice The Confessions of Arsene Lupin | | | FRANCE | 0 | 1913 | 1 | 1864 | 1 | 0 | 0 | 0 | 0 | 0 | 0 | 0 | 0 | 0 | 0 | 0 | 0 | 0 | 0 | 0 |
| LeBlanc | Maurice Arsène Lupin versus Herlock Sholmes | | | FRANCE | 0 | 1908 | 1 | 1864 | 1 | 0 | 0 | 0 | 0 | 0 | 0 | 0 | 0 | 0 | 0 | 0 | 0 | 0 | 0 | 0 |
| Lee | Harper To kill a mockingbird | | | USA | 1 | 1960 | 2 | 1926 | 0 | 0 | 0 | 0 | 1 | 0 | 0 | 0 | 0 | 0 | 0 | 0 | 0 | 0 | 1 | 0 |
| Lee | Jung-Myung The investigation | | | SOUTH KOREA | 0 | 2016 | 4 | 1965 | 1 | 0 | 0 | 0 | 0 | 0 | 0 | 0 | 0 | 0 | 0 | 0 | 0 | 0 | 0 | 0 |
| Lee | Krys How I became a north korean | | | SOUTH KOREA | 1 | 2017 | 4 | ? | 0 | 0 | 0 | 0 | 0 | 0 | 0 | 0 | 0 | 0 | 0 | 0 | 0 | 0 | 0 | 0 |
| Lee | Day Summerfield | | | USA | 0 | 1852 | 2 | 1816 | 0 | 0 | 0 | 0 | 0 | 0 | 0 | 0 | 0 | 0 | 0 | 0 | 0 | 0 | 0 | 0 |
| Lee | Hannah Rich Enough | | | USA | 0 | 1837 | 2 | 1780 | 0 | 0 | 0 | 0 | 0 | 0 | 0 | 0 | 0 | 0 | 0 | 0 | 0 | 0 | 0 | 0 |
| Lee | Holme The Vicissitudes of Bessie Fairfax | | | UK | 1 | 1874 | 1 | 1828 | 0 | 0 | 0 | 0 | 0 | 0 | 0 | 0 | 0 | 0 | 0 | 0 | 0 | 0 | 0 | 0 |
| Lee | Chang-rae The Surrendered | | | USA | 0 | 2010 | 2 | 1965 | 0 | 0 | 0 | 0 | 0 | 0 | 0 | 0 | 0 | 0 | 0 | 0 | 0 | 0 | 0 | 0 |
| LeFeuvre | Amy Bulbs and Blossoms | | | UK | 1 | 1898 | 1 | 1861 | 0 | 0 | 0 | 0 | 0 | 0 | 0 | 0 | 0 | 0 | 0 | 0 | 0 | 0 | 0 | 0 |
| LeFeuvre | Amy Dwell Deep or Hilda Thorn's Life Story | | | UK | 1 | 1896 | 1 | 1861 | 0 | 0 | 0 | 0 | 0 | 0 | 0 | 0 | 0 | 0 | 0 | 0 | 0 | 0 | 0 | 0 |
| LeFeuvre | Amy His Big Opportunity | | | UK | 1 | 1898 | 1 | 1861 | 0 | 0 | 0 | 0 | 0 | 0 | 0 | 0 | 0 | 0 | 0 | 0 | 0 | 0 | 0 | 0 |
| LeFeuvre | Amy Jill's Red Bag | | | UK | 1 | 1903 | 1 | 1861 | 0 | 0 | 0 | 0 | 0 | 0 | 0 | 0 | 0 | 0 | 0 | 0 | 0 | 0 | 0 | 0 |
| LeFeuvre | Amy Probable Sons | | | UK | 1 | 1896 | 1 | 1861 | 0 | 0 | 0 | 0 | 0 | 0 | 0 | 0 | 0 | 0 | 0 | 0 | 0 | 0 | 0 | 0 |
| LeFeuvre | Amy Teddy's Button | | | UK | 1 | 1896 | 1 | 1861 | 0 | 0 | 0 | 0 | 0 | 0 | 0 | 0 | 0 | 0 | 0 | 0 | 0 | 0 | 0 | 0 |
| LeFeuvre | Amy The Carved Cupboard | | | UK | 1 | 1899 | 1 | 1861 | 0 | 0 | 0 | 0 | 0 | 0 | 0 | 0 | 0 | 0 | 0 | 0 | 0 | 0 | 0 | 0 |
| Lehane | Dennis Mystic river | | | USA | 0 | 2001 | 2 | 1965 | 0 | 0 | 0 | 0 | 0 | 0 | 0 | 0 | 0 | 0 | 0 | 0 | 0 | 0 | 0 | 0 |
| Lemebel | Pedro My tender matador | | | CHILE | 0 | 2001 | 3 | 1952 | 1 | 0 | 0 | 0 | 0 | 0 | 0 | 0 | 0 | 0 | 0 | 0 | 0 | 0 | 0 | 0 |
| Lermontov | Mikhail A Hero of Our Time | | | RUSSIA | 0 | 1840 | 1 | 1814 | 1 | 0 | 0 | 0 | 0 | 0 | 0 | 0 | 0 | 0 | 0 | 0 | 0 | 0 | 0 | 0 |
| Lerner | Ben The Topeka School | | | USA | 0 | 2019 | 2 | 1979 | 0 | 0 | 0 | 0 | 0 | 0 | 0 | 0 | 0 | 0 | 0 | 1 | 1 | 0 | 0 | 1 |
| Lerrigo | Charles The Boy Scout Treasure Hunters | | | USA | 0 | 1917 | 2 | 1872 | 0 | 0 | 0 | 0 | 0 | 0 | 0 | 0 | 0 | 0 | 0 | 0 | 0 | 0 | 0 | 0 |
| Leskov | Nikolai The Enchanted Wanderer | | | RUSSIA | 0 | 1873 | 1 | 1831 | 1 | 0 | 0 | 0 | 0 | 0 | 0 | 0 | 0 | 0 | 0 | 0 | 0 | 0 | 0 | 0 |
| Leslie | Emma Kate's Ordeal | | | UK | 1 | 1887 | 1 | 1838 | 0 | 0 | 0 | 0 | 0 | 0 | 0 | 0 | 0 | 0 | 0 | 0 | 0 | 0 | 0 | 0 |
| Lessing | Doris Briefing for a Descent Into Hell | | | UK | 1 | 1971 | 1 | 1919 | 0 | 1 | 0 | 0 | 0 | 0 | 0 | 0 | 0 | 0 | 0 | 0 | 0 | 0 | 0 | 0 |
| Lessing | Doris The fifth child | | | UK | 1 | 1988 | 1 | 1919 | 0 | 1 | 0 | 0 | 0 | 0 | 0 | 0 | 0 | 0 | 0 | 0 | 0 | 0 | 0 | 0 |
| Lessing | Doris The sweetest dream | | | UK | 1 | 2001 | 1 | 1919 | 0 | 1 | 0 | 0 | 0 | 0 | 0 | 0 | 0 | 0 | 0 | 0 | 0 | 0 | 0 | 0 |
| Lethem | Jonathan The fortress of solitude | | | USA | 0 | 2003 | 2 | 1965 | 0 | 0 | 0 | 0 | 0 | 0 | 0 | 0 | 0 | 0 | 0 | 0 | 0 | 0 | 0 | 0 |
| Letts | Billie Where the Heart Is | | | USA | 0 | 1995 | 2 | 1938 | 0 | 0 | 0 | 0 | 0 | 0 | 0 | 0 | 0 | 0 | 0 | 0 | 0 | 0 | 0 | 0 |
| Levi | Primo The periodic table | | | ITALY | 0 | 1975 | 1 | 1919 | 1 | 0 | 0 | 0 | 0 | 0 | 0 | 0 | 0 | 0 | 0 | 0 | 0 | 0 | 0 | 0 |
| Levi | Primo A tranquil star | | | ITALY | 0 | 1971 | 1 | 1919 | 1 | 0 | 0 | 0 | 0 | 0 | 0 | 0 | 0 | 0 | 0 | 0 | 0 | 0 | 0 | 0 |
| Levi | Primo If this is a man | | | ITALY | 0 | 1947 | 1 | 1919 | 1 | 0 | 0 | 0 | 0 | 0 | 0 | 0 | 0 | 0 | 0 | 0 | 0 | 0 | 0 | 0 |
| Levin | Ira Rosemary's baby | | | USA | 0 | 1967 | 2 | 1929 | 0 | 0 | 0 | 0 | 0 | 0 | 0 | 0 | 0 | 0 | 0 | 0 | 0 | 0 | 0 | 0 |
| Levin | Ira The stepford wives | | | USA | 0 | 1972 | 2 | 1929 | 0 | 0 | 0 | 0 | 0 | 0 | 0 | 0 | 0 | 0 | 0 | 0 | 0 | 0 | 0 | 0 |
| Levy | Andrea The Long Song | | | UK | 0 | 2010 | 1 | 1956 | 0 | 0 | 0 | 0 | 0 | 0 | 0 | 0 | 0 | 0 | 0 | 0 | 0 | 0 | 0 | 0 |
| Levy | Deborah Swimming Home | | | SOUTH AFRICA | 1 | 2011 | 5 | 1959 | 0 | 0 | 0 | 0 | 0 | 0 | 0 | 0 | 0 | 0 | 0 | 0 | 0 | 0 | 0 | 0 |
| Lewis | Clive Staples The lion the witch and the wardrobe | | | UK | 0 | 1950 | 1 | 1898 | 0 | 0 | 0 | 0 | 0 | 0 | 0 | 0 | 0 | 0 | 0 | 0 | 0 | 0 | 0 | 0 |
| Lewis | Roy The evolution man | | | UK | 0 | 1960 | 1 | 1913 | 0 | 0 | 0 | 0 | 0 | 0 | 0 | 0 | 0 | 0 | 0 | 0 | 0 | 0 | 0 | 0 |
| Lewis | Charlton Gawayne And The Green Knight | | | USA | 0 | 1916 | 2 | 1834 | 0 | 0 | 0 | 0 | 0 | 0 | 0 | 0 | 0 | 0 | 0 | 0 | 0 | 0 | 0 | 0 |
| Lewis | Sinclair Arrowsmith | | | USA | 0 | 1925 | 2 | 1885 | 0 | 1 | 0 | 0 | 1 | 0 | 0 | 0 | 0 | 0 | 0 | 0 | 0 | 0 | 0 | 0 |
| Lewis | Sinclair Main street | | | USA | 0 | 1920 | 2 | 1885 | 0 | 1 | 0 | 0 | 0 | 0 | 0 | 0 | 0 | 0 | 0 | 0 | 0 | 0 | 0 | 0 |
| Li | Yiyun Kinder Than Solitude | | | CHINA | 1 | 2014 | 4 | 1972 | 0 | 0 | 0 | 0 | 0 | 0 | 0 | 0 | 0 | 0 | 0 | 0 | 0 | 0 | 0 | 0 |
| Li | Yiyun Gold Boy, Emerald Girl | | | CHINA | 1 | 2010 | 4 | 1972 | 0 | 0 | 0 | 0 | 0 | 0 | 0 | 0 | 0 | 0 | 0 | 0 | 1 | 0 | 0 | 0 |

| **SURNAME** | **NAME** | **TITLE** | **NATIONALITY** | | **GENDER** | **PUB_YEAR** | **CONTINENT** | **DATE OF BIRTH** | **TRANSLATED** | **NOBEL** | **NEUSTADT** | **BOOKER** | **PULITZER** | **NEBULA** | **CAINE** | **FRANZ KAFKA** | **NBA** | **BBA** | **WOMEN'S PRIZE FOR FICTION** | **NEW YORK TIMES** | **THE GUARDIAN** | **THE NEW YORKER** | **BBC** | **ATLANTIC** |
| --- | --- | --- | --- | --- | --- | --- | --- | --- | --- | --- | --- | --- | --- | --- | --- | --- | --- | --- | --- | --- | --- | --- | --- | --- |
| Lianke | Yan | Dream of Ding Village | | CHINA | 0 | 2005 | 4 | 1958 | 1 | 0 | 0 | 0 | 0 | 0 | 0 | 1 | 0 | 0 | 0 | 0 | 0 | 0 | 0 | 0 |
| Lianke | Yan | Lenin's Kisses | | CHINA | 0 | 2004 | 4 | 1958 | 1 | 0 | 0 | 0 | 0 | 0 | 0 | 1 | 0 | 0 | 0 | 0 | 0 | 0 | 0 | 0 |
| Lianke | Yan | Serve the People! | | CHINA | 0 | 2005 | 4 | 1958 | 1 | 0 | 0 | 0 | 0 | 0 | 0 | 1 | 0 | 0 | 0 | 0 | 0 | 0 | 0 | 0 |
| Lilin | Nicolai | Siberian education | | MOLDOVA | 0 | 2009 | 1 | 1980 | 1 | 0 | 0 | 0 | 0 | 0 | 0 | 0 | 0 | 0 | 0 | 0 | 0 | 0 | 0 | 0 |
| Lim | uchen Christin | The Man Who Wore His Wife's Sarong | | MALAYSIA | 1 | 2017 | 4 | 1948 | 0 | 0 | 0 | 0 | 0 | 0 | 0 | 0 | 0 | 0 | 0 | 0 | 0 | 0 | 0 | 0 |
| Ling | Ma | Severance | | CHINA | 1 | 2018 | 4 | 1983 | 0 | 0 | 0 | 0 | 0 | 0 | 0 | 0 | 0 | 0 | 0 | 0 | 0 | 0 | 0 | 0 |
| Link | Charlotte | The rose gardener | | GERMANY | 1 | 2000 | 1 | 1963 | 1 | 0 | 0 | 0 | 0 | 0 | 0 | 0 | 0 | 0 | 0 | 0 | 0 | 0 | 0 | 0 |
| Lispector | Clarice | The stream of life | | BRAZIL | 1 | 1973 | 3 | 1920 | 1 | 0 | 0 | 0 | 0 | 0 | 0 | 0 | 0 | 0 | 0 | 0 | 0 | 0 | 0 | 0 |
| Lispector | Clarice | Near to the Wild Heart | | BRAZIL | 1 | 1943 | 3 | 1920 | 1 | 0 | 0 | 0 | 0 | 0 | 0 | 0 | 0 | 0 | 0 | 0 | 0 | 0 | 0 | 0 |
| Lispector | Clarice | The Passion According to G.H. | | BRAZIL | 1 | 1964 | 3 | 1920 | 1 | 0 | 0 | 0 | 0 | 0 | 0 | 0 | 0 | 0 | 0 | 0 | 0 | 0 | 0 | 0 |
| Litchfield | Grace | Only an Incident | | USA | 1 | 1883 | 2 | 1849 | 0 | 0 | 0 | 0 | 0 | 0 | 0 | 0 | 0 | 0 | 0 | 0 | 0 | 0 | 0 | 0 |
| Litsey | Edwin | The Love Story Of Abner Stone | | USA | 0 | 1902 | 2 | 1874 | 0 | 0 | 0 | 0 | 0 | 0 | 0 | 0 | 0 | 0 | 0 | 0 | 0 | 0 | 0 | 0 |
| Lively | Penelope | Moon Tiger | | EGYPT | 1 | 1978 | 5 | 1933 | 0 | 0 | 0 | 1 | 0 | 0 | 0 | 0 | 0 | 0 | 0 | 0 | 0 | 0 | 0 | 0 |
| Lively | Penelope | According to Mark | | EGYPT | 1 | 1984 | 5 | 1933 | 0 | 0 | 0 | 0 | 0 | 0 | 0 | 0 | 0 | 0 | 0 | 0 | 0 | 0 | 0 | 0 |
| Livermore | George | Take it from dad | | USA | 0 | 1920 | 2 | 1809 | 0 | 0 | 0 | 0 | 0 | 0 | 0 | 0 | 0 | 0 | 0 | 0 | 0 | 0 | 0 | 0 |
| Locke | David | Nasby in Exile | | USA | 0 | 1882 | 2 | 1833 | 0 | 0 | 0 | 0 | 0 | 0 | 0 | 0 | 0 | 0 | 0 | 0 | 0 | 0 | 0 | 0 |
| Lockhart | Caroline | Me Smith | | USA | 1 | 1911 | 2 | 1871 | 0 | 0 | 0 | 0 | 0 | 0 | 0 | 0 | 0 | 0 | 0 | 0 | 0 | 0 | 0 | 0 |
| Lockhart | Caroline | The Dude Wrangler | | USA | 1 | 1921 | 2 | 1871 | 0 | 0 | 0 | 0 | 0 | 0 | 0 | 0 | 0 | 0 | 0 | 0 | 0 | 0 | 0 | 0 |
| Lockhart | Caroline | The Fighting Shepherdess | | USA | 1 | 1919 | 2 | 1871 | 0 | 0 | 0 | 0 | 0 | 0 | 0 | 0 | 0 | 0 | 0 | 0 | 0 | 0 | 0 | 0 |
| Lockhart | Caroline | The Lady Doc | | USA | 1 | 1912 | 2 | 1871 | 0 | 0 | 0 | 0 | 0 | 0 | 0 | 0 | 0 | 0 | 0 | 0 | 0 | 0 | 0 | 0 |
| Lockhart | Caroline | The Man from the Bitter Roots | | USA | 1 | 1915 | 2 | 1871 | 0 | 0 | 0 | 0 | 0 | 0 | 0 | 0 | 0 | 0 | 0 | 0 | 0 | 0 | 0 | 0 |
| Lodge | David | Small World | | UK | 0 | 1984 | 1 | 1935 | 0 | 0 | 0 | 0 | 0 | 0 | 0 | 0 | 0 | 0 | 0 | 0 | 0 | 0 | 0 | 0 |
| Loe | Erlend | Doppler | | NORWAY | 0 | 2004 | 1 | 1969 | 1 | 0 | 0 | 0 | 0 | 0 | 0 | 0 | 0 | 0 | 0 | 0 | 0 | 0 | 0 | 0 |
| Lofts | Norah | The Concubine | | UK | 1 | 1963 | 1 | 1904 | 0 | 0 | 0 | 0 | 0 | 0 | 0 | 0 | 0 | 0 | 0 | 0 | 0 | 0 | 0 | 0 |
| Lokko | Lesley | One secret summer | | GHANA | 1 | 2010 | 5 | 1964 | 0 | 0 | 0 | 0 | 0 | 0 | 0 | 0 | 0 | 0 | 0 | 0 | 0 | 0 | 0 | 0 |
| London | Jack | The star rover | | USA | 0 | 1915 | 2 | 1876 | 0 | 0 | 0 | 0 | 0 | 0 | 0 | 0 | 0 | 0 | 0 | 0 | 0 | 0 | 0 | 0 |
| London | Jack | White fang | | USA | 0 | 1906 | 2 | 1876 | 0 | 0 | 0 | 0 | 0 | 0 | 0 | 0 | 0 | 0 | 0 | 0 | 0 | 0 | 0 | 0 |
| London | Jack | The call of the wild | | USA | 0 | 1904 | 2 | 1876 | 0 | 0 | 0 | 0 | 0 | 0 | 0 | 0 | 0 | 0 | 0 | 0 | 0 | 0 | 0 | 0 |
| Long | Helen | How Janice Day Won | | USA | 1 | 1917 | 2 | ? | 0 | 0 | 0 | 0 | 0 | 0 | 0 | 0 | 0 | 0 | 0 | 0 | 0 | 0 | 0 | 0 |
| Long | Helen | Janice Day at Poketown | | USA | 1 | 1914 | 2 | ? | 0 | 0 | 0 | 0 | 0 | 0 | 0 | 0 | 0 | 0 | 0 | 0 | 0 | 0 | 0 | 0 |
| Long | Helen | The Mission of Janice Day | | USA | 1 | 1917 | 2 | ? | 0 | 0 | 0 | 0 | 0 | 0 | 0 | 0 | 0 | 0 | 0 | 0 | 0 | 0 | 0 | 0 |
| Longstreet | Helen | Lee and Longstreet at High Tide | | USA | 1 | 1904 | 2 | 1863 | 0 | 0 | 0 | 0 | 0 | 0 | 0 | 0 | 0 | 0 | 0 | 0 | 0 | 0 | 0 | 0 |
| Loomis | Charles | Just Irish | | USA | 0 | 1911 | 2 | 1861 | 0 | 0 | 0 | 0 | 0 | 0 | 0 | 0 | 0 | 0 | 0 | 0 | 0 | 0 | 0 | 0 |
| Looms | George | Stubble | | USA | 0 | 1922 | 2 | ? | 0 | 0 | 0 | 0 | 0 | 0 | 0 | 0 | 0 | 0 | 0 | 0 | 0 | 0 | 0 | 0 |
| Lord | Karen | Redemption in Indigo | | BARBADOS | 1 | 2010 | 3 | 1968 | 0 | 0 | 0 | 0 | 0 | 0 | 0 | 0 | 0 | 0 | 0 | 0 | 0 | 0 | 0 | 0 |
| Lord | Karen | The Best of All Possible Worlds | | BARBADOS | 1 | 2013 | 3 | 1968 | 0 | 0 | 0 | 0 | 0 | 0 | 0 | 0 | 0 | 0 | 0 | 0 | 0 | 0 | 0 | 0 |
| Lorimer | George | ters from a Self-Made Merchant to His S | | USA | 0 | 1906 | 2 | 1867 | 0 | 0 | 0 | 0 | 0 | 0 | 0 | 0 | 0 | 0 | 0 | 0 | 0 | 0 | 0 | 0 |
| Loring | Emilie | The Trail of Conflict | | USA | 1 | 1922 | 2 | 1864 | 0 | 0 | 0 | 0 | 0 | 0 | 0 | 0 | 0 | 0 | 0 | 0 | 0 | 0 | 0 | 0 |
| Lott | Bret | Jewel | | USA | 0 | 1991 | 2 | 1958 | 0 | 0 | 0 | 0 | 0 | 0 | 0 | 0 | 0 | 0 | 0 | 0 | 0 | 0 | 0 | 0 |
| Lovecraft | HP | The call of Cthulhu | | USA | 0 | 1928 | 2 | 1890 | 0 | 0 | 0 | 0 | 0 | 0 | 0 | 0 | 0 | 0 | 0 | 0 | 0 | 0 | 0 | 0 |
| Lovecraft | HP | At the mountains of madness | | USA | 0 | 1936 | 2 | 1890 | 0 | 0 | 0 | 0 | 0 | 0 | 0 | 0 | 0 | 0 | 0 | 0 | 0 | 0 | 0 | 0 |
| Lovelace | Earl | Is Just a Movie | | TRINIDAD | 0 | 2011 | 3 | 1935 | 0 | 0 | 0 | 0 | 0 | 0 | 0 | 0 | 0 | 0 | 0 | 0 | 0 | 0 | 0 | 0 |
| Lowe | Clara | God's Answers | | CANADA | 1 | 1882 | 2 | ? | 0 | 0 | 0 | 0 | 0 | 0 | 0 | 0 | 0 | 0 | 0 | 0 | 0 | 0 | 0 | 0 |
| Lowry | Malcolm | Under the volcano | | UK | 0 | 1947 | 1 | 1909 | 0 | 0 | 0 | 0 | 0 | 0 | 0 | 0 | 0 | 0 | 0 | 0 | 0 | 0 | 0 | 0 |
| Lowry | Lois | The Giver | | USA | 1 | 1993 | 2 | 1937 | 0 | 0 | 0 | 0 | 0 | 0 | 0 | 0 | 0 | 0 | 0 | 0 | 0 | 0 | 0 | 0 |
| Lowry | Edward | ' Brigade from Bloemfontein to Koomati | | UK | 0 | 1902 | 1 | 1876 | 0 | 0 | 0 | 0 | 0 | 0 | 0 | 0 | 0 | 0 | 0 | 0 | 0 | 0 | 0 | 0 |
| Ludovici | Anthony | Too Old for Dolls | | UK | 0 | 1921 | 1 | 1882 | 0 | 0 | 0 | 0 | 0 | 0 | 0 | 0 | 0 | 0 | 0 | 0 | 0 | 0 | 0 | 0 |
| Luiselli | Valeria | Lost children archive | | MEXICO | 1 | 2019 | 3 | 1983 | 0 | 0 | 0 | 0 | 0 | 0 | 0 | 0 | 0 | 0 | 0 | 1 | 1 | 0 | 0 | 1 |
| Lurie | Alison | Foreign Affairs | | USA | 1 | 1984 | 2 | 1926 | 0 | 0 | 0 | 0 | 1 | 0 | 0 | 0 | 0 | 0 | 0 | 0 | 0 | 0 | 0 | 0 |
| Lustig | Arnošt | Lovely Green Eyes | | CZECH REP. | 0 | 2000 | 1 | 1926 | 1 | 0 | 0 | 0 | 0 | 0 | 0 | 1 | 0 | 0 | 0 | 0 | 0 | 0 | 0 | 0 |
| Lyle | Eugene | The Missourian | | USA | 0 | 1905 | 2 | 1873 | 0 | 0 | 0 | 0 | 0 | 0 | 0 | 0 | 0 | 0 | 0 | 0 | 0 | 0 | 0 | 0 |
| Lynde | Francis | A Romance in Transit | | USA | 0 | 1897 | 2 | 1856 | 0 | 0 | 0 | 0 | 0 | 0 | 0 | 0 | 0 | 0 | 0 | 0 | 0 | 0 | 0 | 0 |
| Lynde | Francis | The Grafters | | USA | 0 | 1904 | 2 | 1856 | 0 | 0 | 0 | 0 | 0 | 0 | 0 | 0 | 0 | 0 | 0 | 0 | 0 | 0 | 0 | 0 |
| Lynde | Francis | The Quickening | | USA | 0 | 1906 | 2 | 1856 | 0 | 0 | 0 | 0 | 0 | 0 | 0 | 0 | 0 | 0 | 0 | 0 | 0 | 0 | 0 | 0 |
| Lynde | Francis | The Taming of Red Butte Western | | USA | 0 | 1916 | 2 | 1856 | 0 | 0 | 0 | 0 | 0 | 0 | 0 | 0 | 0 | 0 | 0 | 0 | 0 | 0 | 0 | 0 |
| Maalouf | Amin | Balthasar's Odyssey | | LEBANON | 0 | 2000 | 4 | 1949 | 1 | 0 | 0 | 0 | 0 | 0 | 0 | 0 | 0 | 0 | 0 | 0 | 0 | 0 | 0 | 0 |
| Maarouf | Mazen | Jokes for the Gunmen | | PALESTINE | 0 | 2019 | 4 | 1978 | 1 | 0 | 0 | 0 | 0 | 0 | 0 | 0 | 0 | 0 | 0 | 0 | 0 | 0 | 0 | 0 |
| Mabanckou | Alain | Broken glass | | CONGO | 0 | 2005 | 5 | 1966 | 1 | 0 | 0 | 0 | 0 | 0 | 0 | 0 | 0 | 0 | 0 | 0 | 0 | 0 | 0 | 0 |
| Mabanckou | Alain | Memoirs of a porcupine | | CONGO | 0 | 2006 | 5 | 1966 | 1 | 0 | 0 | 0 | 0 | 0 | 0 | 0 | 0 | 0 | 0 | 0 | 0 | 0 | 0 | 0 |
| Mabanckou | Alain | Black moses | | CONGO | 0 | 2015 | 5 | 1966 | 1 | 0 | 0 | 0 | 0 | 0 | 0 | 0 | 0 | 0 | 0 | 0 | 0 | 0 | 0 | 0 |
| Mabanckou | Alain | African psycho | | CONGO | 0 | 2003 | 5 | 1966 | 1 | 0 | 0 | 0 | 0 | 0 | 0 | 0 | 0 | 0 | 0 | 0 | 0 | 0 | 0 | 0 |
| MacDonald | Ann-Marie | Fall on Your Knees | | CANADA | 1 | 1996 | 2 | 1958 | 0 | 0 | 0 | 0 | 0 | 0 | 0 | 0 | 0 | 0 | 0 | 0 | 0 | 0 | 0 | 0 |
| Machen | Arthur | The shining pyramid | | UK | 0 | 1895 | 1 | 1863 | 0 | 0 | 0 | 0 | 0 | 0 | 0 | 0 | 0 | 0 | 0 | 0 | 0 | 0 | 0 | 0 |
| MacKinlay | Kantor | Andersonville | | USA | 0 | 1955 | 2 | 1904 | 0 | 0 | 0 | 0 | 1 | 0 | 0 | 0 | 0 | 0 | 0 | 0 | 0 | 0 | 0 | 0 |
| MacLaverty | Bernard | Midwinter break | | IRELAND | 0 | 2017 | 1 | 1942 | 0 | 0 | 0 | 0 | 0 | 0 | 0 | 0 | 0 | 0 | 0 | 0 | 1 | 0 | 0 | 0 |
| MacLaverty | Bernard | Grace Notes | | IRELAND | 0 | 1997 | 1 | 1942 | 0 | 0 | 0 | 0 | 0 | 0 | 0 | 0 | 0 | 0 | 0 | 0 | 0 | 0 | 0 | 0 |
| MacLeod | Alistair | No Great Mischief | | CANADA | 0 | 1999 | 2 | 1936 | 0 | 0 | 0 | 0 | 0 | 0 | 0 | 0 | 0 | 0 | 0 | 0 | 0 | 0 | 0 | 0 |
| MacNeil | Kevin | The Brilliant & Forever | | UK | 0 | 2016 | 1 | 1972 | 0 | 0 | 0 | 0 | 0 | 0 | 0 | 0 | 0 | 0 | 0 | 0 | 1 | 0 | 0 | 0 |
| Magris | Claudio | A different sea | | ITALY | 0 | 1991 | 1 | 1939 | 1 | 0 | 0 | 0 | 0 | 0 | 0 | 1 | 0 | 0 | 0 | 0 | 0 | 0 | 0 | 0 |
| Mahajan | Karan | The Association of Small Bombs | | INDIA | 0 | 2016 | 4 | 1984 | 0 | 0 | 0 | 0 | 0 | 0 | 0 | 0 | 0 | 0 | 0 | 1 | 0 | 0 | 0 | 0 |
| Mahfuz | Nagib | Palace walk | | EGYPT | 0 | 1956 | 5 | 1911 | 1 | 1 | 0 | 0 | 0 | 0 | 0 | 0 | 0 | 0 | 0 | 0 | 0 | 0 | 0 | 0 |
| Mahfuz | Nagib | Palace desire | | EGYPT | 0 | 1957 | 5 | 1911 | 1 | 1 | 0 | 0 | 0 | 0 | 0 | 0 | 0 | 0 | 0 | 0 | 0 | 0 | 0 | 0 |
| Mahfuz | Nagib | Sugar street | | EGYPT | 0 | 1957 | 5 | 1911 | 1 | 1 | 0 | 0 | 0 | 0 | 0 | 0 | 0 | 0 | 0 | 0 | 0 | 0 | 0 | 0 |
| Mai Que | Nguyễn Phan | The mountains sing | | VIETNAM | 1 | 2020 | 4 | 1973 | 0 | 0 | 0 | 0 | 0 | 0 | 0 | 0 | 0 | 0 | 0 | 0 | 0 | 0 | 0 | 0 |
| Mailer | Norman | The naked and the dead | | USA | 0 | 1948 | 2 | 1923 | 0 | 0 | 0 | 0 | 0 | 0 | 0 | 0 | 0 | 0 | 0 | 0 | 0 | 0 | 0 | 0 |
| Mailer | Norman | Ancient evenings | | USA | 0 | 1983 | 2 | 1923 | 0 | 0 | 0 | 0 | 0 | 0 | 0 | 0 | 0 | 0 | 0 | 0 | 0 | 0 | 0 | 0 |
| Mailer | Norman | The Executioner's Song | | USA | 0 | 1979 | 2 | 1923 | 0 | 0 | 0 | 0 | 1 | 0 | 0 | 0 | 0 | 0 | 0 | 0 | 0 | 0 | 0 | 0 |
| Majumdar | Megha | A burning | | INDIA | 1 | 2020 | 4 | 1987 | 0 | 0 | 0 | 0 | 0 | 0 | 0 | 0 | 0 | 0 | 0 | 0 | 0 | 0 | 0 | 1 |
| Makumbi | nsubuga Jenni | Kintu | | UGANDA | 1 | 2014 | 5 | ? | 0 | 0 | 0 | 0 | 0 | 0 | 0 | 0 | 0 | 0 | 0 | 0 | 0 | 0 | 0 | 0 |
| Malamud | Bernard | The assistant | | USA | 0 | 1957 | 2 | 1914 | 0 | 0 | 0 | 0 | 0 | 0 | 0 | 0 | 0 | 0 | 0 | 0 | 0 | 0 | 0 | 0 |
| Malouf | David | Ransom | | AUSTRALIA | 0 | 2009 | 6 | 1934 | 0 | 0 | 1 | 0 | 0 | 0 | 0 | 0 | 0 | 0 | 0 | 0 | 0 | 0 | 0 | 0 |
| Malouf | David | Remembering Babylon | | AUSTRALIA | 0 | 1993 | 6 | 1934 | 0 | 0 | 1 | 0 | 0 | 0 | 0 | 0 | 0 | 0 | 0 | 0 | 0 | 0 | 0 | 0 |
| Malzieu | Mathias | The boy with the Cuckoo-Clock heart | | FRANCE | 0 | 2007 | 1 | 1974 | 1 | 0 | 0 | 0 | 0 | 0 | 0 | 0 | 0 | 0 | 0 | 0 | 0 | 0 | 0 | 0 |
| Mandela | Nelson | The long walk to freedom | | SOUTH AFRICA | 0 | 2013 | 5 | 1918 | 0 | 0 | 0 | 0 | 0 | 0 | 0 | 0 | 0 | 0 | 0 | 0 | 0 | 0 | 0 | 0 |
| Manicka | Rani | The rice mother | | MALAYSIA | 1 | 2002 | 4 | 1964 | 0 | 0 | 0 | 0 | 0 | 0 | 0 | 0 | 0 | 0 | 0 | 0 | 0 | 0 | 0 | 0 |
| Mann | Thomas | Death in Venice | | GERMANY | 0 | 1912 | 1 | 1875 | 1 | 1 | 0 | 0 | 0 | 0 | 0 | 0 | 0 | 0 | 0 | 0 | 0 | 0 | 0 | 0 |
| Mann | Thomas | The magic mountain | | GERMANY | 0 | 1924 | 1 | 1875 | 1 | 1 | 0 | 0 | 0 | 0 | 0 | 0 | 0 | 0 | 0 | 0 | 0 | 0 | 0 | 0 |

| **SURNAME** | **NAME** | | **TITLE** | **NATIONALITY** | **GENDER** | **PUB_YEAR** | **CONTINENT** | **DATE OF BIRTH** | **TRANSLATED** | **NOBEL** | **NEUSTADT** | **BOOKER** | **PULITZER** | **NEBULA** | **CAINE** | **FRANZ KAFKA** | **NBA** | **BBA** | **WOMEN'S PRIZE FOR FICTION** | **NEW YORK TIMES** | **THE GUARDIAN** | **THE NEW YORKER** | **BBC** | **ATLANTIC** |
| --- | --- | --- | --- | --- | --- | --- | --- | --- | --- | --- | --- | --- | --- | --- | --- | --- | --- | --- | --- | --- | --- | --- | --- | --- |
| Mann | Thomas | | Buddenbrooks | GERMANY | 0 | 1901 | 1 | 1875 | 1 | 1 | 0 | 0 | 0 | 0 | 0 | 0 | 0 | 0 | 0 | 0 | 0 | 0 | 0 | 0 |
| Mann | William J. | | All American Boy | USA | 0 | 2005 | 2 | 1963 | 0 | 0 | 0 | 0 | 0 | 0 | 0 | 0 | 0 | 0 | 0 | 0 | 0 | 0 | 0 | 0 |
| Mann | William J. | | Men Who Love Men | USA | 0 | 2007 | 2 | 1963 | 0 | 0 | 0 | 0 | 0 | 0 | 0 | 0 | 0 | 0 | 0 | 0 | 0 | 0 | 0 | 0 |
| Mann | William J. | | Object of Desire | USA | 0 | 2009 | 2 | 1963 | 0 | 0 | 0 | 0 | 0 | 0 | 0 | 0 | 0 | 0 | 0 | 0 | 0 | 0 | 0 | 0 |
| Mann | William J. | | Where the Boys Are | USA | 0 | 2003 | 2 | 1963 | 0 | 0 | 0 | 0 | 0 | 0 | 0 | 0 | 0 | 0 | 0 | 0 | 0 | 0 | 0 | 0 |
| Mansfield | Katherine | | New Zealand Stories | NEW ZEALAND | 1 | 1921 | 6 | 1888 | 0 | 0 | 0 | 0 | 0 | 0 | 0 | 0 | 0 | 0 | 0 | 0 | 0 | 0 | 0 | 0 |
| Manta | Lena | | The house by the river | TURKEY | 1 | 2017 | 4 | 1964 | 1 | 0 | 0 | 0 | 0 | 0 | 0 | 0 | 0 | 0 | 0 | 0 | 0 | 0 | 0 | 0 |
| Mantel | Hilary | | Bring Up the Bodies | UK | 1 | 2012 | 1 | 1952 | 0 | 0 | 0 | 1 | 0 | 0 | 0 | 0 | 0 | 1 | 0 | 1 | 0 | 0 | 0 | 1 |
| Mantel | Hilary | | Wolf Hall | UK | 1 | 2009 | 1 | 1952 | 0 | 0 | 0 | 1 | 0 | 0 | 0 | 0 | 0 | 1 | 0 | 0 | 0 | 0 | 0 | 0 |
| Manzoni | Alessandro | | The betrothed | ITALY | 0 | 1825 | 1 | 1785 | 1 | 0 | 0 | 0 | 0 | 0 | 0 | 0 | 0 | 0 | 0 | 0 | 0 | 0 | 0 | 0 |
| Márai | Sándor | | Embers | HUNGARY | 0 | 1942 | 1 | 1900 | 1 | 0 | 0 | 0 | 0 | 0 | 0 | 0 | 0 | 0 | 0 | 0 | 0 | 0 | 0 | 0 |
| Maraini | Dacia | | Train to Budapest | ITALY | 1 | 2008 | 1 | 1936 | 1 | 0 | 0 | 0 | 0 | 0 | 0 | 0 | 0 | 0 | 0 | 0 | 0 | 0 | 0 | 0 |
| Marías | Javier | | Tomorrow in the battle think on me | SPAIN | 0 | 1994 | 1 | 1951 | 1 | 0 | 0 | 0 | 0 | 0 | 0 | 0 | 0 | 0 | 0 | 0 | 0 | 0 | 0 | 0 |
| Marías | Javier | | The infatuations | SPAIN | 0 | 2011 | 1 | 1951 | 1 | 0 | 0 | 0 | 0 | 0 | 0 | 0 | 0 | 0 | 0 | 0 | 1 | 0 | 0 | 0 |
| Marías | Javier | | The man of feeling | SPAIN | 0 | 1986 | 1 | 1951 | 1 | 0 | 0 | 0 | 0 | 0 | 0 | 0 | 0 | 0 | 0 | 0 | 0 | 0 | 0 | 0 |
| Markandaya | Kamala | | Nectar in a sieve | INDIA | 1 | 1954 | 4 | 1924 | 0 | 0 | 0 | 0 | 0 | 0 | 0 | 0 | 0 | 0 | 0 | 0 | 0 | 0 | 0 | 0 |
| Markaris | Petros | | Che Committed Suicide | TURKEY | 0 | 2004 | 4 | 1937 | 1 | 0 | 0 | 0 | 0 | 0 | 0 | 0 | 0 | 0 | 0 | 0 | 0 | 0 | 0 | 0 |
| Markaris | Petros | | Deadline in Athens | TURKEY | 0 | 1995 | 4 | 1937 | 1 | 0 | 0 | 0 | 0 | 0 | 0 | 0 | 0 | 0 | 0 | 0 | 0 | 0 | 0 | 0 |
| Marone | Lorenzo | | The temptation to be happy | ITALY | 0 | 2015 | 1 | 1974 | 1 | 0 | 0 | 0 | 0 | 0 | 0 | 0 | 0 | 0 | 0 | 0 | 0 | 0 | 0 | 0 |
| Marquand | John | | The Late George Apley | USA | 0 | 1937 | 2 | 1893 | 0 | 0 | 0 | 0 | 1 | 0 | 0 | 0 | 0 | 0 | 0 | 0 | 0 | 0 | 0 | 0 |
| Márquez | Gabriel García | | One hundred years of solitude | COLOMBIA | 0 | 1967 | 3 | 1927 | 1 | 1 | 1 | 0 | 0 | 0 | 0 | 0 | 0 | 0 | 0 | 0 | 0 | 0 | 1 | 0 |
| Márquez | Gabriel García | | Of love and other demons | COLOMBIA | 0 | 1994 | 3 | 1927 | 1 | 1 | 1 | 0 | 0 | 0 | 0 | 0 | 0 | 0 | 0 | 0 | 0 | 0 | 0 | 0 |
| Márquez | Gabriel García | | Love in the time of cholera | COLOMBIA | 0 | 1985 | 3 | 1927 | 1 | 1 | 1 | 0 | 0 | 0 | 0 | 0 | 0 | 0 | 0 | 0 | 0 | 0 | 1 | 0 |
| Martel | Yann | | Life of Pi | CANADA | 0 | 2001 | 2 | 1963 | 0 | 0 | 0 | 1 | 0 | 0 | 0 | 0 | 0 | 0 | 0 | 0 | 0 | 0 | 0 | 0 |
| Martin | George | | A game of thrones | USA | 0 | 1996 | 2 | 1948 | 0 | 0 | 0 | 0 | 0 | 0 | 0 | 0 | 0 | 0 | 0 | 0 | 0 | 0 | 0 | 0 |
| Martin | George | | A clash of kings | USA | 0 | 1998 | 2 | 1948 | 0 | 0 | 0 | 0 | 0 | 0 | 0 | 0 | 0 | 0 | 0 | 0 | 0 | 0 | 0 | 0 |
| Martin | George | | A storm of swords | USA | 0 | 2000 | 2 | 1948 | 0 | 0 | 0 | 0 | 0 | 0 | 0 | 0 | 0 | 0 | 0 | 0 | 0 | 0 | 0 | 0 |
| Martin | George | | A feast for crows | USA | 0 | 2005 | 2 | 1948 | 0 | 0 | 0 | 0 | 0 | 0 | 0 | 0 | 0 | 0 | 0 | 0 | 0 | 0 | 0 | 0 |
| Martin | George | | A dance with dragons | USA | 0 | 2011 | 2 | 1948 | 0 | 0 | 0 | 0 | 0 | 0 | 0 | 0 | 0 | 0 | 0 | 0 | 0 | 0 | 0 | 0 |
| Martínez | Guillermo | | The Oxford murders | ARGENTINA | 0 | 2003 | 3 | 1962 | 1 | 0 | 0 | 0 | 0 | 0 | 0 | 0 | 0 | 0 | 0 | 0 | 0 | 0 | 0 | 0 |
| Martínez | Tomás Eloy | | Purgatory | ARGENTINA | 0 | 1956 | 3 | 1934 | 1 | 0 | 0 | 0 | 0 | 0 | 0 | 0 | 0 | 0 | 0 | 0 | 0 | 0 | 0 | 0 |
| Martínez | Tomás Eloy | | The Tango Singer | ARGENTINA | 0 | 2004 | 3 | 1934 | 1 | 0 | 0 | 0 | 0 | 0 | 0 | 0 | 0 | 0 | 0 | 0 | 0 | 0 | 0 | 0 |
| Matar | Hisham | | In the Country of Men | LIBYA | 0 | 2006 | 5 | 1970 | 0 | 0 | 0 | 0 | 0 | 0 | 0 | 0 | 0 | 0 | 0 | 0 | 0 | 0 | 0 | 0 |
| Matar | Hisham | | Anatomy of a Disappearance | LIBYA | 0 | 2011 | 5 | 1970 | 0 | 0 | 0 | 0 | 0 | 0 | 0 | 0 | 0 | 0 | 0 | 0 | 0 | 0 | 0 | 0 |
| Matar | Hisham | | The return | LIBYA | 0 | 2016 | 5 | 1970 | 0 | 0 | 0 | 0 | 0 | 0 | 0 | 0 | 0 | 0 | 0 | 0 | 0 | 0 | 0 | 0 |
| Matesis | Pavlos | | The Daughter | GREECE | 0 | 1990 | 1 | 1933 | 1 | 0 | 0 | 0 | 0 | 0 | 0 | 0 | 0 | 0 | 0 | 0 | 0 | 0 | 0 | 0 |
| Matlwa | Kopano | | Evening primrose | SOUTH AFRICA | 1 | 2016 | 5 | 1985 | 0 | 0 | 0 | 0 | 0 | 0 | 0 | 0 | 0 | 0 | 0 | 0 | 0 | 0 | 0 | 0 |
| Matsumoto | Seichō | | A quiet place | JAPAN | 0 | 1971 | 4 | 1909 | 1 | 0 | 0 | 0 | 0 | 0 | 0 | 0 | 0 | 0 | 0 | 0 | 0 | 0 | 0 | 0 |
| Maturin | Charles | | Melmoth the Wandered | IRELAND | 0 | 1820 | 1 | 1780 | 0 | 0 | 0 | 0 | 0 | 0 | 0 | 0 | 0 | 0 | 0 | 0 | 0 | 0 | 0 | 0 |
| Maupin | Armistead | | Mary Ann in Autumn | USA | 0 | 2010 | 2 | 1944 | 0 | 0 | 0 | 0 | 0 | 0 | 0 | 0 | 0 | 0 | 0 | 0 | 1 | 0 | 0 | 0 |
| Mauriac | François | | Flesh and Blood | FRANCE | 0 | 1920 | 1 | 1885 | 1 | 1 | 0 | 0 | 0 | 0 | 0 | 0 | 0 | 0 | 0 | 0 | 0 | 0 | 0 | 0 |
| Mawer | Simon | | The Glass Room | UK | 0 | 2009 | 1 | 1948 | 0 | 0 | 0 | 0 | 0 | 0 | 0 | 0 | 0 | 0 | 0 | 0 | 0 | 0 | 0 | 0 |
| Mbue | Imbolo | | Behold the dreamers | CAMEROON | 1 | 2016 | 5 | 1982 | 0 | 0 | 0 | 0 | 0 | 0 | 0 | 0 | 0 | 0 | 0 | 0 | 0 | 0 | 0 | 0 |
| McBride | James | | Deacon King Kong | USA | 0 | 2020 | 2 | 1957 | 0 | 0 | 0 | 0 | 0 | 0 | 0 | 0 | 0 | 0 | 0 | 1 | 0 | 0 | 0 | 0 |
| McCabe | Patrick | | The Butcher Boy | IRELAND | 0 | 1992 | 1 | 1955 | 0 | 0 | 0 | 0 | 0 | 0 | 0 | 0 | 0 | 0 | 0 | 0 | 0 | 0 | 0 | 0 |
| McCann | Colum | | Apeirogon | IRELAND | 0 | 2020 | 1 | 1965 | 0 | 0 | 0 | 0 | 0 | 0 | 0 | 0 | 0 | 0 | 0 | 0 | 0 | 0 | 1 | 0 |
| McCarthy | Cormac | | The crossing | USA | 0 | 1994 | 2 | 1933 | 0 | 0 | 0 | 0 | 0 | 0 | 0 | 0 | 0 | 0 | 0 | 0 | 0 | 0 | 0 | 0 |
| McCarthy | Cormac | | All the pretty horses | USA | 0 | 1992 | 2 | 1933 | 0 | 0 | 0 | 0 | 0 | 0 | 0 | 0 | 1 | 0 | 0 | 0 | 0 | 0 | 0 | 0 |
| McCarthy | Cormac | | Cities of the plain | USA | 0 | 1998 | 2 | 1933 | 0 | 0 | 0 | 0 | 0 | 0 | 0 | 0 | 0 | 0 | 0 | 0 | 0 | 0 | 0 | 0 |
| McCarthy | Cormac | | Child of God | USA | 0 | 1974 | 2 | 1933 | 0 | 0 | 0 | 0 | 0 | 0 | 0 | 0 | 0 | 0 | 0 | 0 | 0 | 0 | 0 | 0 |
| McCarthy | Cormac | | The Road | USA | 0 | 2006 | 2 | 1933 | 0 | 0 | 0 | 0 | 1 | 0 | 0 | 0 | 0 | 0 | 0 | 0 | 0 | 0 | 0 | 0 |
| McCarthy | Tom | | C | UK | 0 | 2010 | 1 | 1969 | 0 | 0 | 0 | 0 | 0 | 0 | 0 | 0 | 0 | 0 | 0 | 0 | 0 | 0 | 0 | 0 |
| McCourt | Frank | | Angela's Ashes | IRELAND | 0 | 1996 | 1 | 1930 | 0 | 0 | 0 | 0 | 0 | 0 | 0 | 0 | 0 | 0 | 0 | 0 | 0 | 0 | 0 | 0 |
| McCullers | Carson | | Heart is a lonely hunter | USA | 1 | 1940 | 2 | 1917 | 0 | 0 | 0 | 0 | 0 | 0 | 0 | 0 | 0 | 0 | 0 | 0 | 0 | 0 | 0 | 0 |
| McCullers | Carson | | Reflections in a Golden Eye | USA | 1 | 1941 | 2 | 1917 | 0 | 0 | 0 | 0 | 0 | 0 | 0 | 0 | 0 | 0 | 0 | 0 | 0 | 0 | 0 | 0 |
| McCullers | Carson | | The Member of the Wedding | USA | 1 | 1946 | 2 | 1917 | 0 | 0 | 0 | 0 | 0 | 0 | 0 | 0 | 0 | 0 | 0 | 0 | 0 | 0 | 0 | 0 |
| McCullers | Carson | | Clock Without Hands | USA | 1 | 1961 | 2 | 1917 | 0 | 0 | 0 | 0 | 0 | 0 | 0 | 0 | 0 | 0 | 0 | 0 | 0 | 0 | 0 | 0 |
| McCullough | Colleen | | The thorn birds | AUSTRALIA | 1 | 1977 | 6 | 1937 | 0 | 0 | 0 | 0 | 0 | 0 | 0 | 0 | 0 | 0 | 0 | 0 | 0 | 0 | 1 | 0 |
| McCullough | Colleen | | Caesar | AUSTRALIA | 1 | 1997 | 6 | 1937 | 0 | 0 | 0 | 0 | 0 | 0 | 0 | 0 | 0 | 0 | 0 | 0 | 0 | 0 | 0 | 0 |
| McEwan | Ian | | Amsterdam | UK | 0 | 1998 | 1 | 1948 | 0 | 0 | 0 | 1 | 0 | 0 | 0 | 0 | 0 | 1 | 0 | 0 | 0 | 0 | 0 | 0 |
| McEwan | Ian | | Atonement | UK | 0 | 2001 | 1 | 1948 | 0 | 0 | 0 | 0 | 0 | 0 | 0 | 0 | 0 | 1 | 0 | 0 | 0 | 0 | 0 | 0 |
| McEwan | Ian | | On Chesil beach | UK | 0 | 2007 | 1 | 1948 | 0 | 0 | 0 | 0 | 0 | 0 | 0 | 0 | 0 | 1 | 0 | 0 | 0 | 0 | 0 | 0 |
| McEwan | Ian | | Black dogs | UK | 0 | 1992 | 1 | 1948 | 0 | 0 | 0 | 0 | 0 | 0 | 0 | 0 | 0 | 1 | 0 | 0 | 0 | 0 | 0 | 0 |
| McEwan | Ian | | The cement garden | UK | 0 | 1978 | 1 | 1948 | 0 | 0 | 0 | 0 | 0 | 0 | 0 | 0 | 0 | 1 | 0 | 0 | 0 | 0 | 0 | 0 |
| McGahern | John | | Amongst Women | IRELAND | 0 | 1990 | 1 | 1934 | 0 | 0 | 0 | 0 | 0 | 0 | 0 | 0 | 0 | 0 | 0 | 0 | 0 | 0 | 0 | 0 |
| McGuire | Ian | | The North Water | UK | 0 | 2016 | 1 | 1964 | 0 | 0 | 0 | 0 | 0 | 0 | 0 | 0 | 0 | 0 | 0 | 1 | 0 | 0 | 0 | 0 |
| McInerney | Jay | | Bright lights big city | USA | 0 | 1984 | 2 | 1955 | 0 | 0 | 0 | 0 | 0 | 0 | 0 | 0 | 0 | 0 | 0 | 0 | 0 | 0 | 0 | 0 |
| McLeod | Cynthia | | The Cost of Sugar | SURINAME | 1 | 1987 | 3 | 1936 | 1 | 0 | 0 | 0 | 0 | 0 | 0 | 0 | 0 | 0 | 0 | 0 | 0 | 0 | 0 | 0 |
| McMurtry | Larry | | Lonesome dove | USA | 0 | 1985 | 2 | 1936 | 0 | 0 | 0 | 0 | 1 | 0 | 0 | 0 | 0 | 0 | 0 | 0 | 0 | 0 | 0 | 0 |
| McPherson | James | | Elbow Room | USA | 0 | 1977 | 2 | 1943 | 0 | 0 | 0 | 0 | 1 | 0 | 0 | 0 | 0 | 0 | 0 | 0 | 0 | 0 | 0 | 0 |
| Mda | Zakes | | The heart of redness | SOUTH AFRICA | 0 | 2000 | 5 | 1948 | 0 | 0 | 0 | 0 | 0 | 0 | 0 | 0 | 0 | 0 | 0 | 0 | 0 | 0 | 0 | 0 |
| Meek | James | | The Heart Broke In | UK | 0 | 2012 | 1 | 1962 | 0 | 0 | 0 | 0 | 0 | 0 | 0 | 0 | 0 | 0 | 0 | 0 | 0 | 0 | 0 | 0 |
| Mehran | Marsha | The Margaret Thatcher School of Beaut | | IRAN | 1 | 2013 | 4 | 1977 | 1 | 0 | 0 | 0 | 0 | 0 | 0 | 0 | 0 | 0 | 0 | 0 | 0 | 0 | 0 | 0 |
| Mehta | Gita | | A River Sutra | INDIA | 1 | 1993 | 4 | 1943 | 0 | 0 | 0 | 0 | 0 | 0 | 0 | 0 | 0 | 0 | 0 | 0 | 0 | 0 | 0 | 0 |
| Melchor | Fernanda | | Hurricane Season | MEXICO | 1 | 2017 | 3 | 1982 | 1 | 0 | 0 | 0 | 0 | 0 | 0 | 0 | 0 | 0 | 0 | 0 | 1 | 0 | 0 | 0 |
| Melville | Herman | | Billy Budd | USA | 0 | 1924 | 2 | 1819 | 0 | 0 | 0 | 0 | 0 | 0 | 0 | 0 | 0 | 0 | 0 | 0 | 0 | 0 | 0 | 0 |
| Melville | Herman | | Moby Dick | USA | 0 | 1851 | 2 | 1819 | 0 | 0 | 0 | 0 | 0 | 0 | 0 | 0 | 0 | 0 | 0 | 0 | 0 | 0 | 0 | 1 |
| Mengestu | Dinaw | | All our names | ETHIOPIA | 0 | 2014 | 5 | 1978 | 0 | 0 | 0 | 0 | 0 | 0 | 0 | 0 | 0 | 0 | 0 | 0 | 0 | 0 | 0 | 0 |
| Mengiste | Maaza | | Beneath the lion's gaze | ETHIOPIA | 1 | 2010 | 5 | 1974 | 0 | 0 | 0 | 0 | 0 | 0 | 0 | 0 | 0 | 0 | 0 | 0 | 0 | 0 | 0 | 0 |
| Mercier | Pascal | | Night train to Lisbon | SWITZERLAND | 0 | 2004 | 1 | 1944 | 1 | 0 | 0 | 0 | 0 | 0 | 0 | 0 | 0 | 0 | 0 | 0 | 0 | 0 | 0 | 0 |
| Mernissi | Fatima | | Dreams of trespass | MOROCCO | 1 | 1994 | 5 | 1940 | 0 | 0 | 0 | 0 | 0 | 0 | 0 | 0 | 0 | 0 | 0 | 0 | 0 | 0 | 0 | 0 |
| Meruane | Lina | | Seeing Red | CHILE | 1 | 2012 | 3 | 1970 | 1 | 0 | 0 | 0 | 0 | 0 | 0 | 0 | 0 | 0 | 0 | 0 | 0 | 0 | 0 | 0 |
| Metalious | Grace | | Peyton Place | USA | 1 | 1956 | 2 | 1924 | 0 | 0 | 0 | 0 | 0 | 0 | 0 | 0 | 0 | 0 | 0 | 0 | 0 | 0 | 0 | 0 |
| Meyer | Deon | | Heart of the hunter | SOUTH AFRICA | 0 | 2000 | 5 | 1958 | 1 | 0 | 0 | 0 | 0 | 0 | 0 | 0 | 0 | 0 | 0 | 0 | 0 | 0 | 0 | 0 |
| Meyer | Deon | | Devil's peak | SOUTH AFRICA | 0 | 2004 | 5 | 1958 | 1 | 0 | 0 | 0 | 0 | 0 | 0 | 0 | 0 | 0 | 0 | 0 | 0 | 0 | 0 | 0 |
| Meyer | Stephenie | | Eclipse | USA | 1 | 2007 | 2 | 1973 | 0 | 0 | 0 | 0 | 0 | 0 | 0 | 0 | 0 | 0 | 0 | 0 | 0 | 0 | 0 | 0 |
| Meyer | Stephenie | | Breaking dawn | USA | 1 | 2008 | 2 | 1973 | 0 | 0 | 0 | 0 | 0 | 0 | 0 | 0 | 0 | 1 | 0 | 0 | 0 | 0 | 0 | 0 |

| **SURNAME** | | **NAME** | **TITLE** | **NATIONALITY** | **GENDER** | **PUB_YEAR** | **CONTINENT** | **DATE OF BIRTH** | **TRANSLATED** | **NOBEL** | **NEUSTADT** | **BOOKER** | **PULITZER** | **NEBULA** | **CAINE** | **FRANZ KAFKA** | **NBA** | **BBA** | **WOMEN'S PRIZE FOR FICTION** | **NEW YORK TIMES** | **THE GUARDIAN** | **THE NEW YORKER** | **BBC** | **ATLANTIC** |
| --- | --- | --- | --- | --- | --- | --- | --- | --- | --- | --- | --- | --- | --- | --- | --- | --- | --- | --- | --- | --- | --- | --- | --- | --- |
| Meyer  Meyer | Stephenie New moon  Stephenie Twilight | | | USA  USA | 1  1 | 2006  2005 | 2  2 | 1973  1973 | 0  0 | 0  0 | 0  0 | 0  0 | 0  0 | 0  0 | 0  0 | 0  0 | 0  0 | 0  0 | 0  0 | 0  0 | 0  0 | 0  0 | 0  0 | 0  0 |
| Meyer | Philipp The son | | | USA | 0 | 2013 | 2 | 1974 | 0 | 0 | 0 | 0 | 0 | 0 | 0 | 0 | 0 | 0 | 0 | 0 | 1 | 0 | 1 | 1 |
| Michon | Pierre Small lives | | | FRANCE | 0 | 1984 | 1 | 1945 | 1 | 0 | 0 | 0 | 0 | 0 | 0 | 1 | 0 | 0 | 0 | 0 | 0 | 0 | 0 | 0 |
| Middleton | Stanley Holiday | | | UK | 0 | 1974 | 1 | 1919 | 0 | 0 | 0 | 1 | 0 | 0 | 0 | 0 | 0 | 0 | 0 | 0 | 0 | 0 | 0 | 0 |
| Millar  Millar | Margaret Beast in view  Margaret Ask for me tomorrow | | | CANADA  CANADA | 1  1 | 1955  1976 | 2  2 | 1915  1915 | 0  0 | 0  0 | 0  0 | 0  0 | 0  0 | 0  0 | 0  0 | 0  0 | 0  0 | 0  0 | 0  0 | 0  0 | 0  0 | 0  0 | 0  0 | 0  0 |
| Miller  Miller | Henry Nexus  Henry Tropic of Cancer | | | USA  USA | 0  0 | 1959  1934 | 2  2 | 1891  1891 | 0  0 | 0  0 | 0  0 | 0  0 | 0  0 | 0  0 | 0  0 | 0  0 | 0  0 | 0  0 | 0  0 | 0  0 | 0  0 | 0  0 | 0  0 | 0  0 |
| Miller  Miller | Madeline The song of Achilles  Madeline Circe | | | USA  USA | 1  1 | 2011  2018 | 2  2 | 1978  1978 | 0  0 | 0  0 | 0  0 | 0  0 | 0  0 | 0  0 | 0  0 | 0  0 | 0  0 | 0  0 | 1  0 | 0  0 | 0  0 | 0  0 | 0  0 | 0  1 |
| Miller | Caroline Pafford Lamb in His Bosom | | | USA | 1 | 1933 | 2 | 1903 | 0 | 0 | 0 | 0 | 1 | 0 | 0 | 0 | 0 | 0 | 0 | 0 | 0 | 0 | 0 | 0 |
| Miller | Sue While I Was Gone | | | USA | 1 | 1999 | 2 | 1943 | 0 | 0 | 0 | 0 | 0 | 0 | 0 | 0 | 0 | 0 | 0 | 0 | 0 | 0 | 0 | 0 |
| Miller  Miller | Kei The Last Warner Woman  Kei Augustown | | | JAMAICA  JAMAICA | 0  0 | 2010  2016 | 3  3 | 1978  1978 | 0  0 | 0  0 | 0  0 | 0  0 | 0  0 | 0  0 | 0  0 | 0  0 | 0  0 | 0  0 | 0  0 | 0  0 | 0  0 | 0  0 | 0  0 | 0  0 |
| Miller | AD Snowdrops | | | UK | 0 | 2011 | 1 | 1974 | 0 | 0 | 0 | 0 | 0 | 0 | 0 | 0 | 0 | 0 | 0 | 0 | 0 | 0 | 0 | 0 |
| Millhauser Steven n Dressler: The Tale of an American Dre USA 0 1996 2 1943 0 0 0 0 1 0 0 0 0 0 0 0 0 0 0 0 | | | | | | | | | | | | | | | | | | | | | | | | |
| Mills | Magnus The Restraint of Beasts | | | UK | 0 | 1998 | 1 | 1954 | 0 | 0 | 0 | 0 | 0 | 0 | 0 | 0 | 0 | 0 | 0 | 0 | 0 | 0 | 0 | 0 |
| Milne | Alan Alexander Once on a Time | | | UK | 0 | 1917 | 1 | 1882 | 0 | 0 | 0 | 0 | 0 | 0 | 0 | 0 | 0 | 0 | 0 | 0 | 0 | 0 | 0 | 0 |
| Minato | Kanae Confession | | | JAPAN | 1 | 2008 | 4 | 1973 | 1 | 0 | 0 | 0 | 0 | 0 | 0 | 0 | 0 | 0 | 0 | 0 | 0 | 0 | 0 | 0 |
| Mishima Mishima Mishima Mishima Mishima Mishima  Mishima | Yukio Confessions of a mask  Yukio Forbidden colors  Yukio The Decay of the Angel  Yukio The Frolic of the Beasts  Yukio e Sailor Who Fell from Grace with the S Yukio The Sound of Waves  Yukio Thirst for Love | | | JAPAN JAPAN JAPAN JAPAN JAPAN JAPAN  JAPAN | 0  0  0  0  0  0  0 | 1949  1951  1971  1961  1963  1954  1950 | 4  4  4  4  4  4  4 | 1925  1925  1925  1925  1925  1925  1925 | 1  1  1  1  1  1  1 | 0  0  0  0  0  0  0 | 0  0  0  0  0  0  0 | 0  0  0  0  0  0  0 | 0  0  0  0  0  0  0 | 0  0  0  0  0  0  0 | 0  0  0  0  0  0  0 | 0  0  0  0  0  0  0 | 0  0  0  0  0  0  0 | 0  0  0  0  0  0  0 | 0  0  0  0  0  0  0 | 0  0  0  0  0  0  0 | 0  0  0  0  0  0  0 | 0  0  0  0  0  0  0 | 0  0  0  0  0  0  0 | 0  0  0  0  0  0  0 |
| Mistry  Mistry Mistry | Rohinton A Fine Balance  Rohinton Family Matters  Rohinton Such a Long Journey | | | INDIA  INDIA INDIA | 0  0  0 | 1995  2002  1991 | 4  4  4 | 1952  1952  1952 | 0  0  0 | 0  0  0 | 1  1  1 | 0  0  0 | 0  0  0 | 0  0  0 | 0  0  0 | 0  0  0 | 0  0  0 | 0  0  0 | 0  0  0 | 0  0  0 | 0  0  0 | 0  0  0 | 0  0  0 | 0  0  0 |
| Mitchard | Jacquelyn The Deep End of the Ocean | | | USA | 1 | 1996 | 2 | 1956 | 0 | 0 | 0 | 0 | 0 | 0 | 0 | 0 | 0 | 0 | 0 | 0 | 0 | 0 | 0 | 0 |
| Mitchell  Mitchell | David The thousand autumns of Jacob de Zoe  David Cloud atlas | | | UK  UK | 0  0 | 2010  2004 | 1  1 | 1969  1969 | 0  0 | 0  0 | 0  0 | 0  0 | 0  0 | 0  0 | 0  0 | 0  0 | 0  0 | 0  1 | 0  0 | 0  0 | 0  0 | 0  0 | 0  0 | 0  1 |
| Mitchell | Margaret Gone with the wind | | | USA | 1 | 1936 | 2 | 1900 | 0 | 0 | 0 | 0 | 1 | 0 | 0 | 0 | 1 | 0 | 0 | 0 | 0 | 0 | 1 | 0 |
| Mitford | Nancy Love in a cold climate | | | UK | 1 | 1949 | 1 | 1904 | 0 | 0 | 0 | 0 | 0 | 0 | 0 | 0 | 0 | 0 | 0 | 0 | 0 | 0 | 0 | 0 |
| Miyabe Miyabe  Miyabe | Miyuki All she was worth  Miyuki Brave Story  Miyuki The Gate of Sorrows | | | JAPAN JAPAN  JAPAN | 1  1  1 | 1992  2003  2016 | 4  4  4 | 1960  1960  1960 | 1  1  1 | 0  0  0 | 0  0  0 | 0  0  0 | 0  0  0 | 0  0  0 | 0  0  0 | 0  0  0 | 0  0  0 | 0  0  0 | 0  0  0 | 0  0  0 | 0  0  0 | 0  0  0 | 0  0  0 | 0  0  0 |
| Modiano  Modiano | Patrick Dora Bruder  Patrick In the café of lost youth | | | FRANCE  FRANCE | 0  0 | 1997  2007 | 1  1 | 1945  1945 | 1  1 | 1  1 | 0  0 | 0  0 | 0  0 | 0  0 | 0  0 | 0  0 | 0  0 | 0  0 | 0  0 | 0  0 | 0  0 | 0  0 | 0  0 | 0  0 |
| Momaday | Navarre Scott House Made of Dawn | | | USA | 0 | 1968 | 2 | 1934 | 0 | 0 | 0 | 0 | 1 | 0 | 0 | 0 | 0 | 0 | 0 | 0 | 0 | 0 | 0 | 0 |
| Monge | Emiliano The arid sky | | | MEXICO | 0 | 2012 | 3 | 1978 | 1 | 0 | 0 | 0 | 0 | 0 | 0 | 0 | 0 | 0 | 0 | 0 | 0 | 0 | 0 | 0 |
| Montalbán  Montalbán | Manuel Tattoo  Manuel Murder in the central committee | | | SPAIN  SPAIN | 0  0 | 1974  1981 | 1  1 | 1939  1939 | 1  1 | 0  0 | 0  0 | 0  0 | 0  0 | 0  0 | 0  0 | 0  0 | 0  0 | 0  0 | 0  0 | 0  0 | 0  0 | 0  0 | 0  0 | 0  0 |
| Montgomery | LM Anne of Green Gables | | | CANADA | 1 | 1908 | 2 | 1874 | 0 | 0 | 0 | 0 | 0 | 0 | 0 | 0 | 0 | 0 | 0 | 0 | 0 | 0 | 1 | 0 |
| Moore | Brian Lies of Silence | | | IRELAND | 0 | 1990 | 1 | 1921 | 0 | 0 | 0 | 0 | 0 | 0 | 0 | 0 | 0 | 0 | 0 | 0 | 0 | 0 | 0 | 0 |
| Moore | Alison The Lighthouse | | | UK | 1 | 2012 | 1 | 1971 | 0 | 0 | 0 | 0 | 0 | 0 | 0 | 0 | 0 | 0 | 0 | 0 | 0 | 0 | 0 | 0 |
| Morante | Elsa Arturo's island | | | ITALY | 1 | 1957 | 1 | 1912 | 1 | 0 | 0 | 0 | 0 | 0 | 0 | 0 | 0 | 0 | 0 | 0 | 0 | 0 | 0 | 0 |
| Moravia | Alberto Boredom | | | ITALY | 0 | 1960 | 1 | 1907 | 1 | 0 | 0 | 0 | 0 | 0 | 0 | 0 | 0 | 0 | 0 | 0 | 0 | 0 | 0 | 0 |
| More  More | Hannah Coelebs in Search of a Wife  Hannah The Shepherd of Salisbury Plain | | | UK  UK | 1  1 | 1809  1796 | 1  1 | 1745  1745 | 0  0 | 0  0 | 0  0 | 0  0 | 0  0 | 0  0 | 0  0 | 0  0 | 0  0 | 0  0 | 0  0 | 0  0 | 0  0 | 0  0 | 0  0 | 0  0 |
| Morgan | Sally My place | | | AUSTRALIA | 1 | 1987 | 6 | 1951 | 0 | 0 | 0 | 0 | 0 | 0 | 0 | 0 | 0 | 0 | 0 | 0 | 0 | 0 | 0 | 0 |
| Morgan | Robert Gap Creek | | | USA | 0 | 1999 | 2 | 1944 | 0 | 0 | 0 | 0 | 0 | 0 | 0 | 0 | 0 | 0 | 0 | 0 | 0 | 0 | 0 | 0 |
| Moriarty  Moriarty | Liane Big little lies  Liane Three wishes | | | AUSTRALIA  AUSTRALIA | 1  1 | 2014  2003 | 6  6 | 1966  1966 | 0  0 | 0  0 | 0  0 | 0  0 | 0  0 | 0  0 | 0  0 | 0  0 | 0  0 | 0  0 | 0  0 | 0  0 | 0  0 | 0  0 | 0  0 | 0  0 |
| Morrall | Clare Astonishing Splashes of Colour | | | UK | 1 | 2003 | 1 | 1952 | 0 | 0 | 0 | 0 | 0 | 0 | 0 | 0 | 0 | 0 | 0 | 0 | 0 | 0 | 0 | 0 |
| Morris | Heather The Tattooist of Auschwitz | | | NEW ZEALAND | 1 | 2018 | 6 | ? | 0 | 0 | 0 | 0 | 0 | 0 | 0 | 0 | 0 | 0 | 0 | 0 | 0 | 0 | 0 | 0 |
| Morris | Mary McGarry Songs in Ordinary Time | | | USA | 1 | 1995 | 2 | 1943 | 0 | 0 | 0 | 0 | 0 | 0 | 0 | 0 | 0 | 0 | 0 | 0 | 0 | 0 | 0 | 0 |
| Morrison Morrison Morrison Morrison Morrison Morrison  Morrison | Toni Beloved  Toni Jazz  Toni Paradise  Toni Song of Solomon Toni Sula  Toni The Bluest Eye  Toni Tar Baby | | | USA USA USA USA USA USA  USA | 1  1  1  1  1  1  1 | 1987  1992  1997  1977  1973  1970  1981 | 2  2  2  2  2  2  2 | 1931  1931  1931  1931  1931  1931  1931 | 0  0  0  0  0  0  0 | 1  1  1  1  1  1  1 | 0  0  0  0  0  0  0 | 0  0  0  0  0  0  0 | 1  0  0  0  0  0  0 | 0  0  0  0  0  0  0 | 0  0  0  0  0  0  0 | 0  0  0  0  0  0  0 | 0  0  0  0  0  0  0 | 0  0  0  0  0  0  0 | 0  0  0  0  0  0  0 | 0  0  0  0  0  0  0 | 0  0  0  0  0  0  0 | 0  0  0  0  0  0  0 | 0  0  0  0  0  0  0 | 0  0  0  0  0  0  0 |
| Morton  Morton | Kate The forgotten garden  Kate The lake house | | | AUSTRALIA  AUSTRALIA | 1  1 | 2008  2015 | 6  6 | 1976  1976 | 0  0 | 0  0 | 0  0 | 0  0 | 0  0 | 0  0 | 0  0 | 0  0 | 0  0 | 0  0 | 0  0 | 0  0 | 0  0 | 0  0 | 0  0 | 0  0 |
| Moshfegh | Ottessa My year of rest and relaxation | | | USA | 1 | 2018 | 2 | 1981 | 0 | 0 | 0 | 0 | 0 | 0 | 0 | 0 | 0 | 0 | 0 | 0 | 0 | 0 | 0 | 0 |
| Moyes | Jojo Me before you | | | UK | 1 | 2012 | 1 | 1969 | 0 | 0 | 0 | 0 | 0 | 0 | 0 | 0 | 0 | 0 | 0 | 0 | 0 | 0 | 0 | 0 |
| Mozley | Fiona Elmet | | | UK | 1 | 2017 | 1 | 1988 | 0 | 0 | 0 | 0 | 0 | 0 | 0 | 0 | 0 | 0 | 0 | 0 | 0 | 0 | 0 | 0 |
| Mphahlele | Es'kia Down Second Avenue | | | SOUTH AFRICA | 0 | 1959 | 5 | 1919 | 0 | 0 | 0 | 0 | 0 | 0 | 0 | 0 | 0 | 0 | 0 | 0 | 0 | 0 | 0 | 0 |
| Mukherjee | Neel The lives of others | | | INDIA | 0 | 2014 | 4 | 1970 | 0 | 0 | 0 | 0 | 0 | 0 | 0 | 0 | 0 | 0 | 0 | 0 | 0 | 0 | 0 | 0 |
| Mukherjee Mukherjee Mukherjee  Mukherjee | Bharati Jasmine  Bharati Leave It to Me  Bharati Miss New India  Bharati The Holder of the World | | | INDIA INDIA INDIA  INDIA | 1  1  1  1 | 1989  1997  2011  1993 | 4  4  4  4 | 1940  1940  1940  1940 | 0  0  0  0 | 0  0  0  0 | 0  0  0  0 | 0  0  0  0 | 0  0  0  0 | 0  0  0  0 | 0  0  0  0 | 0  0  0  0 | 0  0  0  0 | 0  0  0  0 | 0  0  0  0 | 0  0  0  0 | 0  0  0  0 | 0  0  0  0 | 0  0  0  0 | 0  0  0  0 |
| Mulisch | Harry The Discovery of Heaven | | | NETHERLANDS | 0 | 1992 | 1 | 1927 | 1 | 0 | 0 | 0 | 0 | 0 | 0 | 0 | 0 | 0 | 0 | 0 | 0 | 0 | 0 | 0 |
| Muller Muller  Muller | Carl Once Upon a Tender Time Carl Spit and Polish  Carl The Jam Fruit Tree | | | SRI LANKA SRI LANKA  SRI LANKA | 0  0  0 | 1995  1998  1993 | 4  4  4 | 1935  1935  1935 | 0  0  0 | 0  0  0 | 0  0  0 | 0  0  0 | 0  0  0 | 0  0  0 | 0  0  0 | 0  0  0 | 0  0  0 | 0  0  0 | 0  0  0 | 0  0  0 | 0  0  0 | 0  0  0 | 0  0  0 | 0  0  0 |
| Müller | Herta The hunger angel | | | GERMANY | 1 | 2009 | 1 | 1953 | 1 | 1 | 0 | 0 | 0 | 0 | 0 | 0 | 0 | 0 | 0 | 0 | 0 | 0 | 0 | 0 |
| Munro  Munro | Alice Who do you think you are  Alice eship friendship courtship loveship marr | | | CANADA  CANADA | 1  1 | 1978  2001 | 2  2 | 1931  1931 | 0  0 | 1  1 | 0  0 | 1  1 | 0  0 | 0  0 | 0  0 | 0  0 | 0  0 | 0  0 | 0  0 | 0  0 | 0  0 | 0  0 | 0  0 | 0  0 |
| Murakami Murakami Murakami  Murakami | Haruki 1q84  Haruki Men without women  Haruki Killing commendatore  Haruki The elephant vanishes | | | JAPAN JAPAN JAPAN  JAPAN | 0  0  0  0 | 2010  2014  2017  1993 | 4  4  4  4 | 1949  1949  1949  1949 | 1  1  1  1 | 0  0  0  0 | 0  0  0  0 | 0  0  0  0 | 0  0  0  0 | 0  0  0  0 | 0  0  0  0 | 1  1  1  1 | 0  0  0  0 | 0  0  0  0 | 0  0  0  0 | 0  0  0  0 | 0  0  0  0 | 0  0  0  0 | 0  0  0  0 | 0  0  0  0 |

| **SURNAME** | **NAME** | **TITLE** | **NATIONALITY** | | **GENDER** | **PUB_YEAR** | **CONTINENT** | **DATE OF BIRTH** | **TRANSLATED** | **NOBEL** | **NEUSTADT** | **BOOKER** | **PULITZER** | **NEBULA** | **CAINE** | **FRANZ KAFKA** | **NBA** | **BBA** | **WOMEN'S PRIZE FOR FICTION** | **NEW YORK TIMES** | **THE GUARDIAN** | **THE NEW YORKER** | **BBC** | **ATLANTIC** |
| --- | --- | --- | --- | --- | --- | --- | --- | --- | --- | --- | --- | --- | --- | --- | --- | --- | --- | --- | --- | --- | --- | --- | --- | --- |
| Murakami | Haruki | Norwegian Wood | | JAPAN | 0 | 1987 | 4 | 1949 | 1 | 0 | 0 | 0 | 0 | 0 | 0 | 1 | 0 | 0 | 0 | 0 | 0 | 0 | 0 | 0 |
| Murakami | Ryū | Almost transparent blue | | JAPAN | 0 | 1976 | 4 | 1952 | 1 | 0 | 0 | 0 | 0 | 0 | 0 | 0 | 0 | 0 | 0 | 0 | 0 | 0 | 0 | 0 |
| Murata | Sayaka | Convenience store woman | | JAPAN | 1 | 2016 | 4 | 1979 | 1 | 0 | 0 | 0 | 0 | 0 | 0 | 0 | 0 | 0 | 0 | 0 | 0 | 1 | 0 | 0 |
| Murdoch | Iris | The Sea, The Sea | | IRELAND | 1 | 1978 | 1 | 1919 | 0 | 0 | 0 | 1 | 0 | 0 | 0 | 0 | 0 | 0 | 0 | 0 | 0 | 0 | 0 | 0 |
| Murdoch | Iris | Under the net | | IRELAND | 1 | 1954 | 1 | 1919 | 0 | 0 | 0 | 0 | 0 | 0 | 0 | 0 | 0 | 0 | 0 | 0 | 0 | 0 | 0 | 0 |
| Murgia | Michela | Accabadora | | ITALY | 1 | 2009 | 1 | 1972 | 1 | 0 | 0 | 0 | 0 | 0 | 0 | 0 | 0 | 0 | 0 | 0 | 0 | 0 | 0 | 0 |
| Murray | Paul | Skippy Dies | | IRELAND | 0 | 2010 | 1 | 1975 | 0 | 0 | 0 | 0 | 0 | 0 | 0 | 0 | 0 | 0 | 0 | 0 | 1 | 0 | 0 | 1 |
| Musil | Robert | The man without qualities | | AUSTRIA | 0 | 1943 | 1 | 1880 | 1 | 0 | 0 | 0 | 0 | 0 | 0 | 0 | 0 | 0 | 0 | 0 | 0 | 0 | 0 | 0 |
| Musil | Robert | Confusions of young Torless | | AUSTRIA | 0 | 1906 | 1 | 1880 | 1 | 0 | 0 | 0 | 0 | 0 | 0 | 0 | 0 | 0 | 0 | 0 | 0 | 0 | 0 | 0 |
| Mutis | Álvaro | Adventures and Misadventures of Maq | | COLOMBIA | 0 | 1993 | 3 | 1923 | 1 | 0 | 1 | 0 | 0 | 0 | 0 | 0 | 0 | 0 | 0 | 0 | 0 | 0 | 0 | 0 |
| Nabokov | Vladimir | The enchanter | | RUSSIA | 0 | 1939 | 1 | 1899 | 1 | 0 | 0 | 0 | 0 | 0 | 0 | 0 | 0 | 0 | 0 | 0 | 0 | 0 | 0 | 0 |
| Nabokov | Vladimir | Lolita | | RUSSIA | 0 | 1955 | 1 | 1899 | 0 | 0 | 0 | 0 | 0 | 0 | 0 | 0 | 0 | 0 | 0 | 0 | 0 | 0 | 0 | 0 |
| Nabokov | Vladimir | Invitation to a beheading | | RUSSIA | 0 | 1935 | 1 | 1899 | 0 | 0 | 0 | 0 | 0 | 0 | 0 | 0 | 0 | 0 | 0 | 0 | 0 | 0 | 0 | 0 |
| Nádas | Péter | Parallel Stories | | HUNGARY | 0 | 2005 | 1 | 1942 | 1 | 0 | 0 | 0 | 0 | 0 | 0 | 1 | 0 | 0 | 0 | 0 | 0 | 0 | 0 | 0 |
| Nafisi | Azar | Reading Lolita in Tehran | | IRAN | 1 | 2003 | 4 | 1948 | 0 | 0 | 0 | 0 | 0 | 0 | 0 | 0 | 0 | 0 | 0 | 0 | 0 | 0 | 0 | 0 |
| Nafisi | Azar | Things I've Been Silent About | | IRAN | 1 | 2008 | 4 | 1948 | 0 | 0 | 0 | 0 | 0 | 0 | 0 | 0 | 0 | 0 | 0 | 0 | 0 | 0 | 0 | 0 |
| Naipaul | VS | The enigma of arrival | | TRINIDAD | 0 | 1987 | 3 | 1932 | 0 | 1 | 0 | 0 | 0 | 0 | 0 | 0 | 0 | 0 | 0 | 0 | 0 | 0 | 0 | 0 |
| Naipaul | VS | A bend in the river | | TRINIDAD | 0 | 1979 | 3 | 1932 | 0 | 1 | 0 | 0 | 0 | 0 | 0 | 0 | 0 | 0 | 0 | 0 | 0 | 0 | 0 | 0 |
| Naipaul | VS | A house for Mr Biswas | | TRINIDAD | 0 | 1961 | 3 | 1932 | 0 | 1 | 0 | 0 | 0 | 0 | 0 | 0 | 0 | 0 | 0 | 0 | 0 | 0 | 0 | 0 |
| Naipaul | VS | In a Free State | | TRINIDAD | 0 | 1971 | 3 | 1932 | 0 | 1 | 0 | 1 | 0 | 0 | 0 | 0 | 0 | 0 | 0 | 0 | 0 | 0 | 0 | 0 |
| Nair | Anita | Ladies coupe | | INDIA | 1 | 2001 | 4 | 1966 | 0 | 0 | 0 | 0 | 0 | 0 | 0 | 0 | 0 | 0 | 0 | 0 | 0 | 0 | 0 | 0 |
| Nair | Anita | Mistress | | INDIA | 1 | 2005 | 4 | 1966 | 0 | 0 | 0 | 0 | 0 | 0 | 0 | 0 | 0 | 0 | 0 | 0 | 0 | 0 | 0 | 0 |
| ansubuga Makum | Jennifer | A Girl is A Body of Water |  | UGANDA | 1 | 2020 | 5 | 1975 | 0 | 0 | 0 | 0 | 0 | 0 | 0 | 0 | 0 | 0 | 0 | 0 | 0 | 0 | 0 | 0 |
| Narayan | RK | A Tiger for Malgudi | | INDIA | 0 | 1983 | 4 | 1906 | 0 | 0 | 0 | 0 | 0 | 0 | 0 | 0 | 0 | 0 | 0 | 0 | 0 | 0 | 0 | 0 |
| Narayan | RK | Swami and Friends | | INDIA | 0 | 1935 | 4 | 1906 | 0 | 0 | 0 | 0 | 0 | 0 | 0 | 0 | 0 | 0 | 0 | 0 | 0 | 0 | 0 | 0 |
| Narayan | RK | The guide | | INDIA | 0 | 1958 | 4 | 1906 | 0 | 0 | 0 | 0 | 0 | 0 | 0 | 0 | 0 | 0 | 0 | 0 | 0 | 0 | 0 | 0 |
| Nasrallah | Ibrahim | Time of White Horses | | JORDAN | 0 | 2007 | 4 | 1954 | 1 | 0 | 0 | 0 | 0 | 0 | 0 | 0 | 0 | 0 | 0 | 0 | 0 | 0 | 0 | 0 |
| Nasrin | Taslima | French Lover | | BANGLADESH | 1 | 2002 | 4 | 1962 | 1 | 0 | 0 | 0 | 0 | 0 | 0 | 0 | 0 | 0 | 0 | 0 | 0 | 0 | 0 | 0 |
| Nasrin | Taslima | Lajja | | BANGLADESH | 1 | 1993 | 4 | 1962 | 1 | 0 | 0 | 0 | 0 | 0 | 0 | 0 | 0 | 0 | 0 | 0 | 0 | 0 | 0 | 0 |
| Nelson | Blake | Paranoid park | | USA | 0 | 2006 | 2 | 1964 | 0 | 0 | 0 | 0 | 0 | 0 | 0 | 0 | 0 | 0 | 0 | 0 | 0 | 0 | 0 | 0 |
| Nelson | Maggie | The Argonauts | | USA | 1 | 2015 | 2 | 1973 | 0 | 0 | 0 | 0 | 0 | 0 | 0 | 0 | 0 | 0 | 0 | 0 | 1 | 0 | 0 | 0 |
| Némirovsky | Irène | Suite francaise | | FRANCE | 1 | 2004 | 1 | 1903 | 1 | 0 | 0 | 0 | 0 | 0 | 0 | 0 | 0 | 0 | 0 | 0 | 0 | 0 | 0 | 0 |
| Némirovsky | Irène | The dogs and wolves | | FRANCE | 1 | 1940 | 1 | 1903 | 1 | 0 | 0 | 0 | 0 | 0 | 0 | 0 | 0 | 0 | 0 | 0 | 0 | 0 | 0 | 0 |
| Nesbit | Edith | The Railway Children | | UK | 1 | 1906 | 1 | 1858 | 0 | 0 | 0 | 0 | 0 | 0 | 0 | 0 | 0 | 0 | 0 | 0 | 0 | 0 | 0 | 0 |
| Nesbit | Edith | Five Children and It | | UK | 1 | 1902 | 1 | 1858 | 0 | 0 | 0 | 0 | 0 | 0 | 0 | 0 | 0 | 0 | 0 | 0 | 0 | 0 | 0 | 0 |
| Nesbit | Edith | The Story of the Treasure Seekers | | UK | 1 | 1899 | 1 | 1858 | 0 | 0 | 0 | 0 | 0 | 0 | 0 | 0 | 0 | 0 | 0 | 0 | 0 | 0 | 0 | 0 |
| Nesbit | Edith | The Enchanted Castle | | UK | 1 | 1907 | 1 | 1858 | 0 | 0 | 0 | 0 | 0 | 0 | 0 | 0 | 0 | 0 | 0 | 0 | 0 | 0 | 0 | 0 |
| Nesbit | Edith | The Book Of Dragons | | UK | 1 | 1899 | 1 | 1858 | 0 | 0 | 0 | 0 | 0 | 0 | 0 | 0 | 0 | 0 | 0 | 0 | 0 | 0 | 0 | 0 |
| Nettel | Guadalupe | After the winter | | MEXICO | 1 | 2014 | 3 | 1973 | 1 | 0 | 0 | 0 | 0 | 0 | 0 | 0 | 0 | 0 | 0 | 0 | 0 | 0 | 0 | 1 |
| Nettel | Guadalupe | The body where I was born | | MEXICO | 1 | 2011 | 3 | 1973 | 1 | 0 | 0 | 0 | 0 | 0 | 0 | 0 | 0 | 0 | 0 | 0 | 0 | 0 | 0 | 0 |
| Neuman | Andrés | Fracture | | ARGENTINA | 0 | 2018 | 3 | 1977 | 1 | 0 | 0 | 0 | 0 | 0 | 0 | 0 | 0 | 0 | 0 | 0 | 0 | 0 | 0 | 0 |
| Neville | Stuart | Collusion | | IRELAND | 0 | 2010 | 1 | 1972 | 0 | 0 | 0 | 0 | 0 | 0 | 0 | 0 | 0 | 0 | 0 | 0 | 1 | 0 | 0 | 0 |
| Nevo | Eshkol | Three floors up | | ISRAEL | 0 | 2015 | 4 | 1971 | 1 | 0 | 0 | 0 | 0 | 0 | 0 | 0 | 0 | 0 | 0 | 0 | 0 | 0 | 0 | 0 |
| Nevo | Eshkol | World cup wishes | | ISRAEL | 0 | 2007 | 4 | 1971 | 1 | 0 | 0 | 0 | 0 | 0 | 0 | 0 | 0 | 0 | 0 | 0 | 0 | 0 | 0 | 0 |
| Newby | Percy Howard | Something to Answer For | | UK | 0 | 1968 | 1 | 1918 | 0 | 0 | 0 | 1 | 0 | 0 | 0 | 0 | 0 | 0 | 0 | 0 | 0 | 0 | 0 | 0 |
| Nguyen | Viet Thanh | The Sympathizer | | VIETNAM | 0 | 2015 | 4 | 1971 | 0 | 0 | 0 | 0 | 1 | 0 | 0 | 0 | 0 | 0 | 0 | 0 | 1 | 0 | 0 | 0 |
| Nguyen | Viet Thanh | g Ever Dies: Vietnam and the Memory | | VIETNAM | 0 | 2016 | 4 | 1971 | 0 | 0 | 0 | 0 | 0 | 0 | 0 | 0 | 0 | 0 | 0 | 0 | 0 | 0 | 0 | 0 |
| Nhật Ánh | Nguyễn | Give Me a Ticket to Childhood | | VIETNAM | 0 | 2007 | 4 | 1955 | 1 | 0 | 0 | 0 | 0 | 0 | 0 | 0 | 0 | 0 | 0 | 0 | 0 | 0 | 0 | 0 |
| Nicholls | David | Us | | UK | 0 | 2014 | 1 | 1966 | 0 | 0 | 0 | 0 | 0 | 0 | 0 | 0 | 0 | 1 | 0 | 0 | 0 | 0 | 0 | 0 |
| Nicholls | David | One day | | UK | 0 | 2009 | 1 | 1966 | 0 | 0 | 0 | 0 | 0 | 0 | 0 | 0 | 0 | 1 | 0 | 0 | 1 | 0 | 0 | 0 |
| Nievo | Ippolito | Confessions of an italian | | ITALY | 0 | 1867 | 1 | 1831 | 1 | 0 | 0 | 0 | 0 | 0 | 0 | 0 | 0 | 0 | 0 | 0 | 0 | 0 | 0 | 0 |
| Niffenegger | Audrey | The time traveler's wife | | USA | 1 | 2003 | 2 | 1963 | 0 | 0 | 0 | 0 | 0 | 0 | 0 | 0 | 0 | 1 | 0 | 0 | 0 | 0 | 0 | 0 |
| Nin | Anaïs | Delta of Venus | | USA | 1 | 1977 | 2 | 1903 | 0 | 0 | 0 | 0 | 0 | 0 | 0 | 0 | 0 | 0 | 0 | 0 | 0 | 0 | 0 | 0 |
| Ninh | Bao | The Sorrow of War | | VIETNAM | 0 | 1990 | 4 | 1952 | 1 | 0 | 0 | 0 | 0 | 0 | 0 | 0 | 0 | 0 | 0 | 0 | 0 | 0 | 0 | 0 |
| Noah | Trevor | Born a crime | | SOUTH AFRICA | 0 | 2016 | 5 | 1984 | 0 | 0 | 0 | 0 | 0 | 0 | 0 | 0 | 0 | 0 | 0 | 0 | 0 | 0 | 0 | 0 |
| Nothomb | Amélie | Hygiene and the assassin | | BELGIUM | 1 | 1992 | 1 | 1966 | 1 | 0 | 0 | 0 | 0 | 0 | 0 | 0 | 0 | 0 | 0 | 0 | 0 | 0 | 0 | 0 |
| Novalis | Novalis | Henry of Ofterdingen | | GERMANY | 0 | 1802 | 1 | 1772 | 1 | 0 | 0 | 0 | 0 | 0 | 0 | 0 | 0 | 0 | 0 | 0 | 0 | 0 | 0 | 0 |
| Nwokolo | Chuma | The Extinction of Menai | | NIGERIA | 0 | 2018 | 5 | 1963 | 0 | 0 | 0 | 0 | 0 | 0 | 0 | 0 | 0 | 0 | 0 | 0 | 0 | 0 | 0 | 0 |
| O'Brien | Edna | Country girl | | IRELAND | 1 | 1960 | 1 | 1930 | 0 | 0 | 0 | 0 | 0 | 0 | 0 | 0 | 0 | 0 | 0 | 0 | 0 | 0 | 0 | 0 |
| O'Connor | Flannery | The violent bear it away | | USA | 1 | 1960 | 2 | 1925 | 0 | 0 | 0 | 0 | 0 | 0 | 0 | 0 | 0 | 0 | 0 | 0 | 0 | 0 | 0 | 0 |
| O'Connor | Flannery | A Good Man is Hard to Find | | USA | 1 | 1955 | 2 | 1925 | 0 | 0 | 0 | 0 | 0 | 0 | 0 | 0 | 0 | 0 | 0 | 0 | 0 | 0 | 0 | 0 |
| O'Connor | Edwin | The Edge of Sadness | | USA | 0 | 1961 | 2 | 1918 | 0 | 0 | 0 | 0 | 1 | 0 | 0 | 0 | 0 | 0 | 0 | 0 | 0 | 0 | 0 | 0 |
| O'Dell | Tawni | Back Roads | | USA | 1 | 2000 | 2 | 1964 | 0 | 0 | 0 | 0 | 0 | 0 | 0 | 0 | 0 | 0 | 0 | 0 | 0 | 0 | 0 | 0 |
| O'Hagan | Andrew | The secret life | | UK | 0 | 2017 | 1 | 1968 | 0 | 0 | 0 | 0 | 0 | 0 | 0 | 0 | 0 | 0 | 0 | 0 | 0 | 0 | 0 | 0 |
| Oates | Joyce Carol | Black water | | USA | 1 | 1992 | 2 | 1938 | 0 | 0 | 0 | 0 | 0 | 0 | 0 | 0 | 0 | 0 | 0 | 0 | 0 | 0 | 0 | 0 |
| Oates | Joyce Carol | Blonde | | USA | 1 | 2000 | 2 | 1938 | 0 | 0 | 0 | 0 | 0 | 0 | 0 | 0 | 0 | 0 | 0 | 0 | 0 | 0 | 0 | 0 |
| Oates | Joyce Carol | We Were the Mulvaneys | | USA | 1 | 1996 | 2 | 1938 | 0 | 0 | 0 | 0 | 0 | 0 | 0 | 0 | 0 | 0 | 0 | 0 | 0 | 0 | 0 | 0 |
| Oates | Joyce Carol | What I Lived For | | USA | 1 | 1994 | 2 | 1938 | 0 | 0 | 0 | 0 | 0 | 0 | 0 | 0 | 0 | 0 | 0 | 0 | 0 | 0 | 0 | 0 |
| Obioma | Chigozie | The fishermen | | NIGERIA | 0 | 2015 | 5 | 1986 | 0 | 0 | 0 | 0 | 0 | 0 | 0 | 0 | 0 | 0 | 0 | 0 | 0 | 0 | 0 | 0 |
| Ōe | Kenzaburō | The changeling | | JAPAN | 0 | 2000 | 4 | 1935 | 1 | 1 | 0 | 0 | 0 | 0 | 0 | 0 | 0 | 0 | 0 | 0 | 0 | 0 | 0 | 0 |
| Ōe | Kenzaburō | Death by water | | JAPAN | 0 | 2009 | 4 | 1935 | 1 | 1 | 0 | 0 | 0 | 0 | 0 | 0 | 0 | 0 | 0 | 0 | 0 | 0 | 0 | 0 |
| Ōe | Kenzaburō | A personal matter | | JAPAN | 0 | 1964 | 4 | 1935 | 1 | 1 | 0 | 0 | 0 | 0 | 0 | 0 | 0 | 0 | 0 | 0 | 0 | 0 | 0 | 0 |
| Ogawa | Yōko | Hotel Iris | | JAPAN | 1 | 1996 | 4 | 1962 | 1 | 0 | 0 | 0 | 0 | 0 | 0 | 0 | 0 | 0 | 0 | 0 | 0 | 0 | 0 | 0 |
| Ogawa | Yōko | Revenge | | JAPAN | 1 | 1998 | 4 | 1962 | 1 | 0 | 0 | 0 | 0 | 0 | 0 | 0 | 0 | 0 | 0 | 0 | 0 | 0 | 0 | 0 |
| Ogawa | Yōko | The memory police | | JAPAN | 1 | 1994 | 4 | 1962 | 1 | 0 | 0 | 0 | 0 | 0 | 0 | 0 | 0 | 0 | 0 | 0 | 1 | 0 | 0 | 0 |
| Ohanesian | Aline | Orhan's inheritance | | KUWAIT | 1 | 2015 | 4 | ? | 0 | 0 | 0 | 0 | 0 | 0 | 0 | 0 | 0 | 0 | 0 | 0 | 0 | 0 | 0 | 0 |
| Okri | Ben | Astonishing the Gods | | NIGERIA | 0 | 1995 | 5 | 1959 | 0 | 0 | 0 | 0 | 0 | 0 | 0 | 0 | 0 | 0 | 0 | 0 | 0 | 0 | 0 | 0 |
| Okri | Ben | Songs of Enchantment | | NIGERIA | 0 | 1993 | 5 | 1959 | 0 | 0 | 0 | 0 | 0 | 0 | 0 | 0 | 0 | 0 | 0 | 0 | 0 | 0 | 0 | 0 |
| Okri | Ben | The Famished Road | | NIGERIA | 0 | 1991 | 5 | 1959 | 0 | 0 | 0 | 1 | 0 | 0 | 0 | 0 | 0 | 0 | 0 | 0 | 0 | 0 | 0 | 0 |
| Omotoso | Yewande | The woman next door | | NIGERIA | 1 | 2016 | 5 | 1980 | 0 | 0 | 0 | 0 | 0 | 0 | 0 | 0 | 0 | 0 | 0 | 0 | 0 | 0 | 0 | 0 |
| Ondaatje | Michael | The English Patient | | SRI LANKA | 0 | 1992 | 4 | 1943 | 0 | 0 | 0 | 1 | 0 | 0 | 0 | 0 | 0 | 0 | 0 | 0 | 0 | 0 | 0 | 0 |
| Ondjaki | Ondjaki | Transparent City | | ANGOLA | 0 | 2012 | 5 | 1977 | 1 | 0 | 0 | 0 | 0 | 0 | 0 | 0 | 0 | 0 | 0 | 0 | 0 | 0 | 0 | 0 |
| Onuzo | Chibundu | The spider king's daughter | | NIGERIA | 1 | 2012 | 5 | 1991 | 0 | 0 | 0 | 0 | 0 | 0 | 0 | 0 | 0 | 0 | 0 | 0 | 0 | 0 | 0 | 0 |
| Oppenheim | Edward Phillips | The Great Impersonation | | UK | 0 | 1920 | 1 | 1866 | 0 | 0 | 0 | 0 | 0 | 0 | 0 | 0 | 0 | 0 | 0 | 0 | 0 | 0 | 0 | 0 |
| Oppenheim | Edward Phillips | The Wicked Marquis | | UK | 0 | 1919 | 1 | 1866 | 0 | 0 | 0 | 0 | 0 | 0 | 0 | 0 | 0 | 0 | 0 | 0 | 0 | 0 | 0 | 0 |
| Oppenheim | Edward Phillips | General Besserley’s Puzzle Box | | UK | 0 | 1935 | 1 | 1866 | 0 | 0 | 0 | 0 | 0 | 0 | 0 | 0 | 0 | 0 | 0 | 0 | 0 | 0 | 0 | 0 |
| Orange | Tommy | There there | | USA | 0 | 2019 | 2 | 1982 | 0 | 0 | 0 | 0 | 0 | 0 | 0 | 0 | 0 | 0 | 0 | 1 | 0 | 0 | 0 | 1 |

| **SURNAME** | **NAME** | | **TITLE** | | **NATIONALITY** | | **GENDER** | **PUB_YEAR** | **CONTINENT** | **DATE OF BIRTH** | **TRANSLATED** | **NOBEL** | **NEUSTADT** | **BOOKER** | **PULITZER** | **NEBULA** | **CAINE** | **FRANZ KAFKA** | **NBA** | **BBA** | **WOMEN'S PRIZE FOR FICTION** | **NEW YORK TIMES** | **THE GUARDIAN** | **THE NEW YORKER** | **BBC** | **ATLANTIC** |
| --- | --- | --- | --- | --- | --- | --- | --- | --- | --- | --- | --- | --- | --- | --- | --- | --- | --- | --- | --- | --- | --- | --- | --- | --- | --- | --- |
| Orczy | Emma | | | The Scarlet Pimpernel | HUNGARY | | 1 | 1905 | 1 | 1865 | 0 | 0 | 0 | 0 | 0 | 0 | 0 | 0 | 0 | 0 | 0 | 0 | 0 | 0 | 0 | 0 |
| Orczy | Emma | | | The Bronze Eagle | HUNGARY | | 1 | 1915 | 1 | 1865 | 0 | 0 | 0 | 0 | 0 | 0 | 0 | 0 | 0 | 0 | 0 | 0 | 0 | 0 | 0 | 0 |
| Orga | Irfan | | | Portrait of a turkish family | TURKEY | | 0 | 1950 | 4 | 1908 | 1 | 0 | 0 | 0 | 0 | 0 | 0 | 0 | 0 | 0 | 0 | 0 | 0 | 0 | 0 | 0 |
| Ortese | Anna Maria | | | Neapolitan chronicles | ITALY | | 1 | 1953 | 1 | 1914 | 1 | 0 | 0 | 0 | 0 | 0 | 0 | 0 | 0 | 0 | 0 | 0 | 0 | 0 | 0 | 0 |
| Orwell | George | | | 1984 | UK | | 0 | 1949 | 1 | 1903 | 0 | 0 | 0 | 0 | 0 | 0 | 0 | 0 | 0 | 0 | 0 | 0 | 0 | 0 | 1 | 0 |
| Orwell | George | | | Animal farm | UK | | 0 | 1945 | 1 | 1903 | 0 | 0 | 0 | 0 | 0 | 0 | 0 | 0 | 0 | 0 | 0 | 0 | 0 | 0 | 1 | 0 |
| Owuor | vonne Adhiamb | | | Dust | KENYA | | 1 | 2014 | 5 | 1968 | 1 | 0 | 0 | 0 | 0 | 0 | 0 | 0 | 0 | 0 | 0 | 0 | 0 | 0 | 0 | 0 |
| Owuor | vonne Adhiamb | | | The Dragonfly Sea | KENYA | | 1 | 2019 | 5 | 1968 | 1 | 0 | 0 | 0 | 0 | 0 | 0 | 0 | 0 | 0 | 0 | 0 | 0 | 0 | 0 | 0 |
| Oyeyemi | Helen | | | Boy, snow, bird | UK | | 1 | 2014 | 1 | 1984 | 0 | 0 | 0 | 0 | 0 | 0 | 0 | 0 | 0 | 0 | 0 | 0 | 0 | 0 | 0 | 0 |
| Oz | Amos | | | A tale of love and darkness | ISRAEL | | 0 | 2002 | 4 | 1939 | 1 | 0 | 0 | 0 | 0 | 0 | 0 | 1 | 0 | 0 | 0 | 0 | 0 | 0 | 0 | 0 |
| Oz | Amos | | | Black Box | ISRAEL | | 0 | 1987 | 4 | 1939 | 1 | 0 | 0 | 0 | 0 | 0 | 0 | 1 | 0 | 0 | 0 | 0 | 0 | 0 | 0 | 0 |
| Oz | Amos | | | Don't Call It Night | ISRAEL | | 0 | 1994 | 4 | 1939 | 1 | 0 | 0 | 0 | 0 | 0 | 0 | 1 | 0 | 0 | 0 | 0 | 0 | 0 | 0 | 0 |
| Ozeki | Ruth | | | A Tale for the Time Being | USA | | 1 | 2013 | 2 | 1956 | 0 | 0 | 0 | 0 | 0 | 0 | 0 | 0 | 0 | 0 | 0 | 0 | 0 | 0 | 0 | 0 |
| Paasilinna | Arto | | | The year of the hare | FINLAND | | 0 | 1975 | 1 | 1942 | 1 | 0 | 0 | 0 | 0 | 0 | 0 | 0 | 0 | 0 | 0 | 0 | 0 | 0 | 0 | 0 |
| Pacat | CS | | | The summer palace | AUSTRALIA | | 1 | 2018 | 6 | ? | 0 | 0 | 0 | 0 | 0 | 0 | 0 | 0 | 0 | 0 | 0 | 0 | 0 | 0 | 0 | 0 |
| Palacio | RJ | | | Wonder | USA | | 1 | 2012 | 2 | 1964 | 0 | 0 | 0 | 0 | 0 | 0 | 0 | 0 | 0 | 0 | 0 | 0 | 0 | 0 | 0 | 0 |
| Palahniuk | Chuck | | | Fight club | USA | | 0 | 1996 | 2 | 1962 | 0 | 0 | 0 | 0 | 0 | 0 | 0 | 0 | 0 | 0 | 0 | 0 | 0 | 0 | 0 | 0 |
| Palahniuk | Chuck | | | Lullaby | USA | | 0 | 2002 | 2 | 1962 | 0 | 0 | 0 | 0 | 0 | 0 | 0 | 0 | 0 | 0 | 0 | 0 | 0 | 0 | 0 | 0 |
| Palahniuk | Chuck | | | Rant | USA | | 0 | 2007 | 2 | 1962 | 0 | 0 | 0 | 0 | 0 | 0 | 0 | 0 | 0 | 0 | 0 | 0 | 0 | 0 | 0 | 0 |
| Pamuk | Orhan | | | My name is red | TURKEY | | 0 | 1998 | 4 | 1952 | 1 | 1 | 0 | 0 | 0 | 0 | 0 | 0 | 0 | 0 | 0 | 0 | 0 | 0 | 0 | 0 |
| Pamuk | Orhan | | | The black book | TURKEY | | 0 | 1990 | 4 | 1952 | 1 | 1 | 0 | 0 | 0 | 0 | 0 | 0 | 0 | 0 | 0 | 0 | 0 | 0 | 0 | 0 |
| Pamuk | Orhan | | | Snow | TURKEY | | 0 | 2002 | 4 | 1952 | 1 | 1 | 0 | 0 | 0 | 0 | 0 | 0 | 0 | 0 | 0 | 1 | 0 | 0 | 0 | 0 |
| Pamuk | Orhan | | | Silent house | TURKEY | | 0 | 1983 | 4 | 1952 | 1 | 1 | 0 | 0 | 0 | 0 | 0 | 0 | 0 | 0 | 0 | 0 | 0 | 0 | 0 | 0 |
| Parajuly | Prajwal | | | The Gurkha's Daughter: Stories | INDIA | | 0 | 2012 | 4 | 1984 | 0 | 0 | 0 | 0 | 0 | 0 | 0 | 0 | 0 | 0 | 0 | 0 | 0 | 0 | 0 | 0 |
| Parajuly | Prajwal | | | Land Where I Flee | INDIA | | 0 | 2013 | 4 | 1984 | 0 | 0 | 0 | 0 | 0 | 0 | 0 | 0 | 0 | 0 | 0 | 0 | 0 | 0 | 0 | 0 |
| Park | Yeonmi | | | In order to live | NORTH KOREA | | 1 | 2015 | 4 | 1993 | 0 | 0 | 0 | 0 | 0 | 0 | 0 | 0 | 0 | 0 | 0 | 0 | 0 | 0 | 0 | 0 |
| Parkin | Gaile | | | Baking Cakes in Kigali | ZAMBIA | | 1 | 2009 | 5 | ? | 0 | 0 | 0 | 0 | 0 | 0 | 0 | 0 | 0 | 0 | 0 | 0 | 0 | 0 | 0 | 0 |
| Parks | Tim | | | Europa | UK | | 0 | 1997 | 1 | 1954 | 0 | 0 | 0 | 0 | 0 | 0 | 0 | 0 | 0 | 0 | 0 | 0 | 0 | 0 | 0 | 0 |
| Parsipur | Shahrnush | | | Women Without Men | IRAN | | 1 | 1990 | 4 | 1946 | 1 | 0 | 0 | 0 | 0 | 0 | 0 | 0 | 0 | 0 | 0 | 0 | 0 | 0 | 0 | 0 |
| Pasolini | Pier Paolo | | | The ragazzi | ITALY | | 0 | 1955 | 1 | 1922 | 1 | 0 | 0 | 0 | 0 | 0 | 0 | 0 | 0 | 0 | 0 | 0 | 0 | 0 | 0 | 0 |
| Passarlay | Gulwali | | | The lightless sky | AFGHANISTAN | | 0 | 2015 | 4 | 1994 | 0 | 0 | 0 | 0 | 0 | 0 | 0 | 0 | 0 | 0 | 0 | 0 | 0 | 0 | 0 | 0 |
| Passos | John Dos | | | The 42nd parallel | USA | | 0 | 1930 | 2 | 1896 | 0 | 0 | 0 | 0 | 0 | 0 | 0 | 0 | 0 | 0 | 0 | 0 | 0 | 0 | 0 | 0 |
| Pasternak | Boris | | | Doctor Zhivago | RUSSIA | | 0 | 1957 | 1 | 1890 | 1 | 1 | 0 | 0 | 0 | 0 | 0 | 0 | 0 | 0 | 0 | 0 | 0 | 0 | 0 | 0 |
| Paton | Alan | | | Cry, The Beloved Country | SOUTH AFRICA | | 0 | 1948 | 5 | 1903 | 0 | 0 | 0 | 0 | 0 | 0 | 0 | 0 | 0 | 0 | 0 | 0 | 0 | 0 | 0 | 0 |
| Patterson | James | | | Kiss the girls | USA | | 0 | 1995 | 2 | 1947 | 0 | 0 | 0 | 0 | 0 | 0 | 0 | 0 | 0 | 0 | 0 | 0 | 0 | 0 | 0 | 0 |
| Patterson | James | | | Step on a crack | USA | | 0 | 2007 | 2 | 1947 | 0 | 0 | 0 | 0 | 0 | 0 | 0 | 0 | 0 | 0 | 0 | 0 | 0 | 0 | 0 | 0 |
| Pavese | Cesare | | | The beautiful summer | ITALY | | 0 | 1949 | 1 | 1908 | 1 | 0 | 0 | 0 | 0 | 0 | 0 | 0 | 0 | 0 | 0 | 0 | 0 | 0 | 0 | 0 |
| Pelevin | Victor | | | Buddha's little finger | RUSSIA | | 0 | 1996 | 1 | 1962 | 1 | 0 | 0 | 0 | 0 | 0 | 0 | 0 | 0 | 0 | 0 | 0 | 0 | 0 | 0 | 0 |
| Pelevin | Victor | | | Babylon | RUSSIA | | 0 | 1999 | 1 | 1962 | 1 | 0 | 0 | 0 | 0 | 0 | 0 | 0 | 0 | 0 | 0 | 0 | 0 | 0 | 0 | 0 |
| Pennac | Daniel | | | The eye of the wolf | FRANCE | | 0 | 1984 | 1 | 1944 | 1 | 0 | 0 | 0 | 0 | 0 | 0 | 0 | 0 | 0 | 0 | 0 | 0 | 0 | 0 | 0 |
| Perec | Georges | | | e Art and Craft of Approaching Your He | FRANCE | | 0 | 2008 | 1 | 1936 | 1 | 0 | 0 | 0 | 0 | 0 | 0 | 0 | 0 | 0 | 0 | 0 | 0 | 0 | 0 | 0 |
| Perec | Georges | | | Life a user 's manual | FRANCE | | 0 | 1978 | 1 | 1936 | 1 | 0 | 0 | 0 | 0 | 0 | 0 | 0 | 0 | 0 | 0 | 0 | 0 | 0 | 0 | 0 |
| Pérez-Reverte | Arturo | | | Captain Alatriste | SPAIN | | 0 | 1996 | 1 | 1951 | 1 | 0 | 0 | 0 | 0 | 0 | 0 | 0 | 0 | 0 | 0 | 0 | 0 | 0 | 0 | 0 |
| Pérez-Reverte | Arturo | | | Purity of blood | SPAIN | | 0 | 1997 | 1 | 1951 | 1 | 0 | 0 | 0 | 0 | 0 | 0 | 0 | 0 | 0 | 0 | 0 | 0 | 0 | 0 | 0 |
| Pérez-Reverte | Arturo | | | The cavalier in the yellow doublet | SPAIN | | 0 | 2003 | 1 | 1951 | 1 | 0 | 0 | 0 | 0 | 0 | 0 | 0 | 0 | 0 | 0 | 0 | 0 | 0 | 0 | 0 |
| Perutz | Leo | | | The swedish cavalier | AUSTRIA | | 0 | 1936 | 1 | 1882 | 1 | 0 | 0 | 0 | 0 | 0 | 0 | 0 | 0 | 0 | 0 | 0 | 0 | 0 | 0 | 0 |
| Perutz | Leo | | | By night under the stone bridge | AUSTRIA | | 0 | 1953 | 1 | 1882 | 1 | 0 | 0 | 0 | 0 | 0 | 0 | 0 | 0 | 0 | 0 | 0 | 0 | 0 | 0 | 0 |
| Pessoa | Fernando | | | The book of disquiet | PORTUGAL | | 0 | 1982 | 1 | 1888 | 1 | 0 | 0 | 0 | 0 | 0 | 0 | 0 | 0 | 0 | 0 | 0 | 0 | 0 | 0 | 0 |
| Petterson | Per | | | I refuse | NORWAY | | 0 | 2015 | 1 | 1952 | 1 | 0 | 0 | 0 | 0 | 0 | 0 | 0 | 0 | 0 | 0 | 0 | 0 | 0 | 0 | 0 |
| Phillips | Caryl | | | A Distant Shore | S. KITTS | | 0 | 2003 | 3 | 1958 | 0 | 0 | 0 | 0 | 0 | 0 | 0 | 0 | 0 | 0 | 0 | 0 | 0 | 0 | 0 | 0 |
| Phillips | Caryl | | | Crossing the River | S. KITTS | | 0 | 1993 | 3 | 1958 | 0 | 0 | 0 | 0 | 0 | 0 | 0 | 0 | 0 | 0 | 0 | 0 | 0 | 0 | 0 | 0 |
| Phillips | Caryl | | | Dancing in the Dark | S. KITTS | | 0 | 2005 | 3 | 1958 | 0 | 0 | 0 | 0 | 0 | 0 | 0 | 0 | 0 | 0 | 0 | 0 | 1 | 0 | 0 | 0 |
| Pierre | DBC | | | Vernon God Little | AUSTRALIA | | 0 | 2003 | 6 | 1961 | 0 | 0 | 0 | 1 | 0 | 0 | 0 | 0 | 0 | 0 | 0 | 0 | 0 | 0 | 0 | 0 |
| Piglia | Ricardo | | | Target in the night | ARGENTINA | | 0 | 2010 | 3 | 1941 | 1 | 0 | 0 | 0 | 0 | 0 | 0 | 0 | 0 | 0 | 0 | 0 | 0 | 0 | 0 | 0 |
| Piglia | Ricardo | | | The absent city | ARGENTINA | | 0 | 1992 | 3 | 1941 | 1 | 0 | 0 | 0 | 0 | 0 | 0 | 0 | 0 | 0 | 0 | 0 | 0 | 0 | 0 | 0 |
| Piñeiro | Claudia | | | All yours | ARGENTINA | | 1 | 2006 | 3 | 1960 | 1 | 0 | 0 | 0 | 0 | 0 | 0 | 0 | 0 | 0 | 0 | 0 | 0 | 0 | 0 | 0 |
| Piñeiro | Claudia | | | Betty Boo | ARGENTINA | | 1 | 2011 | 3 | 1960 | 1 | 0 | 0 | 0 | 0 | 0 | 0 | 0 | 0 | 0 | 0 | 0 | 0 | 0 | 0 | 0 |
| Piñeiro | Claudia | | | Thursday Night Widows | ARGENTINA | | 1 | 2005 | 3 | 1960 | 1 | 0 | 0 | 0 | 0 | 0 | 0 | 0 | 0 | 0 | 0 | 0 | 0 | 0 | 0 | 0 |
| Pirandello | Luigi | | | The late Mattia Pascal | ITALY | | 0 | 1904 | 1 | 1867 | 1 | 1 | 0 | 0 | 0 | 0 | 0 | 0 | 0 | 0 | 0 | 0 | 0 | 0 | 0 | 0 |
| Pirsig | Robert | en and the art of motorcycle maintenanc | | | | USA | 0 | 1974 | 2 | 1928 | 0 | 0 | 0 | 0 | 0 | 0 | 0 | 0 | 0 | 0 | 0 | 0 | 0 | 0 | 0 | 0 |
| Plath | Sylvia | | | The bell jar | USA | | 1 | 1963 | 2 | 1932 | 0 | 0 | 0 | 0 | 0 | 0 | 0 | 0 | 0 | 0 | 0 | 0 | 0 | 0 | 0 | 0 |
| Platonov | Andrei | | | Happy Moscow | RUSSIA | | 0 | 1996 | 1 | 1899 | 1 | 0 | 0 | 0 | 0 | 0 | 0 | 0 | 0 | 0 | 0 | 0 | 0 | 0 | 0 | 0 |
| Poe | Edgar Allan | | | Tales of mistery and terror | USA | | 0 | 1845 | 2 | 1809 | 0 | 0 | 0 | 0 | 0 | 0 | 0 | 0 | 0 | 0 | 0 | 0 | 0 | 0 | 0 | 0 |
| Pollan | Michael | | | The omnivore's dilemma | USA | | 0 | 2006 | 2 | 1955 | 0 | 0 | 0 | 0 | 0 | 0 | 0 | 0 | 0 | 0 | 0 | 0 | 0 | 0 | 0 | 0 |
| Poole | Ernest | | | His Family | USA | | 0 | 1917 | 2 | 1880 | 0 | 0 | 0 | 0 | 1 | 0 | 0 | 0 | 0 | 0 | 0 | 0 | 0 | 0 | 0 | 0 |
| Porter | Max | | | Lanny | UK | | 0 | 2020 | 1 | 1981 | 0 | 0 | 0 | 0 | 0 | 0 | 0 | 0 | 0 | 0 | 0 | 0 | 1 | 0 | 0 | 0 |
| Porter | Katherine | | | Collected Stories | USA | | 1 | 1965 | 2 | 1890 | 0 | 0 | 0 | 0 | 1 | 0 | 0 | 0 | 1 | 0 | 0 | 0 | 0 | 0 | 0 | 0 |
| Powers | Richard | | | The Overstory | USA | | 0 | 2018 | 2 | 1957 | 0 | 0 | 0 | 0 | 1 | 0 | 0 | 0 | 0 | 0 | 0 | 0 | 0 | 0 | 0 | 0 |
| Powers | Kevin | | | The Yellow Birds | USA | | 0 | 2012 | 2 | 1980 | 0 | 0 | 0 | 0 | 0 | 0 | 0 | 0 | 0 | 0 | 0 | 1 | 0 | 0 | 0 | 0 |
| Proulx | Annie | | | Brokeback mountain | USA | | 1 | 1997 | 2 | 1935 | 0 | 0 | 0 | 0 | 0 | 0 | 0 | 0 | 0 | 0 | 0 | 0 | 0 | 0 | 0 | 0 |
| Proulx | Annie | | | The Shipping News | USA | | 1 | 1993 | 2 | 1935 | 0 | 0 | 0 | 0 | 1 | 0 | 0 | 0 | 1 | 0 | 0 | 0 | 0 | 0 | 0 | 0 |
| Proust | Marcel | | | In search of lost time | FRANCE | | 0 | 1913 | 1 | 1871 | 1 | 0 | 0 | 0 | 0 | 0 | 0 | 0 | 0 | 0 | 0 | 0 | 0 | 0 | 0 | 0 |
| Puig | Manuel | | | Heartbreak tango | ARGENTINA | | 0 | 1969 | 3 | 1932 | 1 | 0 | 0 | 0 | 0 | 0 | 0 | 0 | 0 | 0 | 0 | 0 | 0 | 0 | 0 | 0 |
| Puig | Manuel | | | Kiss of the Spider Woman | ARGENTINA | | 0 | 1976 | 3 | 1932 | 1 | 0 | 0 | 0 | 0 | 0 | 0 | 0 | 0 | 0 | 0 | 0 | 0 | 0 | 0 | 0 |
| Punke | Michael | | | The revenant | USA | | 0 | 2002 | 2 | 1964 | 0 | 0 | 0 | 0 | 0 | 0 | 0 | 0 | 0 | 0 | 0 | 0 | 0 | 0 | 0 | 0 |
| Puzo | Mario | | | The Godfather | USA | | 0 | 1969 | 2 | 1920 | 0 | 0 | 0 | 0 | 0 | 0 | 0 | 0 | 0 | 0 | 0 | 0 | 0 | 0 | 1 | 0 |
| Pym | Barbara | | | Quartet in Autumn | UK | | 1 | 1977 | 1 | 1913 | 0 | 0 | 0 | 0 | 0 | 0 | 0 | 0 | 0 | 0 | 0 | 0 | 0 | 0 | 0 | 0 |
| Pynchon | Thomas | | | The crying lot of 49 | USA | | 0 | 1966 | 2 | 1937 | 0 | 0 | 0 | 0 | 0 | 0 | 0 | 0 | 0 | 0 | 0 | 0 | 0 | 0 | 0 | 0 |
| Pynchon | Thomas | | | Gravity's Rainbow | USA | | 0 | 1973 | 2 | 1937 | 0 | 0 | 0 | 0 | 0 | 0 | 0 | 0 | 1 | 0 | 0 | 0 | 0 | 0 | 0 | 0 |
| Queen | Ellery | | | The French Powder Mystery | USA | | 0 | 1930 | 2 | 1905 | 0 | 0 | 0 | 0 | 0 | 0 | 0 | 0 | 0 | 0 | 0 | 0 | 0 | 0 | 0 | 0 |
| Queen | Ellery | | | The Egyptian Cross Mystery | USA | | 0 | 1932 | 2 | 1905 | 0 | 0 | 0 | 0 | 0 | 0 | 0 | 0 | 0 | 0 | 0 | 0 | 0 | 0 | 0 | 0 |
| Queneau | Raymond | | | The blue flowers | FRANCE | | 0 | 1965 | 1 | 1903 | 1 | 0 | 0 | 0 | 0 | 0 | 0 | 0 | 0 | 0 | 0 | 0 | 0 | 0 | 0 | 0 |
| Quindlen | Anna | | | Black and Blue | USA | | 1 | 1998 | 2 | 1953 | 0 | 0 | 0 | 0 | 0 | 0 | 0 | 0 | 0 | 0 | 0 | 0 | 0 | 0 | 0 | 0 |
| Quiroga | Horacio | | | The Decapitated Chicken | URUGUAY | | 0 | 1925 | 3 | 1878 | 1 | 0 | 0 | 0 | 0 | 0 | 0 | 0 | 0 | 0 | 0 | 0 | 0 | 0 | 0 | 0 |
| Radcliffe | Ann | | | A Sicilian Romance | UK | | 1 | 1790 | 1 | 1764 | 0 | 0 | 0 | 0 | 0 | 0 | 0 | 0 | 0 | 0 | 0 | 0 | 0 | 0 | 0 | 0 |
| Radcliffe | Ann | | | The Mysteries of Udolpho | UK | | 1 | 1794 | 1 | 1764 | 0 | 0 | 0 | 0 | 0 | 0 | 0 | 0 | 0 | 0 | 0 | 0 | 0 | 0 | 0 | 0 |
| Radiguet | Raymond | | | The devil in the flesh | FRANCE | | 0 | 1923 | 1 | 1903 | 1 | 0 | 0 | 0 | 0 | 0 | 0 | 0 | 0 | 0 | 0 | 0 | 0 | 0 | 0 | 0 |

| **SURNAME** | | **NAME** | **TITLE** | | **NATIONALITY** | **GENDER** | **PUB_YEAR** | **CONTINENT** | **DATE OF BIRTH** | **TRANSLATED** | **NOBEL** | **NEUSTADT** | **BOOKER** | **PULITZER** | **NEBULA** | **CAINE** | **FRANZ KAFKA** | **NBA** | **BBA** | **WOMEN'S PRIZE FOR FICTION** | **NEW YORK TIMES** | **THE GUARDIAN** | **THE NEW YORKER** | **BBC** | **ATLANTIC** |
| --- | --- | --- | --- | --- | --- | --- | --- | --- | --- | --- | --- | --- | --- | --- | --- | --- | --- | --- | --- | --- | --- | --- | --- | --- | --- |
| Rahimi | Atiq | | | Earth and Ashes | AFGHANISTAN | 0 | 2003 | 4 | 1962 | 1 | 0 | 0 | 0 | 0 | 0 | 0 | 0 | 0 | 0 | 0 | 0 | 0 | 0 | 0 | 0 |
| Rahimi | Atiq | | | The Patience Stone | AFGHANISTAN | 0 | 2008 | 4 | 1962 | 1 | 0 | 0 | 0 | 0 | 0 | 0 | 0 | 0 | 0 | 0 | 0 | 0 | 0 | 0 | 0 |
| Rand | Edward | | | At the black rocks | USA | 0 | 1910 | 2 | 1871 | 0 | 0 | 0 | 0 | 0 | 0 | 0 | 0 | 0 | 0 | 0 | 0 | 0 | 0 | 0 | 0 |
| Rand | Edward | | | The Knights of the White Shield | USA | 0 | 1886 | 2 | 1871 | 0 | 0 | 0 | 0 | 0 | 0 | 0 | 0 | 0 | 0 | 0 | 0 | 0 | 0 | 0 | 0 |
| Randall | FJ | | | Love and the Ironmonger | UK | 0 | 1908 | 1 | ? | 0 | 0 | 0 | 0 | 0 | 0 | 0 | 0 | 0 | 0 | 0 | 0 | 0 | 0 | 0 | 0 |
| Rankin | Carroll | | | Dandelion Cottage | USA | 1 | 1904 | 2 | 1864 | 0 | 0 | 0 | 0 | 0 | 0 | 0 | 0 | 0 | 0 | 0 | 0 | 0 | 0 | 0 | 0 |
| Rankin | Carroll | | | The Adopting of Rosa Marie | USA | 1 | 1908 | 2 | 1864 | 0 | 0 | 0 | 0 | 0 | 0 | 0 | 0 | 0 | 0 | 0 | 0 | 0 | 0 | 0 | 0 |
| Rankin | Carroll | | | The Castaways of Pete's Patch | USA | 1 | 1911 | 2 | 1864 | 0 | 0 | 0 | 0 | 0 | 0 | 0 | 0 | 0 | 0 | 0 | 0 | 0 | 0 | 0 | 0 |
| Rankin | Carroll | | | The Cinder Pound | USA | 1 | 1915 | 2 | 1864 | 0 | 0 | 0 | 0 | 0 | 0 | 0 | 0 | 0 | 0 | 0 | 0 | 0 | 0 | 0 | 0 |
| Rao | Raja | | | The Cat and Shakespeare | INDIA | 0 | 1965 | 4 | 1908 | 0 | 0 | 1 | 0 | 0 | 0 | 0 | 0 | 0 | 0 | 0 | 0 | 0 | 0 | 0 | 0 |
| Raper | Eleanor | | | The little girl lost | UK | 1 | 1902 | 1 | ? | 0 | 0 | 0 | 0 | 0 | 0 | 0 | 0 | 0 | 0 | 0 | 0 | 0 | 0 | 0 | 0 |
| Ratner | Vaddey | | | In the Shadow of the Banyan | CAMBODIA | 1 | 2012 | 4 | 1970 | 0 | 0 | 0 | 0 | 0 | 0 | 0 | 0 | 0 | 0 | 0 | 0 | 0 | 0 | 0 | 0 |
| Ratner | Vaddey | | | Music of the Ghosts | CAMBODIA | 1 | 2017 | 4 | 1970 | 0 | 0 | 0 | 0 | 0 | 0 | 0 | 0 | 0 | 0 | 0 | 0 | 0 | 0 | 0 | 0 |
| Rawlings | Marjorie Kinnan | | | The Yearling | USA | 1 | 1938 | 2 | 1896 | 0 | 0 | 0 | 0 | 1 | 0 | 0 | 0 | 0 | 0 | 0 | 0 | 0 | 0 | 0 | 0 |
| Ray | Anna | | | Half a Dozen Girls | USA | 1 | 1897 | 2 | 1865 | 0 | 0 | 0 | 0 | 0 | 0 | 0 | 0 | 0 | 0 | 0 | 0 | 0 | 0 | 0 | 0 |
| Ray | Anna | | | In blue creek canon | USA | 1 | 1892 | 2 | 1865 | 0 | 0 | 0 | 0 | 0 | 0 | 0 | 0 | 0 | 0 | 0 | 0 | 0 | 0 | 0 | 0 |
| Ray | Anna | | | Phebe, her profession | USA | 1 | 1902 | 2 | 1865 | 0 | 0 | 0 | 0 | 0 | 0 | 0 | 0 | 0 | 0 | 0 | 0 | 0 | 0 | 0 | 0 |
| Ray | Anna | | | Teddy | USA | 1 | 1898 | 2 | 1865 | 0 | 0 | 0 | 0 | 0 | 0 | 0 | 0 | 0 | 0 | 0 | 0 | 0 | 0 | 0 | 0 |
| Ray | Anna | | | The Brentons | USA | 1 | 1912 | 2 | 1865 | 0 | 0 | 0 | 0 | 0 | 0 | 0 | 0 | 0 | 0 | 0 | 0 | 0 | 0 | 0 | 0 |
| Ray | Anna | | | The Dominant Strain | USA | 1 | 1903 | 2 | 1865 | 0 | 0 | 0 | 0 | 0 | 0 | 0 | 0 | 0 | 0 | 0 | 0 | 0 | 0 | 0 | 0 |
| Raymond | Eveline | | | A Daughter of the Forest | USA | 1 | 1902 | 2 | 1843 | 0 | 0 | 0 | 0 | 0 | 0 | 0 | 0 | 0 | 0 | 0 | 0 | 0 | 0 | 0 | 0 |
| Raymond | Eveline | | | Dorothy at Skyrie | USA | 1 | 1907 | 2 | 1843 | 0 | 0 | 0 | 0 | 0 | 0 | 0 | 0 | 0 | 0 | 0 | 0 | 0 | 0 | 0 | 0 |
| Raymond | Eveline | | | Dorothy on a House Boat | USA | 1 | 1909 | 2 | 1843 | 0 | 0 | 0 | 0 | 0 | 0 | 0 | 0 | 0 | 0 | 0 | 0 | 0 | 0 | 0 | 0 |
| Reach | Angus | | | Claret and Olives | UK | 0 | 1852 | 1 | 1821 | 0 | 0 | 0 | 0 | 0 | 0 | 0 | 0 | 0 | 0 | 0 | 0 | 0 | 0 | 0 | 0 |
| Read | George | | | The Last Cruise of the Saginaw | USA | 0 | 1912 | 2 | 1843 | 0 | 0 | 0 | 0 | 0 | 0 | 0 | 0 | 0 | 0 | 0 | 0 | 0 | 0 | 0 | 0 |
| Reage | Pauline | | | Story of O | FRANCE | 1 | 1954 | 1 | 1907 | 1 | 0 | 0 | 0 | 0 | 0 | 0 | 0 | 0 | 0 | 0 | 0 | 0 | 0 | 0 | 0 |
| Rebreanu | Liviu | | | The Forest of the Hanged | ROMANIA | 0 | 1922 | 1 | 1885 | 1 | 0 | 0 | 0 | 0 | 0 | 0 | 0 | 0 | 0 | 0 | 0 | 0 | 0 | 0 | 0 |
| Rechy | John | | | City of Night | USA | 0 | 1963 | 2 | 1931 | 0 | 0 | 0 | 0 | 0 | 0 | 0 | 0 | 0 | 0 | 0 | 0 | 0 | 0 | 0 | 0 |
| Rechy | John | | | Numbers | USA | 0 | 1967 | 2 | 1931 | 0 | 0 | 0 | 0 | 0 | 0 | 0 | 0 | 0 | 0 | 0 | 0 | 0 | 0 | 0 | 0 |
| Rechy | John | | | Our Lady of Babylon | USA | 0 | 1996 | 2 | 1931 | 0 | 0 | 0 | 0 | 0 | 0 | 0 | 0 | 0 | 0 | 0 | 0 | 0 | 0 | 0 | 0 |
| Rechy | John | | | Rushes | USA | 0 | 1979 | 2 | 1931 | 0 | 0 | 0 | 0 | 0 | 0 | 0 | 0 | 0 | 0 | 0 | 0 | 0 | 0 | 0 | 0 |
| Rechy | John | | | The Coming of the Night | USA | 0 | 1999 | 2 | 1931 | 0 | 0 | 0 | 0 | 0 | 0 | 0 | 0 | 0 | 0 | 0 | 0 | 0 | 0 | 0 | 0 |
| Rechy | John | | | The Vampires | USA | 0 | 1971 | 2 | 1931 | 0 | 0 | 0 | 0 | 0 | 0 | 0 | 0 | 0 | 0 | 0 | 0 | 0 | 0 | 0 | 0 |
| Redfield | James | | | The Celestine prophecy | USA | 0 | 1993 | 2 | 1950 | 0 | 0 | 0 | 0 | 0 | 0 | 0 | 0 | 0 | 0 | 0 | 0 | 0 | 0 | 0 | 0 |
| Reed | Helen | | | Amy in Acadia | CANADA | 1 | 1905 | 2 | ? | 0 | 0 | 0 | 0 | 0 | 0 | 0 | 0 | 0 | 0 | 0 | 0 | 0 | 0 | 0 | 0 |
| Reed | Helen | | | Brenda, Her School and Her Club | CANADA | 1 | 1900 | 2 | ? | 0 | 0 | 0 | 0 | 0 | 0 | 0 | 0 | 0 | 0 | 0 | 0 | 0 | 0 | 0 | 0 |
| Reed | Helen | | | Brenda's Bargain | CANADA | 1 | 1903 | 2 | ? | 0 | 0 | 0 | 0 | 0 | 0 | 0 | 0 | 0 | 0 | 0 | 0 | 0 | 0 | 0 | 0 |
| Reed | Helen | | | Brenda's Ward | CANADA | 1 | 1906 | 2 | ? | 0 | 0 | 0 | 0 | 0 | 0 | 0 | 0 | 0 | 0 | 0 | 0 | 0 | 0 | 0 | 0 |
| Reed | Helen | | | Miss Theodora A West End Story | CANADA | 1 | 1898 | 2 | ? | 0 | 0 | 0 | 0 | 0 | 0 | 0 | 0 | 0 | 0 | 0 | 0 | 0 | 0 | 0 | 0 |
| Reeve | Clara | | | The Old English Baron: A Gothic Story | UK | 1 | 1778 | 1 | 1729 | 0 | 0 | 0 | 0 | 0 | 0 | 0 | 0 | 0 | 0 | 0 | 0 | 0 | 0 | 0 | 0 |
| Reid | Elizabeth | | | Mayne Reid A Memoir of his Life | USA | 1 | 1890 | 2 | ? | 0 | 0 | 0 | 0 | 0 | 0 | 0 | 0 | 0 | 0 | 0 | 0 | 0 | 0 | 0 | 0 |
| Remarque | Erich Maria | | | All quiet on the Western front | GERMANY | 0 | 1929 | 1 | 1898 | 1 | 0 | 0 | 0 | 0 | 0 | 0 | 0 | 0 | 0 | 0 | 0 | 0 | 1 | 0 | 0 |
| Remick | Grace | | | Glenloch Girls | USA | 1 | 1916 | 2 | 1860 | 0 | 0 | 0 | 0 | 0 | 0 | 0 | 0 | 0 | 0 | 0 | 0 | 0 | 0 | 0 | 0 |
| Remoto | Danton | | | Riverrun | PHILIPPINES | 0 | 2015 | 4 | 1963 | 0 | 0 | 0 | 0 | 0 | 0 | 0 | 0 | 0 | 0 | 0 | 0 | 0 | 0 | 0 | 0 |
| Renault | Mary | | | The Last of the Wine | UK | 1 | 1956 | 1 | 1905 | 0 | 0 | 0 | 0 | 0 | 0 | 0 | 0 | 0 | 0 | 0 | 0 | 0 | 0 | 0 | 0 |
| Renault | Mary | | | The Persian Boy | UK | 1 | 1972 | 1 | 1905 | 0 | 0 | 0 | 0 | 0 | 0 | 0 | 0 | 0 | 0 | 0 | 0 | 0 | 0 | 0 | 0 |
| Renault | Mary | | | Fire from Heaven | UK | 1 | 1969 | 1 | 1905 | 0 | 0 | 0 | 0 | 0 | 0 | 0 | 0 | 0 | 0 | 0 | 0 | 0 | 0 | 0 | 0 |
| Renault | Mary | | | Funeral Games | UK | 1 | 1981 | 1 | 1905 | 0 | 0 | 0 | 0 | 0 | 0 | 0 | 0 | 0 | 0 | 0 | 0 | 0 | 0 | 0 | 0 |
| Renault | Mary | | | The Mask of Apollo | UK | 1 | 1966 | 1 | 1905 | 0 | 0 | 0 | 0 | 0 | 0 | 0 | 0 | 0 | 0 | 0 | 0 | 0 | 0 | 0 | 0 |
| Renault | Mary | | | The Praise Singer | UK | 1 | 1978 | 1 | 1905 | 0 | 0 | 0 | 0 | 0 | 0 | 0 | 0 | 0 | 0 | 0 | 0 | 0 | 0 | 0 | 0 |
| Rendell | Ruth | | | Wolf to the Slaughter | UK | 1 | 1967 | 1 | 1930 | 0 | 0 | 0 | 0 | 0 | 0 | 0 | 0 | 0 | 0 | 0 | 0 | 0 | 0 | 0 | 0 |
| Rendell | Ruth | | | Simisola | UK | 1 | 1994 | 1 | 1930 | 0 | 0 | 0 | 0 | 0 | 0 | 0 | 0 | 0 | 0 | 0 | 0 | 0 | 0 | 0 | 0 |
| Restrepo | Laura | | | Delirium | COLOMBIA | 1 | 2004 | 3 | 1950 | 1 | 0 | 0 | 0 | 0 | 0 | 0 | 0 | 0 | 0 | 0 | 0 | 0 | 0 | 0 | 0 |
| Revueltas | José | | | The hole | MEXICO | 0 | 1969 | 3 | 1914 | 1 | 0 | 0 | 0 | 0 | 0 | 0 | 0 | 0 | 0 | 0 | 0 | 0 | 0 | 0 | 0 |
| Reyes | Emma | | | The book of Emma Reyes | COLOMBIA | 1 | 2012 | 3 | 1919 | 1 | 0 | 0 | 0 | 0 | 0 | 0 | 0 | 0 | 0 | 0 | 0 | 0 | 0 | 0 | 0 |
| Reymont | Wladyslaw | | | The Comédienne | POLAND | 0 | 1896 | 1 | 1867 | 1 | 1 | 0 | 0 | 0 | 0 | 0 | 0 | 0 | 0 | 0 | 0 | 0 | 0 | 0 | 0 |
| Reynolds | George | | | The Mysteries of London, v. 1/4 | UK | 0 | 1844 | 1 | 1814 | 0 | 0 | 0 | 0 | 0 | 0 | 0 | 0 | 0 | 0 | 0 | 0 | 0 | 0 | 0 | 0 |
| Reynolds | George | | | Wagner | UK | 0 | 1846 | 1 | 1814 | 0 | 0 | 0 | 0 | 0 | 0 | 0 | 0 | 0 | 0 | 0 | 0 | 0 | 0 | 0 | 0 |
| Reynolds | Sheri | | | The Rapture of Canaan | USA | 1 | 1995 | 2 | 1967 | 0 | 0 | 0 | 0 | 0 | 0 | 0 | 0 | 0 | 0 | 0 | 0 | 0 | 0 | 0 | 0 |
| Rhodes | Eugene | | | Bransford of Rainbow Range | USA | 0 | 1913 | 2 | 1869 | 0 | 0 | 0 | 0 | 0 | 0 | 0 | 0 | 0 | 0 | 0 | 0 | 0 | 0 | 0 | 0 |
| Rhodes | Eugene | | | Copper Streak Trail | USA | 0 | 1917 | 2 | 1869 | 0 | 0 | 0 | 0 | 0 | 0 | 0 | 0 | 0 | 0 | 0 | 0 | 0 | 0 | 0 | 0 |
| Rhodes | Eugene | | | Stepsons of Light | USA | 0 | 1921 | 2 | 1869 | 0 | 0 | 0 | 0 | 0 | 0 | 0 | 0 | 0 | 0 | 0 | 0 | 0 | 0 | 0 | 0 |
| Rhodes | Eugene | | | The Desire of the Moth | USA | 0 | 1916 | 2 | 1869 | 0 | 0 | 0 | 0 | 0 | 0 | 0 | 0 | 0 | 0 | 0 | 0 | 0 | 0 | 0 | 0 |
| Rhys | Jean | | | Wide Sargasso Sea | DOMINICA | 1 | 1966 | 3 | 1890 | 0 | 0 | 0 | 0 | 0 | 0 | 0 | 0 | 0 | 0 | 0 | 0 | 0 | 0 | 0 | 0 |
| Rhys | Jean | | | Good Morning, Midnight | DOMINICA | 1 | 1939 | 3 | 1890 | 0 | 0 | 0 | 0 | 0 | 0 | 0 | 0 | 0 | 0 | 0 | 0 | 0 | 0 | 0 | 0 |
| Rice | Anne | | | Interview with the Vampire | USA | 1 | 1976 | 2 | 1941 | 0 | 0 | 0 | 0 | 0 | 0 | 0 | 0 | 0 | 0 | 0 | 0 | 0 | 0 | 0 | 0 |
| Rice | Anne | | | Memnoch the Devil | USA | 1 | 1995 | 2 | 1941 | 0 | 0 | 0 | 0 | 0 | 0 | 0 | 0 | 0 | 0 | 0 | 0 | 0 | 0 | 0 | 0 |
| Rice | Anne | | | Merrick | USA | 1 | 2000 | 2 | 1941 | 0 | 0 | 0 | 0 | 0 | 0 | 0 | 0 | 0 | 0 | 0 | 0 | 0 | 0 | 0 | 0 |
| Rice | Anne | | | The Queen of the Damned | USA | 1 | 1988 | 2 | 1941 | 0 | 0 | 0 | 0 | 0 | 0 | 0 | 0 | 0 | 0 | 0 | 0 | 0 | 0 | 0 | 0 |
| Rice | Anne | | | The Tale of the Body Thief | USA | 1 | 1992 | 2 | 1941 | 0 | 0 | 0 | 0 | 0 | 0 | 0 | 0 | 0 | 0 | 0 | 0 | 0 | 0 | 0 | 0 |
| Rice | Anne | | | The Vampire Armand | USA | 1 | 1998 | 2 | 1941 | 0 | 0 | 0 | 0 | 0 | 0 | 0 | 0 | 0 | 0 | 0 | 0 | 0 | 0 | 0 | 0 |
| Rice | Anne | | | The Vampire Lestat | USA | 1 | 1985 | 2 | 1941 | 0 | 0 | 0 | 0 | 0 | 0 | 0 | 0 | 0 | 0 | 0 | 0 | 0 | 0 | 0 | 0 |
| Rice | Alfred | | | on Girl A Tale of American Life in the N | UK | 0 | 1914 | 1 | 1851 | 0 | 0 | 0 | 0 | 0 | 0 | 0 | 0 | 0 | 0 | 0 | 0 | 0 | 0 | 0 | 0 |
| Rice | George | | | My Adventures with Your Money | USA | 0 | 1913 | 2 | 1870 | 0 | 0 | 0 | 0 | 0 | 0 | 0 | 0 | 0 | 0 | 0 | 0 | 0 | 0 | 0 | 0 |
| Rich | Charles | | | A Voyage with Captain Dynamite | USA | 0 | 1896 | 2 | ? | 0 | 0 | 0 | 0 | 0 | 0 | 0 | 0 | 0 | 0 | 0 | 0 | 0 | 0 | 0 | 0 |
| Richardson | Samuel | | | Pamela | UK | 0 | 1740 | 1 | 1689 | 0 | 0 | 0 | 0 | 0 | 0 | 0 | 0 | 0 | 0 | 0 | 0 | 0 | 0 | 0 | 0 |
| Richardson | Albert | | | t Service, the Field, the Dungeon, and t | USA | 0 | 1865 | 2 | 1833 | 0 | 0 | 0 | 0 | 0 | 0 | 0 | 0 | 0 | 0 | 0 | 0 | 0 | 0 | 0 | 0 |
| Richardson | Dorothy | | | The long day | UK | 1 | 1905 | 1 | 1873 | 0 | 0 | 0 | 0 | 0 | 0 | 0 | 0 | 0 | 0 | 0 | 0 | 0 | 0 | 0 | 0 |
| Richardson | Sarah | | | Life in the Grey Nunnery at Montreal | CANADA | 1 | 1858 | 2 | ? | 0 | 0 | 0 | 0 | 0 | 0 | 0 | 0 | 0 | 0 | 0 | 0 | 0 | 0 | 0 | 0 |
| Richler | Mordecai | | | Barney's version | CANADA | 0 | 1997 | 2 | 1931 | 0 | 0 | 0 | 0 | 0 | 0 | 0 | 0 | 0 | 0 | 0 | 0 | 0 | 0 | 0 | 0 |
| Richler | Mordecai | | | Solomon Gursky Was Here | CANADA | 0 | 1989 | 2 | 1931 | 0 | 0 | 0 | 0 | 0 | 0 | 0 | 0 | 0 | 0 | 0 | 0 | 0 | 0 | 0 | 0 |
| Richmond | Grace | | | Strawberry Acres | USA | 1 | 1911 | 2 | 1866 | 0 | 0 | 0 | 0 | 0 | 0 | 0 | 0 | 0 | 0 | 0 | 0 | 0 | 0 | 0 | 0 |
| Richmond | Grace | | | The Indifference of Juliet | USA | 1 | 1902 | 2 | 1866 | 0 | 0 | 0 | 0 | 0 | 0 | 0 | 0 | 0 | 0 | 0 | 0 | 0 | 0 | 0 | 0 |
| Richmond | Grace | | | The second violin | USA | 1 | 1905 | 2 | 1866 | 0 | 0 | 0 | 0 | 0 | 0 | 0 | 0 | 0 | 0 | 0 | 0 | 0 | 0 | 0 | 0 |
| Richmond | Grace | | | Under the Country Sky | USA | 1 | 1916 | 2 | 1866 | 0 | 0 | 0 | 0 | 0 | 0 | 0 | 0 | 0 | 0 | 0 | 0 | 0 | 0 | 0 | 0 |
| Richter | Conrad | | | The Town | USA | 0 | 1950 | 2 | 1890 | 0 | 0 | 0 | 0 | 1 | 0 | 0 | 0 | 0 | 0 | 0 | 0 | 0 | 0 | 0 | 0 |
| Riddle | Albert | | | Bart Ridgeley: A story of Northern Ohio | USA | 0 | 1873 | 2 | 1816 | 0 | 0 | 0 | 0 | 0 | 0 | 0 | 0 | 0 | 0 | 0 | 0 | 0 | 0 | 0 | 0 |

| **SURNAME** | **NAME** | | **TITLE** | **NATIONALITY** | | **GENDER** | **PUB_YEAR** | **CONTINENT** | **DATE OF BIRTH** | **TRANSLATED** | **NOBEL** | **NEUSTADT** | **BOOKER** | **PULITZER** | **NEBULA** | **CAINE** | **FRANZ KAFKA** | **NBA** | **BBA** | **WOMEN'S PRIZE FOR FICTION** | **NEW YORK TIMES** | **THE GUARDIAN** | **THE NEW YORKER** | **BBC** | **ATLANTIC** |
| --- | --- | --- | --- | --- | --- | --- | --- | --- | --- | --- | --- | --- | --- | --- | --- | --- | --- | --- | --- | --- | --- | --- | --- | --- | --- |
| Rihani | Ameen | The Book of Khalid | | | LEBANON | 0 | 1911 | 4 | 1876 | 0 | 0 | 0 | 0 | 0 | 0 | 0 | 0 | 0 | 0 | 0 | 0 | 0 | 0 | 0 | 0 |
| Riley | Henry | The Puddleford Papers | | | USA | 0 | 1859 | 2 | 1813 | 0 | 0 | 0 | 0 | 0 | 0 | 0 | 0 | 0 | 0 | 0 | 0 | 0 | 0 | 0 | 0 |
| Ringland | Holly | The Lost Flowers of Alice Hart | | | AUSTRALIA | 1 | 2018 | 6 | 1981 | 0 | 0 | 0 | 0 | 0 | 0 | 0 | 0 | 0 | 0 | 0 | 0 | 0 | 0 | 0 | 0 |
| Ritchie | Eric | With Botha in the Field | | | UK | 0 | 1915 | 1 | ? | 0 | 0 | 0 | 0 | 0 | 0 | 0 | 0 | 0 | 0 | 0 | 0 | 0 | 0 | 0 | 0 |
| Rives | Hallie | A Furnace of Earth | | | USA | 1 | 1900 | 2 | 1874 | 0 | 0 | 0 | 0 | 0 | 0 | 0 | 0 | 0 | 0 | 0 | 0 | 0 | 0 | 0 | 0 |
| Rives | Hallie | Satan Sanderson | | | USA | 1 | 1907 | 2 | 1874 | 0 | 0 | 0 | 0 | 0 | 0 | 0 | 0 | 0 | 0 | 0 | 0 | 0 | 0 | 0 | 0 |
| Rives | Hallie | The Kingdom of Slender Swords | | | USA | 1 | 1910 | 2 | 1874 | 0 | 0 | 0 | 0 | 0 | 0 | 0 | 0 | 0 | 0 | 0 | 0 | 0 | 0 | 0 | 0 |
| Rives | Hallie | The Valiants of Virginia | | | USA | 1 | 1912 | 2 | 1874 | 0 | 0 | 0 | 0 | 0 | 0 | 0 | 0 | 0 | 0 | 0 | 0 | 0 | 0 | 0 | 0 |
| Robbins | Harold | Goodbye, Janette | | | USA | 0 | 1981 | 2 | 1916 | 0 | 0 | 0 | 0 | 0 | 0 | 0 | 0 | 0 | 0 | 0 | 0 | 0 | 0 | 0 | 0 |
| Robbins | Harold | The Dream Merchants | | | USA | 0 | 1949 | 2 | 1916 | 0 | 0 | 0 | 0 | 0 | 0 | 0 | 0 | 0 | 0 | 0 | 0 | 0 | 0 | 0 | 0 |
| Robbins | Harold | Tycoon | | | USA | 0 | 1997 | 2 | 1916 | 0 | 0 | 0 | 0 | 0 | 0 | 0 | 0 | 0 | 0 | 0 | 0 | 0 | 0 | 0 | 0 |
| Roberts | Gregory | Shantaram | | | AUSTRALIA | 0 | 2003 | 6 | 1952 | 0 | 0 | 0 | 0 | 0 | 0 | 0 | 0 | 0 | 0 | 0 | 0 | 0 | 0 | 0 | 0 |
| Roberts | Gregory | The mountain shadow | | | AUSTRALIA | 0 | 2015 | 6 | 1952 | 0 | 0 | 0 | 0 | 0 | 0 | 0 | 0 | 0 | 0 | 0 | 0 | 0 | 0 | 0 | 0 |
| Roberts | Nora | Hidden star | | | USA | 1 | 1997 | 2 | 1950 | 0 | 0 | 0 | 0 | 0 | 0 | 0 | 0 | 0 | 0 | 0 | 0 | 0 | 0 | 0 | 0 |
| Robin | Gallienne | Where Deep Seas Moan | | | UK | 1 | 1907 | 1 | ? | 0 | 0 | 0 | 0 | 0 | 0 | 0 | 0 | 0 | 0 | 0 | 0 | 0 | 0 | 0 | 0 |
| Robinson | Marilynne | Lila | | | USA | 1 | 2014 | 2 | 1943 | 0 | 0 | 0 | 0 | 0 | 0 | 0 | 0 | 0 | 0 | 0 | 0 | 1 | 0 | 1 | 0 |
| Robinson | Marilynne | Gilead | | | USA | 1 | 2004 | 2 | 1943 | 0 | 0 | 0 | 0 | 1 | 0 | 0 | 0 | 0 | 0 | 0 | 1 | 0 | 0 | 0 | 0 |
| Robinson | CH | Longhead: The Story of the First Fire | | | USA | 0 | 1913 | 2 | 1843 | 0 | 0 | 0 | 0 | 0 | 0 | 0 | 0 | 0 | 0 | 0 | 0 | 0 | 0 | 0 | 0 |
| Robinson | Eliot | Smiles': A Rose of the Cumberlands | | | USA | 0 | 1919 | 2 | ? | 0 | 0 | 0 | 0 | 0 | 0 | 0 | 0 | 0 | 0 | 0 | 0 | 0 | 0 | 0 | 0 |
| Robinson | Derek | Goshawk Squadron | | | UK | 0 | 1971 | 1 | 1932 | 0 | 0 | 0 | 0 | 0 | 0 | 0 | 0 | 0 | 0 | 0 | 0 | 0 | 0 | 0 | 0 |
| Roche | Arthur | Find The Woman | | | USA | 0 | 1921 | 2 | 1883 | 0 | 0 | 0 | 0 | 0 | 0 | 0 | 0 | 0 | 0 | 0 | 0 | 0 | 0 | 0 | 0 |
| Roffey | Monique | House of Ashes | | | TRINIDAD | 1 | 2014 | 3 | 1965 | 0 | 0 | 0 | 0 | 0 | 0 | 0 | 0 | 0 | 0 | 0 | 0 | 0 | 0 | 0 | 0 |
| Roffey | Monique | The White Woman on the Green Bicycle | | | TRINIDAD | 1 | 2009 | 3 | 1965 | 0 | 0 | 0 | 0 | 0 | 0 | 0 | 0 | 0 | 0 | 0 | 0 | 0 | 0 | 0 | 0 |
| Rogers | Rosemary | The Wildest Heart | | | USA | 1 | 1974 | 2 | 1932 | 0 | 0 | 0 | 0 | 0 | 0 | 0 | 0 | 0 | 0 | 0 | 0 | 0 | 0 | 0 | 0 |
| Rogers | Rosemary | Bound by love | | | USA | 1 | 2009 | 2 | 1932 | 0 | 0 | 0 | 0 | 0 | 0 | 0 | 0 | 0 | 0 | 0 | 0 | 0 | 0 | 0 | 0 |
| Rolland | Romain | Clerambault | | | FRANCE | 0 | 1920 | 1 | 1866 | 1 | 1 | 0 | 0 | 0 | 0 | 0 | 0 | 0 | 0 | 0 | 0 | 0 | 0 | 0 | 0 |
| Roncagliolo | Santiago | Red april | | | PERU | 0 | 2006 | 3 | 1975 | 1 | 0 | 0 | 0 | 0 | 0 | 0 | 0 | 0 | 0 | 0 | 0 | 1 | 0 | 0 | 0 |
| Rong | Jiang | Wolf Totem | | | CHINA | 0 | 2004 | 4 | 1946 | 1 | 0 | 0 | 0 | 0 | 0 | 0 | 0 | 0 | 0 | 0 | 0 | 0 | 0 | 0 | 0 |
| Rooney | Sally | Conversations with friends | | | IRELAND | 1 | 2017 | 1 | 1991 | 0 | 0 | 0 | 0 | 0 | 0 | 0 | 0 | 0 | 0 | 0 | 0 | 1 | 1 | 0 | 0 |
| Roosevelt | Wyn | Frontier Boys in Frisco | | | USA | 0 | 1911 | 2 | 1870 | 0 | 0 | 0 | 0 | 0 | 0 | 0 | 0 | 0 | 0 | 0 | 0 | 0 | 0 | 0 | 0 |
| Roosevelt | Wyn | Frontier Boys in the South Seas | | | USA | 0 | 1912 | 2 | 1870 | 0 | 0 | 0 | 0 | 0 | 0 | 0 | 0 | 0 | 0 | 0 | 0 | 0 | 0 | 0 | 0 |
| Roosevelt | Wyn | Frontier Boys on the Coast | | | USA | 0 | 1909 | 2 | 1870 | 0 | 0 | 0 | 0 | 0 | 0 | 0 | 0 | 0 | 0 | 0 | 0 | 0 | 0 | 0 | 0 |
| Roosevelt | Wyn | The Frontier Boys in the Grand Canyon | | | USA | 0 | 1908 | 2 | 1870 | 0 | 0 | 0 | 0 | 0 | 0 | 0 | 0 | 0 | 0 | 0 | 0 | 0 | 0 | 0 | 0 |
| Roosevelt | Wyn | The Frontier Boys in the Sierras | | | USA | 0 | 1909 | 2 | 1870 | 0 | 0 | 0 | 0 | 0 | 0 | 0 | 0 | 0 | 0 | 0 | 0 | 0 | 0 | 0 | 0 |
| Roper | Edward | A Claim on Klondyke | | | UK | 0 | 1899 | 1 | 1857 | 0 | 0 | 0 | 0 | 0 | 0 | 0 | 0 | 0 | 0 | 0 | 0 | 0 | 0 | 0 | 0 |
| Rosebery | Lord | Lord Chatham | | | UK | 0 | 1910 | 1 | 1847 | 0 | 0 | 0 | 0 | 0 | 0 | 0 | 0 | 0 | 0 | 0 | 0 | 0 | 0 | 0 | 0 |
| Rosoff | Meg | How I live now | | | USA | 1 | 2004 | 2 | 1956 | 0 | 0 | 0 | 0 | 0 | 0 | 0 | 0 | 0 | 0 | 0 | 0 | 0 | 0 | 0 | 0 |
| Ross | Albert | A New Sensation | | | USA | 0 | 1898 | 2 | 1851 | 0 | 0 | 0 | 0 | 0 | 0 | 0 | 0 | 0 | 0 | 0 | 0 | 0 | 0 | 0 | 0 |
| Ross | Frederick | Bygone London | | | USA | 0 | 1892 | 2 | ? | 0 | 0 | 0 | 0 | 0 | 0 | 0 | 0 | 0 | 0 | 0 | 0 | 0 | 0 | 0 | 0 |
| Roth | Philip | The human stain | | | USA | 0 | 2000 | 2 | 1933 | 0 | 0 | 0 | 1 | 0 | 0 | 0 | 1 | 0 | 0 | 0 | 0 | 0 | 0 | 0 | 0 |
| Roth | Philip | Portnoy's complaint | | | USA | 0 | 1969 | 2 | 1933 | 0 | 0 | 0 | 1 | 0 | 0 | 0 | 1 | 0 | 0 | 0 | 0 | 0 | 0 | 0 | 0 |
| Roth | Philip | When she was good | | | USA | 0 | 1967 | 2 | 1933 | 0 | 0 | 0 | 1 | 0 | 0 | 0 | 1 | 0 | 0 | 0 | 0 | 0 | 0 | 0 | 0 |
| Roth | Philip | The dying animal | | | USA | 0 | 2001 | 2 | 1933 | 0 | 0 | 0 | 1 | 0 | 0 | 0 | 1 | 0 | 0 | 0 | 0 | 0 | 0 | 0 | 0 |
| Roth | Philip | American pastoral | | | USA | 0 | 1997 | 2 | 1933 | 0 | 0 | 0 | 1 | 1 | 0 | 0 | 1 | 0 | 0 | 0 | 0 | 0 | 0 | 0 | 0 |
| Roth | Philip | Everyman | | | USA | 0 | 2006 | 2 | 1933 | 0 | 0 | 0 | 1 | 0 | 0 | 0 | 1 | 0 | 0 | 0 | 0 | 0 | 0 | 0 | 0 |
| Roth | Joseph | Radetzky March | | | AUSTRIA | 0 | 1932 | 1 | 1894 | 1 | 0 | 0 | 0 | 0 | 0 | 0 | 0 | 0 | 0 | 0 | 0 | 0 | 0 | 0 | 0 |
| Roth | Veronica | Divergent | | | USA | 1 | 2011 | 2 | 1988 | 0 | 0 | 0 | 0 | 0 | 0 | 0 | 0 | 0 | 0 | 0 | 0 | 0 | 0 | 0 | 0 |
| Rothmann | Ralf | To Die in Spring | | | GERMANY | 0 | 2017 | 1 | 1953 | 1 | 0 | 0 | 0 | 0 | 0 | 0 | 0 | 0 | 0 | 0 | 0 | 0 | 0 | 0 | 0 |
| Rowell | Rainbow | Eleanor & Park | | | USA | 1 | 2012 | 2 | 1973 | 0 | 0 | 0 | 0 | 0 | 0 | 0 | 0 | 0 | 0 | 0 | 0 | 0 | 1 | 0 | 0 |
| Rowell | Rainbow | Fangirl | | | USA | 1 | 2013 | 2 | 1973 | 0 | 0 | 0 | 0 | 0 | 0 | 0 | 0 | 0 | 0 | 0 | 0 | 0 | 0 | 0 | 0 |
| Rowland | Helen | The Widow To Say Nothing of the Man | | | USA | 1 | 1908 | 2 | 1875 | 0 | 0 | 0 | 0 | 0 | 0 | 0 | 0 | 0 | 0 | 0 | 0 | 0 | 0 | 0 | 0 |
| Rowling | JK | Harry Potter and the philosopher's stone | | | UK | 1 | 1997 | 1 | 1965 | 0 | 0 | 0 | 0 | 0 | 0 | 0 | 0 | 0 | 1 | 0 | 0 | 0 | 0 | 1 | 0 |
| Rowling | JK | Harry Potter and the chamber of secrets | | | UK | 1 | 1998 | 1 | 1965 | 0 | 0 | 0 | 0 | 0 | 0 | 0 | 0 | 0 | 1 | 0 | 0 | 0 | 0 | 1 | 0 |
| Rowling | JK | Harry Potter and the prisoner of Azkaba | | | UK | 1 | 1999 | 1 | 1965 | 0 | 0 | 0 | 0 | 0 | 0 | 0 | 0 | 0 | 1 | 0 | 0 | 0 | 0 | 1 | 0 |
| Rowling | JK | Harry Potter and the goblet of fire | | | UK | 1 | 2000 | 1 | 1965 | 0 | 0 | 0 | 0 | 0 | 0 | 0 | 0 | 0 | 1 | 0 | 0 | 0 | 0 | 1 | 0 |
| Rowling | JK | Harry Potter and the order of the phoeni | | | UK | 1 | 2003 | 1 | 1965 | 0 | 0 | 0 | 0 | 0 | 0 | 0 | 0 | 0 | 1 | 0 | 0 | 0 | 0 | 0 | 0 |
| Rowling | JK | Harry Potter and the half-blood prince | | | UK | 1 | 2005 | 1 | 1965 | 0 | 0 | 0 | 0 | 0 | 0 | 0 | 0 | 0 | 1 | 0 | 0 | 0 | 0 | 0 | 0 |
| Rowling | JK | Harry Potter and the deathly hallows | | | UK | 1 | 2007 | 1 | 1965 | 0 | 0 | 0 | 0 | 0 | 0 | 0 | 0 | 0 | 1 | 0 | 0 | 0 | 0 | 0 | 0 |
| Roy | Arundhati | The god of small things | | | INDIA | 1 | 1997 | 4 | 1961 | 0 | 0 | 0 | 1 | 0 | 0 | 0 | 0 | 0 | 0 | 0 | 0 | 0 | 0 | 1 | 1 |
| Roy | Arundhati | The ministry of utmost happiness | | | INDIA | 1 | 2017 | 4 | 1961 | 0 | 0 | 0 | 0 | 0 | 0 | 0 | 0 | 0 | 0 | 0 | 0 | 1 | 0 | 0 | 0 |
| Rubens | Bernice | The Elected Member | | | UK | 1 | 1969 | 1 | 1923 | 0 | 0 | 0 | 1 | 0 | 0 | 0 | 0 | 0 | 0 | 0 | 0 | 0 | 0 | 0 | 0 |
| Rubio | Gwyn Hyman | Icy Sparks | | | USA | 1 | 1998 | 2 | 1949 | 0 | 0 | 0 | 0 | 0 | 0 | 0 | 0 | 0 | 0 | 0 | 0 | 0 | 0 | 0 | 0 |
| Ruck | Berta | Miss Million's Maid : A romance | | | UK | 1 | 1915 | 1 | 1878 | 0 | 0 | 0 | 0 | 0 | 0 | 0 | 0 | 0 | 0 | 0 | 0 | 0 | 0 | 0 | 0 |
| Ruck | Berta | The Boy with Wings | | | UK | 1 | 1915 | 1 | 1878 | 0 | 0 | 0 | 0 | 0 | 0 | 0 | 0 | 0 | 0 | 0 | 0 | 0 | 0 | 0 | 0 |
| Ruck | Berta | The Disturbing Charm | | | UK | 1 | 1919 | 1 | 1878 | 0 | 0 | 0 | 0 | 0 | 0 | 0 | 0 | 0 | 0 | 0 | 0 | 0 | 0 | 0 | 0 |
| Rulfo | Juan | The plain in flames | | | MEXICO | 0 | 1953 | 3 | 1917 | 1 | 0 | 0 | 0 | 0 | 0 | 0 | 0 | 0 | 0 | 0 | 0 | 0 | 0 | 0 | 0 |
| Rulfo | Juan | Pedro Paramo | | | MEXICO | 0 | 1955 | 3 | 1917 | 1 | 0 | 0 | 0 | 0 | 0 | 0 | 0 | 0 | 0 | 0 | 0 | 0 | 0 | 0 | 0 |
| Rushdie | Salman | The ground beneath her feet | | | INDIA | 0 | 1999 | 4 | 1947 | 0 | 0 | 0 | 0 | 0 | 0 | 0 | 0 | 0 | 1 | 0 | 0 | 0 | 0 | 0 | 0 |
| Rushdie | Salman | Midnight's children | | | INDIA | 0 | 1981 | 4 | 1947 | 0 | 0 | 0 | 1 | 0 | 0 | 0 | 0 | 0 | 1 | 0 | 0 | 0 | 0 | 1 | 0 |
| Russell | Dora | A Country Sweetheart | | | UK | 1 | 1895 | 1 | ? | 0 | 0 | 0 | 0 | 0 | 0 | 0 | 0 | 0 | 0 | 0 | 0 | 0 | 0 | 0 | 0 |
| Russo | Meredith | If I Was Your Girl | | | USA | 1 | 2016 | 2 | 1986 | 0 | 0 | 0 | 0 | 0 | 0 | 0 | 0 | 0 | 0 | 0 | 0 | 0 | 0 | 0 | 0 |
| Russo | Richard | Empire Falls | | | USA | 0 | 2001 | 2 | 1949 | 0 | 0 | 0 | 0 | 1 | 0 | 0 | 0 | 0 | 0 | 0 | 0 | 0 | 0 | 0 | 0 |
| Ruxton | George | In The Old West | | | UK | 0 | 1915 | 1 | 1821 | 0 | 0 | 0 | 0 | 0 | 0 | 0 | 0 | 0 | 0 | 0 | 0 | 0 | 0 | 0 | 0 |
| Sabatini | Rafael | The Sea Hawk | | | ITALY | 0 | 1915 | 1 | 1875 | 0 | 0 | 0 | 0 | 0 | 0 | 0 | 0 | 0 | 0 | 0 | 0 | 0 | 0 | 0 | 0 |
| Sabatini | Rafael | Captain Blood | | | ITALY | 0 | 1922 | 1 | 1875 | 0 | 0 | 0 | 0 | 0 | 0 | 0 | 0 | 0 | 0 | 0 | 0 | 0 | 0 | 0 | 0 |
| Sabatini | Rafael | Scaramouche | | | ITALY | 0 | 1921 | 1 | 1875 | 0 | 0 | 0 | 0 | 0 | 0 | 0 | 0 | 0 | 0 | 0 | 0 | 0 | 0 | 0 | 0 |
| Sabato | Ernesto | On heroes and tombs | | | ARGENTINA | 0 | 1961 | 3 | 1911 | 1 | 0 | 0 | 0 | 0 | 0 | 0 | 0 | 0 | 0 | 0 | 0 | 0 | 0 | 0 | 0 |
| Sabato | Ernesto | The Tunnel | | | ARGENTINA | 0 | 1948 | 3 | 1911 | 1 | 0 | 0 | 0 | 0 | 0 | 0 | 0 | 0 | 0 | 0 | 0 | 0 | 0 | 0 | 0 |
| Sacheri | Eduardo | The secret in their eyes | | | ARGENTINA | 0 | 2005 | 3 | 1967 | 1 | 0 | 0 | 0 | 0 | 0 | 0 | 0 | 0 | 0 | 0 | 0 | 0 | 0 | 0 | 0 |
| Sacks | Oliver | The river of consciousness | | | UK | 0 | 2017 | 1 | 1933 | 0 | 0 | 0 | 0 | 0 | 0 | 0 | 0 | 0 | 0 | 0 | 0 | 0 | 0 | 0 | 0 |
| Saer | Juan José | La Grande | | | ARGENTINA | 0 | 2005 | 3 | 1937 | 1 | 0 | 0 | 0 | 0 | 0 | 0 | 0 | 0 | 0 | 0 | 0 | 0 | 0 | 0 | 0 |
| Saer | Juan José | The Clouds | | | ARGENTINA | 0 | 1997 | 3 | 1937 | 1 | 0 | 0 | 0 | 0 | 0 | 0 | 0 | 0 | 0 | 0 | 0 | 0 | 0 | 0 | 0 |
| Sagan | Françoise | Bonjour tristesse | | | FRANCE | 1 | 1954 | 1 | 1935 | 1 | 0 | 0 | 0 | 0 | 0 | 0 | 0 | 0 | 0 | 0 | 0 | 0 | 0 | 0 | 0 |
| Sahota | Sunjeev | The Year of the Runaways | | | UK | 0 | 2015 | 1 | 1981 | 0 | 0 | 0 | 0 | 0 | 0 | 0 | 0 | 0 | 0 | 0 | 0 | 1 | 0 | 0 | 0 |
| Sakuraba | Kazuki | Red girls | | | JAPAN | 1 | 2015 | 4 | 1971 | 1 | 0 | 0 | 0 | 0 | 0 | 0 | 0 | 0 | 0 | 0 | 0 | 0 | 0 | 0 | 0 |
| Salih | Tayeb | Season of Migration to the North | | | SUDAN | 0 | 1966 | 5 | 1929 | 1 | 0 | 0 | 0 | 0 | 0 | 0 | 0 | 0 | 0 | 0 | 0 | 0 | 0 | 0 | 0 |
| Salinger | JD | Catcher in the rye | | | USA | 0 | 1951 | 2 | 1919 | 0 | 0 | 0 | 0 | 0 | 0 | 0 | 0 | 0 | 0 | 0 | 0 | 0 | 0 | 1 | 0 |

| **SURNAME** | **NAME** | **TITLE** | **NATIONALITY** | **GENDER** | **PUB_YEAR** | **CONTINENT** | **DATE OF BIRTH** | **TRANSLATED** | **NOBEL** | **NEUSTADT** | **BOOKER** | **PULITZER** | **NEBULA** | **CAINE** | **FRANZ KAFKA** | **NBA** | **BBA** | **WOMEN'S PRIZE FOR FICTION** | **NEW YORK TIMES** | **THE GUARDIAN** | **THE NEW YORKER** | **BBC** | **ATLANTIC** |
| --- | --- | --- | --- | --- | --- | --- | --- | --- | --- | --- | --- | --- | --- | --- | --- | --- | --- | --- | --- | --- | --- | --- | --- |
| Salinger | JD | Franny and Zooey | USA | 0 | 1961 | 2 | 1919 | 0 | 0 | 0 | 0 | 0 | 0 | 0 | 0 | 0 | 0 | 0 | 0 | 0 | 0 | 0 | 0 |
| Salmawy | Mohamed | Butterfly wings | EGYPT | 0 | 2014 | 5 | 1945 | 1 | 0 | 0 | 0 | 0 | 0 | 0 | 0 | 0 | 0 | 0 | 0 | 0 | 0 | 0 | 0 |
| Sánchez | Clara | The scent of lemon leaves | SPAIN | 1 | 2010 | 1 | 1955 | 1 | 0 | 0 | 0 | 0 | 0 | 0 | 0 | 0 | 0 | 0 | 0 | 0 | 0 | 0 | 0 |
| Sand | George | Indiana | FRANCE | 1 | 1832 | 1 | 1804 | 1 | 0 | 0 | 0 | 0 | 0 | 0 | 0 | 0 | 0 | 0 | 0 | 0 | 0 | 0 | 0 |
| Sand | George | Mauprat | FRANCE | 1 | 1837 | 1 | 1804 | 1 | 0 | 0 | 0 | 0 | 0 | 0 | 0 | 0 | 0 | 0 | 0 | 0 | 0 | 0 | 0 |
| Sand | George | The Countess Von Rudolstadt | FRANCE | 1 | 1843 | 1 | 1804 | 1 | 0 | 0 | 0 | 0 | 0 | 0 | 0 | 0 | 0 | 0 | 0 | 0 | 0 | 0 | 0 |
| Sand | George | Valentine | FRANCE | 1 | 1832 | 1 | 1804 | 1 | 0 | 0 | 0 | 0 | 0 | 0 | 0 | 0 | 0 | 0 | 0 | 0 | 0 | 0 | 0 |
| Sansal | Boualem | 2084 The end of the world | ALGERIA | 0 | 2015 | 5 | 1949 | 1 | 0 | 0 | 0 | 0 | 0 | 0 | 0 | 0 | 0 | 0 | 0 | 0 | 0 | 0 | 0 |
| Sansal | Boualem | The German Mujahid | ALGERIA | 0 | 2008 | 5 | 1949 | 1 | 0 | 0 | 0 | 0 | 0 | 0 | 0 | 0 | 0 | 0 | 0 | 0 | 0 | 0 | 0 |
| Sapienza | Goliarda | The art of joy | ITALY | 1 | 1994 | 1 | 1924 | 1 | 0 | 0 | 0 | 0 | 0 | 0 | 0 | 0 | 0 | 0 | 0 | 0 | 0 | 0 | 0 |
| Saramago | Josè | Blindness | PORTUGAL | 0 | 1995 | 1 | 1922 | 1 | 1 | 0 | 0 | 0 | 0 | 0 | 0 | 0 | 0 | 0 | 0 | 0 | 0 | 0 | 0 |
| Saramago | Josè | Death with interruptions | PORTUGAL | 0 | 2005 | 1 | 1922 | 1 | 1 | 0 | 0 | 0 | 0 | 0 | 0 | 0 | 0 | 0 | 0 | 0 | 0 | 0 | 0 |
| Saramago | Josè | Cain | PORTUGAL | 0 | 2009 | 1 | 1922 | 1 | 1 | 0 | 0 | 0 | 0 | 0 | 0 | 0 | 0 | 0 | 0 | 0 | 0 | 0 | 0 |
| Sartre | Jean-Paul | Nausea | FRANCE | 0 | 1938 | 1 | 1905 | 1 | 1 | 0 | 0 | 0 | 0 | 0 | 0 | 0 | 0 | 0 | 0 | 0 | 0 | 0 | 0 |
| Sartre | Jean-Paul | The wall | FRANCE | 0 | 1939 | 1 | 1905 | 1 | 1 | 0 | 0 | 0 | 0 | 0 | 0 | 0 | 0 | 0 | 0 | 0 | 0 | 0 | 0 |
| Saul | Jack | The Sins of the Cities of the Plain | IRELAND | 0 | 1881 | 1 | 1857 | 0 | 0 | 0 | 0 | 0 | 0 | 0 | 0 | 0 | 0 | 0 | 0 | 0 | 0 | 0 | 0 |
| Saunders | George | Tenth of december | USA | 0 | 2013 | 2 | 1958 | 0 | 0 | 0 | 0 | 0 | 0 | 0 | 0 | 0 | 0 | 0 | 1 | 0 | 0 | 1 | 1 |
| Saunders | George | Lincoln in the Bardo | USA | 0 | 2017 | 2 | 1958 | 0 | 0 | 0 | 1 | 0 | 0 | 0 | 0 | 0 | 0 | 0 | 0 | 1 | 0 | 1 | 0 |
| Saviano | Roberto | Gomorrah | ITALY | 0 | 2006 | 1 | 1979 | 1 | 0 | 0 | 0 | 0 | 0 | 0 | 0 | 0 | 0 | 0 | 0 | 0 | 0 | 0 | 0 |
| Schami | Rafik | The dark side of love | SYRIA | 0 | 2004 | 4 | 1946 | 1 | 0 | 0 | 0 | 0 | 0 | 0 | 0 | 0 | 0 | 0 | 0 | 0 | 0 | 0 | 0 |
| Schami | Rafik | Damascus Nights | SYRIA | 0 | 1989 | 4 | 1946 | 1 | 0 | 0 | 0 | 0 | 0 | 0 | 0 | 0 | 0 | 0 | 0 | 0 | 0 | 0 | 0 |
| Schami | Rafik | The Calligrapher's Secret | SYRIA | 0 | 2008 | 4 | 1946 | 1 | 0 | 0 | 0 | 0 | 0 | 0 | 0 | 0 | 0 | 0 | 0 | 0 | 0 | 0 | 0 |
| Schätzing | Frank | Death and the devil | GERMANY | 0 | 1995 | 1 | 1957 | 1 | 0 | 0 | 0 | 0 | 0 | 0 | 0 | 0 | 0 | 0 | 0 | 0 | 0 | 0 | 0 |
| Schlink | Bernhard | The Reader | GERMANY | 0 | 1995 | 1 | 1944 | 1 | 0 | 0 | 0 | 0 | 0 | 0 | 0 | 0 | 0 | 0 | 0 | 0 | 0 | 0 | 0 |
| Schnitzler | Arthur | Dream story | AUSTRIA | 0 | 1926 | 1 | 1862 | 1 | 0 | 0 | 0 | 0 | 0 | 0 | 0 | 0 | 0 | 0 | 0 | 0 | 0 | 0 | 0 |
| Schwarz | Christina | Drowning Ruth | USA | 1 | 2000 | 2 | 1962 | 0 | 0 | 0 | 0 | 0 | 0 | 0 | 0 | 0 | 0 | 0 | 0 | 0 | 0 | 0 | 0 |
| Schweblin | Samanta | Little eyes | ARGENTINA | 1 | 2018 | 3 | 1978 | 1 | 0 | 0 | 0 | 0 | 0 | 0 | 0 | 0 | 0 | 0 | 0 | 1 | 0 | 0 | 0 |
| Schweblin | Samanta | Fever Dream | ARGENTINA | 1 | 2014 | 3 | 1978 | 1 | 0 | 0 | 0 | 0 | 0 | 0 | 0 | 0 | 0 | 0 | 0 | 1 | 0 | 0 | 0 |
| Sciascia | Leonardo | The day of the owl | ITALY | 0 | 1960 | 1 | 1921 | 1 | 0 | 0 | 0 | 0 | 0 | 0 | 0 | 0 | 0 | 0 | 0 | 0 | 0 | 0 | 0 |
| Sciascia | Leonardo | Equal danger | ITALY | 0 | 1971 | 1 | 1921 | 1 | 0 | 0 | 0 | 0 | 0 | 0 | 0 | 0 | 0 | 0 | 0 | 0 | 0 | 0 | 0 |
| Scott | Walter | Ivanhoe | UK | 0 | 1819 | 1 | 1771 | 0 | 0 | 0 | 0 | 0 | 0 | 0 | 0 | 0 | 0 | 0 | 0 | 0 | 0 | 0 | 0 |
| Scott | Walter | Rob Roy | UK | 0 | 1817 | 1 | 1771 | 0 | 0 | 0 | 0 | 0 | 0 | 0 | 0 | 0 | 0 | 0 | 0 | 0 | 0 | 0 | 0 |
| Scott | Paul | Staying On | UK | 0 | 1977 | 1 | 1920 | 0 | 0 | 0 | 1 | 0 | 0 | 0 | 0 | 0 | 0 | 0 | 0 | 0 | 0 | 0 | 0 |
| Sebald | Winfried Georg | The emigrants | GERMANY | 0 | 1992 | 1 | 1944 | 1 | 0 | 0 | 0 | 0 | 0 | 0 | 0 | 0 | 0 | 0 | 0 | 0 | 0 | 0 | 0 |
| Sebald | Winfried Georg | Austerlitz | GERMANY | 0 | 2001 | 1 | 1944 | 1 | 0 | 0 | 0 | 0 | 0 | 0 | 0 | 0 | 0 | 0 | 0 | 0 | 0 | 0 | 0 |
| Sebold | Alice | Lovely bones | USA | 1 | 2002 | 2 | 1963 | 0 | 0 | 0 | 0 | 0 | 0 | 0 | 0 | 0 | 1 | 0 | 0 | 0 | 0 | 0 | 0 |
| Sedaris | David | When you are engulfed in flames | USA | 0 | 2008 | 2 | 1956 | 0 | 0 | 0 | 0 | 0 | 0 | 0 | 0 | 0 | 0 | 0 | 0 | 0 | 0 | 0 | 0 |
| Segal | Erich | Love Story | USA | 0 | 1970 | 2 | 1937 | 0 | 0 | 0 | 0 | 0 | 0 | 0 | 0 | 0 | 0 | 0 | 0 | 0 | 0 | 0 | 0 |
| Seghers | Anna | The seventh cross | GERMANY | 1 | 1942 | 1 | 1900 | 1 | 0 | 0 | 0 | 0 | 0 | 0 | 0 | 0 | 0 | 0 | 0 | 0 | 0 | 0 | 0 |
| Seiffert | Rachel | The Dark Room | UK | 1 | 2001 | 1 | 1971 | 0 | 0 | 0 | 0 | 0 | 0 | 0 | 0 | 0 | 0 | 0 | 0 | 0 | 0 | 0 | 0 |
| Selasi | Taiye | Ghana must go | NIGERIA | 1 | 2013 | 5 | 1979 | 0 | 0 | 0 | 0 | 0 | 0 | 0 | 0 | 0 | 0 | 0 | 0 | 0 | 0 | 0 | 1 |
| Self | Will | Umbrella | UK | 0 | 2012 | 1 | 1961 | 0 | 0 | 0 | 0 | 0 | 0 | 0 | 0 | 0 | 0 | 0 | 0 | 0 | 0 | 0 | 0 |
| Selvadurai | Shyam | Funny boy | SRI LANKA | 0 | 1994 | 4 | 1965 | 0 | 0 | 0 | 0 | 0 | 0 | 0 | 0 | 0 | 0 | 0 | 0 | 0 | 0 | 0 | 0 |
| Selvon | Sam | Moses Ascending | TRINIDAD | 0 | 1975 | 3 | 1923 | 0 | 0 | 0 | 0 | 0 | 0 | 0 | 0 | 0 | 0 | 0 | 0 | 0 | 0 | 0 | 0 |
| Selvon | Sam | The Lonely Londoners | TRINIDAD | 0 | 1956 | 3 | 1923 | 0 | 0 | 0 | 0 | 0 | 0 | 0 | 0 | 0 | 0 | 0 | 0 | 0 | 0 | 0 | 0 |
| Sembène | Ousmane | Xala | SENEGAL | 0 | 1973 | 5 | 1923 | 1 | 0 | 0 | 0 | 0 | 0 | 0 | 0 | 0 | 0 | 0 | 0 | 0 | 0 | 0 | 0 |
| Sendker | Jan-Philipp | The art of hearing heartbeats | GERMANY | 0 | 2002 | 1 | 1960 | 1 | 0 | 0 | 0 | 0 | 0 | 0 | 0 | 0 | 0 | 0 | 0 | 0 | 0 | 0 | 0 |
| Sendker | Jan-Philipp | Whispering shadows | GERMANY | 0 | 2007 | 1 | 1960 | 1 | 0 | 0 | 0 | 0 | 0 | 0 | 0 | 0 | 0 | 0 | 0 | 0 | 0 | 0 | 0 |
| Serpell | Namwali | The Old Drift | ZAMBIA | 1 | 2019 | 5 | 1980 | 0 | 0 | 0 | 0 | 0 | 0 | 0 | 0 | 0 | 0 | 0 | 0 | 0 | 0 | 1 | 1 |
| Serrano | Marcela | Ten women | CHILE | 1 | 2011 | 3 | 1951 | 1 | 0 | 0 | 0 | 0 | 0 | 0 | 0 | 0 | 0 | 0 | 0 | 0 | 0 | 0 | 0 |
| Seth | Vikram | A suitable boy | INDIA | 0 | 1993 | 4 | 1952 | 0 | 0 | 0 | 0 | 0 | 0 | 0 | 0 | 0 | 0 | 0 | 0 | 0 | 0 | 1 | 0 |
| Seth | Vikram | The golden gate | INDIA | 0 | 1986 | 4 | 1952 | 0 | 0 | 0 | 0 | 0 | 0 | 0 | 0 | 0 | 0 | 0 | 0 | 0 | 0 | 0 | 0 |
| Sewell | Anna | Black Beauty | UK | 1 | 1877 | 1 | 1820 | 0 | 0 | 0 | 0 | 0 | 0 | 0 | 0 | 0 | 0 | 0 | 0 | 0 | 0 | 1 | 0 |
| Shaara | Michael | The Killer Angels | USA | 0 | 1974 | 2 | 1928 | 0 | 0 | 0 | 0 | 1 | 0 | 0 | 0 | 0 | 0 | 0 | 0 | 0 | 0 | 0 | 0 |
| Shacochis | Bob | The Woman Who Lost Her Soul | USA | 0 | 2013 | 2 | 1951 | 0 | 0 | 0 | 0 | 0 | 0 | 0 | 0 | 0 | 0 | 0 | 0 | 0 | 0 | 1 | 0 |
| Shafak | Elif | The bastard of Istanbul | TURKEY | 1 | 2006 | 4 | 1971 | 0 | 0 | 0 | 0 | 0 | 0 | 0 | 0 | 0 | 0 | 0 | 0 | 0 | 0 | 0 | 0 |
| Shafak | Elif | The flea palace | TURKEY | 1 | 2002 | 4 | 1971 | 1 | 0 | 0 | 0 | 0 | 0 | 0 | 0 | 0 | 0 | 0 | 0 | 0 | 0 | 0 | 0 |
| Shafak | Elif | The Forty Rules of Love | TURKEY | 1 | 2009 | 4 | 1971 | 0 | 0 | 0 | 0 | 0 | 0 | 0 | 0 | 0 | 0 | 0 | 0 | 0 | 0 | 0 | 0 |
| Shah | Bina | A Season for Martyrs | PAKISTAN | 1 | 2014 | 4 | 1972 | 0 | 0 | 0 | 0 | 0 | 0 | 0 | 0 | 0 | 0 | 0 | 0 | 0 | 0 | 0 | 0 |
| Shah | Bina | Before She Sleeps | PAKISTAN | 1 | 2018 | 4 | 1972 | 0 | 0 | 0 | 0 | 0 | 0 | 0 | 0 | 0 | 0 | 0 | 0 | 0 | 0 | 0 | 0 |
| Shalamov | Varlam | Kolyma stories | RUSSIA | 0 | 1973 | 1 | 1907 | 1 | 0 | 0 | 0 | 0 | 0 | 0 | 0 | 0 | 0 | 0 | 0 | 0 | 0 | 0 | 0 |
| Shalev | Meir | The loves of Judith | ISRAEL | 0 | 1994 | 4 | 1948 | 1 | 0 | 0 | 0 | 0 | 0 | 0 | 0 | 0 | 0 | 0 | 0 | 0 | 0 | 0 | 0 |
| Shalev | Zeruya | The remains of love | ISRAEL | 1 | 2014 | 4 | 1959 | 1 | 0 | 0 | 0 | 0 | 0 | 0 | 0 | 0 | 0 | 0 | 0 | 0 | 0 | 0 | 0 |
| Shamsie | Kamila | Home fire | PAKISTAN | 1 | 2017 | 4 | 1973 | 0 | 0 | 0 | 0 | 0 | 0 | 0 | 0 | 0 | 0 | 1 | 0 | 1 | 0 | 0 | 0 |
| Shamsie | Kamila | Burnt shadows | PAKISTAN | 1 | 2009 | 4 | 1973 | 0 | 0 | 0 | 0 | 0 | 0 | 0 | 0 | 0 | 0 | 0 | 0 | 0 | 0 | 0 | 0 |
| Shanbhag | Vivek | Ghachar Ghochar | INDIA | 0 | 2015 | 4 | 1962 | 1 | 0 | 0 | 0 | 0 | 0 | 0 | 0 | 0 | 0 | 0 | 0 | 0 | 1 | 0 | 0 |
| Sharma | Akhil | Family life | INDIA | 0 | 2014 | 4 | 1971 | 0 | 0 | 0 | 0 | 0 | 0 | 0 | 0 | 0 | 0 | 0 | 1 | 0 | 0 | 0 | 0 |
| Sharp | Margery | Britannia Mews | UK | 1 | 1946 | 1 | 1905 | 0 | 0 | 0 | 0 | 0 | 0 | 0 | 0 | 0 | 0 | 0 | 0 | 0 | 0 | 0 | 0 |
| She | Lao | Rickshaw boy | CHINA | 0 | 1937 | 4 | 1899 | 1 | 0 | 0 | 0 | 0 | 0 | 0 | 0 | 0 | 0 | 0 | 0 | 0 | 0 | 0 | 0 |
| Sheldon | Sidney | The Other Side of Midnight | USA | 0 | 1973 | 2 | 1917 | 0 | 0 | 0 | 0 | 0 | 0 | 0 | 0 | 0 | 0 | 0 | 0 | 0 | 0 | 0 | 0 |
| Sheldon | Sidney | The Naked Face | USA | 0 | 1969 | 2 | 1917 | 0 | 0 | 0 | 0 | 0 | 0 | 0 | 0 | 0 | 0 | 0 | 0 | 0 | 0 | 0 | 0 |
| Shields | Carol | The Stone Diaries | USA | 1 | 1993 | 2 | 1935 | 0 | 0 | 0 | 0 | 1 | 0 | 0 | 0 | 0 | 0 | 0 | 0 | 0 | 0 | 0 | 0 |
| Shimada | Soji | The Tokyo Zodiac Murders | JAPAN | 0 | 1981 | 4 | 1948 | 1 | 0 | 0 | 0 | 0 | 0 | 0 | 0 | 0 | 0 | 0 | 0 | 0 | 0 | 0 | 0 |
| Shimada | Soji | Murder in the Crooked House | JAPAN | 0 | 1982 | 4 | 1948 | 1 | 0 | 0 | 0 | 0 | 0 | 0 | 0 | 0 | 0 | 0 | 0 | 0 | 0 | 0 | 0 |
| Shin | Kyung-sook | Please look after mom | SOUTH KOREA | 1 | 2008 | 4 | 1963 | 1 | 0 | 0 | 0 | 0 | 0 | 0 | 0 | 0 | 0 | 0 | 0 | 0 | 0 | 0 | 0 |
| Shin | Kyung-sook | The court dancer | SOUTH KOREA | 1 | 2018 | 4 | 1963 | 1 | 0 | 0 | 0 | 0 | 0 | 0 | 0 | 0 | 0 | 0 | 0 | 0 | 0 | 0 | 0 |
| Shin | Kyung-sook | I'll Be Right There | SOUTH KOREA | 1 | 2014 | 4 | 1963 | 1 | 0 | 0 | 0 | 0 | 0 | 0 | 0 | 0 | 0 | 0 | 0 | 0 | 0 | 0 | 0 |
| Shin | Kyung-sook | The Girl Who Wrote Loneliness | SOUTH KOREA | 1 | 2015 | 4 | 1963 | 1 | 0 | 0 | 0 | 0 | 0 | 0 | 0 | 0 | 0 | 0 | 0 | 0 | 0 | 0 | 0 |
| Sholokhov | Mikhail | And quiet flows the Don | RUSSIA | 0 | 1928 | 1 | 1905 | 1 | 1 | 0 | 0 | 0 | 0 | 0 | 0 | 0 | 0 | 0 | 0 | 0 | 0 | 0 | 0 |
| Shoneyin | Lola | The Secret Lives of Baba Segi's wives | NIGERIA | 1 | 2010 | 5 | 1974 | 0 | 0 | 0 | 0 | 0 | 0 | 0 | 0 | 0 | 0 | 0 | 0 | 0 | 0 | 0 | 0 |
| Shreve | Anita | The Pilot's Wife | USA | 1 | 1998 | 2 | 1946 | 0 | 0 | 0 | 0 | 0 | 0 | 0 | 0 | 0 | 0 | 0 | 0 | 0 | 0 | 0 | 0 |
| Sidhwa | Bapsi | The pakistani bride | PAKISTAN | 1 | 1983 | 4 | 1936 | 0 | 0 | 0 | 0 | 0 | 0 | 0 | 0 | 0 | 0 | 0 | 0 | 0 | 0 | 0 | 0 |
| Sidhwa | Bapsi | Their Language of Love | PAKISTAN | 1 | 2013 | 4 | 1936 | 0 | 0 | 0 | 0 | 0 | 0 | 0 | 0 | 0 | 0 | 0 | 0 | 0 | 0 | 0 | 0 |
| Sidhwa | Bapsi | Ice Candy Man (Cracking India) | PAKISTAN | 1 | 1988 | 4 | 1936 | 0 | 0 | 0 | 0 | 0 | 0 | 0 | 0 | 0 | 0 | 0 | 0 | 0 | 0 | 0 | 0 |
| Sidhwa | Bapsi | The Crow Eaters | PAKISTAN | 1 | 1978 | 4 | 1936 | 0 | 0 | 0 | 0 | 0 | 0 | 0 | 0 | 0 | 0 | 0 | 0 | 0 | 0 | 0 | 0 |
| Sienkiewicz | Henryk | Quo Vadis | POLAND | 0 | 1896 | 1 | 1846 | 1 | 1 | 0 | 0 | 0 | 0 | 0 | 0 | 0 | 0 | 0 | 0 | 0 | 0 | 0 | 0 |
| Sillanpää | Frans | People in the Summer Night | FINLAND | 0 | 1934 | 1 | 1888 | 1 | 1 | 0 | 0 | 0 | 0 | 0 | 0 | 0 | 0 | 0 | 0 | 0 | 0 | 0 | 0 |

| **SURNAME** | **NAME** | **TITLE** | **NATIONALITY** | | **GENDER** | **PUB_YEAR** | **CONTINENT** | **DATE OF BIRTH** | **TRANSLATED** | **NOBEL** | **NEUSTADT** | **BOOKER** | **PULITZER** | **NEBULA** | **CAINE** | **FRANZ KAFKA** | **NBA** | **BBA** | **WOMEN'S PRIZE FOR FICTION** | **NEW YORK TIMES** | **THE GUARDIAN** | **THE NEW YORKER** | **BBC** | **ATLANTIC** |
| --- | --- | --- | --- | --- | --- | --- | --- | --- | --- | --- | --- | --- | --- | --- | --- | --- | --- | --- | --- | --- | --- | --- | --- | --- |
| Simenon | Georges | The man who watched the trains go by | | BELGIUM | 0 | 1938 | 1 | 1903 | 1 | 0 | 0 | 0 | 0 | 0 | 0 | 0 | 0 | 0 | 0 | 0 | 0 | 0 | 0 | 0 |
| Simenon | Georges | Three bedrooms in Manhattan | | BELGIUM | 0 | 1946 | 1 | 1903 | 1 | 0 | 0 | 0 | 0 | 0 | 0 | 0 | 0 | 0 | 0 | 0 | 0 | 0 | 0 | 0 |
| Simenon | Georges | The widow | | BELGIUM | 0 | 1942 | 1 | 1903 | 1 | 0 | 0 | 0 | 0 | 0 | 0 | 0 | 0 | 0 | 0 | 0 | 0 | 0 | 0 | 0 |
| Simon | Claude | The trolley | | FRANCE | 0 | 2001 | 1 | 1913 | 1 | 1 | 0 | 0 | 0 | 0 | 0 | 0 | 0 | 0 | 0 | 0 | 0 | 0 | 0 | 0 |
| Simsion | Graeme | The Rosie project | | NEW ZEALAND | 0 | 2013 | 6 | 1956 | 0 | 0 | 0 | 0 | 0 | 0 | 0 | 0 | 0 | 0 | 0 | 0 | 0 | 0 | 0 | 0 |
| Sinclair | Upton | Dragon's Teeth | | USA | 0 | 1942 | 2 | 1878 | 0 | 0 | 0 | 0 | 1 | 0 | 0 | 0 | 0 | 0 | 0 | 0 | 0 | 0 | 0 | 0 |
| Singer | Isaac | The Spinoza of Market Street | | POLAND | 0 | 1961 | 1 | 1902 | 1 | 1 | 0 | 0 | 0 | 0 | 0 | 0 | 0 | 0 | 0 | 0 | 0 | 0 | 0 | 0 |
| Singer | Isaac | Meshugah | | POLAND | 0 | 1994 | 1 | 1902 | 1 | 1 | 0 | 0 | 0 | 0 | 0 | 0 | 0 | 0 | 0 | 0 | 0 | 0 | 0 | 0 |
| Singer | Isaac | In my father's court | | POLAND | 0 | 1979 | 1 | 1902 | 1 | 1 | 0 | 0 | 0 | 0 | 0 | 0 | 0 | 0 | 0 | 0 | 0 | 0 | 0 | 0 |
| Singh | Khushwant | Train to Pakistan | | INDIA | 0 | 1956 | 4 | 1915 | 0 | 0 | 0 | 0 | 0 | 0 | 0 | 0 | 0 | 0 | 0 | 0 | 0 | 0 | 0 | 0 |
| Sinha | Indra | Animal's People | | INDIA | 0 | 2007 | 4 | 1950 | 0 | 0 | 0 | 0 | 0 | 0 | 0 | 0 | 0 | 0 | 0 | 0 | 0 | 0 | 0 | 0 |
| Sionil | Jose F | Don Vicente | | PHILIPPINES | 0 | 1980 | 4 | 1924 | 0 | 0 | 0 | 0 | 0 | 0 | 0 | 0 | 0 | 0 | 0 | 0 | 0 | 0 | 0 | 0 |
| Sionil | Jose F | Three Filipino Women | | PHILIPPINES | 0 | 1992 | 4 | 1924 | 0 | 0 | 0 | 0 | 0 | 0 | 0 | 0 | 0 | 0 | 0 | 0 | 0 | 0 | 0 | 0 |
| Sionil | Jose F | Sins | | PHILIPPINES | 0 | 1994 | 4 | 1924 | 0 | 0 | 0 | 0 | 0 | 0 | 0 | 0 | 0 | 0 | 0 | 0 | 0 | 0 | 0 | 0 |
| Sirees | Nihad | States of Passion | | SYRIA | 0 | 2018 | 4 | 1950 | 1 | 0 | 0 | 0 | 0 | 0 | 0 | 0 | 0 | 0 | 0 | 0 | 0 | 0 | 0 | 0 |
| Sirees | Nihad | The Silence and the Roar | | SYRIA | 0 | 2013 | 4 | 1950 | 1 | 0 | 0 | 0 | 0 | 0 | 0 | 0 | 0 | 0 | 0 | 0 | 0 | 0 | 0 | 0 |
| Sirleaf | Ellen Johnson | This Child Will Be Great | | LIBERIA | 1 | 2010 | 5 | 1938 | 0 | 0 | 0 | 0 | 0 | 0 | 0 | 0 | 0 | 0 | 0 | 0 | 0 | 0 | 0 | 0 |
| Skloot | Rebecca | The immortal life of Henrietta Lacks | | USA | 1 | 2010 | 2 | 1972 | 0 | 0 | 0 | 0 | 0 | 0 | 0 | 0 | 0 | 0 | 0 | 0 | 0 | 0 | 0 | 0 |
| Škvorecký | Josef | The Cowards | | CZECH REP. | 0 | 1958 | 1 | 1924 | 1 | 0 | 1 | 0 | 0 | 0 | 0 | 0 | 0 | 0 | 0 | 0 | 0 | 0 | 0 | 0 |
| Slimani | Leïla | Lullaby | | MOROCCO | 1 | 2016 | 5 | 1981 | 1 | 0 | 0 | 0 | 0 | 0 | 0 | 0 | 0 | 1 | 0 | 0 | 0 | 0 | 0 | 0 |
| Slimani | Leïla | Adele | | MOROCCO | 1 | 2014 | 5 | 1981 | 1 | 0 | 0 | 0 | 0 | 0 | 0 | 0 | 0 | 0 | 0 | 0 | 0 | 0 | 0 | 0 |
| Smiley | Jane | A Thousand Acres | | USA | 1 | 1992 | 2 | 1949 | 0 | 0 | 0 | 0 | 1 | 0 | 0 | 0 | 0 | 0 | 0 | 0 | 0 | 0 | 0 | 0 |
| Smith | Martin Cruz | Gorky park | | USA | 0 | 1981 | 2 | 1942 | 0 | 0 | 0 | 0 | 0 | 0 | 0 | 0 | 0 | 0 | 0 | 0 | 0 | 0 | 0 | 0 |
| Smith | Wilbur | When the lion feeds | | UK | 0 | 1964 | 1 | 1933 | 0 | 0 | 0 | 0 | 0 | 0 | 0 | 0 | 0 | 1 | 0 | 0 | 0 | 0 | 0 | 0 |
| Smith | Wilbur | The burning shore | | UK | 0 | 1985 | 1 | 1933 | 0 | 0 | 0 | 0 | 0 | 0 | 0 | 0 | 0 | 1 | 0 | 0 | 0 | 0 | 0 | 0 |
| Smith | Zadie | NW | | UK | 1 | 2012 | 1 | 1975 | 0 | 0 | 0 | 0 | 0 | 0 | 0 | 0 | 0 | 0 | 0 | 1 | 0 | 0 | 0 | 0 |
| Smith | Zadie | Swing time | | UK | 1 | 2016 | 1 | 1975 | 0 | 0 | 0 | 0 | 0 | 0 | 0 | 0 | 0 | 0 | 0 | 0 | 1 | 0 | 0 | 1 |
| Smith | Ali | Hotel World | | UK | 1 | 2001 | 1 | 1962 | 0 | 0 | 0 | 0 | 0 | 0 | 0 | 0 | 0 | 0 | 0 | 0 | 0 | 0 | 0 | 0 |
| Solares | Martin | The Black Minutes | | MEXICO | 0 | 2006 | 3 | 1970 | 1 | 0 | 0 | 0 | 0 | 0 | 0 | 0 | 0 | 0 | 0 | 0 | 0 | 0 | 0 | 0 |
| Solares | Martin | Don't Send Flowers | | MEXICO | 0 | 2015 | 3 | 1970 | 1 | 0 | 0 | 0 | 0 | 0 | 0 | 0 | 0 | 0 | 0 | 0 | 0 | 0 | 0 | 0 |
| Solzhenitsyn | Aleksandr | The gulag archipelago | | RUSSIA | 0 | 1973 | 1 | 1918 | 1 | 1 | 0 | 0 | 0 | 0 | 0 | 0 | 0 | 0 | 0 | 0 | 0 | 0 | 0 | 0 |
| Somerset | William | The Painted Veil | | UK | 0 | 1925 | 1 | 1874 | 0 | 0 | 0 | 0 | 0 | 0 | 0 | 0 | 0 | 0 | 0 | 0 | 0 | 0 | 0 | 0 |
| Somerset | William | Theatre | | UK | 0 | 1937 | 1 | 1874 | 0 | 0 | 0 | 0 | 0 | 0 | 0 | 0 | 0 | 0 | 0 | 0 | 0 | 0 | 0 | 0 |
| Sönmez | Burhan | Istanbul Istanbul | | TURKEY | 0 | 2015 | 4 | 1965 | 1 | 0 | 0 | 0 | 0 | 0 | 0 | 0 | 0 | 0 | 0 | 0 | 0 | 0 | 0 | 0 |
| Sontag | Susan | The Volcano Lover | | USA | 1 | 1992 | 2 | 1933 | 0 | 0 | 0 | 0 | 0 | 0 | 0 | 0 | 0 | 0 | 0 | 0 | 0 | 0 | 0 | 0 |
| Sontag | Susan | In America | | USA | 1 | 1999 | 2 | 1933 | 0 | 0 | 0 | 0 | 0 | 0 | 0 | 0 | 1 | 0 | 0 | 0 | 0 | 0 | 0 | 0 |
| Sōseki | Natsume | Kokoro | | JAPAN | 0 | 1914 | 4 | 1867 | 1 | 0 | 0 | 0 | 0 | 0 | 0 | 0 | 0 | 0 | 0 | 0 | 0 | 0 | 0 | 0 |
| Sōseki | Natsume | And then | | JAPAN | 0 | 1909 | 4 | 1867 | 1 | 0 | 0 | 0 | 0 | 0 | 0 | 0 | 0 | 0 | 0 | 0 | 0 | 0 | 0 | 0 |
| Sōseki | Natsume | I am a cat | | JAPAN | 0 | 1905 | 4 | 1867 | 1 | 0 | 0 | 0 | 0 | 0 | 0 | 0 | 0 | 0 | 0 | 0 | 0 | 0 | 0 | 0 |
| Sōseki | Natsume | Sanshiro | | JAPAN | 0 | 1908 | 4 | 1867 | 1 | 0 | 0 | 0 | 0 | 0 | 0 | 0 | 0 | 0 | 0 | 0 | 0 | 0 | 0 | 0 |
| Souad | Souad | Burned alive | | PALESTINE | 1 | 2003 | 4 | 1958 | 1 | 0 | 0 | 0 | 0 | 0 | 0 | 0 | 0 | 0 | 0 | 0 | 0 | 0 | 0 | 0 |
| Soueif | Ahdaf | I Think of You: Stories | | EGYPT | 1 | 2007 | 5 | 1950 | 0 | 0 | 0 | 0 | 0 | 0 | 0 | 0 | 0 | 0 | 0 | 0 | 0 | 0 | 0 | 0 |
| Soueif | Ahdaf | The Map of Love | | EGYPT | 1 | 1999 | 5 | 1950 | 0 | 0 | 0 | 0 | 0 | 0 | 0 | 0 | 0 | 0 | 0 | 0 | 0 | 0 | 0 | 0 |
| Soyinka | Wole | You Must Set Forth at Dawn | | NIGERIA | 0 | 2007 | 5 | 1934 | 0 | 1 | 0 | 0 | 0 | 0 | 0 | 0 | 0 | 0 | 0 | 0 | 0 | 0 | 0 | 0 |
| Spark | Muriel | The public image | | UK | 1 | 1968 | 1 | 1918 | 0 | 0 | 0 | 0 | 0 | 0 | 0 | 0 | 0 | 0 | 0 | 0 | 0 | 0 | 0 | 0 |
| Sparks | Nicholas | The notebook | | USA | 0 | 1996 | 2 | 1965 | 0 | 0 | 0 | 0 | 0 | 0 | 0 | 0 | 0 | 0 | 0 | 0 | 0 | 0 | 0 | 0 |
| Sparks | Nicholas | Message in a bottle | | USA | 0 | 1998 | 2 | 1965 | 0 | 0 | 0 | 0 | 0 | 0 | 0 | 0 | 0 | 0 | 0 | 0 | 0 | 0 | 0 | 0 |
| Sparks | Nicholas | A walk to remember | | USA | 0 | 1999 | 2 | 1965 | 0 | 0 | 0 | 0 | 0 | 0 | 0 | 0 | 0 | 0 | 0 | 0 | 0 | 0 | 0 | 0 |
| Sparks | Nicholas | A bend in the road | | USA | 0 | 2001 | 2 | 1965 | 0 | 0 | 0 | 0 | 0 | 0 | 0 | 0 | 0 | 0 | 0 | 0 | 0 | 0 | 0 | 0 |
| Sparks | Nicholas | Nights in Rodanthe | | USA | 0 | 2002 | 2 | 1965 | 0 | 0 | 0 | 0 | 0 | 0 | 0 | 0 | 0 | 0 | 0 | 0 | 0 | 0 | 0 | 0 |
| Sparks | Nicholas | The rescue | | USA | 0 | 2000 | 2 | 1965 | 0 | 0 | 0 | 0 | 0 | 0 | 0 | 0 | 0 | 0 | 0 | 0 | 0 | 0 | 0 | 0 |
| Spyri | Johanna | Heidi | | SWITZERLAND | 1 | 1880 | 1 | 1827 | 1 | 0 | 0 | 0 | 0 | 0 | 0 | 0 | 0 | 0 | 0 | 0 | 0 | 0 | 0 | 0 |
| St John | Madeleine | Ladies in black | | AUSTRALIA | 1 | 1993 | 6 | 1941 | 0 | 0 | 0 | 0 | 0 | 0 | 0 | 0 | 0 | 0 | 0 | 0 | 0 | 0 | 0 | 0 |
| St John Mandel | Emily | Station eleven | | CANADA | 1 | 2014 | 2 | 1979 | 0 | 0 | 0 | 0 | 0 | 0 | 0 | 0 | 0 | 0 | 0 | 0 | 0 | 0 | 0 | 1 |
| St Omer | Garth | Prisnms | | S. LUCIA | 0 | 2015 | 3 | 1931 | 0 | 0 | 0 | 0 | 0 | 0 | 0 | 0 | 0 | 0 | 0 | 0 | 0 | 0 | 0 | 0 |
| Stafford | Jean | Collected Stories | | USA | 1 | 1969 | 2 | 1915 | 0 | 0 | 0 | 0 | 1 | 0 | 0 | 0 | 0 | 0 | 0 | 0 | 0 | 0 | 0 | 0 |
| Stead | Christina | The Man Who Loved Children | | AUSTRALIA | 1 | 1940 | 6 | 1902 | 0 | 0 | 0 | 0 | 0 | 0 | 0 | 0 | 0 | 0 | 0 | 0 | 0 | 0 | 0 | 0 |
| Stefansson | Jón Kalman | Heaven and hell | | ICELAND | 0 | 2007 | 1 | 1963 | 1 | 0 | 0 | 0 | 0 | 0 | 0 | 0 | 0 | 0 | 0 | 0 | 0 | 0 | 0 | 0 |
| Stegner | Wallace | Angle Of Repose | | USA | 0 | 1971 | 2 | 1909 | 0 | 0 | 0 | 0 | 1 | 0 | 0 | 0 | 0 | 0 | 0 | 0 | 0 | 0 | 0 | 0 |
| Stein | Gertrude | Three lives | | USA | 1 | 1909 | 2 | 1874 | 0 | 0 | 0 | 0 | 0 | 0 | 0 | 0 | 0 | 0 | 0 | 0 | 0 | 0 | 0 | 0 |
| Stein | Gertrude | Everybody's Autobiography | | USA | 1 | 1937 | 2 | 1874 | 0 | 0 | 0 | 0 | 0 | 0 | 0 | 0 | 0 | 0 | 0 | 0 | 0 | 0 | 0 | 0 |
| Stein | Gertrude | The Autobiography of Alice B. Toklas | | USA | 1 | 1933 | 2 | 1874 | 0 | 0 | 0 | 0 | 0 | 0 | 0 | 0 | 0 | 0 | 0 | 0 | 0 | 0 | 0 | 0 |
| Stein | Gertrude | The Making of Americans | | USA | 1 | 1925 | 2 | 1874 | 0 | 0 | 0 | 0 | 0 | 0 | 0 | 0 | 0 | 0 | 0 | 0 | 0 | 0 | 0 | 0 |
| Steinbeck | John | The grapes of wrath | | USA | 0 | 1939 | 2 | 1902 | 0 | 1 | 0 | 0 | 1 | 0 | 0 | 0 | 1 | 0 | 0 | 0 | 0 | 0 | 1 | 0 |
| Steinbeck | John | Of mice and men | | USA | 0 | 1937 | 2 | 1902 | 0 | 1 | 0 | 0 | 0 | 0 | 0 | 0 | 0 | 0 | 0 | 0 | 0 | 0 | 1 | 0 |
| Steinbeck | John | In dubious battle | | USA | 0 | 1936 | 2 | 1902 | 0 | 1 | 0 | 0 | 0 | 0 | 0 | 0 | 0 | 0 | 0 | 0 | 0 | 0 | 0 | 0 |
| Steinbeck | John | East of Eden | | USA | 0 | 1952 | 2 | 1902 | 0 | 1 | 0 | 0 | 0 | 0 | 0 | 0 | 0 | 0 | 0 | 0 | 0 | 0 | 0 | 0 |
| Stendhal | Marie-Henri | The red and the black | | FRANCE | 0 | 1831 | 1 | 1783 | 1 | 0 | 0 | 0 | 0 | 0 | 0 | 0 | 0 | 0 | 0 | 0 | 0 | 0 | 0 | 0 |
| Stendhal | Marie-Henri | The Charterhouse of Parma | | FRANCE | 0 | 1839 | 1 | 1783 | 1 | 0 | 0 | 0 | 0 | 0 | 0 | 0 | 0 | 0 | 0 | 0 | 0 | 0 | 0 | 0 |
| Sterne | Laurence | ntimental Journey Through France and | | IRELAND | 0 | 1768 | 1 | 1713 | 0 | 0 | 0 | 0 | 0 | 0 | 0 | 0 | 0 | 0 | 0 | 0 | 0 | 0 | 0 | 0 |
| Sterne | Laurence | A Political Romance | | IRELAND | 0 | 1759 | 1 | 1713 | 0 | 0 | 0 | 0 | 0 | 0 | 0 | 0 | 0 | 0 | 0 | 0 | 0 | 0 | 0 | 0 |
| Stevenson | Robert Louis | e strange case of Dr. Jekyll and Mr. Hy | | UK | 0 | 1886 | 1 | 1850 | 0 | 0 | 0 | 0 | 0 | 0 | 0 | 0 | 0 | 0 | 0 | 0 | 0 | 0 | 0 | 0 |
| Stevenson | Robert Louis | Treasure island | | UK | 0 | 1883 | 1 | 1850 | 0 | 0 | 0 | 0 | 0 | 0 | 0 | 0 | 0 | 0 | 0 | 0 | 0 | 0 | 1 | 0 |
| Stevenson | Robert Louis | The Wrong Box | | UK | 0 | 1889 | 1 | 1850 | 0 | 0 | 0 | 0 | 0 | 0 | 0 | 0 | 0 | 0 | 0 | 0 | 0 | 0 | 0 | 0 |
| Stevenson | Robert Louis | Weir of Hermiston | | UK | 0 | 1896 | 1 | 1850 | 0 | 0 | 0 | 0 | 0 | 0 | 0 | 0 | 0 | 0 | 0 | 0 | 0 | 0 | 0 | 0 |
| Stevenson | Robert Louis | he Black Arrow: A Tale of the Two Rose | | UK | 0 | 1888 | 1 | 1850 | 0 | 0 | 0 | 0 | 0 | 0 | 0 | 0 | 0 | 0 | 0 | 0 | 0 | 0 | 0 | 0 |
| Stevenson | Robert Louis | The Master of Ballantrae | | UK | 0 | 1889 | 1 | 1850 | 0 | 0 | 0 | 0 | 0 | 0 | 0 | 0 | 0 | 0 | 0 | 0 | 0 | 0 | 0 | 0 |
| Stevenson | Robert Louis | The Wrecker | | UK | 0 | 1892 | 1 | 1850 | 0 | 0 | 0 | 0 | 0 | 0 | 0 | 0 | 0 | 0 | 0 | 0 | 0 | 0 | 0 | 0 |
| Stockett | Kathryn | The help | | USA | 1 | 2009 | 2 | 1969 | 0 | 0 | 0 | 0 | 0 | 0 | 0 | 0 | 0 | 0 | 0 | 0 | 0 | 0 | 0 | 0 |
| Stoker | Bram | Dracula | | IRELAND | 0 | 1897 | 1 | 1847 | 0 | 0 | 0 | 0 | 0 | 0 | 0 | 0 | 0 | 0 | 0 | 0 | 0 | 0 | 0 | 0 |
| Stone | Irving | Lust for Life | | USA | 0 | 1934 | 2 | 1903 | 0 | 0 | 0 | 0 | 0 | 0 | 0 | 0 | 0 | 0 | 0 | 0 | 0 | 0 | 0 | 0 |
| Storey | David | Pasmore | | UK | 0 | 1972 | 1 | 1933 | 0 | 0 | 0 | 0 | 0 | 0 | 0 | 0 | 0 | 0 | 0 | 0 | 0 | 0 | 0 | 0 |
| Storey | David | Saville | | UK | 0 | 1976 | 1 | 1933 | 0 | 0 | 0 | 1 | 0 | 0 | 0 | 0 | 0 | 0 | 0 | 0 | 0 | 0 | 0 | 0 |
| Stout | Rex | Fer-de-Lance | | USA | 0 | 1934 | 2 | 1886 | 0 | 0 | 0 | 0 | 0 | 0 | 0 | 0 | 0 | 0 | 0 | 0 | 0 | 0 | 0 | 0 |
| Stout | Rex | The League of Frightened Men | | USA | 0 | 1935 | 2 | 1886 | 0 | 0 | 0 | 0 | 0 | 0 | 0 | 0 | 0 | 0 | 0 | 0 | 0 | 0 | 0 | 0 |
| Stowe | Harriet | Uncle Tom's cabin | | USA | 1 | 1852 | 2 | 1811 | 0 | 0 | 0 | 0 | 0 | 0 | 0 | 0 | 0 | 0 | 0 | 0 | 0 | 0 | 0 | 0 |
| Stowe | Harriet | Agnes of Sorrento | | USA | 1 | 1862 | 2 | 1811 | 0 | 0 | 0 | 0 | 0 | 0 | 0 | 0 | 0 | 0 | 0 | 0 | 0 | 0 | 0 | 0 |

| **SURNAME** | | **NAME** | **TITLE** | **NATIONALITY** | | **GENDER** | **PUB_YEAR** | **CONTINENT** | **DATE OF BIRTH** | **TRANSLATED** | **NOBEL** | **NEUSTADT** | **BOOKER** | **PULITZER** | **NEBULA** | **CAINE** | **FRANZ KAFKA** | **NBA** | **BBA** | **WOMEN'S PRIZE FOR FICTION** | **NEW YORK TIMES** | **THE GUARDIAN** | **THE NEW YORKER** | **BBC** | **ATLANTIC** |
| --- | --- | --- | --- | --- | --- | --- | --- | --- | --- | --- | --- | --- | --- | --- | --- | --- | --- | --- | --- | --- | --- | --- | --- | --- | --- |
| Stowe Stowe Stowe Stowe  Stowe | Harriet Harriet Harriet Harriet  Harriet | | Lady Byron Vindicated Palmetto Leaves  Pink and White Tyranny The Minister's Wooing  The Pearl of Orr's Island | | USA USA USA USA  USA | 1  1  1  1  1 | 1870  1873  1871  1859  1862 | 2  2  2  2  2 | 1811  1811  1811  1811  1811 | 0  0  0  0  0 | 0  0  0  0  0 | 0  0  0  0  0 | 0  0  0  0  0 | 0  0  0  0  0 | 0  0  0  0  0 | 0  0  0  0  0 | 0  0  0  0  0 | 0  0  0  0  0 | 0  0  0  0  0 | 0  0  0  0  0 | 0  0  0  0  0 | 0  0  0  0  0 | 0  0  0  0  0 | 0  0  0  0  0 | 0  0  0  0  0 |
| Stribling | homas Sigismu | | The Store | | USA | 0 | 1932 | 2 | 1881 | 0 | 0 | 0 | 0 | 1 | 0 | 0 | 0 | 0 | 0 | 0 | 0 | 0 | 0 | 0 | 0 |
| Stroud | Carsten | | Niceville | | CANADA | 0 | 2012 | 2 | 1946 | 0 | 0 | 0 | 0 | 0 | 0 | 0 | 0 | 0 | 0 | 0 | 0 | 0 | 0 | 0 | 0 |
| Strout  Strout | Elizabeth  Elizabeth | | Olive Kitteridge  The Burgess boys | | USA  USA | 1  1 | 2008  2013 | 2  2 | 1956  1956 | 0  0 | 0  0 | 0  0 | 0  0 | 1  0 | 0  0 | 0  0 | 0  0 | 0  0 | 0  0 | 0  0 | 0  0 | 0  0 | 0  0 | 0  0 | 0  0 |
| Stuart | Douglas | | Shuggie Bain | | UK | 0 | 2020 | 1 | 1976 | 0 | 0 | 0 | 1 | 0 | 0 | 0 | 0 | 0 | 1 | 0 | 0 | 1 | 0 | 1 | 0 |
| Sturgeon | Theodore | | The dreaming jewels | | USA | 0 | 1950 | 2 | 1918 | 0 | 0 | 0 | 0 | 0 | 0 | 0 | 0 | 0 | 0 | 0 | 0 | 0 | 0 | 0 | 0 |
| Styron  Styron | William  William | | Sophie’s Choice  The Confessions of Nat Turner | | USA  USA | 0  0 | 1979  1967 | 2  2 | 1925  1925 | 0  0 | 0  0 | 0  0 | 0  0 | 0  1 | 0  0 | 0  0 | 0  0 | 0  0 | 0  0 | 0  0 | 0  0 | 0  0 | 0  0 | 0  0 | 0  0 |
| Sudbanthad | Pitchaya | | Bangkok Wakes to Rain | | THAILAND | 0 | 2019 | 4 | ? | 0 | 0 | 0 | 0 | 0 | 0 | 0 | 0 | 0 | 0 | 0 | 0 | 0 | 0 | 0 | 0 |
| Sukegawa | Durian | | Sweet bean paste | | JAPAN | 0 | 2015 | 4 | 1962 | 1 | 0 | 0 | 0 | 0 | 0 | 0 | 0 | 0 | 0 | 0 | 0 | 0 | 0 | 0 | 0 |
| Susann | Jacqueline | | Valley of the Dolls | | USA | 1 | 1966 | 2 | 1918 | 0 | 0 | 0 | 0 | 0 | 0 | 0 | 0 | 0 | 0 | 0 | 0 | 0 | 0 | 0 | 0 |
| Süskind | Patrick | | Perfume | | GERMANY | 0 | 1985 | 1 | 1949 | 1 | 0 | 0 | 0 | 0 | 0 | 0 | 0 | 0 | 0 | 0 | 0 | 0 | 0 | 1 | 0 |
| Suzuki  Suzuki | Kōji  Kōji | | Ring  Loop | | JAPAN  JAPAN | 0  0 | 1991  1998 | 4  4 | 1957  1957 | 1  1 | 0  0 | 0  0 | 0  0 | 0  0 | 0  0 | 0  0 | 0  0 | 0  0 | 0  0 | 0  0 | 0  0 | 0  0 | 0  0 | 0  0 | 0  0 |
| Svevo  Svevo | Italo  Italo | | Zeno's conscience  As a Man Grows Older | | ITALY  ITALY | 0  0 | 1923  1898 | 1  1 | 1861  1861 | 1  1 | 0  0 | 0  0 | 0  0 | 0  0 | 0  0 | 0  0 | 0  0 | 0  0 | 0  0 | 0  0 | 0  0 | 0  0 | 0  0 | 0  0 | 0  0 |
| Swarup  Swarup | Vikas  Vikas | | Q&A  The Accidental Apprentice | | INDIA  INDIA | 0  0 | 2005  2013 | 4  4 | 1963  1963 | 0  0 | 0  0 | 0  0 | 0  0 | 0  0 | 0  0 | 0  0 | 0  0 | 0  0 | 0  0 | 0  0 | 0  0 | 0  0 | 0  0 | 0  0 | 0  0 |
| Swift | Jonathan | | Gulliver's travels | | IRELAND | 0 | 1726 | 1 | 1667 | 0 | 0 | 0 | 0 | 0 | 0 | 0 | 0 | 0 | 0 | 0 | 0 | 0 | 0 | 0 | 0 |
| Swift | Graham | | Last Orders | | UK | 0 | 1996 | 1 | 1949 | 0 | 0 | 0 | 1 | 0 | 0 | 0 | 0 | 0 | 0 | 0 | 0 | 0 | 0 | 0 | 0 |
| Syjuco | Miguel | | Ilustrado | | PHILIPPINES | 0 | 2008 | 4 | 1976 | 0 | 0 | 0 | 0 | 0 | 0 | 0 | 0 | 0 | 0 | 0 | 0 | 0 | 0 | 0 | 0 |
| Szabó | Magda | | The door | | HUNGARY | 1 | 1987 | 1 | 1917 | 1 | 0 | 0 | 0 | 0 | 0 | 0 | 0 | 0 | 0 | 0 | 1 | 0 | 0 | 0 | 0 |
| Szalay | David | | All That Man Is | | CANADA | 0 | 2016 | 2 | 1974 | 0 | 0 | 0 | 0 | 0 | 0 | 0 | 0 | 0 | 0 | 0 | 0 | 1 | 0 | 0 | 0 |
| Tabucchi  Tabucchi | Antonio  Antonio | | ittle misunderstandings of no importanc  Pereira Maintains | | ITALY  ITALY | 0  0 | 1985  1994 | 1  1 | 1943  1943 | 1  1 | 0  0 | 0  0 | 0  0 | 0  0 | 0  0 | 0  0 | 0  0 | 0  0 | 0  0 | 0  0 | 0  0 | 0  0 | 0  0 | 0  0 | 0  0 |
| Tademy | Lalita | | Cane River | | USA | 1 | 2001 | 2 | 1948 | 0 | 0 | 0 | 0 | 0 | 0 | 0 | 0 | 0 | 0 | 0 | 0 | 0 | 0 | 0 | 0 |
| Tagore | Rabindranath | | Home and the world | | INDIA | 0 | 1916 | 4 | 1861 | 1 | 1 | 0 | 0 | 0 | 0 | 0 | 0 | 0 | 0 | 0 | 0 | 0 | 0 | 0 | 0 |
| Taibo II | Paco Ignacio | | Some clouds | | MEXICO | 0 | 1985 | 3 | 1949 | 1 | 0 | 0 | 0 | 0 | 0 | 0 | 0 | 0 | 0 | 0 | 0 | 0 | 0 | 0 | 0 |
| Takagi | Akimitsu | | Tattoo murder case | | JAPAN | 0 | 1999 | 4 | 1920 | 1 | 0 | 0 | 0 | 0 | 0 | 0 | 0 | 0 | 0 | 0 | 0 | 0 | 0 | 0 | 0 |
| Takami | Koushun | | Battle royale | | JAPAN | 0 | 1999 | 4 | 1969 | 1 | 0 | 0 | 0 | 0 | 0 | 0 | 0 | 0 | 0 | 0 | 0 | 0 | 0 | 0 | 0 |
| Tanizaki Tanizaki  Tanizaki | Jun'ichirō Jun'ichirō  Jun'ichirō | | Quicksand The key  Some Prefer Nettles | | JAPAN JAPAN  JAPAN | 0  0  0 | 1931  1956  1928 | 4  4  4 | 1886  1886  1886 | 1  1  1 | 0  0  0 | 0  0  0 | 0  0  0 | 0  0  0 | 0  0  0 | 0  0  0 | 0  0  0 | 0  0  0 | 0  0  0 | 0  0  0 | 0  0  0 | 1  0  0 | 0  0  0 | 0  0  0 | 0  0  0 |
| Tanpınar  Tanpınar | Ahmet Hamdi  Ahmet Hamdi | | The Time Regulation Institute  A Mind at Peace | | TURKEY  TURKEY | 0  0 | 1954  1948 | 4  4 | 1901  1901 | 1  1 | 0  0 | 0  0 | 0  0 | 0  0 | 0  0 | 0  0 | 0  0 | 0  0 | 0  0 | 0  0 | 0  0 | 0  0 | 0  0 | 0  0 | 0  0 |
| Tarkington  Tarkington | Booth  Booth | | Alice Adams  The Magnificent Ambersons | | USA  USA | 0  0 | 1921  1918 | 2  2 | 1869  1869 | 0  0 | 0  0 | 0  0 | 0  0 | 1  1 | 0  0 | 0  0 | 0  0 | 0  0 | 0  0 | 0  0 | 0  0 | 0  0 | 0  0 | 0  0 | 0  0 |
| Tartt  Tartt | Donna  Donna | | The goldfinch  The little friend | | USA  USA | 1  1 | 2013  2002 | 2  2 | 1963  1963 | 0  0 | 0  0 | 0  0 | 0  0 | 1  0 | 0  0 | 0  0 | 0  0 | 0  0 | 0  0 | 0  0 | 1  0 | 1  0 | 0  0 | 0  0 | 1  0 |
| Tarttelin | Abigail | | Golden Boy | | UK | 1 | 2013 | 1 | 1987 | 0 | 0 | 0 | 0 | 0 | 0 | 0 | 0 | 0 | 0 | 0 | 0 | 0 | 0 | 0 | 0 |
| Tawada Tawada  Tawada | Yoko Yoko  Yoko | | Memoirs of a Polar Bear Facing the Bridge  The emissary | | JAPAN JAPAN  JAPAN | 1  1  1 | 2014  2013  2014 | 4  4  4 | 1960  1960  1960 | 1  1  1 | 0  0  0 | 0  0  0 | 0  0  0 | 0  0  0 | 0  0  0 | 0  0  0 | 0  0  0 | 0  0  0 | 0  0  0 | 0  0  0 | 0  0  0 | 0  0  0 | 0  0  0 | 0  0  0 | 0  0  0 |
| Taylor | Laini | | Strange the dreamer | | USA | 1 | 2017 | 2 | 1971 | 0 | 0 | 0 | 0 | 0 | 0 | 0 | 0 | 0 | 0 | 0 | 0 | 0 | 0 | 0 | 0 |
| Taylor | Bayard | | The Story of Kennett | | USA | 0 | 1866 | 2 | 1825 | 0 | 0 | 0 | 0 | 0 | 0 | 0 | 0 | 0 | 0 | 0 | 0 | 0 | 0 | 0 | 0 |
| Taylor | Peter | | A Summons to Memphis | | USA | 0 | 1986 | 2 | 1917 | 0 | 0 | 0 | 0 | 1 | 0 | 0 | 0 | 0 | 0 | 0 | 0 | 0 | 0 | 0 | 0 |
| Taylor | Brandon | | Real life | | USA | 0 | 2020 | 2 | 1989 | 0 | 0 | 0 | 0 | 0 | 0 | 0 | 0 | 0 | 0 | 0 | 0 | 1 | 1 | 1 | 0 |
| Taylor | Elizabeth | | Mrs. Palfrey at the Claremont | | UK | 1 | 1971 | 1 | 1912 | 0 | 0 | 0 | 0 | 0 | 0 | 0 | 0 | 0 | 0 | 0 | 0 | 0 | 0 | 0 | 0 |
| Taylor | Robert Lewis | | The Travels of Jaimie McPheeters | | USA | 0 | 1958 | 2 | 1912 | 0 | 0 | 0 | 0 | 1 | 0 | 0 | 0 | 0 | 0 | 0 | 0 | 0 | 0 | 0 | 0 |
| Tekin | Latife | | Swords of Ice | | TURKEY | 1 | 1989 | 4 | 1957 | 1 | 0 | 0 | 0 | 0 | 0 | 0 | 0 | 0 | 0 | 0 | 0 | 0 | 0 | 0 | 0 |
| Temple | Peter | | Truth | | SOUTH AFRICA | 0 | 2010 | 5 | 1946 | 0 | 0 | 0 | 0 | 0 | 0 | 0 | 0 | 0 | 0 | 0 | 0 | 1 | 0 | 0 | 0 |
| Teo | Sharlene | | Ponti | | SINGAPORE | 1 | 2018 | 4 | 1987 | 0 | 0 | 0 | 0 | 0 | 0 | 0 | 0 | 0 | 0 | 0 | 0 | 0 | 0 | 0 | 0 |
| Thackeray | illiam Makepea | | Vanity Fair | | UK | 0 | 1848 | 1 | 1811 | 0 | 0 | 0 | 0 | 0 | 0 | 0 | 0 | 0 | 0 | 0 | 0 | 0 | 0 | 0 | 0 |
| Thayil | Jeet | | Narcopolis | | INDIA | 0 | 2012 | 4 | 1959 | 0 | 0 | 0 | 0 | 0 | 0 | 0 | 0 | 0 | 0 | 0 | 0 | 0 | 0 | 0 | 0 |
| Thien | Madeleine | | Do Not Say We Have Nothing | | CANADA | 1 | 2016 | 2 | 1974 | 0 | 0 | 0 | 0 | 0 | 0 | 0 | 0 | 0 | 0 | 0 | 0 | 0 | 0 | 0 | 0 |
| Thiong'o  Thiong'o | Ngũgĩ wa  Ngũgĩ wa | | Wizard of the crow  The River Between | | KENYA  KENYA | 0  0 | 2019  1965 | 5  5 | 1938  1938 | 1  0 | 0  0 | 0  0 | 0  0 | 0  0 | 0  0 | 0  0 | 0  0 | 0  0 | 0  0 | 0  0 | 0  0 | 0  0 | 0  0 | 0  0 | 0  0 |
| Thirkell  Thirkell | Angela  Angela | | Before lunch  Wild Strawberries | | UK  UK | 1  1 | 1940  1934 | 1  1 | 1890  1890 | 0  0 | 0  0 | 0  0 | 0  0 | 0  0 | 0  0 | 0  0 | 0  0 | 0  0 | 0  0 | 0  0 | 0  0 | 0  0 | 0  0 | 0  0 | 0  0 |
| Thomas | Scarlett | | PopCo | | UK | 1 | 2005 | 1 | 1972 | 0 | 0 | 0 | 0 | 0 | 0 | 0 | 0 | 0 | 0 | 0 | 0 | 0 | 0 | 0 | 0 |
| Thomas | DM | | The White Hotel | | UK | 0 | 1981 | 1 | 1935 | 0 | 0 | 0 | 0 | 0 | 0 | 0 | 0 | 0 | 0 | 0 | 0 | 0 | 0 | 0 | 0 |
| Thompson | Harry | | This thing of darkness | | UK | 0 | 2005 | 1 | 1960 | 0 | 0 | 0 | 0 | 0 | 0 | 0 | 0 | 0 | 0 | 0 | 0 | 0 | 0 | 0 | 0 |
| Thoreau | Henry David | | Walden or life in the woods | | USA | 0 | 1854 | 2 | 1817 | 0 | 0 | 0 | 0 | 0 | 0 | 0 | 0 | 0 | 0 | 0 | 0 | 0 | 0 | 0 | 0 |
| Thurman | Wallace | | The Blacker the Berry | | USA | 0 | 1929 | 2 | 1902 | 0 | 0 | 0 | 0 | 0 | 0 | 0 | 0 | 0 | 0 | 0 | 0 | 0 | 0 | 0 | 0 |
| Thúy | Kim | | Riva | | VIETNAM | 1 | 2009 | 4 | 1968 | 1 | 0 | 0 | 0 | 0 | 0 | 0 | 0 | 0 | 0 | 0 | 0 | 0 | 0 | 0 | 0 |
| Toer Toer Toer  Toer | amoedya Anan amoedya Anan amoedya Anan  amoedya Anan | | Child of All Nations Footsteps  House of Glass  This Earth of Mankind | | INDONESIA INDONESIA INDONESIA  INDONESIA | 0  0  0  0 | 1980  1985  1988  1980 | 4  4  4  4 | 1925  1925  1925  1925 | 1  1  1  1 | 0  0  0  0 | 0  0  0  0 | 0  0  0  0 | 0  0  0  0 | 0  0  0  0 | 0  0  0  0 | 0  0  0  0 | 0  0  0  0 | 0  0  0  0 | 0  0  0  0 | 0  0  0  0 | 0  0  0  0 | 0  0  0  0 | 0  0  0  0 | 0  0  0  0 |
| Toews | Miriam | | All My Puny Sorrows | | CANADA | 1 | 2014 | 2 | 1964 | 0 | 0 | 0 | 0 | 0 | 0 | 0 | 0 | 0 | 0 | 0 | 0 | 0 | 0 | 0 | 0 |
| Tóibín  Tóibín | Colm  Colm | | Nora Webster  The Blackwater Lightship | | IRELAND  IRELAND | 0  0 | 2014  1999 | 1  1 | 1955  1955 | 0  0 | 0  0 | 0  0 | 0  0 | 0  0 | 0  0 | 0  0 | 0  0 | 0  0 | 0  0 | 0  0 | 0  0 | 1  0 | 0  0 | 1  0 | 0  0 |
| Tokarczuk Olga Flights POLAND 1 2007 1 1962 1 1 0 1 0 0 0 0 0 0 0 0 1 0 0 0  Tokarczuk Olga ive Your Plow Over the Bones of the De POLAND 1 2009 1 1962 1 1 0 0 0 0 0 0 0 0 0 0 0 0 0 0 | | | | | | | | | | | | | | | | | | | | | | | | | |
| Tolkien  Tolkien | JRR  JRR | | The lord of the rings  The hobbit | | UK  UK | 0  0 | 1955  1937 | 1  1 | 1892  1892 | 0  0 | 0  0 | 0  0 | 0  0 | 0  0 | 0  0 | 0  0 | 0  0 | 0  0 | 0  0 | 0  0 | 0  0 | 0  0 | 0  0 | 1  1 | 0  0 |
| Tolstoy  Tolstoy | Leo  Leo | | Anna Karenina  War and peace | | RUSSIA  RUSSIA | 0  0 | 1877  1869 | 1  1 | 1828  1828 | 1  1 | 0  0 | 0  0 | 0  0 | 0  0 | 0  0 | 0  0 | 0  0 | 0  0 | 0  0 | 0  0 | 0  0 | 0  0 | 0  0 | 1  1 | 0  0 |
| Toltz | Steve | | A Fraction of the Whole | | AUSTRALIA | 0 | 2008 | 6 | 1972 | 0 | 0 | 0 | 0 | 0 | 0 | 0 | 0 | 0 | 0 | 0 | 0 | 0 | 0 | 0 | 0 |
| Toole | John Kennedy | | A Confederacy of Dunces | | USA | 0 | 1980 | 2 | 1937 | 0 | 0 | 0 | 0 | 1 | 0 | 0 | 0 | 0 | 0 | 0 | 0 | 0 | 0 | 0 | 0 |
| Townsend | Sue | | e Secret Diary of Adrian Mole, Aged 13 | | UK | 1 | 1982 | 1 | 1946 | 0 | 0 | 0 | 0 | 0 | 0 | 0 | 0 | 0 | 0 | 0 | 0 | 0 | 0 | 0 | 0 |
| Travers | PL | | Mary Poppins | | AUSTRALIA | 1 | 1934 | 6 | 1899 | 0 | 0 | 0 | 0 | 0 | 0 | 0 | 0 | 0 | 0 | 0 | 0 | 0 | 0 | 0 | 0 |

| **SURNAME** | **NAME** | **TITLE** | **NATIONALITY** | | **GENDER** | **PUB_YEAR** | **CONTINENT** | **DATE OF BIRTH** | **TRANSLATED** | **NOBEL** | **NEUSTADT** | **BOOKER** | **PULITZER** | **NEBULA** | **CAINE** | **FRANZ KAFKA** | **NBA** | **BBA** | **WOMEN'S PRIZE FOR FICTION** | **NEW YORK TIMES** | **THE GUARDIAN** | **THE NEW YORKER** | **BBC** | **ATLANTIC** |
| --- | --- | --- | --- | --- | --- | --- | --- | --- | --- | --- | --- | --- | --- | --- | --- | --- | --- | --- | --- | --- | --- | --- | --- | --- |
| Tremain | Rose Restoration | | | UK | 1 | 1989 | 1 | 1943 | 0 | 0 | 0 | 0 | 0 | 0 | 0 | 0 | 0 | 0 | 0 | 0 | 0 | 0 | 0 | 0 |
| Trevor | William The Children of Dynmouth | | | IRELAND | 0 | 1976 | 1 | 1928 | 0 | 0 | 0 | 0 | 0 | 0 | 0 | 0 | 0 | 0 | 0 | 0 | 0 | 0 | 0 | 0 |
| Trollope | Anthony Phineas Finn | | | UK | 0 | 1869 | 1 | 1815 | 0 | 0 | 0 | 0 | 0 | 0 | 0 | 0 | 0 | 0 | 0 | 0 | 0 | 0 | 0 | 0 |
| Trollope | Anthony The Last Chronicle of Barset | | | UK | 0 | 1867 | 1 | 1815 | 0 | 0 | 0 | 0 | 0 | 0 | 0 | 0 | 0 | 0 | 0 | 0 | 0 | 0 | 0 | 0 |
| Tsiolkas | Christos The slap | | | AUSTRALIA | 0 | 2008 | 6 | 1965 | 0 | 0 | 0 | 0 | 0 | 0 | 0 | 0 | 0 | 0 | 0 | 0 | 0 | 0 | 0 | 0 |
| Tucker | Wilson The Time Masters | | | USA | 0 | 1953 | 2 | 1914 | 0 | 0 | 0 | 0 | 0 | 0 | 0 | 0 | 0 | 0 | 0 | 0 | 0 | 0 | 0 | 0 |
| Tucker | Wilson The Year of the Quiet Sun | | | USA | 0 | 1970 | 2 | 1914 | 0 | 0 | 0 | 0 | 0 | 0 | 0 | 0 | 0 | 0 | 0 | 0 | 0 | 0 | 0 | 0 |
| Turgenev | Ivan Fathers and children | | | RUSSIA | 0 | 1862 | 1 | 1818 | 1 | 0 | 0 | 0 | 0 | 0 | 0 | 0 | 0 | 0 | 0 | 0 | 0 | 0 | 0 | 0 |
| Turgenev | Ivan Diary of a superfluous man | | | RUSSIA | 0 | 1850 | 1 | 1818 | 1 | 0 | 0 | 0 | 0 | 0 | 0 | 0 | 0 | 0 | 0 | 0 | 0 | 0 | 0 | 0 |
| Turgenev | Ivan The Torrents of Spring | | | RUSSIA | 0 | 1872 | 1 | 1818 | 1 | 0 | 0 | 0 | 0 | 0 | 0 | 0 | 0 | 0 | 0 | 0 | 0 | 0 | 0 | 0 |
| Turner | Ethel Seven Little Australians | | | UK | 1 | 1894 | 1 | 1870 | 0 | 0 | 0 | 0 | 0 | 0 | 0 | 0 | 0 | 0 | 0 | 0 | 0 | 0 | 0 | 0 |
| Tutuola | Amos My life in the bush of ghosts | | | NIGERIA | 0 | 1954 | 5 | 1920 | 0 | 0 | 0 | 0 | 0 | 0 | 0 | 0 | 0 | 0 | 0 | 0 | 0 | 0 | 0 | 0 |
| Twain | Mark Adventures of Tom Sawyer | | | USA | 0 | 1876 | 2 | 1835 | 0 | 0 | 0 | 0 | 0 | 0 | 0 | 0 | 0 | 0 | 0 | 0 | 0 | 0 | 0 | 0 |
| Twain | Mark Adventures of Huckleberry Finn | | | USA | 0 | 1884 | 2 | 1835 | 0 | 0 | 0 | 0 | 0 | 0 | 0 | 0 | 0 | 0 | 0 | 0 | 0 | 0 | 0 | 0 |
| Twain | Mark Connecticut yankee in King Arthur's co | | | USA | 0 | 1889 | 2 | 1835 | 0 | 0 | 0 | 0 | 0 | 0 | 0 | 0 | 0 | 0 | 0 | 0 | 0 | 0 | 0 | 0 |
| Twan Eng | Tan The gift of rain | | | MALAYSIA | 0 | 2007 | 4 | 1972 | 0 | 0 | 0 | 0 | 0 | 0 | 0 | 0 | 0 | 0 | 0 | 0 | 0 | 0 | 0 | 0 |
| Twan Eng | Tan The garden of evening mists | | | MALAYSIA | 0 | 2012 | 4 | 1972 | 0 | 0 | 0 | 0 | 0 | 0 | 0 | 0 | 0 | 0 | 0 | 0 | 0 | 0 | 0 | 0 |
| Tyler | Anne Breathing Lessons | | | USA | 1 | 1988 | 2 | 1941 | 0 | 0 | 0 | 0 | 1 | 0 | 0 | 0 | 0 | 0 | 0 | 0 | 0 | 0 | 0 | 0 |
| Tynan | Katharine An isle in the water | | | IRELAND | 1 | 1896 | 1 | 1859 | 0 | 0 | 0 | 0 | 0 | 0 | 0 | 0 | 0 | 0 | 0 | 0 | 0 | 0 | 0 | 0 |
| Tynan | Katharine Mary Gray | | | IRELAND | 1 | 1909 | 1 | 1859 | 0 | 0 | 0 | 0 | 0 | 0 | 0 | 0 | 0 | 0 | 0 | 0 | 0 | 0 | 0 | 0 |
| Tynan | Katharine The great captain | | | IRELAND | 1 | 1902 | 1 | 1859 | 0 | 0 | 0 | 0 | 0 | 0 | 0 | 0 | 0 | 0 | 0 | 0 | 0 | 0 | 0 | 0 |
| Tynan | Katharine The story of bawn | | | IRELAND | 1 | 1906 | 1 | 1859 | 0 | 0 | 0 | 0 | 0 | 0 | 0 | 0 | 0 | 0 | 0 | 0 | 0 | 0 | 0 | 0 |
| Tyszka | Alberto Barrera The Sickness | | | VENEZUELA | 0 | 2006 | 3 | 1960 | 1 | 0 | 0 | 0 | 0 | 0 | 0 | 0 | 0 | 0 | 0 | 0 | 0 | 0 | 0 | 0 |
| Ugrešić | Dubravka The Ministry of Pain | | | CROATIA | 1 | 2004 | 1 | 1949 | 1 | 0 | 1 | 0 | 0 | 0 | 0 | 0 | 0 | 0 | 0 | 0 | 0 | 0 | 0 | 0 |
| Uhlman | Fred Reunion | | | GERMANY | 0 | 1971 | 1 | 1901 | 0 | 0 | 0 | 0 | 0 | 0 | 0 | 0 | 0 | 0 | 0 | 0 | 0 | 0 | 0 | 0 |
| Un-su | Kim The plotters | | | SOUTH KOREA | 0 | 2010 | 4 | 1972 | 1 | 0 | 0 | 0 | 0 | 0 | 0 | 0 | 0 | 0 | 0 | 0 | 0 | 0 | 0 | 0 |
| Undset | Sigrid Catherine Of Siena | | | NORWAY | 1 | 1951 | 1 | 1882 | 1 | 1 | 0 | 0 | 0 | 0 | 0 | 0 | 0 | 0 | 0 | 0 | 0 | 0 | 0 | 0 |
| Ung | Loung First They Killed My Father | | | CAMBODIA | 1 | 2000 | 4 | 1970 | 0 | 0 | 0 | 0 | 0 | 0 | 0 | 0 | 0 | 0 | 0 | 0 | 0 | 0 | 0 | 0 |
| Unsworth | Barry Sacred Hunger | | | UK | 0 | 1992 | 1 | 1930 | 0 | 0 | 0 | 1 | 0 | 0 | 0 | 0 | 0 | 0 | 0 | 0 | 0 | 0 | 0 | 0 |
| Unsworth | Barry Morality Play | | | UK | 0 | 1995 | 1 | 1930 | 0 | 0 | 0 | 0 | 0 | 0 | 0 | 0 | 0 | 0 | 0 | 0 | 0 | 0 | 0 | 0 |
| Upadhyay | Samrat Arresting God in Kathmandu | | | NEPAL | 0 | 2001 | 4 | 1974 | 0 | 0 | 0 | 0 | 0 | 0 | 0 | 0 | 0 | 0 | 0 | 0 | 0 | 0 | 0 | 0 |
| Upadhyay | Samrat The Guru of Love | | | NEPAL | 0 | 2003 | 4 | 1974 | 0 | 0 | 0 | 0 | 0 | 0 | 0 | 0 | 0 | 0 | 0 | 0 | 0 | 0 | 0 | 0 |
| Updike | John Rabbit Is Rich | | | USA | 0 | 1981 | 2 | 1932 | 0 | 0 | 0 | 0 | 1 | 0 | 0 | 0 | 1 | 0 | 0 | 0 | 0 | 0 | 0 | 0 |
| Van Dine | SS The Canary Murder Case | | | USA | 0 | 1927 | 2 | 1888 | 0 | 0 | 0 | 0 | 0 | 0 | 0 | 0 | 0 | 0 | 0 | 0 | 0 | 0 | 0 | 0 |
| Van Dine | SS The Greene Murder Case | | | USA | 0 | 1928 | 2 | 1888 | 0 | 0 | 0 | 0 | 0 | 0 | 0 | 0 | 0 | 0 | 0 | 0 | 0 | 0 | 0 | 0 |
| van Niekerk | Marlene Triomf | | | SOUTH AFRICA | 1 | 2004 | 5 | 1954 | 1 | 0 | 0 | 0 | 0 | 0 | 0 | 0 | 0 | 0 | 0 | 0 | 0 | 0 | 0 | 0 |
| van Niekerk | Marlene Agaat | | | SOUTH AFRICA | 1 | 2004 | 5 | 1954 | 1 | 0 | 0 | 0 | 0 | 0 | 0 | 0 | 0 | 0 | 0 | 0 | 0 | 0 | 0 | 0 |
| Vargas | Fred The three evangelists | | | FRANCE | 1 | 1995 | 1 | 1957 | 1 | 0 | 0 | 0 | 0 | 0 | 0 | 0 | 0 | 0 | 0 | 0 | 0 | 0 | 0 | 0 |
| Vargas | Jose F Dear America: Notes | | | PHILIPPINES | 0 | 2018 | 4 | 1981 | 0 | 0 | 0 | 0 | 0 | 0 | 0 | 0 | 0 | 0 | 0 | 0 | 0 | 0 | 0 | 0 |
| Vargas Llosa | Mario Captain Pantoja and the Special Service | | | PERU | 0 | 1973 | 3 | 1936 | 1 | 1 | 0 | 0 | 0 | 0 | 0 | 0 | 0 | 0 | 0 | 0 | 0 | 0 | 0 | 0 |
| Vargas Llosa | Mario In Praise of the Stepmother | | | PERU | 0 | 1988 | 3 | 1936 | 1 | 1 | 0 | 0 | 0 | 0 | 0 | 0 | 0 | 0 | 0 | 0 | 0 | 0 | 0 | 0 |
| Vargas Llosa | Mario The bad girl | | | PERU | 0 | 2006 | 3 | 1936 | 1 | 1 | 0 | 0 | 0 | 0 | 0 | 0 | 0 | 0 | 0 | 0 | 0 | 0 | 0 | 0 |
| Vargas Llosa | Mario The Time of the Hero | | | PERU | 0 | 1962 | 3 | 1936 | 1 | 1 | 0 | 0 | 0 | 0 | 0 | 0 | 0 | 0 | 0 | 0 | 0 | 0 | 0 | 0 |
| Vásquez | Juan Gabriel The informers | | | COLOMBIA | 0 | 2004 | 3 | 1973 | 1 | 0 | 0 | 0 | 0 | 0 | 0 | 0 | 0 | 0 | 0 | 0 | 0 | 0 | 0 | 0 |
| Vásquez | Juan Gabriel Reputations | | | COLOMBIA | 0 | 2013 | 3 | 1973 | 1 | 0 | 0 | 0 | 0 | 0 | 0 | 0 | 0 | 0 | 0 | 0 | 0 | 0 | 0 | 0 |
| Verga | Giovanni I Malavoglia | | | ITALY | 0 | 1881 | 1 | 1840 | 1 | 0 | 0 | 0 | 0 | 0 | 0 | 0 | 0 | 0 | 0 | 0 | 0 | 0 | 0 | 0 |
| Verne | Jules A journey to the center of the Earth | | | FRANCE | 0 | 1864 | 1 | 1828 | 1 | 0 | 0 | 0 | 0 | 0 | 0 | 0 | 0 | 0 | 0 | 0 | 0 | 0 | 0 | 0 |
| Verne | Jules Twenty thousand leagues under the sea | | | FRANCE | 0 | 1870 | 1 | 1828 | 1 | 0 | 0 | 0 | 0 | 0 | 0 | 0 | 0 | 0 | 0 | 0 | 0 | 0 | 0 | 0 |
| Verne | Jules The master of the world | | | FRANCE | 0 | 1904 | 1 | 1828 | 1 | 0 | 0 | 0 | 0 | 0 | 0 | 0 | 0 | 0 | 0 | 0 | 0 | 0 | 0 | 0 |
| Verne | Jules Around the world in 80 days | | | FRANCE | 0 | 1873 | 1 | 1828 | 1 | 0 | 0 | 0 | 0 | 0 | 0 | 0 | 0 | 0 | 0 | 0 | 0 | 0 | 0 | 0 |
| Vian | Boris I spit on your graves | | | FRANCE | 0 | 1946 | 1 | 1920 | 1 | 0 | 0 | 0 | 0 | 0 | 0 | 0 | 0 | 0 | 0 | 0 | 0 | 0 | 0 | 0 |
| Vidal | Gore The City and the Pillar | | | USA | 0 | 1946 | 2 | 1925 | 1 | 0 | 0 | 0 | 0 | 0 | 0 | 0 | 0 | 0 | 0 | 0 | 0 | 0 | 0 | 0 |
| Viggers | Karen The lightkeeper's wife | | | AUSTRALIA | 1 | 2011 | 6 | ? | 0 | 0 | 0 | 0 | 0 | 0 | 0 | 0 | 0 | 0 | 0 | 0 | 0 | 0 | 0 | 0 |
| Vila-Matas | Enrique Bartleby & co | | | SPAIN | 0 | 2000 | 1 | 1948 | 1 | 0 | 0 | 0 | 0 | 0 | 0 | 0 | 0 | 0 | 0 | 0 | 0 | 0 | 0 | 0 |
| Villalobos | Juan Pablo Down the Rabbit Hole | | | MEXICO | 0 | 2011 | 3 | 1973 | 1 | 0 | 0 | 0 | 0 | 0 | 0 | 0 | 0 | 0 | 0 | 0 | 0 | 0 | 0 | 0 |
| Villalobos | Juan Pablo I Don't Expect Anyone to Believe Me | | | MEXICO | 0 | 2020 | 3 | 1973 | 1 | 0 | 0 | 0 | 0 | 0 | 0 | 0 | 0 | 0 | 0 | 0 | 0 | 0 | 0 | 0 |
| Villalobos | Juan Pablo I'll Sell You a Dog | | | MEXICO | 0 | 2016 | 3 | 1973 | 1 | 0 | 0 | 0 | 0 | 0 | 0 | 0 | 0 | 0 | 0 | 0 | 0 | 0 | 0 | 0 |
| Vinci | Simona What we don't know about children | | | ITALY | 1 | 1997 | 1 | 1970 | 1 | 0 | 0 | 0 | 0 | 0 | 0 | 0 | 0 | 0 | 0 | 0 | 0 | 0 | 0 | 0 |
| Volpi | Jorge In Search of Klingsor | | | MEXICO | 0 | 1999 | 3 | 1968 | 1 | 0 | 0 | 0 | 0 | 0 | 0 | 0 | 0 | 0 | 0 | 0 | 0 | 0 | 0 | 0 |
| Volpi | Jorge In Spite of the Dark Silence | | | MEXICO | 0 | 1993 | 3 | 1968 | 1 | 0 | 0 | 0 | 0 | 0 | 0 | 0 | 0 | 0 | 0 | 0 | 0 | 0 | 0 | 0 |
| von Arnim | Elizabeth Vera | | | AUSTRALIA | 1 | 1921 | 6 | 1866 | 0 | 0 | 0 | 0 | 0 | 0 | 0 | 0 | 0 | 0 | 0 | 0 | 0 | 0 | 0 | 0 |
| von Arnim | Elizabeth Princess Priscilla's Fortnight | | | AUSTRALIA | 1 | 1905 | 6 | 1866 | 0 | 0 | 0 | 0 | 0 | 0 | 0 | 0 | 0 | 0 | 0 | 0 | 0 | 0 | 0 | 0 |
| von Arnim | Elizabeth The enchanted April | | | AUSTRALIA | 1 | 1922 | 6 | 1866 | 0 | 0 | 0 | 0 | 0 | 0 | 0 | 0 | 0 | 0 | 0 | 0 | 0 | 0 | 0 | 0 |
| von Goethe | ohann Wolfgan The sorrows of young Werther | | | GERMANY | 0 | 1774 | 1 | 1749 | 1 | 0 | 0 | 0 | 0 | 0 | 0 | 0 | 0 | 0 | 0 | 0 | 0 | 0 | 0 | 0 |
| von Goethe | ohann Wolfgan Elective affinities | | | GERMANY | 0 | 1809 | 1 | 1749 | 1 | 0 | 0 | 0 | 0 | 0 | 0 | 0 | 0 | 0 | 0 | 0 | 0 | 0 | 0 | 0 |
| von Kleist | Heinrich The Marquise of O | | | GERMANY | 0 | 1808 | 1 | 1777 | 1 | 0 | 0 | 0 | 0 | 0 | 0 | 0 | 0 | 0 | 0 | 0 | 0 | 0 | 0 | 0 |
| von Kleist | Heinrich Michael Kohlhaas | | | GERMANY | 0 | 1810 | 1 | 1777 | 1 | 0 | 0 | 0 | 0 | 0 | 0 | 0 | 0 | 0 | 0 | 0 | 0 | 0 | 0 | 0 |
| Vonnegut | Kurt Mother night | | | USA | 0 | 1961 | 2 | 1922 | 0 | 0 | 0 | 0 | 0 | 0 | 0 | 0 | 0 | 0 | 0 | 0 | 0 | 0 | 0 | 0 |
| Vonnegut | Kurt Cat's Cradle | | | USA | 0 | 1963 | 2 | 1922 | 0 | 0 | 0 | 0 | 0 | 0 | 0 | 0 | 0 | 0 | 0 | 0 | 0 | 0 | 0 | 0 |
| Vuong | Ocean On Earth We’re Briefly Gorgeous | | | USA | 0 | 2019 | 2 | 1988 | 0 | 0 | 0 | 0 | 0 | 0 | 0 | 0 | 0 | 0 | 0 | 0 | 1 | 1 | 1 | 0 |
| Wainaina | Binyavanga One day I will write about this place | | | KENYA | 0 | 2011 | 5 | 1971 | 0 | 0 | 0 | 0 | 0 | 0 | 0 | 0 | 0 | 0 | 0 | 0 | 0 | 0 | 0 | 0 |
| Walker | Alice The color purple | | | USA | 1 | 1982 | 2 | 1944 | 0 | 0 | 0 | 0 | 1 | 0 | 0 | 0 | 1 | 0 | 0 | 0 | 0 | 0 | 0 | 0 |
| Wallace | David Foster Infinite Jest | | | USA | 0 | 1996 | 2 | 1962 | 0 | 0 | 0 | 0 | 0 | 0 | 0 | 0 | 0 | 0 | 0 | 0 | 0 | 0 | 0 | 0 |
| Wallace | David Foster supposedly fun thing I'll never do agai | | | USA | 0 | 1997 | 2 | 1962 | 0 | 0 | 0 | 0 | 0 | 0 | 0 | 0 | 0 | 0 | 0 | 0 | 0 | 0 | 0 | 0 |
| Wallace | Lew Ben-Hur: A tale of the Christ | | | USA | 0 | 1880 | 2 | 1827 | 0 | 0 | 0 | 0 | 0 | 0 | 0 | 0 | 0 | 0 | 0 | 0 | 0 | 0 | 0 | 0 |
| Waller | Robert James The bridges of Madison County | | | USA | 0 | 1992 | 2 | 1939 | 0 | 0 | 0 | 0 | 0 | 0 | 0 | 0 | 0 | 0 | 0 | 0 | 0 | 0 | 0 | 0 |
| Walpole | Horace The castle of Otranto | | | UK | 0 | 1764 | 1 | 1717 | 0 | 0 | 0 | 0 | 0 | 0 | 0 | 0 | 0 | 0 | 0 | 0 | 0 | 0 | 0 | 0 |
| Walser | Robert Jakob Von Gunten | | | SWITZERLAND | 0 | 1908 | 1 | 1878 | 1 | 0 | 0 | 0 | 0 | 0 | 0 | 0 | 0 | 0 | 0 | 0 | 0 | 0 | 0 | 0 |
| Wamariya | Clemantine The girl who smiled beads | | | RWANDA | 1 | 2018 | 5 | 1988 | 0 | 0 | 0 | 0 | 0 | 0 | 0 | 0 | 0 | 0 | 0 | 0 | 0 | 0 | 0 | 0 |
| Wambaugh | Joseph The Black Marble | | | USA | 0 | 1978 | 2 | 1937 | 0 | 0 | 0 | 0 | 0 | 0 | 0 | 0 | 0 | 0 | 0 | 0 | 0 | 0 | 0 | 0 |
| Wambaugh | Joseph The Choirboys | | | USA | 0 | 1975 | 2 | 1937 | 0 | 0 | 0 | 0 | 0 | 0 | 0 | 0 | 0 | 0 | 0 | 0 | 0 | 0 | 0 | 0 |
| Warren | Patricia The Front Runner | | | USA | 1 | 1974 | 2 | 1936 | 0 | 0 | 0 | 0 | 0 | 0 | 0 | 0 | 0 | 0 | 0 | 0 | 0 | 0 | 0 | 0 |
| Warren | Robert Penn All the King's Men | | | USA | 0 | 1946 | 2 | 1905 | 0 | 0 | 0 | 0 | 1 | 0 | 0 | 0 | 0 | 0 | 0 | 0 | 0 | 0 | 0 | 0 |
| Washburn | Kawai Sharks in the Time of Saviors | | | USA | 0 | 2020 | 2 | ? | 0 | 0 | 0 | 0 | 0 | 0 | 0 | 0 | 0 | 0 | 0 | 0 | 0 | 0 | 0 | 0 |
| Washington | Bryan Lot | | | USA | 0 | 2019 | 2 | 1993 | 0 | 0 | 0 | 0 | 0 | 0 | 0 | 0 | 0 | 0 | 0 | 0 | 0 | 0 | 0 | 0 |
| Wataya | Risa I want to kick you in the back | | | JAPAN | 1 | 2003 | 4 | 1984 | 1 | 0 | 0 | 0 | 0 | 0 | 0 | 0 | 0 | 0 | 0 | 0 | 0 | 0 | 0 | 0 |
| Waters | Sarah Fingersmith | | | UK | 1 | 2002 | 1 | 1966 | 0 | 0 | 0 | 0 | 0 | 0 | 0 | 0 | 0 | 1 | 0 | 0 | 0 | 0 | 0 | 0 |

| **SURNAME** | **NAME** | **TITLE** | **NATIONALITY** | **GENDER** | **PUB_YEAR** | **CONTINENT** | **DATE OF BIRTH** | **TRANSLATED** | **NOBEL** | **NEUSTADT** | **BOOKER** | **PULITZER** | **NEBULA** | **CAINE** | **FRANZ KAFKA** | **NBA** | **BBA** | **WOMEN'S PRIZE FOR FICTION** | **NEW YORK TIMES** | **THE GUARDIAN** | **THE NEW YORKER** | **BBC** | **ATLANTIC** |
| --- | --- | --- | --- | --- | --- | --- | --- | --- | --- | --- | --- | --- | --- | --- | --- | --- | --- | --- | --- | --- | --- | --- | --- |
| Weldon | Fay | Praxis | UK | 1 | 1978 | 1 | 1931 | 0 | 0 | 0 | 0 | 0 | 0 | 0 | 0 | 0 | 0 | 0 | 0 | 0 | 0 | 0 | 0 |
| Wells | HG | The Island Of Dr Moreau | UK | 0 | 1896 | 1 | 1866 | 0 | 0 | 0 | 0 | 0 | 0 | 0 | 0 | 0 | 0 | 0 | 0 | 0 | 0 | 0 | 0 |
| Wells | HG | The Time Machine | UK | 0 | 1895 | 1 | 1866 | 0 | 0 | 0 | 0 | 0 | 0 | 0 | 0 | 0 | 0 | 0 | 0 | 0 | 0 | 0 | 0 |
| Welsapar | Ak | The Tale of Aypi | TURKMENISTAN | 0 | 2015 | 4 | 1956 | 1 | 0 | 0 | 0 | 0 | 0 | 0 | 0 | 0 | 0 | 0 | 0 | 0 | 0 | 0 | 0 |
| Welty | Eudora | The Optimist's Daughter | USA | 1 | 1972 | 2 | 1909 | 0 | 0 | 0 | 0 | 1 | 0 | 0 | 0 | 0 | 0 | 0 | 0 | 0 | 0 | 0 | 0 |
| Wendt | Albert | Breaking Connections | SAMOA | 0 | 2015 | 6 | 1939 | 0 | 0 | 0 | 0 | 0 | 0 | 0 | 0 | 0 | 0 | 0 | 0 | 0 | 0 | 0 | 0 |
| Wendt | Albert | The Adventures of Vela | SAMOA | 0 | 2009 | 6 | 1939 | 0 | 0 | 0 | 0 | 0 | 0 | 0 | 0 | 0 | 0 | 0 | 0 | 0 | 0 | 0 | 0 |
| West | Nathanael | Miss lonelyhearts | USA | 0 | 1933 | 2 | 1903 | 0 | 0 | 0 | 0 | 0 | 0 | 0 | 0 | 0 | 0 | 0 | 0 | 0 | 0 | 0 | 0 |
| West | Rebecca | The fountain overflows | UK | 1 | 1956 | 1 | 1892 | 0 | 0 | 0 | 0 | 0 | 0 | 0 | 0 | 0 | 0 | 0 | 0 | 0 | 0 | 0 | 0 |
| West | Rebecca | This real night | UK | 1 | 1984 | 1 | 1892 | 0 | 0 | 0 | 0 | 0 | 0 | 0 | 0 | 0 | 0 | 0 | 0 | 0 | 0 | 0 | 0 |
| West | Morris | Daughter of silence | AUSTRALIA | 0 | 1961 | 6 | 1916 | 0 | 0 | 0 | 0 | 0 | 0 | 0 | 0 | 0 | 0 | 0 | 0 | 0 | 0 | 0 | 0 |
| West | Morris | Summer of the red wolf | AUSTRALIA | 0 | 1971 | 6 | 1916 | 0 | 0 | 0 | 0 | 0 | 0 | 0 | 0 | 0 | 0 | 0 | 0 | 0 | 0 | 0 | 0 |
| West | Dorothy | The Living Is Easy | USA | 1 | 1948 | 2 | 1907 | 0 | 0 | 0 | 0 | 0 | 0 | 0 | 0 | 0 | 0 | 0 | 0 | 0 | 0 | 0 | 0 |
| West | Dorothy | The Wedding | USA | 1 | 1995 | 2 | 1907 | 0 | 0 | 0 | 0 | 0 | 0 | 0 | 0 | 0 | 0 | 0 | 0 | 0 | 0 | 0 | 0 |
| Westlake | Donald | The Hot Rock | USA | 0 | 1970 | 2 | 1933 | 0 | 0 | 0 | 0 | 0 | 0 | 0 | 0 | 0 | 0 | 0 | 0 | 0 | 0 | 0 | 0 |
| Westlake | Donald | The hunter | USA | 0 | 1962 | 2 | 1933 | 0 | 0 | 0 | 0 | 0 | 0 | 0 | 0 | 0 | 0 | 0 | 0 | 0 | 0 | 0 | 0 |
| Westover | Tara | Educated | USA | 1 | 2018 | 2 | 1986 | 0 | 0 | 0 | 0 | 0 | 0 | 0 | 0 | 0 | 0 | 0 | 0 | 0 | 0 | 0 | 0 |
| Wharton | Edith | Age of innocence | USA | 1 | 1920 | 2 | 1862 | 0 | 0 | 0 | 0 | 1 | 0 | 0 | 0 | 0 | 0 | 0 | 0 | 0 | 0 | 0 | 0 |
| Wharton | Edith | Summer | USA | 1 | 1917 | 2 | 1862 | 0 | 0 | 0 | 0 | 0 | 0 | 0 | 0 | 0 | 0 | 0 | 0 | 0 | 0 | 0 | 0 |
| Wharton | Edith | The Glimpses of the Moon | USA | 1 | 1922 | 2 | 1862 | 0 | 0 | 0 | 0 | 0 | 0 | 0 | 0 | 0 | 0 | 0 | 0 | 0 | 0 | 0 | 0 |
| Wharton | Edith | The House of Mirth | USA | 1 | 1905 | 2 | 1862 | 0 | 0 | 0 | 0 | 0 | 0 | 0 | 0 | 0 | 0 | 0 | 0 | 0 | 0 | 0 | 0 |
| Wharton | Edith | The Mother's Recompense | USA | 1 | 1925 | 2 | 1862 | 0 | 0 | 0 | 0 | 0 | 0 | 0 | 0 | 0 | 0 | 0 | 0 | 0 | 0 | 0 | 0 |
| Wharton | Edith | The Reef | USA | 1 | 1912 | 2 | 1862 | 0 | 0 | 0 | 0 | 0 | 0 | 0 | 0 | 0 | 0 | 0 | 0 | 0 | 0 | 0 | 0 |
| Wharton | Edith | The Valley of Decision | USA | 1 | 1902 | 2 | 1862 | 0 | 0 | 0 | 0 | 0 | 0 | 0 | 0 | 0 | 0 | 0 | 0 | 0 | 0 | 0 | 0 |
| Wharton | Edith | Twilight Sleep | USA | 1 | 1927 | 2 | 1862 | 0 | 0 | 0 | 0 | 0 | 0 | 0 | 0 | 0 | 0 | 0 | 0 | 0 | 0 | 0 | 0 |
| White | Patrick | The hanging garden | AUSTRALIA | 0 | 2012 | 6 | 1912 | 0 | 1 | 0 | 0 | 0 | 0 | 0 | 0 | 0 | 0 | 0 | 0 | 0 | 0 | 0 | 0 |
| White | Patrick | Voss | AUSTRALIA | 0 | 1957 | 6 | 1912 | 0 | 1 | 0 | 0 | 0 | 0 | 0 | 0 | 0 | 0 | 0 | 0 | 0 | 0 | 0 | 0 |
| Whitehead | Colson | The Nickel Boys | USA | 0 | 2019 | 2 | 1969 | 0 | 0 | 0 | 0 | 1 | 0 | 0 | 0 | 0 | 0 | 0 | 0 | 1 | 0 | 1 | 0 |
| Whitehead | Colson | The Underground Railroad | USA | 0 | 2016 | 2 | 1969 | 0 | 0 | 0 | 0 | 1 | 0 | 0 | 0 | 1 | 0 | 0 | 1 | 1 | 0 | 0 | 0 |
| Wiesel | Elie | Night | USA | 0 | 1958 | 2 | 1928 | 1 | 0 | 0 | 0 | 0 | 0 | 0 | 0 | 0 | 0 | 0 | 0 | 0 | 0 | 0 | 0 |
| Wilde | Oscar | The picture of Dorian Gray | UK | 0 | 1890 | 1 | 1854 | 0 | 0 | 0 | 0 | 0 | 0 | 0 | 0 | 0 | 0 | 0 | 0 | 0 | 0 | 0 | 0 |
| Wilder | Thornton | The Bridge of San Luis Rey | USA | 0 | 1927 | 2 | 1897 | 0 | 0 | 0 | 0 | 1 | 0 | 0 | 0 | 0 | 0 | 0 | 0 | 0 | 0 | 0 | 0 |
| Williams | John | Stoner | USA | 0 | 1965 | 2 | 1922 | 0 | 0 | 0 | 0 | 0 | 0 | 0 | 0 | 0 | 0 | 0 | 0 | 0 | 0 | 0 | 0 |
| Williams | John | Nothing but the night | USA | 0 | 1948 | 2 | 1922 | 0 | 0 | 0 | 0 | 0 | 0 | 0 | 0 | 0 | 0 | 0 | 0 | 0 | 0 | 0 | 0 |
| Wilson | Margaret | The Able McLaughlins | USA | 1 | 1923 | 2 | 1882 | 0 | 0 | 0 | 0 | 1 | 0 | 0 | 0 | 0 | 0 | 0 | 0 | 0 | 0 | 0 | 0 |
| Winfrey | Oprah | What I Know For Sure | USA | 1 | 2014 | 2 | 1954 | 0 | 0 | 0 | 0 | 0 | 0 | 0 | 0 | 0 | 0 | 0 | 0 | 0 | 0 | 0 | 0 |
| Winslow | Don | The border | USA | 0 | 2019 | 2 | 1953 | 0 | 0 | 0 | 0 | 0 | 0 | 0 | 0 | 0 | 0 | 0 | 0 | 0 | 0 | 0 | 0 |
| Winters | Ben | Underground Airlines | USA | 0 | 2016 | 2 | 1976 | 0 | 0 | 0 | 0 | 0 | 0 | 0 | 0 | 0 | 0 | 0 | 0 | 0 | 0 | 1 | 1 |
| Winterson | Jeanette | Written on the body | UK | 1 | 1992 | 1 | 1959 | 0 | 0 | 0 | 0 | 0 | 0 | 0 | 0 | 0 | 0 | 0 | 0 | 0 | 0 | 0 | 0 |
| Winton | Tim | Eyrie | AUSTRALIA | 0 | 2013 | 6 | 1960 | 0 | 0 | 0 | 0 | 0 | 0 | 0 | 0 | 0 | 0 | 0 | 0 | 0 | 0 | 0 | 0 |
| Winton | Tim | The turning | AUSTRALIA | 0 | 2004 | 6 | 1960 | 0 | 0 | 0 | 0 | 0 | 0 | 0 | 0 | 0 | 0 | 0 | 0 | 0 | 0 | 0 | 0 |
| Winton | Tim | Breath | AUSTRALIA | 0 | 2008 | 6 | 1960 | 0 | 0 | 0 | 0 | 0 | 0 | 0 | 0 | 0 | 0 | 0 | 0 | 0 | 0 | 0 | 0 |
| Winton | Tim | Cloudstreet | AUSTRALIA | 0 | 1991 | 6 | 1960 | 0 | 0 | 0 | 0 | 0 | 0 | 0 | 0 | 0 | 0 | 0 | 0 | 0 | 0 | 0 | 0 |
| Witting | Amy | I for Isobel | AUSTRALIA | 1 | 1990 | 6 | 1918 | 0 | 0 | 0 | 0 | 0 | 0 | 0 | 0 | 0 | 0 | 0 | 0 | 0 | 0 | 0 | 0 |
| Wolf | Christa | Medea | GERMANY | 1 | 1996 | 1 | 1929 | 1 | 0 | 0 | 0 | 0 | 0 | 0 | 0 | 0 | 0 | 0 | 0 | 0 | 0 | 0 | 0 |
| Wolf | Christa | They divided the sky | GERMANY | 1 | 1963 | 1 | 1929 | 1 | 0 | 0 | 0 | 0 | 0 | 0 | 0 | 0 | 0 | 0 | 0 | 0 | 0 | 0 | 0 |
| Wollstonecraft | Mary | Frankenstein | UK | 1 | 1818 | 1 | 1759 | 0 | 0 | 0 | 0 | 0 | 0 | 0 | 0 | 0 | 0 | 0 | 0 | 0 | 0 | 0 | 0 |
| Wollstonecraft | Mary | The last man | UK | 1 | 1826 | 1 | 1759 | 0 | 0 | 0 | 0 | 0 | 0 | 0 | 0 | 0 | 0 | 0 | 0 | 0 | 0 | 0 | 0 |
| Woodward | Gerard | I'll Go to Bed at Noon | UK | 0 | 2004 | 1 | 1961 | 0 | 0 | 0 | 0 | 0 | 0 | 0 | 0 | 0 | 0 | 0 | 0 | 0 | 0 | 0 | 0 |
| Woolf | Virginia | Mrs Dalloway | UK | 1 | 1925 | 1 | 1882 | 0 | 0 | 0 | 0 | 0 | 0 | 0 | 0 | 0 | 0 | 0 | 0 | 0 | 0 | 0 | 0 |
| Woolf | Virginia | To the lighthouse | UK | 1 | 1927 | 1 | 1882 | 0 | 0 | 0 | 0 | 0 | 0 | 0 | 0 | 0 | 0 | 0 | 0 | 0 | 0 | 0 | 0 |
| Woolf | Virginia | Orlando | UK | 1 | 1928 | 1 | 1882 | 0 | 0 | 0 | 0 | 0 | 0 | 0 | 0 | 0 | 0 | 0 | 0 | 0 | 0 | 0 | 0 |
| Woolf | Virginia | The Waves | UK | 1 | 1931 | 1 | 1882 | 0 | 0 | 0 | 0 | 0 | 0 | 0 | 0 | 0 | 0 | 0 | 0 | 0 | 0 | 0 | 1 |
| Wouk | Herman | The Caine Mutiny | USA | 0 | 1951 | 2 | 1915 | 0 | 0 | 0 | 0 | 1 | 0 | 0 | 0 | 0 | 0 | 0 | 0 | 0 | 0 | 0 | 0 |
| Wright | Stephen | Going Native | USA | 0 | 1994 | 2 | 1955 | 0 | 0 | 0 | 0 | 0 | 0 | 0 | 0 | 0 | 0 | 0 | 0 | 0 | 0 | 0 | 0 |
| Wroblewski | David | The Story of Edgar Sawtelle | USA | 0 | 2008 | 2 | 1959 | 0 | 0 | 0 | 0 | 0 | 0 | 0 | 0 | 0 | 0 | 0 | 0 | 0 | 0 | 0 | 0 |
| Wyndham | John | The Kraken Wakes | USA | 0 | 1953 | 2 | 1903 | 0 | 0 | 0 | 0 | 0 | 0 | 0 | 0 | 0 | 0 | 0 | 0 | 0 | 0 | 0 | 0 |
| Wyndham | John | The Day of The Triffids | USA | 0 | 1951 | 2 | 1903 | 0 | 0 | 0 | 0 | 0 | 0 | 0 | 0 | 0 | 0 | 0 | 0 | 0 | 0 | 0 | 0 |
| Xiaolong | Qiu | A loyal character dancer | CHINA | 0 | 2002 | 4 | 1953 | 0 | 0 | 0 | 0 | 0 | 0 | 0 | 0 | 0 | 0 | 0 | 0 | 0 | 0 | 0 | 0 |
| Xiaolong | Qiu | When red is black | CHINA | 0 | 2004 | 4 | 1953 | 0 | 0 | 0 | 0 | 0 | 0 | 0 | 0 | 0 | 0 | 0 | 0 | 0 | 0 | 0 | 0 |
| Xiaolong | Qiu | Years of red dust | CHINA | 0 | 2008 | 4 | 1953 | 0 | 0 | 0 | 0 | 0 | 0 | 0 | 0 | 0 | 0 | 0 | 0 | 0 | 0 | 0 | 0 |
| Xilonen | Aura | The gringo champion | MEXICO | 1 | 2015 | 3 | 1995 | 1 | 0 | 0 | 0 | 0 | 0 | 0 | 0 | 0 | 0 | 0 | 0 | 0 | 0 | 0 | 0 |
| Xingjian | Gao | Soul mountain | CHINA | 0 | 1989 | 4 | 1940 | 1 | 1 | 0 | 0 | 0 | 0 | 0 | 0 | 0 | 0 | 0 | 0 | 0 | 0 | 0 | 0 |
| Xingjian | Gao | Buying a fishing rod for my grandfather | CHINA | 0 | 1986 | 4 | 1940 | 1 | 1 | 0 | 0 | 0 | 0 | 0 | 0 | 0 | 0 | 0 | 0 | 0 | 0 | 0 | 0 |
| Xueqin | Cao | Dream of the red chamber | CHINA | 0 | 1792 | 4 | 1715 | 1 | 0 | 0 | 0 | 0 | 0 | 0 | 0 | 0 | 0 | 0 | 0 | 0 | 0 | 0 | 0 |
| Yan | Mo | Red sorghum | CHINA | 0 | 1986 | 4 | 1955 | 1 | 1 | 0 | 0 | 0 | 0 | 0 | 0 | 0 | 0 | 0 | 0 | 0 | 0 | 0 | 0 |
| Yan | Mo | Big breasts and wide hips | CHINA | 0 | 1996 | 4 | 1955 | 1 | 1 | 0 | 0 | 0 | 0 | 0 | 0 | 0 | 0 | 0 | 0 | 0 | 0 | 0 | 0 |
| Yan | Mo | Life and death are wearing me out | CHINA | 0 | 2006 | 4 | 1955 | 1 | 1 | 0 | 0 | 0 | 0 | 0 | 0 | 0 | 0 | 0 | 0 | 0 | 0 | 0 | 0 |
| Yanagihara | Hanya | A little life | USA | 1 | 2015 | 2 | 1974 | 0 | 0 | 0 | 0 | 0 | 0 | 0 | 0 | 0 | 0 | 0 | 0 | 1 | 0 | 0 | 1 |
| Yanagihara | Hanya | The People in the Trees | USA | 1 | 2013 | 2 | 1974 | 0 | 0 | 0 | 0 | 0 | 0 | 0 | 0 | 0 | 0 | 0 | 0 | 0 | 0 | 0 | 0 |
| Yates | Richard | Easter parade | USA | 0 | 1976 | 2 | 1926 | 0 | 0 | 0 | 0 | 0 | 0 | 0 | 0 | 0 | 0 | 0 | 0 | 0 | 0 | 0 | 0 |
| Yates | Richard | Revolutionary road | USA | 0 | 1961 | 2 | 1926 | 0 | 0 | 0 | 0 | 0 | 0 | 0 | 0 | 0 | 0 | 0 | 0 | 0 | 0 | 0 | 0 |
| Yates | Dornford | Adele and Co | UK | 0 | 1931 | 1 | 1885 | 0 | 0 | 0 | 0 | 0 | 0 | 0 | 0 | 0 | 0 | 0 | 0 | 0 | 0 | 0 | 0 |
| Yates | Dornford | Blood Royal | UK | 0 | 1929 | 1 | 1885 | 0 | 0 | 0 | 0 | 0 | 0 | 0 | 0 | 0 | 0 | 0 | 0 | 0 | 0 | 0 | 0 |
| Yates | Dornford | She Fell Among Thieves | UK | 0 | 1935 | 1 | 1885 | 0 | 0 | 0 | 0 | 0 | 0 | 0 | 0 | 0 | 0 | 0 | 0 | 0 | 0 | 0 | 0 |
| Yates | Christopher | Grist Mill Road | UK | 0 | 2018 | 1 | 1972 | 0 | 0 | 0 | 0 | 0 | 0 | 0 | 0 | 0 | 0 | 0 | 0 | 0 | 0 | 0 | 0 |
| Yefremov | Ivan | Andromeda Nebula | RUSSIA | 0 | 1957 | 1 | 1908 | 1 | 0 | 0 | 0 | 0 | 0 | 0 | 0 | 0 | 0 | 0 | 0 | 0 | 0 | 0 | 0 |
| Yehoshua | Abraham | A woman in Jerusalem | ISRAEL | 0 | 2004 | 4 | 1936 | 1 | 0 | 0 | 0 | 0 | 0 | 0 | 0 | 0 | 0 | 0 | 0 | 0 | 0 | 0 | 0 |
| Yi | Nu Nu | Smile As They Bow | MYANMAR | 1 | 1994 | 4 | 1957 | 1 | 0 | 0 | 0 | 0 | 0 | 0 | 0 | 0 | 0 | 0 | 0 | 0 | 0 | 0 | 0 |
| Yong | Jin | The Legend of the Condor Heroes | CHINA | 0 | 1957 | 4 | 1924 | 1 | 0 | 0 | 0 | 0 | 0 | 0 | 0 | 0 | 0 | 0 | 0 | 0 | 0 | 0 | 0 |
| Yoshida | Shūichi | Parade | JAPAN | 0 | 2002 | 4 | 1968 | 1 | 0 | 0 | 0 | 0 | 0 | 0 | 0 | 0 | 0 | 0 | 0 | 0 | 0 | 0 | 0 |
| Yoshimoto | Banana | Kitchen | JAPAN | 1 | 1988 | 4 | 1964 | 1 | 0 | 0 | 0 | 0 | 0 | 0 | 0 | 0 | 0 | 0 | 0 | 0 | 0 | 0 | 0 |
| Yoshimoto | Banana | Moshi moshi | JAPAN | 1 | 2010 | 4 | 1964 | 1 | 0 | 0 | 0 | 0 | 0 | 0 | 0 | 0 | 0 | 0 | 0 | 0 | 0 | 0 | 0 |
| Young | William | The Shack | CANADA | 0 | 2007 | 2 | 1955 | 0 | 0 | 0 | 0 | 0 | 0 | 0 | 0 | 0 | 0 | 0 | 0 | 0 | 0 | 0 | 0 |
| Young | Lani Wendt | Telesa - The Covenant Keeper | SAMOA | 1 | 2011 | 6 | 1973 | 0 | 0 | 0 | 0 | 0 | 0 | 0 | 0 | 0 | 0 | 0 | 0 | 0 | 0 | 0 | 0 |
| Yourcenar | Marguerite | Memoirs of Hadrian | FRANCE | 1 | 1951 | 1 | 1903 | 1 | 0 | 0 | 0 | 0 | 0 | 0 | 0 | 0 | 0 | 0 | 0 | 0 | 0 | 0 | 0 |
| Yousafzai | Malala | I am Malala | PAKISTAN | 1 | 2013 | 4 | 1997 | 0 | 0 | 0 | 0 | 0 | 0 | 0 | 0 | 0 | 0 | 0 | 0 | 0 | 0 | 0 | 0 |

| **SURNAME** | **NAME** | **TITLE** | **NATIONALITY** | **GENDER** | **PUB_YEAR** | **CONTINENT** | **DATE OF BIRTH** | **TRANSLATED** | **NOBEL** | **NEUSTADT** | **BOOKER** | **PULITZER** | **NEBULA** | **CAINE** | **FRANZ KAFKA** | **NBA** | **BBA** | **WOMEN'S PRIZE FOR FICTION** | **NEW YORK TIMES** | **THE GUARDIAN** | **THE NEW YORKER** | **BBC** | **ATLANTIC** |
| --- | --- | --- | --- | --- | --- | --- | --- | --- | --- | --- | --- | --- | --- | --- | --- | --- | --- | --- | --- | --- | --- | --- | --- |
| Zafón | Carlos Ruiz | The shadow of the wind | SPAIN | 0 | 2001 | 1 | 1964 | 1 | 0 | 0 | 0 | 0 | 0 | 0 | 0 | 0 | 0 | 0 | 0 | 0 | 0 | 0 | 0 |
| Zafón | Carlos Ruiz | Marina | SPAIN | 0 | 1999 | 1 | 1964 | 1 | 0 | 0 | 0 | 0 | 0 | 0 | 0 | 0 | 0 | 0 | 0 | 0 | 0 | 0 | 0 |
| Zafón | Carlos Ruiz | The angel's game | SPAIN | 0 | 2008 | 1 | 1964 | 1 | 0 | 0 | 0 | 0 | 0 | 0 | 0 | 0 | 0 | 0 | 0 | 0 | 0 | 0 | 0 |
| Zail | Suzy | The wrong boy | AUSTRALIA | 1 | 2012 | 6 | 1966 | 0 | 0 | 0 | 0 | 0 | 0 | 0 | 0 | 0 | 0 | 0 | 0 | 0 | 0 | 0 | 0 |
| Zambra | Alejandro | Bonsai | CHILE | 0 | 2006 | 3 | 1975 | 1 | 0 | 0 | 0 | 0 | 0 | 0 | 0 | 0 | 0 | 0 | 0 | 0 | 0 | 0 | 0 |
| Zambra | Alejandro | Ways of Going Home | CHILE | 0 | 2011 | 3 | 1975 | 1 | 0 | 0 | 0 | 0 | 0 | 0 | 0 | 0 | 0 | 0 | 0 | 0 | 0 | 0 | 0 |
| Zola | Émile | Nanà | FRANCE | 0 | 1880 | 1 | 1840 | 1 | 0 | 0 | 0 | 0 | 0 | 0 | 0 | 0 | 0 | 0 | 0 | 0 | 0 | 0 | 0 |
| Zola | Émile | Germinal | FRANCE | 0 | 1885 | 1 | 1840 | 1 | 0 | 0 | 0 | 0 | 0 | 0 | 0 | 0 | 0 | 0 | 0 | 0 | 0 | 0 | 0 |
| Zusak | Markus | The book thief | AUSTRALIA | 0 | 2005 | 6 | 1975 | 0 | 0 | 0 | 0 | 0 | 0 | 0 | 0 | 0 | 0 | 0 | 0 | 0 | 0 | 0 | 0 |
| Zusak | Markus | I am the messenger | AUSTRALIA | 0 | 2002 | 6 | 1975 | 0 | 0 | 0 | 0 | 0 | 0 | 0 | 0 | 0 | 0 | 0 | 0 | 0 | 0 | 0 | 0 |
| Zweig | Stefan | The world of yesterday | AUSTRIA | 0 | 1941 | 1 | 1881 | 1 | 0 | 0 | 0 | 0 | 0 | 0 | 0 | 0 | 0 | 0 | 0 | 0 | 0 | 0 | 0 |
