# Supplementary material for: How Male and Female Literary Authors Write About Affect Across Cultures and Over Historical Periods
Source: Affect Sci. 2023 Sep 5;4(4):770–80. doi: 10.1007/s42761-023-00219-9 (PMC10751284; doi:10.1007/s42761-023-00219-9)
Supplement: Supplementary file 3 — Supplementary file3 (DOCX 17 KB) [file 42761_2023_219_MOESM3_ESM.docx]

hypernyms

| **artifact** | **social groups** | **weapons** | **body** | **affect** | **food & beverages** | **numbers** | **time** | **location** | **clothes** | **plants** |
| --- | --- | --- | --- | --- | --- | --- | --- | --- | --- | --- |
| *artifact* | *social_group* | *weapon* | *body_part* | *feeling* | *food* | *number* | *time_period* | *location* | *clothing* | *plant* |
| *object* | *group* | *firearm* | *body_covering* | *emotion* | *fluid* | *measure* | *season* | *region* | *fabric* |  |
| *instrumentality* | *group_action* | *gun* | *bodily_process* | *morality* | *plant_product* | *definite_quantity* |  | *structure* | *protective_covering* |  |
| *device* | *social_event* | *projectile* | *body_waste* |  |  | *magnitude* |  | *facility* | *footwear* |  |
| *matter* | *organization* | *weaponry* | *facial_expression* |  |  |  |  | *geological_formation* | *strip* |  |
|  | *military_unit* | *shooting* |  |  |  |  |  | *direction* | *cloth_covering* |  |
|  | *person* |  |  |  |  |  |  | *street* | *needlework* |  |
|  | *adult* |  |  |  |  |  |  | *boundary* |  |  |
|  | *male* |  |  |  |  |  |  | *side* |  |  |
|  | *female* |  |  |  |  |  |  |  |  |  |
|  | *skilled_worker* |  |  |  |  |  |  |  |  |  |
|  | *worker* |  |  |  |  |  |  |  |  |  |
|  | *commissioned_military_officer* |  |  |  |  |  |  |  |  |  |
|  | *serviceman* |  |  |  |  |  |  |  |  |  |
|  | *name* |  |  |  |  |  |  |  |  |  |
|  | *legal_status* |  |  |  |  |  |  |  |  |  |
|  | *work* |  |  |  |  |  |  |  |  |  |

Page 1
