# Supplementary material for: How Male and Female Literary Authors Write About Affect Across Cultures and Over Historical Periods
Source: Affect Sci. 2023 Sep 5;4(4):770–80. doi: 10.1007/s42761-023-00219-9 (PMC10751284; doi:10.1007/s42761-023-00219-9)
Supplement: Supplementary file 4 — Supplementary file4 (DOCX 16 KB) [file 42761_2023_219_MOESM4_ESM.docx]

| **Benchmarks of word embeddings** | | | | |
| --- | --- | --- | --- | --- |
| **Test** | **Reference** | **Correlation Coefficient** | **Accuracy in %** | **Coverage in %** |
| **RG-65** | *Rubenstein and Goodenough, 1965* | 0.871 |  | 100.0 |
| **WordSimilarity-353** | *Finkelstein et al., 2001* | 0.626 |  | 98.6 |
| **MEN dataset** | *Bruni et al., 2014* | 0.754 |  | 100.0 |
| **TOEFL Synonym Questions** | *Landauer and Dumais, 1997* |  | 85.9 | 88.8 |
| **BLIND eng** | *Lenci et al., 2013* | 0.712 |  | 100.0 |
| **McRAE** | *McRae et al., 2005* | 0.485 |  | 98.7 |
| **VINSON** | *Vinson & Vigliocco, 2008* | 0.582 |  | 99.6 |
| **SIMLEX-999** | *Reichart & Korhonen, 2015* | 0.451 |  | 99.9 |
| **MTURK-771** | *Halawi et al., 2012* | 0.647 |  | 99.7 |
| **WORD NORMS** | *Buchanan et al., 2013* | 0.379 |  | 98.8 |
|  |  |  |  |  |
|  |  |  |  |  |
|  |  |  |  |  |
|  |  |  |  |  |
| **REFERENCES** | | | | |
| *Rubenstein, H., & Goodenough, J. B. (1965). Contextual correlates of synonymy. Communications of the ACM, 8(10), 627-633.* | | | | |
| *Finkelstein, L., Gabrilovich, E., Matias, Y., Rivlin, E., Solan, Z., Wolfman, G., & Ruppin, E. (2001, April). Placing search in context: The concept revisited. In Proceedings of the 10th international conference on World Wide Web (pp. 406-414).* | | | | |
| *Bruni, E., Tran, N. K., & Baroni, M. (2014). Multimodal distributional semantics. Journal of artificial intelligence research, 49, 1-47.* | | | | |
| *Landauer, T. K., & Dumais, S. T. (1997). A solution to Plato's problem: The latent semantic analysis theory of acquisition, induction, and representation of knowledge. Psychological review, 104(2), 211.* | | | | |
| Lenci, A., Baroni, M., Cazzolli, G., & Marotta, G. (2013). BLIND: A set of semantic feature norms from the congenitally blind. Behavior research methods, 45(4), 1218-1233. | | | | |
| *McRae, K., Cree, G. S., Seidenberg, M. S., & McNorgan, C. (2005). Semantic feature production norms for a large set of living and nonliving things. Behavior research methods, 37(4), 547-559.* | | | | |
| *Vinson, D. P., & Vigliocco, G. (2008). Semantic feature production norms for a large set of objects and events. Behavior Research Methods, 40(1), 183-190.* | | | | |
| *Hill, F., Reichart, R., & Korhonen, A. (2015). Simlex-999: Evaluating semantic models with (genuine) similarity estimation. Computational Linguistics, 41(4), 665-695.* | | | | |
| *Halawi, G., Dror, G., Gabrilovich, E., & Koren, Y. (2012, August). Large-scale learning of word relatedness with constraints. In Proceedings of the 18th ACM SIGKDD international conference on Knowledge discovery and data mining (pp. 1406-1414).* | | | | |
| *Buchanan, E. M., Valentine, K. D., & Maxwell, N. P. (2019). English semantic feature production norms: An extended database of 4436 concepts. Behavior Research Methods, 51(4), 1849-1863.* | | | | |

**Table S4. Benchmarks of word embeddings.**
