# Supplementary material for: How Male and Female Literary Authors Write About Affect Across Cultures and Over Historical Periods
Source: Affect Sci. 2023 Sep 5;4(4):770–80. doi: 10.1007/s42761-023-00219-9 (PMC10751284; doi:10.1007/s42761-023-00219-9)
Supplement: Supplementary file 5 — Supplementary file5 (DOCX 14 KB) [file 42761_2023_219_MOESM5_ESM.docx]

| **Authors** | **Reference** |
| --- | --- |
| Bamler et al. (2017) | Bamler, R., & Mandt, S. (2017). Dynamic word embeddings. In International conference on Machine learning (pp. 380-389). PMLR. |
| Banks (2004) | Banks, M. A., "Semantic Changes in Present-Day English (PDE)" (2004). McCabe Thesis Collection. Paper 25. |
| Boukhaled et al. (2019) | Boukhaled, M., Fagard, B., & Poibeau, T. (2019). Modelling the semantic change dynamics using diachronic word embedding. In 11th International Conference on Agents and Artificial Intelligence (NLPinAI Special Session). |
| Curzane (2014) | Curzane A. (2014). What makes a word "real"? TED Conferences. https://www.ted.com/talks/anne_curzan_what_makes_a_word_real |
| Delpech (2018) | Delpech, J. (2018). Unsupervised detection of diachronic word sense evolution. ArXiv, abs/1805.11295. |
| Duan et al. (2021) | Duan, Y., Jatowt, A., Yoshikawa, M., Liu, X., Matono, A. (2021). Diachronic Linguistic Periodization of Temporal Document Collections for Discovering Evolutionary Word Semantics. In: Ke, HR., Lee, C.S., Sugiyama, K. (eds) Towards Open and Trustworthy Digital Societies. ICADL 2021. Lecture Notes in Computer Science, vol 13133. Springer, Cham. https://doi.org/10.1007/978-3-030-91669-5_1 |
| Finegan (2014) | Finegan, E. (2014). Language: Its structure and use. Cengage Learning. |
| Hamilton et al. (2016) | Hamilton, W.L., Leskovec, J., & Jurafsky, D. (2016). Diachronic Word Embeddings Reveal Statistical Laws of Semantic Change. ArXiv, abs/1605.09096. |
| Hamilton et al. (2016) | Hamilton, W. L., Leskovec, J., & Jurafsky, D. (2016). Cultural shift or linguistic drift? comparing two computational measures of semantic change. In Proceedings of the Conference on Empirical Methods in Natural Language Processing. Conference on Empirical Methods in Natural Language Processing (Vol. 2016, p. 2116). NIH Public Access. |
| Jones (2019) | Jones, P. A. (2017). The Accidental Dictionary: The Remarkable Twists and Turns of English Words. Pegasus Books. |
| Kulkarni et al. (2015) | Kulkarni, V., Al-Rfou, R., Perozzi, B., & Skiena, S. (2015). Statistically significant detection of linguistic change. In Proceedings of the 24th international conference on world wide web (pp. 625-635). |
| McWhorter (2016) | McWhorter, J. (2016). 4 reasons to learn a new language. TED Conferences. https://www.ted.com/talks/john_mcwhorter_4_reasons_to_learn_a_new_language |
| Qiu et al. (2022) | Qiu, Wenjun & Xu, Yang. (2022). HistBERT: A Pre-trained Language Model for Diachronic Lexical Semantic Analysis. 10.13140/RG.2.2.14905.44649. |
| Tsakalidis et al. (2021) | Tsakalidis, A., Basile, P., Bazzi, M. et al. (2021). DUKweb, diachronic word representations from the UK Web Archive corpus. Sci Data 8, 269. https://doi.org/10.1038/s41597-021-01047-x |
| Vijayarani & Geetha (2020) | Vijayarani, J., Geetha, T.V. (2020). Knowledge-enhanced temporal word embedding for diachronic semantic change estimation. Soft Comput 24, 12901–12918. https://doi.org/10.1007/s00500-020-04714-0 |

**Table S5. References for the selection of diachronic words.**
