# Supplementary material for: How Male and Female Literary Authors Write About Affect Across Cultures and Over Historical Periods
Source: Affect Sci. 2023 Sep 5;4(4):770–80. doi: 10.1007/s42761-023-00219-9 (PMC10751284; doi:10.1007/s42761-023-00219-9)
Supplement: Supplementary file 6 — Supplementary file6 (DOCX 2757 KB) [file 42761_2023_219_MOESM6_ESM.docx]

**How male and female literary authors write about affect**

**across cultures and over historical periods**

Giada Lettieri^1,2^*, Giacomo Handjaras^2^*, Erika Bucci^2^, Pietro Pietrini^3^ and Luca Cecchetti^2^

^1^ Crossmodal Perception and Plasticity Laboratory, Institute of Research in Psychology & Institute of Neuroscience, Université Catholique de Louvain, Louvain-la-Neuve, Belgium

^2^ Social and Affective Neuroscience Group, MoMiLab, IMT School for Advanced Studies Lucca, Lucca, Italy

^3^ Molecular Mind Laboratory, MoMiLab, IMT School for Advanced Studies Lucca, Lucca, Italy

* denotes equal contribution

**This PDF file includes:**

Supplementary Text

Figs. S1 to S8

Tables S1 to S5

References

**Supplementary Method**

*Composition of the corpus*

For the selection of books, we started from the narratives recognized as being exemplary or noteworthy and a classic. We based our selection on lists of classic books redacted by prominent literary scholars (Moretti, 1999), writers, and editors (e.g., Robert B. Downs, Penguin Random House). Indeed, books that are considered to have cultural importance and are well-written are commonly acknowledged in lists of classic narratives, even though there may be some discrepancy in the definition of a classic within genres or literary movements. Moreover, we added authors that received appreciation for their work through international literary awards (n=339, ~25%), such as the *Nobel* prize in literature (n=72, ~5% of all authors). We also included authors awarded or shortlisted with well-renowned country-specific literary prizes, specifically the *International Booker Prize* for works published in English in the United Kingdom and Ireland, the *Pulitzer Prize* for fiction produced by an American author, the *Franz Kafka Prize*, the *Nebula Award* for works of science fiction published in the USA, and the *Caine Prize* for short stories by African writers. Regarding our selection of more recent books (from 2000 onward), we also employed literature reviews published in the most prominent international newspapers in terms of circulation: the *Best books through time* lists in *The New York Times*, *Best fiction* drawn up every year in *The Guardian*, the *Best books* and the *Books we loved* lists drafted every year by *The New Yorker*, the annual *10 best books* and the *Ten books to read* by *BBC*, and the *Best book I read this year* and *Books we loved* year-end lists by *The Atlantic*. Of note, we also explored the *List of writers by country* on Wikipedia for each country in the world, and lists related to writers coming from specific areas (e.g., *The notable African books* by *Brittle Paper*). We also reviewed lists on the Wikipedia encyclopedia of best-selling fiction authors and included books that sold millions of copies consistently around the entire world.

*Text preprocessing*

Starting from the digital version of the books, we manually checked each of them and removed all the information and paragraphs that were not related to the narrative *per se*. Specifically, we deleted text coming from the front and back cover, translator’s note, notes, prefaces, and afterwords from the editor or other persons who were not the authors, acknowledgments, appendices, and everything else that was not first intended to be written by the writers and did not appear in the original version of the books. Preprocessed books were aggregated by author to obtain 1,365 documents. The text in each document was first lowercase converted. Then, we defined a set of 2-grams across all the documents by manually inspecting the ones with a relatively high frequency (>100 occurrences). In addition to the aforementioned procedure, we also included a smaller set of 2-grams describing specific affective states of particular interest (e.g., *self-confident*, *absent-minded*). A complete list of all the considered ~1,300 2-gram is reported in the *SaneNLP toolbox* [(https://github.com/giacomohandjaras/SaneNLP_toolbox](https://github.com/giacomohandjaras/SaneNLP_toolbox)). Subsequently, we removed any reference to Arabic numerals and any form of punctuation from the raw text. Lastly, the text was tokenized to obtain the raw count of occurrences of words, selecting those present at least 40 times across all the 1,356 documents (Michel et al., 2011), defining a final vocabulary of ~70,000 words.
To measure the impact of sex in word frequency across authors, we first selected a subset of 25,040 words expressed in at least 10% of male or female writers. This procedure restricted the number of statistical tests to be performed, excluding relatively rare words. A two-dimensional authors by words matrix reporting word frequencies was generated.

*Modeling word frequencies through Heaps’ and Zipf’s Laws*

Firstly, we measured the number of total words across authors (Fig. S1a), as well as the size of their lexicon (i.e., number of unique words). The Heaps’ Law was used (V=Kn^β^; where *V* is the number of unique words, n the total number of words, K and β the two parameters to estimate; Popescu, 2009) to uncover the non-linear association between the number of distinct words in a document and the document length (Fig. S1b). Parameters K and β were calculated in male and female authors separately by fitting the Heaps’ Law with the linear least-squares method and by estimating their standard errors through a bootstrapping procedure (n=100).

Secondly, we computed Zipf’s Law (F = *β*/x^α^; where *F* is the frequency of the words in an author’s text, *x* their ranks, *β* a scaling factor, and *α* the exponential factor; Zipf, 1949). We estimated the frequency of each term per millions of words, as well as its ranking position in the lexicon of each author. Then, the *β* and *α* coefficients were calculated using the linear least-squares method. Sex differences were assessed through a Wilcoxon rank-sum test (Fig. S1c-e).
Lastly, we evaluated the vocabulary size by randomly bootstrapping (n=100) sets of 10k consecutive words (i.e., approximately the length of the shortest book in our corpus) and measuring the average number of unique words across samples (Fig. S1f). Sex differences in vocabulary size were assessed through a Wilcoxon rank-sum test.

*Sex differences over time*

To measure the temporal characteristics of sex differences in the use of words, we first defined a set of overlapping sliding temporal windows (i.e., historical periods). The window width was set to a decile of the historical-period distribution resulting in a non-linear sampling of the literature timeline and the overlap between neighboring windows was 20%. This procedure allowed for fair comparisons between timepoints as the calculation of the effect was based on the same number of degrees of freedom (i.e., authors).

*Similarity between our corpus and Google English Fiction 2020*

Aiming to validate word frequencies and trends found in the present work, we compared our corpus with the 1-gram corpus of English Fiction from Google (version 20200217). The Google English Fiction corpus mainly includes texts identified as fiction published in the English language until 2020. Starting from the Google corpus, we discarded all tokens which included numbers and ended with the part-of-speech tags. For each token, we generated a vocabulary by calculating the raw count of occurrences across time. We then applied our text preprocessing pipeline to convert tokens in lowercase and to remove punctuation marks. Since this procedure could generate multiple tokens (e.g., *john’s* would split into *john* and *s*), we updated the count of occurrences of the unique tokens. These were used to assess the similarity in word frequencies and trends between the two corpora (Fig. S2). To do this, we identified the subset of words present in both corpora and correlated (Spearman’s ρ) the average word frequency across years in the Google dataset with our corpus, as well as with male and female authors separately.

We also evaluated the coherence of temporal trends between the two corpora. For each term, we applied the set of overlapping sliding temporal windows to the Google dataset. We then scaled each time series in Google and our corpus to its maximum value. We did this as we were interested in estimating the linear trends irrespectively from the frequency of each word, and because term frequency varies by several orders of magnitude across words. Linear trends were estimated in Google English Fiction 2020 as well as in our corpus. The same analysis was performed considering only female authors and a randomly selected subsample of males, to match sample size. Spearman’s correlation measured the association between trends found in the Google corpus and those revealed in our dataset (Fig. S2b-c). To test whether linear trends of words from Google were more similar to those found in male rather than female authors, we performed a permutation test on the difference of the ρ coefficients by shuffling the sex variable (n=10,000). We repeated the same analysis for words showing significant sex differences and for the full set of 25,040 terms (Fig. S2d). To visualize the extent to which trends in Google books are biased toward male or female authors, we first sorted the words based on the absolute t-score of the sex effect. For each term, we then measured the absolute value of the difference between the estimate of the historical trend in Google and those obtained from the male and female corpora. We reported this difference in Fig. S2e, after applying a moving average filter to improve readability (windows size=5000 words, 5% of overlap across windows).

*Revealing sex differences in literature using ranks*

To rule out the possibility that results of the GLM *frequency* ~ *intercept* + *sex* + *historical period* + (*sex * historical period*) + *translation* + *continent* were affected by the long-tail distribution of word frequencies, we ran the same analysis using word ranks as the dependent variable. Specifically, for each author, terms were sorted so that words occurring more frequently had higher ranks. Then, for each term and author, we log-transformed the ranks and scaled them from 1 (i.e., the most frequent word for that author) to 0 (i.e., a word that was never used by the author). For each of the words showing a significant *sex* or *sex* by *historical period* effect, we performed a partial F-test [formula 1 in the Main text], comparing the full model against a nested simplified version of it, in which these two effects are not modeled. Similar to the main analysis with frequencies, we rank transformed the *historical period*, and statistical significance was obtained through a non-parametric permutation test (n=10,000), shuffling in each iteration the variable *sex*. Results were corrected for multiple comparisons using the Bonferroni method (i.e., number of words with significant *sex* or *sex* by *historical period* effect).

*Revealing sex differences in literature using country of origin*

Following a suggestion from a Reviewer, we tested an alternative GLM model including the country of origin of authors, instead of the continent. The model we test is:

*word frequency = intercept + country of origin + sex + historical period + sex by historical period interaction + translation*

As in previous analyses, words showing a significant sex and/or sex by historical period interaction were identified through the partial F test and non parametric testing (n = 1,000 permutations).

*Mapping sex differences in time, place, and meaning for each word*

For each word showing significant *sex* or *sex* by *historical period* effects, we reported Cohen's *d* in each country of the world across three centuries (Fig. S6a), and estimated the temporal characteristics of raw frequencies (Fig. S6b; <https://www.sane-elab.eu/litemo/welcome.php>), similarly to Google Ngram Viewer (<https://books.google.com/ngrams>). In addition, we tested whether differences in word frequency between male and female authors were driven by shifts in meaning. Following the distributional hypothesis, which states that words that occur in the same contexts share similar semantics (Harris, 1954), and taking advantage of local embedding (Hamilton et al., 2016a), we explored the semantic nearest neighbors of each word (Fig. S6c). Firstly, we trained two word2vec embeddings, one for each sex, using the same parameters and procedures described in the main text (*Revealing sex differences in literature*). Secondly, we hyper-aligned the two sex-specific embeddings to the one obtained by considering all authors, using orthogonal Procrustes. This solution does not affect pairwise cosine-similarities within each semantic space and allows comparisons of word embeddings across multiple corpora (Hamilton et al., 2016b). Hence, for each one of the significant terms, we found the union of *k* nearest neighbors (*k*=25, as in Hamilton et al., 2016a) across the two sexes. We evaluated the sum of the cosine distances of the word of interest and all its neighbors for male and female authors separately and then computed the absolute difference between the two. Statistical significance was assessed by randomly sampling 10k words (out of the original ~25k) and performing the same analysis described above to generate a distribution of 10k effects. P-values were estimated by fitting a generalized Pareto distribution to the tail of the obtained distribution. Correction for multiple comparisons was carried out using the False Discovery Rate procedure (Benjamini & Hochberg, 1995; q<0.05, for Gaussian variables which were positively correlated or independent). Crucially, this method tested whether words showing a difference in frequency between the sexes were those demonstrating larger shifts in semantics as well.

*Analysis of diachronic words*

Together with shifts in meaning between the two sexes, we also explored the presence of diachronic words showing a semantic shift in time among our significant terms. Specifically, we identified 1,257 terms showing a semantic shift in time, including the top 10^th^ percentile of words in Hamilton et al., 2016b and from other 14 resources (see *SaneNLP toolbox* and Table S5). Then, we measured the overlap between our set of significant terms and the aggregated list of diachronic words. Moreover, since a shift in meaning could be accompanied by a change in the affective components of words (e.g., *awful*, *terrific*), we excluded diachronic words from the affective analysis.

*Measuring the relationship between affective lexicon and human development*

In addition, we investigated whether male and female authors write differently about emotions as a function of culture. To this aim, we first computed for each country the effect size (i.e., Cohen’s d) of sex differences in valence (Fig. S7a) and arousal (Fig. S7b) obtained from the formula [2] in the main manuscript. We then obtained the average score of the Human Development Index (HDI, https://hdr.undp.org/) - a composite index of life expectancy, gross income per capita, and level of education - for the countries with at least seven authors (i.e., the top quartile of the distribution) published between 1990 and 2020 (i.e., the years for which HDI is available). The HDI was then correlated (Spearman’s ρ) with valence and arousal scores averaged across authors regardless of their sex to test whether books from more developed countries are more (or less) positive (Fig. S7c) and/or arousing (Fig. S7d). Lastly, to evaluate whether sex differences in the affective lexicon are associated with societal progress, we correlated across countries the HDI and the Cohen’s d of the male *versus* female valence and arousal scores.

**Supplementary Results**

*Composition of the corpus*

To summarize the influence of the author sex, country of origin, language, and historical period on writing style, we map the distribution of these variables on a graph of writers’ similarity based on word frequency (Jockers, 2013; Fig. 1). In line with data on publishing records, in our corpus there is a significant relationship between author’s sex and country of origin (χ^2^_(1,1365)_ = 26.74; p < 0.0001), with male writers being more frequently European (Verboord, 2012; odds ratio [OR] = 0.664, CI95: 0.526 0.839, p<0.0001, Fisher’s Exact Test), and female writers Oceanian (Zwar et al., 2015; OR = 3.002, CI95: 1.677 5.374, p = 0.0002, Fisher’s Exact Test). As far as language is concerned, while the percentage of male authors translated into English is 39%, translated female authors represent 23% (OR = 0.470, CI95: 0.366 0.604, p<0.0001, Fisher’s Exact Test). Also, as in other reports (Underwood et al., 2018), the female-to-male proportion increases over time: the percentage of female authors included in our corpus active before 1900 is 23% (p < 0.0001, Binomial test), with this percentage reaching 50% in the last ten years (p = 0. 9111; Fig. 1b). Lastly, we observe a strong predominance of male authors in the assignment of the Nobel Prize (~83% were for male writers, χ^2^_(1,1365)_ = 12.55; p = 0.0004), whereas the most renowned literary awards are in agreement with the overall ratio of male and female writers (~65% of the winners of at least one prize were males, χ^2^_(1,1365)_ = 0.55; p = 0.4586).

*Heaps’ and Zipf’s Laws*

We estimate the average number of words written by male (186,624 ± 193,524) and female (171,515 ± 183,835) authors. The difference between the two groups is 15,109 terms (p = 0.046, Wilcoxon rank-sum test; Fig. S1a). However, this effect could reflect different factors in the history of literature, such as trends in publishing policy (e.g., releasing novels in smaller, sequential installments - as frequently done during the 19^th^ century -), or to the ratio of female authors changing across time. We also explore the lexicon size using Heaps’ law (Fig. S1b). This analysis estimates the free parameters K (mean±standard error; K_male_=36.6±5.9; K_female_=33.1±5.4) and β (β_male_=0.48±0.01; β_female_=0.48±0.01), as well as the adjusted-R^2^ (R^2^_male_= 0.80; R^2^_female_= 0.83). We observe no differences in the estimation of the parameters across sexes (p > 0.05).

We then model the non-linear relationship between the rank and the frequency of the words used (Zipf’s law; see Fig. 1c). This analysis estimates in each author the exponential parameters *α* (mean±standard error; *α*_male_=1.003 ± 0.001; *α*_female_=1.006 ± 0.002; Fig. S1d), the scaling factor *β* (*β*_male_=133699 ± 764; *β*_female_=140270 ± 921; Fig. S1e), as well as the adjusted-R^2^ (R^2^_male_= 0.99; R^2^_female_= 0.99). Of note, only the scaling factor *β* shows a significant sex effect (p < 0.0001).

Lastly, bootstrap analysis reveals that females have a smaller vocabulary size than males (mean ± standard deviation for 10k words; males: 2173 ± 293; females: 2098 ± 258; difference M-F: 75, p < 0.0001; Fig. S1f).

*Similarity between our corpus and Google English Fiction 2020*

Terms showing *sex* or *sex * historical period* significant effects are relatively frequent (e.g., from 5.4% average occurrence across authors for the term *the*, to 5.3*10^-5^% for *sparkly*), and their occurrence in our corpus is in line with the one reported in the largest available corpus of English Fiction from Google (ρ = 0.981; CI95: 0.975 0.984; p < 0.0001, Fig. S2a). In addition, we show that Google English Fiction 2020 historical trends of the 576 significant terms are more similar to trends present in male (ρ = 0.599, CI95: 0.534 0.657), as compared to those observed in female writers (ρ = 0.479, CI95: 0.407 0.543; sex effect: ρ = 0.120, p < 0.0001; Fig. S2c). Notably, similar results are observed when considering the initial pool of ~25,000 words: at least 20% of these terms show trends in Google English Fiction 2020 comparable to those of male authors (Fig. S2e). Thus, caution should be exerted when drawing cultural, linguistic, and social conclusions from trend analyses of large uncontrolled datasets (Pechenick et al., 2015; Lazer et al., 2021), as they might reflect the unequal representation of the sexes.

*Revealing sex differences in literature using ranks*

By using word ranks instead of raw frequencies, 564 out of 576 words (~98%) show significant *sex* or *sex* * *historical period* effect (p < 0.05, Bonferroni corrected). Terms that do not replicate their effect in this control analysis are: *t*, *come*, *eyes*, *look*, *help*, *glad*, *position*, *shoes*, *leading*, *quarter*, *porn*, *hesitatingly*.

*Revealing sex differences in literature using country of origin*

We show that 525 terms reach statistical significance when modelling country of origin. Of these, the 84% (485) overlaps with the 576 words identified by modelling continent instead of country. The 91 words specific to the modelling of continent are:

*"men", "dear", "wall", "order", "parents", "pointed", "shirt", "ladies", "spend", "distant", "wrapped", "fucking", "local", "fuck", "gentlemen", "silk", "evidence", "down stairs", "upper", "staff", "grateful", "staying", "team", "emerged", "shocked", "wheel", "nation", "tray", "pregnant", "recent", "clerk", "goodness", "inspector", "shy", "blade", "lessons", "tobacco", "roar", "mister", "based", "complex", "investigation", "indicated", "player", "scarf", "aspect", "liquor", "proceed", "cells", "examination", "policemen", "butt", "explosion", "areas", "recorded", "wrap", "posts", "mother in law", "warehouse", "madman", "corporal", "dolls", "data", "politicians", "signals", "clerks", "vicinity", "pissed", "sentry", "regulations", "forearm", "captains", "controls", "avenues", "fucked up", "phenomena", "fart", "cartridge", "manufactured", "cartridges", "holster", "porn", "gandhi", "testicles", "masonry", "explosives", "shooter", "rusting", "shitting", "converging", "urinating".*

The 40 words specific to the modelling of country are:

*"hands", "voice", "far", "cold", "case", "ground", "minutes", "couple", "angry", "group", "happiness", "attempt", "valley", "manage", "series", "remembering", "washing", "jealous", "united states", "empire", "organization", "profit", "located", "commercial", "teasing", "sobbed", "ached", "ribbons", "bearings", "sector", "kittens", "birthdays", "bureaucratic", "outgrown", "leverage", "chintz", "darning", "hairbrush", "denizens", "raspberries".*

Of note, all words found in one model and not the other, reach statistical significance when using the more lenient threshold of p < 0.001 not corrected for multiple comparisons. Overall, the modelling of country instead of continent results in a relatively smaller set of words showing a sex or sex by historical period effect. However, the fact that the majority of terms is significant in both models suggests that the ratio between the number of observations (1,365 modelling continent versus 1,295 modelling country) and of predictors (10 versus 67) may play a crucial role in determining the differences between the original approach and the one described here.

*Analysis of diachronic words*

Among the 576 significant words, only ~4% have changed their meaning over time (e.g., *terrific*). To test semantic shifts between male and female writers, we quantify rearrangements of neighbor terms in word-embedding as a function of the author sex (Fig. S6c). None of the 576 terms show a substantial semantic shift between the sexes (p > 0.05, corrected for multiple comparisons), indicating that while the frequency of terms changed over time, male and female authors attributed the same meaning to these words across the last three centuries.

*Measuring the relationship between affective lexicon and human development*

The mapping of sex differences in the affective lexicon across cultures shows that while in some countries women’s writings are more positive and in others the opposite is true (Fig. S7a), men’s lexicon is more arousing in all the explored cultures (Fig. S7b). When testing the association between societal progress and affective characteristics of writings, we observe that HDI relates positively to valence (Fig. 5c; ρ = 0.433, p = 0.045, CI95: 0.094 0.697) and negatively to arousal (Fig. 5d; ρ = -0.439, p = 0.042, CI95: -0.756 -0.017) across 22 countries, meaning that authors native to regions characterized by higher HDI (e.g., Germany, Australia) write more positive and less arousing narratives. Lastly, there are no significant associations between the degree of societal progress and sex differences in valence (ρ = 0.121, p = 0.589, CI95: -0.429 0.532) or arousal (ρ = -0.286, p = 0.196, CI95: -0.566 0.159).
We demonstrate that people from more developed societies write on average more positive and less arousing books. This tendency, present in both men and women, is in line with a cross-national study on life satisfaction showing that the human development index explains 10% of the variance in reports of subjective well-being (Bonini, 2008).


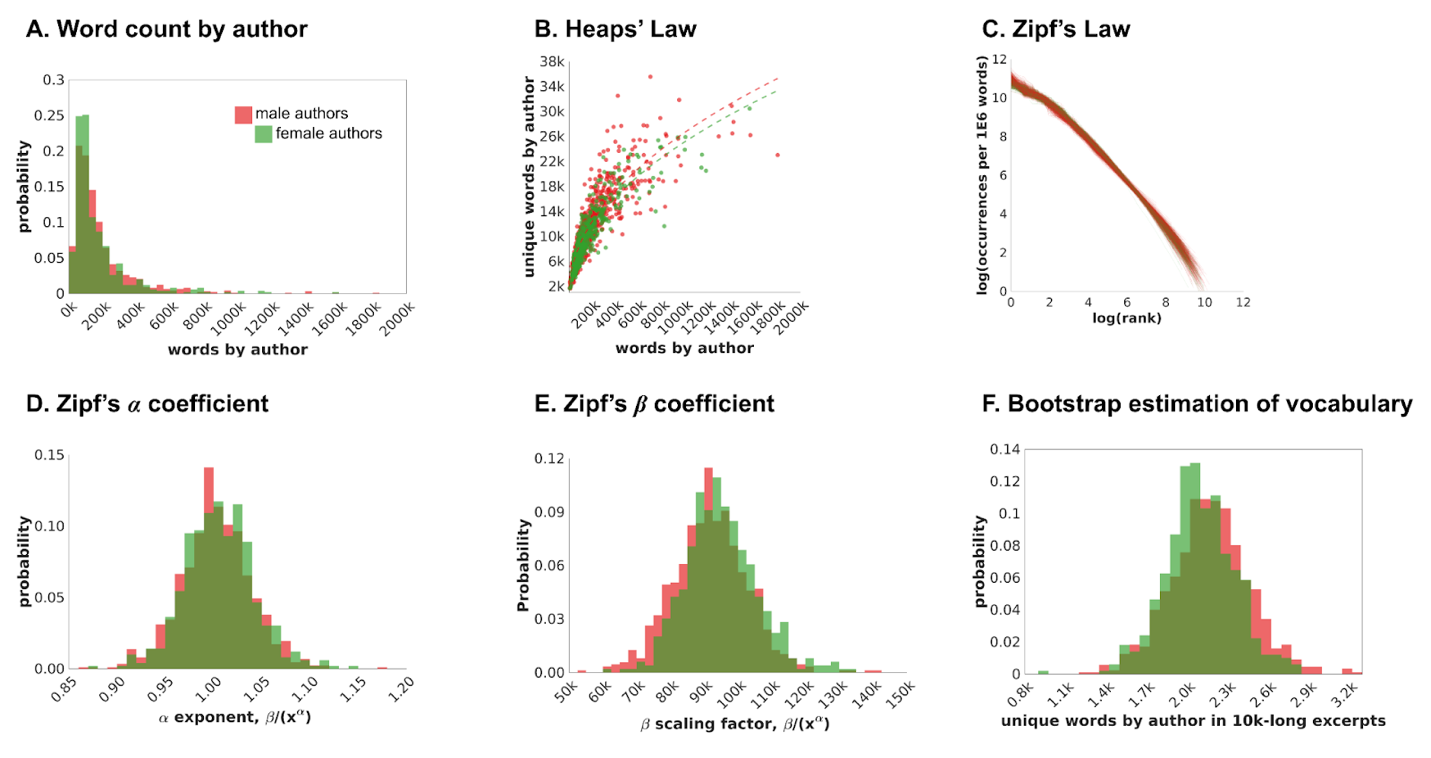


**Fig. S1.** In panel a, we report the total number of words in male and female authors, separately. In panel b, we model the relationship between the total number of words in a text and the lexicon size using Heaps’ law. In panel c, we represent the non-linear relationship between the rank and the frequency of the words using Zipf’s law. In panels d and e, we test sex differences for the exponential parameters *α* and the scaling factor *β* estimated in each author from Zipf’s law. In panel f, we report the results of a bootstrapping procedure to estimate sex differences in lexicon size.

**
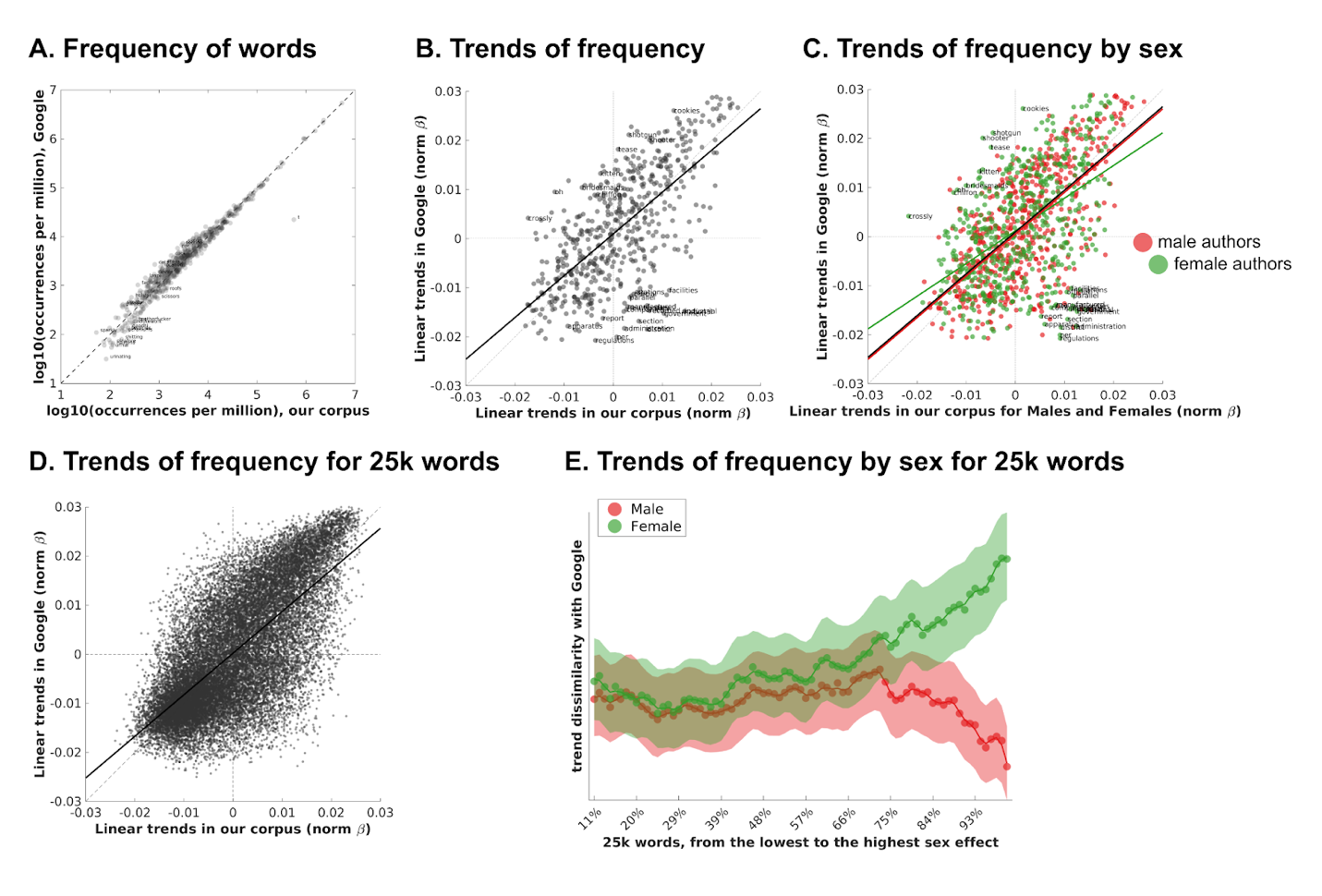
**

**Fig. S2.** We compare our estimate of the frequency of words with the one proposed in the Google Fiction 2020 dataset (a). In panels b and c, we assess the coherence of temporal trends for our significant words between the two corpora (b) and separately for male and female authors (c). Also, we explore the similarity of linear trends between Google and our corpus across all 25,040 words (d). In panel e, after sorting words according to the statistical magnitude of the sex effect (i.e., t-score), we measure the absolute value of the difference between the estimation of the trend in Google and the one from male and female authors separately. Shaded areas represent 95% confidence intervals of the estimation across authors. Results show that at least 20% of terms exhibit trends in Google English Fiction 2020 more similar to male as compared to female authors.

**
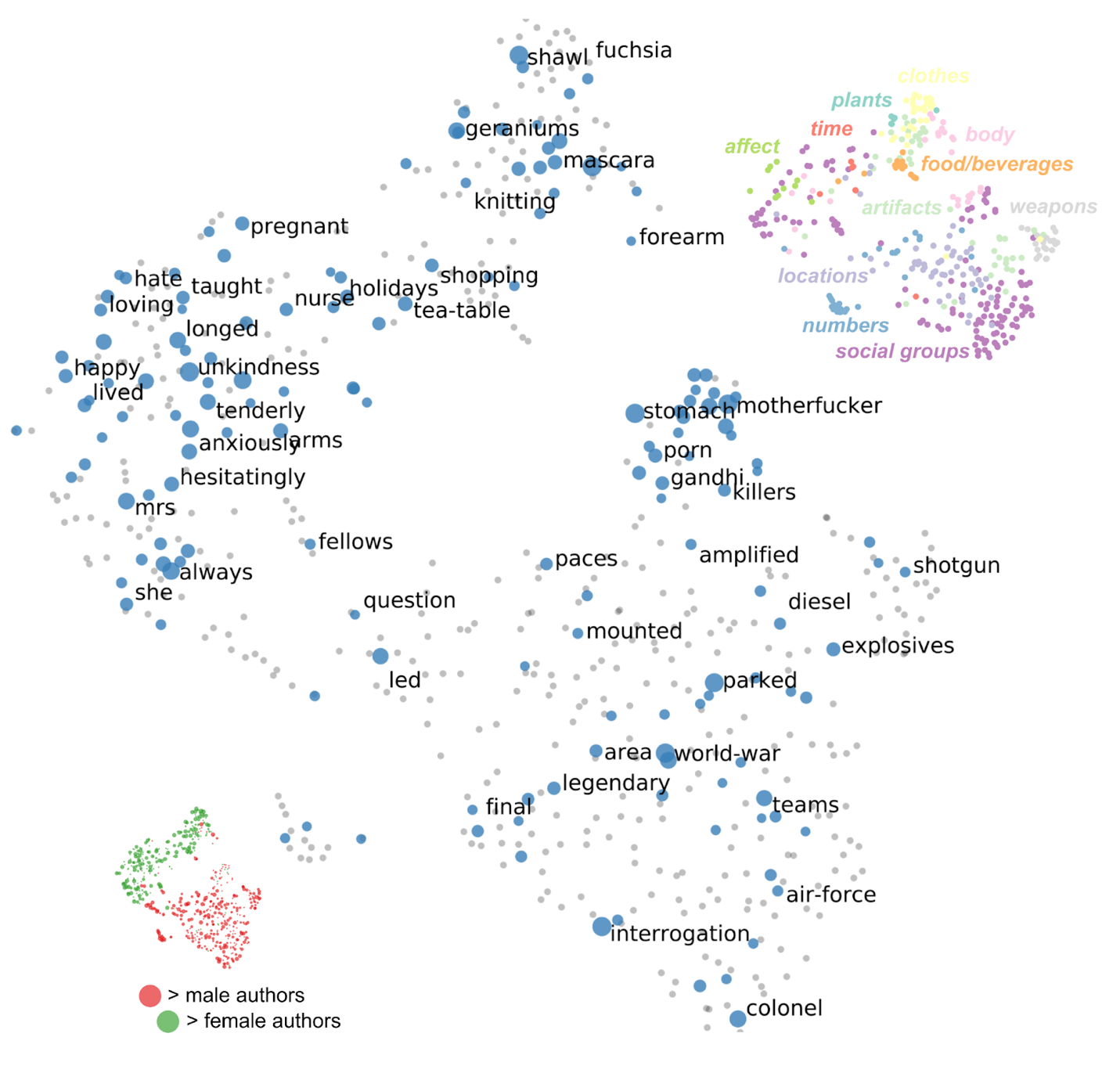
**

**Fig. S3.** The figure reports the words which retained significant *sex* by *historical period* effect in a 2D map obtained through t-SNE applied to word2vec word embeddings. Terms were grouped according to the membership to one out of eleven semantic domains defined using Wordnet. Each dot represents a word.


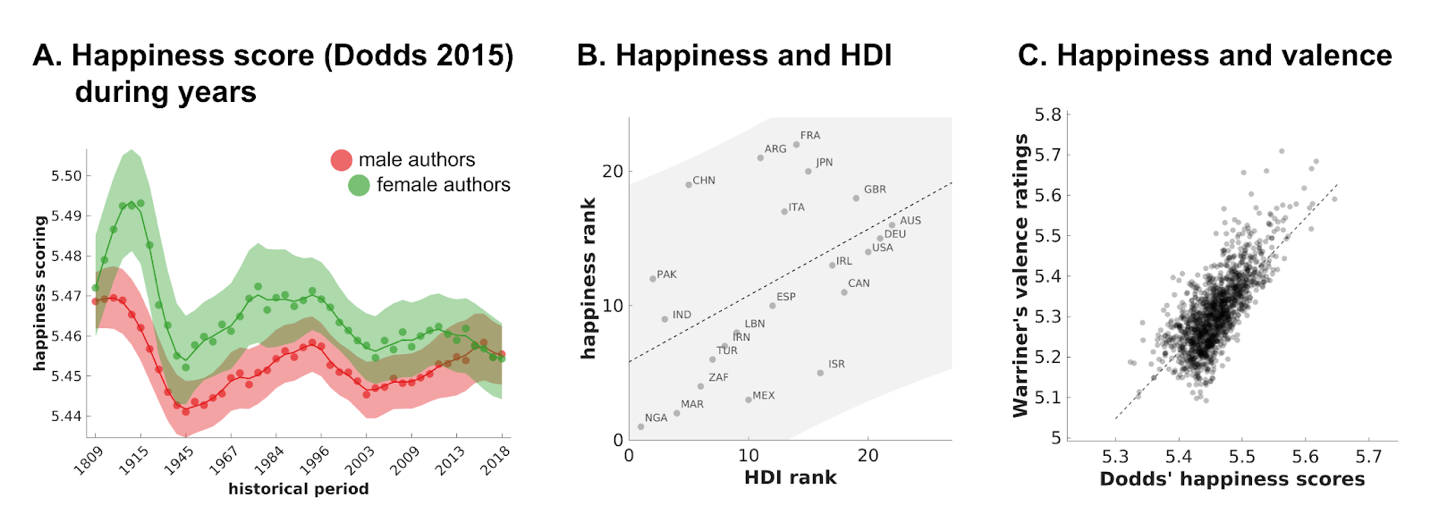


**Fig. S4.** Panel a represents the score of Happiness using ratings of Dodds and colleagues evaluated across authors and historical periods. Since Dodd’s dataset did not include raters’ sex, the happiness scores were measured in male and female writers using the same values. The trend in time and across sexes mimicked the one reported in Figure 3e. Shaded areas represent 95% confidence intervals of the estimation across authors. In Panel b, we test the relationship between the Happiness score and the HDI between countries. HDI was positively associated with Happiness (ρ = 0.494, p = 0.0207, CI95: 0.096 0.771). In Panel c, we report the similarity between the author’s affective scorings measured in Dodds and Warriner datasets (ρ = 0.796, p < 0.0001, CI95: 0.773 0.816).

**
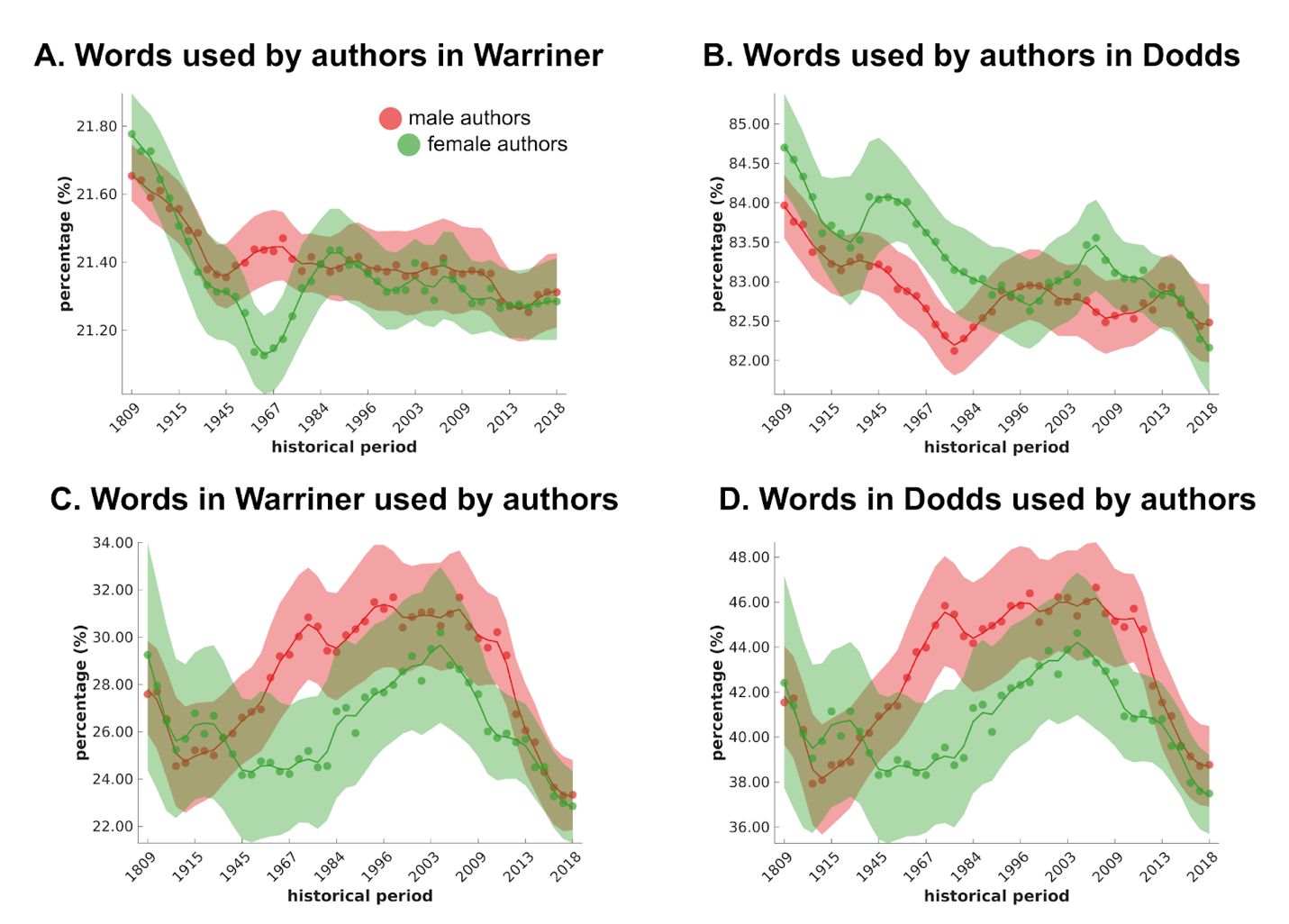
**

**Fig. S5.** For affective words in Warriner (2013) and Dodds (2015) datasets, we measured the stability of frequency in time and across sexes. A stable coverage of these dictionaries limits the possibility that estimates of the affective components of words, including the sentiment analysis of authors’ writings, are biased. The figure depicts the percentage of words that were present in Warriner’s and Dodds’ datasets through time and across sex. In Panels a and b, we measure the percentage of words used by our writers included in the two dictionaries. Both datasets exhibit a negligible negative trend in time with a decrease of ~2% in modern times. The Dodds dictionary was conceived to maximize language coverage, reaching ~84%. In Panels c and d, we also report the percentage of words in the dictionary which were retrieved in the writings. Shaded areas represent 95% confidence intervals of the estimation across authors.


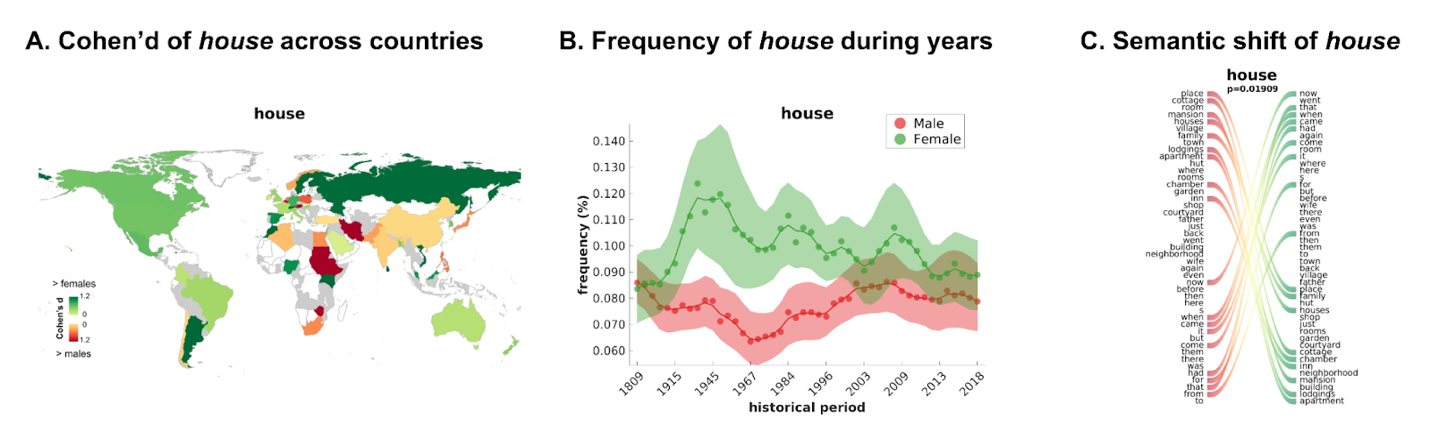


**Fig. S6.** Panel a shows an exemplar world map of Cohen’s d of the sex effect of the word *house*. Panel b represents the trend in time of the raw frequency for *house*, similarly to Google Ngram Viewer, but reporting the use frequency in male and female authors separately, as well as the 95% confidence intervals (i.e., shaded areas) of the estimation across authors. Panel c reports the semantic neighbors of the word *house*, as well as their rearrangements due to the effect of sex through a Sankey plot. The left column shows the neighbors of *house* for male authors, arranged from the most to the least related. The right column depicts the same procedure for females. Ribbons connect words that sustain a relatively high local shift between the two sexes. The information on these three panels is available for each significant word at <https://www.sane-elab.eu/litemo/welcome.php>.

**
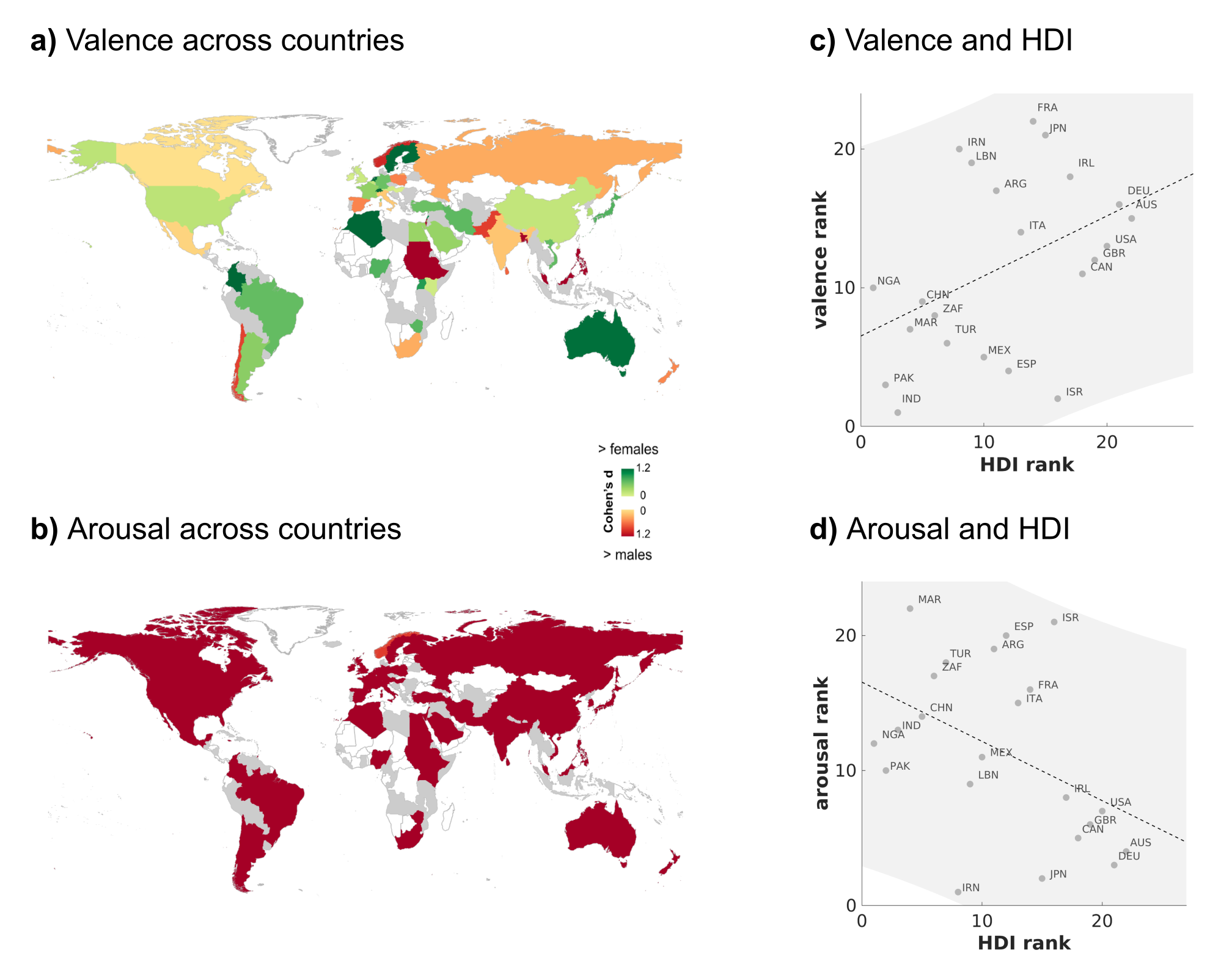
**

**Fig. S7.** Maps of sex differences between male and female writers in valence (a) and arousal (b). Panels c and d show the across countries association between the human development index and valence or arousal, respectively. Affective dimensions obtained from the authors’ writings were averaged and correlated with the HDI measure for each country. Results demonstrate a positive correlation with valence (i.e., higher HDI countries have writers using more positive words) and a negative one with arousal (i.e., higher HDI countries have writers using words with lower arousal).


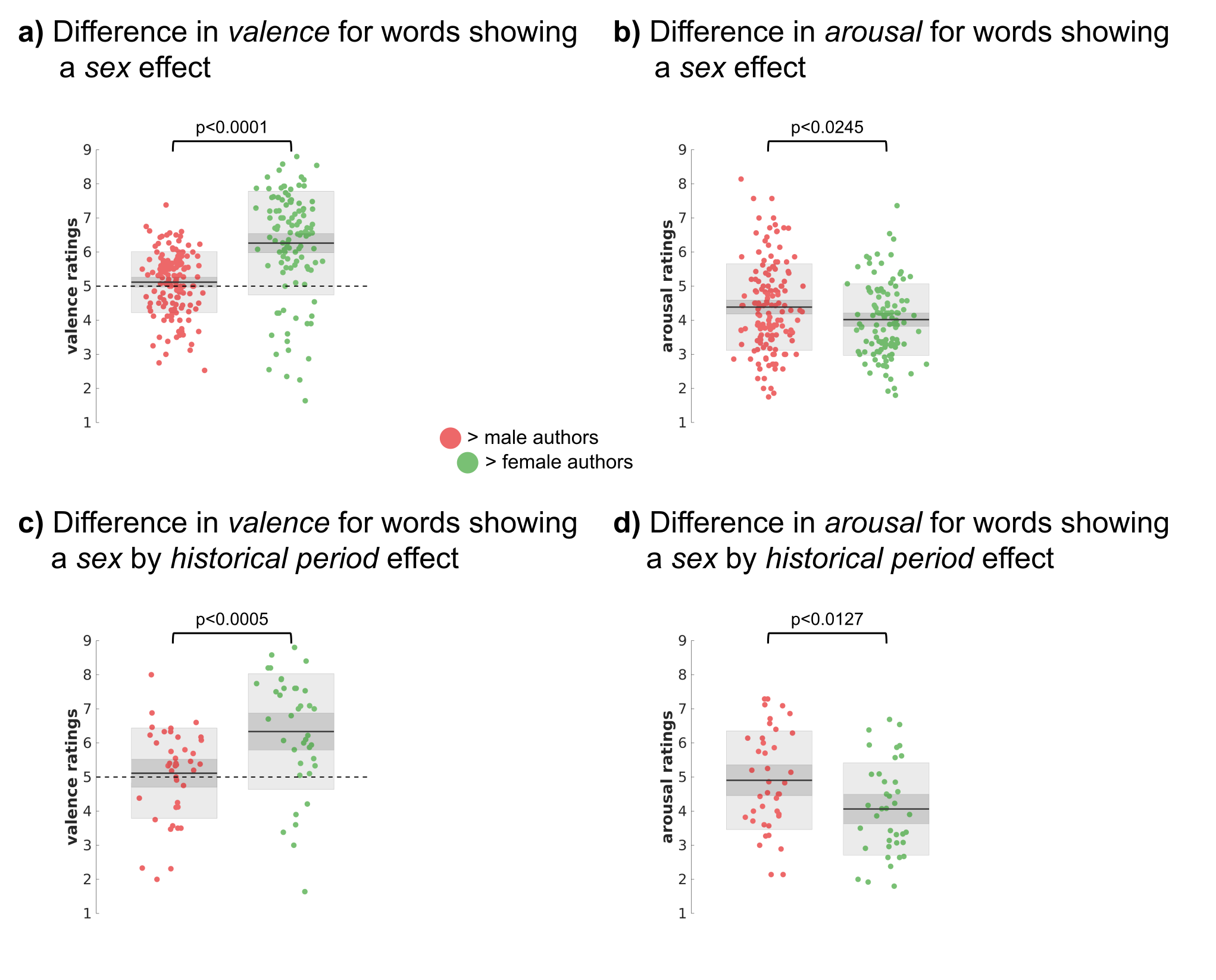


**Fig. S8.** Valence and arousal of terms showing sex and sex by historical period differences. Panels a and b show that female authors use more positive and less arousing terms. In box plots each dot is a word showing a significant sex effect also present in the Warriner database (n = 265), and the dark-gray shaded area shows the 95% confidence intervals of the SE of the mean, while the light-gray area is the SD. Panels c and d show that female authors use more positive and less arousing terms, with a significant effect of sex by historical period. In box plots each dot is a word showing a significant sex by historical period effect also present in the Warriner database (n = 78), and the dark-gray shaded area shows the 95% confidence intervals of the SE of the mean, while the light-gray area is the SD.

Tables related to our 576 significant terms (S1) and composition of the corpus (S2) are available as extended data and also here at: <https://osf.io/mcx5a/>.

**Table S4. Benchmarks of word embeddings.**

| **Benchmarks of word embeddings** | | | | |
| --- | --- | --- | --- | --- |
| **Test** | **Reference** | **Correlation Coefficient** | **Accuracy in %** | **Coverage in %** |
| **RG-65** | *Rubenstein and Goodenough, 1965* | 0.871 |  | 100.0 |
| **WordSimilarity-353** | *Finkelstein et al., 2001* | 0.626 |  | 98.6 |
| **MEN dataset** | *Bruni et al., 2014* | 0.754 |  | 100.0 |
| **TOEFL Synonym Questions** | *Landauer and Dumais, 1997* |  | 85.9 | 88.8 |
| **BLIND eng** | *Lenci et al., 2013* | 0.712 |  | 100.0 |
| **McRAE** | *McRae et al., 2005* | 0.485 |  | 98.7 |
| **VINSON** | *Vinson & Vigliocco, 2008* | 0.582 |  | 99.6 |
| **SIMLEX-999** | *Reichart & Korhonen, 2015* | 0.451 |  | 99.9 |
| **MTURK-771** | *Halawi et al., 2012* | 0.647 |  | 99.7 |
| **WORD NORMS** | *Buchanan et al., 2013* | 0.379 |  | 98.8 |
|  |  |  |  |  |
|  |  |  |  |  |
|  |  |  |  |  |
|  |  |  |  |  |
| **REFERENCES** | | | | |
| *Rubenstein, H., & Goodenough, J. B. (1965). Contextual correlates of synonymy. Communications of the ACM, 8(10), 627-633.* | | | | |
| *Finkelstein, L., Gabrilovich, E., Matias, Y., Rivlin, E., Solan, Z., Wolfman, G., & Ruppin, E. (2001, April). Placing search in context: The concept revisited. In Proceedings of the 10th international conference on World Wide Web (pp. 406-414).* | | | | |
| *Bruni, E., Tran, N. K., & Baroni, M. (2014). Multimodal distributional semantics. Journal of artificial intelligence research, 49, 1-47.* | | | | |
| *Landauer, T. K., & Dumais, S. T. (1997). A solution to Plato's problem: The latent semantic analysis theory of acquisition, induction, and representation of knowledge. Psychological review, 104(2), 211.* | | | | |
| Lenci, A., Baroni, M., Cazzolli, G., & Marotta, G. (2013). BLIND: A set of semantic feature norms from the congenitally blind. Behavior research methods, 45(4), 1218-1233. | | | | |
| *McRae, K., Cree, G. S., Seidenberg, M. S., & McNorgan, C. (2005). Semantic feature production norms for a large set of living and nonliving things. Behavior research methods, 37(4), 547-559.* | | | | |
| *Vinson, D. P., & Vigliocco, G. (2008). Semantic feature production norms for a large set of objects and events. Behavior Research Methods, 40(1), 183-190.* | | | | |
| *Hill, F., Reichart, R., & Korhonen, A. (2015). Simlex-999: Evaluating semantic models with (genuine) similarity estimation. Computational Linguistics, 41(4), 665-695.* | | | | |
| *Halawi, G., Dror, G., Gabrilovich, E., & Koren, Y. (2012, August). Large-scale learning of word relatedness with constraints. In Proceedings of the 18th ACM SIGKDD international conference on Knowledge discovery and data mining (pp. 1406-1414).* | | | | |
| *Buchanan, E. M., Valentine, K. D., & Maxwell, N. P. (2019). English semantic feature production norms: An extended database of 4436 concepts. Behavior Research Methods, 51(4), 1849-1863.* | | | | |

**Table S5. References for the selection of diachronic words.**

| **Authors** | **Reference** |
| --- | --- |
| Bamler et al. (2017) | Bamler, R., & Mandt, S. (2017). Dynamic word embeddings. In International conference on Machine learning (pp. 380-389). PMLR. |
| Banks (2004) | Banks, M. A., "Semantic Changes in Present-Day English (PDE)" (2004). McCabe Thesis Collection. Paper 25. |
| Boukhaled et al. (2019) | Boukhaled, M., Fagard, B., & Poibeau, T. (2019). Modelling the semantic change dynamics using diachronic word embedding. In 11th International Conference on Agents and Artificial Intelligence (NLPinAI Special Session). |
| Curzane (2014) | Curzane A. (2014). What makes a word "real"? TED Conferences. https://www.ted.com/talks/anne_curzan_what_makes_a_word_real |
| Delpech (2018) | Delpech, J. (2018). Unsupervised detection of diachronic word sense evolution. ArXiv, abs/1805.11295. |
| Duan et al. (2021) | Duan, Y., Jatowt, A., Yoshikawa, M., Liu, X., Matono, A. (2021). Diachronic Linguistic Periodization of Temporal Document Collections for Discovering Evolutionary Word Semantics. In: Ke, HR., Lee, C.S., Sugiyama, K. (eds) Towards Open and Trustworthy Digital Societies. ICADL 2021. Lecture Notes in Computer Science, vol 13133. Springer, Cham. https://doi.org/10.1007/978-3-030-91669-5_1 |
| Finegan (2014) | Finegan, E. (2014). Language: Its structure and use. Cengage Learning. |
| Hamilton et al. (2016) | Hamilton, W.L., Leskovec, J., & Jurafsky, D. (2016). Diachronic Word Embeddings Reveal Statistical Laws of Semantic Change. ArXiv, abs/1605.09096. |
| Hamilton et al. (2016) | Hamilton, W. L., Leskovec, J., & Jurafsky, D. (2016). Cultural shift or linguistic drift? comparing two computational measures of semantic change. In Proceedings of the Conference on Empirical Methods in Natural Language Processing. Conference on Empirical Methods in Natural Language Processing (Vol. 2016, p. 2116). NIH Public Access. |
| Jones (2019) | Jones, P. A. (2017). The Accidental Dictionary: The Remarkable Twists and Turns of English Words. Pegasus Books. |
| Kulkarni et al. (2015) | Kulkarni, V., Al-Rfou, R., Perozzi, B., & Skiena, S. (2015). Statistically significant detection of linguistic change. In Proceedings of the 24th international conference on world wide web (pp. 625-635). |
| McWhorter (2016) | McWhorter, J. (2016). 4 reasons to learn a new language. TED Conferences. https://www.ted.com/talks/john_mcwhorter_4_reasons_to_learn_a_new_language |
| Qiu et al. (2022) | Qiu, Wenjun & Xu, Yang. (2022). HistBERT: A Pre-trained Language Model for Diachronic Lexical Semantic Analysis. 10.13140/RG.2.2.14905.44649. |
| Tsakalidis et al. (2021) | Tsakalidis, A., Basile, P., Bazzi, M. et al. (2021). DUKweb, diachronic word representations from the UK Web Archive corpus. Sci Data 8, 269. https://doi.org/10.1038/s41597-021-01047-x |
| Vijayarani & Geetha (2020) | Vijayarani, J., Geetha, T.V. (2020). Knowledge-enhanced temporal word embedding for diachronic semantic change estimation. Soft Comput 24, 12901–12918. https://doi.org/10.1007/s00500-020-04714-0 |

**References**

Benjamini, Y., & Hochberg, Y. (1995). Controlling the false discovery rate: a practical and powerful approach to multiple testing. *Journal of the Royal statistical society: series B (Methodological)*, *57*(1), 289-300.

Bonini, A. N. (2008). Cross-national variation in individual life satisfaction: Effects of national wealth, human development, and environmental conditions. Social indicators research, 87, 223-236.

Dodds, P. S., Clark, E. M., Desu, S., Frank, M. R., Reagan, A. J., Williams, J. R., ... & Danforth, C. M. (2015). Human language reveals a universal positivity bias. Proceedings of the national academy of sciences, 112(8), 2389-2394.

Hamilton, W. L., Leskovec, J., & Jurafsky, D. (2016a). Diachronic word embeddings reveal statistical laws of semantic change. *arXiv preprint arXiv:1605.09096*.

Hamilton, W. L., Leskovec, J., & Jurafsky, D. (2016b). Cultural shift or linguistic drift? comparing two computational measures of semantic change. In *Proceedings of the conference on empirical methods in natural language processing. Conference on empirical methods in natural language processing* (Vol. 2016, p. 2116). NIH Public Access.

Jockers, M. L. (2013). Macroanalysis: Digital methods and literary history. University of Illinois Press.

Lazer, D., Hargittai, E., Freelon, D., Gonzalez-Bailon, S., Munger, K., Ognyanova, K., & Radford, J. (2021). Meaningful measures of human society in the twenty-first century. Nature, 595(7866), 189-196.

Michel, J. B., Shen, Y. K., Aiden, A. P., Veres, A., Gray, M. K., Google Books Team, ... & Aiden, E. L. (2011). Quantitative analysis of culture using millions of digitized books. *Science*, 331(6014), 176-182.

Moretti, F. (1999). Atlas of the European novel: 1800-1900. verso.

Pechenick, E. A., Danforth, C. M., & Dodds, P. S. (2015). Characterizing the Google Books corpus: Strong limits to inferences of socio-cultural and linguistic evolution. *PloS one*, 10(10), e0137041.

Popescu, Ioan-Iovitz. Word frequency studies. De Gruyter Mouton, 2009.

Twenge, J. M., Campbell, W. K., & Gentile, B. (2012). Male and female pronoun use in US books reflects women’s status, 1900–2008. *Sex roles*, 67(9), 488-493.

Underwood, T., Bamman, D., & Lee, S. (2018). The transformation of gender in English-language fiction. *Journal of Cultural Analytics*, 3(2), 11035.

Verboord, M. (2012). Female bestsellers: A cross-national study of gender inequality and the popular–highbrow culture divide in fiction book production, 1960–2009. *European Journal of Communication*, 27(4), 395-409.

Warriner, A.B., Kuperman, V., & Brysbaert, M. (2013). Norms of valence, arousal, and dominance for 13,915 English lemmas. Behavior Research Methods, 45, 1191-1207.

Williams, J. R., Bagrow, J. P., Danforth, C. M., & Dodds, P. S. (2015). Text mixing shapes the anatomy of rank-frequency distributions. Physical Review E, 91(5), 052811.

Zipf, G. K. (1949). Human behavior and the principle of least effort: An introduction to human ecology.

Zwar, J., Throsby, D., & Longden, T. (2015). Demographics of Australian book authors. (Australian authors Industry Briefs; No. 2). Macquarie University.
